# Double hybrid DFT calculations with Slater type orbitals

**DOI:** 10.1002/jcc.26209

**Published:** 2020-04-16

**Authors:** Arno Förster, Lucas Visscher

**Affiliations:** ^1^ Theoretical Chemistry Vrije Universiteit Amsterdam The Netherlands

**Keywords:** ADF, benchmark, double‐hybrid, KS‐DFT, STO

## Abstract

On a comprehensive database with 1,644 datapoints, covering several aspects of main‐group as well as of transition metal chemistry, we assess the performance of 60 density functional approximations (DFA), among them 36 double hybrids (DH). All calculations are performed using a Slater type orbital (STO) basis set of triple‐*ζ* (TZ) quality and the highly efficient pair atomic resolution of the identity approach for the exchange‐ and Coulomb‐term of the KS matrix (PARI‐*K* and PARI‐*J*, respectively) and for the evaluation of the MP2 energy correction (PARI‐MP2). Employing the quadratic scaling SOS‐AO‐PARI‐MP2 algorithm, DHs based on the spin‐opposite‐scaled (SOS) MP2 approximation are benchmarked against a database of large molecules. We evaluate the accuracy of STO/PARI calculations for B3LYP as well as for the DH B2GP‐PLYP and show that the combined basis set and PARI‐error is comparable to the one obtained using the well‐known def2‐TZVPP Gaussian‐type basis set in conjunction with global density fitting. While quadruple‐*ζ* (QZ) calculations are currently not feasible for PARI‐MP2 due to numerical issues, we show that, on the TZ level, Jacob's ladder for classifying DFAs is reproduced. However, while the best DHs are more accurate than the best hybrids, the improvements are less pronounced than the ones commonly found on the QZ level. For conformers of organic molecules and noncovalent interactions where very high accuracy is required for qualitatively correct results, DHs provide only small improvements over hybrids, while they still excel in thermochemistry, kinetics, transition metal chemistry and the description of strained organic systems.

## Graphical abstract

The authors assess the accuracy of 60 density functional approximations, including 36 double hybrid functionals, using Slater type orbitals. Employing triple‐zeta quality, Basis set errors are comparable to the ones obtained from standard Gaussian type orbital basis sets. For a large data set of 1,644 data points, Jacob's ladder is reproduced also on the triple‐zeta level. Using a modern SOS‐MP2 algorithm, very large double hybrid calculations do not require much more computational time than hybrid calculations.

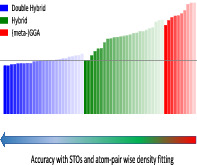

## 1 INTRODUCTION

Due to its unrivaled price/performance‐ratio,^[^ ^1^ ^]^ Kohn‐Sham (KS)^[^ ^2^ ^]^ density functional theory (DFT),^[^ 3, 4, 5 ^]^ is the workhorse of modern computational chemistry.^[^ 6, 7, 8, 9 ^]^ Despite recent tremendous progress in wave function (WF) theory, especially in the field of local Coupled Cluster (CC)^[^ 10, 11, 12, 13 ^]^ methods,^[^ 14, 15, 16, 17, 18, 19, 20, 21, 22, 23, 24, 25, 26, 27, 28, 29, 30, 31, 32, 33, 34, 35, 36, 37, 38, 39, 40, 41 ^]^ little doubt exist that this situation will not change in the foreseeable future.

No satisfactory way to systematically improve the exchange‐correlation (XC) functional of KS‐DFT is known^[^ ^42^ ^]^ and in practice approximations are necessary. Density functional approximations (DFA) explicitly depending on both, the electron density and one‐electron orbitals resolve many deficiencies of (meta‐)generalized gradient approximations (GGA).^[^ 5, 43, 44, 45, 46, 47 ^]^ If only the exchange part of such DFAs depends on these orbitals, they are referred to as hybrid functionals. For an orbital‐dependent treatment of correlation based on the KS reference Hamiltonian, a dependence of the DFA on virtual orbitals must be introduced. A particularly economic strategy to do so is via second order Møller–Plesset perturbation theory (MP2). As both, the exchange‐ and the correlation part of such DFAs are bridging standard DFAs and WF theory, they are commonly referred to as double‐hybrid (DH)^[^ 48, 49, 50, 51, 52, 53, 54, 55 ^]^ functionals.

According to the famous Jacob's ladder classification scheme for DFAs,^[^ ^56^ ^]^ the generally most accurate and robust functionals should be DH‐DFAs. Indeed, rigorous validation on the comprehensive GMTKN55^[^ ^57^ ^]^ database by Goerigk, Grimme, Martin and coworkers has substantiated this conjecture.^[^ 57, 58, 59, 60, 61 ^]^ Unfortunately, blending WF theory into KS‐DFT not only goes along with increased accuracy but also with increased computational cost. The calculation of the KS matrix for hybrid functionals is commonly associated with a *N* ^4^ scaling operation count,^[^ ^62^ ^]^ and the CPU time for the evaluation of the canonical MP2 correlation energy increases with *N* ^5^ as a function of system size *N*.^[^ ^63^ ^]^

Given these scaling properties, the availability of cost‐effective algorithms for both tasks is crucial to enable routine application of hybrid‐ and DH‐DFAs to large molecules. For both, the Coulomb (*J*) and the exchange (*K*)‐term, exploiting sparsity in the rank‐4 electron repulsion integral (ERI) tensor is key to reducing the computational cost of the KS matrix construction for hybrid functionals. Algorithms based on integral prescreening techniques^[^ 64, 65, 66, 67, 68, 69, 70 ^]^ offer favorable scalability but suffer from a high prefactor. To circumvent this issue, modern codes additionally rely on density‐fitting (DF)^[^ 71, 72, 73, 74, 75, 76, 77, 78, 79, 80, 81, 82 ^]^ approximations being an efficient approach to evaluate the *J*‐term, especially in conjunction with the *J*‐engine technique.^[^ 83, 84, 85, 86, 87 ^]^ Global density fitting is less efficient for the *K*‐term and promising approaches to achieve better performance are the pseudo‐spectral method,^[^ 88, 89, 90, 91, 92, 93 ^]^ the auxiliary density matrix method^[^ 94, 95, 96 ^]^ or different flavors of local DF (LDF)^[^ 97, 98, 99 ^]^ approximations.^[^ ^100^ ^]^ The most promising variant of the latter approach might be the pair‐atomic resolution of the identity (PARI) approximation^[^ 101, 102, 103 ^]^ (Known as PARI‐*K* when applied to the *K*‐term^[^ ^103^ ^]^). In this most extreme variant of LDF methods, following the treatment of Baerends et al.^[^ ^71^ ^]^ for the *J*‐term, each pair product of AOs is expanded in a set of auxiliary basis functions centered on the same two atoms as the target pair of primitives.

To accelerate the evaluation of MP2 energies, DF is the only approach which has found widespread use.^[^ 104, 105, 106, 107, 108, 109, 110, 111, 112, 113, 114, 115 ^]^ Reducing the prefactor of canonical implementations by at least one order of magnitude, it retains their *N* ^5^ scaling. However, relying on the short‐rangedness of dynamical electron correlation,^[^ 116, 117, 118, 119 ^]^ many reduced‐scaling MP2 algorithms, employing localized molecular orbitals (MO),^[^ 120, 121, 122 ^]^ relying on fragmentation approaches,^[^ 113, 114, 123, 124 ^]^ or evaluating the MP2 energy in the AO basis and exploiting sparsity in the ERI tensor^[^ 125, 126, 127, 128, 129, 130, 131, 132, 133, 134, 135, 136, 137, 138, 139, 140, 141, 142, 143 ^]^ have been developed.

Following the latter approach, we have shown that the PARI approach can not only be used to accelerate the KS matrix construction but also to efficiently compute MP2 correlation energies.^[^ ^144^ ^]^ While we have demonstrated that an MO based PARI‐MP2 algorithm can easily compete with DF‐MP2 in terms of accuracy, we have also formulated the Spin opposite‐scaled^[^ ^145^ ^]^ (SOS)‐AO‐PARI‐MP2 algorithm, enabling to obtain accurate SOS‐MP2 energies with quadratic scaling operation count. Implemented in the STO‐based Amsterdam density functional (ADF)^[^ 79, 146, 147 ^]^ code, the SCF, although extensively accelerated via PARI‐*K*, represents the bottleneck (both CPU time and memory‐wise) for single‐point SOS‐MP2 calculations on molecules of several hundreds of atoms using basis sets of TZ quality. We concluded that this enables quantum chemists to carry out DH calculations whenever a hybrid calculation is feasible too.

Employing any of the mentioned local approximations might lead to tremendous speed‐up to the price of an additional source of error. For an approximation to be useful in practice, the introduced errors must be small and well controlled and the inequalities

(1) $$\Delta_{m}>\Delta_{b}>>\Delta_{a}\;,$$

where Δ_*m*_, Δ_*b*_, and Δ_*a*_ denote the inherent error of the method, basis set and additional approximations, respectively, should be fulfilled. The latter inequality is of high importance, as in most quantum‐chemistry packages algorithmic details will differ and it is often non‐obvious to users how to tweak them. In this sense, the second inequality is crucial to make comparisons of results between different codes meaningful.

Even for very small Δ_*m*_ and Δ_*b*_, (1) is certainly met by global DF approximations. Is this also the case for PARI? Concerns regarding robustness^[^ 103, 148, 149 ^]^ and accuracy^[^ ^100^ ^]^ of PARI‐*K*, especially for the description of virtual orbital energies,^[^ 100, 150 ^]^ have recently been expressed. Additionally, the exact exchange energy from the PARI‐*K* algorithm is unbounded from below which might result in convergence to artificial low‐lying states.^[^ 103, 149 ^]^ This should not be confused with errors due to nonconvergence of the SCF, an algorithm for which convergence is not guaranteed.^[^ 151, 152, 153 ^]^ The appearance of fully converged, but unphysical results is much more problematic. These shortcomings have been found to get more pronounced with increasing number of AOs and molecular size.^[^ ^100^ ^]^ Hybrid calculations with a QZ basis are unreliable for large molecules. For PARI‐MP2, additional difficulties arise in the fitting of virtual orbitals,^[^ ^144^ ^]^ leading to even larger numerical instabilities with increased number of primitives. Effectively, this limits the largest basis sets which can be safely be used to TZ quality with a moderate number of diffuse functions. In practice, this might be the most severe limitation for DH calculations. Although some exceptions are known,^[^ ^154^ ^]^ TZ basis sets are usually sufficient to fulfill the first inequality in (1) for GGAs and hybrids.^[^ ^155^ ^]^ Due to the exceptionally slow convergence to the CBS limit of MP2,^[^ 156, 157, 158, 159, 160, 161 ^]^ considerably larger basis sets are needed for DHs.^[^ ^162^ ^]^

As it only requires the evaluation of a subset of two‐center Coulomb integrals, PARI is a crucial technique for efficient exact exchange algorithms in quantum chemistry packages based on Slater type orbitals (STO) or numerical atomic orbitals (NAOs)^[^ 163, 164, 165, 166 ^]^ relying on numerical integration techniques. Therefore, it is of utmost importance to investigate whether accurate, robust and efficient hybrid‐ and DH‐KS calculations can be performed within the PARI‐approximation. The answer immediately implies the answer to a far more important question—is it possible to perform accurate, robust and efficient hybrid‐ and DH KS calculations using STOs? To the best of our knowledge, a final answer has not been given yet. Although we recently presented accurate PARI‐MP2 energies using TZ basis sets,^[^ ^144^ ^]^ a systematic study on large databases is yet to be conducted for hybrid‐ and DH‐DFAs.

Different DFAs show different dependencies on the choice of basis set and numerical approximations.^[^ 154, 167 ^]^ The superior performance of a certain DFA under ideal conditions that is, using large basis sets and very small Δ_*a*_ does not necessarily imply the same behavior when smaller basis sets and PARI are employed. Employing these ideal conditions, in the last years large numbers of DFAs have been ranked according to their performance on large databases.^[^ 57, 58, 59, 154, 168 ^]^ It is certainly of great interest to investigate to what extend these rankings can be reproduced using STOs and PARI (STO/PARI in the following).

Against this background, we herein present benchmark calculations with 60 DFAs using a comprehensive database consisting of 1,644 data points, covering both, main‐group and transition metal (TM) chemistry. This is the first comprehensive benchmark of DFAs using PARI and, to the best of our knowledge, the first one using STOs.

This paper is organized as follows: After giving an overview of our database and of the herein benchmarked DFAs we shorty describe our computational approach and give representative timings before we discuss the results of our benchmarks. We will investigate the accuracy of the STO/PARI approach in comparison to GTO‐type basis sets for two exemplary DFAs and subsequently present benchmarks for all 60 assessed functionals. In the end, we conclude and summarize this work.

## 2 METHODS

### 2.1 Test sets

Large compilations of diverse datasets covering a wide range of main‐group chemistry have now a long‐standing tradition in quantum chemistry. The importance of meta‐databases like GMTKN55^[^ ^57^ ^]^ (and its predecessors GMTKN24^[^ ^169^ ^]^ and GMTKN30^[^ ^162^ ^]^) and MGCDB84^[^ ^168^ ^]^ compiled by Grimme and coworkers and Head‐Gordon and coworkers, respectively, to assess the performance of DFAs and also wave‐function methods^[^ ^170^ ^]^ can barely be overemphasized.

In this work, we benchmark the DFAs in Table 1 against a comprehensive database comprised of 58 subsets, organized in five subcategories of different types of chemical problems with 1,644 data points in total. Additionally, we benchmark all SOS‐MP2 based DHs as well as selected hybrids against a sixth subdatabase comprised of five test sets of large organic molecules with a total of 85 data points.

**TABLE 1:** List of DFAs benchmarked in this work. The functional name is given together with the amount of HF‐exchange (*a*_*x*_ in (2)) and MP2 correlation energy (*a*_*c*, *os*_/*a*_*c*, *ss*_ in (2), if only one number is given, *a*_*c*, *os*_ = *a*_*c*, *ss*_) as well as the variants of empirical dispersion correction with which it is combined. The corresponding parameters used herein are given in the ESI.

Functional	*a*_*x*_	*a*_*c*_	Dispersion correction
GGA			
PBE^[^ 171, 172 ^]^			D3(BJ)^[^ ^173^ ^]^/D4^[^ ^174^ ^]^
BLYP^[^ 175, 176, 177 ^]^			D3(BJ)^[^ ^173^ ^]^/D4^[^ ^174^ ^]^
REVPBE^[^ ^178^ ^]^			D3(BJ)^[^ ^173^ ^]^/D4^[^ ^174^ ^]^
Meta‐GGA			
TPSS^[^ 179, 180 ^]^			D3(BJ)^[^ ^173^ ^]^/D4^[^ ^181^ ^]^
revTPSS^[^ 182, 183 ^]^			D3(BJ)^[^ ^57^ ^]^/D4^[^ ^174^ ^]^
SCAN^[^ ^184^ ^]^			D3(BJ)^[^ ^185^ ^]^/D4^[^ ^174^ ^]^
Hybrid			
B97^[^ ^186^ ^]^	0.19		D3(0)^a^/D4^[^ ^174^ ^]^
B3LYP^[^ ^187^ ^]^	0.2		D3(BJ)^[^ ^173^ ^]^/D4^[^ ^181^ ^]^
mPW1B95^[^ ^188^ ^]^	0.31		D3(BJ)^[^ ^189^ ^]^
mPWB1K^[^ ^188^ ^]^	0.44		D3(BJ)^[^ ^189^ ^]^/D4^[^ ^174^ ^]^
mPW1PW	0.25		D3(BJ)^a^
PBE0^[^ 190, 191 ^]^	0.25		D3(BJ)^[^ ^173^ ^]^/D4^[^ ^181^ ^]^
SOGGA11‐X^[^ ^192^ ^]^	0.40		D3(BJ)^a^
TPSSH^[^ ^193^ ^]^	0.10		D3(BJ)^[^ ^189^ ^]^/D4^[^ ^174^ ^]^
PWB6K^[^ ^194^ ^]^	0.46		D3(0)
PW6B95^[^ ^194^ ^]^	0.28		D3(0)^[^ ^173^ ^]^/D4^[^ ^174^ ^]^
CAM‐B3LYP	r.s.		D3(BJ)^[^ ^189^ ^]^/D4^[^ ^174^ ^]^
*ω*B97‐X^[^ ^195^ ^]^	r.s.		D3(0)^a^
M05‐2X^[^ ^196^ ^]^	0.56		D3(0)^a^
M06^[^ ^197^ ^]^	0.27		D3(0)^a^/D4^[^ ^174^ ^]^
M06‐2X^[^ ^197^ ^]^	0.54		D3(0)^a^
M08‐HX^[^ ^198^ ^]^	0.52		D3(0)^a^
M08‐SO^[^ ^198^ ^]^	0.57		D3(0)^a^
MN12‐SX^[^ ^199^ ^]^	r.s.		D3(BJ)^a^
DH			
B2‐PLYP^[^ ^49^ ^]^	0.53	0.27	D3(BJ)^[^ ^189^ ^]^/D4^[^ ^174^ ^]^
B2GP‐PLYP^[^ ^200^ ^]^	0.65	0.36	D3(BJ)^[^ ^189^ ^]^/D4^[^ ^174^ ^]^
B2K‐PLYP^[^ ^55^ ^]^	0.72	0.42	D3(BJ)^b^
B2T‐PLYP^[^ ^55^ ^]^	0.60	0.31	D3(BJ)^b^
B2*π*‐PLYP^[^ ^201^ ^]^	0.60	0.27	D3(BJ)^b^
B2NC‐PLYP^[^ ^202^ ^]^	0.67	0.49	D3(BJ)^[^ ^58^ ^]^
mPW2‐PLYP^[^ ^50^ ^]^	0.55	0.25	D3(BJ)^[^ ^57^ ^]^
mPW2K‐PLYP^[^ ^55^ ^]^	0.72	0.42	D3(BJ)^b^
mPW2NC‐PLYP^[^ ^202^ ^]^	0.67	0.49	D3(BJ)^[^ ^58^ ^]^
DH‐BLYP^[^ ^203^ ^]^	0.65	0.42	D3(BJ)^b^
PBE0‐DH^[^ ^204^ ^]^	0.5	0.125	D3(BJ)^[^ ^205^ ^]^/D4^[^ ^174^ ^]^
PBE0‐2^[^ ^206^ ^]^	0.79	0.5	D3(BJ)^[^ ^205^ ^]^/D4^[^ ^174^ ^]^
LS1‐DH^[^ ^207^ ^]^	0.75	0.42	D3(BJ)^[^ ^58^ ^]^
LS1‐TPSS^[^ ^208^ ^]^	0.85	0.61	D3(BJ)^[^ ^58^ ^]^
DS1‐TPSS^[^ ^208^ ^]^	0.73	0.53	D3(BJ)^2^
PBE‐QIDH^[^ ^209^ ^]^	0.69	0.33/0.33	D3(BJ)^[^ ^210^ ^]^
SOS1‐PBE‐QIDH^[^ ^211^ ^]^	0.69	0.44/0.00	D3(BJ)^[^ ^210^ ^]^
SD‐SCAN69^[^ ^60^ ^]^	0.69	0.62/0.24	c
DOD‐SCAN^[^ ^60^ ^]^	0.66	0.63/0.00	D3(BJ)^[^ ^60^ ^]^
revDSD‐SCAN‐D4^[^ ^60^ ^]^	0.66	0.63/0.01	D4^[^ ^60^ ^]^
revDOD‐SCAN‐D4^[^ ^60^ ^]^	0.66	0.63/0.00	D4^[^ ^60^ ^]^
DSD‐BLYP^[^ ^212^ ^]^	0.69	0.46/0.37	D3(BJ)^[^ ^189^ ^]^
revDSD‐BLYP^[^ ^60^ ^]^	0.71	0.55/0.20	D3(BJ)^[^ ^60^ ^]^
revDOD‐BLYP^[^ ^60^ ^]^	0.71	0.62/0.00	D3(BJ)^[^ ^60^ ^]^
revDSD‐BLYP‐D4^[^ ^60^ ^]^	0.71	0.56/0.20	D4^[^ ^60^ ^]^
revDOD‐BLYP‐D4^[^ ^60^ ^]^	0.71	0.63/0.00	D4^[^ ^60^ ^]^
DSD‐PBEP86^[^ ^213^ ^]^	0.69	0.52/0.22	D3(BJ)^[^ ^213^ ^]^
revDSD‐PBEP86^[^ ^60^ ^]^	0.69	0.58/0.08	D3(BJ)^[^ ^60^ ^]^
revDOD‐PBEP86^[^ ^60^ ^]^	0.69	0.61/0.00	D3(BJ)^[^ ^60^ ^]^
revDSD‐PBEP86‐D4^[^ ^60^ ^]^	0.69	0.59/0.06	D4^[^ ^60^ ^]^
revDOD‐PBEP86‐D4^[^ ^60^ ^]^	0.69	0.61/0.00	D4^[^ ^60^ ^]^
DSD‐PBE^[^ 213, 214 ^]^	0.68	0.55/0.13	D3(BJ)^[^ ^214^ ^]^
revDSD‐PBE^[^ ^60^ ^]^	0.68	0.58/0.07	D3(BJ)^[^ ^60^ ^]^
revDOD‐PBE^[^ ^60^ ^]^	0.68	0.61/0.00	D3(BJ)^[^ ^60^ ^]^
revDSD‐PBE‐D4^[^ ^60^ ^]^	0.68	0.60/0.04	D4^[^ ^60^ ^]^
revDOD‐PBE‐D4^[^ ^60^ ^]^	0.68	0.62/0.00	D4^[^ ^60^ ^]^

a PBE‐parameters have been used.

b Parametrization in this work.

c Not evaluated with empirical dispersion correction.

We do not present any new database or test set in this work. Instead, we selected nearly all subsets from GMTKN55^[^ ^57^ ^]^ and MGCDB84^[^ ^168^ ^]^ and additionally include two databases featuring energies and barrier heights of reactions between TM containing species. An overview of all categories in which we organize our database is given in Table 2, while a short description of all subsets is given in Table 3. In the latter table we also reference for each test set the publication where it was introduced first. The excellent papers of Grimme and coworkers^[^ ^57^ ^]^ and Head‐Gordon and coworkers^[^ ^168^ ^]^ relief us from the burden of a detailed description of all subsets and we refer to them for details. Nevertheless, we will briefly introduce all six subcategories employed herein.

**TABLE 2:** List of all 6 subcategories comprising our database. The subsets of large molecules are analyzed separately

Category	Description	#_subsets_	#	$\bar{\left|\Delta E\right|}$
TC	Basic properties and reaction energies	21	572	132.34
BH	Reaction barrier heights	9	264	21.92
IE	Simple isomerization reactions	10	274	4.52
ID	Difficult isomerization reactions	6	159	25.96
INC	Intermolecular noncovalent interactions	12	375	11.84
	Total database	58	1,644	55.58
LM	Large molecules	5	85	23.60

**TABLE 3:** List of all subsets that comprise the database employed in this work together with a short description of the types of systems they contain. The third column displays the average reaction energy in a subset and the fourth column its number of datapoints

Name	Description	$\bar{\left|\Delta E\right|}$	#
Basic properties and reaction energies			
FH51^[^ 57, 215, 216 ^]^	Diverse reaction energies in inorganic systems	31.01	51
YBDE18^[^ 57, 217 ^]^	Bond dissociation energies in Ylides	49.28	18
AL2X6^[^ ^57^ ^]^	Dimerization energies for Al_2_X_3_‐compounds	35.88	6
DARC^[^ 169, 218 ^]^	Diels‐Alder reactions	32.47	14
NBPRC^[^ 57, 169, 219 ^]^	H_2_ activation reactions by frustrated Lewis pairs	27.71	12
HEAVYSB9^[^ ^220^ ^]^	Dissociation energies in group 14–16 hydrides	58.02	9
BSR36^[^ 57, 221, 222 ^]^	Bond‐separation reactions of saturated hydrocarbons	16.20	36
RSE43^[^ 57, 223 ^]^	Radical‐stabilization energies	7.60	43
W4‐11^[^ 57, 224 ^]^	Atomization energies	306.91	140
G21EA^[^ 57, 169, 225 ^]^	Electron affinities	33.62	25
G21IP^[^ 57, 169, 225 ^]^	Ionization potentials	257.61	36
DIPCS10^[^ ^57^ ^]^	Double ionization potentials of closed‐shell systems	654.26	10
PA26^[^ ^57^ ^]^	Adiabatic proton affinities	189.05	26
SIE4x4^[^ ^57^ ^]^	Dissociation curves of very small molecules/self‐interaction error related problems	33.72	12
ALKBDE10^[^ ^57^ ^]^	Dissociation energies of group 1–2 diatomic molecules	100.69	10
RC21^[^ ^57^ ^]^	Fragmentation and rearrangement reactions in radical cations	35.70	21
ALK8^[^ ^57^ ^]^	Reactions energies for alkaline compounds	62.60	8
DC13^[^ ^57^ ^]^	Compilation of difficult reactions for DFT methods	54.98	13
G2RC^[^ ^57^ ^]^	Selected reaction energies of selected G2/97 systems	51.26	25
BH76RC^[^ ^169^ ^]^	Reaction energies of the BH76 test set	21.39	30
MOR23^[^ 226, 227 ^]^	Selected metalorganic reactions between single‐reference species containing various Tis	35.57	23
Reaction barrier heights			
WCPT18^[^ 169, 228 ^]^	Proton‐transfer barrier heights	34.99	18
BHROT27^[^ ^57^ ^]^	Barrier heights for rotation around single bonds	6.37	27
BHPERI^[^ 57, 169, 229, 230, 231 ^]^	Barrier heights of pericyclic reactions	20.87	26
BHDIV10^[^ ^57^ ^]^	Diverse reaction barrier heights	45.33	10
INV24^[^ 57, 232 ^]^	Inversion/racemisation barrier heights	32.85	24
CR20^[^ 168, 233 ^]^	Cyclo‐reversion reaction energies	19.31	20
CRBH20^[^ 168, 234 ^]^	Barrier heights for cyclo‐reversion of heterocyclic rings	46.13	20
TMBH43^[^ 226, 235, 236, 237, 238 ^]^	Barrier heights for reactions catalyzed by 4d‐ and 5d‐TMs	11.08	43
BH76^[^ 57, 169, 239, 240 ^]^	Diverse barrier heights for reactions involving small molecules	18.61	76
Simple isomerization reactions			
ISO34^[^ 57, 241 ^]^	Isomerisation energies of small and medium organic molecules	14.57	34
ICONF^[^ ^57^ ^]^	Isomers of inorganic systems	3.27	17
ACONF^[^ 57, 242 ^]^	Relative energies of alkane conformers	1.83	15
TAUT15^[^ ^57^ ^]^	Relative energies in tautomers	3.05	15
Amino20x4^[^ 57, 243 ^]^	Relative energies in amino acid conformers	2.44	80
PCONF^[^ 57, 244, 245 ^]^	Relative energies in tri‐ and tetrapeptide conformers	1.62	10
MCONF^[^ 57, 246 ^]^	Relative energies in melatonin conformers	4.97	20
SCONF^[^ 57, 169, 247 ^]^	Relative energies of sugar conformers	4.60	10
PArel^[^ ^57^ ^]^	Relative energies in protonated isomers	4.63	20
BUT14DIOL^[^ 57, 248 ^]^	Relative energies in butane‐1,4‐diol conformers	2.80	32
Difficult isomerization reactions			
EIE22^[^ 168, 249 ^]^	Isomerisation energies of enecarbonyls	5.44	22
Styrene45^[^ 168, 250 ^]^	Isomerisation energies of C_8_H_8_	62.64	45
ISOMERIZATION20^[^ ^168^ ^]^	Compilation of difficult isomerisation energies	31.84	20
DIE60^[^ 168, 202 ^]^	Double‐bond isomerisation energies in dienes	4.71	60
IDISP^[^ 51, 57, 162, 169, 241, 251 ^]^	Intramolecular dispersion interactions for medium molecules	14.22	6
C20C24^[^ 168, 252 ^]^	Isomerisation energies of C_20_ and C_24_	30.77	6
Intermolecular noncovalent interactions			
S66^[^ 57, 253 ^]^	NCI energies in organic	5.47	66
	Molecules and biomolecules		
S10x8^[^ ^253^ ^]^	10 dimers from S66 in 8 nonequilibrium geometries	5.47	80
X40^[^ ^254^ ^]^	Binding energies of NCIs involving	3.76	40
	Halogenated molecules		
HEAVY28^[^ 57, 255 ^]^	NCI energies for heavy element hydrides	1.24	28
CHB6^[^ 57, 256 ^]^	Interaction energies in cation–neutral dimers	26.79	6
AHB21^[^ 57, 256 ^]^	Interaction energies in anion–neutral dimers	22.49	21
IL16^[^ 57, 256 ^]^	Interaction energies in anion–cation dimers	109.04	16
PNICO23^[^ 57, 257 ^]^	Interaction energies in pnicogen‐containing dimers	4.27	23
CT20^[^ 168, 258 ^]^	Binding energies of charge‐transfer complexes	0.98	20
CARBHB12^[^ ^57^ ^]^	Hydrogen‐bonded complexes between carbene analogues and H_2_O, NH_3_, or HCl	6.04	12
ADIM6^[^ 57, 255 ^]^	Interaction energies of n‐alkane dimers	3.36	6
3B‐69‐TRIM^[^ 168, 259 ^]^	Binding energies of trimers, with three different orientations of 23 distinct molecular crystals	12.30	69
Isomerization reactions, NCIs and enzymatic reactions for large systems			
ISOL24^[^ 57, 260 ^]^	Isomerisation energies of large organic molecules	21.92	24
C60ISO^[^ 57, 261 ^]^	Relative energies between C_60_ isomers	98.25	9
L7^[^ ^262^ ^]^	Dimerization energies in large organic systems	15.32	7
UPU23^[^ 57, 263 ^]^	Relative energies between RNA‐backbone conformers	5.72	22
ENZYMES23^[^ ^264^ ^]^	Reaction energies and barrier heights in enzymatic reactions	18.20	23

While there is considerable overlap between MGCDB84 and GMTKN55, the reference data in the latter one is generally newer and often more accurate.^[^ ^226^ ^]^ In example, MGCDB84 contains much data from GMTKN30,^[^ ^162^ ^]^ which has been updated in GMTKN55.^[^ 57, 226 ^]^ Consequently, for test sets contained in both databases, we always compared our results to the reference values presented in the latter one and in total we selected 45 out of the 55 subsets in GMTKN55 (two of them, (MCONF, BUT14DIOL) with reduced size^[^ ^265^ ^]^) to benchmark all DFAs in Table 1 and included three more into our subcategory of large molecules.

#### 2.1.1 Transition metal containing systems

As they are not part of GMTKN55 or MGCDB84, we describe the TM containing subsets in our database in some detail. We employ two subsets with 66 reaction energies combined, capturing different aspects of TM chemistry.

The MOR23 test set is a subset of the MOR41 database recently presented by Grimme and coworkers,^[^ ^227^ ^]^ consisting of 41 reactions involving large molecules with up to 120 atoms and including 13 different TMs. All reference values have been obtained on the DLPNO‐CCSD(T)^[^ 23, 31 ^]^/CBS level of theory and all structures are chosen to have single‐reference character. To keep the overall computational effort manageable, we excluded some of the largest systems from our benchmarks and herein only present all 23 remaining reaction energies with an average reference value of 35.57 kcal/mol.

The TMBH43 test set is a compilation of several smaller test sets^[^ 235, 236, 237, 238 ^]^ and contains 43 barrier heights of reactions catalyzed by TMs, 6 are catalyzed by the 4d‐elements Zr^[^ ^236^ ^]^ or Mo,^[^ ^238^ ^]^ and 37 are catalyzed by one of the 5d‐elements Pt,^[^ ^235^ ^]^ Au,^[^ ^235^ ^]^ Ir,^[^ ^235^ ^]^ Re^[^ ^237^ ^]^ or W,^[^ ^238^ ^]^ out of these 22 by Au. All reference values have been obtained at the CCSD(T)/CBS level of theory. The average barrier height in TMBH43 is 11.08 kcal/mol.

#### 2.1.2 Basic properties and reaction energies

This subcategory (TC for thermochemistry) contains 21 subsets with a total of 572 datapoints and covers many aspects of thermochemistry. With the exception of the MOR23 test set, all test sets in this category are taken from GMTKN55. While it mainly features basic properties like atomization energies (W4‐11^[^ ^224^ ^]^), bond dissociation energies (BSR36,^[^ 57, 221, 222 ^]^ SIE4x4^[^ ^57^ ^]^), electron affinities (G21EA^[^ ^225^ ^]^), ionization potentials (G21IP,^[^ ^225^ ^]^ DIPCS10^[^ ^57^ ^]^) or proton affinities (PA26^[^ ^57^ ^]^) for small model systems, TC also contains subsets covering bonded interactions of various types like Diels‐Alder reactions (DARC^[^ 169, 218 ^]^) or H_2_‐activation by frustrated Lewis‐pairs (NBPRC^[^ 57, 169, 219 ^]^), both being of great practical interest,^[^ 219, 266, 267 ^]^ as well as dissociation reactions (HEAVYSB9,^[^ ^57^ ^]^ YBDE18,^[^ ^217^ ^]^ ALKBDE10^[^ ^57^ ^]^). We especially emphasize the importance of the challenging SIE4x4 subset which is a compilation of self‐interaction error (SIE)‐related problems,^[^ ^57^ ^]^ and DC13^[^ ^57^ ^]^ (which also contains isomerization reactions), being a selection of difficult cases for many DFAs. The average reaction energy for this category is 132.34 kcal/mol.

#### 2.1.3 Reaction barrier heights

The BH (barrier heights) category consists of nine subsets, six being taken from GMTKN55, two from MGCDB84 and one of them involving TM species, with a total of 264 datapoints and average barrier height of 21.92 kcal/mol. It contains the BH76^[^ 169, 239, 240 ^]^ and BHDIV10^[^ ^57^ ^]^ datasets featuring all kind of reactions involving small molecules as well as the CRBH20,^[^ ^234^ ^]^ CR20,^[^ ^233^ ^]^ and BHPERI^[^ 57, 169, 229, 230, 231 ^]^ test sets with reference values for cycloreversions and pericyclic reactions. It also contains rotational barriers around single bonds which are usually very low. To the best of our knowledge, the TM containing test set TMBH43 is herein included for the first time in a comprehensive benchmark.

#### 2.1.4 Easy isomerization energies

This category is comprised of 10 subsets with a total of 274 relative energies of mainly organic molecules like alkane chains (ACONF^[^ ^242^ ^]^), sugars (SCONF^[^ 57, 169, 247 ^]^), peptides (PCONF^[^ 57, 244, 245 ^]^) and various kinds of other species (BUT14DIOL,^[^ ^248^ ^]^ MCONF,^[^ ^246^ ^]^ ISO34,^[^ ^241^ ^]^ TAUT15,^[^ ^57^ ^]^ PArel^[^ ^57^ ^]^). The biggest subset in this category is Amino20x4,^[^ 57, 243 ^]^ containing the four relative energies between the five energetically lowest conformations of the 20 proteogenic amino acids. ICONF^[^ ^57^ ^]^ is the only subset with isomers of inorganic systems. The average relative energy of this category is with 4.52 kcal/mol very low. The electronic structures of these species are rather simple (isomerization easy [IE]) and are usually very well described already on the GGA + Dispersion level of theory. However, given the small energy differences in this subcategory, high accuracy is required to correctly reproduce the energetic ordering of conformers.

#### 2.1.5 Difficult isomerization reactions

Inspired by Head‐Gordon and coworkers,^[^ ^168^ ^]^ we decided to introduce a second category of isomerization reactions comprising of six test sets with 274 relative energies. The electronic structures of the systems in this category are notoriously difficult (isomerization difficult [ID]) to describe for GGAs,^[^ ^168^ ^]^ suffering from many‐electron self‐interaction error.^[^ ^268^ ^]^ Among others, ID features strained ring systems (Styrene45,^[^ ^250^ ^]^ C20C24^[^ ^252^ ^]^), Dienes (DIE60^[^ ^202^ ^]^) and isomerization reactions of enecarbonyls (EIE22^[^ ^249^ ^]^). With 25.96 kcal/mol, the average of the relative energies in this category is considerably higher than for IE.

#### 2.1.6 Intermolecular noncovalent interactions

The fifth category of our database contains the energies of 274 noncovalently bounded complexes, relative to the monomers of which they consist. This category contains 12 subsets and features the prominent S66^[^ ^253^ ^]^ databases of dimerization reactions between organic molecules and biomolecules and the X40^[^ ^254^ ^]^ test set of interaction energies between halogene‐containing organic molecules, both complied by Hobza and coworkers. While these systems are in their equilibrium geometry, Hobza and coworkers S66x8^[^ ^253^ ^]^ test set additionally contains all dimers in the S66 test set at eight different nonequilibrium geometries and is also part of MGCDB84. While it is certainly insightful to benchmark nonequilibrium geometries also, we only selected 10 out the 66 potential energy curves in order to keep our dataset well balanced, resulting in the S10x8 subset.

We note, that the very popular S22^[^ ^269^ ^]^ subset (and its extension to nonequilibrium geometries in the S22x5 database), which is also a part of GMTKN55 is not part of our database. Despite its usefulness, it is rather imbalanced and does not represent important interactions like single hydrogen bonds or aliphatic‐aliphatic dispersion interactions.^[^ ^253^ ^]^ These shortcomings have been addressed with the compilation of S66 and we argue that with its introduction S22 is obsolete.

Other important subsets are CHB6,^[^ ^256^ ^]^ AHB21,^[^ ^256^ ^]^ and IL16^[^ ^256^ ^]^ containing dimerization energies for charged systems and Hobza and coworkers 3B‐69‐TRIM,^[^ ^259^ ^]^ being part of MGCDB84, containing 23 trimers of organic molecules in three different orientations each. The average energy of the reactions contained in this category is 11.84 kcal/mol.

#### 2.1.7 Large systems

The last category differs from all other categories in the sense that its subsets cannot be assigned to a certain type of chemical interactions. We refer to the systems in this category as *large* in the sense that it contains mainly molecules for which a canonical DH‐DFA calculation requires a considerable amount of CPU‐time. To give an example, calculation of the 10 isomers in the C60ISO^[^ ^261^ ^]^ test set on the B2GP‐PLYP/TZ2P level of theory already requires 58 hr on a single node with 24 cores in our implementation.

The category contains five subsets with 85 dimerization energies, relative conformational energies, reaction energies and barrier heights with an average energy of 23.60 kcal/mol and reference values are usually obtained on the DLPNO‐CCSD(T)/CBS level of theory. Benchmarking large systems is crucial to assess the robustness and accuracy of local approximations which do not come into play for small systems. Especially PARI‐errors errors have already been reported to accumulate with increasing system size.^[^ ^100^ ^]^ Furthermore, growing concern has been expressed that MP2 might diverge for weak interactions in large molecular complexes.^[^ 270, 271 ^]^

ISOL24,^[^ ^260^ ^]^ C60ISO, and UPU23^[^ ^263^ ^]^ contain isomerization reactions and are already part of GMTKN55. Additionally, we selected Hobza and coworkers popular L7^[^ ^262^ ^]^ test set of noncovalently bounded dimers with up to 120 atoms as well as another benchmark set which we designate herein ENZYMES23. It is based on recent work of Goerigk an coworkers^[^ ^264^ ^]^ who published reference values for different model systems of five enzymatically catalyzed reactions, resulting in a dataset containing 28 datapoints. For this work, we considered the four enzymatic reactions with 23 data points in total for which the reference values have been obtained at the DLPNO‐CCSD(T)/CBS level of theory.

### 2.2 Selection of density functional approximations

In total, we selected 60 DFAs, among these 36 DHs, 18 hybrids and 6 (meta‐)GGAs. We focus on DHs in this study as many of them have only been published recently and have not been comprehensively benchmarked so far. The performance of Hybrid‐functionals and (meta‐)GGAs, however, has been assessed excessively in the last decades and due to their rather fast convergence to the CBS limit, we do not expect significant deterioration of performance compared to results obtained with QZ basis sets. DHs, on the other hand, suffer significantly more from BSE and are more challenging for the PARI‐approximation. Thus, we herein restrict ourselves to the best hybrids and (meta‐)GGAs known to the literature and the results we present for them in this paper can be seen as a frame of reference to put the performance of the DHs into prespective.

The XC energy $E_{\mathrm{xc}}^{\mathrm{DFA}}$ for all DFAs benchmarked in the current paper can be expressed as

(2) $$E_{\mathrm{xc}}^{\mathrm{DFA}}=\left(1-a_{x}\right)E_{x}^{\mathrm{GGA}}+a_{x}E_{x}^{\mathrm{HF}}+a_{c,\mathrm{gga}}E_{c}^{\mathrm{GGA}}+a_{c,\mathrm{os}}E_{c,\mathrm{os}}^{\mathrm{MP}2}+a_{c,\mathrm{ss}}E_{c,\mathrm{ss}}^{\mathrm{MP}2}+E_{\text{empirical}}^{\text{DISP}},$$

were $E_{x}^{\mathrm{GGA}}$ ($E_{c}^{\mathrm{GGA}}$) denote the exchange (correlation) part of some (meta‐)GGA, $E_{x}^{\mathrm{HF}}$ the exact‐exchange like energy contribution, $E_{c,\mathrm{os}}^{\mathrm{MP}2}$ ($E_{c,\mathrm{ss}}^{\mathrm{MP}2}$) denotes the opposite‐spin (os) (same‐spin [ss]) contribution to the MP2 correlation energy and finally, $E_{\text{empirical}}^{\text{DISP}}$ is an empirical dispersion correction term. While many other forms of dispersion corrections have been suggested,^[^ 272, 273, 274, 275, 276 ^]^ we restrict ourselves to the popular atom‐atom dispersion potentials DFT‐D3^[^ 173, 255 ^]^ and DFT‐D4.^[^ 174, 181 ^]^

For (meta‐)GGAs (*a*_*c*, *os*_ = *a*_*c*, *ss*_ = *a*_*x*_ = 0) and hybrids (*a*_*c*, *os*_ = *a*_*c*, *ss*_ = 0) our selection is mainly based on their performance in the recent comprehensive benchmarks by Goerigk et al. on the GMTKN55 database^[^ 57, 58 ^]^ and Mardirossian et al. on the MGCDB84 database.^[^ ^168^ ^]^ The only exceptions are the B3LYP and PBE0 hybrid functionals, which we included due to their continuing high popularity despite their now well established^[^ 9, 57, 162, 168, 264, 277 ^]^ average performance. As functionals based on the B95^[^ ^278^ ^]^ and B97 correlation functionals were not available to us (except for the hybrids *ω*B97‐X and B97 which were available to us through libxc^[^ ^279^ ^]^), we excluded the popular hybrids B97M‐V^[^ ^280^ ^]^ and *ω*B97X‐V^[^ ^281^ ^]^ as well as the DHs *ω*B97X‐2,^[^ ^282^ ^]^ *ω*B97M(2),^[^ ^283^ ^]^ PWPB95,^[^ ^162^ ^]^ and DSD‐PBEB95 from this study.^[^ ^214^ ^]^

The majority of the herein benchmarked DH‐DFAs are of the DSD (Double hybrid, Spin‐component scaled, Dispersion)‐type.^[^ ^212^ ^]^ These functionals are parametrized together with an empirical dispersion correction term as well as without any constraints on *a*_*c*, *ss*_, *a*_*c*, *os*_ and *a*_*c*, *gga*_. Consequently, in this approach different forms of dispersion correction give rise to different overall parametrizations. Examples are the recently published^[^ ^60^ ^]^ reparametrizations of DSD‐type functionals by the Martin group, both for the D4^[^ 174, 181 ^]^ dispersion correction as well as for the older D3^[^ ^255^ ^]^ version with Becke‐Johnson damping (D3[BJ]).^[^ ^173^ ^]^ While we consistently assess all other functionals with and without an empirical‐dispersion correction term, we benchmark DSD‐functionals in their dispersion corrected form only.^[^ ^284^ ^]^

We especially emphasize DOD‐functionals in our study, a special variant of DSD‐functionals with *a*_*c*, *ss*_ = 0. $E_{c,\mathrm{os}}^{\mathrm{MP}2}$ can be evaluated efficiently using SOS‐AO‐PARI‐MP2, and DOD‐functionals can therefore be applied to molecules consisting of hundreds of atoms in a routine fashion.^[^ ^144^ ^]^ As they can provide accuracies comparable to their DSD‐counterparts, they are in our opinion the most‐interesting DHs for practical applications.

Table 1 list the DFAs assessed herein together with the employed dispersion correction as well as the parameters *a*_*x*_ and *a*_*c*_ from (2). In total, 122 unique combinations of DFA and dispersion‐correction are evaluated in this study.

### 2.3 Computational details

The majority of our DFT calculations as well as all empirical dispersion corrections have been performed with a locally modified development version of the ADF code.^[^ 79, 146, 147 ^]^ For a variety of subsets, we also performed B3LYP and B2GP‐PLYP calculations with PSI4.^[^ ^285^ ^]^

For all test sets contained in GMTKN55, the structures and reference energies as available on the dedicated website^[^ ^286^ ^]^ have been used. For all other datasets we employed the structures and reference energies from the ACCDB^[^ ^226^ ^]^ database.

All energies for the S66, S66x10, X40, L7, AHB21, IL16, CHB6, and CT20 subsets have been computed using the counterpoise (CP) method of Boys and Bernardi^[^ ^287^ ^]^ to correct for the basis set superposition error (BSSE).

#### 2.3.1 ADF calculations

PARI‐*K* has been used for all hybrid and DH calculations and, if not stated otherwise, PARI‐MP2 for the post‐SCF energy correction required for the latter ones, except for all test sets in the category LM, for which SOS‐AO‐PARI‐MP2 has been used instead. The accuracy of the latter algorithm depends on the quality of the numerical approximation to an integral.^[^ ^288^ ^]^ We use nine quadrature points, being the default in ADF and a number for which this approximation can be considered as sufficiently converged.^[^ 132, 289 ^]^ For the evaluation of the exact exchange as well as for all SOS‐AO‐PARI‐MP2 calculations, the *Normal* tier of threshold qualities has been used. We refer to our recent work^[^ ^144^ ^]^ for a detailed explanation and discussion of these algorithms as well as explicit threshold values.

The majority of the herein presented numbers have been calculated on the all‐electron level using the TZ2P (TZ with two shells of polarization functions) STO‐type basis set.^[^ ^290^ ^]^ The *Normal* auxiliary fit set has been used for for PARI‐*K* and all PARI‐MP2 calculations with the only exception of the IDISP test set for which we employed the *Very Good* auxiliary fit set for both, PARI‐*K* and PARI‐MP2 in all DH calculations. For both, the numerical integration quality as well as the quality of the DF for the evaluation of the *J*‐term,^[^ ^291^ ^]^ we used *Good* quality.^[^ 290, 291 ^]^

For all systems containing fourth‐row elements or heavier, relativistic effects have been treated with the Zero Order Regular Approximation (ZORA)^[^ 292, 293, 294, 295 ^]^ in conjunction with ZORA‐optimized basis sets and the Minimum of neutral Atomic potential Approximation (MAPA).

#### 2.3.2 PSI4 calculations

All PSI4 calculations have been performed using def2‐TZVPP^[^ 296, 297 ^]^ and employing DF for the *J*‐ and *K*‐terms as well as for the evaluation of MP2 energies; default auxiliary fits sets have been employed.^[^ ^299^ ^]^ For all subsets, calculations have been performed on the all‐electron level. Default settings have been used for the integration grids and all other numerical settings.

### 2.4 Representative timings

To give an estimate on the CPU‐time requirements for a prototypical GGA, hybrid and double‐hybrid, respectively, we herein report timings for our whole database. Calculations have been performed sequentially on a 2 × 12‐core 2.4 GHz Intel Xeon E5‐2695 v2 (ivy Bridge) with 64 GB of memory. Sequential calculation is obviously not appropriate for small molecules as parallelization will be inefficient. In practice, the best performance will be achieved by running many jobs in parallel on one core each and only parallelize individual calculations for which the memory available on a single core is not sufficient. For the present context, this is irrelevant as we only aim to compare the relative timings. Results for all calculations required to obtain values for our whole database of 1,644 datapoints without large molecules are given in Table 4. For BLYP, B3LYP and revDOD‐BLYP we also give timings for the subset of large systems.

**TABLE 4:** CPU‐times for our whole database obtained with BLYP,B3LYP,B2GP‐PLYP and revDOD‐BLYP

	BLYP	B3LYP	B2GP‐PLYP	revDOD‐BLYP
Small	33.10	57.00	89.08	
Large	18.42	26.56		36.03
S66	2.55	3.51	3.95	4.12

*Note:* All timings are given in hours and have been obtained on a single node with 24 cores. In the first row, the CPU‐times for our whole database except for the subcategory of large molecules are given while the timings for this subcategory are displayed in the second row. B2GP‐PLYP calculations have been performed using PARI‐MP2. For revDOD‐BLYP, SOS‐AO‐PARI‐MP2 has been used.

For all systems in our database except for the large ones, all BLYP calculations can be performed in 33 hr; for B3LYP, less than twice CPU‐time is required and less than thrice for B2GP‐PLYP. The most‐time consuming part here are spin‐unrestricted MP2 calculations as they are currently less‐efficient than spin‐restricted ones. For the S66 test set of organic molecules of between roughly 10–30 atoms, timings differ considerably less. With 3.5 hr, all 198 B3LYP‐calculations can be performed in only 1 hr more than required for BLYP and the calculation of the post‐SCF correlation energy only requires a negligible amount of time for both, PARI‐MP2 and SOS‐AO‐PARI‐MP2. A similar picture is obtained for large molecules. Moving from BLYP to B3LYP, CPU‐time requirements increase by roughly 50% and double moving from BLYP to revDOD‐BLYP when the AO‐based algorithm is used. Here, the evaluation of $E_{c,\mathrm{os}}^{\mathrm{MP}2}$ only consumes about 25% of the total CPU‐time. Clearly, these timings show that CPU‐time is hardly a concern when climbing Jacob's ladder to the top, provided a DOD‐functional is used.

## 3 RESULTS

In this section, the results of our calculations are presented. In the following discussion, we proceed in two steps. First, we investigate the basis set dependence of the STO/PARI‐approach in detail. To do so, we present results for B3LYP as an exemplary hybrid, and B2GP‐PLYP as an exemplary DH, respectively. Second, we analyze and discuss the results of our ADF calculations for all DFAs in Table 1 in order to identify the most accurate and robust DFAs. All numbers presented in the following figures can also be found in table form in the ESI.

### 3.1 Comparison of STO/PARI approach to Gaussian type basis sets

As a first step in the analysis of our results, we compare our TZ2P calculations to calculations using the GTO‐type basis set def2‐TZVPP for a selection of 30 subsets from our database. def2‐TZVPP is comparable in size to the TZ2P basis set but has more shells of polarization functions for the first three rows of the periodic table. Table 5 shows the sizes of the herein employed basis sets for selected elements.

**TABLE 5:** Composition and basis set sizes (in parentheses) for selected elements of the STO‐type TZ2P and the GTO‐type def2‐TZVPP basis sets^[^ ^298^ ^]^

Element	TZ2P	def2‐TZVPP
H	3s,1p,1d (11)	3s,2p,1d (14)
C	5s,3p,1d,1f (26)	5s,3p,2d,1f (31)
Ge	9s,7p,4d,1f (57)	6s,5p,4d,1f (48)

For our analysis we use two exemplary DFAs, the hybrid B3LYP and the DH B2GP‐PLYP. The results of the respective functional as calculated by Grimme and coworkers using an augmented def2‐QZVP basis set (aug‐def2‐QZVP in the following, for details see Reference [57]) close to the CBS limit and available online^[^ ^286^ ^]^ serve as reference values. This allows to investigate the basis set errors (BSE) without being flawed by cancellation between functional error and BSE.

While for hybrid‐functionals basis sets of TZ quality are usually assumed to give results close to the CBS limit, a larger basis set dependence can be expected for B2‐GPPLYP. Also due to possible difficulties in the description of virtual orbitals, the PARI approximation might introduce larger errors than for B3LYP. To this end, larger BSEs as well as larger differences between TZ2P and def2‐TZVPP are to be expected.

**FIGURE 1:**
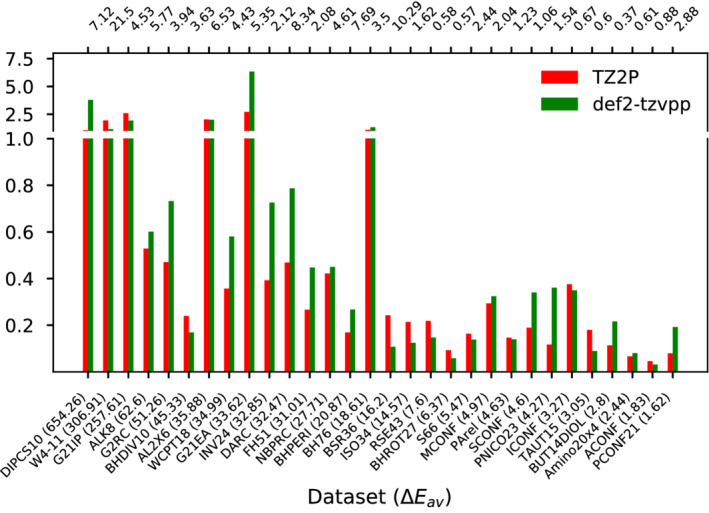
Comparison of the BSE with respect to aug‐def2‐QZVP for B3LYP. For each of the selected subsets the sign‐corrected average BSE is shown. The subsets are sorted according to their average reaction energy ($|\bar{\Delta E}|$, in parentheses after each subsets name) in descending order. For each subset, the MAD obtained with aug‐def2‐QZVP with respect to the higher level reference is given on the secondary x‐axis on top. All values are in kcal/mol [Color figure can be viewed at wileyonlinelibrary.com]

Figure 1 shows the MADs obtained with B3LYP/TZ2P and B3LYP/def2‐TZVPP with respect to B3LYP/aug‐def2‐QZVP for all 30 subsets considered herein. The subsets are sorted according to their average reaction energy $|\bar{\Delta E}|$ in descending order and $|\bar{\Delta E}|$ is given in parentheses after each subsets name. For comparison, the secondary x‐axis displays the MAD of B3LYP/aug‐def2‐QZVP with respect to the usual reference values from high‐level WF methods.

It can be verified immediately, that the average BSEs for both basis sets are of the same order of magnitude. One also identifies a rough correlation between $|\bar{\Delta E}|$ and the error for both basis sets. On the left hand side of the plot, BSEs often exceed 1 kcal/mol, being most pronounced for ionization potentials (DIPCS10, G21IP) and electron affinities (G21EA) of atoms and small molecules. Possibly due to the more accurate description of the wave function close to the nuclei, the STO basis set outperforms the GTO one considerably for these subsets. The right hand side of the plot (BSR36 and subsets further to the right) features subsets with smaller $|\bar{\Delta E}|$, mostly containing isomerization energies and NCIs, but also smaller BSEs well below 0.4 kcal/mol.

Except for ionization potentials and electron affinities, TZ2P and def2‐TZVP show comparable performance and no basis set can clearly be identified as superior to the other. There are subsets, where the def2‐TZVPP BSE is roughly twice as large as the one from TZ2P (INV24, DARC, PNICO23) or the other way round (BSR36, ISO34, RSE43). This is also reflected in the average BSE for the entirety of all 30 subsets, which is 0.68 kcal/mol for TZ2P and 0.72 kcal/mol for the slightly larger def2‐TZVPP.

For all subsets, the BSE is at least one order of magnitude smaller than $|\bar{\Delta E}|$ which is of course a necessary prerequisite for qualitatively correct reaction energies. On the other hand, the BSE is sometimes even larger than the error of the functional at the basis set limit. In example, def2‐TZVPP gives a BSE of 6.34 kcal/mol for G21EA while B3LYP/aug‐def2‐QZVP gives a MAD of 5.35 kcal/mol. Clearly, this is a rather special case of arguably little practical relevance. However, especially for isomerization energies, where method errors are sometimes of the same order of magnitude than energetic differences between isomers, already small BSEs can lead to skewed results. For ICONF, both basis sets give an error larger than 0.4 kcal/mol while B3LYP/aug‐def2‐QZVP only gives a slightly larger MAD of 0.67 kcal/mol. These examples seriously question the common belief that basis set of TZ quality are sufficient for hybrid calculations.

**FIGURE 2:**
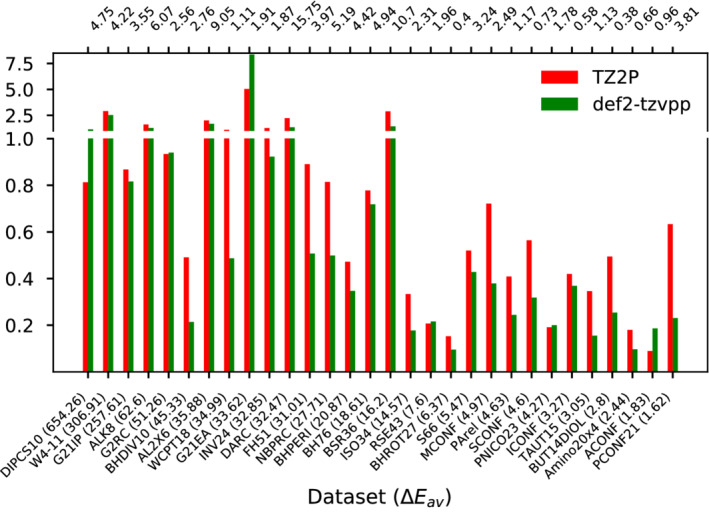
Comparison of the BSE with respect to aug‐def2‐QZVP for B2‐GPPLYP. For each of the selected subsets the sign‐corrected average BSE is shown. The subsets are sorted according to their average reaction energy ($|\bar{\Delta E}|$, in parentheses after each subsets name) in descending order. For each subset, the MAD obtained with aug‐def2‐QZVP with respect to the higher level reference is given on the secondary x‐axis on top. All values are in kcal/mol [Color figure can be viewed at wileyonlinelibrary.com]

In the same way as Figure 1, Figure 2 shows the BSEs of TZ2P and def2‐TZVPP for B2GP‐PLYP for all subsets. While essentially the same trends can be observed than for B3LYP, for the subsets featuring smaller $|\bar{\Delta E}|$, def2‐TZVPP often outperforms TZ2P. While this could hint on the importance of a large set of polarization functions for the calculation of orbital‐dependent correlation energies which is known to be considerably larger for DHs than for hybrids, it could also be due to a larger PARI‐error which is more pronounced when virtual orbitals are involved. Both hypotheses cannot be verified. Increasing the basis set leads to unreliable and therefore flawed PARI‐MP2 energies for the subsets containing medium‐sized or larger molecules while it is technically impossible for us to perform STO‐calculations without PARI.

While it would of course be useful to be able to identify the exact source of error, it is of little relevance in practice. As already outlined, the use of STO‐type basis sets and PARI go hand in hand and of practical relevance is only the combined error of the approach. With 0.96 kcal/mol for def2‐TZVPP and 1.12 kcal/mol for TZ2P, respectively, the errors for both basis sets are of the same order of magnitude, although TZ2P produces larger errors than def2‐TZVPP. These numbers suggest that the STO/PARI approach can clearly compete with standard GTO‐type calculations also for DHs and the slightly decreased accuracy might be seen as a small price which is to be paid for the increased efficiency attainable with PARI‐*K* and PARI‐MP2.

Given the large number of reactions considered herein, we can state with high confidence that the data presented in this section clearly demonstrate that the STO/PARI approach can easily compete with GTO‐type basis sets in terms of accuracy for a variety of chemical problems, for both, hybrid and DH calculations.

Of course, for both types of DFAs, the possibility that the good agreement of the STO/PARI calculations with the ones using GTOs are the result of fortuitous error cancellation cannot be excluded.Whether this is of practical relevance might be more of a philosophical question. While being unpleasant from a purists point of view, the success of approximate KS‐DFT is to some extent rooted in error cancellation. In a field which is mostly based on heuristics, a method can be regarded as reliable if it only works well in virtually all cases in which it is applied. In this sense, the STO/PARI‐approach clearly works. In our introduction we asked whether it possible to perform accurate, robust and efficient hybrid‐ and DH‐KS calculations using STOs. We think that an affirmative answer to this question can already be given.

On the other hand, our data also show that the results obtained with the STO/PARI approach for individual subsets can differ considerably from GTO calculations. Depending on the details of their construction, different DFAs of the same rung will give quite different results when paired with basis sets not sufficiently close to the CBS limit. Furthermore, it is not clear if method error and BSE add up or cancel each other. Consequently, the trends observed here cannot be expected to be the same when changing the DFA for the same rung. In this sense, the results of this subsection strongly suggest that a comprehensive benchmark of DFAs using STO‐type basis sets is of high interest. This benchmark is the subject of the next subsection.

### 3.2 Discussion of the whole database and its categories

As we have performed well over 150.000 single point calculations in total, we can herein only highlight the most important trends and conclusions while we refer to Data S1 for details. We will discuss the performance of the assessed DFAs for each of the five subcategories. Results for large molecules will also be discussed whenever appropriate. Arguably, the information given in the following is most relevant for users of the ADF code. However, having established that STO/PARI calculations offer accuracies comparable to ones attainable using GTO‐type TZ basis sets, we hope the herein presented results can also serve as an important guideline for users of GTO‐based quantum chemistry codes who are not always willing or able to afford QZ sized basis sets. Final recommendations will be given based on WTMAD‐2 values as well as on a best‐worse analysis, that is, we recommend functionals based on their accuracy but also on their robustness. Before we dive into the discussion of the benchmarked DFAs performance, both metrics will shortly be introduced.

### 3.3 Statistical criteria

#### 3.3.1 Weighted mean absolute deviations

We follow the approach of Grimme and coworkers^[^ ^57^ ^]^ and base the discussion of our data on weighted mean absolute deviations (WTMAD‐2),

(3) $$\text{WTMAD}‐2=\frac{1}{N}\sum_{i}N_{i}\times {\mathrm{MAD}}_{i}\times \frac{|\bar{\Delta E}|}{{\left|\bar{\Delta E}\right|}_{i}}\;,$$

where the sum runs over all subsets in a subcategory, MAD_*i*_ denotes the mean absolute deviation of subset *i*, ${\left|\bar{\Delta E}\right|}_{i}$ its average reaction energy and *N*_*i*_ the number of datapoints in it. The benefits of this scheme have been adressed in detail by Grimme and coworkers.^[^ ^57^ ^]^ We prefer this metric over simple MADs as it does not focus on absolute accuracies but on relative ones, being clearly of higher relevance to predict and reproduce chemical trends.

#### 3.3.2 Best‐Worse‐Analysis

For this we also adopt the approach of Grimme and coworkers^[^ ^57^ ^]^ and present best‐worse analyses for the total database and subcategories. Instead of simply counting how often a functional yields the lowest (best) and the highest (worse) MAD, we rather count how often a certain functional yields a MAD which is not more than 15% higher (lower) than the MAD of the best (worse)‐performing functional.

We consider an illustrative example to point out the advantage of this approach. The functional with the lowest MAD for the Amino20x4 subset is revDSD‐PBEP86‐D4 with 0.13 kcal/mol. For six other functionals we obtain MADs not more than 15% worse. All of these functionals perform well for this subset which means that there a seven reliable (within our arbitrary criterion) DHs to describe it. As another example, we consider the DARC test set. The best DH here is B2GP‐PLYP‐D4 with a MAD of 0.23 kcal/mol, followed by mPW2K‐PLYP with a MAD of 0.57 kcal/mol. Thus, B2GP‐PLYP‐D4 is the only logical choice for this test set.

### 3.4 Discussion of all subcategories

#### 3.4.1 Basic properties and reaction energies

**FIGURE 3:**
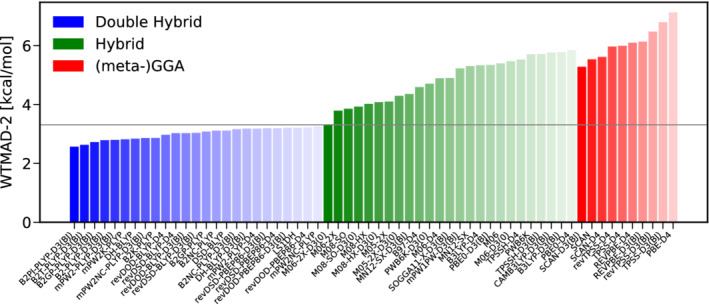
The best 25 DHs (blue) and hybrids (green) as well as the best 10 (meta‐)GGAs (red) according to their WTMAD‐2 s for the category of basic properties and reaction energies [Color figure can be viewed at wileyonlinelibrary.com]

To start with, we discuss the category of basic properties of small systems and bonded interactions. Figure 3 shows the WTAMD‐2s obtained with 60 pairings of a DFA with a variant of empirical dispersion correction; the best 25 DHs and hybrids as well as the best 10 (meta‐)GGAs. Due to its large average reaction energy of 132.34 kcal/mol, all WTMAD‐2s are relatively small, ranging from about 2.5 kcal/mol for the best DH B2T‐PLYP‐D3(BJ) to nearly 7.0 kcal/mol for PBE‐D4 being the worst GGA displayed here. Overall, Figure 3 clearly reveals that double‐hybrids are the method of choice for this category. The Minnesota functional M06‐2X‐D3(0) clearly outperforms all other hybrids by nearly 0.5 kcal/mol but the obtained WTMAD‐2 is comparable to dispersion uncorrected mPW2NC‐PLYP being ranked 25th and having been parametrized for NCIs. To our surprise, B2*π*‐PLYP‐D3(BJ), parametrized for *π*‐*π* interactions, is the best DH in this category. As it has been optimized for thermochemistry,^[^ ^55^ ^]^ it is not surprising that B2T‐PLYP‐D3(BJ) performs well too.

Taking a closer look at the performance of the benchmarked hybrid‐functionals reveals that the Minnesota functionals M06‐2X, M08‐SO and M08‐HX with different flavors of dispersion correction are the methods of choice on the hybrid level. The amount of exact exchange contained in these functionals is with more than 50% rather large. This is in line with results for GMTKN55 where the two best performing functionals are M06‐2X‐D3(0) and M08‐HX‐D3(0) and in contrast to the common belief that, due to the ability of the SIE to mimic static electron correlation,^[^ 299, 300 ^]^ a smaller fraction of exact exchange should be ideal for thermochemistry.^[^ 301, 302 ^]^

After this general analysis we proceed by taking a closer look at selected individual test sets. SIE4x4 and DC13 are interesting benchmark sets as they are composed of systems which are challenging for DFT methods. Possibly due to their large amount of exact exchange, DHs outperform all hybrids and (meta‐)GGAs by far. The only hybrid with a MAD below 6 kcal/mol is M05‐2X, while LS1‐TPSS is the best DH with a MAD below 1 kcal/mol. The best DH at the aug‐def‐QZVP level is PBE0‐2 with 1.81 kcal/mol for which we obtain a smaller MAD of 1.49 kcal/mol. Also for DC13, DHs clearly outperform all other functionals; revDOD‐PBE‐D4 performs best with 2.50 kcal/mol.

An other interesting subsets is MOR23 containing TMs. SOS1‐PBE‐QIDH performs best with a MAD of 1.8 kcal/mol (followed by PBE‐DH and the hybrid mPW1PW‐D3(BJ)) and a maximum error of 6.16 kcal/mol. Given the average reaction energy of 35.57 kcal/mol for this test set, this performance is quite satisfactory.

**FIGURE 4:**
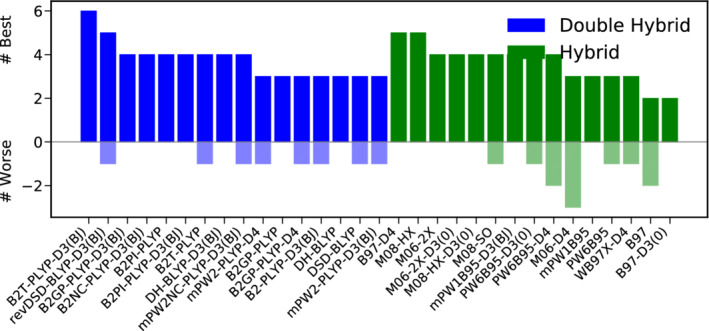
Best (full colored bars, positive x‐range)–worse (shaded bars, negative x‐range) analysis for the best 15 DHs (blue) and hybrids (green) for thermochemistry [Color figure can be viewed at wileyonlinelibrary.com]

Figure 4 presents the results of a best‐worse analysis. The good performance of B2T‐PLYP‐D3(BJ) is substantiated further, being six times among the best DH‐DFAs and never among the worst. Also revDSD‐BLYP‐D3(BJ) reaches MADs not worse more than 15% than the best DH for five times and can be recommended as a reliable functional for thermochemistry. On the hybrid level, B97‐D4 and M08‐HX are five times among the best functionals. Again, this does not come as a surprise for the latter one, given its fourth position in the WTMAD‐2 ranking, while the former only ranks tenth there. The hybrid yielding by far the lowest WTMAD‐2, M08‐SO ranks among the best hybrids four times, but once it can also be found among the worst ones.

In conclusion, we recommend B2T‐PLYP‐D3(BJ) as the method of choice for the description of bonded interaction and basic properties and we generally advise to use DH‐DFAs whenever feasible. If CPU‐time is a concern, we recommend the best DOD‐DH, revDOD‐BLYP‐D3(BJ)/D4 as well as the Minnesota functional M06‐2X‐D3(0).

#### 3.4.2 Reaction barrier heights

**FIGURE 5:**
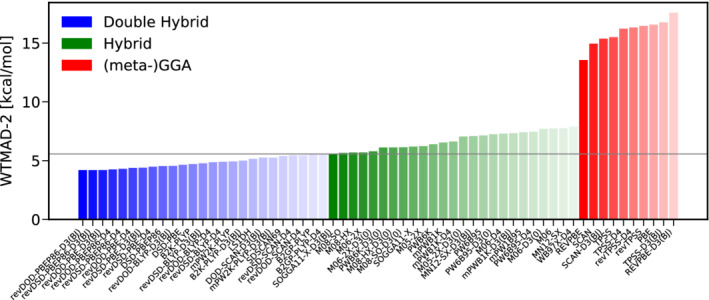
The best 25 DHs (blue) and hybrids (green) as well as the best 10 (meta‐)GGAs (red) according to their WTMAD‐2 s for the category of reaction barrier heights [Color figure can be viewed at wileyonlinelibrary.com]

The next category consists of nine subsets with 264 data points, containing barrier heights of 21.92 kcal/mol on average. The WTAMD‐2 s for the best functionals of each rung are displayed in Figure 5. As they suffer considerably from many‐body self‐interaction error, (meta‐)GGAs show a devastating performance for stretched bonds^[^ 268, 302, 303, 304, 305 ^]^ and should never be used for the calculation of barrier heights.

DHs, especially DSD‐functionals, yield clear improvements over hybrid functionals. The best 25 DHs all have a lower WTMAD‐2 than the best hybrid‐DFA. The neglect of explicit same‐spin correlation does not seem to have an adverse effect on a DHs performance illustrated by the success of the first ranked functional revDOD‐PBEP86‐D3(BJ), which even slightly outperforms its sibling revDSD‐PBEP86‐D3(BJ). Replacing the D3(BJ) dispersion correction by its successor D4 results in essentially the same picture and also the pair revDOD‐PBE‐D3(BJ)/revDSD‐PBE‐D3(BJ) shows a comparable performance.

**FIGURE 6:**
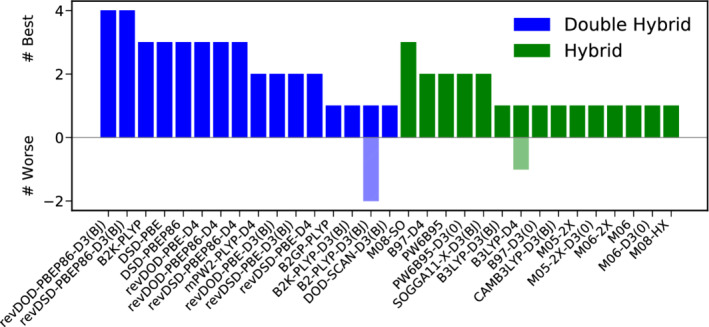
Best (full colored bars, positive x‐range)–worse (shaded bars, negative x‐range) analysis for the best 15 DHs (blue) and hybrids (green) for reaction barrier heights [Color figure can be viewed at wileyonlinelibrary.com]

As for TC, the best hybrid functionals are the dispersion uncorrected M08‐SO, M08‐HX and M06‐2X functionals outperforming their dispersion corrected counterparts slightly. The best‐worse analysis in Figure 6 substantiates the conclusions drawn from the inspection of the WTMAD‐2s. revDOD‐PBEP86‐D3(BJ) and revDSD‐PBE86‐D3(BJ) are among the best functionals in four of nine cases. On the hybrid level, M08‐SO is the leader in this category, three times being among the best hybrids. M08‐HX and M06‐2X, on the other hand only rank once among the best hybrids.

The picture drawn so far chances substantially when the TMBH43 test set, with only 15% of all datapoints in BH, is excluded from this analysis. In Data S1 we present a plot similar to Figure 5 which shows that revDOD‐PBEP86‐D3(BJ) and revDSD‐PBE86‐D3(BJ) are still the best available DHs for this category, while M08‐HX takes the lead from M08‐SO. However, without TMBH43, no DH yields clear improvements over M08‐HX. Although many DHs yield a comparatively small WTMAD‐2, none of them can clearly be recommended as the general tool for the calculation of barrier heights when no TMs are involved.

This immediately implies that DHs are the methods of choice for the calculation of barrier heights for TM involving reactions, that is, for TMBH43. Here, dispersion‐uncorrected B2K‐PLYP yields a MAD of 1.61 kcal/mol, with 1.70 and 1.71 kcal/mol, respectively, revDSD‐PBE‐D4 and revDOD‐PBE‐D4 also perform well. It should be noted, however, that none of the benchmarked methods provides a fully satisfactory description of TMBH43, given the average reaction energy of only 11.08 kcal/mol.

As it contains many barrier heights, we briefly discuss our results for ENZYMES23 here. We generally observe good performance of functionals with a large fraction of exact exchange. The best hybrid functional is M08‐HX‐D3(0). It is only slightly outperformed by revDOD‐PBEP86‐D3(BJ) with a MAD of roughly 1.2 kcal/mol. Note that Goerigk and coworkers obtained a only slightly better MAD of 1.07 kcal/mol with the herein not benchmarked SOS0‐PBE‐2‐D3(BJ) functional in their benchmark study on this test set.^[^ ^264^ ^]^

In conclusion, we cannot unreservedly recommend to use DH‐DFAs for the calculation of BHs unless TMs are involved. In this case, the method of choice is dispersion uncorrected B2K‐PLYP, having been optimized for kinetic properties, and, when CPU time is a concern, revDOD‐PBE‐D4. Otherwise, hybrid functionals seem to provide the best price/performance‐ratio for the calculation of barrier heights and we recommend M08‐HX as the most robust and accurate alternative.

#### 3.4.3 Easy isomerization reactions

The availability of a plethora of benchmark sets containing isomers of organic molecules illustrates the great relevance of this type of systems. In total, our database contains 433 relative conformational energies of which only 15 are not organic. In this work, we consider two categories of isomers, and in this paragraph we are concerned with the category containing 274 relative energies of isomers with an easy electronic structures and an average isomerization energy of only 4.52 kcal/mol.

**FIGURE 7:**
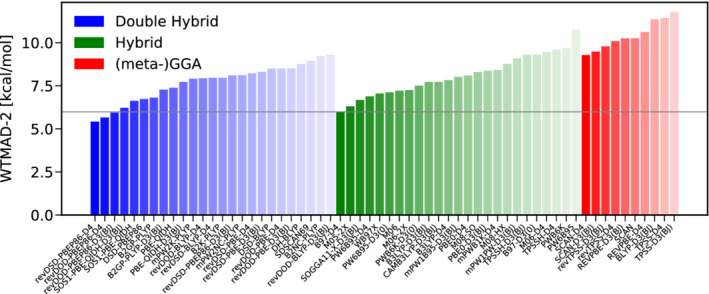
The best 25 DHs (blue) and hybrids (green) as well as the best 10 (meta‐)GGAs (red) according to their WTMAD‐2 s for the category of easy isomerization reactions [Color figure can be viewed at wileyonlinelibrary.com]

Given these subtle energy differences, obtaining qualitatively correct results requires high precision and Figure 7 reveals large differences in the accuracies of the benchmarked DFAs. While the best DH has a WTMAD‐2 of 5.4 kcal/mol, the WTMAD‐2 of the 25th is with more than 9 kcal/mol already 71% higher, for hybrids, the corresponding ratio is 79%. This might be contrasted with the corresponding number for BH, where we obtain a ratio of 25% for DHs and of 29% for hybrids, receptively. We already mention at this point that the WTMAD‐2s for IE are higher than for all other subcategories, that is, the largest relative errors are obtained here.

**FIGURE 8:**
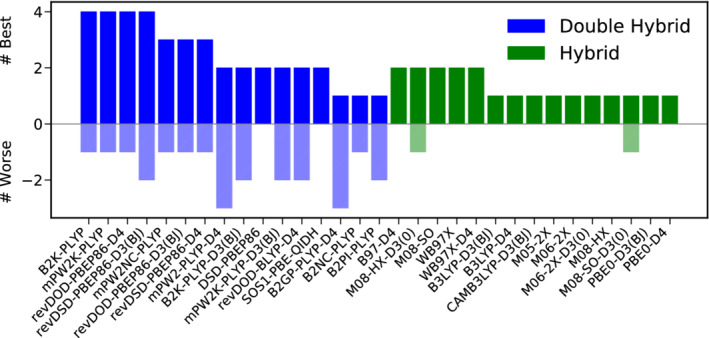
Best (full colored bars, positive x‐range)–worse (shaded bars, negative x‐range) analysis for the best 15 DHs (blue) and hybrids (green) for easy isomerization reactions [Color figure can be viewed at wileyonlinelibrary.com]

Going from the best hybrid, B97‐D4, to the best DH, revDSD‐PBEP86‐D4, the WTMAD‐2 only improves by roughly 10%, only three DHs yield improvements at all and only 16 perform better than the best meta‐GGA, SCAN‐D4. Thus, some care must be taken when selecting a DFA for these systems and with an uneducated choice of DH, one easily ends up with a worse performance than with a GGA. Hybrids, although the best of them performing slightly worse than the best DH, seem to be the most robust class of DFAs for IE. Also the best‐worse analysis in Figure 8 suggests B97‐D4 to be the most reliable DFA being never among the worst‐performing DFAs, while each of the three best DHs, revDSD‐PBEP86‐D4, revDOD‐PBEP86‐D4 and revDSD‐PBEP86‐D3(BJ), can be found among the worst performing DHs in this subcategory twice.

The source of the difficulties of DHs for this category is most likely the BSE for MP2 as exemplified by the PCONF test set of relative energies of oligopeptide conformers. Recently, Goerigk et al. investigated the basis set dependence of MP2 for parts of this subset and found a tremendous BSE for MP2 calculations on the TZ level.^[^ ^245^ ^]^ Although to a lesser extent, the same is true for SCONF.^[^ ^247^ ^]^ Indeed, eliminating the 35 data points of these two test set from our analysis, we observe that the WTMAD‐2 of the best DH is now more than 17% smaller than the one of the best hybrid. Furthermore, instead of three DHs when PCONF and SCONF are included, 11 DHs outperform the best hybrid when they are excluded (See the ESI).

On the other hand, this implies that DHs must outperform DFAs of lower rung for some test sets. For the large Amino20x4 subset, revDSD‐PBEP86‐D4 is the clear winner with 0.13 kcal/mol while B97‐D4 yields the lowest MAD of all hybrids with 0.21 kcal/mol. For the diverse ISO34 test set, the best hybrid *ω*B97X yields with 0.87 kcal/mol a MAD 91% worse than the 0.45 kcal/mol obtained for revDSD‐PBEP86‐D3(BJ). For ISO34, the unrevised version of the latter functional is also the winner in the GMTKN55 study, where Grimme and coworkers calculated a MAD of 0.41 kcal/mol.

From the subcategory of large molecules, the UPU23 and ISOL24 subsets also fall into the category of easy isomerization reactions, although with $|\bar{\Delta E}|=$ 21.92 kcal/mol the latter one features considerably larger relative energies than other subsets in IE. Our results for both subsets are in line with the numbers obtained for other subsets in this category, DHs do not perform particularly well here, although revDOD‐PBEP86‐D3(BJ)/D4 yield MADs below 1.0 kcal/mol for ISOL24, whereas the best hybrid is SOGGA11‐X‐D3(BJ) with nearly 1.5 kcal/mol. With SCAN‐D4, the best functional for UPU23 is actually a meta‐GGA.

Finally, we recommend the B97‐D4 functionals as a reliable, robust and cheap DFA for easy isomerization reactions. Also revDSD‐PBEP86‐D3(BJ)/D4 and the cheaper alternative revDOD‐PBEP86‐D3(BJ) can be recommended, although some care must be taken for test sets for which a slow convergence to the CBS limit is documented.

#### 3.4.4 Difficult isomerization reactions

We proceed with our discussion of relative conformational energies by analyzing our results for the 159 isomerization reactions in the category of difficult isomerization reactions. As poined out by Head‐Gordon and coworkers, the systems in this category, mainly consisting of strained and conjugated organic molecules with a difficult electronic structure and an average energy of 25.96, pose a big challenge for DFT methods.^[^ ^168^ ^]^ For the corresponding subcategory in MGCDB84, essentially being identical to ours, no GGA or hybrid yields a RMSD lower than 2 kcal/mol, while for their dataset of easy isomerization reactions 23 functionals yield RMSDs lower than 0.5 kcal/mol.^[^ ^168^ ^]^

**FIGURE 9:**
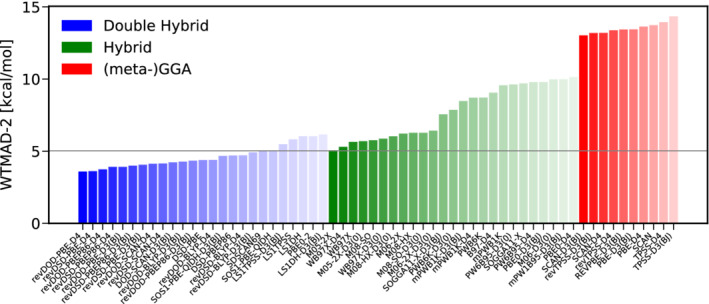
The best 25 DHs (blue) and hybrids (green) as well as the best 10 (meta‐)GGAs (red) according to their WTMAD‐2 s for the category of difficult isomerization reactions [Color figure can be viewed at wileyonlinelibrary.com]

The failure of common DFAs originates from the pronounced many‐electron SIE in the systems contained in ID^[^ ^168^ ^]^ and DHs, usually featuring large fractions of exact exchange, should provide a better description of their electronic structures. This expectation is immediately confirmed by the WTMAD‐2s presented in Figure 9. As for barrier heights, with WTMAD‐2s of up to more than 15 kcal/mol, the (meta‐)GGAs benchmarked by us show a devastating performance and are clearly inadequate for ID. The best hybrids all feature a large amount of exact exchange—M05‐2X with 56%, the range‐separated *ω*B97‐X with 100% in the long‐range‐regime, and M08‐SO with 57%. However, the WTMAD‐2 of 2.35 kcal/mol obtained with M05‐2X is still 41% higher than the 1.67 kcal/mol obtained with revDOD‐PBE‐D4. The latter functional has 68% exact exchange, a feature common to all other well‐performing DHs in this category.

Notwithstanding the great performance of DHs for this subcategory, they are generally outperformed by hybrids in the description of strained carbon clusters. It was already noted by Martin et al. more than 20 years ago^[^ ^307^ ^]^ that MP2 tends to overestimate the resonance effect in these systems and indeed, *ω*B97‐X outperforms all DHs for C20C24. Furthermore, for reasons remaining elusive to us, compared to DFAs of lower rung, DHs show a devastating performance for the C60ISO subset, confirming previous results.^[^ 57, 252 ^]^ As all C60 isomers are nonaromatic,^[^ ^308^ ^]^ the overestimation of the resonance effect is ruled out as possible cause. The best‐performing functional here is revDOD‐BLYP‐D3(BJ) with a MAD of nearly 3 kcal/mol. Given the average reaction energy of nearly 100 kcal/mol of this subset, this number if clearly satisfying. However, a MAD of less than 2 kcal/mol can be obtained using M06.

**FIGURE 10:**
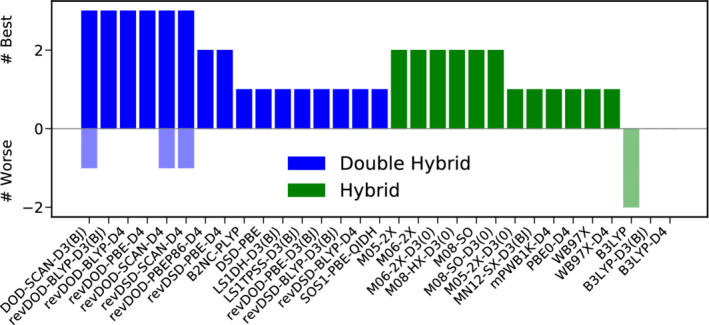
Best (full colored bars, positive x‐range)–worse (shaded bars, negative x‐range) analysis for the best 15 DHs (blue) and hybrids (green) for difficult isomerization reactions [Color figure can be viewed at wileyonlinelibrary.com]

Also DHs based on the SCAN meta‐GGA, usually not performing well in our benchmarks, are among the best DFAs for ID. On the other hand, the best‐worse analysis shown in Figure 10 reveals that DOD‐SCAN‐D3(BJ), revDSD‐SCAN‐D4 and revDOD‐SCAN‐D4 are both among the worst DHs once (for the very small IDISP subset), while they are among the best performing functionals three times. These functionals are especially suitable for the description of strained ring systems, that is, Styrene45, C20C24 and also DIE60. revDOD‐PBE‐D4, the functional with the smallest WTMAD‐2, is three times among the best functional but never among the worst. We also emphasize the excellent performance of revDOD‐BLYP‐D3(BJ) being the best functional for EIE22, DIE60 and ISOMERIZATION20 but only showing a mediocre performance for the remaining subsets.

Our final recommendation for this category is therefore clear—DOD‐functionals, especially revDOD‐PBE‐D4 and revDODPBEP86‐D4, but also revDOD‐BLYP‐D3(BJ) and DOD‐SCAN‐D3(BJ) in case of strained ring systems, should always be used.

#### 3.4.5 Intermolecular noncovalent interactions

The interactions stabilizing the structures of biomacromolecules like DNA and proteins^[^ ^269^ ^]^ or governing the self‐assembly process of nanomaterials, but also interactions between a drug and a protein or intramolecular interactions in donor‐acceptor complexes are mostly of noncovalent nature.^[^ ^309^ ^]^ Consequently, with 11 subsets and a total of 375 datapoints, this interaction type comprises a large fraction of our database. The low average reaction energy of 11.84 kcal/mol nicely illustrates why NCIs are often denoted as weak interactions.

The fact that semi‐local and hybrid‐functionals do not reproduce the correct 1/*r* ^6^‐behaviour of the London‐dispersion interaction^[^ 309, 310, 311 ^]^ has often been used as an argument for the importance of explicit electron correlation to accurately describe NCIs.^[^ 48, 132, 134, 135, 144 ^]^ Indeed, Engel et al. could show^[^ ^313^ ^]^ that the exchange‐correlation functional from second order Levy–Görling perturbation theory (LG‐PT2) contains the leading contribution to the van der Waals interaction which might have served as the main motivation for the construction of an early DH which includes an MP2 term in the long‐range regime only.^[^ ^314^ ^]^

Modern DHs can be seen as semiempirical “corrections” of LG‐PT2 and it seems to be reasonable to expect them to yield substantial improvements over hybrids and (meta‐)GGAs for NCIs. In practice however, NCIs can be described very well by partnering a GGA or a hybrid with some form of empirical dispersion correction.^[^ ^57^ ^]^ It has been pointed out by Goerigk et al.^[^ ^311^ ^]^ that a plethora of DFAs performs well on the S66 and the S66x8 test sets if only some form of empirical dispersion correction is considered.

**FIGURE 11:**
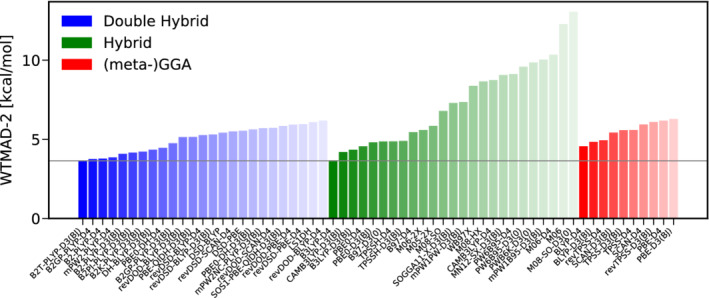
The best 25 DHs (blue) and hybrids (green) as well as the best 10 (meta‐)GGAs (red) according to their WTMAD‐2 s for the category of intermolecular noncovalent interactions [Color figure can be viewed at wileyonlinelibrary.com]

The WTMAD‐2s we present in Figure 11 partly confirm these findings. Dispersion corrections are crucial for the accurate description of NCIs. The only exception are Minnesota functionals already containing terms taking into account the interaction of overlapping electron densities of individual monomers. As already pointed out by others,^[^ 57, 154, 314 ^]^ they generally do not perform well for the present category. Many GGAs perform well here; the best GGA, BLYP‐D4, is only outperformed by four hybrid functionals, the best of them being B3LYP‐D4. In general, we find that D4 is to be preferred over D3(BJ), although the best DH is B2T‐PLYP‐D3(BJ) not being assessed in conjunction with D4. DHs do not give an improved description of NCIs, although they are more robust—the 25th ranked DH is outperformed by 11 hybrids only. With the exception of mPW2‐PLYP‐D4, the best eight DHs contain B88 exchange and LYP correlation.

**FIGURE 12:**
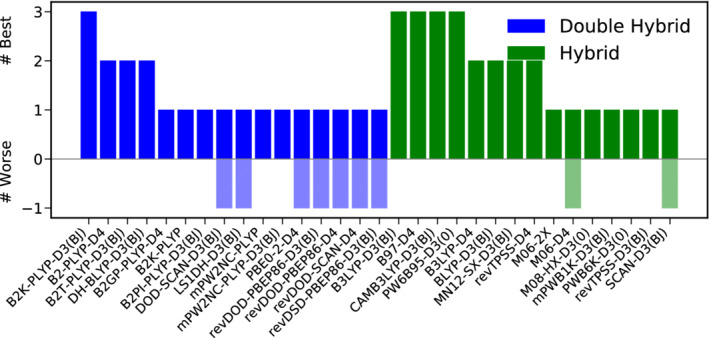
Best (full colored bars, positive x‐range)–worse (shaded bars, negative x‐range) analysis for the best 15 DHs (blue) and hybrids (green) for intermolecular noncovalent interactions [Color figure can be viewed at wileyonlinelibrary.com]

While the analysis of the WTMAD‐2s favors D4 over D3(BJ) for B3LYP, as shown in Figure 12, the latter combination is three times among the best hybrid functionals but the former only twice. Also B97‐D4, CAM‐B3LYP‐D3(BJ) and PW6B95‐D3(0) are among the best DFAs thrice. B2K‐PLYP‐D3(BJ) is the only DH being three times among the best ones of this rung on Jacob's ladder.

It is instructive to take a closer look at the important S66, S10x8 and 3B‐69‐TRIM subsets. With 215 datapoints in total, they comprise nearly two third of the NCI category and serve as a representative benchmark for NCIs between neutral organic molecules, including dimers in nonequilibrium as well as in their equilibrium geometry and trimers. B2K‐PLYP‐D3(BJ) describes noncovalently bounded complexes in their equilibrium geometry by far most accurately and its fifth position for S10x8 demonstrates that it is reliable for nonequilibrium geometries also. B3LYP‐D3(BJ) is the best hybrid functional for 3B‐69‐TRIM and S66 but cannot be found among the best 15 DFAs for S10x8. On the GGA level, we also emphasize the great performance of BLYP‐D3(BJ) for all three subsets.

For the large molecular complexes in the L7 test sets, BLYP‐D3(BJ) is the second best of all benchmarked DFAs, only being outperformed by B3LYP‐D3(BJ). As for S66 and 3B‐69‐Trim and S66x10, a DOD‐functional cannot be found among the best 15 DFAs, indicating the importance of a balanced inclusion of same‐spin and opposite‐spin correlation for the description of NCIs.

In conclusion, B2K‐PLYP‐D3(BJ) is the method of choice for the description of neutral, noncovalently bounded organic complexes, although B3LYP‐D3(BJ) and BLYP‐D3(BJ) perform quite good as well and especially the latter provides an excellent price/performance‐ratio. With the exception of these (quite important) systems, D4 dispersion correction is generally to be preferred over D3(BJ).

#### 3.4.6 The entire database

**FIGURE 13:**
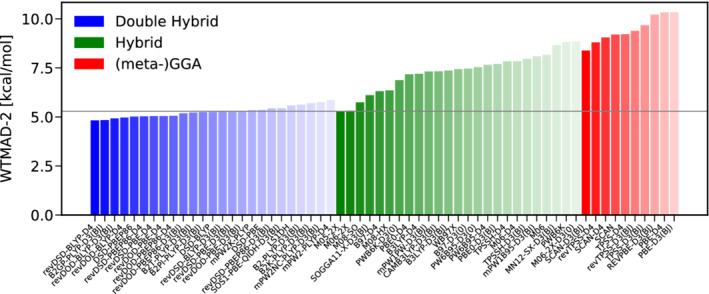
The best 25 DHs (blue) and hybrids (green) as well as the best 10 (meta‐)GGAs (red) according to their WTMAD‐2 s for the whole database [Color figure can be viewed at wileyonlinelibrary.com]

To conclude the discussion in this subsection, we shortly comment on the performance of the benchmarked DFAs for all 1,644 datapoints in our database. As for all subcategories, Figure 13 shows the WTMAD‐2 s of the 25 best performing DHs (blue) and hybrids (green) as well as the best 10 meta‐GGAs (red) together with their empirical dispersion correction.

We find that, also on the TZ level, using STOs and PARI, Jacob's ladder is still reproduced—the best hybrid functionals clearly outperform the best (meta‐)GGAs and the best DHs outperform all other functionals. However, DHs do not show the tremendous increase in accuracy over hybrids which has been observed with QZ basis sets.^[^ 57, 58 ^]^ The reasons for this, basis set incompleteness and possible PARI‐errors, have already been discussed. On the other hand, the dependence on the particular choice of DH is much smaller than for hybrids, that is, the difference of the WTMAD‐2 of the best and worse DH displayed here is only small, whereas this number is considerably larger for hybrids.

Sorting the benchmarked DHs according to their WTMAD‐2s, we cannot reproduce the rankings that have been reported for GMTKN55.^[^ 59, 61 ^]^ While the herein not assessed *ω*B97M(2) outperforms all other functionals, it is closely followed by revDSD‐PBEP86‐D4 and the same functional in its DOD‐variant and with different flavors of dispersion correction. Our benchmark favors DHs based on B88 exchange and LYP correlation, revDSD‐BLYP‐D4 being the best functional and revDSD‐PBEP86‐D4 only ranked sixth. While we can confirm the DSD‐approach (with the exception of range‐separated DHs based on *ω*B97) to be the method of choice towards the construction of robust DHs, B2GP‐PLYP‐D3(BJ), one of the first DHs ever constructed, also performs surprisingly well in our benchmark, being only average in studies on GMTKN55.^[^ ^61^ ^]^

Although our database is not identical to GMTKN55, it has considerable overlap. The highlighted differences show that care must be taken in the interpretation of benchmarks of DFAs and result obtained at the CBS limit are not necessarily transferable to smaller basis sets.

It is also interesting to observe, that the best three hybrid functionals, M05‐2X, M06‐2X and M08‐S0 do not feature an empirical dispersion‐correction term unlike, with the exception of LS1DH, the best 25 DHs. revDSD‐BLYP‐D4 outperforms all other functionals in this study, followed by B2GP‐PLYP and revDOD‐BLYP‐D3(BJ). Using PARI‐AO‐MP2, the latter one is faster than the best hybrid‐functionals M05‐2X and M06‐2X for medium systems and only negligibly slower for large molecules.

**FIGURE 14:**
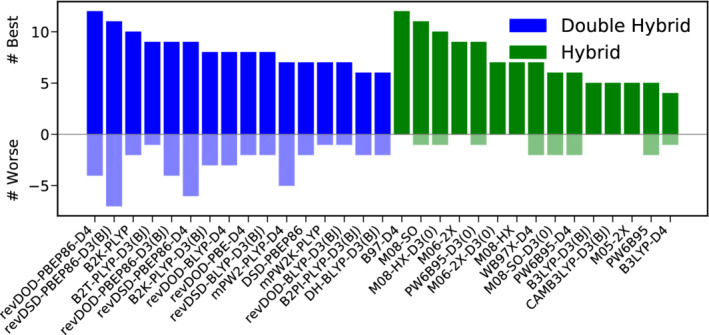
Best (full colored bars, positive x‐range)–worse (shaded bars, negative x‐range) analysis for the best 15 DHs (blue) and hybrids (green) for the whole database [Color figure can be viewed at wileyonlinelibrary.com]

However, we hesitate to recommend it as a general purpose functional. The best‐worse analysis in Figure 14 reveals that it can also be found once among the worst DHs and the same holds for all other DHs. On the hybrid level, B97‐D4 is 12 times among the best and never among the worst functionals. On the other hand, it shows a considerably worse WTMAD‐2 than all of the best 25 DHs. Given the large number of subsets investigated herein, 12 subsets out of 58 does actually not indicate great general performance: It rather means that B97‐D4 is only a mediocre choice for nearly 80% of all subsets.

Given these observations, we do not recommend a general‐purpose functional here. For different chemical problems, different functionals should be used. This insight highlights the utmost importance of the availability of high‐quality benchmarks on large and diverse datasets which can serve as a guideline for the selection of the right functional for the particular problem at hand.

## 4 CONCLUSION

In this work, we demonstrated that accurate PARI‐MP2 and PARI‐*K* calculations at the TZ level can be performed in a numerically robust way. As shown for the hybrid‐DFA B3LYP and the DH‐functional B2GP‐PLYP, the BSE of the STO‐type TZ2P basis set in conjunction with PARI is comparable to the one from the slightly larger GTO‐type def2‐TZVPP basis set used with global DF. While we obtained BSEs of 0.68 kcal/mol (0.72 for def2‐TZVPP) on average for B3LYP, the same quantity is with 1.12 kcal/mol (0.96 for def2‐TZVPP) considerably larger for B2GP‐PLYP.

These BSEs strongly suggest that the use of QZ basis sets is clearly indispensable for DHs to exploit their full potential. This can be seen most clearly for ground state energies of conformers of organic molecules with easy electronic structure and NCIs where much higher accuracy than the often cited 1 kcal/mol criterion of chemical accuracy is crucial for predictive quantum chemistry. It is the most pressing problem of PARI‐MP2, that QZ calculations are not doable at the moment. Research towards a strategy to overcome the limiting numerical issues is currently pursued in our group.

Nevertheless, we could demonstrate on a large and diverse database of 1,644 datapoints, that Jacob's ladder is still reproduced—DHs are the most accurate and robust DFAs available and many of them outperform the best hybrid functionals. Possibly due to the large BSEs for DHs and maybe also due to larger PARI‐errors in the computation of four‐center integrals, the increase in accuracy of DHs over hybrids is less pronounced than the one found in recent studies on GMTKN55.

Among the 14 DHs outperforming all hybrid functionals, also five DOD‐functionals can be found which enable DH calculations to essentially the price of a hybrid calculation. For five test sets of large molecules we could show that their accuracy is also retained for molecules of more than 100 atoms, including biologically relevant enzymes and isomers of large organic molecules. Consequently, DOD‐functionals are the method of choice when CPU‐time and memory requirements are an issue and highly accurate energies are required.

In summary, the use of DHs is recommended for various chemical problems. On the TZ level, they especially excel in thermochemistry, the calculation of barrier heights and the calculation of relative conformational energies of organic molecules with challenging electronic structure. This is not only true for main group chemistry but especially when TMs are involved. In this case, highly accurate energies can be obtained from DHs in conjunction with the ZORA formalism.

As an important results of this work, we shortly recapitulate our recommendations for the individual subcategories of our database.

1. *Basic properties and reaction energies*: DHs should always be used here. B2T‐PLYP‐D3(BJ) provides the best balance between accuracy and robustness. If CPU‐time is a concern, revDOD‐BLYP‐D3(BJ) should be used.
2. *Reaction barrier heights*: revDOD‐PBEP86‐D3(BJ) can always be recommended, but in the absence of TMs, M08‐HX is a robust and accurate alternative. When TMs are involved, B2K‐PLYP provides excellent performance.
3. *Simple isomerization reactions*: DHs cannot be recommended here. We recommend B97‐D4 due to its excellent price/performance ratio.
4. *Difficult isomerization reactions*: DOD‐functionals are the method of choice here, especially revDOD‐PBE‐D4 and revDOD‐PBEP86‐D4. Note, however, that DHs fail badly in the description of the electronic structure of fullerenes.
5. *Intermolecular noncovalent interactions*: For neutral, noncovalently bounded complexes, the DH B2K‐PLYP‐D3(BJ) can be recommended. BLYP‐D3(BJ) on the other hand offers an excellent price/performance ratio and NCIs are the only category where we can safely recommend a GGA. Empirical dispersion correction should always be used, and D4 is generally to be preferred over D3(BJ).

Apart from these findings, we could draw two other important conclusions from the results presented herein. First, a general purpose functional remains to be developed. None of the herein benchmarked functionals can safely be applied to all chemical problems assessed here. Even for a single subcategory, there is almost never a functional which can be recommended for all subsets. Thus, the recommendations given herein might serve as important guidelines but they should never be understood as black‐box solutions for a problem at hand.

Second, in our benchmarks we could not reproduce the ranking of functionals according to their accuracy on GMTKN55 when large QZ basis sets are used. While we can confirm some important results presented previously, for example, the outstanding success of the Minnesota‐functional M05‐2X, M06‐2X and M08‐HX on the hybrid level, the great performance of revDSD‐functionals^[^ 60, 61 ^]^ or the considerably worse performance of one‐parameter functionals,^[^ ^58^ ^]^ revDSD‐PBEP86‐D4 is not the frontrunner in our study but rather revDSD‐BLYP‐D4. On the other hand, the second best DFA in our study, B2GP‐PLYP, only shows an average performance on GMTKN55 with QZ basis sets. This indicates that benchmark results obtained with QZ basis sets do not necessarily apply to the TZ level also, especially for DHs, where BSEs can become quite large.

As the certainly most important outcome of this study, we conclude this work by giving a definite answer to the question asked in the very beginning—is it possible to perform accurate, robust and efficient hybrid‐ and DH‐KS calculations using STOs? We are convinced to have sufficiently demonstrated that this is indeed possible. Clearly, the numerical instability of PARI‐MP2 for QZ basis sets and larger is still an important issue which must be tackled to unleash the full potential of the PARI‐approach and research in this direction is currently pursued in our group.

PARI‐*K* and PARI‐MP2 as implemented in ADF essentially retain the accuracy of their parent algorithms and enable accurate and reliable quantum chemistry with STOs on the TZ level. At the same time, they tremendously accelerate hybrid‐ as well as DH calculations for DOD‐functionals and therefore enable routine application of both, hybrids and DHs to molecules of hundreds of atoms.

## Supporting information


**Data S1**: Supporting information
