# Supplementary material for: Double hybrid DFT calculations with Slater type orbitals
Source: J Comput Chem. 2020 Apr 16;41(18):1660–84. doi: 10.1002/jcc.26209 (PMC7317772; doi:10.1002/jcc.26209)
Supplement: Supplementary file 1 — Data S1: Supporting information [file JCC-41-1660-s001.pdf]

Supporting Information for: Double Hybrid DFT calculations with Slater Type Orbitals

Arno Förster* and Lucas Visscher

Theoretical Chemistry, Vrije Universiteit, De Boelelaan 1083, NL-1081 HV, Amsterdam, The Netherlands

E-mail: a.t.l.foerster@vu.nl

Contents

List of Tables **S2**

S1 Empirical Dispersion Parameters used in this Work **S7**

S1.1 D3(BJ)-Parameters S7

S1.2 D3(0)-Parameters S8

S1.3 D4-Parameters S8

S2 Performance of STOs and GTOs for all subsets **S9**

S3 Additional Rankings of DFAs for Subcategories according to their WTMAD-2s **S10**

S4 WTMAD-2s and MADs for all assessed functionals for all Subcategories **S12**

S5 Performance of all functionals for all Test Sets **S41**

S5.1 D3-Dispersion-Corrected Double Hybrids S41

S5.2 D4-Dispersion-Corrected Double Hybrids S83

S5.3 Dispersion-Uncorrected Double Hybrids S104

S5.4 D3-Dispersion-Corrected Hybrids S132

S5.5 D4-Dispersion-Corrected Hybrids S162

S5.6 Dispersion-Uncorrected Hybrids S179

S5.7 D3-Dispersion-Corrected (meta-)GGAs S209

S5.8 D4-Dispersion-Corrected (meta-)GGAs S219

S5.9 Dispersion-Uncorrected (meta-)GGAs S229

List of Tables

S1 WTMAD-2 values: for the whole database S7

S2 WTMAD-2 values: for the whole database S8

S3 WTMAD-2 values: for the whole database S8

S4 WTMAD-2 values: for the whole database S9

S5 WTMAD-2 values: for the whole database S12

S6 WTMAD-2 values: Noncovalent interactions S15

S7 WTMAD-2 values: Easy isomerization reactions S18

S8 WTMAD-2 values: Easy isomerization excluding PCONF and SCONF . . . S21

S9 WTMAD-2 values: Difficult Isomerization reactions S24

S10 WTMAD-2 values: Barrier heights S27

S11 WTMAD-2 values: Barrier heights without transition metals S30

S12 WTMAD-2 values: Thermochemistry S33

S13 WTMAD-2 values: Transition metals S36

S14 WTMAD-2 values: Large systems S39

S15 Functional performance: B2-PLYP-D3(BJ) S41

S16 Functional performance: B2GP-PLYP-D3(BJ) S42

S17 Functional performance: B2K-PLYP-D3(BJ) S44

S18 Functional performance: B2T-PLYP-D3(BJ) S46

S19 Functional performance: B2π-PLYP-D3(BJ) S47

S20 Functional performance: B2NC-PLYP-D3(BJ) S49

S21 Functional performance: mPW2-PLYP-D3(BJ) S50

S22 Functional performance: mPW2NC-PLYP-D3(BJ) S52

S23 Functional performance: mPW2K-PLYP-D3(BJ) S53

S24 Functional performance: PBE0-DH-D3(BJ) S55

S25 Functional performance: PBE0-2-D3(BJ) S56

S26 Functional performance: LS1TPSS-D3(BJ) S58

S27 Functional performance: LS1DH-D3(BJ) S60

S28 Functional performance: DS1TPSS-D3(BJ) S61
S29 Functional performance: DH-BLYP-D3(BJ) S63
S30 Functional performance: PBE-QIDH-D3(BJ) S64
S31 Functional performance: SOS1-PBE-QIDH-D3(BJ) S66
S32 Functional performance: DSD-BLYP S67
S33 Functional performance: revDSD-BLYP-D3(BJ) S69
S34 Functional performance: revDOD-BLYP-D3(BJ) S71
S35 Functional performance: DSD-PBEP86 . . . . S72
S36 Functional performance: revDSD-PBEP86-D3(BJ) S74
S37 Functional performance: revDOD-PBEP86-D3(BJ) S75
S38 Functional performance: DSD-PBE S77
S39 Functional performance: revDSD-PBE-D3(BJ) S79
S40 Functional performance: revDOD-PBE-D3(BJ) S80
S41 Functional performance: DOD-SCAN-D3(BJ) S82
S42 Functional performance: B2-PLYP-D4 . . . . S84
S43 Functional performance: B2GP-PLYP-D4 . . . . S85
S44 Functional performance: mPW2-PLYP-D4 . . . . S87
S45 Functional performance: PBE0-DH-D4 . . . . S88
S46 Functional performance: PBE0-2-D4 . . . . S90
S47 Functional performance: revDSD-BLYP-D4 . . . . S91
S48 Functional performance: revDOD-BLYP-D4 . . . . S93
S49 Functional performance: revDSD-PBEP86-D4 . . . . S95
S50 Functional performance: revDOD-PBEP86-D4 . . . . S96
S51 Functional performance: revDSD-PBE-D4 . . . . S98
S52 Functional performance: revDOD-PBE-D4 . . . . S99
S53 Functional performance: revDSD-SCAN-D4 . . . . S101
S54 Functional performance: revDOD-SCAN-D4 . . . . S103
S55 Functional performance: B2-PLYP S104
S56 Functional performance: B2GP-PLYP S106

S57 Functional performance: B2K-PLYP S107
S58 Functional performance: B2T-PLYP S109
S59 Functional performance: B2π-PLYP S111
S60 Functional performance: B2NC-PLYP S112
S61 Functional performance: mPW2-PLYP S114
S62 Functional performance: mPW2NC-PLYP S115
S63 Functional performance: mPW2K-PLYP S117
S64 Functional performance: PBE0-DH S118
S65 Functional performance: PBE0-2 . . . . S120
S66 Functional performance: LS1TPSS S121
S67 Functional performance: LS1DH S123
S68 Functional performance: DS1TPSS S125
S69 Functional performance: DH-BLYP S126
S70 Functional performance: PBE-QIDH S128
S71 Functional performance: SOS1-PBE-QIDH S129
S72 Functional performance: SDSCAN69 . . . . S131
S73 Functional performance: B3LYP-D3(BJ) S133
S74 Functional performance: CAMB3LYP-D3(BJ) S134
S75 Functional performance: B97-D3(0) S136
S76 Functional performance: WB97X-D3(0) S138
S77 Functional performance: PBE0-D3(BJ) S139
S78 Functional performance: TPSSH-D3(BJ) S141
S79 Functional performance: PW6B95-D3(0) S143
S80 Functional performance: mPWB1K-D3(BJ) S144
S81 Functional performance: mPW1B95-D3(BJ) S146
S82 Functional performance: mPW1PW-D3(BJ) S148
S83 Functional performance: PWB6K-D3(0) S149
S84 Functional performance: SOGGA11-X-D3(BJ) S151
S85 Functional performance: M05-2X-D3(0) S153

S86 Functional performance: M06-D3(0) S154
S87 Functional performance: M06-2X-D3(BJ) S156
S88 Functional performance: M08-SO-D3(0) S158
S89 Functional performance: M08-HX-D3(0) S159
S90 Functional performance: MN12-SX-D3(BJ) S161
S91 Functional performance: B3LYP-D4 . . . . S163
S92 Functional performance: CAMB3LYP-D4 . . . . S164
S93 Functional performance: B97-D4 . . . . S166
S94 Functional performance: WB97X-D4 . . . . S168
S95 Functional performance: PBE0-D4 . . . . S169
S96 Functional performance: TPSSH-D4 . . . . S171
S97 Functional performance: PW6B95-D4 . . . . S173
S98 Functional performance: mPWB1K-D4 . . . . S174
S99 Functional performance: mPW1PW-D4 . . . . S176
S100 Functional performance: M06-D4 . . . . S178
S101 Functional performance: B3LYP S179
S102 Functional performance: CAMB3LYP S181
S103 Functional performance: B97 . . . . S183
S104 Functional performance: WB97X S184
S105 Functional performance: PBE0 . . . . S186
S106 Functional performance: TPSSH S188
S107 Functional performance: PW6B95 . . . . S189
S108 Functional performance: mPWB1K S191
S109 Functional performance: mPW1B95 . . . . S193
S110 Functional performance: mPW1PW S194
S111 Functional performance: PWB6K S196
S112 Functional performance: SOGGA11-X S198
S113 Functional performance: M05-2X S199
S114 Functional performance: M06 . . . . S201

S115 Functional performance: M06-2X S203
S116 Functional performance: M08-SO S204
S117 Functional performance: M08-HX S206
S118 Functional performance: MN12-SX S208
S119 Functional performance: BLYP-D3(BJ) S209
S120 Functional performance: PBE-D3(BJ) S211
S121 Functional performance: REVPBE-D3(BJ) S213
S122 Functional performance: TPSS-D3(BJ) S214
S123 Functional performance: revTPSS-D3(BJ) S216
S124 Functional performance: SCAN-D3(BJ) S218
S125 Functional performance: BLYP-D4 . . . . S219
S126 Functional performance: PBE-D4 . . . . S221
S127 Functional performance: REVPBE-D4 . . . . S223
S128 Functional performance: TPSS-D4 . . . . S224
S129 Functional performance: revTPSS-D4 . . . . S226
S130 Functional performance: SCAN-D4 . . . . S228
S131 Functional performance: BLYP S229
S132 Functional performance: PBE S231
S133 Functional performance: REVPBE S233
S134 Functional performance: TPSS S234
S135 Functional performance: revTPSS S236
S136 Functional performance: SCAN S238

S1 Empirical Dispersion Parameters used in this Work

S1.1 D3(BJ)-Parameters

Table S1: WTMAD-2 and MAD values of all benchmarked DFAs for for the whole database separated for different rungs on Jacobs ladder in descending order. All energies in kcal/mol

Functional	Parameters				
	s6	a1	s8	a2	α
revDSD-PBE	0.5746	0.0	0.0	5.5	14.0
revDSD-PBEP86	0.4377	0.0	0.0	5.5	14.0
revDSD-BLYP	0.5451	0.0	0.0	5.2	14.0
revDOD-PBE	0.6067	0.0	0.0	5.5	14.0
revDOD-PBEP86	0.4770	0.0	0.0	5.5	14.0
revDOD-BLYP	0.6145	0.0	0.0	5.2	14.0
DSD-PBE	0.7800	0.0	0.0	6.1	14.0
DSD-PBEP86	0.4800	0.0	0.0	5.6	14.0
DSD-BLYP	0.500	0.0	0.2130	6.0519	14.0
DOD-SCAN	0.3152	0.0	0.0	5.75	14.0
PBE-QIDH	0.610	0.114	0.566	7.538	14.2
SOS1-PBEQIDH	0.750	0.002	0.200	6.794	8.1
PBE0-DH	0.880	0.0	0.165	6.385	14.0
PBE0-2	0.5400	0.0	0.515	8.345	14.0
mPW2-PLYP	0.40	0.3065	0.9147	5.0570	14.0
mPW2NC-PLYP	0.385	-0.493	0.0	9.529	14.0
mPW2K-PLYP	0.490	0.306	0.915	5.057	14.0
LS1-TPSS	0.305	1.941	0.660	0.053	14.0
LS1-DH	0.508	0.387	1.073	6.747	14.0
DS1TPSS	0.384	0.306	0.915	5.057	14.0
DHBLYP	0.488	0.306	0.915	5.057	14.0
B2T-PLYP	0.640	0.3065	0.9147	5.0570	14.0
B2-PLYP	0.640	0.306	0.915	5.057	14.0
B2π-PLYP	0.637	0.306	0.915	5.057	14.0
B2NC-PLYP	0.363	0.609	0.616	5.354	14.0
B2K-PLYP	0.490	0.306	0.915	5.057	14.0
B2GP-PLYP	0.56	0.00	0.2597	6.3332	14.0
revTPSS	1.0	0.429	0.788	4.441	14.0
TPSSH	1.0	0.453	2.238	4.655	14.0
SOGGA11-X	1.0	0.429	0.788	4.441	14.0
SCAN	1.0	0.538	0.0	5.420	14.0
REVPBE	1.0	0.524	2.355	3.502	14.0
PBE0	1.0	0.414	1.218	4.859	14.0
mPWB1K	1.0	0.429	0.788	4.441	14.0
mPW1B95	1.0	0.196	1.051	6.418	14.0

Continued on next page

Functional	Parameters				
	s6	a1	s8	a2	α
CAMB3LYP	1.0	0.398	1.989	4.421	14.0
BLYP	1.0	0.430	2.700	4.236	14.0
B3LYP	1.0	0.398	1.989	4.421	14.0
TPSS	1.0	0.454	1.944	4.475	14.0
PBE	1.0	0.429	0.788	4.441	14.0
MN12-SX	1.0	0.429	0.788	4.441	14.0
mPW1PW	1.0	0.429	0.788	4.441	14.0

S1.2 D3(0)-Parameters

Table S2: WTMAD-2 and MAD values of all benchmarked DFAs for for the whole database separated for different rungs on Jacobs ladder in descending order. All energies in kcal/mol

Functional	Parameters		
	s6	sr,6	s8
ωB97-X	1.0	1.217	0.722
PW6B95	1.0	1.532	0.862
M08-HX	1.0	1.217	0.722
M06	1.0	1.217	0.722
M06-2X	1.0	1.217	0.722
M05-2X	1.0	1.217	0.722
B97	1.0	1.217	0.722
M08-SO	1.0	1.217	0.722
PWB6K	1.0	1.66	0.55

S1.3 D4-Parameters

Table S3: WTMAD-2 and MAD values of all benchmarked DFAs for for the whole database separated for different rungs on Jacobs ladder in descending order. All energies in kcal/mol

Functional	Parameters			
	s6	a1	s8	a2
revDSD-SCAN	0.3203	0.4	0.0	3.6
revDSD-PBEP86	0.5132	0.44	0.0	3.60
revDSD-PBE	0.6706	0.4	0.0	3.60
revDSD-BLYP	0.6141	0.38	0.0	3.52
revDOD-SCAN	0.3270	0.4	0.0	3.6

Continued on next page

Functional	Parameters			
	s6	a1	s8	a2
revDOD-PBEP86	0.5552	0.44	0.0	3.6
revDOD-PBE	0.6992	0.4	0.0	3.6
revDOD-BLYP	0.7105	0.38	0.0	3.52
B2-PLYP	0.640	0.4415	1.1689	4.7311
B2GP-PLYP	0.560	0.4291	0.9463	5.188
mPW2-PLYP	0.750	0.4300	0.4579	5.0765
PBE02	0.5000	0.7654	0.6430	5.7858
PBE0DH	0.8750	0.4759	0.9681	5.0862
mPW1PW	1.0	0.4246	1.8066	4.6813
B3LYP	1.0	0.4028	2.0025	4.5278
CAM-B3LYP	1.0	0.4014	1.7441	5.1873
PBE0	1.0	0.3991	1.2683	5.0395
TPSSH	1.0	0.4457	1.5254	4.6988
B97	1.0	0.2932	0.8785	4.5165
mPWB1K	1.0	0.4422	0.6222	5.2132
PW6B95	1.0	0.0400	-0.3163	5.8369
M06	1.0	0.5293	0.2295	6.0652
WB97X	1.0	0.4745	0.3882	6.9137
SCAN	1.0	0.6357	1.7541	6.3569
TPSS	1.0	0.4282	1.7660	4.5426
revTPSS	1.0	0.4424	1.5186	4.6288
BLYP	1.0	0.4473	2.3397	4.0658
PBE	1.0	0.4993	3.6532	4.2429
REVPBE	1.0	0.5403	1.6254	2.9797

S2 Performance of STOs and GTOs for all subsets

Table S4: MADs for dispersion-uncorrected B3LYP and B2GP-PLYP using TZ2P (ADF) as well as def2-TZVPP (psi4) with respect to the CBS limit (def2-QZVPP, psi4). In the last four lines, the MADs and WTMAD2s with respect to the high-level reference values are given as well as with respect to the CBS-limit of the respective functional. All energies in kcal/mol.

	B3LYP		B2GP-PLYP			
subset	def2-TZVPP	TZ2P	def2-TZVPP	TZ2P	#	$\lvert\overline{\Delta E}\rvert$
DIPCS10	3.79	1.05	1.14	0.81	10	654.26
W4-11	1.16	1.94	2.53	2.91	140	306.91.
G21IP	1.93	2.59	0.82	0.87	36	257.61
ALK8	0.60	0.53	1.28	1.62	8	62.60

Continued on next page

	B3LYP		B2GP-PLYP			
subset	def2-TZVPP	TZ2P	def2-TZVPP	TZ2P	#	$\lvert\overline{\Delta E}\rvert$
G2RC	0.73	0.47	0.94	0.93	25	51.26
BHDIV10	0.17	0.24	0.21	0.49	10	45.33
AL2X6	2.00	2.04	1.68	2.00	6	35.88
WCPT18	0.58	0.36	0.49	1.11	18	34.99
G21EA	6.34	2.71	8.37	5.05	25	33.62
INV24	0.73	0.39	0.92	1.27	24	32.85
DARC	0.79	0.47	1.35	2.22	14	32.47
FH51	0.45	0.27	0.51	0.89	51	31.01
NBPRC	0.45	0.42	0.50	0.81	12	27.71
BHPERI	0.27	0.17	0.35	0.47	26	20.87
BH76	1.32	1.10	0.72	0.78	76	18.61
BSR36	0.11	0.24	1.44	2.88	36	16.20
ISO34	0.12	0.21	0.18	0.33	34	14.57
RSE43	0.15	0.22	0.22	0.21	43	7.60
BHROT27	0.06	0.09	0.10	0.15	27	6.37
S66	0.14	0.16	0.43	0.52	66	5.47
MCONF	0.32	0.29	0.38	0.72	51	4.97
PArel	0.14	0.15	0.24	0.41	20	4.63
SCONF	0.34	0.19	0.32	0.56	17	4.60
PNICO23	0.36	0.12	0.20	0.19	23	4.27
ICONF	0.35	0.38	0.37	0.42	17	3.27
TAUT15	0.09	0.18	0.16	0.35	15	3.05
BUT14DIOL	0.22	0.11	0.25	0.49	64	2.80
Amino20x4	0.08	0.07	0.10	0.18	80	2.44
ACONF	0.03	0.05	0.19	0.09	15	1.83
PCONF21	0.19	0.08	0.23	0.63	18	1.62
MAD (high-level ref.)	3.15	3.32	2.06	1.89		
WTMAD2 (high-level ref.)	14.61	14.98	5.82	5.52		
MAD (CBS)	0.72	0.68	0.96	1.12		
WTMAD2 (CBS)	2.11	1.63	2.73	4.05		

S3 Additional Rankings of DFAs for Subcategories according to their WTMAD-2s

Figure S1: The best 25 DHs (blue) and hybrids (green) as well as the best 10 (meta-)GGAs (red) according to their WTMAD-2s for easy isomerization reactions without PCONF and SCONF.

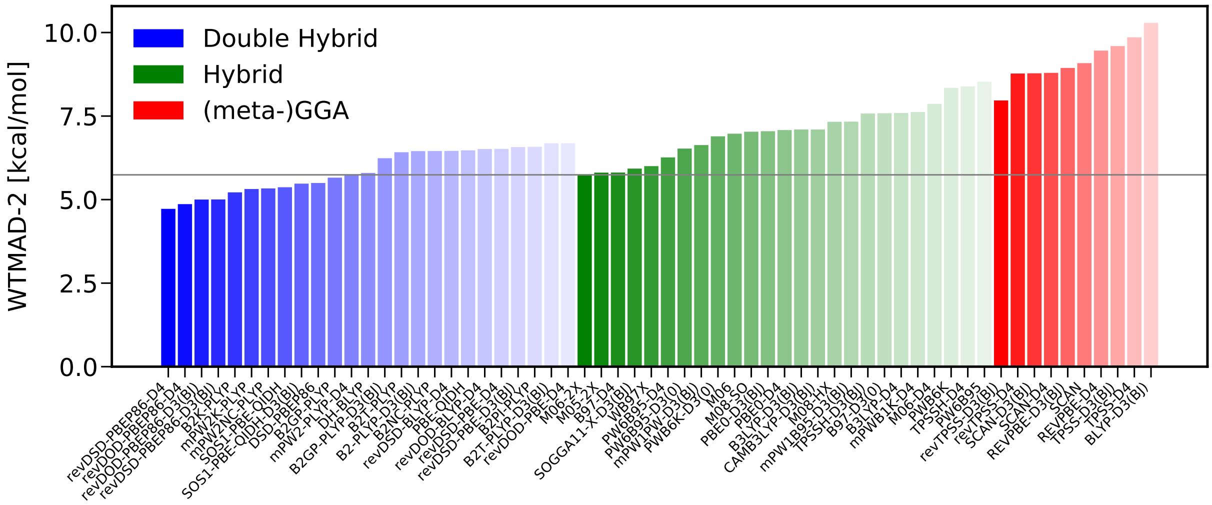

Figure S2: The best 25 DHs (blue) and hybrids (green) as well as the best 10 (meta-)GGAs (red) according to their WTMAD-2s for barrier heights without transition metal containing systems.

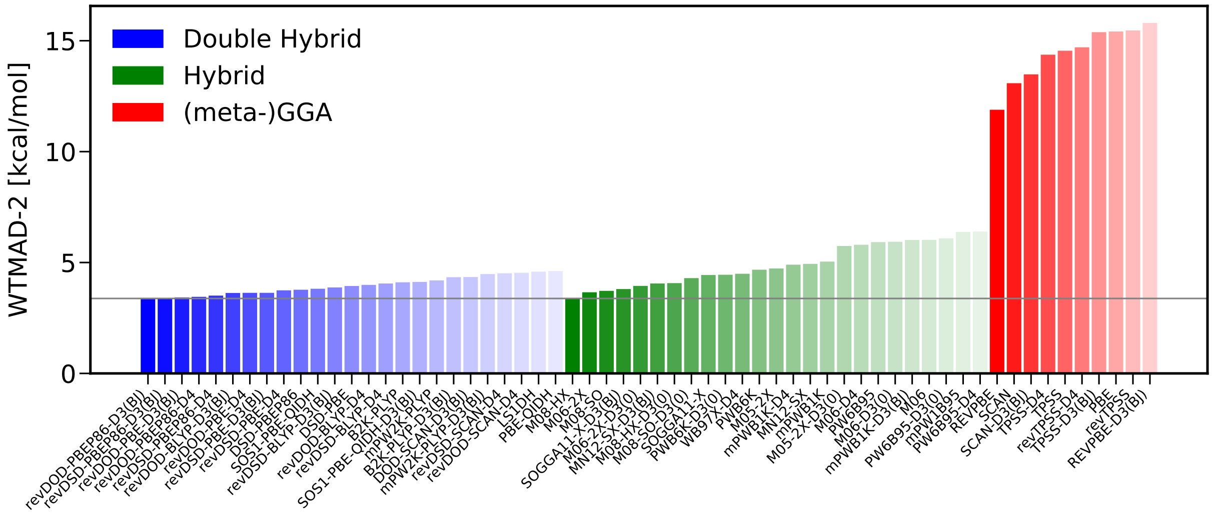

Figure S3: The best 25 DHs (blue) and hybrids (green) as well as the best 10 (meta-)GGAs (red) according to their WTMAD-2s for all transition metal containing systems.

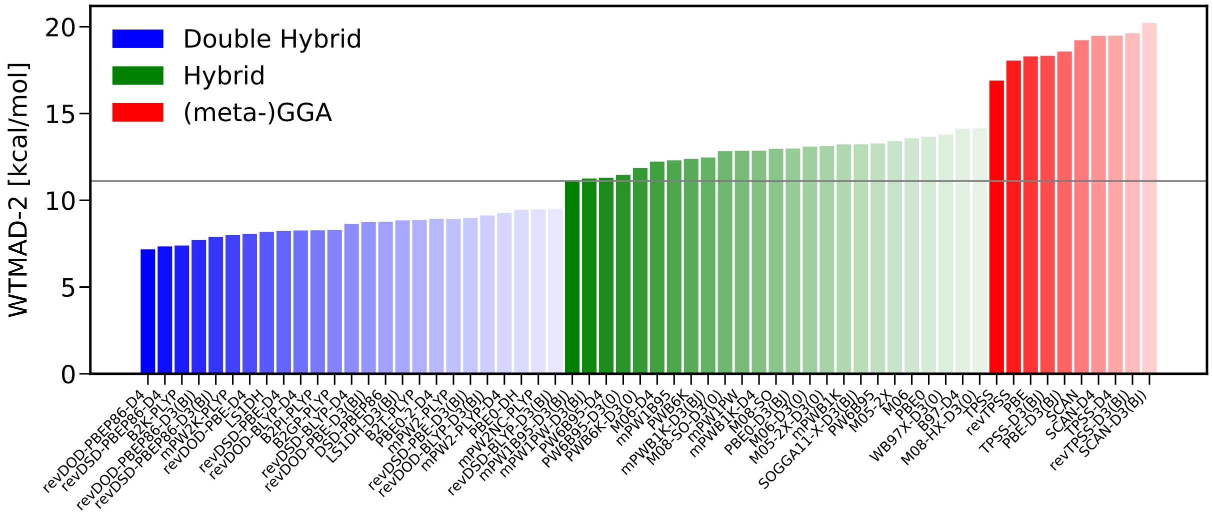

S4 WTMAD-2s and MADs for all assessed functionals for all Subcategories

Table S5: WTMAD-2 and MAD values of all benchmarked DFAs for the whole database separated for different rungs on Jacobs ladder in descending order. All energies in kcal/mol

Functional	WTMAD-2	MAD
Double hybrids		
revDSD-BLYP-D4	4.81	1.63
B2GP-PLYP-D3(BJ)	4.83	1.77
revDOD-BLYP-D3(BJ)	4.91	1.56
revDOD-BLYP-D4	4.96	1.55
DSD-PBEP86	5.01	2.09
revDSD-PBEP86-D4	5.02	2.10
revDSD-PBE-D4	5.02	1.79
revDOD-PBE-D4	5.03	1.75
revDOD-PBEP86-D4	5.05	2.06
revDOD-PBEP86-D3(BJ)	5.18	2.05
B2T-PLYP-D3(BJ)	5.21	1.80
B2π-PLYP-D3(BJ)	5.23	1.67
DSD-BLYP	5.25	1.82
revDSD-BLYP-D3(BJ)	5.25	1.67
revDSD-PBE-D3(BJ)	5.27	1.83
revDOD-PBE-D3(BJ)	5.27	1.73
mPW2K-PLYP	5.33	1.93

Continued on next page

Functional	WTMAD-2	MAD
DSD-PBE	5.35	1.74
revDSD-PBEP86-D3(BJ)	5.42	2.13
SOS1-PBE-QIDH-D3(BJ)	5.43	2.49
LS1DH	5.57	1.98
B2-PLYP-D3(BJ)	5.61	1.86
B2K-PLYP-D3(BJ)	5.69	1.93
mPW2NC-PLYP-D3(BJ)	5.74	2.13
mPW2-PLYP-D4	5.85	2.01
SDSCAN69	5.89	1.76
B2K-PLYP	6.05	2.04
PBE-QIDH-D3(BJ)	6.07	2.54
DH-BLYP-D3(BJ)	6.10	1.76
B2GP-PLYP-D4	6.27	1.98
mPW2NC-PLYP	6.37	2.33
B2-PLYP-D4	6.44	1.98
PBE0-DH-D3(BJ)	6.51	2.41
PBE0-DH-D4	6.59	2.39
mPW2K-PLYP-D3(BJ)	6.60	1.99
PBE0-2	6.61	2.26
revDSD-SCAN-D4	6.62	1.87
B2GP-PLYP	6.68	2.10
DS1TPSS	6.72	1.93
PBE-QIDH	6.73	2.55
revDOD-SCAN-D4	6.74	1.89
B2NC-PLYP-D3(BJ)	6.74	2.01
DH-BLYP	6.97	1.95
mPW2-PLYP-D3(BJ)	7.00	2.06
DOD-SCAN-D3(BJ)	7.01	1.89
LS1DH-D3(BJ)	7.01	2.19
LS1TPSS	7.03	2.34
B2NC-PLYP	7.05	2.10
SOS1-PBE-QIDH	7.31	2.61
LS1TPSS-D3(BJ)	7.35	2.39
B2π-PLYP	7.49	2.06
DS1TPSS-D3(BJ)	7.55	2.11
PBE0-2-D3(BJ)	7.59	2.43
mPW2-PLYP	7.62	2.40
B2T-PLYP	8.03	2.31
PBE0-DH	8.61	2.56
B2-PLYP	9.39	2.49
PBE0-2-D4	12.54	4.10
hybrids		
M05-2X	5.29	2.29
M06-2X	5.30	1.92

Continued on next page

Functional	WTMAD-2	MAD
M08-SO	5.72	2.13
SOGGA11-X-D3(BJ)	6.10	2.12
B97-D4	6.30	2.03
M08-HX	6.34	2.03
PWB6K-D3(0)	6.86	2.80
PBE0-D4	7.16	2.58
B3LYP-D4	7.19	2.82
mPW1PW-D3(BJ)	7.30	2.65
CAMB3LYP-D3(BJ)	7.31	2.91
B3LYP-D3(BJ)	7.35	3.03
WB97X	7.42	2.64
B97-D3(0)	7.45	2.49
PW6B95-D3(0)	7.52	2.41
PW6B95-D4	7.65	2.50
PBE0-D3(BJ)	7.68	2.76
TPSSH-D4	7.82	2.70
M06-D4	7.83	2.46
TPSSH-D3(BJ)	7.94	2.77
mPW1B95-D3(BJ)	8.07	2.77
M06	8.15	2.63
MN12-SX-D3(BJ)	8.65	2.31
PWB6K	8.80	3.07
M06-2X-D3(0)	8.84	2.16
M05-2X-D3(0)	8.92	2.66
M08-SO-D3(0)	9.04	2.39
MN12-SX	9.16	2.66
CAMB3LYP-D4	9.27	3.07
M08-HX-D3(0)	9.56	2.30
WB97X-D4	9.71	2.65
M06-D3(0)	10.06	2.72
PW6B95	10.26	2.82
mPWB1K-D4	10.47	3.03
mPWB1K-D3(BJ)	10.69	3.18
mPW1B95	10.73	3.12
WB97X-D3(0)	10.77	2.91
SOGGA11-X	11.52	2.88
PBE0	12.04	3.23
mPWB1K	12.55	3.43
mPW1PW	13.66	3.50
B97	13.91	3.31
TPSSH	14.77	3.67
CAMB3LYP	16.67	4.39
B3LYP	16.76	4.52
(meta-)GGAs		

Continued on next page

Functional	WTMAD-2	MAD
SCAN-D3(BJ)	8.37	2.82
revTPSS-D4	8.79	3.09
SCAN-D4	9.04	2.96
SCAN	9.18	2.90
TPSS-D4	9.20	3.05
revTPSS-D3(BJ)	9.38	3.29
TPSS-D3(BJ)	9.66	3.45
REVPBE-D3(BJ)	10.20	3.35
PBE-D4	10.31	4.29
PBE-D3(BJ)	10.32	4.30
BLYP-D4	10.36	4.14
BLYP-D3(BJ)	10.40	4.23
REVPBE-D4	10.47	3.44
PBE	14.30	4.78
revTPSS	14.80	3.91
TPSS	16.29	4.29
REVPBE	19.65	4.59
BLYP	21.52	5.86

Table S6: WTMAD-2 and MAD values of all benchmarked DFAs for noncovalent interactions separated for different rungs on Jacobs ladder in descending order. All energies in kcal/mol

Functional	WTMAD-2	MAD
Double hybrids		
B2T-PLYP-D3(BJ)	3.64	0.33
B2GP-PLYP-D4	3.73	0.39
B2-PLYP-D4	3.76	0.41
mPW2-PLYP-D4	3.84	0.40
B2-PLYP-D3(BJ)	4.07	0.41
B2π-PLYP-D3(BJ)	4.14	0.39
B2K-PLYP-D3(BJ)	4.21	0.33
DH-BLYP-D3(BJ)	4.33	0.35
PBE0-DH-D4	4.45	0.50
B2GP-PLYP-D3(BJ)	4.74	0.48
PBE-QIDH-D3(BJ)	5.11	0.60
revDOD-BLYP-D3(BJ)	5.13	0.54
revDSD-BLYP-D4	5.25	0.57
revDSD-BLYP-D3(BJ)	5.29	0.57
DSD-BLYP	5.40	0.55
revDSD-SCAN-D4	5.49	0.45
DSD-PBE	5.53	0.50
PBE0-DH-D3(BJ)	5.61	0.53

Continued on next page

Functional	WTMAD-2	MAD
mPW2NC-PLYP-D3(BJ)	5.69	0.59
revDOD-SCAN-D4	5.71	0.48
SOS1-PBE-QIDH-D3(BJ)	5.83	0.76
revDOD-PBE-D4	5.91	0.64
revDSD-PBE-D4	5.94	0.65
LS1DH	6.07	0.72
revDOD-BLYP-D4	6.17	0.63
DOD-SCAN-D3(BJ)	6.20	0.51
DS1TPSS-D3(BJ)	6.41	0.47
DSD-PBEP86	6.77	0.77
PBE0-2-D3(BJ)	6.81	0.55
revDSD-PBE-D3(BJ)	6.85	0.74
B2NC-PLYP-D3(BJ)	6.91	0.72
revDOD-PBE-D3(BJ)	7.00	0.75
mPW2K-PLYP	7.04	0.87
mPW2K-PLYP-D3(BJ)	7.29	0.63
SDSCAN69	7.60	0.91
PBE0-2	7.65	0.88
LS1DH-D3(BJ)	7.77	0.64
revDSD-PBEP86-D3(BJ)	8.37	1.02
LS1TPSS	8.51	0.97
revDSD-PBEP86-D4	8.53	1.01
revDOD-PBEP86-D4	8.54	1.01
revDOD-PBEP86-D3(BJ)	8.72	1.05
LS1TPSS-D3(BJ)	8.76	0.80
PBE0-2-D4	8.99	0.73
mPW2-PLYP-D3(BJ)	9.07	0.91
PBE-QIDH	9.24	1.07
DS1TPSS	9.45	1.11
mPW2NC-PLYP	9.49	1.16
mPW2-PLYP	10.07	1.20
B2K-PLYP	10.23	1.19
B2NC-PLYP	10.99	1.29
DH-BLYP	11.82	1.37
B2π-PLYP	12.12	1.36
B2GP-PLYP	12.37	1.41
PBE0-DH	12.97	1.46
SOS1-PBE-QIDH	14.02	1.61
B2T-PLYP	14.05	1.58
B2-PLYP	16.17	1.80
hybrids		
B3LYP-D4	3.65	0.38
CAMB3LYP-D3(BJ)	4.18	0.37
B3LYP-D3(BJ)	4.32	0.89

Continued on next page

Functional	WTMAD-2	MAD
PBE0-D4	4.54	0.53
PBE0-D3(BJ)	4.79	0.53
B97-D3(0)	4.85	0.54
TPSSH-D4	4.85	0.46
TPSSH-D3(BJ)	4.89	0.47
B97-D4	5.44	0.43
M06-2X	5.57	0.63
M05-2X	5.83	0.73
M08-SO	6.78	0.80
SOGGA11-X-D3(BJ)	7.28	0.67
mPW1PW-D3(BJ)	7.34	0.79
WB97X	8.36	0.72
M08-HX	8.64	0.82
CAMB3LYP-D4	8.72	0.92
MN12-SX-D3(BJ)	9.05	0.80
PW6B95-D4	9.10	1.06
PW6B95-D3(0)	9.57	1.09
PWB6K-D3(0)	9.84	0.80
mPW1B95-D3(BJ)	10.02	1.26
M06-D4	10.33	0.71
M06	12.27	1.19
M08-SO-D3(0)	13.04	1.38
M06-D3(0)	13.42	1.14
WB97X-D4	13.51	1.11
MN12-SX	13.61	1.45
M06-2X-D3(0)	13.82	1.59
PWB6K	14.67	1.38
M05-2X-D3(0)	14.78	1.57
M08-HX-D3(0)	15.63	1.47
PBE0	16.02	1.81
PW6B95	17.31	2.27
mPW1B95	18.00	2.44
WB97X-D3(0)	19.86	1.88
mPWB1K-D3(BJ)	20.97	1.21
B97	21.64	2.44
mPWB1K-D4	22.27	1.17
TPSSH	23.24	2.57
SOGGA11-X	23.78	2.45
mPW1PW	24.11	2.59
CAMB3LYP	27.12	2.88
B3LYP	27.46	3.44
mPWB1K	28.35	2.24
(meta-)GGAs		
BLYP-D4	4.55	0.43

Continued on next page

Functional	WTMAD-2	MAD
BLYP-D3(BJ)	4.82	0.38
revTPSS-D4	4.92	0.44
SCAN-D3(BJ)	5.41	0.68
TPSS-D3(BJ)	5.56	0.53
TPSS-D4	5.57	0.51
SCAN-D4	5.92	0.63
revTPSS-D3(BJ)	6.08	0.64
PBE-D4	6.16	0.62
PBE-D3(BJ)	6.27	0.62
SCAN	8.03	0.96
REVPBE-D4	9.19	0.63
REVPBE-D3(BJ)	9.86	0.69
PBE	16.05	1.87
revTPSS	21.45	2.42
TPSS	23.77	2.65
REVPBE	33.89	4.00
BLYP	34.05	3.64

Table S7: WTMAD-2 and MAD values of all benchmarked DFAs for easy isomerization reactions separated for different rungs on Jacobs ladder in descending order. All energies in kcal/mol

Functional	WTMAD-2	MAD
Double hybrids		
revDSD-PBEP86-D4	5.40	0.34
revDOD-PBEP86-D4	5.65	0.35
revDOD-PBEP86-D3(BJ)	5.92	0.36
SOS1-PBE-QIDH-D3(BJ)	6.20	0.44
SOS1-PBE-QIDH	6.61	0.44
DSD-PBEP86	6.72	0.41
B2GP-PLYP	6.80	0.43
B2GP-PLYP-D3(BJ)	7.26	0.48
PBE-QIDH	7.37	0.52
PBE-QIDH-D3(BJ)	7.70	0.56
mPW2K-PLYP	7.89	0.57
revDOD-BLYP-D4	7.93	0.50
revDSD-BLYP-D4	7.95	0.49
B2K-PLYP	7.95	0.56
revDSD-PBEP86-D3(BJ)	8.09	0.55
mPW2NC-PLYP	8.11	0.60
revDSD-PBE-D4	8.22	0.49
revDSD-PBE-D3(BJ)	8.29	0.49
DSD-BLYP	8.49	0.54

Continued on next page

Functional	WTMAD-2	MAD
revDOD-PBE-D4	8.49	0.50
revDOD-PBE-D3(BJ)	8.49	0.50
DH-BLYP	8.75	0.62
SDSCAN69	8.93	0.56
B2NC-PLYP	9.22	0.66
revDOD-BLYP-D3(BJ)	9.28	0.55
DS1TPSS	9.46	0.62
DSD-PBE	9.68	0.57
B2-PLYP-D3(BJ)	9.76	0.72
LS1DH	10.02	0.70
B2T-PLYP-D3(BJ)	10.33	0.72
B2T-PLYP	10.48	0.70
mPW2NC-PLYP-D3(BJ)	10.49	0.76
LS1TPSS	10.51	0.67
B2π-PLYP-D3(BJ)	10.53	0.71
revDSD-BLYP-D3(BJ)	10.55	0.69
PBE0-2	10.61	0.77
B2π-PLYP	10.62	0.69
PBE0-DH-D3(BJ)	10.70	0.79
mPW2-PLYP	11.26	0.76
LS1TPSS-D3(BJ)	11.36	0.72
mPW2-PLYP-D4	12.18	0.77
B2-PLYP	12.25	0.80
mPW2-PLYP-D3(BJ)	12.33	0.83
B2NC-PLYP-D3(BJ)	12.66	0.87
B2K-PLYP-D3(BJ)	12.72	0.80
DH-BLYP-D3(BJ)	12.95	0.85
PBE0-DH-D4	13.40	0.84
mPW2K-PLYP-D3(BJ)	13.57	0.84
PBE0-2-D4	13.63	0.87
PBE0-DH	13.65	0.88
B2-PLYP-D4	13.84	0.85
LS1DH-D3(BJ)	14.18	0.95
revDSD-SCAN-D4	14.64	0.81
B2GP-PLYP-D4	14.79	0.87
revDOD-SCAN-D4	14.82	0.82
DS1TPSS-D3(BJ)	14.98	0.90
PBE0-2-D3(BJ)	15.05	1.02
DOD-SCAN-D3(BJ)	15.93	0.87
	hybrids	
B97-D4	5.98	0.43
M05-2X	6.29	0.44
SOGGA11-X-D3(BJ)	6.66	0.46
PW6B95-D4	6.87	0.48

Continued on next page

Functional	WTMAD-2	MAD
WB97X	7.03	0.46
PW6B95-D3(0)	7.10	0.50
M06	7.20	0.51
M06-2X	7.23	0.47
PWB6K-D3(0)	7.49	0.52
B3LYP-D3(BJ)	7.71	0.60
CAMB3LYP-D3(BJ)	7.71	0.60
B3LYP-D4	7.82	0.62
mPW1B95-D3(BJ)	8.01	0.55
PBE0-D4	8.09	0.57
M08-SO	8.28	0.51
PBE0-D3(BJ)	8.36	0.57
mPWB1K-D4	8.39	0.58
M08-HX	8.75	0.51
mPW1PW-D3(BJ)	9.08	0.56
TPSSH-D3(BJ)	9.28	0.69
B97-D3(0)	9.29	0.60
M06-D4	9.45	0.61
TPSSH-D4	9.58	0.73
PWB6K	9.68	0.64
PW6B95	10.76	0.67
MN12-SX	10.79	0.68
CAMB3LYP-D4	10.94	0.76
mPWB1K-D3(BJ)	11.10	0.70
mPWB1K	11.13	0.72
mPW1B95	11.60	0.72
SOGGA11-X	12.11	0.73
WB97X-D3(0)	12.67	0.72
WB97X-D4	13.33	0.75
M05-2X-D3(0)	14.28	0.82
PBE0	16.78	0.94
MN12-SX-D3(BJ)	17.34	0.92
M06-D3(0)	17.40	0.96
M06-2X-D3(0)	17.92	0.95
M08-HX-D3(0)	18.08	0.93
M08-SO-D3(0)	18.69	0.97
mPW1PW	19.60	1.05
TPSSH	20.12	1.17
B97	20.36	1.12
B3LYP	22.20	1.28
CAMB3LYP	22.20	1.28
(meta-)GGAs		
SCAN-D4	9.27	0.66
SCAN-D3(BJ)	9.47	0.67

Continued on next page

Functional	WTMAD-2	MAD
revTPSS-D3(BJ)	9.78	0.82
revTPSS-D4	10.08	0.86
REVPBE-D3(BJ)	10.24	0.69
SCAN	10.24	0.69
REVPBE-D4	10.61	0.75
BLYP-D3(BJ)	11.35	0.89
TPSS-D4	11.42	0.85
TPSS-D3(BJ)	11.76	0.86
PBE-D4	12.07	0.80
BLYP-D4	12.24	0.93
PBE-D3(BJ)	12.72	0.82
revTPSS	18.31	1.20
PBE	19.88	1.12
TPSS	21.60	1.28
BLYP	27.16	1.62
REVPBE	28.97	1.58

Table S8: WTMAD-2 and MAD values of all benchmarked DFAs for easy isomerization reactions exlcuding PCONF and SCONF separated for different rungs on Jacobs ladder in descending order. All energies in kcal/mol

Functional	WTMAD-2	MAD
Double hybrids		
revDSD-PBEP86-D4	4.71	0.33
revDOD-PBEP86-D4	4.85	0.34
revDOD-PBEP86-D3(BJ)	4.99	0.35
revDSD-PBEP86-D3(BJ)	4.99	0.35
B2K-PLYP	5.20	0.37
mPW2K-PLYP	5.30	0.39
mPW2NC-PLYP	5.32	0.42
SOS1-PBE-QIDH	5.36	0.43
SOS1-PBE-QIDH-D3(BJ)	5.46	0.44
DSD-PBEP86	5.48	0.38
B2GP-PLYP	5.64	0.42
mPW2-PLYP-D4	5.74	0.47
DH-BLYP	5.78	0.44
B2GP-PLYP-D3(BJ)	6.23	0.46
B2T-PLYP	6.40	0.49
B2-PLYP-D3(BJ)	6.44	0.51
B2NC-PLYP	6.44	0.49
revDSD-BLYP-D4	6.44	0.46
PBE-QIDH	6.46	0.51
revDOD-BLYP-D4	6.50	0.47

Continued on next page

Functional	WTMAD-2	MAD
revDSD-PBE-D4	6.50	0.45
revDSD-PBE-D3(BJ)	6.56	0.45
B2π-PLYP	6.57	0.48
B2T-PLYP-D3(BJ)	6.67	0.50
revDOD-PBE-D4	6.67	0.46
revDOD-PBE-D3(BJ)	6.69	0.46
PBE0-DH-D4	6.71	0.55
revDSD-BLYP-D3(BJ)	6.79	0.48
B2π-PLYP-D3(BJ)	6.84	0.49
B2-PLYP-D4	6.86	0.54
DSD-BLYP	6.95	0.50
mPW2-PLYP	7.02	0.55
PBE-QIDH-D3(BJ)	7.15	0.56
revDOD-BLYP-D3(BJ)	7.18	0.50
SDSCAN69	7.19	0.52
B2-PLYP	7.45	0.58
LS1DH	7.48	0.54
DSD-PBE	7.53	0.52
mPW2NC-PLYP-D3(BJ)	7.67	0.57
PBE0-DH-D3(BJ)	7.70	0.60
DS1TPSS	7.71	0.58
PBE0-2	7.86	0.60
B2GP-PLYP-D4	7.92	0.57
mPW2-PLYP-D3(BJ)	8.01	0.60
B2K-PLYP-D3(BJ)	8.11	0.55
PBE0-DH	8.11	0.64
PBE0-2-D4	8.46	0.64
LS1TPSS	8.49	0.62
mPW2K-PLYP-D3(BJ)	8.63	0.59
DH-BLYP-D3(BJ)	8.67	0.61
LS1TPSS-D3(BJ)	9.09	0.66
B2NC-PLYP-D3(BJ)	9.52	0.67
revDSD-SCAN-D4	10.66	0.70
revDOD-SCAN-D4	10.78	0.71
LS1DH-D3(BJ)	10.99	0.76
PBE0-2-D3(BJ)	11.44	0.82
DOD-SCAN-D3(BJ)	11.46	0.75
DS1TPSS-D3(BJ)	11.63	0.81
hybrids		
M06-2X	5.74	0.45
M05-2X	5.79	0.45
B97-D4	5.80	0.44
SOGGA11-X-D3(BJ)	5.91	0.46
WB97X	5.99	0.46

Continued on next page

Functional	WTMAD-2	MAD
PW6B95-D4	6.25	0.49
PW6B95-D3(0)	6.51	0.51
mPW1PW-D3(BJ)	6.62	0.52
PWB6K-D3(0)	6.88	0.54
M06	6.96	0.54
M08-SO	7.02	0.48
PBE0-D3(BJ)	7.03	0.56
PBE0-D4	7.07	0.56
B3LYP-D3(BJ)	7.08	0.62
CAMB3LYP-D3(BJ)	7.08	0.62
M08-HX	7.31	0.50
mPW1B95-D3(BJ)	7.32	0.56
TPSSH-D3(BJ)	7.56	0.66
B97-D3(0)	7.57	0.58
B3LYP-D4	7.58	0.65
mPWB1K-D4	7.61	0.60
M06-D4	7.85	0.60
PWB6K	8.33	0.64
TPSSH-D4	8.37	0.72
PW6B95	8.52	0.65
SOGGA11-X	8.75	0.67
CAMB3LYP-D4	8.86	0.75
mPW1B95	9.01	0.69
mPWB1K	9.30	0.71
mPWB1K-D3(BJ)	9.33	0.69
WB97X-D3(0)	9.63	0.66
MN12-SX	9.66	0.68
WB97X-D4	10.05	0.67
M05-2X-D3(0)	10.18	0.73
PBE0	11.25	0.84
M06-2X-D3(0)	11.45	0.80
M08-HX-D3(0)	11.94	0.78
MN12-SX-D3(BJ)	12.12	0.82
M06-D3(0)	12.33	0.86
M08-SO-D3(0)	12.50	0.81
mPW1PW	12.83	0.90
TPSSH	13.21	1.03
B97	13.72	0.96
B3LYP	15.14	1.14
CAMB3LYP	15.14	1.14
(meta-)GGAs		
revTPSS-D3(BJ)	7.95	0.79
revTPSS-D4	8.76	0.84
SCAN-D3(BJ)	8.77	0.67

Continued on next page

Functional	WTMAD-2	MAD
SCAN-D4	8.78	0.67
REVPBE-D3(BJ)	8.93	0.69
SCAN	9.07	0.68
REVPBE-D4	9.45	0.74
TPSS-D3(BJ)	9.58	0.81
TPSS-D4	9.84	0.82
BLYP-D3(BJ)	10.28	0.90
PBE-D4	10.30	0.76
PBE-D3(BJ)	10.40	0.77
BLYP-D4	11.38	0.95
revTPSS	12.43	1.09
PBE	13.35	0.99
TPSS	14.12	1.13
BLYP	18.78	1.48
REVPBE	19.48	1.37

Table S9: WTMAD-2 and MAD values of all benchmarked DFAs for difficult Isomerization reactions separated for different rungs on Jacobs ladder in descending order. All energies in kcal/mol

Functional	WTMAD-2	MAD
Double hybrids		
revDOD-PBE-D4	3.57	1.03
revDSD-PBE-D4	3.60	0.98
revDOD-PBEP86-D4	3.72	1.19
revDSD-PBEP86-D4	3.89	1.26
revDOD-PBE-D3(BJ)	3.90	1.20
revDSD-PBEP86-D3(BJ)	3.97	1.29
revDSD-PBE-D3(BJ)	4.04	1.18
revDOD-SCAN-D4	4.11	1.12
revDSD-SCAN-D4	4.13	1.13
DOD-SCAN-D3(BJ)	4.21	1.15
revDOD-BLYP-D3(BJ)	4.26	1.87
revDOD-PBEP86-D3(BJ)	4.32	1.43
DSD-PBE	4.37	1.17
revDOD-BLYP-D4	4.38	1.85
SOS1-PBE-QIDH-D3(BJ)	4.65	1.46
DSD-PBEP86	4.68	1.38
revDSD-BLYP-D4	4.70	1.83
revDSD-BLYP-D3(BJ)	4.90	1.97
SDSCAN69	4.99	1.35
SOS1-PBE-QIDH	5.00	1.46
LS1TPSS-D3(BJ)	5.47	1.77

Continued on next page

Functional	WTMAD-2	MAD
LS1TPSS	5.80	1.79
LS1DH	6.02	1.75
PBE0-2	6.02	1.82
LS1DH-D3(BJ)	6.14	1.89
B2K-PLYP-D3(BJ)	6.23	2.32
mPW2K-PLYP-D3(BJ)	6.26	2.41
DSD-BLYP	6.30	2.33
PBE0-2-D3(BJ)	6.39	2.04
mPW2K-PLYP	6.62	2.48
B2K-PLYP	6.70	2.43
DS1TPSS	6.86	1.84
B2GP-PLYP-D4	6.87	2.43
PBE-QIDH	6.89	1.94
B2GP-PLYP-D3(BJ)	6.91	2.52
mPW2NC-PLYP-D3(BJ)	6.93	2.64
PBE-QIDH-D3(BJ)	6.93	1.97
DS1TPSS-D3(BJ)	6.99	1.90
B2π-PLYP-D3(BJ)	7.11	2.62
DH-BLYP-D3(BJ)	7.22	2.50
B2T-PLYP-D3(BJ)	7.29	2.68
B2NC-PLYP-D3(BJ)	7.30	2.44
B2NC-PLYP	7.47	2.53
mPW2NC-PLYP	7.67	2.87
mPW2-PLYP-D3(BJ)	7.68	3.00
PBE0-DH-D3(BJ)	7.80	2.14
DH-BLYP	7.83	2.70
B2GP-PLYP	7.91	2.80
B2-PLYP-D4	8.12	2.82
PBE0-DH-D4	8.16	2.16
B2-PLYP-D3(BJ)	8.25	2.97
mPW2-PLYP-D4	8.25	3.08
B2π-PLYP	8.61	3.00
PBE0-DH	8.79	2.18
B2T-PLYP	8.82	3.10
mPW2-PLYP	9.58	3.50
B2-PLYP	10.04	3.46
PBE0-2-D4	11.84	5.11
hybrids		
M05-2X	5.03	2.19
WB97X-D4	5.28	1.56
WB97X	5.63	1.62
M05-2X-D3(0)	5.67	2.29
M08-SO	5.74	1.91
WB97X-D3(0)	5.85	1.70

Continued on next page

Functional	WTMAD-2	MAD
M08-HX-D3(0)	6.01	1.88
M06-2X	6.20	2.34
M08-HX	6.26	1.91
M08-SO-D3(0)	6.26	1.98
M06-2X-D3(0)	6.40	2.34
SOGGA11-X-D3(BJ)	7.54	1.96
PWB6K-D3(0)	7.84	2.05
mPWB1K-D3(BJ)	8.46	2.45
mPWB1K-D4	8.69	2.41
PWB6K	8.70	2.20
B97-D4	9.04	2.21
mPWB1K	9.55	2.51
PW6B95-D3(0)	9.60	2.34
SOGGA11-X	9.68	2.35
PW6B95-D4	9.77	2.32
PBE0-D3(BJ)	9.77	2.15
M06-D3(0)	9.95	2.90
mPW1B95-D3(BJ)	9.96	2.48
PBE0-D4	10.12	2.29
mPW1PW-D3(BJ)	10.18	2.24
M06-D4	10.63	2.95
M06	10.78	3.00
MN12-SX-D3(BJ)	10.79	2.43
PW6B95	10.88	2.57
B97-D3(0)	11.13	3.13
mPW1B95	11.14	2.73
B3LYP-D4	11.69	3.87
PBE0	11.80	2.56
TPSSH-D4	12.10	2.51
B3LYP-D3(BJ)	12.15	4.25
MN12-SX	12.15	3.03
CAMB3LYP-D3(BJ)	12.16	4.26
TPSSH-D3(BJ)	12.33	2.70
mPW1PW	12.72	2.86
B97	13.33	3.64
CAMB3LYP-D4	13.48	4.49
TPSSH	15.53	3.64
B3LYP	16.13	5.34
CAMB3LYP	16.15	5.34
(meta-)GGAs		
SCAN-D3(BJ)	13.00	2.67
revTPSS-D3(BJ)	13.17	2.98
SCAN-D4	13.18	2.69
revTPSS-D4	13.36	3.09

Continued on next page

Functional	WTMAD-2	MAD
REVPBE-D3(BJ)	13.41	3.24
PBE-D3(BJ)	13.42	2.73
PBE-D4	13.61	2.77
SCAN	13.71	2.83
TPSS-D4	13.92	2.98
TPSS-D3(BJ)	14.33	3.29
REVPBE-D4	14.47	3.69
BLYP-D4	14.83	4.79
revTPSS	15.18	3.53
BLYP-D3(BJ)	15.60	5.35
PBE	16.07	3.55
TPSS	17.64	4.29
REVPBE	17.83	4.39
BLYP	19.91	6.45

Table S10: WTMAD-2 and MAD values of all benchmarked DFAs for barrier heights separated for different rungs on Jacobs ladder in descending order. All energies in kcal/mol

Functional	WTMAD-2	MAD
Double hybrids		
revDOD-PBEP86-D3(BJ)	4.16	1.36
revDSD-PBEP86-D3(BJ)	4.17	1.37
revDOD-PBE-D3(BJ)	4.18	1.45
revDOD-PBEP86-D4	4.23	1.39
revDSD-PBEP86-D4	4.28	1.41
revDOD-PBE-D4	4.35	1.52
revDSD-PBE-D3(BJ)	4.37	1.51
revDSD-PBE-D4	4.46	1.56
DSD-PBEP86	4.51	1.51
revDOD-BLYP-D3(BJ)	4.53	1.49
DSD-PBE	4.62	1.65
B2K-PLYP	4.69	1.57
revDSD-BLYP-D3(BJ)	4.75	1.57
revDOD-BLYP-D4	4.84	1.61
revDSD-BLYP-D4	4.88	1.62
mPW2K-PLYP	4.90	1.63
B2K-PLYP-D3(BJ)	4.98	1.67
LS1DH	5.13	1.91
DOD-SCAN-D3(BJ)	5.24	1.77
mPW2K-PLYP-D3(BJ)	5.24	1.74
SDSCAN69	5.36	1.89
revDSD-SCAN-D4	5.43	1.83
revDOD-SCAN-D4	5.43	1.84

Continued on next page

Functional	WTMAD-2	MAD
B2GP-PLYP	5.55	1.87
B2GP-PLYP-D4	5.56	1.88
DSD-BLYP	5.65	1.89
B2GP-PLYP-D3(BJ)	5.75	1.92
LS1DH-D3(BJ)	5.80	2.17
PBE0-2	5.92	2.28
B2π-PLYP	5.93	1.97
B2π-PLYP-D3(BJ)	5.93	1.95
PBE0-DH	5.94	2.03
mPW2NC-PLYP	6.11	2.10
LS1TPSS	6.14	2.36
DS1TPSS	6.20	2.28
PBE0-DH-D4	6.29	2.10
B2T-PLYP	6.30	2.15
LS1TPSS-D3(BJ)	6.34	2.44
mPW2NC-PLYP-D3(BJ)	6.40	2.21
SOS1-PBE-QIDH	6.42	2.14
mPW2-PLYP-D4	6.47	2.20
B2T-PLYP-D3(BJ)	6.48	2.19
DH-BLYP	6.67	2.29
PBE0-2-D3(BJ)	6.81	2.62
SOS1-PBE-QIDH-D3(BJ)	6.87	2.28
mPW2-PLYP	7.00	2.45
DS1TPSS-D3(BJ)	7.00	2.55
DH-BLYP-D3(BJ)	7.20	2.46
mPW2-PLYP-D3(BJ)	7.24	2.49
PBE0-DH-D3(BJ)	7.39	2.55
B2NC-PLYP	7.51	2.61
B2-PLYP-D4	7.68	2.66
B2NC-PLYP-D3(BJ)	7.82	2.73
B2-PLYP	7.89	2.74
B2-PLYP-D3(BJ)	8.04	2.76
PBE-QIDH	8.26	2.67
PBE-QIDH-D3(BJ)	9.09	2.88
PBE0-2-D4	11.78	3.68
hybrids		
SOGGA11-X-D3(BJ)	5.57	1.66
M08-SO	5.63	1.74
M08-HX	5.66	1.76
M06-2X	5.67	1.73
M06-2X-D3(0)	5.77	1.80
PWB6K-D3(0)	6.09	1.86
M08-HX-D3(0)	6.10	1.94
M08-SO-D3(0)	6.12	1.93

Continued on next page

Functional	WTMAD-2	MAD
SOGGA11-X	6.17	1.91
M05-2X	6.21	2.11
PWB6K	6.37	1.96
mPWB1K	6.52	2.17
mPWB1K-D4	6.60	2.18
M05-2X-D3(0)	7.02	2.41
MN12-SX-D3(BJ)	7.06	2.02
PW6B95	7.12	2.19
PW6B95-D3(0)	7.22	2.23
M06-D4	7.28	2.32
mPWB1K-D3(BJ)	7.31	2.47
mPW1B95	7.38	2.42
PW6B95-D4	7.44	2.33
M06-D3(0)	7.68	2.44
M06	7.71	2.45
MN12-SX	7.73	2.30
WB97X-D4	7.86	2.31
mPW1B95-D3(BJ)	7.94	2.62
mPW1PW-D3(BJ)	8.19	2.62
mPW1PW	8.22	2.64
WB97X-D3(0)	8.23	2.79
WB97X	8.38	2.85
B97-D3(0)	9.79	3.36
B97-D4	9.91	3.35
B97	10.23	3.52
PBE0-D4	11.01	3.45
CAMB3LYP-D4	11.37	3.94
CAMB3LYP-D3(BJ)	11.82	4.05
B3LYP-D3(BJ)	11.83	4.05
TPSSH	12.19	4.23
TPSSH-D4	12.61	4.32
CAMB3LYP	12.85	4.46
B3LYP	12.86	4.46
B3LYP-D4	12.87	4.25
TPSSH-D3(BJ)	13.09	4.50
PBE0	13.87	4.57
PBE0-D3(BJ)	14.92	4.93
(meta-)GGAs		
REVPBE	13.53	4.62
SCAN	14.92	5.03
SCAN-D3(BJ)	15.34	5.18
TPSS	15.48	5.55
TPSS-D4	16.20	5.69
revTPSS-D4	16.31	5.64

Continued on next page

Functional	WTMAD-2	MAD
revTPSS	16.44	5.72
PBE	16.54	5.95
TPSS-D3(BJ)	16.73	5.93
REVPBE-D3(BJ)	17.57	5.95
revTPSS-D3(BJ)	17.66	6.10
PBE-D3(BJ)	17.67	6.31
SCAN-D4	18.19	5.59
REVPBE-D4	19.20	6.45
PBE-D4	19.34	6.75
BLYP-D3(BJ)	19.71	7.06
BLYP	19.85	7.19
BLYP-D4	20.57	7.23

Table S11: WTMAD-2 and MAD values of all benchmarked DFAs for barrier heights without transition metal containing systems separated for different rungs on Jacobs ladder in descending order. All energies in kcal/mol

Functional	WTMAD-2	MAD
Double hybrids		
revDOD-PBEP86-D3(BJ)	3.33	1.29
revDSD-PBEP86-D3(BJ)	3.34	1.30
revDOD-PBE-D3(BJ)	3.40	1.41
revDOD-PBEP86-D4	3.43	1.33
revDSD-PBEP86-D4	3.49	1.35
revDOD-BLYP-D3(BJ)	3.61	1.41
revDOD-PBE-D4	3.61	1.50
revDSD-PBE-D3(BJ)	3.61	1.48
revDSD-PBE-D4	3.72	1.54
DSD-PBEP86	3.75	1.47
SOS1-PBE-QIDH	3.80	1.71
revDSD-BLYP-D3(BJ)	3.86	1.50
DSD-PBE	3.92	1.64
revDOD-BLYP-D4	3.97	1.55
revDSD-BLYP-D4	4.03	1.57
B2K-PLYP	4.09	1.56
SOS1-PBE-QIDH-D3(BJ)	4.10	1.83
mPW2K-PLYP	4.17	1.60
B2K-PLYP-D3(BJ)	4.32	1.66
DOD-SCAN-D3(BJ)	4.32	1.72
mPW2K-PLYP-D3(BJ)	4.46	1.71
revDSD-SCAN-D4	4.49	1.79
revDOD-SCAN-D4	4.52	1.80
LS1DH	4.57	1.96

Continued on next page

Functional	WTMAD-2	MAD
PBE-QIDH	4.59	2.02
SDSCAN69	4.62	1.89
PBE0-DH	4.71	1.94
DSD-BLYP	4.75	1.85
B2GP-PLYP-D4	4.79	1.87
B2GP-PLYP	4.79	1.86
B2GP-PLYP-D3(BJ)	4.84	1.88
PBE-QIDH-D3(BJ)	4.92	2.14
PBE0-DH-D4	4.94	1.98
B2π-PLYP-D3(BJ)	5.02	1.92
B2π-PLYP	5.16	1.97
mPW2NC-PLYP	5.26	2.10
LS1DH-D3(BJ)	5.39	2.28
PBE0-2	5.42	2.39
B2T-PLYP-D3(BJ)	5.47	2.16
B2T-PLYP	5.49	2.16
mPW2-PLYP-D4	5.50	2.17
LS1TPSS	5.57	2.45
mPW2NC-PLYP-D3(BJ)	5.58	2.23
DS1TPSS	5.63	2.36
DH-BLYP	5.80	2.30
LS1TPSS-D3(BJ)	5.82	2.55
DH-BLYP-D3(BJ)	6.18	2.45
PBE0-DH-D3(BJ)	6.29	2.53
mPW2-PLYP	6.31	2.51
mPW2-PLYP-D3(BJ)	6.32	2.51
DS1TPSS-D3(BJ)	6.32	2.63
PBE0-2-D3(BJ)	6.49	2.79
B2-PLYP-D4	6.61	2.66
B2NC-PLYP	6.63	2.64
B2-PLYP-D3(BJ)	6.86	2.76
B2-PLYP	6.94	2.78
B2NC-PLYP-D3(BJ)	6.99	2.79
PBE0-2-D4	12.51	4.07
hybrids		
M08-HX	3.38	1.40
M06-2X	3.64	1.41
M08-SO	3.70	1.44
SOGGA11-X-D3(BJ)	3.78	1.41
M06-2X-D3(0)	3.92	1.52
MN12-SX-D3(BJ)	4.03	1.56
M08-HX-D3(0)	4.05	1.63
M08-SO-D3(0)	4.27	1.68
SOGGA11-X	4.41	1.66

Continued on next page

Functional	WTMAD-2	MAD
PWB6K-D3(0)	4.43	1.63
WB97X-D4	4.47	1.69
PWB6K	4.65	1.73
M05-2X	4.71	1.95
mPWB1K-D4	4.88	1.98
MN12-SX	4.92	1.92
mPWB1K	5.02	2.02
M05-2X-D3(0)	5.72	2.31
M06-D4	5.78	2.19
PW6B95	5.90	2.09
M06-D3(0)	5.91	2.28
mPWB1K-D3(BJ)	6.00	2.38
M06	6.00	2.30
PW6B95-D3(0)	6.07	2.15
mPW1B95	6.36	2.40
PW6B95-D4	6.38	2.28
WB97X-D3(0)	6.74	2.69
WB97X	6.76	2.72
mPW1PW-D3(BJ)	6.86	2.55
mPW1B95-D3(BJ)	6.98	2.62
mPW1PW	7.02	2.59
PBE0-D4	8.22	3.09
B97-D3(0)	8.47	3.38
B97-D4	8.58	3.37
B97	9.02	3.56
B3LYP-D4	10.10	3.98
CAMB3LYP-D4	10.14	4.02
CAMB3LYP-D3(BJ)	10.35	4.10
B3LYP-D3(BJ)	10.35	4.10
TPSSH-D4	11.20	4.40
TPSSH	11.26	4.41
CAMB3LYP	11.71	4.61
B3LYP	11.71	4.61
TPSSH-D3(BJ)	11.85	4.64
PBE0	13.74	4.88
PBE0-D3(BJ)	14.86	5.30
(meta-)GGAs		
REVPBE	11.87	4.69
SCAN	13.06	5.08
SCAN-D3(BJ)	13.46	5.23
TPSS-D4	14.35	5.81
TPSS	14.53	5.85
revTPSS-D4	14.68	5.78
TPSS-D3(BJ)	15.36	6.18

Continued on next page

Functional	WTMAD-2	MAD
PBE	15.39	6.25
revTPSS	15.44	6.02
REVPBE-D3(BJ)	15.78	6.11
PBE-D3(BJ)	16.28	6.58
revTPSS-D3(BJ)	16.39	6.37
PBE-D4	16.50	6.71
SCAN-D4	16.61	5.67
REVPBE-D4	17.36	6.60
BLYP-D4	17.46	7.18
BLYP-D3(BJ)	17.62	7.28
BLYP	18.31	7.53

Table S12: WTMAD-2 and MAD values of all benchmarked DFAs for thermochemistry separated for different rungs on Jacobs ladder in descending order. All energies in kcal/mol

Functional	WTMAD-2	MAD
Double hybrids		
B2π-PLYP-D3(BJ)	2.56	2.58
B2T-PLYP-D3(BJ)	2.62	2.86
B2GP-PLYP-D3(BJ)	2.71	2.96
B2-PLYP-D3(BJ)	2.78	2.63
mPW2-PLYP-D3(BJ)	2.79	2.95
mPW2K-PLYP	2.81	3.26
DH-BLYP	2.83	2.60
mPW2NC-PLYP-D3(BJ)	2.85	3.61
B2K-PLYP	2.86	3.41
revDOD-BLYP-D4	2.96	2.54
revDSD-BLYP-D4	3.02	2.83
revDOD-BLYP-D3(BJ)	3.02	2.67
revDSD-BLYP-D3(BJ)	3.03	2.84
B2GP-PLYP	3.07	3.25
B2NC-PLYP	3.10	2.95
DSD-BLYP	3.11	3.09
B2NC-PLYP-D3(BJ)	3.15	2.94
DH-BLYP-D3(BJ)	3.17	2.60
mPW2-PLYP-D4	3.17	3.27
revDSD-PBEP86-D3(BJ)	3.18	4.20
revDSD-PBEP86-D4	3.18	4.20
revDOD-PBEP86-D3(BJ)	3.20	4.00
LS1DH	3.20	3.50
revDOD-PBEP86-D4	3.22	4.10
mPW2NC-PLYP	3.24	3.89
DSD-PBEP86	3.34	4.22

Continued on next page

Functional	WTMAD-2	MAD
B2π-PLYP	3.37	2.95
B2K-PLYP-D3(BJ)	3.46	3.51
B2T-PLYP	3.48	3.42
revDOD-PBE-D3(BJ)	3.49	3.23
mPW2K-PLYP-D3(BJ)	3.52	3.44
revDOD-PBE-D4	3.53	3.37
revDSD-PBE-D4	3.55	3.50
revDSD-PBE-D3(BJ)	3.56	3.50
B2-PLYP-D4	3.62	2.99
DSD-PBE	3.75	3.30
SDSCAN69	3.80	2.94
DS1TPSS	3.81	2.96
LS1DH-D3(BJ)	3.89	3.90
mPW2-PLYP	4.00	3.66
B2GP-PLYP-D4	4.01	3.48
PBE-QIDH	4.02	4.62
B2-PLYP	4.10	3.37
PBE-QIDH-D3(BJ)	4.28	4.77
SOS1-PBE-QIDH	4.31	4.85
PBE0-DH-D3(BJ)	4.33	4.43
SOS1-PBE-QIDH-D3(BJ)	4.35	4.98
PBE0-DH-D4	4.43	4.57
PBE0-2	4.49	3.98
PBE0-DH	4.51	4.42
revDSD-SCAN-D4	4.76	3.54
DOD-SCAN-D3(BJ)	4.88	3.55
revDOD-SCAN-D4	4.89	3.55
LS1TPSS	5.13	4.17
DS1TPSS-D3(BJ)	5.15	3.62
PBE0-2-D3(BJ)	5.21	4.36
LS1TPSS-D3(BJ)	5.50	4.38
PBE0-2-D4	14.90	7.76
hybrids		
M06-2X-D3(0)	3.31	3.22
M06-2X	3.78	3.42
M08-SO	3.85	4.01
M08-SO-D3(0)	3.92	4.06
M08-HX	4.01	3.72
M08-HX-D3(0)	4.07	3.80
M05-2X	4.09	4.31
M05-2X-D3(0)	4.29	4.48
MN12-SX-D3(BJ)	4.35	4.08
B97-D4	4.58	3.18
PWB6K-D3(0)	4.70	5.84

Continued on next page

Functional	WTMAD-2	MAD
M06-D4	4.88	4.42
SOGGA11-X-D3(BJ)	4.89	4.13
mPW1PW-D3(BJ)	5.22	5.01
MN12-SX	5.29	4.48
B3LYP-D4	5.32	4.53
PBE0-D3(BJ)	5.34	4.43
M06	5.39	4.58
M06-D3(0)	5.46	4.68
TPSSH-D4	5.52	4.41
PWB6K	5.69	6.11
TPSSH-D3(BJ)	5.71	4.51
CAMB3LYP-D3(BJ)	5.75	4.77
B3LYP-D3(BJ)	5.77	4.78
PBE0-D4	5.83	4.58
mPWB1K-D3(BJ)	5.92	6.18
PW6B95-D3(0)	5.95	4.30
mPWB1K-D4	6.00	5.98
B97-D3(0)	6.16	4.08
SOGGA11-X	6.19	4.78
mPW1B95-D3(BJ)	6.36	4.98
PBE0	6.39	4.84
WB97X-D3(0)	6.44	5.03
mPWB1K	6.50	6.34
PW6B95-D4	6.57	4.55
PW6B95	6.68	4.57
CAMB3LYP-D4	6.69	4.80
mPW1PW	6.73	5.84
mPW1B95	6.99	5.15
WB97X	7.04	5.13
WB97X-D4	7.55	5.05
B97	7.62	4.73
TPSSH	7.64	5.33
CAMB3LYP	9.08	6.56
B3LYP	9.11	6.58
(meta-)GGAs		
SCAN-D3(BJ)	5.27	4.22
SCAN	5.52	4.28
SCAN-D4	5.61	4.46
revTPSS-D4	5.96	4.72
TPSS-D4	5.98	4.58
REVPBE-D4	6.09	5.12
REVPBE-D3(BJ)	6.13	5.19
revTPSS-D3(BJ)	6.47	4.99
TPSS-D3(BJ)	6.78	5.51

Continued on next page

Functional	WTMAD-2	MAD
PBE-D4	7.12	7.65
BLYP-D4	7.32	6.50
PBE-D3(BJ)	7.59	7.88
BLYP-D3(BJ)	7.86	6.73
revTPSS	7.89	5.46
TPSS	8.82	6.23
PBE	8.95	8.25
REVPBE	9.18	6.45
BLYP	11.83	8.58

Table S13: WTMAD-2 and MAD values of all benchmarked DFAs for transition metals separated for different rungs on Jacobs ladder in descending order. All energies in kcal/mol

Functional	WTMAD-2	MAD
Double hybrids		
revDOD-PBEP86-D4	7.14	2.21
revDSD-PBEP86-D4	7.31	2.32
B2K-PLYP	7.37	2.53
revDOD-PBEP86-D3(BJ)	7.69	2.53
revDSD-PBEP86-D3(BJ)	7.87	2.64
mPW2K-PLYP	7.97	2.65
revDOD-PBE-D4	8.05	2.82
LS1DH	8.16	2.96
revDSD-PBE-D4	8.20	2.89
revDOD-BLYP-D4	8.23	2.64
B2π-PLYP	8.24	2.45
B2GP-PLYP	8.26	2.59
revDSD-BLYP-D4	8.62	2.90
revDOD-PBE-D3(BJ)	8.72	3.26
DSD-PBEP86	8.73	3.19
LS1DH-D3(BJ)	8.81	3.42
B2T-PLYP	8.84	2.66
PBE0-2-D4	8.90	3.41
mPW2-PLYP	8.91	2.71
revDSD-PBE-D3(BJ)	8.95	3.38
revDOD-BLYP-D3(BJ)	9.10	3.19
mPW2-PLYP-D4	9.24	2.67
PBE0-DH	9.42	2.56
mPW2NC-PLYP	9.45	3.06
revDSD-BLYP-D3(BJ)	9.50	3.43
DSD-PBE	9.55	3.77
PBE0-2	9.63	3.75
B2K-PLYP-D3(BJ)	9.69	3.84

Continued on next page

Functional	WTMAD-2	MAD
B2GP-PLYP-D4	9.71	3.51
SDSCAN69	9.82	3.68
B2π-PLYP-D3(BJ)	9.86	3.26
DH-BLYP	10.06	3.27
mPW2NC-PLYP-D3(BJ)	10.24	3.52
mPW2K-PLYP-D3(BJ)	10.46	4.07
B2GP-PLYP-D3(BJ)	10.47	3.74
mPW2-PLYP-D3(BJ)	10.58	3.35
PBE0-2-D3(BJ)	10.58	4.39
B2-PLYP	10.58	3.11
B2T-PLYP-D3(BJ)	10.67	3.48
DS1TPSS	10.69	4.26
PBE0-DH-D4	10.72	3.09
LS1TPSS	10.89	4.41
revDOD-SCAN-D4	10.94	4.10
B2-PLYP-D4	10.99	3.29
revDSD-SCAN-D4	11.00	4.12
DSD-BLYP	11.00	4.12
LS1TPSS-D3(BJ)	11.02	4.50
DOD-SCAN-D3(BJ)	11.13	4.29
B2NC-PLYP	11.32	3.83
B2NC-PLYP-D3(BJ)	11.48	3.91
PBE0-DH-D3(BJ)	11.70	3.79
B2-PLYP-D3(BJ)	11.88	3.56
DH-BLYP-D3(BJ)	12.64	4.53
DS1TPSS-D3(BJ)	13.79	5.86
SOS1-PBE-QIDH	13.95	3.48
SOS1-PBE-QIDH-D3(BJ)	17.32	5.29
PBE-QIDH	20.03	5.43
PBE-QIDH-D3(BJ)	23.39	6.63
hybrids		
mPW1B95-D3(BJ)	11.11	3.45
mPW1PW-D3(BJ)	11.23	2.85
PW6B95-D4	11.27	3.53
PW6B95-D3(0)	11.43	3.54
PWB6K-D3(0)	11.83	3.45
M06-D4	12.20	3.49
mPW1B95	12.27	4.26
PWB6K	12.36	3.63
mPWB1K-D3(BJ)	12.44	4.00
M08-SO-D3(0)	12.80	3.81
mPW1PW	12.82	4.09
mPWB1K-D4	12.83	3.88
M08-SO	12.94	3.91

Continued on next page

Functional	WTMAD-2	MAD
PBE0-D3(BJ)	12.96	3.90
M06-D3(0)	13.07	3.49
M05-2X-D3(0)	13.09	4.56
mPWB1K	13.19	4.45
SOGGA11-X-D3(BJ)	13.20	4.26
PW6B95	13.25	4.64
M05-2X	13.38	4.68
M06	13.55	3.90
PBE0	13.64	4.58
WB97X-D3(0)	13.76	4.33
B97-D4	14.10	4.18
M08-HX-D3(0)	14.12	4.37
M06-2X-D3(0)	14.43	4.97
M08-HX	15.08	4.78
B97-D3(0)	15.16	4.93
WB97X	15.35	5.13
TPSSH	15.71	5.14
SOGGA11-X	15.83	5.84
CAMB3LYP-D4	16.00	5.17
M06-2X	16.05	5.77
B3LYP-D3(BJ)	16.23	4.77
CAMB3LYP-D3(BJ)	16.23	4.77
TPSSH-D3(BJ)	16.26	4.77
TPSSH-D4	16.60	4.89
MN12-SX	16.83	4.33
MN12-SX-D3(BJ)	17.14	4.39
B97	17.26	6.31
PBE0-D4	18.25	4.59
WB97X-D4	18.63	4.93
B3LYP-D4	19.68	4.97
B3LYP	19.75	7.25
CAMB3LYP	19.75	7.25
(meta-)GGAs		
TPSS	16.87	4.90
revTPSS	18.02	5.30
PBE	18.27	5.21
TPSS-D3(BJ)	18.30	4.84
PBE-D3(BJ)	18.55	4.70
SCAN	19.20	5.22
SCAN-D4	19.45	4.87
TPSS-D4	19.46	5.07
revTPSS-D3(BJ)	19.60	5.56
SCAN-D3(BJ)	20.19	5.69
revTPSS-D4	20.40	5.97

Continued on next page

Functional	WTMAD-2	MAD
REVPBE-D3(BJ)	21.13	5.74
REVPBE	21.73	7.49
REVPBE-D4	22.42	6.07
BLYP-D3(BJ)	22.50	5.57
PBE-D4	24.47	6.06
BLYP	25.15	8.08
BLYP-D4	26.10	6.34

Table S14: WTMAD-2 and MAD values of all benchmarked DFAs for large systems separated for different rungs on Jacobs ladder in descending order. All energies in kcal/mol

Functional	WTMAD-2	MAD
Double hybrids		
revDOD-PBEP86-D3(BJ)	4.88	1.47
revDOD-PBEP86-D4	5.14	1.64
revDOD-BLYP-D3(BJ)	5.36	1.45
revDOD-BLYP-D4	5.56	1.65
revDOD-PBE-D3(BJ)	5.62	1.68
revDOD-PBE-D4	5.76	1.85
revDOD-SCAN-D4	8.71	2.59
SOS1-PBE-QIDH	9.03	3.14
DOD-SCAN-D3(BJ)	10.10	2.87
SOS1-PBE-QIDH-D3(BJ)	13.34	4.08
hybrids		
PBE0-D4	5.88	1.77
PBE0-D3(BJ)	6.04	1.86
mPW1B95-D3(BJ)	6.18	1.92
mPWB1K-D4	6.39	2.44
SOGGA11-X-D3(BJ)	6.62	2.27
mPWB1K-D3(BJ)	6.64	2.59
M06-2X	7.49	2.82
M08-SO	7.59	2.65
M08-HX	7.84	2.83
PW6B95-D4	8.17	2.47
B97-D4	8.30	2.58
PW6B95-D3(0)	8.38	2.64
M06-D4	8.62	2.53
mPW1PW-D3(BJ)	8.65	2.53
WB97X-D4	8.82	3.72
M05-2X	9.01	3.31
TPSSH-D4	9.02	3.02
PWB6K-D3(0)	9.26	3.48
M08-HX-D3(0)	9.44	3.19

Continued on next page

Functional	WTMAD-2	MAD
M05-2X-D3(0)	9.52	3.34
TPSSH-D3(BJ)	9.56	3.14
B3LYP-D4	9.81	3.07
CAMB3LYP-D3(BJ)	10.08	3.18
WB97X-D3(0)	10.22	3.97
M08-SO-D3(0)	10.30	3.26
M06	10.57	3.26
MN12-SX-D3(BJ)	10.62	2.93
M06-2X-D3(0)	10.69	3.39
MN12-SX	10.92	3.37
B97-D3(0)	11.07	3.48
WB97X	11.51	4.40
M06-D3(0)	12.52	3.22
mPWB1K	12.68	4.01
B3LYP-D3(BJ)	13.06	4.35
mPW1B95	13.09	3.66
CAMB3LYP-D4	13.49	4.13
PWB6K	13.56	4.53
PW6B95	15.10	4.35
PBE0	15.21	4.17
SOGGA11-X	15.83	4.60
mPW1PW	18.64	5.04
B97	20.49	5.77
TPSSH	22.23	6.36
B3LYP	24.25	6.86
CAMB3LYP	24.38	6.91
(meta-)GGAs		
REVPBE-D4	10.17	3.89
PBE-D4	10.69	4.03
REVPBE-D3(BJ)	10.80	3.98
PBE-D3(BJ)	11.02	4.05
revTPSS-D4	11.02	4.18
SCAN-D3(BJ)	12.15	3.97
SCAN-D4	12.24	4.03
revTPSS-D3(BJ)	12.55	4.50
SCAN	15.05	4.78
BLYP-D4	15.07	5.54
BLYP-D3(BJ)	15.49	5.52
TPSS-D4	17.51	5.89
TPSS-D3(BJ)	18.18	6.00
PBE	20.43	6.47
revTPSS	22.40	7.00
REVPBE	28.53	8.61
TPSS	30.28	9.10

Continued on next page

Functional	WTMAD-2	MAD
BLYP	31.21	9.63

S5 Performance of all functionals for all Test Sets

S5.1 D3-Dispersion-Corrected Double Hybrids

Table S15: Statistical analysis for B2-PLYP-D3(BJ) for all testset in our databsase. The numbers given (all in kcal/mol) are average reaction energy ($|\overline{\Delta E}|$), mean deviation (MD), mean absolute deviation (MAD), MAD normalized with respect to $|\overline{\Delta E}|$ (NMAD), root-mean-square deviation (RMSD), deviation span (Δ_{err}), maximum (max) and minimum deviation (min).

Test set	$\lvert\overline{\Delta E}\rvert$	MD	MAD	NMAD	RMSD	Δ_{err}	max	min
FH51	31.01	1.24	1.24	0.04	1.70	4.88	4.87	0.01
YBDE18	49.28	-2.00	2.91	0.06	3.13	6.45	5.13	1.32
AL2X6	35.88	-1.26	1.26	0.04	1.32	2.40	1.67	0.73
DARC	32.47	3.30	3.30	0.10	3.35	6.27	3.98	2.29
NBPRC	27.71	0.72	2.02	0.07	2.50	5.54	5.33	0.21
HEAVYSB9	58.02	-1.23	1.72	0.03	2.32	5.04	5.01	0.03
BSR36	16.20	-0.08	0.32	0.02	0.47	1.59	1.57	0.02
RSE43	7.60	-0.36	0.47	0.06	0.62	1.75	1.68	0.07
W4-11	306.91	-2.60	3.01	0.01	3.79	13.33	13.31	0.02
G21EA	33.62	-5.65	5.80	0.17	6.11	12.18	10.32	1.86
G21IP	257.61	-1.17	2.61	0.01	3.04	7.75	7.70	0.05
DIPCS10	654.26	-3.31	3.82	0.01	4.42	10.87	9.01	1.86
PA26	189.05	1.92	2.18	0.01	2.97	7.46	7.45	0.01
SIE4x4	33.72	9.48	9.48	0.28	10.98	21.79	21.42	0.37
ALKBDE10	100.69	0.78	3.71	0.04	4.70	8.09	7.95	0.14
RC21	35.70	-0.20	1.06	0.03	1.33	3.32	3.21	0.11
ALK8	62.60	1.04	1.59	0.03	2.68	6.94	6.89	0.05
DC13	54.98	2.06	7.25	0.13	9.78	20.79	20.58	0.21
G2RC	51.26	-0.04	1.87	0.04	2.32	6.52	6.17	0.35
BH76RC	21.39	-0.38	1.01	0.05	1.40	4.52	4.51	0.01
MOR23	35.57	-3.80	4.97	0.14	6.57	16.05	15.81	0.24
WCPT18	34.99	2.46	2.46	0.07	3.04	5.50	5.48	0.02
BHROT27	6.37	0.24	0.24	0.04	0.34	1.05	1.04	0.01
BHPERI	20.87	1.76	1.76	0.08	1.99	4.30	4.28	0.02
BHDIV10	45.33	2.16	2.16	0.05	2.66	4.96	4.86	0.10
INV24	32.85	1.19	1.19	0.04	1.39	2.54	2.51	0.03
CR20	19.31	-6.04	6.04	0.31	6.07	11.78	7.18	4.60

Continued on next page

Test set	$\lvert\overline{\Delta E}\rvert$	MD	MAD	NMAD	RMSD	Δ_{err}	max	min
CRBH20	46.13	-4.90	4.90	0.11	4.93	10.13	6.26	3.87
TMBH17	12.76	-0.14	3.10	0.24	4.27	10.33	10.25	0.08
LTMBH26	9.98	-1.82	2.60	0.26	3.46	10.15	10.14	0.01
BH76	18.61	-2.99	3.21	0.17	3.68	7.98	7.71	0.27
ISO34	14.57	1.19	1.19	0.08	1.83	7.61	7.59	0.02
ICONF	3.27	0.21	0.21	0.06	0.30	0.75	0.75	0.00
ACONF	1.83	0.10	0.10	0.06	0.12	0.25	0.24	0.01
TAUT15	3.05	0.81	0.81	0.27	0.96	1.88	1.87	0.01
Amino20x4	2.44	0.19	0.19	0.08	0.25	0.95	0.95	0.00
PCONF	1.62	0.57	0.57	0.35	0.73	1.64	1.62	0.02
MCONF	4.97	0.69	0.69	0.14	0.76	1.30	1.25	0.05
SCONF	4.60	3.84	3.84	0.83	4.24	8.65	8.47	0.18
PArel	4.63	-0.04	0.73	0.16	1.12	2.76	2.75	0.01
BUT14DIOL	2.80	0.54	0.54	0.19	0.54	0.70	0.68	0.02
EIE22	5.44	0.86	0.86	0.16	0.97	1.93	1.92	0.01
Styrene45	62.64	3.40	4.09	0.07	5.22	14.20	14.20	0.00
ISOMERIZATION20	31.84	-0.41	1.47	0.05	2.04	4.47	4.40	0.07
DIE60	4.71	0.76	0.76	0.16	0.82	1.65	1.35	0.30
IDISP	14.22	2.05	2.05	0.14	2.96	6.19	6.15	0.04
C20C24	30.77	0.40	31.11	1.01	33.19	68.56	49.92	18.64
S66	5.47	-0.40	0.40	0.07	0.45	1.04	1.02	0.02
S10x8	6.59	0.11	0.19	0.03	0.28	1.16	1.16	0.00
X40	3.76	0.25	0.25	0.07	0.27	0.46	0.43	0.03
HEAVY28	1.24	0.28	0.30	0.24	0.37	1.10	1.07	0.03
CHB6	26.79	0.54	0.66	0.02	0.87	1.78	1.72	0.06
AHB21	22.49	-0.63	0.76	0.03	0.99	2.24	2.20	0.04
IL16	109.04	0.67	0.74	0.01	0.87	1.65	1.56	0.09
PNICO23	4.27	0.18	0.19	0.04	0.23	0.51	0.50	0.01
CT20	0.98	0.16	0.17	0.17	0.19	0.38	0.34	0.04
CARBHB12	6.04	1.07	1.07	0.18	1.19	2.27	2.00	0.27
ADIM6	3.36	0.96	0.96	0.28	1.07	1.98	1.68	0.30
3B-69-TRIM	12.30	0.66	0.69	0.06	0.82	2.44	2.43	0.01

Table S16: Statistical analysis for B2GP-PLYP-D3(BJ) for all testset in our databsase. The numbers given (all in kcal/mol) are average reaction energy ($\lvert\overline{\Delta E}\rvert$), mean deviation (MD), mean absolute deviation (MAD), MAD normalized with respect to $\lvert\overline{\Delta E}\rvert$ (NMAD), root-mean-square deviation (RMSD), deviation span (Δ_{err}), maximum (max) and minimum deviation (min).

Test set	$\lvert\overline{\Delta E}\rvert$	MD	MAD	NMAD	RMSD	Δ_{err}	max	min
FH51	31.01	1.06	1.06	0.03	1.44	3.70	3.69	0.01
YBDE18	49.28	-0.84	1.72	0.04	2.01	4.97	4.77	0.20
AL2X6	35.88	0.70	0.73	0.02	0.95	1.97	1.88	0.09

Continued on next page

Test set	$\lvert\overline{\Delta E}\rvert$	MD	MAD	NMAD	RMSD	Δ_{err}	max	min
DARC	32.47	0.36	0.63	0.02	0.76	1.72	1.66	0.06
NBPRC	27.71	0.17	1.64	0.06	1.90	2.96	2.89	0.07
HEAVYSB9	58.02	0.94	2.63	0.05	2.89	5.23	4.72	0.51
BSR36	16.20	0.80	0.90	0.06	1.31	4.58	4.58	0.00
RSE43	7.60	0.24	0.41	0.05	0.73	3.38	3.36	0.02
W4-11	306.91	-4.80	5.03	0.02	5.93	16.96	16.88	0.08
G21EA	33.62	-6.18	6.24	0.19	6.63	12.20	11.44	0.76
G21IP	257.61	-1.29	2.38	0.01	2.89	8.35	8.35	0.00
DIPCS10	654.26	-3.49	3.52	0.01	4.51	9.51	9.48	0.03
PA26	189.05	0.64	1.42	0.01	2.04	5.92	5.91	0.01
SIE4x4	33.72	6.09	6.12	0.18	7.19	14.72	14.52	0.20
ALKBDE10	100.69	-0.12	3.35	0.03	4.22	8.20	8.16	0.04
RC21	35.70	-0.39	1.11	0.03	1.35	3.38	3.38	0.00
ALK8	62.60	3.09	3.17	0.05	4.59	11.56	11.24	0.32
DC13	54.98	0.94	5.05	0.09	7.37	16.26	15.98	0.28
G2RC	51.26	-1.21	2.05	0.04	2.54	7.65	7.33	0.32
BH76RC	21.39	-0.44	0.95	0.04	1.42	4.79	4.74	0.05
MOR23	35.57	-5.59	6.80	0.19	8.10	19.05	18.64	0.41
WCPT18	34.99	2.15	2.15	0.06	2.63	4.98	4.84	0.14
BHROT27	6.37	0.25	0.25	0.04	0.35	1.04	1.04	0.00
BHPERI	20.87	1.62	1.62	0.08	1.86	4.35	4.01	0.34
BHDIV10	45.33	1.63	1.63	0.04	2.03	4.45	4.25	0.20
INV24	32.85	1.53	1.53	0.05	1.79	3.40	3.27	0.13
CR20	19.31	-4.57	4.57	0.24	4.61	9.03	5.71	3.32
CRBH20	46.13	-1.61	1.61	0.03	1.70	3.46	2.91	0.55
TMBH17	12.76	0.32	2.64	0.21	3.63	9.06	8.89	0.17
LTMBH26	9.98	-1.05	1.74	0.17	2.51	8.40	8.28	0.12
BH76	18.61	-1.40	2.00	0.11	2.51	8.04	7.93	0.11
ISO34	14.57	0.83	0.83	0.06	1.45	6.58	6.58	0.00
ICONF	3.27	0.24	0.24	0.07	0.33	0.84	0.78	0.06
ACONF	1.83	0.10	0.10	0.06	0.12	0.21	0.20	0.01
TAUT15	3.05	0.68	0.68	0.22	0.85	1.82	1.79	0.03
Amino20x4	2.44	0.18	0.18	0.07	0.24	0.78	0.78	0.00
PCONF	1.62	0.62	0.62	0.38	0.83	1.90	1.85	0.05
MCONF	4.97	0.78	0.78	0.16	0.86	1.43	1.35	0.08
SCONF	4.60	0.59	0.59	0.13	0.70	1.68	1.64	0.04
PArel	4.63	0.09	0.63	0.14	0.87	1.85	1.85	0.00
BUT14DIOL	2.80	0.58	0.58	0.21	0.59	0.73	0.69	0.04
EIE22	5.44	0.68	0.68	0.13	0.77	1.56	1.56	0.00
Styrene45	62.64	3.31	3.56	0.06	4.41	11.67	11.67	0.00
ISOMERIZATION20	31.84	-0.70	1.52	0.05	1.99	4.03	4.02	0.01
DIE60	4.71	0.64	0.64	0.14	0.72	1.47	1.26	0.21
IDISP	14.22	3.66	3.66	0.26	5.78	13.84	13.35	0.49
C20C24	30.77	0.00	24.91	0.81	26.83	53.93	40.49	13.44

Continued on next page

Test set	$\|\overline{\Delta E}\|$	MD	MAD	NMAD	RMSD	Δ_{err}	max	min
S66	5.47	-0.51	0.51	0.09	0.58	1.38	1.37	0.01
S10x8	6.59	0.16	0.22	0.03	0.32	1.35	1.35	0.00
X40	3.76	0.27	0.27	0.07	0.30	0.57	0.54	0.03
HEAVY28	1.24	0.31	0.35	0.28	0.43	1.21	1.13	0.08
CHB6	26.79	0.08	0.26	0.01	0.31	0.61	0.59	0.02
AHB21	22.49	-0.70	0.83	0.04	1.13	2.63	2.55	0.08
IL16	109.04	0.90	0.90	0.01	1.07	1.90	1.88	0.02
PNICO23	4.27	0.27	0.28	0.07	0.32	0.61	0.59	0.02
CT20	0.98	0.16	0.16	0.17	0.18	0.30	0.30	0.00
CARBHB12	6.04	1.12	1.12	0.19	1.23	2.17	1.89	0.28
ADIM6	3.36	1.06	1.06	0.32	1.19	2.25	1.91	0.34
3B-69-TRIM	12.30	0.82	0.84	0.07	1.00	2.91	2.88	0.03

Table S17: Statistical analysis for B2K-PLYP-D3(BJ) for all testset in our databsase. The numbers given (all in kcal/mol) are average reaction energy ($|\overline{\Delta E}|$), mean deviation (MD), mean absolute deviation (MAD), MAD normalized with respect to $|\overline{\Delta E}|$ (NMAD), root-mean-square deviation (RMSD), deviation span (Δ_{err}), maximum (max) and minimum deviation (min).

Test set	$\|\overline{\Delta E}\|$	MD	MAD	NMAD	RMSD	Δ_{err}	max	min
FH51	31.01	1.54	1.54	0.05	1.98	4.42	4.35	0.07
YBDE18	49.28	0.18	1.74	0.04	2.13	6.16	5.94	0.22
AL2X6	35.88	0.98	1.15	0.03	1.36	2.80	2.36	0.44
DARC	32.47	-2.49	2.49	0.08	2.68	4.36	3.96	0.40
NBPRC	27.71	-0.56	1.62	0.06	1.89	3.39	3.33	0.06
HEAVYSB9	58.02	0.29	1.92	0.03	2.31	5.17	4.83	0.34
BSR36	16.20	2.35	2.39	0.15	3.04	8.97	8.94	0.03
RSE43	7.60	0.68	0.69	0.09	1.20	4.99	4.97	0.02
W4-11	306.91	-6.00	6.32	0.02	7.35	19.01	18.93	0.08
G21EA	33.62	-6.51	6.52	0.19	6.98	12.29	12.13	0.16
G21IP	257.61	-1.41	2.37	0.01	2.95	9.01	8.83	0.18
DIPCS10	654.26	-3.72	3.89	0.01	4.79	9.93	9.83	0.10
PA26	189.05	0.73	1.45	0.01	2.07	5.69	5.69	0.00
SIE4x4	33.72	4.23	4.29	0.13	5.07	11.03	10.54	0.49
ALKBDE10	100.69	-0.77	3.36	0.03	4.44	9.66	9.58	0.08
RC21	35.70	-0.22	1.44	0.04	1.72	3.73	3.56	0.17
ALK8	62.60	2.63	2.63	0.04	4.18	11.15	10.74	0.41
DC13	54.98	0.23	4.72	0.09	6.32	13.71	13.58	0.13
G2RC	51.26	-2.06	2.68	0.05	3.33	9.53	9.23	0.30
BH76RC	21.39	-0.48	1.16	0.05	1.65	4.95	4.94	0.01
MOR23	35.57	-6.53	7.79	0.22	9.10	21.51	21.04	0.47
WCPT18	34.99	2.33	2.33	0.07	2.78	5.47	4.94	0.53
BHROT27	6.37	0.27	0.27	0.04	0.36	1.03	1.02	0.01

Continued on next page

Test set	$\lvert\overline{\Delta E}\rvert$	MD	MAD	NMAD	RMSD	Δ_{err}	max	min
BHPERI	20.87	2.30	2.30	0.11	2.66	6.34	6.01	0.33
BHDIV10	45.33	1.46	1.46	0.03	1.87	4.25	4.10	0.15
INV24	32.85	1.71	1.71	0.05	2.07	4.52	4.25	0.27
CR20	19.31	-3.23	3.23	0.17	3.31	6.65	4.61	2.04
CRBH20	46.13	0.23	0.51	0.01	0.60	1.26	1.23	0.03
TMBH17	12.76	0.51	2.59	0.20	3.39	8.05	8.00	0.05
LTMBH26	9.98	-0.43	1.16	0.12	1.80	6.91	6.88	0.03
BH76	18.61	-0.60	1.67	0.09	2.33	8.75	8.75	0.00
ISO34	14.57	0.71	0.71	0.05	1.30	5.85	5.84	0.01
ICONF	3.27	0.28	0.28	0.08	0.38	0.85	0.84	0.01
ACONF	1.83	0.28	0.28	0.15	0.31	0.59	0.52	0.07
TAUT15	3.05	0.63	0.63	0.21	0.82	1.79	1.75	0.04
Amino20x4	2.44	0.28	0.28	0.12	0.36	1.03	1.02	0.01
PCONF	1.62	1.23	1.23	0.76	1.62	3.05	3.04	0.01
MCONF	4.97	1.27	1.27	0.26	1.42	2.30	2.23	0.07
SCONF	4.60	3.84	3.84	0.83	4.24	8.65	8.47	0.18
PArel	4.63	0.15	0.59	0.13	0.80	1.79	1.78	0.01
BUT14DIOL	2.80	0.70	0.70	0.25	0.72	0.95	0.93	0.02
EIE22	5.44	0.53	0.54	0.10	0.62	1.38	1.35	0.03
Styrene45	62.64	3.37	3.40	0.05	4.11	10.18	10.18	0.00
ISOMERIZATION20	31.84	-0.92	1.57	0.05	2.06	4.87	4.82	0.05
DIE60	4.71	0.53	0.53	0.11	0.63	1.35	1.30	0.05
IDISP	14.22	6.92	6.92	0.49	9.72	23.34	21.57	1.77
C20C24	30.77	-0.41	19.64	0.64	21.43	41.67	32.38	9.29
S66	5.47	-0.13	0.13	0.02	0.18	0.59	0.59	0.00
S10x8	6.59	-0.17	0.20	0.03	0.27	0.59	0.59	0.00
X40	3.76	0.16	0.16	0.04	0.24	0.84	0.84	0.00
HEAVY28	1.24	0.51	0.52	0.42	0.60	1.39	1.39	0.00
CHB6	26.79	0.32	0.42	0.02	0.57	1.13	1.08	0.05
AHB21	22.49	-0.88	0.92	0.04	1.26	2.92	2.91	0.01
IL16	109.04	0.48	0.57	0.01	0.72	1.51	1.51	0.00
PNICO23	4.27	0.55	0.55	0.13	0.59	0.90	0.90	0.00
CT20	0.98	0.06	0.06	0.07	0.08	0.17	0.16	0.01
CARBHB12	6.04	1.38	1.38	0.23	1.49	2.60	2.18	0.42
ADIM6	3.36	2.11	2.11	0.63	2.34	4.41	3.66	0.75
3B-69-TRIM	12.30	-0.20	0.32	0.03	0.44	1.33	1.33	0.00

Table S18: Statistical analysis for B2T-PLYP-D3(BJ) for all testset in our databsase. The numbers given (all in kcal/mol) are average reaction energy ($|\overline{\Delta E}|$), mean deviation (MD), mean absolute deviation (MAD), MAD normalized with respect to $|\overline{\Delta E}|$ (NMAD), root-mean-square deviation (RMSD), deviation span (Δ_{err}), maximum (max) and minimum deviation (min).

Test set	$\lvert\overline{\Delta E}\rvert$	MD	MAD	NMAD	RMSD	Δ_{err}	max	min
FH51	31.01	1.01	1.01	0.03	1.42	3.82	3.81	0.01
YBDE18	49.28	-1.39	2.18	0.04	2.29	5.00	4.01	0.99
AL2X6	35.88	-0.45	0.64	0.02	0.71	1.37	1.12	0.25
DARC	32.47	1.13	1.13	0.03	1.30	2.66	2.33	0.33
NBPRC	27.71	0.27	1.75	0.06	2.07	3.45	3.43	0.02
HEAVYSB9	58.02	-0.87	1.64	0.03	2.20	5.31	5.16	0.15
BSR36	16.20	0.57	0.74	0.05	1.00	3.68	3.59	0.09
RSE43	7.60	0.01	0.34	0.04	0.52	2.31	2.30	0.01
W4-11	306.91	-4.60	4.73	0.02	5.65	16.60	16.54	0.06
G21EA	33.62	-6.02	6.13	0.18	6.50	12.53	11.14	1.39
G21IP	257.61	-1.22	2.46	0.01	2.95	8.11	8.06	0.05
DIPCS10	654.26	-3.34	3.60	0.01	4.48	10.03	9.53	0.50
PA26	189.05	0.81	1.49	0.01	2.16	6.38	6.30	0.08
SIE4x4	33.72	7.40	7.40	0.22	8.66	17.43	17.38	0.05
ALKBDE10	100.69	-0.39	3.48	0.03	4.35	9.12	8.92	0.20
RC21	35.70	-0.11	0.96	0.03	1.14	3.22	2.99	0.23
ALK8	62.60	1.63	1.78	0.03	3.22	8.47	8.42	0.05
DC13	54.98	1.27	5.64	0.10	8.27	18.25	17.93	0.32
G2RC	51.26	-0.92	2.03	0.04	2.44	7.04	6.53	0.51
BH76RC	21.39	-0.44	1.01	0.05	1.42	4.77	4.64	0.13
MOR23	35.57	-4.60	5.63	0.16	7.09	17.60	17.51	0.09
WCPT18	34.99	2.18	2.18	0.06	2.71	5.01	4.99	0.02
BHROT27	6.37	0.26	0.26	0.04	0.36	1.05	1.04	0.01
BHPERI	20.87	1.66	1.66	0.08	1.89	4.23	3.94	0.29
BHDIV10	45.33	1.80	1.80	0.04	2.19	4.64	4.37	0.27
INV24	32.85	1.34	1.34	0.04	1.56	3.15	2.97	0.18
CR20	19.31	-4.97	4.97	0.26	5.01	9.80	6.14	3.66
CRBH20	46.13	-2.90	2.90	0.06	2.95	6.06	4.17	1.89
TMBH17	12.76	0.31	2.71	0.21	3.81	9.77	9.70	0.07
LTMBH26	9.98	-1.35	2.08	0.21	2.92	9.57	9.47	0.10
BH76	18.61	-2.00	2.38	0.13	2.84	7.33	7.19	0.14
ISO34	14.57	0.95	0.95	0.07	1.57	7.03	7.02	0.01
ICONF	3.27	0.23	0.23	0.07	0.33	0.81	0.81	0.00
ACONF	1.83	0.15	0.15	0.08	0.17	0.34	0.31	0.03
TAUT15	3.05	0.72	0.72	0.24	0.86	1.85	1.81	0.04
Amino20x4	2.44	0.21	0.21	0.08	0.27	0.88	0.88	0.00
PCONF	1.62	0.73	0.73	0.45	0.98	2.07	2.07	0.00
MCONF	4.97	0.86	0.86	0.17	0.95	1.53	1.52	0.01

Continued on next page

Test set	$\|\overline{\Delta E}\|$	MD	MAD	NMAD	RMSD	Δ_{err}	max	min
SCONF	4.60	3.84	3.84	0.83	4.24	8.65	8.47	0.18
PArel	4.63	0.05	0.61	0.13	0.92	2.19	2.19	0.00
BUT14DIOL	2.80	0.58	0.58	0.21	0.59	0.72	0.70	0.02
EIE22	5.44	0.73	0.73	0.13	0.83	1.69	1.67	0.02
Styrene45	62.64	3.50	3.80	0.06	4.80	12.75	12.75	0.00
ISOMERIZATION20	31.84	-0.55	1.47	0.05	1.99	4.45	4.40	0.05
DIE60	4.71	0.66	0.66	0.14	0.74	1.55	1.28	0.27
IDISP	14.22	3.47	3.47	0.24	5.04	11.47	11.05	0.42
C20C24	30.77	0.27	27.01	0.88	28.88	59.10	43.31	15.79
S66	5.47	-0.23	0.23	0.04	0.26	0.58	0.58	0.00
S10x8	6.59	-0.02	0.15	0.02	0.22	0.69	0.69	0.00
X40	3.76	0.16	0.16	0.04	0.18	0.42	0.41	0.01
HEAVY28	1.24	0.35	0.37	0.30	0.44	1.20	1.17	0.03
CHB6	26.79	0.39	0.53	0.02	0.72	1.46	1.45	0.01
AHB21	22.49	-0.77	0.84	0.04	1.14	2.56	2.55	0.01
IL16	109.04	0.60	0.66	0.01	0.80	1.67	1.54	0.13
PNICO23	4.27	0.31	0.32	0.07	0.35	0.71	0.63	0.08
CT20	0.98	0.11	0.12	0.12	0.14	0.27	0.26	0.01
CARBHB12	6.04	1.17	1.17	0.19	1.28	2.35	2.02	0.33
ADIM6	3.36	1.33	1.33	0.40	1.48	2.77	2.32	0.45
3B-69-TRIM	12.30	0.29	0.42	0.03	0.52	1.85	1.82	0.03

Table S19: Statistical analysis for B2π-PLYP-D3(BJ) for all testset in our databsase. The numbers given (all in kcal/mol) are average reaction energy ($|\overline{\Delta E}|$), mean deviation (MD), mean absolute deviation (MAD), MAD normalized with respect to $|\overline{\Delta E}|$ (NMAD), root-mean-square deviation (RMSD), deviation span (Δ_{err}), maximum (max) and minimum deviation (min).

Test set	$\|\overline{\Delta E}\|$	MD	MAD	NMAD	RMSD	Δ_{err}	max	min
FH51	31.01	1.30	1.30	0.04	1.77	4.77	4.73	0.04
YBDE18	49.28	-0.62	1.28	0.03	1.49	3.22	3.15	0.07
AL2X6	35.88	0.19	0.51	0.01	0.71	1.50	1.42	0.08
DARC	32.47	-0.43	0.67	0.02	0.81	1.60	1.50	0.10
NBPRC	27.71	-0.13	1.86	0.07	2.05	3.27	2.88	0.39
HEAVYSB9	58.02	-0.39	1.49	0.03	1.94	4.61	4.51	0.10
BSR36	16.20	0.17	0.56	0.03	0.68	2.35	2.25	0.10
RSE43	7.60	-0.10	0.33	0.04	0.47	1.63	1.61	0.02
W4-11	306.91	-2.37	3.68	0.01	5.13	19.71	19.65	0.06
G21EA	33.62	-5.06	5.25	0.16	5.70	12.46	10.11	2.35
G21IP	257.61	0.09	2.24	0.01	2.84	6.77	6.71	0.06
DIPCS10	654.26	-0.68	2.74	0.00	3.55	7.81	7.76	0.05
PA26	189.05	0.98	1.61	0.01	2.30	6.60	6.58	0.02
SIE4x4	33.72	7.23	7.23	0.21	8.54	17.82	17.77	0.05

Continued on next page

Test set	$\lvert\overline{\Delta E}\rvert$	MD	MAD	NMAD	RMSD	Δ_{err}	max	min
ALKBDE10	100.69	-0.62	3.42	0.03	4.19	8.77	8.76	0.01
RC21	35.70	0.96	1.25	0.03	1.55	4.12	4.06	0.06
ALK8	62.60	2.23	2.35	0.04	3.97	10.30	10.27	0.03
DC13	54.98	0.09	5.70	0.10	7.49	17.36	16.78	0.58
G2RC	51.26	-1.97	2.77	0.05	3.41	7.79	7.78	0.01
BH76RC	21.39	-0.68	1.48	0.07	1.90	5.01	4.87	0.14
MOR23	35.57	-4.63	5.37	0.15	6.67	18.26	18.05	0.21
WCPT18	34.99	2.28	2.28	0.07	2.71	4.97	4.72	0.25
BHROT27	6.37	0.33	0.33	0.05	0.46	1.17	1.17	0.00
BHPERI	20.87	1.29	1.29	0.06	1.58	3.57	3.51	0.06
BHDIV10	45.33	1.67	1.67	0.04	2.05	4.65	4.57	0.08
INV24	32.85	1.43	1.43	0.04	1.72	4.02	3.94	0.08
CR20	19.31	-3.65	3.65	0.19	3.71	7.35	5.04	2.31
CRBH20	46.13	-1.63	1.63	0.04	1.72	3.56	2.83	0.73
TMBH17	12.76	0.91	2.48	0.19	3.80	10.04	10.00	0.04
LTMBH26	9.98	-1.04	1.90	0.19	2.87	10.70	10.67	0.03
BH76	18.61	-1.96	2.42	0.13	2.80	7.31	6.96	0.35
ISO34	14.57	0.82	0.82	0.06	1.46	6.95	6.94	0.01
ICONF	3.27	0.27	0.27	0.08	0.39	0.95	0.94	0.01
ACONF	1.83	0.17	0.17	0.10	0.19	0.38	0.34	0.04
TAUT15	3.05	0.72	0.72	0.23	0.87	1.68	1.64	0.04
Amino20x4	2.44	0.21	0.21	0.09	0.29	0.88	0.88	0.00
PCONF	1.62	0.75	0.75	0.46	1.02	2.11	2.10	0.01
MCONF	4.97	0.86	0.86	0.17	0.95	1.51	1.50	0.01
SCONF	4.60	3.84	3.84	0.83	4.24	8.65	8.47	0.18
PArel	4.63	0.17	0.56	0.12	0.87	2.49	2.49	0.00
BUT14DIOL	2.80	0.64	0.64	0.23	0.66	0.80	0.78	0.02
EIE22	5.44	0.72	0.72	0.13	0.81	1.66	1.63	0.03
Styrene45	62.64	3.87	3.96	0.06	4.82	11.79	11.79	0.00
ISOMERIZATION20	31.84	-0.69	1.46	0.05	1.97	4.10	4.09	0.01
DIE60	4.71	0.65	0.65	0.14	0.75	1.56	1.42	0.14
IDISP	14.22	3.36	3.36	0.24	5.02	11.36	10.94	0.42
C20C24	30.77	0.13	24.34	0.79	25.97	53.93	38.91	15.02
S66	5.47	-0.16	0.16	0.03	0.24	0.81	0.81	0.00
S10x8	6.59	-0.28	0.33	0.05	0.47	1.43	1.43	0.00
X40	3.76	0.16	0.16	0.04	0.24	0.89	0.88	0.01
HEAVY28	1.24	0.42	0.43	0.35	0.50	1.27	1.25	0.02
CHB6	26.79	-0.11	0.55	0.02	0.65	1.06	1.02	0.04
AHB21	22.49	-1.25	1.25	0.06	1.63	3.80	3.74	0.06
IL16	109.04	0.14	0.44	0.00	0.52	1.03	1.00	0.03
PNICO23	4.27	0.49	0.49	0.11	0.52	0.93	0.82	0.11
CT20	0.98	0.01	0.07	0.07	0.09	0.24	0.23	0.01
CARBHB12	6.04	1.36	1.36	0.22	1.47	2.63	2.23	0.40
ADIM6	3.36	1.44	1.44	0.43	1.58	2.99	2.47	0.52

Continued on next page

Test set	$\|\overline{\Delta E}\|$	MD	MAD	NMAD	RMSD	Δ_{err}	max	min
3B-69-TRIM	12.30	-0.31	0.48	0.04	0.63	1.54	1.54	0.00

Table S20: Statistical analysis for B2NC-PLYP-D3(BJ) for all testset in our databsase. The numbers given (all in kcal/mol) are average reaction energy ($|\overline{\Delta E}|$), mean deviation (MD), mean absolute deviation (MAD), MAD normalized with respect to $|\overline{\Delta E}|$ (NMAD), root-mean-square deviation (RMSD), deviation span (Δ_{err}), maximum (max) and minimum deviation (min).

Test set	$\|\overline{\Delta E}\|$	MD	MAD	NMAD	RMSD	Δ_{err}	max	min
FH51	31.01	1.15	1.15	0.04	1.59	4.63	4.62	0.01
YBDE18	49.28	1.65	1.84	0.04	3.43	10.43	10.39	0.04
AL2X6	35.88	0.67	0.87	0.02	1.01	2.05	1.77	0.28
DARC	32.47	-0.74	1.01	0.03	1.36	2.51	2.48	0.03
NBPRC	27.71	-0.52	1.29	0.05	1.56	3.25	3.25	0.00
HEAVYSB9	58.02	1.95	2.60	0.04	2.97	5.17	4.99	0.18
BSR36	16.20	1.64	1.67	0.10	2.33	7.56	7.45	0.11
RSE43	7.60	0.39	0.65	0.08	1.28	5.80	5.80	0.00
W4-11	306.91	1.73	4.08	0.01	5.24	14.19	14.06	0.13
G21EA	33.62	-5.61	5.61	0.17	5.89	10.42	9.91	0.51
G21IP	257.61	-1.38	2.46	0.01	3.02	8.66	8.53	0.13
DIPCS10	654.26	-4.04	4.04	0.01	4.53	7.48	7.02	0.46
PA26	189.05	-0.13	1.41	0.01	1.81	4.87	4.54	0.33
SIE4x4	33.72	6.75	6.79	0.20	7.86	13.83	13.51	0.32
ALKBDE10	100.69	4.74	6.51	0.06	7.81	12.94	12.91	0.03
RC21	35.70	-1.69	2.13	0.06	2.75	6.40	6.26	0.14
ALK8	62.60	-0.53	1.67	0.03	2.06	3.74	3.58	0.16
DC13	54.98	2.56	6.10	0.11	8.34	20.31	20.20	0.11
G2RC	51.26	-0.17	2.20	0.04	3.00	8.13	8.13	0.00
BH76RC	21.39	-0.27	1.60	0.07	2.01	5.07	4.94	0.13
MOR23	35.57	-4.19	6.63	0.19	9.39	23.41	23.22	0.19
WCPT18	34.99	4.04	4.04	0.12	4.54	8.51	7.62	0.89
BHROT27	6.37	0.20	0.20	0.03	0.28	0.94	0.93	0.01
BHPERI	20.87	4.78	4.78	0.23	5.04	11.18	9.20	1.98
BHDIV10	45.33	2.68	2.68	0.06	3.14	5.93	5.50	0.43
INV24	32.85	1.79	1.79	0.05	2.36	7.14	7.14	0.00
CR20	19.31	-4.32	4.32	0.22	4.38	9.05	5.97	3.08
CRBH20	46.13	-1.93	1.93	0.04	2.04	4.07	3.29	0.78
TMBH17	12.76	-1.24	3.24	0.25	3.90	7.00	6.85	0.15
LTMBH26	9.98	-1.46	1.94	0.19	2.49	6.87	6.87	0.00
BH76	18.61	-2.17	2.88	0.15	3.52	8.47	8.47	0.00
ISO34	14.57	0.98	0.98	0.07	1.45	5.62	5.61	0.01
ICONF	3.27	0.19	0.19	0.06	0.26	0.62	0.62	0.00
ACONF	1.83	0.35	0.35	0.19	0.39	0.74	0.65	0.09

Continued on next page

Test set	$\|\overline{\Delta E}\|$	MD	MAD	NMAD	RMSD	Δ_{err}	max	min
TAUT15	3.05	0.80	0.80	0.26	1.00	1.92	1.89	0.03
Amino20x4	2.44	0.36	0.36	0.15	0.45	1.32	1.32	0.00
PCONF	1.62	0.66	0.66	0.41	0.80	1.83	1.79	0.04
MCONF	4.97	1.49	1.49	0.30	1.67	2.72	2.71	0.01
SCONF	4.60	3.84	3.84	0.83	4.24	8.65	8.47	0.18
PArel	4.63	-0.08	0.94	0.20	1.36	3.75	3.71	0.04
BUT14DIOL	2.80	0.64	0.64	0.23	0.65	0.80	0.74	0.06
EIE22	5.44	0.69	0.71	0.13	0.81	1.83	1.81	0.02
Styrene45	62.64	2.42	2.89	0.05	3.77	11.04	11.04	0.00
ISOMERIZATION20	31.84	-0.47	2.27	0.07	2.99	7.35	7.34	0.01
DIE60	4.71	0.65	0.65	0.14	0.70	1.51	1.30	0.21
IDISP	14.22	8.44	8.44	0.59	11.03	25.49	23.41	2.08
C20C24	30.77	-1.36	21.32	0.69	24.22	43.96	37.43	6.53
S66	5.47	-0.26	0.26	0.05	0.31	0.77	0.75	0.02
S10x8	6.59	0.59	0.59	0.09	0.78	2.43	2.42	0.01
X40	3.76	0.21	0.21	0.06	0.26	0.85	0.83	0.02
HEAVY28	1.24	0.55	0.56	0.45	0.63	1.59	1.46	0.13
CHB6	26.79	0.97	0.97	0.04	1.09	2.00	1.59	0.41
AHB21	22.49	-0.17	0.59	0.03	0.72	1.60	1.58	0.02
IL16	109.04	0.49	0.72	0.01	0.81	1.74	1.57	0.17
PNICO23	4.27	-0.20	0.29	0.07	0.33	0.87	0.82	0.05
CT20	0.98	0.31	0.31	0.32	0.33	0.61	0.49	0.12
CARBHB12	6.04	1.02	1.02	0.17	1.15	2.03	1.88	0.15
ADIM6	3.36	1.04	1.04	0.31	1.18	2.21	1.93	0.28
3B-69-TRIM	12.30	1.94	1.94	0.16	2.10	4.61	4.56	0.05

Table S21: Statistical analysis for mPW2-PLYP-D3(BJ) for all testset in our databsase. The numbers given (all in kcal/mol) are average reaction energy ($|\overline{\Delta E}|$), mean deviation (MD), mean absolute deviation (MAD), MAD normalized with respect to $|\overline{\Delta E}|$ (NMAD), root-mean-square deviation (RMSD), deviation span (Δ_{err}), maximum (max) and minimum deviation (min).

Test set	$\|\overline{\Delta E}\|$	MD	MAD	NMAD	RMSD	Δ_{err}	max	min
FH51	31.01	1.18	1.18	0.04	1.61	4.52	4.48	0.04
YBDE18	49.28	-1.58	2.31	0.05	2.43	5.02	3.92	1.10
AL2X6	35.88	-0.45	0.63	0.02	0.77	1.29	1.20	0.09
DARC	32.47	1.35	1.35	0.04	1.49	3.12	2.59	0.53
NBPRC	27.71	0.02	2.01	0.07	2.31	3.56	3.50	0.06
HEAVYSB9	58.02	-1.42	1.87	0.03	2.41	5.61	5.48	0.13
BSR36	16.20	0.28	0.81	0.05	0.93	2.11	2.07	0.04
RSE43	7.60	-0.16	0.34	0.04	0.46	1.29	1.29	0.00
W4-11	306.91	-4.46	4.63	0.02	5.72	18.81	18.74	0.07
G21EA	33.62	-5.47	5.68	0.17	6.09	13.25	10.56	2.69

Continued on next page

Test set	$\|\overline{\Delta E}\|$	MD	MAD	NMAD	RMSD	Δ_{err}	max	min
G21IP	257.61	-0.56	2.44	0.01	2.97	6.99	6.91	0.08
DIPCS10	654.26	-1.86	3.13	0.00	4.05	9.45	8.85	0.60
PA26	189.05	0.90	1.56	0.01	2.30	6.70	6.69	0.01
SIE4x4	33.72	8.72	8.72	0.26	10.12	20.34	20.13	0.21
ALKBDE10	100.69	-0.66	3.63	0.04	4.36	9.71	9.24	0.47
RC21	35.70	0.64	1.21	0.03	1.40	2.68	2.51	0.17
ALK8	62.60	1.28	2.79	0.04	3.95	9.43	9.25	0.18
DC13	54.98	0.96	6.92	0.13	9.74	21.76	21.53	0.23
G2RC	51.26	-1.03	2.30	0.04	2.71	6.13	6.08	0.05
BH76RC	21.39	-0.46	1.25	0.06	1.62	4.50	4.50	0.00
MOR23	35.57	-4.27	5.15	0.14	6.74	19.89	19.30	0.59
WCPT18	34.99	2.41	2.41	0.07	2.98	5.55	5.31	0.24
BHROT27	6.37	0.29	0.29	0.05	0.40	1.07	1.07	0.00
BHPERI	20.87	1.88	1.88	0.09	2.13	4.32	4.25	0.07
BHDIV10	45.33	1.96	1.96	0.04	2.36	5.14	4.75	0.39
INV24	32.85	1.14	1.14	0.03	1.39	2.86	2.85	0.01
CR20	19.31	-4.72	4.72	0.24	4.76	9.37	5.99	3.38
CRBH20	46.13	-4.19	4.19	0.09	4.22	8.62	5.39	3.23
TMBH17	12.76	0.56	2.88	0.23	4.17	11.38	11.06	0.32
LTMBH26	9.98	-1.06	2.07	0.21	3.04	11.14	10.86	0.28
BH76	18.61	-2.78	3.03	0.16	3.49	7.84	7.73	0.11
ISO34	14.57	1.14	1.14	0.08	1.80	7.81	7.80	0.01
ICONF	3.27	0.25	0.25	0.08	0.36	0.88	0.86	0.02
ACONF	1.83	0.28	0.28	0.15	0.31	0.63	0.57	0.06
TAUT15	3.05	0.74	0.74	0.24	0.88	1.82	1.78	0.04
Amino20x4	2.44	0.26	0.26	0.10	0.34	0.99	0.99	0.00
PCONF	1.62	1.10	1.10	0.68	1.49	2.83	2.78	0.05
MCONF	4.97	1.14	1.14	0.23	1.28	2.03	2.01	0.02
SCONF	4.60	3.84	3.84	0.83	4.24	8.65	8.47	0.18
PArel	4.63	0.01	0.65	0.14	0.96	2.63	2.61	0.02
BUT14DIOL	2.80	0.65	0.65	0.23	0.67	0.88	0.87	0.01
EIE22	5.44	0.74	0.75	0.14	0.84	1.82	1.79	0.03
Styrene45	62.64	4.21	4.47	0.07	5.71	14.95	14.95	0.00
ISOMERIZATION20	31.84	-0.52	1.35	0.04	1.87	3.99	3.96	0.03
DIE60	4.71	0.64	0.64	0.13	0.71	1.30	1.25	0.05
IDISP	14.22	3.94	3.94	0.28	5.07	9.98	9.20	0.78
C20C24	30.77	1.60	30.18	0.98	31.70	65.77	45.80	19.97
S66	5.47	-0.82	0.82	0.15	0.88	1.82	1.52	0.30
S10x8	6.59	-0.70	0.70	0.11	0.80	1.57	1.53	0.04
X40	3.76	0.46	0.46	0.12	0.55	1.75	1.55	0.20
HEAVY28	1.24	0.52	0.52	0.42	0.58	1.36	1.31	0.05
CHB6	26.79	0.00	0.75	0.03	0.91	1.80	1.67	0.13
AHB21	22.49	-1.32	1.32	0.06	1.55	3.47	3.09	0.38
IL16	109.04	-0.39	0.50	0.00	0.58	1.05	1.01	0.04

Continued on next page

Test set	$\|\overline{\Delta E}\|$	MD	MAD	NMAD	RMSD	Δ_{err}	max	min
PNICO23	4.27	0.73	0.73	0.17	0.76	1.28	1.15	0.13
CT20	0.98	-0.31	0.31	0.31	0.32	0.76	0.57	0.19
CARBHB12	6.04	1.59	1.59	0.26	1.69	3.06	2.44	0.62
ADIM6	3.36	2.67	2.67	0.79	2.89	5.45	4.33	1.12
3B-69-TRIM	12.30	-1.75	1.75	0.14	1.84	3.73	3.12	0.61

Table S22: Statistical analysis for mPW2NC-PLYP-D3(BJ) for all testset in our databsase. The numbers given (all in kcal/mol) are average reaction energy ($|\overline{\Delta E}|$), mean deviation (MD), mean absolute deviation (MAD), MAD normalized with respect to $|\overline{\Delta E}|$ (NMAD), root-mean-square deviation (RMSD), deviation span (Δ_{err}), maximum (max) and minimum deviation (min).

Test set	$\|\overline{\Delta E}\|$	MD	MAD	NMAD	RMSD	Δ_{err}	max	min
FH51	31.01	1.06	1.06	0.03	1.41	3.58	3.57	0.01
YBDE18	49.28	-0.77	2.04	0.04	2.61	6.82	6.81	0.01
AL2X6	35.88	0.21	0.65	0.02	0.74	1.33	1.14	0.19
DARC	32.47	0.33	0.86	0.03	0.97	2.20	2.09	0.11
NBPRC	27.71	-0.07	1.66	0.06	1.93	2.80	2.79	0.01
HEAVYSB9	58.02	-0.23	1.81	0.03	2.37	5.46	5.36	0.10
BSR36	16.20	0.07	0.39	0.02	0.68	2.96	2.96	0.00
RSE43	7.60	0.49	0.55	0.07	1.07	4.81	4.80	0.01
W4-11	306.91	-7.20	7.43	0.02	8.64	22.78	22.60	0.18
G21EA	33.62	-6.90	6.90	0.21	7.28	12.35	12.29	0.06
G21IP	257.61	-2.22	2.86	0.01	3.43	9.37	9.29	0.08
DIPCS10	654.26	-5.34	5.34	0.01	5.92	11.81	10.60	1.21
PA26	189.05	0.18	1.33	0.01	1.88	5.30	5.26	0.04
SIE4x4	33.72	5.70	5.75	0.17	6.74	13.20	12.78	0.42
ALKBDE10	100.69	0.29	3.74	0.04	4.71	8.57	8.54	0.03
RC21	35.70	-1.82	2.02	0.06	2.51	5.73	5.61	0.12
ALK8	62.60	1.22	1.67	0.03	2.96	7.89	7.84	0.05
DC13	54.98	2.22	6.43	0.12	9.40	21.88	21.45	0.43
G2RC	51.26	-0.43	1.57	0.03	2.16	7.03	6.97	0.06
BH76RC	21.39	-0.20	1.06	0.05	1.49	4.50	4.47	0.03
MOR23	35.57	-4.40	6.10	0.17	7.83	18.10	17.19	0.91
WCPT18	34.99	2.49	2.49	0.07	3.07	5.63	5.61	0.02
BHROT27	6.37	0.19	0.19	0.03	0.28	0.91	0.90	0.01
BHPERI	20.87	2.85	2.85	0.14	3.10	7.54	6.33	1.21
BHDIV10	45.33	1.81	1.81	0.04	2.24	4.20	4.13	0.07
INV24	32.85	1.60	1.60	0.05	1.91	3.38	3.30	0.08
CR20	19.31	-4.96	4.96	0.26	5.00	10.16	6.27	3.89
CRBH20	46.13	-2.47	2.47	0.05	2.52	5.06	3.72	1.34
TMBH17	12.76	0.14	2.76	0.22	3.70	9.24	9.14	0.10
LTMBH26	9.98	-1.08	1.75	0.17	2.44	6.68	6.58	0.10

Continued on next page

Test set	$\lvert\overline{\Delta E}\rvert$	MD	MAD	NMAD	RMSD	Δ_{err}	max	min
BH76	18.61	-1.43	2.15	0.12	2.77	8.15	8.09	0.06
ISO34	14.57	1.03	1.03	0.07	1.58	6.61	6.58	0.03
ICONF	3.27	0.20	0.20	0.06	0.29	0.69	0.67	0.02
ACONF	1.83	0.26	0.26	0.14	0.29	0.55	0.50	0.05
TAUT15	3.05	0.66	0.66	0.22	0.83	1.96	1.93	0.03
Amino20x4	2.44	0.27	0.27	0.11	0.34	1.02	1.02	0.00
PCONF	1.62	0.41	0.41	0.26	0.53	1.29	1.28	0.01
MCONF	4.97	1.26	1.26	0.25	1.41	2.30	2.23	0.07
SCONF	4.60	3.84	3.84	0.83	4.24	8.65	8.47	0.18
PArel	4.63	-0.02	0.66	0.14	0.95	2.40	2.39	0.01
BUT14DIOL	2.80	0.50	0.50	0.18	0.51	0.74	0.66	0.08
EIE22	5.44	0.61	0.61	0.11	0.70	1.56	1.56	0.00
Styrene45	62.64	3.26	3.60	0.06	4.63	13.03	13.03	0.00
ISOMERIZATION20	31.84	-0.52	1.77	0.06	2.34	6.45	6.41	0.04
DIE60	4.71	0.56	0.56	0.12	0.64	1.11	1.10	0.01
IDISP	14.22	6.27	6.27	0.44	8.44	19.98	18.22	1.76
C20C24	30.77	0.49	25.89	0.84	27.88	54.48	41.28	13.20
S66	5.47	-0.17	0.17	0.03	0.24	0.74	0.74	0.00
S10x8	6.59	0.46	0.48	0.07	0.69	2.76	2.76	0.00
X40	3.76	0.15	0.15	0.04	0.24	0.91	0.91	0.00
HEAVY28	1.24	0.62	0.62	0.50	0.68	1.53	1.45	0.08
CHB6	26.79	0.47	0.54	0.02	0.70	1.35	1.27	0.08
AHB21	22.49	-0.33	0.65	0.03	0.82	1.87	1.85	0.02
IL16	109.04	0.54	0.63	0.01	0.77	1.62	1.58	0.04
PNICO23	4.27	-0.02	0.20	0.05	0.25	0.60	0.60	0.00
CT20	0.98	0.13	0.13	0.13	0.15	0.28	0.26	0.02
CARBHB12	6.04	0.98	0.98	0.16	1.07	1.93	1.65	0.28
ADIM6	3.36	0.82	0.82	0.24	0.89	1.72	1.39	0.33
3B-69-TRIM	12.30	1.57	1.57	0.13	1.76	4.49	4.44	0.05

Table S23: Statistical analysis for mPW2K-PLYP-D3(BJ) for all testset in our databsase. The numbers given (all in kcal/mol) are average reaction energy ($\lvert\overline{\Delta E}\rvert$), mean deviation (MD), mean absolute deviation (MAD), MAD normalized with respect to $\lvert\overline{\Delta E}\rvert$ (NMAD), root-mean-square deviation (RMSD), deviation span (Δ_{err}), maximum (max) and minimum deviation (min).

Test set	$\lvert\overline{\Delta E}\rvert$	MD	MAD	NMAD	RMSD	Δ_{err}	max	min
FH51	31.01	1.64	1.64	0.05	2.09	4.75	4.67	0.08
YBDE18	49.28	0.48	1.73	0.04	2.19	6.35	6.34	0.01
AL2X6	35.88	1.36	1.43	0.04	1.69	2.98	2.82	0.16
DARC	32.47	-2.69	2.69	0.08	2.88	4.70	4.19	0.51
NBPRC	27.71	-0.77	1.84	0.07	2.12	3.94	3.66	0.28
HEAVYSB9	58.02	0.58	2.05	0.04	2.43	5.31	4.69	0.62

Continued on next page

Test set	$\lvert\overline{\Delta E}\rvert$	MD	MAD	NMAD	RMSD	Δ_{err}	max	min
BSR36	16.20	2.40	2.46	0.15	3.03	9.06	8.91	0.15
RSE43	7.60	0.69	0.69	0.09	1.20	4.98	4.96	0.02
W4-11	306.91	-5.45	5.87	0.02	6.89	18.14	18.04	0.10
G21EA	33.62	-6.20	6.25	0.19	6.69	12.32	11.77	0.55
G21IP	257.61	-1.02	2.22	0.01	2.79	8.29	8.24	0.05
DIPCS10	654.26	-2.89	3.36	0.01	4.22	9.16	9.13	0.03
PA26	189.05	0.60	1.43	0.01	2.04	5.62	5.58	0.04
SIE4x4	33.72	4.33	4.40	0.13	5.15	11.06	10.49	0.57
ALKBDE10	100.69	-0.40	3.45	0.03	4.41	9.73	9.31	0.42
RC21	35.70	-0.03	1.43	0.04	1.74	3.60	3.55	0.05
ALK8	62.60	3.06	3.06	0.05	4.66	12.19	11.86	0.33
DC13	54.98	0.01	4.90	0.09	6.48	12.93	12.79	0.14
G2RC	51.26	-2.20	2.85	0.06	3.52	9.74	9.50	0.24
BH76RC	21.39	-0.48	1.20	0.06	1.66	4.93	4.92	0.01
MOR23	35.57	-6.94	8.13	0.23	9.48	22.47	22.39	0.08
WCPT18	34.99	2.48	2.48	0.07	2.96	5.80	5.18	0.62
BHROT27	6.37	0.28	0.28	0.04	0.37	1.04	1.03	0.01
BHPERI	20.87	2.54	2.54	0.12	2.91	6.65	6.43	0.22
BHDIV10	45.33	1.51	1.51	0.03	1.93	4.35	4.22	0.13
INV24	32.85	1.71	1.71	0.05	2.07	4.53	4.21	0.32
CR20	19.31	-2.94	2.94	0.15	3.02	5.99	4.32	1.67
CRBH20	46.13	0.06	0.45	0.01	0.56	1.06	1.06	0.00
TMBH17	12.76	0.48	2.64	0.21	3.47	8.30	8.28	0.02
LTMBH26	9.98	-0.81	1.41	0.14	2.02	6.74	6.72	0.02
BH76	18.61	-0.79	1.79	0.10	2.43	8.50	8.50	0.00
ISO34	14.57	0.76	0.76	0.05	1.35	6.03	6.02	0.01
ICONF	3.27	0.28	0.28	0.08	0.38	0.88	0.83	0.05
ACONF	1.83	0.32	0.32	0.17	0.35	0.67	0.59	0.08
TAUT15	3.05	0.62	0.62	0.20	0.81	1.76	1.73	0.03
Amino20x4	2.44	0.30	0.30	0.12	0.38	1.09	1.09	0.00
PCONF	1.62	1.41	1.41	0.87	1.82	3.31	3.30	0.01
MCONF	4.97	1.39	1.39	0.28	1.55	2.46	2.43	0.03
SCONF	4.60	3.84	3.84	0.83	4.24	8.65	8.47	0.18
PArel	4.63	0.13	0.58	0.12	0.79	1.74	1.73	0.01
BUT14DIOL	2.80	0.75	0.75	0.27	0.78	1.04	1.02	0.02
EIE22	5.44	0.52	0.52	0.10	0.61	1.40	1.38	0.02
Styrene45	62.64	3.58	3.60	0.06	4.35	10.90	10.90	0.00
ISOMERIZATION20	31.84	-0.95	1.56	0.05	2.04	4.77	4.72	0.05
DIE60	4.71	0.50	0.51	0.11	0.61	1.30	1.28	0.02
IDISP	14.22	7.34	7.34	0.52	10.05	23.94	21.94	2.00
C20C24	30.77	0.01	20.20	0.66	21.87	42.45	32.49	9.96
S66	5.47	-0.43	0.43	0.08	0.48	1.06	1.00	0.06
S10x8	6.59	-0.43	0.43	0.07	0.51	1.01	0.99	0.02
X40	3.76	0.36	0.36	0.10	0.45	1.46	1.39	0.07

Continued on next page

Test set	$\lvert\overline{\Delta E}\rvert$	MD	MAD	NMAD	RMSD	Δ_{err}	max	min
HEAVY28	1.24	0.70	0.71	0.57	0.78	1.66	1.58	0.08
CHB6	26.79	-0.03	0.41	0.02	0.47	0.81	0.73	0.08
AHB21	22.49	-1.15	1.15	0.05	1.45	3.27	3.16	0.11
IL16	109.04	0.08	0.45	0.00	0.52	1.08	1.07	0.01
PNICO23	4.27	0.81	0.81	0.19	0.84	1.49	1.24	0.25
CT20	0.98	-0.16	0.16	0.16	0.16	0.36	0.28	0.08
CARBHB12	6.04	1.60	1.60	0.26	1.70	2.99	2.40	0.59
ADIM6	3.36	2.64	2.64	0.79	2.88	5.47	4.42	1.05
3B-69-TRIM	12.30	-0.97	0.97	0.08	1.06	2.49	2.24	0.25

Table S24: Statistical analysis for PBE0-DH-D3(BJ) for all testset in our databsase. The numbers given (all in kcal/mol) are average reaction energy ($\lvert\overline{\Delta E}\rvert$), mean deviation (MD), mean absolute deviation (MAD), MAD normalized with respect to $\lvert\overline{\Delta E}\rvert$ (NMAD), root-mean-square deviation (RMSD), deviation span (Δ_{err}), maximum (max) and minimum deviation (min).

Test set	$\lvert\overline{\Delta E}\rvert$	MD	MAD	NMAD	RMSD	Δ_{err}	max	min
FH51	31.01	3.42	3.42	0.11	4.41	13.27	13.19	0.08
YBDE18	49.28	0.60	2.31	0.05	2.57	4.78	4.42	0.36
AL2X6	35.88	2.85	2.85	0.08	3.10	5.76	4.77	0.99
DARC	32.47	-9.07	9.07	0.28	9.16	19.26	11.89	7.37
NBPRC	27.71	-1.56	3.80	0.14	4.24	8.52	7.64	0.88
HEAVYSB9	58.02	-0.01	0.73	0.01	0.96	2.21	2.10	0.11
BSR36	16.20	-1.02	1.02	0.06	1.08	2.09	1.59	0.50
RSE43	7.60	-0.35	0.44	0.06	0.58	1.61	1.60	0.01
W4-11	306.91	-6.60	6.76	0.02	8.46	28.42	28.36	0.06
G21EA	33.62	-4.91	5.16	0.15	6.61	12.48	12.39	0.09
G21IP	257.61	0.49	3.06	0.01	3.79	8.27	8.19	0.08
DIPCS10	654.26	0.30	2.92	0.00	3.40	6.51	6.02	0.49
PA26	189.05	1.64	2.06	0.01	2.64	7.40	7.00	0.40
SIE4x4	33.72	6.93	6.93	0.21	8.21	18.72	18.01	0.71
ALKBDE10	100.69	-5.28	5.94	0.06	7.03	15.69	14.76	0.93
RC21	35.70	4.33	4.87	0.14	5.94	15.62	15.05	0.57
ALK8	62.60	7.72	7.75	0.12	9.94	16.55	16.44	0.11
DC13	54.98	-3.37	8.21	0.15	10.48	20.15	20.03	0.12
G2RC	51.26	-4.46	6.41	0.12	8.43	23.00	22.93	0.07
BH76RC	21.39	-0.51	2.11	0.10	2.68	6.52	6.47	0.05
MOR23	35.57	-5.59	5.91	0.17	7.20	18.42	18.31	0.11
WCPT18	34.99	3.12	3.12	0.09	3.91	7.41	7.25	0.16
BHROT27	6.37	0.54	0.54	0.08	0.72	1.36	1.35	0.01
BHPERI	20.87	3.36	3.36	0.16	3.81	6.72	6.57	0.15
BHDIV10	45.33	2.87	2.87	0.06	3.23	6.12	5.20	0.92
INV24	32.85	1.62	1.62	0.05	2.30	6.59	6.57	0.02

Continued on next page

Test set	$\lvert\overline{\Delta E}\rvert$	MD	MAD	NMAD	RMSD	Δ_{err}	max	min
CR20	19.31	3.10	3.10	0.16	3.20	5.82	4.39	1.43
CRBH20	46.13	4.22	4.22	0.09	4.29	8.26	5.52	2.74
TMBH17	12.76	1.21	3.62	0.28	4.21	7.22	7.02	0.20
LTMBH26	9.98	-0.43	2.02	0.20	3.05	12.77	12.50	0.27
BH76	18.61	-1.85	2.47	0.13	2.76	7.02	6.78	0.24
ISO34	14.57	1.32	1.32	0.09	1.84	4.08	3.99	0.09
ICONF	3.27	0.40	0.40	0.12	0.55	1.39	1.38	0.01
ACONF	1.83	0.14	0.14	0.08	0.16	0.32	0.29	0.03
TAUT15	3.05	0.84	0.84	0.27	1.11	2.36	2.25	0.11
Amino20x4	2.44	0.30	0.30	0.12	0.37	1.15	1.15	0.00
PCONF	1.62	0.49	0.49	0.30	0.58	1.22	1.16	0.06
MCONF	4.97	0.69	0.69	0.14	0.77	1.30	1.28	0.02
SCONF	4.60	3.84	3.84	0.83	4.24	8.65	8.47	0.18
PArel	4.63	0.74	1.19	0.26	1.51	3.60	3.56	0.04
BUT14DIOL	2.80	0.36	0.36	0.13	0.37	0.65	0.60	0.05
EIE22	5.44	0.78	0.82	0.15	0.95	1.94	1.88	0.06
Styrene45	62.64	1.18	3.40	0.05	4.11	9.08	9.08	0.00
ISOMERIZATION20	31.84	-0.59	1.75	0.05	2.20	4.71	4.69	0.02
DIE60	4.71	0.97	0.98	0.21	1.19	2.58	2.56	0.02
IDISP	14.22	3.36	3.36	0.24	5.94	14.17	13.97	0.20
C20C24	30.77	-10.12	10.12	0.33	10.28	21.53	13.50	8.03
S66	5.47	-0.27	0.27	0.05	0.38	1.46	1.44	0.02
S10x8	6.59	-0.25	0.31	0.05	0.54	2.34	2.33	0.01
X40	3.76	0.30	0.30	0.08	0.45	1.68	1.68	0.00
HEAVY28	1.24	0.56	0.57	0.46	0.65	1.65	1.56	0.09
CHB6	26.79	-0.32	0.52	0.02	0.75	1.70	1.69	0.01
AHB21	22.49	-1.92	1.93	0.09	2.51	5.82	5.77	0.05
IL16	109.04	-0.68	0.71	0.01	0.92	1.78	1.78	0.00
PNICO23	4.27	0.96	0.96	0.22	1.21	3.51	3.47	0.04
CT20	0.98	0.14	0.15	0.16	0.18	0.43	0.38	0.05
CARBHB12	6.04	1.57	1.57	0.26	1.78	3.57	3.08	0.49
ADIM6	3.36	0.36	0.36	0.11	0.39	0.78	0.61	0.17
3B-69-TRIM	12.30	0.19	0.69	0.06	0.87	2.60	2.59	0.01

Table S25: Statistical analysis for PBE0-2-D3(BJ) for all testset in our databsase. The numbers given (all in kcal/mol) are average reaction energy ($\lvert\overline{\Delta E}\rvert$), mean deviation (MD), mean absolute deviation (MAD), MAD normalized with respect to $\lvert\overline{\Delta E}\rvert$ (NMAD), root-mean-square deviation (RMSD), deviation span (Δ_{err}), maximum (max) and minimum deviation (min).

Test set	$\lvert\overline{\Delta E}\rvert$	MD	MAD	NMAD	RMSD	Δ_{err}	max	min
FH51	31.01	3.73	3.73	0.12	4.62	13.25	13.10	0.15
YBDE18	49.28	3.95	4.40	0.09	5.66	10.05	9.91	0.14

Continued on next page

Test set	$\|\overline{\Delta E}\|$	MD	MAD	NMAD	RMSD	Δ_{err}	max	min
AL2X6	35.88	4.34	4.34	0.12	4.62	9.47	7.20	2.27
DARC	32.47	-11.82	11.82	0.36	11.91	23.39	14.12	9.27
NBPRC	27.71	-2.59	3.92	0.14	4.68	9.93	9.90	0.03
HEAVYSB9	58.02	3.15	3.50	0.06	3.84	8.20	6.62	1.58
BSR36	16.20	3.02	3.03	0.19	4.02	11.98	11.70	0.28
RSE43	7.60	1.15	1.16	0.15	2.00	8.00	7.90	0.10
W4-11	306.91	-4.94	5.79	0.02	6.85	17.43	17.42	0.01
G21EA	33.62	-5.99	6.00	0.18	6.78	12.99	12.87	0.12
G21IP	257.61	-0.66	2.31	0.01	3.02	9.82	9.75	0.07
DIPCS10	654.26	-2.34	3.45	0.01	4.22	8.85	8.12	0.73
PA26	189.05	0.72	1.57	0.01	2.02	4.88	4.81	0.07
SIE4x4	33.72	1.17	1.50	0.04	1.85	3.64	3.63	0.01
ALKBDE10	100.69	-0.95	4.00	0.04	4.70	9.54	8.71	0.83
RC21	35.70	0.98	2.86	0.08	3.89	11.67	10.87	0.80
ALK8	62.60	4.92	4.92	0.08	6.59	13.10	12.55	0.55
DC13	54.98	-2.05	7.94	0.14	9.11	17.31	16.89	0.42
G2RC	51.26	-4.26	5.23	0.10	6.40	17.10	16.14	0.96
BH76RC	21.39	-0.45	2.16	0.10	2.75	6.45	6.32	0.13
MOR23	35.57	-7.91	9.33	0.26	10.94	20.28	19.94	0.34
WCPT18	34.99	3.48	3.48	0.10	4.28	7.40	7.37	0.03
BHROT27	6.37	0.38	0.38	0.06	0.47	1.12	1.11	0.01
BHPERI	20.87	5.38	5.38	0.26	6.00	13.04	11.25	1.79
BHDIV10	45.33	2.17	2.17	0.05	2.46	3.96	3.81	0.15
INV24	32.85	1.75	1.75	0.05	2.33	5.41	5.29	0.12
CR20	19.31	2.75	2.75	0.14	2.87	5.24	4.40	0.84
CRBH20	46.13	6.60	6.60	0.14	6.64	12.96	7.75	5.21
TMBH17	12.76	0.18	2.62	0.21	3.19	6.24	6.20	0.04
LTMBH26	9.98	0.05	1.17	0.12	1.61	4.82	4.80	0.02
BH76	18.61	0.37	2.02	0.11	2.92	11.18	11.12	0.06
ISO34	14.57	1.32	1.32	0.09	1.83	3.91	3.90	0.01
ICONF	3.27	0.44	0.44	0.14	0.55	1.07	1.04	0.03
ACONF	1.83	0.45	0.45	0.25	0.50	1.05	0.92	0.13
TAUT15	3.05	0.85	0.85	0.28	1.06	2.02	1.93	0.09
Amino20x4	2.44	0.44	0.44	0.18	0.56	1.55	1.55	0.00
PCONF	1.62	0.98	0.98	0.60	1.28	2.66	2.63	0.03
MCONF	4.97	1.81	1.81	0.36	2.04	3.17	3.17	0.00
SCONF	4.60	3.84	3.84	0.83	4.24	8.65	8.47	0.18
PArel	4.63	0.53	1.10	0.24	1.39	3.99	3.91	0.08
BUT14DIOL	2.80	0.65	0.65	0.23	0.67	0.85	0.84	0.01
EIE22	5.44	0.41	0.46	0.08	0.55	1.29	1.21	0.08
Styrene45	62.64	1.19	3.05	0.05	3.88	8.94	8.94	0.00
ISOMERIZATION20	31.84	-1.25	2.00	0.06	2.84	9.83	9.65	0.18
DIE60	4.71	0.61	0.61	0.13	0.77	1.95	1.86	0.09
IDISP	14.22	12.24	12.24	0.86	17.17	40.94	38.43	2.51

Continued on next page

Test set	$\lvert\overline{\Delta E}\rvert$	MD	MAD	NMAD	RMSD	Δ_{err}	max	min
C20C24	30.77	-8.11	8.11	0.26	9.69	16.77	15.16	1.61
S66	5.47	-0.39	0.39	0.07	0.50	1.32	1.30	0.02
S10x8	6.59	0.09	0.18	0.03	0.24	0.58	0.58	0.00
X40	3.76	0.35	0.35	0.09	0.55	1.78	1.78	0.00
HEAVY28	1.24	0.76	0.78	0.63	0.86	2.02	1.86	0.16
CHB6	26.79	0.38	0.45	0.02	0.47	0.85	0.66	0.19
AHB21	22.49	-1.10	1.18	0.05	1.68	3.92	3.89	0.03
IL16	109.04	0.62	0.83	0.01	0.97	1.71	1.69	0.02
PNICO23	4.27	0.61	0.61	0.14	0.68	1.54	1.44	0.10
CT20	0.98	0.27	0.27	0.27	0.28	0.59	0.44	0.15
CARBHB12	6.04	1.52	1.52	0.25	1.69	3.06	2.66	0.40
ADIM6	3.36	1.60	1.60	0.48	1.80	3.43	2.92	0.51
3B-69-TRIM	12.30	0.82	0.87	0.07	1.05	2.98	2.96	0.02

Table S26: Statistical analysis for LS1TPSS-D3(BJ) for all testset in our databsase. The numbers given (all in kcal/mol) are average reaction energy ($\lvert\overline{\Delta E}\rvert$), mean deviation (MD), mean absolute deviation (MAD), MAD normalized with respect to $\lvert\overline{\Delta E}\rvert$ (NMAD), root-mean-square deviation (RMSD), deviation span (Δ_{err}), maximum (max) and minimum deviation (min).

Test set	$\lvert\overline{\Delta E}\rvert$	MD	MAD	NMAD	RMSD	Δ_{err}	max	min
FH51	31.01	-2.39	2.81	0.09	3.61	9.12	9.06	0.06
YBDE18	49.28	3.90	4.76	0.10	6.05	11.38	11.31	0.07
AL2X6	35.88	4.33	4.33	0.12	4.65	9.02	7.11	1.91
DARC	32.47	-10.46	10.46	0.32	10.61	20.80	13.16	7.64
NBPRC	27.71	-2.54	3.55	0.13	4.19	9.37	8.85	0.52
HEAVYSB9	58.02	3.62	4.08	0.07	4.46	9.52	7.47	2.05
BSR36	16.20	3.69	3.70	0.23	4.87	13.96	13.92	0.04
RSE43	7.60	1.87	1.87	0.25	3.28	13.08	13.08	0.00
W4-11	306.91	-4.90	6.02	0.02	7.39	20.77	20.57	0.20
G21EA	33.62	-6.52	6.77	0.20	7.35	16.15	13.62	2.53
G21IP	257.61	-1.23	2.57	0.01	3.37	9.62	9.62	0.00
DIPCS10	654.26	-3.65	4.09	0.01	5.28	9.72	9.49	0.23
PA26	189.05	0.59	1.84	0.01	2.31	5.37	5.19	0.18
SIE4x4	33.72	0.43	0.99	0.03	1.20	2.50	2.48	0.02
ALKBDE10	100.69	-0.26	3.76	0.04	4.65	9.80	9.33	0.47
RC21	35.70	-1.01	3.05	0.09	3.66	9.31	9.04	0.27
ALK8	62.60	2.99	2.99	0.05	4.09	9.22	8.72	0.50
DC13	54.98	-0.98	7.04	0.13	8.34	15.51	14.75	0.76
G2RC	51.26	-3.48	4.19	0.08	5.15	12.50	11.97	0.53
BH76RC	21.39	-0.52	2.03	0.09	2.52	5.97	5.84	0.13
MOR23	35.57	-7.43	9.45	0.27	11.41	22.92	22.34	0.58
WCPT18	34.99	2.82	2.82	0.08	3.45	6.21	6.13	0.08

Continued on next page

Test set	$\lvert\overline{\Delta E}\rvert$	MD	MAD	NMAD	RMSD	Δ_{err}	max	min
BHROT27	6.37	0.30	0.32	0.05	0.40	1.03	1.00	0.03
BHPERI	20.87	-3.95	4.13	0.20	4.64	9.60	9.35	0.25
BHDIV10	45.33	-0.23	1.48	0.03	1.69	3.37	2.99	0.38
INV24	32.85	-0.15	2.23	0.07	2.78	5.19	5.07	0.12
CR20	19.31	1.39	1.56	0.08	1.71	3.86	3.25	0.61
CRBH20	46.13	6.66	6.66	0.14	6.70	12.94	7.81	5.13
TMBH17	12.76	0.21	2.73	0.21	3.51	7.34	7.26	0.08
LTMBH26	9.98	0.25	1.29	0.13	1.66	3.19	3.18	0.01
BH76	18.61	0.88	2.14	0.11	3.44	14.53	14.48	0.05
ISO34	14.57	1.14	1.14	0.08	1.61	4.65	4.65	0.00
ICONF	3.27	0.35	0.40	0.12	0.47	0.99	0.93	0.06
ACONF	1.83	-0.22	0.22	0.12	0.25	0.57	0.53	0.04
TAUT15	3.05	0.94	0.94	0.31	1.13	2.19	2.11	0.08
Amino20x4	2.44	0.08	0.30	0.12	0.38	1.02	1.02	0.00
PCONF	1.62	0.90	0.90	0.56	1.16	2.49	2.45	0.04
MCONF	4.97	1.08	1.08	0.22	1.18	1.99	1.87	0.12
SCONF	4.60	0.99	0.99	0.22	1.09	1.91	1.86	0.05
PArel	4.63	0.39	1.01	0.22	1.39	4.25	4.22	0.03
BUT14DIOL	2.80	0.69	0.69	0.25	0.72	0.96	0.94	0.02
EIE22	5.44	0.35	0.39	0.07	0.47	1.07	1.06	0.01
Styrene45	62.64	1.40	2.67	0.04	3.44	8.80	8.80	0.00
ISOMERIZATION20	31.84	-1.44	2.14	0.07	3.10	10.78	10.69	0.09
DIE60	4.71	0.55	0.55	0.12	0.71	1.74	1.72	0.02
IDISP	14.22	9.12	9.12	0.64	14.63	35.78	34.44	1.34
C20C24	30.77	-7.01	7.01	0.23	8.82	13.96	13.92	0.04
S66	5.47	-1.05	1.05	0.19	1.16	2.96	2.74	0.22
S10x8	6.59	0.34	0.35	0.05	0.45	1.06	1.06	0.00
X40	3.76	0.68	0.68	0.18	0.74	1.23	1.19	0.04
HEAVY28	1.24	0.72	0.73	0.59	0.82	1.87	1.79	0.08
CHB6	26.79	0.75	0.75	0.03	0.78	1.63	1.18	0.45
AHB21	22.49	-0.67	0.92	0.04	1.30	3.16	3.09	0.07
IL16	109.04	1.23	1.24	0.01	1.43	2.77	2.70	0.07
PNICO23	4.27	0.27	0.33	0.08	0.40	0.79	0.78	0.01
CT20	0.98	0.35	0.35	0.35	0.37	0.88	0.66	0.22
CARBHB12	6.04	1.34	1.34	0.22	1.49	2.46	2.21	0.25
ADIM6	3.36	1.78	1.78	0.53	1.99	3.81	3.24	0.57
3B-69-TRIM	12.30	1.46	1.48	0.12	1.63	4.07	3.72	0.35

Table S27: Statistical analysis for LS1DH-D3(BJ) for all testset in our databsase. The numbers given (all in kcal/mol) are average reaction energy ($|\overline{\Delta E}|$), mean deviation (MD), mean absolute deviation (MAD), MAD normalized with respect to $|\overline{\Delta E}|$ (NMAD), root-mean-square deviation (RMSD), deviation span (Δ_{err}), maximum (max) and minimum deviation (min).

Test set	$\lvert\overline{\Delta E}\rvert$	MD	MAD	NMAD	RMSD	Δ_{err}	max	min
FH51	31.01	3.45	3.45	0.11	4.22	11.38	11.33	0.05
YBDE18	49.28	3.76	3.76	0.08	4.45	8.04	7.98	0.06
AL2X6	35.88	3.56	3.56	0.10	3.76	7.01	5.31	1.70
DARC	32.47	-8.70	8.70	0.27	8.79	16.77	10.53	6.24
NBPRC	27.71	-2.27	3.54	0.13	3.95	7.86	7.31	0.55
HEAVYSB9	58.02	3.23	3.44	0.06	3.76	7.33	6.40	0.93
BSR36	16.20	1.41	1.49	0.09	2.02	6.71	6.68	0.03
RSE43	7.60	0.45	0.61	0.08	1.12	4.73	4.73	0.00
W4-11	306.91	3.13	5.88	0.02	7.73	22.14	22.13	0.01
G21EA	33.62	-1.84	2.41	0.07	3.15	7.95	7.95	0.00
G21IP	257.61	3.44	3.78	0.01	4.44	9.12	9.05	0.07
DIPCS10	654.26	4.86	5.15	0.01	6.06	10.53	9.93	0.60
PA26	189.05	0.43	1.45	0.01	1.96	4.88	4.78	0.10
SIE4x4	33.72	3.56	3.69	0.11	4.42	10.31	9.70	0.61
ALKBDE10	100.69	2.07	4.57	0.05	5.29	10.41	9.02	1.39
RC21	35.70	1.67	2.34	0.07	3.45	9.56	9.45	0.11
ALK8	62.60	2.65	2.65	0.04	3.64	9.24	8.77	0.47
DC13	54.98	-1.75	5.70	0.10	6.45	9.28	9.15	0.13
G2RC	51.26	-4.40	5.16	0.10	6.27	14.19	13.58	0.61
BH76RC	21.39	-1.17	1.72	0.08	2.30	6.37	6.20	0.17
MOR23	35.57	-5.67	6.74	0.19	8.05	16.18	16.05	0.13
WCPT18	34.99	3.33	3.33	0.10	4.18	7.00	6.92	0.08
BHROT27	6.37	0.47	0.47	0.07	0.61	1.46	1.45	0.01
BHPERI	20.87	3.89	3.89	0.19	4.41	9.68	8.66	1.02
BHDIV10	45.33	1.96	1.96	0.04	2.22	3.76	3.64	0.12
INV24	32.85	1.81	1.81	0.06	2.32	5.62	5.36	0.26
CR20	19.31	0.91	1.06	0.06	1.20	2.21	2.17	0.04
CRBH20	46.13	4.81	4.81	0.10	4.85	9.24	5.58	3.66
TMBH17	12.76	0.95	2.58	0.20	3.21	6.25	5.89	0.36
LTMBH26	9.98	0.06	1.03	0.10	1.73	7.18	7.17	0.01
BH76	18.61	-0.66	1.96	0.11	2.54	9.37	9.29	0.08
ISO34	14.57	1.02	1.02	0.07	1.45	3.76	3.71	0.05
ICONF	3.27	0.42	0.42	0.13	0.56	1.18	1.16	0.02
ACONF	1.83	0.42	0.42	0.23	0.47	0.97	0.84	0.13
TAUT15	3.05	0.80	0.80	0.26	1.11	2.41	2.37	0.04
Amino20x4	2.44	0.41	0.41	0.17	0.53	1.35	1.35	0.00
PCONF	1.62	0.76	0.76	0.47	1.03	2.25	2.22	0.03
MCONF	4.97	1.68	1.68	0.34	1.90	2.98	2.96	0.02

Continued on next page

Test set	$\|\overline{\Delta E}\|$	MD	MAD	NMAD	RMSD	Δ_{err}	max	min
SCONF	4.60	3.84	3.84	0.83	4.24	8.65	8.47	0.18
PArel	4.63	0.46	1.00	0.22	1.26	3.45	3.38	0.07
BUT14DIOL	2.80	0.76	0.76	0.27	0.78	0.99	0.95	0.04
EIE22	5.44	0.51	0.55	0.10	0.64	1.47	1.36	0.11
Styrene45	62.64	2.56	2.88	0.05	3.45	9.23	9.23	0.00
ISOMERIZATION20	31.84	-1.52	1.97	0.06	2.76	8.55	8.53	0.02
DIE60	4.71	0.61	0.61	0.13	0.76	1.79	1.75	0.04
IDISP	14.22	9.84	9.84	0.69	13.60	32.39	30.04	2.35
C20C24	30.77	-4.26	6.87	0.22	8.30	17.73	14.48	3.25
S66	5.47	-0.78	0.78	0.14	0.86	1.78	1.60	0.18
S10x8	6.59	-0.22	0.34	0.05	0.48	1.73	1.73	0.00
X40	3.76	0.60	0.60	0.16	0.76	2.05	1.99	0.06
HEAVY28	1.24	0.86	0.87	0.70	0.94	1.95	1.88	0.07
CHB6	26.79	0.03	0.22	0.01	0.26	0.46	0.45	0.01
AHB21	22.49	-1.62	1.63	0.07	2.13	5.10	5.00	0.10
IL16	109.04	-0.66	0.69	0.01	0.86	1.45	1.44	0.01
PNICO23	4.27	0.54	0.54	0.13	0.60	1.01	1.01	0.00
CT20	0.98	0.09	0.10	0.10	0.11	0.20	0.20	0.00
CARBHB12	6.04	1.65	1.65	0.27	1.80	3.14	2.68	0.46
ADIM6	3.36	1.60	1.60	0.48	1.78	3.39	2.82	0.57
3B-69-TRIM	12.30	0.08	0.62	0.05	0.80	2.60	2.59	0.01

Table S28: Statistical analysis for DS1TPSS-D3(BJ) for all testset in our databsase. The numbers given (all in kcal/mol) are average reaction energy ($|\overline{\Delta E}|$), mean deviation (MD), mean absolute deviation (MAD), MAD normalized with respect to $|\overline{\Delta E}|$ (NMAD), root-mean-square deviation (RMSD), deviation span (Δ_{err}), maximum (max) and minimum deviation (min).

Test set	$\|\overline{\Delta E}\|$	MD	MAD	NMAD	RMSD	Δ_{err}	max	min
FH51	31.01	-1.75	2.28	0.07	2.98	7.07	7.04	0.03
YBDE18	49.28	3.35	3.80	0.08	4.93	11.59	11.37	0.22
AL2X6	35.88	3.89	3.89	0.11	4.18	8.79	6.65	2.14
DARC	32.47	-8.04	8.04	0.25	8.17	16.03	10.35	5.68
NBPRC	27.71	-2.30	3.07	0.11	3.63	7.50	7.36	0.14
HEAVYSB9	58.02	3.85	4.09	0.07	4.46	8.33	7.25	1.08
BSR36	16.20	5.17	5.17	0.32	6.47	17.73	17.22	0.51
RSE43	7.60	1.04	1.04	0.14	2.00	8.35	8.32	0.03
W4-11	306.91	0.21	3.06	0.01	3.96	13.38	13.37	0.01
G21EA	33.62	-5.65	5.65	0.17	6.24	13.50	12.15	1.35
G21IP	257.61	-0.82	2.15	0.01	2.78	8.35	8.34	0.01
DIPCS10	654.26	-2.79	3.29	0.01	4.15	8.11	7.63	0.48
PA26	189.05	0.80	1.62	0.01	2.14	5.32	5.26	0.06
SIE4x4	33.72	3.96	4.02	0.12	4.74	9.19	8.73	0.46

Continued on next page

Test set	$\lvert\overline{\Delta E}\rvert$	MD	MAD	NMAD	RMSD	Δ_{err}	max	min
ALKBDE10	100.69	2.30	5.16	0.05	6.14	10.72	10.71	0.01
RC21	35.70	0.48	2.73	0.08	3.15	8.67	7.85	0.82
ALK8	62.60	5.83	5.83	0.09	8.15	15.20	14.46	0.74
DC13	54.98	-0.95	5.76	0.10	7.64	13.25	13.25	0.00
G2RC	51.26	-2.09	3.52	0.07	4.55	12.07	12.07	0.00
BH76RC	21.39	-0.41	1.56	0.07	1.97	5.62	5.62	0.00
MOR23	35.57	-10.81	12.77	0.36	14.90	25.63	25.61	0.02
WCPT18	34.99	3.86	3.86	0.11	4.37	7.51	7.46	0.05
BHROT27	6.37	0.24	0.26	0.04	0.34	1.02	1.01	0.01
BHPERI	20.87	-6.19	6.23	0.30	6.81	12.97	12.43	0.54
BHDIV10	45.33	-2.08	2.87	0.06	3.13	6.86	5.62	1.24
INV24	32.85	-0.31	1.66	0.05	2.11	4.47	4.45	0.02
CR20	19.31	0.57	0.86	0.04	1.04	2.32	2.28	0.04
CRBH20	46.13	3.15	3.15	0.07	3.20	6.45	4.47	1.98
TMBH17	12.76	-1.24	3.67	0.29	4.38	11.25	10.17	1.08
LTMBH26	9.98	-1.00	1.52	0.15	1.92	3.94	3.93	0.01
BH76	18.61	-1.31	2.46	0.13	3.07	8.74	8.59	0.15
ISO34	14.57	1.09	1.09	0.07	1.42	4.37	4.36	0.01
ICONF	3.27	0.25	0.34	0.10	0.42	0.72	0.72	0.00
ACONF	1.83	-0.47	0.47	0.26	0.52	1.05	0.92	0.13
TAUT15	3.05	0.83	0.83	0.27	1.14	2.13	2.11	0.02
Amino20x4	2.44	0.10	0.45	0.19	0.56	1.59	1.58	0.01
PCONF	1.62	0.64	0.64	0.39	0.79	1.79	1.77	0.02
MCONF	4.97	0.86	0.86	0.17	0.95	1.67	1.63	0.04
SCONF	4.60	1.02	1.02	0.22	1.12	1.95	1.95	0.00
PArel	4.63	0.28	1.12	0.24	1.50	4.17	4.16	0.01
BUT14DIOL	2.80	0.76	0.76	0.27	0.80	1.11	1.11	0.00
EIE22	5.44	0.61	0.65	0.12	0.74	1.67	1.61	0.06
Styrene45	62.64	0.84	2.22	0.04	2.72	6.84	6.84	0.00
ISOMERIZATION20	31.84	-0.79	2.14	0.07	2.76	7.14	7.06	0.08
DIE60	4.71	0.73	0.73	0.15	0.82	1.75	1.63	0.12
IDISP	14.22	6.96	6.96	0.49	11.15	27.23	26.22	1.01
C20C24	30.77	-8.29	8.78	0.29	10.57	15.79	15.76	0.03
S66	5.47	-1.26	1.26	0.23	1.38	3.57	3.31	0.26
S10x8	6.59	-0.11	0.22	0.03	0.36	1.67	1.67	0.00
X40	3.76	0.78	0.78	0.21	0.86	1.80	1.76	0.04
HEAVY28	1.24	0.73	0.74	0.59	0.81	1.91	1.77	0.14
CHB6	26.79	0.89	0.89	0.03	0.92	1.93	1.30	0.63
AHB21	22.49	-0.88	0.93	0.04	1.25	2.84	2.82	0.02
IL16	109.04	0.08	0.58	0.01	0.64	0.98	0.95	0.03
PNICO23	4.27	1.06	1.06	0.25	1.16	2.68	2.34	0.34
CT20	0.98	0.18	0.18	0.18	0.19	0.38	0.31	0.07
CARBHB12	6.04	1.71	1.71	0.28	1.89	3.58	3.06	0.52
ADIM6	3.36	2.64	2.64	0.78	2.91	5.51	4.58	0.93

Continued on next page

Test set	$\|\overline{\Delta E}\|$	MD	MAD	NMAD	RMSD	Δ_{err}	max	min
3B-69-TRIM	12.30	0.02	0.49	0.04	0.59	1.59	1.57	0.02

Table S29: Statistical analysis for DH-BLYP-D3(BJ) for all testset in our databsase. The numbers given (all in kcal/mol) are average reaction energy ($|\overline{\Delta E}|$), mean deviation (MD), mean absolute deviation (MAD), MAD normalized with respect to $|\overline{\Delta E}|$ (NMAD), root-mean-square deviation (RMSD), deviation span (Δ_{err}), maximum (max) and minimum deviation (min).

Test set	$\|\overline{\Delta E}\|$	MD	MAD	NMAD	RMSD	Δ_{err}	max	min
FH51	31.01	1.06	1.06	0.03	1.46	4.29	4.28	0.01
YBDE18	49.28	0.52	1.40	0.03	2.54	7.90	7.90	0.00
AL2X6	35.88	0.32	0.62	0.02	0.71	1.05	0.99	0.06
DARC	32.47	-0.23	0.75	0.02	0.94	1.63	1.62	0.01
NBPRC	27.71	-0.26	1.41	0.05	1.66	2.95	2.92	0.03
HEAVYSB9	58.02	0.89	1.97	0.03	2.33	3.81	3.70	0.11
BSR36	16.20	2.65	2.67	0.16	3.37	9.98	9.64	0.34
RSE43	7.60	0.30	0.51	0.07	0.96	4.42	4.41	0.01
W4-11	306.91	-0.62	2.74	0.01	3.40	10.49	10.44	0.05
G21EA	33.62	-5.81	5.82	0.17	6.12	10.64	10.47	0.17
G21IP	257.61	-1.32	2.38	0.01	2.86	8.36	8.36	0.00
DIPCS10	654.26	-3.74	3.74	0.01	4.33	8.41	8.02	0.39
PA26	189.05	0.34	1.30	0.01	1.87	5.33	5.31	0.02
SIE4x4	33.72	6.86	6.89	0.20	7.95	14.79	14.61	0.18
ALKBDE10	100.69	2.80	5.09	0.05	5.93	11.23	9.84	1.39
RC21	35.70	-0.62	1.42	0.04	1.85	4.87	4.87	0.00
ALK8	62.60	2.14	2.38	0.04	3.60	9.29	9.09	0.20
DC13	54.98	1.50	5.08	0.09	6.95	14.08	13.90	0.18
G2RC	51.26	-0.62	2.07	0.04	2.68	7.76	7.50	0.26
BH76RC	21.39	-0.34	1.15	0.05	1.53	4.97	4.88	0.09
MOR23	35.57	-6.67	8.31	0.23	10.07	21.03	20.66	0.37
WCPT18	34.99	3.13	3.13	0.09	3.74	6.62	6.55	0.07
BHROT27	6.37	0.20	0.20	0.03	0.30	0.98	0.98	0.00
BHPERI	20.87	3.54	3.54	0.17	3.78	8.59	7.19	1.40
BHDIV10	45.33	2.16	2.16	0.05	2.64	4.71	4.71	0.00
INV24	32.85	1.54	1.54	0.05	1.91	4.49	4.48	0.01
CR20	19.31	-4.46	4.46	0.23	4.51	9.23	5.81	3.42
CRBH20	46.13	-2.16	2.16	0.05	2.23	4.56	3.47	1.09
TMBH17	12.76	-0.94	3.21	0.25	3.86	7.47	7.38	0.09
LTMBH26	9.98	-1.55	2.06	0.21	2.61	6.86	6.72	0.14
BH76	18.61	-2.03	2.59	0.14	3.14	7.53	7.52	0.01
ISO34	14.57	0.93	0.93	0.06	1.45	6.10	6.09	0.01
ICONF	3.27	0.20	0.20	0.06	0.28	0.68	0.67	0.01
ACONF	1.83	0.28	0.28	0.16	0.32	0.60	0.53	0.07

Continued on next page

Test set	$\|\overline{\Delta E}\|$	MD	MAD	NMAD	RMSD	Δ_{err}	max	min
TAUT15	3.05	0.76	0.76	0.25	0.93	1.87	1.86	0.01
Amino20x4	2.44	0.30	0.30	0.12	0.38	1.09	1.09	0.00
PCONF	1.62	1.11	1.11	0.69	1.50	2.97	2.93	0.04
MCONF	4.97	1.26	1.26	0.25	1.41	2.32	2.27	0.05
SCONF	4.60	3.84	3.84	0.83	4.24	8.65	8.47	0.18
PArel	4.63	-0.03	0.80	0.17	1.14	2.75	2.73	0.02
BUT14DIOL	2.80	0.71	0.71	0.25	0.73	0.95	0.93	0.02
EIE22	5.44	0.69	0.70	0.13	0.80	1.76	1.74	0.02
Styrene45	62.64	2.85	3.20	0.05	4.10	11.56	11.56	0.00
ISOMERIZATION20	31.84	-0.52	1.95	0.06	2.54	6.33	6.28	0.05
DIE60	4.71	0.65	0.65	0.14	0.70	1.38	1.16	0.22
IDISP	14.22	6.65	6.65	0.47	8.86	20.79	19.04	1.75
C20C24	30.77	-0.76	23.14	0.75	25.52	48.99	39.11	9.88
S66	5.47	-0.21	0.21	0.04	0.25	0.55	0.54	0.01
S10x8	6.59	0.01	0.15	0.02	0.20	0.49	0.49	0.00
X40	3.76	0.17	0.17	0.05	0.21	0.57	0.57	0.00
HEAVY28	1.24	0.48	0.49	0.40	0.56	1.43	1.36	0.07
CHB6	26.79	0.72	0.72	0.03	0.88	1.53	1.46	0.07
AHB21	22.49	-0.61	0.70	0.03	0.92	2.13	2.11	0.02
IL16	109.04	0.52	0.67	0.01	0.78	1.54	1.54	0.00
PNICO23	4.27	0.47	0.47	0.11	0.51	1.00	0.85	0.15
CT20	0.98	0.12	0.13	0.13	0.14	0.29	0.25	0.04
CARBHB12	6.04	1.33	1.33	0.22	1.46	2.70	2.33	0.37
ADIM6	3.36	1.97	1.97	0.59	2.18	4.10	3.43	0.67
3B-69-TRIM	12.30	0.26	0.40	0.03	0.48	1.25	1.25	0.00

Table S30: Statistical analysis for PBE-QIDH-D3(BJ) for all testset in our databsase. The numbers given (all in kcal/mol) are average reaction energy ($|\overline{\Delta E}|$), mean deviation (MD), mean absolute deviation (MAD), MAD normalized with respect to $|\overline{\Delta E}|$ (NMAD), root-mean-square deviation (RMSD), deviation span (Δ_{err}), maximum (max) and minimum deviation (min).

Test set	$\|\overline{\Delta E}\|$	MD	MAD	NMAD	RMSD	Δ_{err}	max	min
FH51	31.01	-2.82	3.25	0.10	4.14	12.79	12.75	0.04
YBDE18	49.28	1.30	3.04	0.06	3.43	6.14	6.00	0.14
AL2X6	35.88	2.11	2.11	0.06	2.22	4.62	3.33	1.29
DARC	32.47	-8.73	8.73	0.27	8.81	17.77	10.28	7.49
NBPRC	27.71	-2.68	2.68	0.10	2.82	6.03	4.36	1.67
HEAVYSB9	58.02	0.33	1.24	0.02	1.54	2.74	2.67	0.07
BSR36	16.20	0.51	0.68	0.04	1.17	4.22	4.21	0.01
RSE43	7.60	0.46	0.54	0.07	0.90	3.87	3.86	0.01
W4-11	306.91	-6.62	6.74	0.02	8.18	23.50	23.48	0.02
G21EA	33.62	-5.59	5.59	0.17	6.68	13.03	12.87	0.16

Continued on next page

Test set	$\lvert\overline{\Delta E}\rvert$	MD	MAD	NMAD	RMSD	Δ_{err}	max	min
G21IP	257.61	-0.08	2.49	0.01	3.13	8.99	8.92	0.07
DIPCS10	654.26	-0.89	3.15	0.00	3.64	7.00	6.21	0.79
PA26	189.05	1.16	1.76	0.01	2.27	5.94	5.84	0.10
SIE4x4	33.72	2.80	2.83	0.08	3.57	8.43	8.22	0.21
ALKBDE10	100.69	42.10	42.10	0.42	47.73	91.07	75.08	15.99
RC21	35.70	2.52	3.24	0.09	4.42	12.99	12.54	0.45
ALK8	62.60	5.29	5.29	0.08	6.50	11.26	10.63	0.63
DC13	54.98	-2.56	8.06	0.15	9.45	17.36	17.32	0.04
G2RC	51.26	-4.59	5.81	0.11	7.22	19.35	18.69	0.66
BH76RC	21.39	-0.52	1.96	0.09	2.56	6.52	6.37	0.15
MOR23	35.57	-5.63	6.45	0.18	7.63	14.69	14.09	0.60
WCPT18	34.99	-2.42	2.75	0.08	3.46	6.08	6.06	0.02
BHROT27	6.37	0.44	0.44	0.07	0.59	1.25	1.25	0.00
BHPERI	20.87	-2.38	2.92	0.14	3.33	5.91	5.68	0.23
BHDIV10	45.33	-0.89	1.83	0.04	2.08	3.73	3.39	0.34
INV24	32.85	0.23	1.76	0.05	2.31	6.27	6.19	0.08
CR20	19.31	1.85	1.85	0.10	1.98	3.07	2.63	0.44
CRBH20	46.13	5.82	5.82	0.13	5.86	11.31	6.82	4.49
TMBH17	12.76	-9.05	14.38	1.13	18.65	51.33	51.02	0.31
LTMBH26	9.98	-0.29	1.72	0.17	2.29	8.45	8.33	0.12
BH76	18.61	-0.10	1.59	0.09	2.24	8.83	8.76	0.07
ISO34	14.57	0.21	1.21	0.08	1.79	3.87	3.86	0.01
ICONF	3.27	0.36	0.39	0.12	0.52	1.20	1.16	0.04
ACONF	1.83	-0.01	0.05	0.03	0.06	0.14	0.13	0.01
TAUT15	3.05	0.68	0.79	0.26	1.04	1.89	1.89	0.00
Amino20x4	2.44	0.10	0.24	0.10	0.30	0.78	0.78	0.00
PCONF	1.62	0.16	0.45	0.28	0.63	1.41	1.38	0.03
MCONF	4.97	0.53	0.68	0.14	0.76	1.28	1.25	0.03
SCONF	4.60	0.39	0.62	0.13	0.69	1.27	1.25	0.02
PArel	4.63	0.67	1.14	0.25	1.39	3.36	3.28	0.08
BUT14DIOL	2.80	0.47	0.47	0.17	0.48	0.55	0.55	0.00
EIE22	5.44	0.61	0.65	0.12	0.74	1.65	1.58	0.07
Styrene45	62.64	0.76	3.00	0.05	3.79	8.40	8.40	0.00
ISOMERIZATION20	31.84	-0.93	1.88	0.06	2.59	7.69	7.69	0.00
DIE60	4.71	0.79	0.80	0.17	0.98	2.16	2.15	0.01
IDISP	14.22	-3.29	4.94	0.35	9.47	23.40	22.87	0.53
C20C24	30.77	-8.13	8.13	0.26	8.79	16.86	13.45	3.41
S66	5.47	-0.57	0.59	0.11	0.71	1.94	1.91	0.03
S10x8	6.59	0.09	0.25	0.04	0.38	1.43	1.43	0.00
X40	3.76	0.31	0.40	0.11	0.44	0.98	0.94	0.04
HEAVY28	1.24	0.25	0.31	0.25	0.40	1.29	1.28	0.01
CHB6	26.79	-1.38	1.38	0.05	1.64	3.30	2.97	0.33
AHB21	22.49	-3.39	3.39	0.15	4.11	10.04	9.10	0.94
IL16	109.04	-1.71	1.71	0.02	1.83	3.44	2.68	0.76

Continued on next page

Test set	$\lvert\overline{\Delta E}\rvert$	MD	MAD	NMAD	RMSD	Δ_{err}	max	min
PNICO23	4.27	0.49	0.49	0.12	0.61	1.67	1.66	0.01
CT20	0.98	-0.07	0.07	0.08	0.10	0.18	0.18	0.00
CARBHB12	6.04	1.41	1.41	0.23	1.58	2.96	2.60	0.36
ADIM6	3.36	0.65	0.65	0.19	0.73	1.43	1.24	0.19
3B-69-TRIM	12.30	1.02	1.11	0.09	1.34	3.90	3.89	0.01

Table S31: Statistical analysis for SOS1-PBE-QIDH-D3(BJ) for all testset in our databsase. The numbers given (all in kcal/mol) are average reaction energy ($\lvert\overline{\Delta E}\rvert$), mean deviation (MD), mean absolute deviation (MAD), MAD normalized with respect to $\lvert\overline{\Delta E}\rvert$ (NMAD), root-mean-square deviation (RMSD), deviation span (Δ_{err}), maximum (max) and minimum deviation (min).

Test set	$\lvert\overline{\Delta E}\rvert$	MD	MAD	NMAD	RMSD	Δ_{err}	max	min
FH51	31.01	-2.31	2.84	0.09	3.84	14.79	14.77	0.02
YBDE18	49.28	0.54	2.30	0.05	2.51	4.59	4.40	0.19
AL2X6	35.88	2.51	2.51	0.07	2.62	5.20	3.73	1.47
DARC	32.47	-8.26	8.26	0.25	8.27	16.94	9.04	7.90
NBPRC	27.71	-2.47	2.47	0.09	2.62	5.82	4.17	1.65
HEAVYSB9	58.02	0.72	1.87	0.03	2.16	3.82	3.55	0.27
BSR36	16.20	-0.88	0.89	0.05	0.96	1.74	1.59	0.15
RSE43	7.60	0.86	0.87	0.11	1.19	4.32	4.29	0.03
W4-11	306.91	-8.01	8.07	0.03	9.91	28.55	28.52	0.03
G21EA	33.62	-6.23	6.23	0.19	7.13	13.92	12.74	1.18
G21IP	257.61	-0.41	2.21	0.01	2.79	6.75	6.73	0.02
DIPCS10	654.26	-1.42	3.16	0.00	3.78	7.30	7.29	0.01
PA26	189.05	2.56	2.80	0.01	3.23	7.63	7.01	0.62
SIE4x4	33.72	2.34	2.48	0.07	3.56	9.28	9.27	0.01
ALKBDE10	100.69	39.81	39.81	0.40	45.29	85.44	71.14	14.30
RC21	35.70	1.89	2.39	0.07	3.40	9.77	9.48	0.29
ALK8	62.60	6.33	6.33	0.10	7.89	14.08	13.66	0.42
DC13	54.98	-3.78	6.18	0.11	7.62	15.79	15.07	0.72
G2RC	51.26	-3.73	4.68	0.09	6.06	15.11	14.77	0.34
BH76RC	21.39	-0.38	1.89	0.09	2.39	5.67	5.66	0.01
MOR23	35.57	-6.46	6.58	0.19	7.86	18.44	18.40	0.04
WCPT18	34.99	-0.63	1.97	0.06	2.28	3.96	3.86	0.10
BHROT27	6.37	0.28	0.31	0.05	0.40	0.74	0.74	0.00
BHPERI	20.87	0.20	1.72	0.08	2.11	6.74	6.32	0.42
BHDIV10	45.33	0.28	1.25	0.03	1.31	2.87	2.02	0.85
INV24	32.85	0.78	1.84	0.06	2.79	9.07	9.02	0.05
CR20	19.31	1.82	1.82	0.09	1.91	3.24	2.70	0.54
CRBH20	46.13	5.63	5.63	0.12	5.69	11.41	6.93	4.48
TMBH17	12.76	-1.46	9.37	0.73	15.22	41.17	41.16	0.01
LTMBH26	9.98	0.27	1.48	0.15	2.44	10.56	10.53	0.03

Continued on next page

Test set	$\|\overline{\Delta E}\|$	MD	MAD	NMAD	RMSD	Δ_{err}	max	min
BH76	18.61	0.38	1.45	0.08	2.27	8.95	8.95	0.00
ISO34	14.57	-0.07	0.95	0.07	1.41	3.47	3.46	0.01
ICONF	3.27	0.23	0.30	0.09	0.41	0.95	0.95	0.00
ACONF	1.83	-0.05	0.06	0.03	0.07	0.17	0.16	0.01
TAUT15	3.05	0.14	0.57	0.19	0.73	1.92	1.90	0.02
Amino20x4	2.44	0.04	0.17	0.07	0.22	0.56	0.56	0.00
PCONF	1.62	0.22	0.52	0.32	0.71	1.58	1.56	0.02
MCONF	4.97	0.44	0.65	0.13	0.69	1.10	1.01	0.09
SCONF	4.60	0.15	0.35	0.08	0.44	1.02	1.01	0.01
PArel	4.63	0.44	0.79	0.17	1.06	2.63	2.60	0.03
BUT14DIOL	2.80	0.34	0.34	0.12	0.35	0.44	0.44	0.00
EIE22	5.44	0.30	0.33	0.06	0.39	1.00	0.99	0.01
Styrene45	62.64	-0.35	2.25	0.04	2.97	7.75	7.75	0.00
ISOMERIZATION20	31.84	-0.23	1.39	0.04	2.05	6.63	6.48	0.15
DIE60	4.71	0.46	0.51	0.11	0.78	1.88	1.88	0.00
IDISP	14.22	-2.55	3.99	0.28	7.97	19.25	19.22	0.03
C20C24	30.77	-6.85	6.85	0.22	7.96	15.98	13.24	2.74
S66	5.47	-0.64	0.64	0.12	0.72	1.89	1.87	0.02
S10x8	6.59	0.25	0.27	0.04	0.39	1.50	1.50	0.00
X40	3.76	0.37	0.38	0.10	0.42	0.97	0.94	0.03
HEAVY28	1.24	0.30	0.35	0.28	0.42	1.16	1.12	0.04
CHB6	26.79	-1.78	1.78	0.07	2.03	3.96	3.40	0.56
AHB21	22.49	-2.96	2.96	0.13	3.64	9.18	8.41	0.77
IL16	109.04	-1.32	1.32	0.01	1.43	2.57	2.14	0.43
PNICO23	4.27	0.47	0.47	0.11	0.53	1.03	0.97	0.06
CT20	0.98	0.07	0.10	0.10	0.11	0.22	0.18	0.04
CARBHB12	6.04	1.17	1.17	0.19	1.27	2.41	2.03	0.38
ADIM6	3.36	0.84	0.84	0.25	0.92	1.78	1.46	0.32
3B-69-TRIM	12.30	1.23	1.24	0.10	1.40	3.82	3.66	0.16
ISOL24	21.92	3.88	6.44	0.29	10.05	35.55	35.52	0.03
C60ISO	98.25	4.44	4.44	0.05	4.92	9.82	8.64	1.18
L7	18.20	-10.63	10.63	0.58	11.19	18.91	15.19	3.72
UPU23	5.72	-0.82	1.51	0.26	2.20	7.25	7.23	0.02
ENZYMES23	15.32	1.06	1.89	0.12	2.40	7.45	7.26	0.19

Table S32: Statistical analysis for DSD-BLYP for all testset in our databsase. The numbers given (all in kcal/mol) are average reaction energy ($|\overline{\Delta E}|$), mean deviation (MD), mean absolute deviation (MAD), MAD normalized with respect to $|\overline{\Delta E}|$ (NMAD), root-mean-square deviation (RMSD), deviation span (Δ_{err}), maximum (max) and minimum deviation (min).

Test set	$\|\overline{\Delta E}\|$	MD	MAD	NMAD	RMSD	Δ_{err}	max	min
FH51	31.01	1.02	1.02	0.03	1.35	3.36	3.36	0.00

Continued on next page

Test set	$\lvert\overline{\Delta E}\rvert$	MD	MAD	NMAD	RMSD	Δ_{err}	max	min
YBDE18	49.28	-0.14	1.54	0.03	2.19	6.55	6.49	0.06
AL2X6	35.88	1.17	1.17	0.03	1.39	2.39	2.31	0.08
DARC	32.47	-0.34	0.63	0.02	0.88	1.58	1.58	0.00
NBPRC	27.71	-0.03	1.49	0.05	1.73	2.88	2.80	0.08
HEAVYSB9	58.02	1.71	3.04	0.05	3.36	4.55	4.40	0.15
BSR36	16.20	1.82	1.85	0.11	2.47	7.66	7.60	0.06
RSE43	7.60	0.63	0.64	0.08	1.21	5.08	5.07	0.01
W4-11	306.91	-4.61	4.97	0.02	5.84	18.81	18.81	0.00
G21EA	33.62	-6.66	6.66	0.20	7.00	12.30	11.72	0.58
G21IP	257.61	-1.87	2.57	0.01	3.10	8.71	8.68	0.03
DIPCS10	654.26	-4.74	4.74	0.01	5.36	11.20	9.95	1.25
PA26	189.05	0.70	1.39	0.01	1.96	5.60	5.57	0.03
SIE4x4	33.72	5.27	5.32	0.16	6.24	12.68	12.31	0.37
ALKBDE10	100.69	0.64	3.64	0.04	4.43	8.33	7.73	0.60
RC21	35.70	-1.05	1.48	0.04	1.90	4.52	4.49	0.03
ALK8	62.60	3.30	3.37	0.05	5.04	12.88	12.57	0.31
DC13	54.98	0.90	4.53	0.08	6.58	14.29	14.21	0.08
G2RC	51.26	-0.82	1.76	0.03	2.23	6.99	6.92	0.07
BH76RC	21.39	-0.30	0.97	0.05	1.42	4.71	4.69	0.02
MOR23	35.57	-6.39	7.90	0.22	9.29	21.25	20.26	0.99
WCPT18	34.99	2.23	2.23	0.06	2.76	5.23	5.11	0.12
BHROT27	6.37	0.19	0.19	0.03	0.27	0.86	0.85	0.01
BHPERI	20.87	2.15	2.15	0.10	2.40	5.66	5.17	0.49
BHDIV10	45.33	1.56	1.56	0.03	1.94	3.95	3.73	0.22
INV24	32.85	1.65	1.65	0.05	1.95	3.52	3.42	0.10
CR20	19.31	-4.35	4.35	0.23	4.40	8.99	5.69	3.30
CRBH20	46.13	-1.08	1.09	0.02	1.23	2.50	2.38	0.12
TMBH17	12.76	-0.05	2.80	0.22	3.54	7.68	7.64	0.04
LTMBH26	9.98	-1.04	1.63	0.16	2.26	6.38	6.37	0.01
BH76	18.61	-1.00	1.90	0.10	2.52	8.48	8.46	0.02
ISO34	14.57	0.84	0.84	0.06	1.32	5.64	5.64	0.00
ICONF	3.27	0.22	0.22	0.07	0.29	0.67	0.66	0.01
ACONF	1.83	0.18	0.18	0.10	0.20	0.36	0.33	0.03
TAUT15	3.05	0.67	0.67	0.22	0.83	2.01	1.98	0.03
Amino20x4	2.44	0.22	0.22	0.09	0.28	0.80	0.80	0.00
PCONF	1.62	0.85	0.85	0.53	1.14	2.34	2.33	0.01
MCONF	4.97	0.97	0.97	0.20	1.07	1.73	1.70	0.03
SCONF	4.60	0.68	0.68	0.15	0.79	1.84	1.80	0.04
PArel	4.63	-0.00	0.67	0.14	0.91	2.10	2.07	0.03
BUT14DIOL	2.80	0.60	0.60	0.22	0.62	0.80	0.76	0.04
EIE22	5.44	0.57	0.58	0.11	0.66	1.38	1.37	0.01
Styrene45	62.64	2.76	3.07	0.05	3.88	10.57	10.57	0.00
ISOMERIZATION20	31.84	-0.48	1.69	0.05	2.33	6.91	6.91	0.00
DIE60	4.71	0.54	0.54	0.11	0.62	1.11	1.10	0.01

Continued on next page

Test set	$\lvert\overline{\Delta E}\rvert$	MD	MAD	NMAD	RMSD	Δ_{err}	max	min
IDISP	14.22	5.39	5.39	0.38	7.82	18.99	17.68	1.31
C20C24	30.77	0.00	23.09	0.75	25.04	48.86	37.59	11.27
S66	5.47	-0.56	0.56	0.10	0.62	1.40	1.37	0.03
S10x8	6.59	0.26	0.29	0.04	0.38	1.09	1.09	0.00
X40	3.76	0.31	0.31	0.08	0.33	0.65	0.62	0.03
HEAVY28	1.24	0.31	0.36	0.29	0.44	1.23	1.14	0.09
CHB6	26.79	0.34	0.35	0.01	0.44	0.86	0.84	0.02
AHB21	22.49	-0.40	0.65	0.03	0.84	2.00	1.98	0.02
IL16	109.04	1.09	1.09	0.01	1.24	2.30	2.14	0.16
PNICO23	4.27	0.30	0.31	0.07	0.35	0.70	0.66	0.04
CT20	0.98	0.20	0.20	0.21	0.21	0.40	0.33	0.07
CARBHB12	6.04	1.11	1.11	0.18	1.21	2.11	1.82	0.29
ADIM6	3.36	1.45	1.45	0.43	1.61	3.05	2.56	0.49
3B-69-TRIM	12.30	0.99	1.01	0.08	1.11	3.06	2.72	0.34

Table S33: Statistical analysis for revDSD-BLYP-D3(BJ) for all testset in our databsase. The numbers given (all in kcal/mol) are average reaction energy ($\lvert\overline{\Delta E}\rvert$), mean deviation (MD), mean absolute deviation (MAD), MAD normalized with respect to $\lvert\overline{\Delta E}\rvert$ (NMAD), root-mean-square deviation (RMSD), deviation span (Δ_{err}), maximum (max) and minimum deviation (min).

Test set	$\lvert\overline{\Delta E}\rvert$	MD	MAD	NMAD	RMSD	Δ_{err}	max	min
FH51	31.01	1.01	1.01	0.03	1.35	3.32	3.30	0.02
YBDE18	49.28	0.44	1.06	0.02	1.89	6.05	6.04	0.01
AL2X6	35.88	1.22	1.26	0.04	1.59	2.88	2.80	0.08
DARC	32.47	-1.40	1.44	0.04	1.62	2.85	2.57	0.28
NBPRC	27.71	-0.31	1.51	0.05	1.77	3.36	3.11	0.25
HEAVYSB9	58.02	1.01	2.13	0.04	2.44	4.26	4.05	0.21
BSR36	16.20	1.27	1.37	0.08	1.83	6.04	6.03	0.01
RSE43	7.60	0.97	0.97	0.13	1.53	6.25	6.01	0.24
W4-11	306.91	-3.99	4.33	0.01	5.25	15.70	15.61	0.09
G21EA	33.62	-6.87	6.87	0.20	7.23	13.13	11.43	1.70
G21IP	257.61	-1.85	2.61	0.01	3.06	7.44	7.29	0.15
DIPCS10	654.26	-4.62	4.62	0.01	5.44	10.69	10.36	0.33
PA26	189.05	1.59	1.88	0.01	2.47	6.24	6.22	0.02
SIE4x4	33.72	4.61	4.66	0.14	5.58	12.29	11.90	0.39
ALKBDE10	100.69	-0.06	2.93	0.03	3.74	8.14	7.94	0.20
RC21	35.70	-0.92	1.31	0.04	1.64	3.26	3.22	0.04
ALK8	62.60	2.85	3.08	0.05	5.71	15.76	15.56	0.20
DC13	54.98	-0.40	4.28	0.08	5.85	14.80	14.68	0.12
G2RC	51.26	-0.78	1.67	0.03	2.03	5.66	5.46	0.20
BH76RC	21.39	-0.22	1.04	0.05	1.43	4.51	4.50	0.01
MOR23	35.57	-5.08	6.28	0.18	7.61	18.36	18.34	0.02

Continued on next page

Test set	$\|\overline{\Delta E}\|$	MD	MAD	NMAD	RMSD	Δ_{err}	max	min
WCPT18	34.99	2.01	2.01	0.06	2.37	4.94	4.39	0.55
BHROT27	6.37	0.15	0.15	0.02	0.20	0.56	0.56	0.00
BHPERI	20.87	1.29	1.29	0.06	1.61	3.95	3.89	0.06
BHDIV10	45.33	1.15	1.15	0.03	1.54	2.95	2.93	0.02
INV24	32.85	1.62	1.62	0.05	1.97	4.70	4.42	0.28
CR20	19.31	-3.26	3.26	0.17	3.31	6.97	4.65	2.32
CRBH20	46.13	-0.24	0.66	0.01	0.79	1.55	1.51	0.04
TMBH17	12.76	0.61	2.63	0.21	3.51	7.65	7.64	0.01
LTMBH26	9.98	-0.78	1.43	0.14	2.10	6.82	6.80	0.02
BH76	18.61	-0.55	1.71	0.09	2.45	8.56	8.54	0.02
ISO34	14.57	0.76	0.76	0.05	1.10	4.11	4.11	0.00
ICONF	3.27	0.23	0.23	0.07	0.27	0.62	0.57	0.05
ACONF	1.83	0.21	0.21	0.12	0.24	0.43	0.39	0.04
TAUT15	3.05	0.70	0.70	0.23	0.90	2.37	2.32	0.05
Amino20x4	2.44	0.22	0.22	0.09	0.27	0.80	0.80	0.00
PCONF	1.62	0.96	0.96	0.59	1.27	2.42	2.38	0.04
MCONF	4.97	0.95	0.95	0.19	1.04	1.66	1.63	0.03
SCONF	4.60	3.28	3.28	0.71	3.60	6.92	6.82	0.10
PArel	4.63	-0.11	0.56	0.12	0.83	2.23	2.18	0.05
BUT14DIOL	2.80	0.58	0.58	0.21	0.59	0.77	0.74	0.03
EIE22	5.44	0.39	0.39	0.07	0.45	0.97	0.97	0.00
Styrene45	62.64	2.32	2.59	0.04	3.37	8.45	8.45	0.00
ISOMERIZATION20	31.84	-0.52	1.00	0.03	1.24	2.58	2.56	0.02
DIE60	4.71	0.32	0.36	0.08	0.46	1.03	1.01	0.02
IDISP	14.22	5.61	5.61	0.39	7.87	18.90	17.46	1.44
C20C24	30.77	1.14	21.39	0.70	22.66	44.86	32.52	12.34
S66	5.47	-0.56	0.56	0.10	0.62	1.54	1.51	0.03
S10x8	6.59	0.29	0.31	0.05	0.42	1.23	1.23	0.00
X40	3.76	0.41	0.41	0.11	0.45	0.71	0.70	0.01
HEAVY28	1.24	0.13	0.23	0.19	0.33	0.93	0.93	0.00
CHB6	26.79	0.62	0.71	0.03	1.05	2.30	2.25	0.05
AHB21	22.49	-0.26	0.57	0.03	0.71	1.72	1.72	0.00
IL16	109.04	1.20	1.20	0.01	1.31	2.73	2.33	0.40
PNICO23	4.27	0.24	0.29	0.07	0.33	0.64	0.60	0.04
CT20	0.98	0.20	0.20	0.20	0.21	0.41	0.34	0.07
CARBHB12	6.04	1.06	1.06	0.18	1.14	1.85	1.56	0.29
ADIM6	3.36	1.70	1.70	0.51	1.88	3.57	2.95	0.62
3B-69-TRIM	12.30	1.05	1.05	0.09	1.15	2.92	2.75	0.17

Table S34: Statistical analysis for revDOD-BLYP-D3(BJ) for all testset in our databsase. The numbers given (all in kcal/mol) are average reaction energy ($|\overline{\Delta E}|$), mean deviation (MD), mean absolute deviation (MAD), MAD normalized with respect to $|\overline{\Delta E}|$ (NMAD), root-mean-square deviation (RMSD), deviation span (Δ_{err}), maximum (max) and minimum deviation (min).

Test set	$\|\overline{\Delta E}\|$	MD	MAD	NMAD	RMSD	Δ_{err}	max	min
FH51	31.01	-0.51	1.35	0.04	1.78	6.21	6.20	0.01
YBDE18	49.28	1.36	1.48	0.03	2.24	6.42	6.29	0.13
AL2X6	35.88	1.75	1.75	0.05	2.11	3.78	3.61	0.17
DARC	32.47	-2.15	2.15	0.07	2.33	3.64	3.23	0.41
NBPRC	27.71	-0.52	1.75	0.06	2.06	3.88	3.44	0.44
HEAVYSB9	58.02	1.91	2.62	0.05	2.96	5.08	4.90	0.18
BSR36	16.20	0.55	0.90	0.06	1.13	3.85	3.84	0.01
RSE43	7.60	1.07	1.07	0.14	1.60	6.71	6.43	0.28
W4-11	306.91	0.34	3.62	0.01	4.71	13.79	13.72	0.07
G21EA	33.62	-6.08	6.08	0.18	6.61	11.80	11.22	0.58
G21IP	257.61	-0.82	2.36	0.01	2.84	6.15	5.96	0.19
DIPCS10	654.26	-2.53	3.48	0.01	4.14	9.27	8.60	0.67
PA26	189.05	2.34	2.56	0.01	3.10	6.90	6.77	0.13
SIE4x4	33.72	4.74	4.78	0.14	5.86	13.46	13.09	0.37
ALKBDE10	100.69	0.50	2.77	0.03	3.34	7.98	7.30	0.68
RC21	35.70	-0.50	1.00	0.03	1.19	2.22	2.18	0.04
ALK8	62.60	3.40	3.69	0.06	6.69	18.58	18.19	0.39
DC13	54.98	-1.78	4.13	0.08	5.77	16.06	15.29	0.77
G2RC	51.26	-0.76	1.79	0.03	2.11	4.40	4.35	0.05
BH76RC	21.39	-0.29	1.38	0.06	1.68	4.57	4.37	0.20
MOR23	35.57	-4.65	5.59	0.16	6.75	18.97	18.55	0.42
WCPT18	34.99	-0.58	1.90	0.05	2.24	3.90	3.80	0.10
BHROT27	6.37	0.02	0.14	0.02	0.17	0.33	0.33	0.00
BHPERI	20.87	-0.17	0.97	0.05	1.13	2.61	2.56	0.05
BHDIV10	45.33	0.80	1.13	0.02	1.43	2.71	2.63	0.08
INV24	32.85	0.54	1.56	0.05	2.07	5.79	5.68	0.11
CR20	19.31	-2.24	2.24	0.12	2.30	4.85	3.57	1.28
CRBH20	46.13	0.41	0.75	0.02	0.92	2.06	2.02	0.04
TMBH17	12.76	1.10	2.82	0.22	3.74	8.40	8.38	0.02
LTMBH26	9.98	-0.52	1.32	0.13	2.10	7.96	7.95	0.01
BH76	18.61	-0.50	1.85	0.10	2.54	8.24	8.15	0.09
ISO34	14.57	-0.01	0.71	0.05	1.00	2.74	2.73	0.01
ICONF	3.27	0.12	0.22	0.07	0.25	0.50	0.46	0.04
ACONF	1.83	-0.25	0.25	0.14	0.28	0.53	0.47	0.06
TAUT15	3.05	-0.19	0.92	0.30	1.11	2.63	2.56	0.07
Amino20x4	2.44	0.01	0.22	0.09	0.28	0.79	0.79	0.00
PCONF	1.62	1.12	1.12	0.69	1.43	2.60	2.51	0.09
MCONF	4.97	0.73	0.96	0.19	1.05	1.63	1.62	0.01

Continued on next page

Test set	$\lvert\overline{\Delta E}\rvert$	MD	MAD	NMAD	RMSD	Δ_{err}	max	min
SCONF	4.60	0.27	0.65	0.14	0.79	1.82	1.80	0.02
PArel	4.63	-0.20	0.56	0.12	0.84	2.18	2.18	0.00
BUT14DIOL	2.80	0.59	0.59	0.21	0.61	0.81	0.78	0.03
EIE22	5.44	0.19	0.24	0.04	0.29	0.64	0.61	0.03
Styrene45	62.64	1.57	2.79	0.04	3.55	9.03	9.03	0.00
ISOMERIZATION20	31.84	-0.09	0.84	0.03	1.25	4.00	3.96	0.04
DIE60	4.71	0.15	0.33	0.07	0.41	0.99	0.98	0.01
IDISP	14.22	3.76	3.76	0.26	5.67	13.44	12.86	0.58
C20C24	30.77	1.55	20.07	0.65	20.93	42.93	29.41	13.52
S66	5.47	-0.50	0.51	0.09	0.57	1.46	1.44	0.02
S10x8	6.59	0.26	0.30	0.05	0.40	1.20	1.20	0.00
X40	3.76	0.37	0.37	0.10	0.42	0.71	0.68	0.03
HEAVY28	1.24	0.17	0.26	0.21	0.34	0.90	0.89	0.01
CHB6	26.79	0.40	0.68	0.03	1.00	2.36	2.20	0.16
AHB21	22.49	-0.24	0.56	0.02	0.70	1.67	1.65	0.02
IL16	109.04	1.16	1.16	0.01	1.26	2.63	2.21	0.42
PNICO23	4.27	0.32	0.38	0.09	0.43	0.78	0.69	0.09
CT20	0.98	0.17	0.17	0.17	0.18	0.36	0.33	0.03
CARBHB12	6.04	1.08	1.08	0.18	1.16	1.97	1.63	0.34
ADIM6	3.36	1.92	1.92	0.57	2.11	4.01	3.27	0.74
3B-69-TRIM	12.30	0.94	0.94	0.08	1.04	2.62	2.50	0.12
ISOL24	21.92	0.89	1.57	0.07	2.55	9.39	9.38	0.01
C60ISO	98.25	-0.40	2.93	0.03	3.44	7.02	6.76	0.26
L7	18.20	0.88	1.02	0.06	1.32	2.64	2.58	0.06
UPU23	5.72	-0.05	0.96	0.17	1.25	3.71	3.70	0.01
ENZYMES23	15.32	-0.16	1.26	0.08	1.52	3.47	3.43	0.04

Table S35: Statistical analysis for DSD-PBEP86 for all testset in our databsase. The numbers given (all in kcal/mol) are average reaction energy ($\lvert\overline{\Delta E}\rvert$), mean deviation (MD), mean absolute deviation (MAD), MAD normalized with respect to $\lvert\overline{\Delta E}\rvert$ (NMAD), root-mean-square deviation (RMSD), deviation span (Δ_{err}), maximum (max) and minimum deviation (min).

Test set	$\lvert\overline{\Delta E}\rvert$	MD	MAD	NMAD	RMSD	Δ_{err}	max	min
FH51	31.01	1.08	1.08	0.03	1.47	4.14	4.14	0.00
YBDE18	49.28	-0.69	2.15	0.04	2.63	5.91	5.86	0.05
AL2X6	35.88	0.65	0.76	0.02	0.97	1.80	1.72	0.08
DARC	32.47	-2.53	2.53	0.08	2.64	4.90	3.66	1.24
NBPRC	27.71	-0.07	1.24	0.04	1.56	3.28	3.12	0.16
HEAVYSB9	58.02	-0.43	1.42	0.02	2.00	4.85	4.79	0.06
BSR36	16.20	0.97	1.04	0.06	1.53	5.41	5.37	0.04
RSE43	7.60	1.07	1.07	0.14	1.67	6.29	6.23	0.06
W4-11	306.91	-9.61	9.63	0.03	10.70	24.68	24.40	0.28

Continued on next page

Test set	$\lvert\overline{\Delta E}\rvert$	MD	MAD	NMAD	RMSD	Δ_{err}	max	min
G21EA	33.62	-7.25	7.25	0.22	7.72	15.49	13.22	2.27
G21IP	257.61	-2.39	3.04	0.01	3.51	8.93	8.78	0.15
DIPCS10	654.26	-5.65	5.65	0.01	6.00	12.34	9.96	2.38
PA26	189.05	1.56	1.72	0.01	2.28	6.00	5.99	0.01
SIE4x4	33.72	3.97	4.01	0.12	4.92	10.47	10.16	0.31
ALKBDE10	100.69	-2.58	3.91	0.04	4.72	11.08	9.77	1.31
RC21	35.70	-1.00	1.56	0.04	2.05	4.32	4.32	0.00
ALK8	62.60	3.22	3.22	0.05	4.61	11.34	11.13	0.21
DC13	54.98	0.08	3.34	0.06	4.33	10.98	10.81	0.17
G2RC	51.26	-0.83	1.85	0.04	2.24	4.80	4.62	0.18
BH76RC	21.39	0.04	1.31	0.06	1.68	4.83	4.78	0.05
MOR23	35.57	-4.57	5.97	0.17	7.40	15.86	15.19	0.67
WCPT18	34.99	2.01	2.01	0.06	2.47	4.92	4.79	0.13
BHROT27	6.37	0.14	0.14	0.02	0.19	0.54	0.54	0.00
BHPERI	20.87	1.59	1.59	0.08	1.93	4.35	4.32	0.03
BHDIV10	45.33	1.12	1.12	0.02	1.37	2.48	2.44	0.04
INV24	32.85	1.48	1.48	0.05	1.84	4.15	4.03	0.12
CR20	19.31	-2.84	2.84	0.15	2.91	6.43	4.41	2.02
CRBH20	46.13	0.57	0.73	0.02	0.94	2.01	2.00	0.01
TMBH17	12.76	0.39	2.22	0.17	2.83	6.09	6.07	0.02
LTMBH26	9.98	-0.66	1.37	0.14	1.98	6.89	6.68	0.21
BH76	18.61	-0.15	1.64	0.09	2.37	9.30	9.05	0.25
ISO34	14.57	0.47	0.47	0.03	0.74	2.67	2.67	0.00
ICONF	3.27	0.21	0.21	0.06	0.27	0.66	0.63	0.03
ACONF	1.83	0.12	0.12	0.06	0.13	0.24	0.23	0.01
TAUT15	3.05	0.68	0.68	0.22	0.89	2.35	2.32	0.03
Amino20x4	2.44	0.18	0.18	0.07	0.23	0.69	0.69	0.00
PCONF	1.62	0.72	0.72	0.45	0.96	1.97	1.95	0.02
MCONF	4.97	0.83	0.83	0.17	0.90	1.47	1.42	0.05
SCONF	4.60	0.41	0.41	0.09	0.51	1.23	1.19	0.04
PArel	4.63	0.04	0.61	0.13	0.85	1.96	1.94	0.02
BUT14DIOL	2.80	0.37	0.37	0.13	0.38	0.50	0.49	0.01
EIE22	5.44	0.41	0.43	0.08	0.49	1.11	1.09	0.02
Styrene45	62.64	0.63	1.17	0.02	1.46	3.76	3.76	0.00
ISOMERIZATION20	31.84	-0.25	1.40	0.04	1.79	4.40	4.39	0.01
DIE60	4.71	0.48	0.48	0.10	0.62	1.27	1.25	0.02
IDISP	14.22	5.01	5.01	0.35	7.91	19.38	18.45	0.93
C20C24	30.77	-3.71	14.38	0.47	17.38	33.87	29.97	3.90
S66	5.47	-0.80	0.80	0.15	0.88	2.26	2.09	0.17
S10x8	6.59	0.51	0.51	0.08	0.72	1.71	1.71	0.00
X40	3.76	0.53	0.53	0.14	0.56	1.13	1.00	0.13
HEAVY28	1.24	0.17	0.25	0.20	0.32	1.01	0.98	0.03
CHB6	26.79	1.12	1.12	0.04	1.24	2.77	2.13	0.64
AHB21	22.49	-0.05	0.53	0.02	0.66	1.50	1.46	0.04

Continued on next page

Test set	$\|\overline{\Delta E}\|$	MD	MAD	NMAD	RMSD	Δ_{err}	max	min
IL16	109.04	1.55	1.55	0.01	1.68	3.06	2.58	0.48
PNICO23	4.27	0.20	0.22	0.05	0.28	0.71	0.69	0.02
CT20	0.98	0.26	0.26	0.27	0.28	0.61	0.48	0.13
CARBHB12	6.04	0.99	0.99	0.16	1.07	1.90	1.56	0.34
ADIM6	3.36	1.46	1.46	0.43	1.59	3.04	2.45	0.59
3B-69-TRIM	12.30	1.54	1.54	0.13	1.67	3.77	3.46	0.31

Table S36: Statistical analysis for revDSD-PBEP86-D3(BJ) for all testset in our databsase. The numbers given (all in kcal/mol) are average reaction energy ($|\overline{\Delta E}|$), mean deviation (MD), mean absolute deviation (MAD), MAD normalized with respect to $|\overline{\Delta E}|$ (NMAD), root-mean-square deviation (RMSD), deviation span (Δ_{err}), maximum (max) and minimum deviation (min).

Test set	$\|\overline{\Delta E}\|$	MD	MAD	NMAD	RMSD	Δ_{err}	max	min
FH51	31.01	1.08	1.08	0.03	1.44	4.69	4.67	0.02
YBDE18	49.28	-0.90	1.76	0.04	2.33	5.13	5.11	0.02
AL2X6	35.88	0.54	0.77	0.02	0.92	1.75	1.50	0.25
DARC	32.47	-1.99	1.99	0.06	2.09	3.78	2.96	0.82
NBPRC	27.71	0.11	1.35	0.05	1.68	3.84	3.71	0.13
HEAVYSB9	58.02	-0.61	1.39	0.02	2.01	4.97	4.91	0.06
BSR36	16.20	-0.19	0.42	0.03	0.60	2.20	2.20	0.00
RSE43	7.60	1.24	1.24	0.16	1.84	7.16	6.98	0.18
W4-11	306.91	-9.92	9.92	0.03	10.95	24.22	23.76	0.46
G21EA	33.62	-7.54	7.54	0.22	7.98	15.98	13.08	2.90
G21IP	257.61	-2.60	3.22	0.01	3.61	8.05	7.85	0.20
DIPCS10	654.26	-6.05	6.05	0.01	6.44	13.21	10.68	2.53
PA26	189.05	2.09	2.20	0.01	2.71	6.42	6.40	0.02
SIE4x4	33.72	3.85	3.88	0.12	4.87	10.72	10.67	0.05
ALKBDE10	100.69	-3.32	3.80	0.04	4.85	10.88	10.53	0.35
RC21	35.70	-1.50	1.84	0.05	2.16	4.08	3.78	0.30
ALK8	62.60	2.57	2.57	0.04	3.99	10.63	10.36	0.27
DC13	54.98	-0.11	3.25	0.06	4.54	10.24	9.93	0.31
G2RC	51.26	-0.32	1.46	0.03	1.79	4.05	3.99	0.06
BH76RC	21.39	0.13	1.24	0.06	1.60	4.49	4.44	0.05
MOR23	35.57	-3.07	4.37	0.12	5.69	12.80	12.30	0.50
WCPT18	34.99	1.63	1.63	0.05	1.98	4.22	4.02	0.20
BHROT27	6.37	0.14	0.14	0.02	0.16	0.31	0.29	0.02
BHPERI	20.87	0.83	0.83	0.04	1.06	2.63	2.63	0.00
BHDIV10	45.33	0.92	0.92	0.02	1.10	2.18	1.94	0.24
INV24	32.85	1.44	1.44	0.04	1.87	4.99	4.88	0.11
CR20	19.31	-2.88	2.88	0.15	2.94	6.46	4.41	2.05
CRBH20	46.13	0.47	0.78	0.02	0.96	2.23	2.11	0.12
TMBH17	12.76	0.94	2.34	0.18	3.07	6.74	6.50	0.24

Continued on next page

Test set	$\vert\overline{\Delta E}\vert$	MD	MAD	NMAD	RMSD	Δ_{err}	max	min
LTMBH26	9.98	-0.46	1.31	0.13	2.01	7.48	7.41	0.07
BH76	18.61	0.09	1.53	0.08	2.38	9.08	9.06	0.02
ISO34	14.57	0.45	0.45	0.03	0.67	1.97	1.95	0.02
ICONF	3.27	0.18	0.18	0.06	0.23	0.53	0.51	0.02
ACONF	1.83	0.09	0.09	0.05	0.10	0.16	0.16	0.00
TAUT15	3.05	0.91	0.91	0.30	1.08	2.69	2.60	0.09
Amino20x4	2.44	0.15	0.15	0.06	0.20	0.56	0.56	0.00
PCONF	1.62	0.57	0.57	0.35	0.75	1.59	1.57	0.02
MCONF	4.97	0.68	0.68	0.14	0.73	1.24	1.14	0.10
SCONF	4.60	3.28	3.28	0.71	3.60	6.92	6.82	0.10
PArel	4.63	-0.08	0.56	0.12	0.79	1.80	1.80	0.00
BUT14DIOL	2.80	0.29	0.29	0.10	0.29	0.41	0.39	0.02
EIE22	5.44	0.30	0.32	0.06	0.37	0.91	0.86	0.05
Styrene45	62.64	0.16	1.03	0.02	1.26	2.95	2.95	0.00
ISOMERIZATION20	31.84	0.09	1.22	0.04	1.75	5.78	5.78	0.00
DIE60	4.71	0.33	0.38	0.08	0.54	1.21	1.21	0.00
IDISP	14.22	4.11	4.11	0.29	6.77	16.47	15.93	0.54
C20C24	30.77	-2.75	15.61	0.51	17.99	36.73	30.19	6.54
S66	5.47	-1.03	1.03	0.19	1.14	2.96	2.69	0.27
S10x8	6.59	0.75	0.76	0.11	1.03	2.56	2.55	0.01
X40	3.76	0.70	0.70	0.19	0.76	1.67	1.51	0.16
HEAVY28	1.24	0.08	0.19	0.15	0.28	0.82	0.81	0.01
CHB6	26.79	1.23	1.23	0.05	1.43	3.22	2.64	0.58
AHB21	22.49	0.29	0.53	0.02	0.65	1.34	1.33	0.01
IL16	109.04	2.06	2.06	0.02	2.16	4.12	3.13	0.99
PNICO23	4.27	-0.03	0.21	0.05	0.27	0.62	0.61	0.01
CT20	0.98	0.33	0.33	0.34	0.35	0.81	0.64	0.17
CARBHB12	6.04	0.78	0.78	0.13	0.84	1.50	1.22	0.28
ADIM6	3.36	1.17	1.17	0.35	1.27	2.43	1.93	0.50
3B-69-TRIM	12.30	2.16	2.16	0.18	2.31	4.95	4.45	0.50

Table S37: Statistical analysis for revDOD-PBEP86-D3(BJ) for all testset in our databsase. The numbers given (all in kcal/mol) are average reaction energy ($\vert\overline{\Delta E}\vert$), mean deviation (MD), mean absolute deviation (MAD), MAD normalized with respect to $\vert\overline{\Delta E}\vert$ (NMAD), root-mean-square deviation (RMSD), deviation span (Δ_{err}), maximum (max) and minimum deviation (min).

Test set	$\vert\overline{\Delta E}\vert$	MD	MAD	NMAD	RMSD	Δ_{err}	max	min
FH51	31.01	-0.19	1.19	0.04	1.60	5.43	5.42	0.01
YBDE18	49.28	-1.14	1.81	0.04	2.22	4.53	4.42	0.11
AL2X6	35.88	0.17	0.70	0.02	0.75	1.26	1.06	0.20
DARC	32.47	-1.72	1.72	0.05	1.83	3.13	2.61	0.52
NBPRC	27.71	0.31	1.55	0.06	1.86	4.83	4.34	0.49

Continued on next page

Test set	$\lvert\overline{\Delta E}\rvert$	MD	MAD	NMAD	RMSD	Δ_{err}	max	min
HEAVYSB9	58.02	-0.84	1.54	0.03	2.14	5.32	5.21	0.11
BSR36	16.20	-0.53	0.60	0.04	0.69	1.48	1.40	0.08
RSE43	7.60	1.30	1.30	0.17	1.89	7.46	7.23	0.23
W4-11	306.91	-9.12	9.12	0.03	10.06	22.05	21.50	0.55
G21EA	33.62	-7.38	7.38	0.22	7.83	15.28	12.64	2.64
G21IP	257.61	-2.34	3.06	0.01	3.41	7.11	6.99	0.12
DIPCS10	654.26	-5.51	5.51	0.01	6.00	12.09	10.31	1.78
PA26	189.05	2.44	2.54	0.01	3.02	6.93	6.66	0.27
SIE4x4	33.72	3.78	3.83	0.11	4.85	11.05	10.99	0.06
ALKBDE10	100.69	-3.48	3.73	0.04	4.78	10.74	10.54	0.20
RC21	35.70	-1.42	1.77	0.05	2.03	3.89	3.53	0.36
ALK8	62.60	2.78	2.78	0.04	4.32	11.56	11.26	0.30
DC13	54.98	-0.60	2.96	0.05	4.35	9.98	9.87	0.11
G2RC	51.26	-0.27	1.51	0.03	1.84	4.38	4.30	0.08
BH76RC	21.39	0.12	1.26	0.06	1.60	4.38	4.36	0.02
MOR23	35.57	-2.82	4.01	0.11	5.17	12.49	12.10	0.39
WCPT18	34.99	-0.76	1.40	0.04	1.70	3.55	3.44	0.11
BHROT27	6.37	-0.02	0.15	0.02	0.17	0.32	0.32	0.00
BHPERI	20.87	0.36	0.74	0.04	0.92	1.82	1.79	0.03
BHDIV10	45.33	0.55	0.96	0.02	1.11	1.81	1.76	0.05
INV24	32.85	0.19	1.46	0.04	1.95	5.53	5.50	0.03
CR20	19.31	-3.00	3.00	0.16	3.05	6.60	4.44	2.16
CRBH20	46.13	0.57	0.78	0.02	1.00	2.30	2.26	0.04
TMBH17	12.76	1.19	2.45	0.19	3.22	7.06	6.83	0.23
LTMBH26	9.98	-0.35	1.26	0.13	2.02	7.94	7.92	0.02
BH76	18.61	0.27	1.53	0.08	2.39	9.13	9.10	0.03
ISO34	14.57	-0.25	0.47	0.03	0.69	2.28	2.27	0.01
ICONF	3.27	0.11	0.16	0.05	0.20	0.46	0.46	0.00
ACONF	1.83	-0.06	0.06	0.04	0.07	0.14	0.13	0.01
TAUT15	3.05	-0.25	1.04	0.34	1.21	2.78	2.69	0.09
Amino20x4	2.44	-0.06	0.16	0.06	0.20	0.52	0.52	0.00
PCONF	1.62	0.60	0.60	0.37	0.78	1.59	1.59	0.00
MCONF	4.97	0.37	0.62	0.13	0.67	1.18	1.06	0.12
SCONF	4.60	0.05	0.28	0.06	0.35	0.90	0.86	0.04
PArel	4.63	-0.12	0.56	0.12	0.78	1.69	1.67	0.02
BUT14DIOL	2.80	0.27	0.27	0.10	0.27	0.40	0.38	0.02
EIE22	5.44	0.28	0.30	0.06	0.36	0.84	0.83	0.01
Styrene45	62.64	-0.83	1.24	0.02	1.64	4.42	4.42	0.00
ISOMERIZATION20	31.84	0.33	1.22	0.04	2.11	8.13	8.11	0.02
DIE60	4.71	0.27	0.38	0.08	0.53	1.25	1.24	0.01
IDISP	14.22	5.64	5.64	0.40	8.74	21.50	20.26	1.24
C20C24	30.77	-2.84	16.16	0.53	18.52	38.99	31.23	7.76
S66	5.47	-1.13	1.13	0.21	1.25	3.20	2.91	0.29
S10x8	6.59	0.77	0.77	0.12	1.06	2.63	2.62	0.01

Continued on next page

Test set	$\|\overline{\Delta E}\|$	MD	MAD	NMAD	RMSD	Δ_{err}	max	min
X40	3.76	0.77	0.77	0.20	0.83	1.84	1.66	0.18
HEAVY28	1.24	0.05	0.18	0.15	0.27	0.74	0.74	0.00
CHB6	26.79	1.23	1.23	0.05	1.40	3.16	2.51	0.65
AHB21	22.49	0.35	0.56	0.02	0.67	1.37	1.35	0.02
IL16	109.04	2.11	2.11	0.02	2.20	4.23	3.15	1.08
PNICO23	4.27	-0.03	0.21	0.05	0.29	0.74	0.73	0.01
CT20	0.98	0.34	0.34	0.34	0.36	0.84	0.67	0.17
CARBHB12	6.04	0.75	0.75	0.12	0.81	1.44	1.15	0.29
ADIM6	3.36	1.21	1.21	0.36	1.31	2.51	1.99	0.52
3B-69-TRIM	12.30	2.19	2.19	0.18	2.33	4.92	4.43	0.49
ISOL24	21.92	0.25	0.98	0.04	1.31	4.50	4.39	0.11
C60ISO	98.25	-1.90	3.34	0.03	4.35	9.94	9.11	0.83
L7	18.20	3.74	3.74	0.21	3.94	7.89	5.61	2.28
UPU23	5.72	0.10	0.69	0.12	0.88	2.25	2.24	0.01
ENZYMES23	15.32	-0.38	1.20	0.08	1.72	5.61	5.56	0.05

Table S38: Statistical analysis for DSD-PBE for all testset in our databsase. The numbers given (all in kcal/mol) are average reaction energy ($|\overline{\Delta E}|$), mean deviation (MD), mean absolute deviation (MAD), MAD normalized with respect to $|\overline{\Delta E}|$ (NMAD), root-mean-square deviation (RMSD), deviation span (Δ_{err}), maximum (max) and minimum deviation (min).

Test set	$\|\overline{\Delta E}\|$	MD	MAD	NMAD	RMSD	Δ_{err}	max	min
FH51	31.01	1.69	1.69	0.05	2.30	8.18	8.17	0.01
YBDE18	49.28	1.35	2.00	0.04	2.54	6.97	6.91	0.06
AL2X6	35.88	2.33	2.33	0.06	2.57	5.31	4.32	0.99
DARC	32.47	-5.69	5.69	0.18	5.75	11.24	7.05	4.19
NBPRC	27.71	-0.98	2.14	0.08	2.40	4.84	4.21	0.63
HEAVYSB9	58.02	1.73	2.35	0.04	2.67	4.46	4.40	0.06
BSR36	16.20	1.92	1.96	0.12	2.58	7.91	7.82	0.09
RSE43	7.60	1.01	1.01	0.13	1.60	6.12	6.09	0.03
W4-11	306.91	-4.52	4.59	0.01	5.38	17.81	17.72	0.09
G21EA	33.62	-6.89	6.89	0.20	7.29	15.36	12.33	3.03
G21IP	257.61	-1.99	2.66	0.01	3.08	8.06	7.96	0.10
DIPCS10	654.26	-4.90	4.90	0.01	5.19	10.47	8.44	2.03
PA26	189.05	1.74	1.87	0.01	2.36	5.98	5.90	0.08
SIE4x4	33.72	4.26	4.29	0.13	5.15	10.79	10.58	0.21
ALKBDE10	100.69	-1.24	3.35	0.03	4.05	8.57	8.16	0.41
RC21	35.70	0.24	1.86	0.05	2.22	6.71	6.04	0.67
ALK8	62.60	5.00	5.00	0.08	6.80	14.55	14.35	0.20
DC13	54.98	-1.53	2.79	0.05	4.02	8.28	8.16	0.12
G2RC	51.26	-1.40	2.66	0.05	3.32	8.39	8.11	0.28
BH76RC	21.39	-0.00	1.45	0.07	1.89	5.25	5.24	0.01

Continued on next page

Test set	$\|\overline{\Delta E}\|$	MD	MAD	NMAD	RMSD	Δ_{err}	max	min
MOR23	35.57	-6.53	7.69	0.22	9.06	20.07	19.88	0.19
WCPT18	34.99	2.67	2.67	0.08	3.26	6.21	5.97	0.24
BHROT27	6.37	0.14	0.14	0.02	0.19	0.51	0.50	0.01
BHPERI	20.87	2.64	2.64	0.13	3.06	6.79	6.19	0.60
BHDIV10	45.33	1.62	1.62	0.04	1.77	2.89	2.73	0.16
INV24	32.85	1.35	1.35	0.04	1.71	4.14	4.09	0.05
CR20	19.31	-0.27	0.53	0.03	0.70	1.97	1.90	0.07
CRBH20	46.13	2.22	2.22	0.05	2.33	4.40	3.47	0.93
TMBH17	12.76	-0.11	2.16	0.17	2.75	5.57	5.57	0.00
LTMBH26	9.98	-0.65	1.34	0.13	1.94	7.01	7.01	0.00
BH76	18.61	-0.55	1.83	0.10	2.46	8.51	8.33	0.18
ISO34	14.57	0.63	0.63	0.04	0.80	1.75	1.75	0.00
ICONF	3.27	0.22	0.22	0.07	0.29	0.62	0.62	0.00
ACONF	1.83	0.27	0.27	0.15	0.30	0.56	0.50	0.06
TAUT15	3.05	0.71	0.71	0.23	0.90	2.31	2.21	0.10
Amino20x4	2.44	0.27	0.27	0.11	0.34	1.03	1.03	0.00
PCONF	1.62	1.16	1.16	0.71	1.50	2.90	2.76	0.14
MCONF	4.97	1.20	1.20	0.24	1.33	2.15	2.05	0.10
SCONF	4.60	0.68	0.68	0.15	0.80	1.84	1.83	0.01
PArel	4.63	0.09	0.73	0.16	1.03	2.87	2.86	0.01
BUT14DIOL	2.80	0.50	0.50	0.18	0.52	0.71	0.69	0.02
EIE22	5.44	0.36	0.39	0.07	0.45	1.00	0.99	0.01
Styrene45	62.64	-0.24	0.98	0.02	1.33	3.44	3.44	0.00
ISOMERIZATION20	31.84	0.05	1.60	0.05	2.29	7.75	7.72	0.03
DIE60	4.71	0.44	0.44	0.09	0.61	1.37	1.37	0.00
IDISP	14.22	7.30	7.30	0.51	10.41	25.17	23.39	1.78
C20C24	30.77	-5.62	7.68	0.25	10.47	21.94	19.62	2.32
S66	5.47	-0.39	0.39	0.07	0.47	1.17	1.16	0.01
S10x8	6.59	0.23	0.28	0.04	0.41	1.02	1.02	0.00
X40	3.76	0.26	0.26	0.07	0.30	0.74	0.69	0.05
HEAVY28	1.24	0.40	0.42	0.34	0.49	1.32	1.23	0.09
CHB6	26.79	0.76	0.76	0.03	0.83	1.68	1.38	0.30
AHB21	22.49	-0.37	0.63	0.03	0.81	2.00	1.97	0.03
IL16	109.04	0.88	0.90	0.01	1.06	1.69	1.69	0.00
PNICO23	4.27	0.62	0.62	0.15	0.68	1.46	1.24	0.22
CT20	0.98	0.23	0.23	0.23	0.24	0.47	0.38	0.09
CARBHB12	6.04	1.28	1.28	0.21	1.37	2.53	2.08	0.45
ADIM6	3.36	2.05	2.05	0.61	2.24	4.25	3.47	0.78
3B-69-TRIM	12.30	0.78	0.80	0.07	0.92	2.13	2.11	0.02

Table S39: Statistical analysis for revDSD-PBE-D3(BJ) for all testset in our databsase. The numbers given (all in kcal/mol) are average reaction energy ($|\overline{\Delta E}|$), mean deviation (MD), mean absolute deviation (MAD), MAD normalized with respect to $|\overline{\Delta E}|$ (NMAD), root-mean-square deviation (RMSD), deviation span (Δ_{err}), maximum (max) and minimum deviation (min).

Test set	$\lvert\overline{\Delta E}\rvert$	MD	MAD	NMAD	RMSD	Δ_{err}	max	min
FH51	31.01	-0.75	1.48	0.05	1.97	7.16	7.14	0.02
YBDE18	49.28	0.79	1.79	0.04	2.21	7.17	6.52	0.65
AL2X6	35.88	1.88	1.88	0.05	2.11	4.32	3.63	0.69
DARC	32.47	-4.50	4.50	0.14	4.57	8.89	5.70	3.19
NBPRC	27.71	-0.67	1.75	0.06	2.02	3.96	3.77	0.19
HEAVYSB9	58.02	1.12	1.99	0.03	2.25	4.29	3.78	0.51
BSR36	16.20	1.22	1.29	0.08	1.79	6.01	5.92	0.09
RSE43	7.60	1.15	1.15	0.15	1.76	6.87	6.75	0.12
W4-11	306.91	-5.79	5.82	0.02	6.56	15.47	15.36	0.11
G21EA	33.62	-7.48	7.48	0.22	7.86	16.14	12.76	3.38
G21IP	257.61	-2.62	3.18	0.01	3.56	8.08	8.01	0.07
DIPCS10	654.26	-6.15	6.15	0.01	6.39	13.13	9.88	3.25
PA26	189.05	1.98	2.08	0.01	2.56	6.13	6.10	0.03
SIE4x4	33.72	4.24	4.26	0.13	5.17	11.00	10.80	0.20
ALKBDE10	100.69	-1.72	3.34	0.03	4.05	8.82	8.41	0.41
RC21	35.70	-0.33	1.56	0.04	1.95	4.76	4.71	0.05
ALK8	62.60	4.46	4.46	0.07	6.28	15.09	14.85	0.24
DC13	54.98	-1.34	2.71	0.05	3.32	6.98	6.57	0.41
G2RC	51.26	-0.83	2.23	0.04	2.69	5.85	5.85	0.00
BH76RC	21.39	0.13	1.43	0.07	1.82	5.03	4.95	0.08
MOR23	35.57	-5.41	6.56	0.18	7.89	17.77	17.34	0.43
WCPT18	34.99	-2.24	2.34	0.07	2.89	5.51	5.46	0.05
BHROT27	6.37	0.03	0.13	0.02	0.16	0.35	0.34	0.01
BHPERI	20.87	-1.79	1.99	0.10	2.37	5.01	4.90	0.11
BHDIV10	45.33	-0.71	1.32	0.03	1.48	2.44	2.33	0.11
INV24	32.85	0.14	1.35	0.04	1.73	4.43	4.29	0.14
CR20	19.31	-0.90	0.92	0.05	1.10	2.71	2.53	0.18
CRBH20	46.13	1.61	1.61	0.03	1.79	3.32	3.04	0.28
TMBH17	12.76	0.10	2.18	0.17	2.78	6.01	6.00	0.01
LTMBH26	9.98	-0.66	1.34	0.13	1.95	7.00	6.99	0.01
BH76	18.61	-0.40	1.76	0.09	2.45	8.93	8.92	0.01
ISO34	14.57	-0.15	0.54	0.04	0.70	1.79	1.78	0.01
ICONF	3.27	0.15	0.20	0.06	0.26	0.53	0.53	0.00
ACONF	1.83	-0.21	0.21	0.12	0.23	0.43	0.39	0.04
TAUT15	3.05	-0.02	0.79	0.26	1.00	2.46	2.42	0.04
Amino20x4	2.44	-0.01	0.22	0.09	0.28	0.86	0.86	0.00
PCONF	1.62	0.95	0.95	0.58	1.22	2.39	2.29	0.10
MCONF	4.97	0.74	0.97	0.20	1.06	1.63	1.61	0.02

Continued on next page

Test set	$\|\overline{\Delta E}\|$	MD	MAD	NMAD	RMSD	Δ_{err}	max	min
SCONF	4.60	0.26	0.59	0.13	0.70	1.59	1.59	0.00
PArel	4.63	-0.02	0.70	0.15	0.99	2.60	2.59	0.01
BUT14DIOL	2.80	0.43	0.43	0.15	0.44	0.61	0.60	0.01
EIE22	5.44	0.32	0.35	0.06	0.41	0.90	0.90	0.00
Styrene45	62.64	-0.50	1.04	0.02	1.39	3.36	3.36	0.00
ISOMERIZATION20	31.84	0.23	1.48	0.05	2.24	8.02	8.01	0.01
DIE60	4.71	0.36	0.38	0.08	0.54	1.19	1.19	0.00
IDISP	14.22	6.88	6.88	0.48	9.85	23.79	22.21	1.58
C20C24	30.77	-4.46	8.90	0.29	12.16	22.69	22.15	0.54
S66	5.47	-0.72	0.72	0.13	0.80	2.00	1.83	0.17
S10x8	6.59	0.47	0.47	0.07	0.66	1.69	1.69	0.00
X40	3.76	0.48	0.48	0.13	0.51	1.13	0.97	0.16
HEAVY28	1.24	0.23	0.29	0.24	0.36	1.05	1.03	0.02
CHB6	26.79	0.97	0.97	0.04	1.11	2.52	2.04	0.48
AHB21	22.49	-0.09	0.51	0.02	0.63	1.54	1.47	0.07
IL16	109.04	1.30	1.30	0.01	1.43	2.48	2.18	0.30
PNICO23	4.27	0.40	0.41	0.10	0.46	0.96	0.84	0.12
CT20	0.98	0.29	0.29	0.29	0.30	0.63	0.48	0.15
CARBHB12	6.04	1.09	1.09	0.18	1.17	2.07	1.70	0.37
ADIM6	3.36	1.68	1.68	0.50	1.83	3.50	2.83	0.67
3B-69-TRIM	12.30	1.47	1.47	0.12	1.58	3.27	3.08	0.19

Table S40: Statistical analysis for revDOD-PBE-D3(BJ) for all testset in our databsase. The numbers given (all in kcal/mol) are average reaction energy ($|\overline{\Delta E}|$), mean deviation (MD), mean absolute deviation (MAD), MAD normalized with respect to $|\overline{\Delta E}|$ (NMAD), root-mean-square deviation (RMSD), deviation span (Δ_{err}), maximum (max) and minimum deviation (min).

Test set	$\|\overline{\Delta E}\|$	MD	MAD	NMAD	RMSD	Δ_{err}	max	min
FH51	31.01	-0.72	1.61	0.05	2.12	8.04	8.03	0.01
YBDE18	49.28	1.01	1.56	0.03	2.11	6.52	6.32	0.20
AL2X6	35.88	2.02	2.02	0.06	2.26	4.57	3.82	0.75
DARC	32.47	-4.72	4.72	0.15	4.78	9.29	5.90	3.39
NBPRC	27.71	-0.71	1.87	0.07	2.14	4.03	3.84	0.19
HEAVYSB9	58.02	1.37	2.12	0.04	2.40	4.38	4.08	0.30
BSR36	16.20	0.97	1.08	0.07	1.51	5.22	5.20	0.02
RSE43	7.60	1.21	1.21	0.16	1.82	7.20	7.03	0.17
W4-11	306.91	-4.76	4.81	0.02	5.44	12.89	12.71	0.18
G21EA	33.62	-7.32	7.32	0.22	7.70	15.23	12.30	2.93
G21IP	257.61	-2.39	3.02	0.01	3.39	7.26	7.21	0.05
DIPCS10	654.26	-5.68	5.68	0.01	5.96	12.14	9.47	2.67
PA26	189.05	2.27	2.36	0.01	2.81	6.44	6.30	0.14
SIE4x4	33.72	4.22	4.25	0.13	5.18	11.30	11.11	0.19

Continued on next page

Test set	$\|\overline{\Delta E}\|$	MD	MAD	NMAD	RMSD	Δ_{err}	max	min
ALKBDE10	100.69	-1.68	3.08	0.03	3.84	8.71	8.30	0.41
RC21	35.70	-0.28	1.47	0.04	1.85	4.87	4.80	0.07
ALK8	62.60	4.64	4.64	0.07	6.56	15.91	15.65	0.26
DC13	54.98	-1.76	2.65	0.05	3.37	6.89	6.74	0.15
G2RC	51.26	-0.75	2.30	0.04	2.70	5.57	5.52	0.05
BH76RC	21.39	0.13	1.39	0.07	1.78	4.91	4.89	0.02
MOR23	35.57	-5.23	6.26	0.18	7.54	17.40	17.36	0.04
WCPT18	34.99	-2.02	2.20	0.06	2.73	5.29	5.22	0.07
BHROT27	6.37	0.01	0.14	0.02	0.16	0.31	0.30	0.01
BHPERI	20.87	-1.41	1.70	0.08	2.11	4.45	4.41	0.04
BHDIV10	45.33	-0.58	1.18	0.03	1.33	2.55	2.19	0.36
INV24	32.85	0.23	1.33	0.04	1.75	4.85	4.72	0.13
CR20	19.31	-0.61	0.67	0.03	0.88	2.27	2.23	0.04
CRBH20	46.13	1.82	1.82	0.04	1.99	3.83	3.32	0.51
TMBH17	12.76	0.27	2.21	0.17	2.82	6.27	6.23	0.04
LTMBH26	9.98	-0.57	1.29	0.13	1.95	7.33	7.33	0.00
BH76	18.61	-0.33	1.71	0.09	2.44	9.28	9.25	0.03
ISO34	14.57	-0.21	0.53	0.04	0.72	2.03	2.02	0.01
ICONF	3.27	0.13	0.20	0.06	0.25	0.50	0.49	0.01
ACONF	1.83	-0.22	0.22	0.12	0.25	0.45	0.40	0.05
TAUT15	3.05	-0.12	0.90	0.30	1.09	2.62	2.52	0.10
Amino20x4	2.44	-0.02	0.23	0.09	0.28	0.86	0.86	0.00
PCONF	1.62	0.09	0.99	0.61	1.27	2.45	2.33	0.12
MCONF	4.97	0.74	0.97	0.20	1.06	1.62	1.60	0.02
SCONF	4.60	0.24	0.57	0.12	0.69	1.60	1.59	0.01
PArel	4.63	-0.06	0.70	0.15	0.99	2.61	2.61	0.00
BUT14DIOL	2.80	0.42	0.42	0.15	0.43	0.61	0.59	0.02
EIE22	5.44	0.27	0.30	0.06	0.36	0.80	0.79	0.01
Styrene45	62.64	-0.90	1.32	0.02	1.70	4.61	4.61	0.00
ISOMERIZATION20	31.84	0.18	1.20	0.04	1.62	4.60	4.58	0.02
DIE60	4.71	0.30	0.36	0.08	0.52	1.17	1.16	0.01
IDISP	14.22	7.09	7.09	0.50	10.06	24.26	22.60	1.66
C20C24	30.77	-4.38	8.34	0.27	11.47	21.87	21.03	0.84
S66	5.47	-0.73	0.73	0.13	0.82	2.04	1.86	0.18
S10x8	6.59	0.49	0.50	0.08	0.70	1.83	1.83	0.00
X40	3.76	0.50	0.50	0.13	0.52	1.17	1.01	0.16
HEAVY28	1.24	0.24	0.30	0.24	0.36	1.04	1.00	0.04
CHB6	26.79	0.93	0.93	0.03	1.09	2.46	2.05	0.41
AHB21	22.49	-0.03	0.49	0.02	0.61	1.39	1.37	0.02
IL16	109.04	1.34	1.34	0.01	1.46	2.55	2.19	0.36
PNICO23	4.27	0.41	0.42	0.10	0.47	0.96	0.85	0.11
CT20	0.98	0.29	0.29	0.30	0.31	0.66	0.51	0.15
CARBHB12	6.04	1.07	1.07	0.18	1.14	1.99	1.61	0.38
ADIM6	3.36	1.74	1.74	0.52	1.89	3.61	2.91	0.70

Continued on next page

Test set	$\|\overline{\Delta E}\|$	MD	MAD	NMAD	RMSD	Δ_{err}	max	min
3B-69-TRIM	12.30	1.50	1.50	0.12	1.61	3.48	3.20	0.28
ISOL24	21.92	1.19	1.97	0.09	3.29	13.18	13.00	0.18
C60ISO	98.25	-2.72	3.93	0.04	5.14	11.65	10.74	0.91
L7	18.20	1.20	1.39	0.08	1.74	3.38	3.04	0.34
UPU23	5.72	-0.11	0.89	0.16	1.17	3.42	3.40	0.02
ENZYMES23	15.32	-0.30	1.28	0.08	1.61	4.14	4.02	0.12

Table S41: Statistical analysis for DOD-SCAN-D3(BJ) for all testset in our databsase. The numbers given (all in kcal/mol) are average reaction energy ($|\overline{\Delta E}|$), mean deviation (MD), mean absolute deviation (MAD), MAD normalized with respect to $|\overline{\Delta E}|$ (NMAD), root-mean-square deviation (RMSD), deviation span (Δ_{err}), maximum (max) and minimum deviation (min).

Test set	$\|\overline{\Delta E}\|$	MD	MAD	NMAD	RMSD	Δ_{err}	max	min
FH51	31.01	2.48	2.48	0.08	3.35	12.47	12.44	0.03
YBDE18	49.28	1.76	2.46	0.05	3.20	8.59	8.44	0.15
AL2X6	35.88	3.41	3.41	0.09	3.81	7.51	6.23	1.28
DARC	32.47	-7.54	7.54	0.23	7.70	14.23	9.54	4.69
NBPRC	27.71	-1.96	2.98	0.11	3.35	7.81	6.58	1.23
HEAVYSB9	58.02	1.62	2.51	0.04	2.99	5.34	5.33	0.01
BSR36	16.20	4.44	4.48	0.28	5.29	14.26	13.82	0.44
RSE43	7.60	1.66	1.66	0.22	2.26	9.46	8.96	0.50
W4-11	306.91	-3.90	4.14	0.01	4.84	13.79	13.63	0.16
G21EA	33.62	-6.41	6.41	0.19	6.97	12.53	11.47	1.06
G21IP	257.61	-1.44	2.42	0.01	2.85	7.14	7.11	0.03
DIPCS10	654.26	-3.78	3.88	0.01	4.48	8.04	7.52	0.52
PA26	189.05	2.32	2.39	0.01	2.89	6.75	6.58	0.17
SIE4x4	33.72	2.76	2.88	0.09	3.48	7.53	6.95	0.58
ALKBDE10	100.69	-1.23	3.35	0.03	4.28	8.59	8.31	0.28
RC21	35.70	0.35	1.64	0.05	2.22	7.04	6.94	0.10
ALK8	62.60	6.21	6.27	0.10	9.12	22.81	22.57	0.24
DC13	54.98	-2.40	3.33	0.06	4.99	15.47	14.89	0.58
G2RC	51.26	-1.33	2.73	0.05	3.24	7.81	7.52	0.29
BH76RC	21.39	-0.22	1.34	0.06	1.69	5.25	4.80	0.45
MOR23	35.57	-7.59	8.55	0.24	10.06	20.21	20.20	0.01
WCPT18	34.99	2.76	2.76	0.08	3.49	6.56	6.35	0.21
BHROT27	6.37	0.18	0.18	0.03	0.24	0.59	0.56	0.03
BHPERI	20.87	3.38	3.38	0.16	3.98	8.83	8.74	0.09
BHDIV10	45.33	1.45	1.45	0.03	1.64	3.32	3.06	0.26
INV24	32.85	1.26	1.26	0.04	1.68	3.70	3.67	0.03
CR20	19.31	0.41	0.68	0.04	0.89	2.20	2.17	0.03
CRBH20	46.13	1.28	1.28	0.03	1.39	2.65	2.34	0.31
TMBH17	12.76	-0.53	2.58	0.20	3.09	5.70	5.50	0.20

Continued on next page

Test set	$\lvert\overline{\Delta E}\rvert$	MD	MAD	NMAD	RMSD	Δ_{err}	max	min
LTMBH26	9.98	-0.91	1.64	0.16	2.15	7.14	6.96	0.18
BH76	18.61	-0.96	2.03	0.11	2.64	8.12	7.90	0.22
ISO34	14.57	0.81	0.81	0.06	1.18	4.56	4.47	0.09
ICONF	3.27	0.29	0.29	0.09	0.36	0.71	0.70	0.01
ACONF	1.83	0.67	0.67	0.36	0.74	1.42	1.22	0.20
TAUT15	3.05	0.97	0.97	0.32	1.17	2.47	2.46	0.01
Amino20x4	2.44	0.45	0.45	0.19	0.56	1.53	1.53	0.00
PCONF	1.62	2.28	2.28	1.40	2.80	4.49	4.39	0.10
MCONF	4.97	1.83	1.83	0.37	2.07	3.11	3.08	0.03
SCONF	4.60	1.08	1.08	0.23	1.24	2.65	2.57	0.08
PArel	4.63	0.06	0.78	0.17	1.05	2.78	2.70	0.08
BUT14DIOL	2.80	0.67	0.68	0.24	0.74	1.15	1.15	0.00
EIE22	5.44	0.28	0.30	0.06	0.38	0.89	0.88	0.01
Styrene45	62.64	-0.34	0.84	0.01	1.14	2.95	2.95	0.00
ISOMERIZATION20	31.84	-0.38	1.13	0.04	1.36	2.77	2.72	0.05
DIE60	4.71	0.25	0.35	0.07	0.47	1.45	1.45	0.00
IDISP	14.22	8.93	8.93	0.63	9.95	19.12	16.12	3.00
C20C24	30.77	-6.31	6.74	0.22	9.43	18.17	17.58	0.59
S66	5.47	-0.52	0.52	0.09	0.63	1.38	1.37	0.01
S10x8	6.59	-0.19	0.25	0.04	0.41	1.91	1.91	0.00
X40	3.76	0.26	0.26	0.07	0.45	1.80	1.79	0.01
HEAVY28	1.24	0.60	0.60	0.49	0.67	1.29	1.24	0.05
CHB6	26.79	-0.13	0.79	0.03	0.94	1.96	1.79	0.17
AHB21	22.49	-0.65	0.70	0.03	0.94	2.31	2.31	0.00
IL16	109.04	0.14	0.33	0.00	0.38	0.73	0.68	0.05
PNICO23	4.27	0.88	0.88	0.21	0.92	2.04	1.54	0.50
CT20	0.98	-0.02	0.05	0.05	0.06	0.12	0.12	0.00
CARBHB12	6.04	1.49	1.49	0.25	1.60	2.76	2.25	0.51
ADIM6	3.36	3.40	3.40	1.01	3.73	7.11	5.79	1.32
3B-69-TRIM	12.30	-0.57	0.60	0.05	0.78	2.50	2.48	0.02
ISOL24	21.92	2.24	3.56	0.16	6.54	27.07	26.98	0.09
C60ISO	98.25	-4.08	4.76	0.05	6.47	13.25	13.12	0.13
L7	18.20	-5.12	5.12	0.28	6.43	12.11	11.47	0.64
UPU23	5.72	-0.68	1.56	0.27	2.21	6.78	6.75	0.03
ENZYMES23	15.32	-0.61	1.86	0.12	2.24	4.68	4.67	0.01

S5.2 D4-Dispersion-Corrected Double Hybrids

Table S42: Statistical analysis for B2-PLYP-D4 for all testset in our databsase. The numbers given (all in kcal/mol) are average reaction energy ($|\overline{\Delta E}|$), mean deviation (MD), mean absolute deviation (MAD), MAD normalized with respect to $|\overline{\Delta E}|$ (NMAD), root-mean-square deviation (RMSD), deviation span (Δ_{err}), maximum (max) and minimum deviation (min).

Test set	$\|\overline{\Delta E}\|$	MD	MAD	NMAD	RMSD	Δ_{err}	max	min
FH51	31.01	0.41	1.27	0.04	1.72	4.63	4.63	0.00
YBDE18	49.28	-2.23	3.14	0.06	3.35	7.03	5.52	1.51
AL2X6	35.88	-2.01	2.01	0.06	2.11	4.13	2.90	1.23
DARC	32.47	3.39	3.39	0.10	3.45	6.37	4.03	2.34
NBPRC	27.71	-0.47	1.44	0.05	1.45	2.92	1.67	1.25
HEAVYSB9	58.02	-2.06	2.12	0.04	2.78	5.48	5.34	0.14
BSR36	16.20	0.13	0.39	0.02	0.61	2.22	2.22	0.00
RSE43	7.60	-0.38	0.48	0.06	0.63	1.74	1.70	0.04
W4-11	306.91	-2.76	3.16	0.01	3.98	13.47	13.44	0.03
G21EA	33.62	5.07	5.93	0.18	9.72	32.80	32.04	0.76
G21IP	257.61	-1.11	2.56	0.01	3.00	7.81	7.70	0.11
DIPCS10	654.26	-3.05	3.57	0.01	4.14	9.68	8.35	1.33
PA26	189.05	1.49	1.85	0.01	2.64	6.86	6.86	0.00
SIE4x4	33.72	9.42	9.42	0.28	10.94	21.70	21.40	0.30
ALKBDE10	100.69	0.68	3.76	0.04	4.80	8.65	8.54	0.11
RC21	35.70	-0.35	1.15	0.03	1.38	3.38	3.24	0.14
ALK8	62.60	-1.18	2.37	0.04	3.25	6.94	6.83	0.11
DC13	54.98	2.47	6.99	0.13	9.41	19.83	19.38	0.45
G2RC	51.26	-0.02	1.91	0.04	2.39	6.33	6.22	0.11
BH76RC	21.39	-0.38	1.02	0.05	1.42	4.53	4.52	0.01
MOR23	35.57	-3.13	4.29	0.12	5.88	12.37	12.32	0.05
WCPT18	34.99	-2.37	2.41	0.07	2.97	5.40	5.39	0.01
BHROT27	6.37	0.17	0.24	0.04	0.35	1.09	1.08	0.01
BHPERI	20.87	-1.65	1.65	0.08	1.91	4.35	4.27	0.08
BHDIV10	45.33	-0.72	2.16	0.05	2.64	4.88	4.77	0.11
INV24	32.85	-0.62	1.32	0.04	1.59	3.04	3.02	0.02
CR20	19.31	-6.01	6.01	0.31	6.05	11.80	7.29	4.51
CRBH20	46.13	-4.64	4.64	0.10	4.68	9.54	5.93	3.61
TMBH17	12.76	0.15	3.54	0.28	4.66	10.39	10.35	0.04
LTMBH26	9.98	-1.17	2.09	0.21	2.96	10.28	10.20	0.08
BH76	18.61	-2.96	3.18	0.17	3.68	7.98	7.84	0.14
ISO34	14.57	0.07	1.21	0.08	1.84	7.58	7.56	0.02
ICONF	3.27	0.12	0.25	0.08	0.32	0.75	0.70	0.05
ACONF	1.83	-0.09	0.09	0.05	0.11	0.24	0.23	0.01
TAUT15	3.05	0.20	0.80	0.26	0.95	1.88	1.86	0.02
Amino20x4	2.44	0.02	0.19	0.08	0.27	1.08	1.08	0.00
PCONF	1.62	-0.40	2.24	1.39	2.47	4.27	4.02	0.25
MCONF	4.97	0.48	0.81	0.16	1.00	3.69	3.61	0.08

Continued on next page

Test set	$\lvert\overline{\Delta E}\rvert$	MD	MAD	NMAD	RMSD	Δ_{err}	max	min
SCONF	4.60	1.98	3.74	0.81	4.13	8.34	8.19	0.15
PArel	4.63	-0.01	0.72	0.16	1.11	2.71	2.70	0.01
BUT14DIOL	2.80	0.62	0.62	0.22	0.63	0.78	0.72	0.06
EIE22	5.44	0.87	0.87	0.16	0.98	2.02	1.99	0.03
Styrene45	62.64	3.23	3.94	0.06	5.06	13.89	13.89	0.00
ISOMERIZATION20	31.84	-0.41	1.47	0.05	2.04	4.51	4.43	0.08
DIE60	4.71	0.78	0.78	0.17	0.84	1.70	1.39	0.31
IDISP	14.22	-0.17	2.12	0.15	3.15	6.97	6.88	0.09
C20C24	30.77	-2.03	28.14	0.91	30.96	64.56	49.54	15.02
S66	5.47	-0.39	0.40	0.07	0.46	0.98	0.96	0.02
S10x8	6.59	0.09	0.19	0.03	0.30	1.16	1.16	0.00
X40	3.76	0.33	0.38	0.10	0.47	1.61	1.58	0.03
HEAVY28	1.24	0.05	0.20	0.16	0.27	0.79	0.78	0.01
CHB6	26.79	0.81	0.85	0.03	1.08	2.24	2.11	0.13
AHB21	22.49	-0.50	0.76	0.03	0.95	2.34	2.16	0.18
IL16	109.04	0.86	0.86	0.01	1.01	1.84	1.84	0.00
PNICO23	4.27	0.01	0.12	0.03	0.19	0.53	0.52	0.01
CT20	0.98	0.13	0.14	0.14	0.15	0.29	0.25	0.04
CARBHB12	6.04	0.99	0.99	0.16	1.11	2.07	1.86	0.21
ADIM6	3.36	0.85	0.85	0.25	0.95	1.76	1.49	0.27
3B-69-TRIM	12.30	0.50	0.58	0.05	0.68	1.88	1.86	0.02

Table S43: Statistical analysis for B2GP-PLYP-D4 for all testset in our databsase. The numbers given (all in kcal/mol) are average reaction energy ($\lvert\overline{\Delta E}\rvert$), mean deviation (MD), mean absolute deviation (MAD), MAD normalized with respect to $\lvert\overline{\Delta E}\rvert$ (NMAD), root-mean-square deviation (RMSD), deviation span (Δ_{err}), maximum (max) and minimum deviation (min).

Test set	$\lvert\overline{\Delta E}\rvert$	MD	MAD	NMAD	RMSD	Δ_{err}	max	min
FH51	31.01	-0.62	1.15	0.04	1.54	4.02	3.98	0.04
YBDE18	49.28	-0.91	1.83	0.04	2.11	5.17	5.01	0.16
AL2X6	35.88	-0.60	0.88	0.02	1.01	1.87	1.57	0.30
DARC	32.47	-0.08	0.23	0.01	0.29	0.61	0.56	0.05
NBPRC	27.71	-1.28	1.58	0.06	1.81	2.59	2.55	0.04
HEAVYSB9	58.02	-0.98	1.78	0.03	2.33	5.64	5.29	0.35
BSR36	16.20	1.74	1.79	0.11	2.36	7.33	7.16	0.17
RSE43	7.60	0.26	0.41	0.05	0.73	3.43	3.41	0.02
W4-11	306.91	-4.95	5.17	0.02	6.11	17.36	17.22	0.14
G21EA	33.62	5.22	6.09	0.18	9.81	32.71	31.94	0.77
G21IP	257.61	-1.24	2.35	0.01	2.86	8.35	8.35	0.00
DIPCS10	654.26	-3.27	3.39	0.01	4.29	9.07	8.91	0.16
PA26	189.05	0.35	1.38	0.01	1.95	5.51	5.47	0.04
SIE4x4	33.72	6.09	6.12	0.18	7.19	14.75	14.51	0.24

Continued on next page

Test set	$\lvert\overline{\Delta E}\rvert$	MD	MAD	NMAD	RMSD	Δ_{err}	max	min
ALKBDE10	100.69	-0.37	3.47	0.03	4.50	9.70	9.61	0.09
RC21	35.70	-0.33	1.13	0.03	1.35	3.43	3.35	0.08
ALK8	62.60	-0.12	2.37	0.04	3.01	5.34	5.01	0.33
DC13	54.98	1.27	4.93	0.09	7.20	17.48	17.19	0.29
G2RC	51.26	-1.31	2.21	0.04	2.72	8.21	7.90	0.31
BH76RC	21.39	-0.46	0.98	0.05	1.45	4.83	4.77	0.06
MOR23	35.57	-5.06	6.19	0.17	7.46	15.68	15.37	0.31
WCPT18	34.99	-1.77	2.21	0.06	2.72	5.09	4.97	0.12
BHROT27	6.37	0.21	0.25	0.04	0.35	1.07	1.06	0.01
BHPERI	20.87	-1.88	1.88	0.09	2.16	5.49	5.14	0.35
BHDIV10	45.33	0.09	1.67	0.04	2.04	4.37	4.21	0.16
INV24	32.85	-0.17	1.59	0.05	1.87	3.39	3.28	0.11
CR20	19.31	-4.34	4.34	0.22	4.40	8.72	5.62	3.10
CRBH20	46.13	-1.47	1.47	0.03	1.56	3.06	2.63	0.43
TMBH17	12.76	0.75	2.81	0.22	3.84	9.28	9.05	0.23
LTMBH26	9.98	-0.60	1.41	0.14	2.18	8.52	8.38	0.14
BH76	18.61	-1.44	2.04	0.11	2.56	7.93	7.87	0.06
ISO34	14.57	0.23	0.86	0.06	1.45	6.55	6.54	0.01
ICONF	3.27	0.18	0.26	0.08	0.35	0.76	0.76	0.00
ACONF	1.83	-0.21	0.21	0.11	0.24	0.46	0.42	0.04
TAUT15	3.05	0.35	0.66	0.22	0.83	1.79	1.77	0.02
Amino20x4	2.44	0.05	0.25	0.10	0.33	1.07	1.07	0.00
PCONF	1.62	-0.42	2.28	1.41	2.52	4.40	4.16	0.24
MCONF	4.97	1.01	1.33	0.27	1.49	3.53	3.50	0.03
SCONF	4.60	1.94	3.66	0.80	4.05	8.19	8.03	0.16
PArel	4.63	0.10	0.60	0.13	0.86	1.94	1.93	0.01
BUT14DIOL	2.80	0.70	0.70	0.25	0.72	0.98	0.91	0.07
EIE22	5.44	0.65	0.66	0.12	0.75	1.62	1.60	0.02
Styrene45	62.64	3.22	3.44	0.05	4.29	11.52	11.52	0.00
ISOMERIZATION20	31.84	-0.69	1.51	0.05	1.98	4.04	4.02	0.02
DIE60	4.71	0.63	0.63	0.13	0.71	1.41	1.27	0.14
IDISP	14.22	-1.49	5.57	0.39	7.96	18.68	17.63	1.05
C20C24	30.77	-1.97	21.73	0.71	24.18	49.12	38.77	10.35
S66	5.47	0.13	0.20	0.04	0.29	0.95	0.94	0.01
S10x8	6.59	-0.22	0.28	0.04	0.35	0.80	0.80	0.00
X40	3.76	0.08	0.26	0.07	0.40	1.33	1.33	0.00
HEAVY28	1.24	0.19	0.27	0.22	0.35	0.98	0.97	0.01
CHB6	26.79	0.70	0.74	0.03	0.94	1.93	1.81	0.12
AHB21	22.49	-0.69	0.82	0.04	1.12	2.60	2.59	0.01
IL16	109.04	0.65	0.65	0.01	0.83	1.84	1.83	0.01
PNICO23	4.27	0.26	0.30	0.07	0.35	0.80	0.79	0.01
CT20	0.98	0.04	0.05	0.05	0.06	0.12	0.12	0.00
CARBHB12	6.04	1.22	1.22	0.20	1.33	2.31	1.98	0.33
ADIM6	3.36	2.02	2.02	0.60	2.25	4.19	3.52	0.67

Continued on next page

Test set	$\lvert\overline{\Delta E}\rvert$	MD	MAD	NMAD	RMSD	Δ_{err}	max	min
3B-69-TRIM	12.30	-0.54	0.58	0.05	0.74	1.93	1.93	0.00

Table S44: Statistical analysis for mPW2-PLYP-D4 for all testset in our databsase. The numbers given (all in kcal/mol) are average reaction energy ($\lvert\overline{\Delta E}\rvert$), mean deviation (MD), mean absolute deviation (MAD), MAD normalized with respect to $\lvert\overline{\Delta E}\rvert$ (NMAD), root-mean-square deviation (RMSD), deviation span (Δ_{err}), maximum (max) and minimum deviation (min).

Test set	$\lvert\overline{\Delta E}\rvert$	MD	MAD	NMAD	RMSD	Δ_{err}	max	min
FH51	31.01	0.10	1.21	0.04	1.71	5.20	5.20	0.00
YBDE18	49.28	-2.83	3.38	0.07	3.52	7.45	5.42	2.03
AL2X6	35.88	-2.22	2.22	0.06	2.51	4.08	3.61	0.47
DARC	32.47	3.56	3.56	0.11	3.66	6.37	4.38	1.99
NBPRC	27.71	-0.69	2.02	0.07	2.08	3.97	2.66	1.31
HEAVYSB9	58.02	-2.95	2.95	0.05	3.47	7.33	6.19	1.14
BSR36	16.20	-1.60	1.60	0.10	1.86	4.82	4.40	0.42
RSE43	7.60	-0.27	0.39	0.05	0.52	1.43	1.41	0.02
W4-11	306.91	-5.03	5.16	0.02	6.29	19.24	19.13	0.11
G21EA	33.62	5.01	5.91	0.18	9.69	32.60	31.82	0.78
G21IP	257.61	-0.52	2.41	0.01	2.95	6.96	6.91	0.05
DIPCS10	654.26	-1.71	3.07	0.00	3.92	9.37	8.46	0.91
PA26	189.05	0.39	1.48	0.01	2.11	6.13	6.10	0.03
SIE4x4	33.72	8.53	8.53	0.25	10.00	20.19	20.10	0.09
ALKBDE10	100.69	-0.85	3.68	0.04	4.47	10.42	9.87	0.55
RC21	35.70	-0.17	0.92	0.03	1.21	2.83	2.78	0.05
ALK8	62.60	-1.03	2.68	0.04	3.82	8.28	8.13	0.15
DC13	54.98	1.85	7.65	0.14	10.80	22.23	22.08	0.15
G2RC	51.26	-0.82	2.10	0.04	2.49	6.43	6.26	0.17
BH76RC	21.39	-0.46	1.23	0.06	1.62	4.46	4.41	0.05
MOR23	35.57	-1.56	3.17	0.09	4.56	12.75	12.70	0.05
WCPT18	34.99	-1.45	2.00	0.06	2.43	4.74	4.48	0.26
BHROT27	6.37	0.22	0.28	0.04	0.39	1.14	1.12	0.02
BHPERI	20.87	-0.45	0.73	0.04	0.96	3.18	3.14	0.04
BHDIV10	45.33	0.14	1.84	0.04	2.32	4.80	4.78	0.02
INV24	32.85	-0.42	1.40	0.04	1.64	3.01	2.90	0.11
CR20	19.31	-5.91	5.91	0.31	5.97	11.70	7.61	4.09
CRBH20	46.13	-4.06	4.06	0.09	4.10	8.30	5.31	2.99
TMBH17	12.76	1.37	3.22	0.25	4.71	11.90	11.87	0.03
LTMBH26	9.98	-0.69	1.75	0.18	2.89	11.24	11.17	0.07
BH76	18.61	-2.51	2.77	0.15	3.29	7.52	7.43	0.09
ISO34	14.57	0.12	1.26	0.09	1.92	8.13	8.13	0.00
ICONF	3.27	0.15	0.33	0.10	0.40	0.86	0.77	0.09
ACONF	1.83	0.03	0.03	0.02	0.05	0.12	0.12	0.00

Continued on next page

Test set	$\|\overline{\Delta E}\|$	MD	MAD	NMAD	RMSD	Δ_{err}	max	min
TAUT15	3.05	0.18	0.72	0.24	0.87	1.75	1.75	0.00
Amino20x4	2.44	0.03	0.15	0.06	0.21	0.94	0.94	0.00
PCONF	1.62	-0.49	2.00	1.23	2.25	3.54	3.45	0.09
MCONF	4.97	0.26	0.43	0.09	0.47	0.84	0.79	0.05
SCONF	4.60	1.92	3.56	0.77	3.93	7.83	7.69	0.14
PArel	4.63	0.04	0.66	0.14	0.96	2.83	2.80	0.03
BUT14DIOL	2.80	0.55	0.55	0.20	0.56	0.80	0.72	0.08
EIE22	5.44	0.83	0.83	0.15	0.92	1.77	1.77	0.00
Styrene45	62.64	3.75	4.38	0.07	5.53	14.85	14.85	0.00
ISOMERIZATION20	31.84	-0.51	1.33	0.04	1.85	3.95	3.89	0.06
DIE60	4.71	0.72	0.72	0.15	0.80	1.58	1.40	0.18
IDISP	14.22	0.07	1.85	0.13	2.05	3.94	3.43	0.51
C20C24	30.77	-0.05	31.86	1.04	34.07	71.93	52.03	19.90
S66	5.47	-0.24	0.34	0.06	0.42	1.20	1.18	0.02
S10x8	6.59	-0.11	0.33	0.05	0.44	2.01	2.01	0.00
X40	3.76	0.20	0.34	0.09	0.45	1.21	1.19	0.02
HEAVY28	1.24	0.08	0.23	0.18	0.31	0.81	0.80	0.01
CHB6	26.79	0.27	0.67	0.03	0.90	1.96	1.85	0.11
AHB21	22.49	-0.93	0.99	0.04	1.32	2.99	2.98	0.01
IL16	109.04	0.71	0.73	0.01	0.89	1.85	1.81	0.04
PNICO23	4.27	0.06	0.22	0.05	0.29	0.87	0.87	0.00
CT20	0.98	-0.13	0.13	0.14	0.15	0.39	0.34	0.05
CARBHB12	6.04	1.14	1.14	0.19	1.24	2.29	1.91	0.38
ADIM6	3.36	0.98	0.98	0.29	1.05	2.01	1.53	0.48
3B-69-TRIM	12.30	0.02	0.44	0.04	0.60	2.19	2.19	0.00

Table S45: Statistical analysis for PBE0-DH-D4 for all testset in our databsase. The numbers given (all in kcal/mol) are average reaction energy ($|\overline{\Delta E}|$), mean deviation (MD), mean absolute deviation (MAD), MAD normalized with respect to $|\overline{\Delta E}|$ (NMAD), root-mean-square deviation (RMSD), deviation span (Δ_{err}), maximum (max) and minimum deviation (min).

Test set	$\|\overline{\Delta E}\|$	MD	MAD	NMAD	RMSD	Δ_{err}	max	min
FH51	31.01	-2.48	3.20	0.10	4.11	12.35	12.30	0.05
YBDE18	49.28	-0.33	2.04	0.04	2.25	4.43	4.35	0.08
AL2X6	35.88	1.14	1.15	0.03	1.44	2.26	2.24	0.02
DARC	32.47	-7.74	7.74	0.24	7.96	16.86	10.95	5.91
NBPRC	27.71	-2.48	2.48	0.09	2.77	4.56	4.01	0.55
HEAVYSB9	58.02	-1.54	1.54	0.03	1.70	3.47	2.84	0.63
BSR36	16.20	-1.56	1.56	0.10	1.65	3.38	2.66	0.72
RSE43	7.60	-0.37	0.44	0.06	0.59	1.68	1.66	0.02
W4-11	306.91	-7.02	7.13	0.02	8.76	28.60	28.60	0.00
G21EA	33.62	5.03	5.91	0.18	9.69	32.63	31.86	0.77

Continued on next page

Test set	$\lvert\overline{\Delta E}\rvert$	MD	MAD	NMAD	RMSD	Δ_{err}	max	min
G21IP	257.61	0.53	3.04	0.01	3.78	8.30	8.22	0.08
DIPCS10	654.26	0.48	2.96	0.00	3.39	6.88	6.17	0.71
PA26	189.05	1.31	1.84	0.01	2.43	6.56	6.56	0.00
SIE4x4	33.72	6.87	6.87	0.20	8.18	18.66	18.00	0.66
ALKBDE10	100.69	-5.65	6.18	0.06	7.38	15.43	15.09	0.34
RC21	35.70	4.01	4.54	0.13	5.56	14.97	14.35	0.62
ALK8	62.60	3.72	3.72	0.06	4.58	8.27	7.79	0.48
DC13	54.98	-2.65	8.40	0.15	10.28	20.62	20.16	0.46
G2RC	51.26	-4.48	6.23	0.12	8.17	21.83	21.81	0.02
BH76RC	21.39	-0.53	2.10	0.10	2.67	6.58	6.46	0.12
MOR23	35.57	-3.00	3.70	0.10	4.32	9.72	9.07	0.65
WCPT18	34.99	-2.60	2.87	0.08	3.57	6.83	6.66	0.17
BHROT27	6.37	0.52	0.53	0.08	0.71	1.39	1.38	0.01
BHPERI	20.87	-1.85	2.51	0.12	2.89	4.80	4.79	0.01
BHDIV10	45.33	-1.81	2.56	0.06	2.88	5.87	4.96	0.91
INV24	32.85	0.17	1.58	0.05	2.26	6.63	6.56	0.07
CR20	19.31	2.32	2.32	0.12	2.43	4.38	3.45	0.93
CRBH20	46.13	4.35	4.35	0.09	4.42	8.57	5.69	2.88
TMBH17	12.76	2.21	3.62	0.28	4.10	7.00	6.51	0.49
LTMBH26	9.98	-0.26	2.08	0.21	3.27	13.07	12.77	0.30
BH76	18.61	-1.68	2.34	0.13	2.64	6.71	6.66	0.05
ISO34	14.57	0.01	1.36	0.09	1.85	3.67	3.65	0.02
ICONF	3.27	0.34	0.37	0.11	0.53	1.32	1.31	0.01
ACONF	1.83	0.03	0.03	0.02	0.05	0.09	0.09	0.00
TAUT15	3.05	0.52	0.84	0.27	1.13	2.27	2.26	0.01
Amino20x4	2.44	0.10	0.24	0.10	0.31	0.76	0.76	0.00
PCONF	1.62	-0.45	2.16	1.33	2.39	3.87	3.71	0.16
MCONF	4.97	0.30	0.41	0.08	0.47	0.83	0.81	0.02
SCONF	4.60	1.93	3.60	0.78	3.97	7.99	7.84	0.15
PArel	4.63	0.75	1.16	0.25	1.49	3.70	3.67	0.03
BUT14DIOL	2.80	0.38	0.38	0.13	0.39	0.60	0.59	0.01
EIE22	5.44	0.82	0.86	0.16	0.99	2.01	1.98	0.03
Styrene45	62.64	0.83	3.17	0.05	3.92	8.46	8.46	0.00
ISOMERIZATION20	31.84	-0.59	1.74	0.05	2.20	4.67	4.64	0.03
DIE60	4.71	1.02	1.03	0.22	1.24	2.65	2.63	0.02
IDISP	14.22	-1.55	2.97	0.21	4.94	12.11	11.74	0.37
C20C24	30.77	-11.82	11.82	0.38	12.01	24.51	14.99	9.52
S66	5.47	-0.16	0.33	0.06	0.42	1.12	1.11	0.01
S10x8	6.59	-0.22	0.34	0.05	0.55	2.24	2.24	0.00
X40	3.76	0.18	0.39	0.10	0.49	1.41	1.34	0.07
HEAVY28	1.24	0.08	0.24	0.19	0.34	1.05	1.02	0.03
CHB6	26.79	0.19	0.61	0.02	0.75	1.33	1.22	0.11
AHB21	22.49	-1.75	1.80	0.08	2.40	5.86	5.75	0.11
IL16	109.04	-0.02	0.59	0.01	0.66	1.36	1.14	0.22

Continued on next page

Test set	$\|\overline{\Delta E}\|$	MD	MAD	NMAD	RMSD	Δ_{err}	max	min
PNICO23	4.27	0.54	0.55	0.13	0.74	2.31	2.30	0.01
CT20	0.98	0.14	0.15	0.15	0.17	0.35	0.32	0.03
CARBHB12	6.04	1.43	1.43	0.24	1.63	3.20	2.81	0.39
ADIM6	3.36	0.26	0.26	0.08	0.28	0.55	0.44	0.11
3B-69-TRIM	12.30	0.16	0.66	0.05	0.84	2.54	2.49	0.05

Table S46: Statistical analysis for PBE0-2-D4 for all testset in our databsase. The numbers given (all in kcal/mol) are average reaction energy ($|\overline{\Delta E}|$), mean deviation (MD), mean absolute deviation (MAD), MAD normalized with respect to $|\overline{\Delta E}|$ (NMAD), root-mean-square deviation (RMSD), deviation span (Δ_{err}), maximum (max) and minimum deviation (min).

Test set	$\|\overline{\Delta E}\|$	MD	MAD	NMAD	RMSD	Δ_{err}	max	min
FH51	31.01	-2.80	3.22	0.10	4.07	11.72	11.67	0.05
YBDE18	49.28	2.56	3.58	0.07	4.41	8.58	8.31	0.27
AL2X6	35.88	1.91	1.91	0.05	2.18	3.82	3.35	0.47
DARC	32.47	-8.83	8.83	0.27	8.87	18.21	10.18	8.03
NBPRC	27.71	-2.65	2.65	0.10	2.79	6.31	4.55	1.76
HEAVYSB9	58.02	1.00	1.73	0.03	2.02	3.87	3.45	0.42
BSR36	16.20	2.60	2.62	0.16	3.54	10.84	10.55	0.29
RSE43	7.60	1.14	1.15	0.15	2.00	8.01	7.90	0.11
W4-11	306.91	-5.03	5.86	0.02	6.93	17.50	17.45	0.05
G21EA	33.62	5.01	6.04	0.18	9.72	32.27	31.52	0.75
G21IP	257.61	-0.66	2.30	0.01	3.02	9.82	9.75	0.07
DIPCS10	654.26	-2.32	3.45	0.01	4.21	8.86	8.12	0.74
PA26	189.05	0.67	1.55	0.01	2.00	4.76	4.74	0.02
SIE4x4	33.72	1.15	1.49	0.04	1.84	3.61	3.61	0.00
ALKBDE10	100.69	-1.03	4.03	0.04	4.77	9.87	9.09	0.78
RC21	35.70	0.88	2.82	0.08	3.82	11.34	10.65	0.69
ALK8	62.60	3.38	3.38	0.05	4.37	9.41	8.71	0.70
DC13	54.98	-1.84	7.95	0.14	9.05	17.27	16.85	0.42
G2RC	51.26	-4.27	5.22	0.10	6.37	16.66	15.81	0.85
BH76RC	21.39	-0.45	2.15	0.10	2.74	6.46	6.32	0.14
MOR23	35.57	-6.86	8.27	0.23	9.92	19.70	19.67	0.03
WCPT18	34.99	-2.85	3.11	0.09	3.80	6.69	6.67	0.02
BHROT27	6.37	0.36	0.37	0.06	0.47	1.14	1.13	0.01
BHPERI	20.87	-3.53	3.87	0.19	4.29	9.23	8.12	1.11
BHDIV10	45.33	-0.68	1.73	0.04	1.99	2.77	2.69	0.08
INV24	32.85	-0.01	1.91	0.06	2.43	5.14	5.07	0.07
CR20	19.31	1.32	1.40	0.07	1.55	2.60	2.49	0.11
CRBH20	46.13	6.56	6.56	0.14	6.60	12.73	7.42	5.31
TMBH17	12.76	0.75	2.26	0.18	2.75	5.87	5.72	0.15
LTMBH26	9.98	0.10	1.24	0.12	1.70	4.86	4.84	0.02

Continued on next page

Test set	$\lvert\overline{\Delta E}\rvert$	MD	MAD	NMAD	RMSD	Δ_{err}	max	min
BH76	18.61	0.61	1.95	0.10	2.91	11.23	11.21	0.02
ISO34	14.57	0.31	1.18	0.08	1.72	4.23	4.22	0.01
ICONF	3.27	0.38	0.43	0.13	0.51	1.09	0.99	0.10
ACONF	1.83	-0.12	0.12	0.07	0.15	0.36	0.35	0.01
TAUT15	3.05	0.83	0.89	0.29	1.09	1.89	1.83	0.06
Amino20x4	2.44	0.10	0.26	0.11	0.34	0.83	0.83	0.00
PCONF	1.62	-0.62	1.67	1.03	2.28	4.76	4.60	0.16
MCONF	4.97	0.86	1.05	0.21	1.17	1.91	1.89	0.02
SCONF	4.60	1.86	3.31	0.72	3.63	7.04	6.93	0.11
PArel	4.63	0.54	1.10	0.24	1.39	3.98	3.91	0.07
BUT14DIOL	2.80	0.64	0.64	0.23	0.66	0.83	0.81	0.02
EIE22	5.44	0.49	0.53	0.10	0.61	1.40	1.35	0.05
Styrene45	62.64	0.77	2.79	0.04	3.54	8.08	8.08	0.00
ISOMERIZATION20	31.84	-1.25	2.01	0.06	2.84	9.78	9.59	0.19
DIE60	4.71	0.67	0.67	0.14	0.83	1.76	1.74	0.02
IDISP	14.22	-4.47	8.15	0.57	13.38	33.01	31.66	1.35
C20C24	30.77	-8.31	8.31	0.27	9.22	15.29	12.41	2.88
S66	5.47	-0.65	0.65	0.12	0.75	1.99	1.96	0.03
S10x8	6.59	0.20	0.25	0.04	0.35	1.19	1.19	0.00
X40	3.76	0.46	0.54	0.14	0.58	1.22	1.22	0.00
HEAVY28	1.24	0.10	0.27	0.22	0.41	1.16	1.15	0.01
CHB6	26.79	1.06	1.06	0.04	1.17	2.15	1.74	0.41
AHB21	22.49	-1.05	1.16	0.05	1.65	3.89	3.88	0.01
IL16	109.04	0.82	0.95	0.01	1.12	2.10	2.01	0.09
PNICO23	4.27	0.38	0.41	0.10	0.49	1.13	1.12	0.01
CT20	0.98	0.29	0.29	0.30	0.31	0.69	0.51	0.18
CARBHB12	6.04	1.42	1.42	0.24	1.59	2.81	2.49	0.32
ADIM6	3.36	1.31	1.31	0.39	1.48	2.83	2.43	0.40
3B-69-TRIM	12.30	1.17	1.20	0.10	1.40	3.63	3.63	0.00

Table S47: Statistical analysis for revDSD-BLYP-D4 for all testset in our databsase. The numbers given (all in kcal/mol) are average reaction energy ($\lvert\overline{\Delta E}\rvert$), mean deviation (MD), mean absolute deviation (MAD), MAD normalized with respect to $\lvert\overline{\Delta E}\rvert$ (NMAD), root-mean-square deviation (RMSD), deviation span (Δ_{err}), maximum (max) and minimum deviation (min).

Test set	$\lvert\overline{\Delta E}\rvert$	MD	MAD	NMAD	RMSD	Δ_{err}	max	min
FH51	31.01	1.10	1.10	0.04	1.51	4.13	4.12	0.01
YBDE18	49.28	0.16	1.26	0.03	2.10	6.55	6.51	0.04
AL2X6	35.88	-0.61	1.15	0.03	1.29	2.27	1.92	0.35
DARC	32.47	-1.54	1.55	0.05	1.72	2.65	2.59	0.06
NBPRC	27.71	-0.32	1.48	0.05	1.75	3.10	2.95	0.15
HEAVYSB9	58.02	-0.94	1.65	0.03	2.15	5.41	5.06	0.35

Continued on next page

Test set	$\lvert\overline{\Delta E}\rvert$	MD	MAD	NMAD	RMSD	Δ_{err}	max	min
BSR36	16.20	1.48	1.56	0.10	2.00	6.45	6.34	0.11
RSE43	7.60	1.00	1.00	0.13	1.58	6.33	6.15	0.18
W4-11	306.91	-4.22	4.51	0.01	5.50	18.16	18.07	0.09
G21EA	33.62	-6.63	6.63	0.20	7.03	12.80	11.34	1.46
G21IP	257.61	-1.86	2.59	0.01	3.06	7.50	7.38	0.12
DIPCS10	654.26	-4.36	4.44	0.01	5.10	9.66	9.23	0.43
PA26	189.05	0.99	1.52	0.01	2.07	5.30	5.29	0.01
SIE4x4	33.72	4.61	4.68	0.14	5.59	12.35	11.86	0.49
ALKBDE10	100.69	-0.36	3.13	0.03	4.25	10.27	10.11	0.16
RC21	35.70	-1.04	1.45	0.04	1.78	3.48	3.39	0.09
ALK8	62.60	-0.28	2.69	0.04	3.39	5.49	5.49	0.00
DC13	54.98	0.39	4.31	0.08	5.78	12.83	12.60	0.23
G2RC	51.26	-0.79	1.86	0.04	2.31	5.93	5.67	0.26
BH76RC	21.39	-0.23	1.11	0.05	1.51	4.59	4.58	0.01
MOR23	35.57	-3.63	4.82	0.14	6.22	13.07	13.07	0.00
WCPT18	34.99	2.08	2.08	0.06	2.50	5.04	4.67	0.37
BHROT27	6.37	0.16	0.16	0.03	0.21	0.59	0.58	0.01
BHPERI	20.87	1.52	1.52	0.07	1.84	4.43	4.33	0.10
BHDIV10	45.33	1.22	1.22	0.03	1.55	2.94	2.79	0.15
INV24	32.85	1.91	1.91	0.06	2.35	4.78	4.72	0.06
CR20	19.31	-3.03	3.03	0.16	3.11	5.93	4.38	1.55
CRBH20	46.13	-0.00	0.51	0.01	0.62	1.37	1.35	0.02
TMBH17	12.76	0.55	2.54	0.20	3.41	7.56	7.40	0.16
LTMBH26	9.98	-0.84	1.44	0.14	2.05	6.78	6.77	0.01
BH76	18.61	-0.63	1.80	0.10	2.57	8.53	8.50	0.03
ISO34	14.57	0.80	0.80	0.05	1.13	3.90	3.89	0.01
ICONF	3.27	0.20	0.20	0.06	0.25	0.47	0.46	0.01
ACONF	1.83	0.18	0.18	0.10	0.20	0.39	0.34	0.05
TAUT15	3.05	0.68	0.68	0.22	0.88	2.34	2.31	0.03
Amino20x4	2.44	0.20	0.20	0.08	0.24	0.52	0.51	0.01
PCONF	1.62	0.80	0.80	0.50	1.06	1.83	1.81	0.02
MCONF	4.97	0.67	0.67	0.13	0.73	1.29	1.16	0.13
SCONF	4.60	0.70	0.70	0.15	0.81	1.68	1.61	0.07
PArel	4.63	-0.08	0.56	0.12	0.79	1.93	1.87	0.06
BUT14DIOL	2.80	0.67	0.67	0.24	0.69	0.99	0.90	0.09
EIE22	5.44	0.40	0.40	0.07	0.47	1.08	1.05	0.03
Styrene45	62.64	2.10	2.39	0.04	3.12	8.00	8.00	0.00
ISOMERIZATION20	31.84	-0.32	1.13	0.04	1.46	3.61	3.60	0.01
DIE60	4.71	0.35	0.36	0.08	0.46	0.96	0.96	0.00
IDISP	14.22	5.18	5.18	0.36	7.44	17.91	16.76	1.15
C20C24	30.77	-0.47	19.08	0.62	20.93	40.60	32.00	8.60
S66	5.47	-0.69	0.69	0.13	0.80	2.07	2.05	0.02
S10x8	6.59	0.25	0.32	0.05	0.45	1.60	1.60	0.00
X40	3.76	0.47	0.47	0.12	0.56	1.48	1.44	0.04

Continued on next page

Test set	$\|\overline{\Delta E}\|$	MD	MAD	NMAD	RMSD	Δ_{err}	max	min
HEAVY28	1.24	0.00	0.21	0.17	0.29	0.78	0.77	0.01
CHB6	26.79	1.53	1.53	0.06	1.88	3.48	3.24	0.24
AHB21	22.49	-0.33	0.56	0.02	0.73	1.89	1.85	0.04
IL16	109.04	0.75	0.82	0.01	0.97	1.66	1.64	0.02
PNICO23	4.27	0.14	0.34	0.08	0.42	1.10	1.09	0.01
CT20	0.98	0.14	0.14	0.14	0.15	0.31	0.27	0.04
CARBHB12	6.04	1.00	1.00	0.17	1.09	1.73	1.49	0.24
ADIM6	3.36	1.51	1.51	0.45	1.65	3.20	2.57	0.63
3B-69-TRIM	12.30	0.92	0.95	0.08	1.14	2.97	2.96	0.01

Table S48: Statistical analysis for revDOD-BLYP-D4 for all testset in our databsase. The numbers given (all in kcal/mol) are average reaction energy ($|\overline{\Delta E}|$), mean deviation (MD), mean absolute deviation (MAD), MAD normalized with respect to $|\overline{\Delta E}|$ (NMAD), root-mean-square deviation (RMSD), deviation span (Δ_{err}), maximum (max) and minimum deviation (min).

Test set	$\|\overline{\Delta E}\|$	MD	MAD	NMAD	RMSD	Δ_{err}	max	min
FH51	31.01	1.24	1.24	0.04	1.67	5.10	5.08	0.02
YBDE18	49.28	-1.29	2.22	0.05	2.45	5.28	4.68	0.60
AL2X6	35.88	-1.43	1.76	0.05	1.90	3.56	2.75	0.81
DARC	32.47	-0.18	0.79	0.02	0.85	1.41	1.40	0.01
NBPRC	27.71	0.24	1.89	0.07	2.09	3.36	3.18	0.18
HEAVYSB9	58.02	-2.19	2.35	0.04	3.04	7.06	6.61	0.45
BSR36	16.20	0.91	1.14	0.07	1.43	4.68	4.59	0.09
RSE43	7.60	1.13	1.13	0.15	1.69	6.95	6.70	0.25
W4-11	306.91	-0.72	3.37	0.01	4.54	17.09	17.04	0.05
G21EA	33.62	-6.00	6.00	0.18	6.59	12.05	11.41	0.64
G21IP	257.61	-1.06	2.46	0.01	2.92	6.17	6.01	0.16
DIPCS10	654.26	-2.67	3.46	0.01	4.06	7.86	7.77	0.09
PA26	189.05	1.66	1.97	0.01	2.54	5.81	5.72	0.09
SIE4x4	33.72	4.68	4.75	0.14	5.80	13.43	12.94	0.49
ALKBDE10	100.69	-0.08	2.90	0.03	3.89	10.66	9.94	0.72
RC21	35.70	-0.75	1.20	0.03	1.41	2.40	2.40	0.00
ALK8	62.60	-0.20	3.18	0.05	3.97	7.00	6.70	0.30
DC13	54.98	-0.78	4.43	0.08	5.37	12.26	11.50	0.76
G2RC	51.26	-0.68	1.92	0.04	2.34	4.85	4.79	0.06
BH76RC	21.39	-0.27	1.44	0.07	1.74	4.45	4.42	0.03
MOR23	35.57	-3.09	4.01	0.11	5.13	10.60	10.54	0.06
WCPT18	34.99	1.52	1.52	0.04	1.84	3.11	3.04	0.07
BHROT27	6.37	0.16	0.16	0.03	0.20	0.41	0.41	0.00
BHPERI	20.87	0.69	0.69	0.03	0.91	1.95	1.94	0.01
BHDIV10	45.33	1.57	1.57	0.03	1.72	3.49	2.99	0.50
INV24	32.85	1.92	1.92	0.06	2.47	5.72	5.71	0.01

Continued on next page

Test set	$\lvert\overline{\Delta E}\rvert$	MD	MAD	NMAD	RMSD	Δ_{err}	max	min
CR20	19.31	-3.81	3.81	0.20	3.87	7.49	5.13	2.36
CRBH20	46.13	-0.66	0.82	0.02	0.97	1.85	1.73	0.12
TMBH17	12.76	1.03	2.73	0.21	3.64	7.43	7.33	0.10
LTMBH26	9.98	-0.62	1.36	0.14	2.06	7.95	7.86	0.09
BH76	18.61	0.01	1.81	0.10	2.65	8.88	8.88	0.00
ISO34	14.57	0.94	0.94	0.06	1.27	3.10	3.08	0.02
ICONF	3.27	0.18	0.18	0.05	0.21	0.47	0.45	0.02
ACONF	1.83	0.15	0.15	0.08	0.16	0.29	0.26	0.03
TAUT15	3.05	1.01	1.01	0.33	1.20	2.82	2.77	0.05
Amino20x4	2.44	0.17	0.17	0.07	0.22	0.61	0.61	0.00
PCONF	1.62	0.85	0.85	0.52	1.05	1.70	1.68	0.02
MCONF	4.97	0.55	0.55	0.11	0.59	1.02	1.00	0.02
SCONF	4.60	0.46	0.46	0.10	0.57	1.25	1.23	0.02
PArel	4.63	-0.18	0.54	0.12	0.79	1.85	1.84	0.01
BUT14DIOL	2.80	0.69	0.69	0.25	0.71	1.05	0.96	0.09
EIE22	5.44	0.21	0.25	0.05	0.31	0.65	0.63	0.02
Styrene45	62.64	1.33	2.45	0.04	3.12	8.09	8.09	0.00
ISOMERIZATION20	31.84	0.04	0.84	0.03	1.06	2.22	2.22	0.00
DIE60	4.71	0.14	0.33	0.07	0.41	0.98	0.98	0.00
IDISP	14.22	4.04	4.04	0.28	5.98	14.59	13.60	0.99
C20C24	30.77	0.40	21.74	0.71	23.17	47.57	34.69	12.88
S66	5.47	-0.98	0.98	0.18	1.08	2.65	2.56	0.09
S10x8	6.59	0.22	0.31	0.05	0.42	1.44	1.44	0.00
X40	3.76	0.72	0.72	0.19	0.81	2.16	1.91	0.25
HEAVY28	1.24	-0.13	0.24	0.20	0.32	0.88	0.86	0.02
CHB6	26.79	1.89	1.89	0.07	2.26	4.30	3.85	0.45
AHB21	22.49	-0.28	0.52	0.02	0.69	1.76	1.71	0.05
IL16	109.04	0.67	0.74	0.01	0.88	1.48	1.45	0.03
PNICO23	4.27	0.20	0.42	0.10	0.52	1.47	1.41	0.06
CT20	0.98	0.11	0.11	0.11	0.12	0.28	0.26	0.02
CARBHB12	6.04	0.99	0.99	0.16	1.08	1.78	1.52	0.26
ADIM6	3.36	1.79	1.79	0.53	1.94	3.74	2.98	0.76
3B-69-TRIM	12.30	0.76	0.79	0.06	0.96	2.49	2.48	0.01
ISOL24	21.92	0.90	1.59	0.07	2.53	9.47	9.44	0.03
C60ISO	98.25	-1.94	3.27	0.03	4.30	9.87	8.94	0.93
L7	18.20	2.97	2.97	0.16	3.21	6.42	4.69	1.73
UPU23	5.72	0.06	0.77	0.13	1.00	2.72	2.67	0.05
ENZYMES23	15.32	-0.37	1.40	0.09	1.62	3.70	3.48	0.22

Table S49: Statistical analysis for revDSD-PBEP86-D4 for all testset in our databsase. The numbers given (all in kcal/mol) are average reaction energy ($|\overline{\Delta E}|$), mean deviation (MD), mean absolute deviation (MAD), MAD normalized with respect to $|\overline{\Delta E}|$ (NMAD), root-mean-square deviation (RMSD), deviation span (Δ_{err}), maximum (max) and minimum deviation (min).

Test set	$\lvert\overline{\Delta E}\rvert$	MD	MAD	NMAD	RMSD	Δ_{err}	max	min
FH51	31.01	1.16	1.16	0.04	1.59	4.80	4.79	0.01
YBDE18	49.28	-1.54	2.27	0.05	2.67	5.21	5.00	0.21
AL2X6	35.88	-1.31	1.31	0.04	1.58	2.83	2.72	0.11
DARC	32.47	-1.44	1.44	0.04	1.57	2.56	2.30	0.26
NBPRC	27.71	0.39	1.63	0.06	1.86	4.67	4.08	0.59
HEAVYSB9	58.02	-2.45	2.45	0.04	2.99	6.15	6.07	0.08
BSR36	16.20	-0.18	0.37	0.02	0.56	2.03	2.02	0.01
RSE43	7.60	1.28	1.28	0.17	1.89	7.31	7.15	0.16
W4-11	306.91	-9.99	9.99	0.03	11.09	25.84	25.35	0.49
G21EA	33.62	-7.43	7.43	0.22	7.88	15.80	12.97	2.83
G21IP	257.61	-2.63	3.25	0.01	3.63	8.02	7.77	0.25
DIPCS10	654.26	-5.92	5.92	0.01	6.29	12.15	10.08	2.07
PA26	189.05	1.77	1.91	0.01	2.45	5.95	5.86	0.09
SIE4x4	33.72	3.86	3.91	0.12	4.91	10.79	10.75	0.04
ALKBDE10	100.69	-3.52	3.98	0.04	5.19	12.34	11.97	0.37
RC21	35.70	-1.64	1.95	0.05	2.28	4.15	3.83	0.32
ALK8	62.60	0.28	1.35	0.02	1.75	3.97	3.87	0.10
DC13	54.98	0.39	3.36	0.06	4.87	13.14	12.89	0.25
G2RC	51.26	-0.26	1.54	0.03	1.90	4.13	4.11	0.02
BH76RC	21.39	0.12	1.29	0.06	1.66	4.44	4.44	0.00
MOR23	35.57	-1.80	3.47	0.10	4.77	11.82	11.59	0.23
WCPT18	34.99	1.55	1.55	0.04	1.91	4.07	3.89	0.18
BHROT27	6.37	0.16	0.16	0.02	0.18	0.36	0.34	0.02
BHPERI	20.87	0.70	0.70	0.03	0.87	2.36	2.36	0.00
BHDIV10	45.33	1.08	1.08	0.02	1.20	2.13	1.85	0.28
INV24	32.85	1.69	1.69	0.05	2.20	4.90	4.84	0.06
CR20	19.31	-3.26	3.26	0.17	3.31	6.78	4.55	2.23
CRBH20	46.13	0.39	0.65	0.01	0.82	2.09	1.99	0.10
TMBH17	12.76	0.99	2.33	0.18	3.04	6.05	5.81	0.24
LTMBH26	9.98	-0.47	1.28	0.13	1.98	7.51	7.48	0.03
BH76	18.61	0.13	1.57	0.08	2.44	9.14	9.12	0.02
ISO34	14.57	0.52	0.52	0.04	0.76	2.42	2.42	0.00
ICONF	3.27	0.19	0.19	0.06	0.22	0.46	0.43	0.03
ACONF	1.83	0.04	0.04	0.02	0.05	0.09	0.09	0.00
TAUT15	3.05	0.93	0.93	0.30	1.09	2.68	2.61	0.07
Amino20x4	2.44	0.13	0.13	0.05	0.17	0.50	0.50	0.00
PCONF	1.62	0.47	0.47	0.29	0.61	1.21	1.18	0.03
MCONF	4.97	0.44	0.44	0.09	0.48	0.87	0.82	0.05

Continued on next page

Test set	$\|\overline{\Delta E}\|$	MD	MAD	NMAD	RMSD	Δ_{err}	max	min
SCONF	4.60	0.31	0.31	0.07	0.38	0.91	0.87	0.04
PArel	4.63	-0.09	0.55	0.12	0.78	1.96	1.95	0.01
BUT14DIOL	2.80	0.35	0.35	0.13	0.36	0.50	0.45	0.05
EIE22	5.44	0.29	0.30	0.06	0.37	0.85	0.83	0.02
Styrene45	62.64	-0.20	1.07	0.02	1.38	3.65	3.65	0.00
ISOMERIZATION20	31.84	0.03	1.07	0.03	1.38	3.15	3.15	0.00
DIE60	4.71	0.34	0.39	0.08	0.55	1.21	1.21	0.00
IDISP	14.22	3.29	3.29	0.23	6.07	14.69	14.55	0.14
C20C24	30.77	-4.16	15.43	0.50	18.63	37.79	32.71	5.08
S66	5.47	-1.17	1.17	0.21	1.29	3.35	3.12	0.23
S10x8	6.59	0.71	0.71	0.11	0.97	2.73	2.73	0.00
X40	3.76	0.81	0.81	0.21	0.89	2.27	2.05	0.22
HEAVY28	1.24	-0.08	0.20	0.16	0.28	0.82	0.81	0.01
CHB6	26.79	1.97	1.97	0.07	2.16	4.43	3.41	1.02
AHB21	22.49	0.27	0.52	0.02	0.61	1.13	1.11	0.02
IL16	109.04	1.78	1.78	0.02	1.91	3.48	2.88	0.60
PNICO23	4.27	-0.15	0.29	0.07	0.42	1.36	1.35	0.01
CT20	0.98	0.28	0.28	0.29	0.30	0.74	0.61	0.13
CARBHB12	6.04	0.73	0.73	0.12	0.79	1.35	1.14	0.21
ADIM6	3.36	1.10	1.10	0.33	1.17	2.27	1.74	0.53
3B-69-TRIM	12.30	2.00	2.00	0.16	2.17	4.87	4.36	0.51

Table S50: Statistical analysis for revDOD-PBEP86-D4 for all testset in our databsase. The numbers given (all in kcal/mol) are average reaction energy ($|\overline{\Delta E}|$), mean deviation (MD), mean absolute deviation (MAD), MAD normalized with respect to $|\overline{\Delta E}|$ (NMAD), root-mean-square deviation (RMSD), deviation span (Δ_{err}), maximum (max) and minimum deviation (min).

Test set	$\|\overline{\Delta E}\|$	MD	MAD	NMAD	RMSD	Δ_{err}	max	min
FH51	31.01	1.27	1.27	0.04	1.73	5.66	5.64	0.02
YBDE18	49.28	-1.48	2.18	0.04	2.50	4.85	4.65	0.20
AL2X6	35.88	-1.28	1.28	0.04	1.57	2.69	2.68	0.01
DARC	32.47	-1.64	1.64	0.05	1.76	2.90	2.47	0.43
NBPRC	27.71	0.37	1.68	0.06	1.91	4.94	4.28	0.66
HEAVYSB9	58.02	-2.43	2.43	0.04	2.99	6.07	6.06	0.01
BSR36	16.20	-0.42	0.49	0.03	0.63	1.40	1.39	0.01
RSE43	7.60	1.33	1.33	0.18	1.93	7.54	7.34	0.20
W4-11	306.91	-9.57	9.57	0.03	10.61	26.61	25.39	1.22
G21EA	33.62	-7.33	7.33	0.22	7.80	15.31	12.68	2.63
G21IP	257.61	-2.46	3.14	0.01	3.50	7.32	7.13	0.19
DIPCS10	654.26	-5.54	5.54	0.01	5.99	11.31	9.83	1.48
PA26	189.05	2.02	2.14	0.01	2.66	6.14	6.04	0.10
SIE4x4	33.72	3.79	3.85	0.11	4.87	11.05	10.98	0.07

Continued on next page

Test set	$\lvert\overline{\Delta E}\rvert$	MD	MAD	NMAD	RMSD	Δ_{err}	max	min
ALKBDE10	100.69	-3.76	4.01	0.04	5.22	12.33	12.14	0.19
RC21	35.70	-1.57	1.90	0.05	2.18	4.02	3.64	0.38
ALK8	62.60	0.28	1.47	0.02	1.90	4.16	4.13	0.03
DC13	54.98	0.04	3.28	0.06	4.70	11.62	11.22	0.40
G2RC	51.26	-0.23	1.59	0.03	1.94	3.88	3.84	0.04
BH76RC	21.39	0.12	1.32	0.06	1.67	4.38	4.37	0.01
MOR23	35.57	-1.54	3.13	0.09	4.27	10.16	10.11	0.05
WCPT18	34.99	1.44	1.44	0.04	1.76	3.60	3.58	0.02
BHROT27	6.37	0.16	0.16	0.02	0.19	0.38	0.37	0.01
BHPERI	20.87	0.74	0.74	0.04	0.90	1.93	1.87	0.06
BHDIV10	45.33	1.05	1.05	0.02	1.19	1.74	1.68	0.06
INV24	32.85	1.67	1.67	0.05	2.21	5.41	5.28	0.13
CR20	19.31	-3.02	3.02	0.16	3.08	6.27	4.29	1.98
CRBH20	46.13	0.58	0.71	0.02	0.94	2.23	2.22	0.01
TMBH17	12.76	1.21	2.44	0.19	3.18	6.15	5.88	0.27
LTMBH26	9.98	-0.38	1.23	0.12	1.99	7.95	7.92	0.03
BH76	18.61	0.23	1.58	0.08	2.46	9.30	9.28	0.02
ISO34	14.57	0.52	0.52	0.04	0.76	2.60	2.59	0.01
ICONF	3.27	0.18	0.18	0.06	0.21	0.42	0.39	0.03
ACONF	1.83	0.06	0.06	0.03	0.06	0.11	0.11	0.00
TAUT15	3.05	1.02	1.02	0.33	1.18	2.77	2.69	0.08
Amino20x4	2.44	0.14	0.14	0.06	0.18	0.51	0.51	0.00
PCONF	1.62	0.53	0.53	0.33	0.67	1.24	1.23	0.01
MCONF	4.97	0.45	0.45	0.09	0.49	0.87	0.84	0.03
SCONF	4.60	0.30	0.30	0.06	0.37	0.87	0.86	0.01
PArel	4.63	-0.12	0.55	0.12	0.77	1.87	1.83	0.04
BUT14DIOL	2.80	0.35	0.35	0.12	0.35	0.50	0.45	0.05
EIE22	5.44	0.23	0.25	0.05	0.32	0.73	0.72	0.01
Styrene45	62.64	-0.45	1.01	0.02	1.36	3.72	3.72	0.00
ISOMERIZATION20	31.84	-0.10	0.92	0.03	1.14	2.19	2.19	0.00
DIE60	4.71	0.28	0.38	0.08	0.53	1.23	1.22	0.01
IDISP	14.22	3.24	3.24	0.23	5.96	14.36	14.26	0.10
C20C24	30.77	-4.20	14.85	0.48	17.98	37.08	31.77	5.31
S66	5.47	-1.15	1.15	0.21	1.27	3.30	3.07	0.23
S10x8	6.59	0.71	0.72	0.11	0.98	2.70	2.70	0.00
X40	3.76	0.81	0.81	0.22	0.90	2.25	2.03	0.22
HEAVY28	1.24	-0.09	0.21	0.17	0.28	0.80	0.80	0.00
CHB6	26.79	1.97	1.97	0.07	2.18	4.48	3.48	1.00
AHB21	22.49	0.30	0.54	0.02	0.62	1.16	1.10	0.06
IL16	109.04	1.80	1.80	0.02	1.92	3.54	2.89	0.65
PNICO23	4.27	-0.15	0.30	0.07	0.45	1.52	1.50	0.02
CT20	0.98	0.28	0.28	0.29	0.30	0.75	0.63	0.12
CARBHB12	6.04	0.71	0.71	0.12	0.77	1.31	1.13	0.18
ADIM6	3.36	1.15	1.15	0.34	1.23	2.38	1.82	0.56

Continued on next page

Test set	$\|\overline{\Delta E}\|$	MD	MAD	NMAD	RMSD	Δ_{err}	max	min
3B-69-TRIM	12.30	1.98	1.98	0.16	2.14	4.74	4.25	0.49
ISOL24	21.92	0.21	0.97	0.04	1.23	4.04	3.91	0.13
C60ISO	98.25	-2.93	3.85	0.04	5.14	11.20	10.57	0.63
L7	18.20	5.27	5.27	0.29	5.65	11.20	8.79	2.41
UPU23	5.72	0.23	0.59	0.10	0.74	1.54	1.51	0.03
ENZYMES23	15.32	-0.51	1.27	0.08	1.75	5.69	5.63	0.06

Table S51: Statistical analysis for revDSD-PBE-D4 for all testset in our databsase. The numbers given (all in kcal/mol) are average reaction energy ($|\overline{\Delta E}|$), mean deviation (MD), mean absolute deviation (MAD), MAD normalized with respect to $|\overline{\Delta E}|$ (NMAD), root-mean-square deviation (RMSD), deviation span (Δ_{err}), maximum (max) and minimum deviation (min).

Test set	$\|\overline{\Delta E}\|$	MD	MAD	NMAD	RMSD	Δ_{err}	max	min
FH51	31.01	1.66	1.66	0.05	2.20	7.87	7.84	0.03
YBDE18	49.28	0.60	1.67	0.03	2.21	7.29	6.84	0.45
AL2X6	35.88	0.33	0.88	0.02	1.00	2.27	1.80	0.47
DARC	32.47	-4.77	4.77	0.15	4.83	9.34	5.88	3.46
NBPRC	27.71	-0.75	1.76	0.06	2.03	3.52	3.29	0.23
HEAVYSB9	58.02	-0.65	1.35	0.02	1.79	4.16	4.15	0.01
BSR36	16.20	1.46	1.53	0.09	1.99	6.47	6.28	0.19
RSE43	7.60	1.21	1.21	0.16	1.83	7.10	7.00	0.10
W4-11	306.91	-6.18	6.19	0.02	7.08	19.69	19.60	0.09
G21EA	33.62	-7.35	7.35	0.22	7.78	15.71	12.69	3.02
G21IP	257.61	-2.69	3.24	0.01	3.62	8.02	7.90	0.12
DIPCS10	654.26	-5.99	5.99	0.01	6.22	11.63	9.10	2.53
PA26	189.05	1.56	1.71	0.01	2.21	5.37	5.37	0.00
SIE4x4	33.72	4.23	4.27	0.13	5.19	11.20	10.92	0.28
ALKBDE10	100.69	-2.13	3.50	0.03	4.47	10.76	10.31	0.45
RC21	35.70	-0.41	1.56	0.04	1.96	4.64	4.56	0.08
ALK8	62.60	1.57	1.67	0.03	2.59	7.12	6.74	0.38
DC13	54.98	-0.80	2.54	0.05	3.33	7.33	7.14	0.19
G2RC	51.26	-0.77	2.34	0.05	2.74	5.76	5.63	0.13
BH76RC	21.39	0.14	1.49	0.07	1.88	5.08	4.96	0.12
MOR23	35.57	-4.02	5.19	0.15	6.41	13.07	12.88	0.19
WCPT18	34.99	2.38	2.38	0.07	2.96	5.74	5.60	0.14
BHROT27	6.37	0.15	0.15	0.02	0.17	0.31	0.31	0.00
BHPERI	20.87	2.14	2.14	0.10	2.56	5.28	5.25	0.03
BHDIV10	45.33	1.23	1.23	0.03	1.42	2.88	2.51	0.37
INV24	32.85	1.59	1.59	0.05	2.05	4.33	4.32	0.01
CR20	19.31	-0.55	0.64	0.03	0.80	2.11	2.01	0.10
CRBH20	46.13	1.77	1.77	0.04	1.90	3.69	3.16	0.53
TMBH17	12.76	0.15	2.09	0.16	2.67	5.13	5.06	0.07

Continued on next page

Test set	$\|\overline{\Delta E}\|$	MD	MAD	NMAD	RMSD	Δ_{err}	max	min
LTMBH26	9.98	-0.71	1.38	0.14	1.96	7.11	7.10	0.01
BH76	18.61	-0.46	1.83	0.10	2.55	9.35	9.21	0.14
ISO34	14.57	0.60	0.60	0.04	0.80	2.24	2.23	0.01
ICONF	3.27	0.18	0.18	0.05	0.21	0.43	0.42	0.01
ACONF	1.83	0.23	0.23	0.12	0.25	0.48	0.42	0.06
TAUT15	3.05	0.80	0.80	0.26	1.02	2.56	2.48	0.08
Amino20x4	2.44	0.21	0.21	0.08	0.25	0.67	0.67	0.00
PCONF	1.62	0.91	0.91	0.56	1.18	1.98	1.96	0.02
MCONF	4.97	0.79	0.79	0.16	0.86	1.43	1.33	0.10
SCONF	4.60	0.66	0.66	0.14	0.77	1.64	1.60	0.04
PArel	4.63	-0.02	0.68	0.15	0.96	2.52	2.49	0.03
BUT14DIOL	2.80	0.53	0.53	0.19	0.55	0.76	0.73	0.03
EIE22	5.44	0.29	0.31	0.06	0.38	0.84	0.84	0.00
Styrene45	62.64	-0.48	0.77	0.01	1.00	2.32	2.32	0.00
ISOMERIZATION20	31.84	-0.07	1.05	0.03	1.33	2.77	2.77	0.00
DIE60	4.71	0.34	0.36	0.08	0.51	1.11	1.11	0.00
IDISP	14.22	6.09	6.09	0.43	8.81	21.25	19.97	1.28
C20C24	30.77	-6.01	8.24	0.27	11.07	23.80	20.95	2.85
S66	5.47	-0.71	0.71	0.13	0.80	1.97	1.96	0.01
S10x8	6.59	0.37	0.40	0.06	0.54	1.20	1.20	0.00
X40	3.76	0.51	0.51	0.14	0.55	1.39	1.22	0.17
HEAVY28	1.24	0.09	0.22	0.17	0.28	0.78	0.77	0.01
CHB6	26.79	1.84	1.84	0.07	2.03	4.21	3.26	0.95
AHB21	22.49	-0.16	0.48	0.02	0.60	1.63	1.56	0.07
IL16	109.04	0.80	0.90	0.01	1.05	1.74	1.70	0.04
PNICO23	4.27	0.32	0.34	0.08	0.39	0.88	0.86	0.02
CT20	0.98	0.20	0.20	0.21	0.22	0.47	0.40	0.07
CARBHB12	6.04	1.05	1.05	0.17	1.13	1.92	1.58	0.34
ADIM6	3.36	1.68	1.68	0.50	1.81	3.49	2.75	0.74
3B-69-TRIM	12.30	1.15	1.16	0.09	1.28	2.95	2.69	0.26

Table S52: Statistical analysis for revDOD-PBE-D4 for all testset in our databsase. The numbers given (all in kcal/mol) are average reaction energy ($|\overline{\Delta E}|$), mean deviation (MD), mean absolute deviation (MAD), MAD normalized with respect to $|\overline{\Delta E}|$ (NMAD), root-mean-square deviation (RMSD), deviation span (Δ_{err}), maximum (max) and minimum deviation (min).

Test set	$\|\overline{\Delta E}\|$	MD	MAD	NMAD	RMSD	Δ_{err}	max	min
FH51	31.01	1.75	1.75	0.06	2.31	8.45	8.42	0.03
YBDE18	49.28	0.72	1.54	0.03	2.15	6.83	6.71	0.12
AL2X6	35.88	0.39	0.91	0.03	1.04	2.40	1.90	0.50
DARC	32.47	-4.96	4.96	0.15	5.02	9.67	6.07	3.60
NBPRC	27.71	-0.79	1.85	0.07	2.12	3.77	3.53	0.24

Continued on next page

Test set	$\|\overline{\Delta E}\|$	MD	MAD	NMAD	RMSD	Δ_{err}	max	min
HEAVYSB9	58.02	-0.56	1.37	0.02	1.78	4.23	4.07	0.16
BSR36	16.20	1.36	1.44	0.09	1.88	6.06	5.97	0.09
RSE43	7.60	1.25	1.25	0.16	1.86	7.28	7.15	0.13
W4-11	306.91	-5.68	5.70	0.02	6.54	19.59	19.42	0.17
G21EA	33.62	-7.26	7.26	0.22	7.70	15.40	12.45	2.95
G21IP	257.61	-2.56	3.16	0.01	3.53	7.53	7.45	0.08
DIPCS10	654.26	-5.73	5.73	0.01	5.98	11.01	8.86	2.15
PA26	189.05	1.71	1.85	0.01	2.33	5.48	5.47	0.01
SIE4x4	33.72	4.21	4.25	0.13	5.18	11.37	11.09	0.28
ALKBDE10	100.69	-2.17	3.38	0.03	4.38	10.89	10.32	0.57
RC21	35.70	-0.36	1.52	0.04	1.90	4.71	4.64	0.07
ALK8	62.60	1.60	1.70	0.03	2.67	7.37	6.98	0.39
DC13	54.98	-1.04	2.50	0.05	3.32	7.56	7.36	0.20
G2RC	51.26	-0.74	2.38	0.05	2.76	5.57	5.52	0.05
BH76RC	21.39	0.14	1.47	0.07	1.86	5.09	4.93	0.16
MOR23	35.57	-3.92	5.01	0.14	6.15	12.28	12.19	0.09
WCPT18	34.99	2.29	2.29	0.07	2.86	5.46	5.46	0.00
BHROT27	6.37	0.15	0.15	0.02	0.17	0.33	0.32	0.01
BHPERI	20.87	2.00	2.00	0.10	2.43	5.07	5.03	0.04
BHDIV10	45.33	1.15	1.15	0.03	1.32	2.87	2.43	0.44
INV24	32.85	1.57	1.57	0.05	2.05	4.61	4.60	0.01
CR20	19.31	-0.34	0.51	0.03	0.67	1.86	1.80	0.06
CRBH20	46.13	1.91	1.91	0.04	2.04	3.97	3.33	0.64
TMBH17	12.76	0.26	2.10	0.16	2.70	5.19	5.18	0.01
LTMBH26	9.98	-0.66	1.35	0.14	1.96	7.34	7.32	0.02
BH76	18.61	-0.42	1.79	0.10	2.54	9.41	9.37	0.04
ISO34	14.57	0.61	0.61	0.04	0.83	2.37	2.36	0.01
ICONF	3.27	0.17	0.17	0.05	0.21	0.41	0.40	0.01
ACONF	1.83	0.24	0.24	0.13	0.26	0.50	0.44	0.06
TAUT15	3.05	0.86	0.86	0.28	1.07	2.62	2.54	0.08
Amino20x4	2.44	0.21	0.21	0.09	0.26	0.68	0.68	0.00
PCONF	1.62	0.97	0.97	0.60	1.24	2.05	2.02	0.03
MCONF	4.97	0.81	0.81	0.16	0.88	1.43	1.34	0.09
SCONF	4.60	0.66	0.66	0.14	0.77	1.66	1.62	0.04
PArel	4.63	-0.04	0.68	0.15	0.95	2.49	2.48	0.01
BUT14DIOL	2.80	0.53	0.53	0.19	0.55	0.77	0.74	0.03
EIE22	5.44	0.25	0.27	0.05	0.34	0.77	0.77	0.00
Styrene45	62.64	-0.66	0.87	0.01	1.08	2.37	2.37	0.00
ISOMERIZATION20	31.84	0.42	1.39	0.04	2.25	8.31	8.31	0.00
DIE60	4.71	0.30	0.35	0.07	0.50	1.11	1.11	0.00
IDISP	14.22	6.15	6.15	0.43	8.84	21.30	19.97	1.33
C20C24	30.77	-6.08	7.70	0.25	10.56	22.41	20.08	2.33
S66	5.47	-0.69	0.69	0.13	0.78	1.90	1.89	0.01
S10x8	6.59	0.37	0.39	0.06	0.54	1.23	1.23	0.00

Continued on next page

Test set	$\|\overline{\Delta E}\|$	MD	MAD	NMAD	RMSD	Δ_{err}	max	min
X40	3.76	0.51	0.51	0.13	0.55	1.35	1.19	0.16
HEAVY28	1.24	0.10	0.22	0.18	0.28	0.76	0.75	0.01
CHB6	26.79	1.85	1.85	0.07	2.05	4.24	3.30	0.94
AHB21	22.49	-0.14	0.47	0.02	0.58	1.60	1.52	0.08
IL16	109.04	0.79	0.89	0.01	1.03	1.72	1.69	0.03
PNICO23	4.27	0.33	0.35	0.08	0.41	0.92	0.88	0.04
CT20	0.98	0.20	0.20	0.21	0.22	0.47	0.40	0.07
CARBHB12	6.04	1.04	1.04	0.17	1.11	1.87	1.53	0.34
ADIM6	3.36	1.74	1.74	0.52	1.88	3.62	2.85	0.77
3B-69-TRIM	12.30	1.12	1.13	0.09	1.25	2.80	2.57	0.23
ISOL24	21.92	1.28	2.00	0.09	3.44	13.77	13.65	0.12
C60ISO	98.25	-3.80	4.44	0.05	6.04	12.45	12.24	0.21
L7	18.20	2.62	2.62	0.14	2.76	5.20	3.73	1.47
UPU23	5.72	-0.01	0.75	0.13	0.97	2.63	2.62	0.01
ENZYMES23	15.32	-0.43	1.40	0.09	1.67	4.09	3.93	0.16

Table S53: Statistical analysis for revDSD-SCAN-D4 for all testset in our databsase. The numbers given (all in kcal/mol) are average reaction energy ($|\overline{\Delta E}|$), mean deviation (MD), mean absolute deviation (MAD), MAD normalized with respect to $|\overline{\Delta E}|$ (NMAD), root-mean-square deviation (RMSD), deviation span (Δ_{err}), maximum (max) and minimum deviation (min).

Test set	$\|\overline{\Delta E}\|$	MD	MAD	NMAD	RMSD	Δ_{err}	max	min
FH51	31.01	2.52	2.52	0.08	3.37	12.23	12.23	0.00
YBDE18	49.28	1.62	2.49	0.05	3.22	9.22	8.80	0.42
AL2X6	35.88	2.63	2.63	0.07	3.11	5.94	5.25	0.69
DARC	32.47	-7.48	7.48	0.23	7.63	13.99	9.39	4.60
NBPRC	27.71	-1.99	2.92	0.11	3.33	8.02	6.78	1.24
HEAVYSB9	58.02	0.72	1.85	0.03	2.43	4.68	4.53	0.15
BSR36	16.20	4.54	4.58	0.28	5.37	14.36	13.86	0.50
RSE43	7.60	1.70	1.70	0.22	2.31	9.63	9.14	0.49
W4-11	306.91	-4.37	4.53	0.01	5.23	15.73	15.07	0.66
G21EA	33.62	2.56	3.61	0.11	4.11	8.08	7.43	0.65
G21IP	257.61	-1.66	2.56	0.01	2.99	7.55	7.44	0.11
DIPCS10	654.26	-4.13	4.21	0.01	4.78	8.30	7.88	0.42
PA26	189.05	1.92	2.15	0.01	2.65	6.31	6.25	0.06
SIE4x4	33.72	0.68	5.00	0.15	8.25	26.56	25.95	0.61
ALKBDE10	100.69	-1.33	3.51	0.03	4.52	9.12	9.10	0.02
RC21	35.70	0.24	1.67	0.05	2.22	6.80	6.74	0.06
ALK8	62.60	4.81	4.84	0.08	7.39	19.07	18.94	0.13
DC13	54.98	-1.95	3.33	0.06	4.86	15.39	14.67	0.72
G2RC	51.26	-1.25	2.77	0.05	3.24	7.66	7.40	0.26
BH76RC	21.39	-0.16	1.33	0.06	1.69	5.17	4.79	0.38

Continued on next page

Test set	$\|\overline{\Delta E}\|$	MD	MAD	NMAD	RMSD	Δ_{err}	max	min
MOR23	35.57	-6.85	7.97	0.22	9.29	16.67	16.51	0.16
WCPT18	34.99	2.82	2.82	0.08	3.58	6.77	6.52	0.25
BHROT27	6.37	0.20	0.20	0.03	0.25	0.61	0.58	0.03
BHPERI	20.87	3.56	3.56	0.17	4.17	9.08	8.93	0.15
BHDIV10	45.33	1.45	1.45	0.03	1.69	3.42	3.22	0.20
INV24	32.85	1.47	1.47	0.04	1.90	3.68	3.67	0.01
CR20	19.31	0.46	0.75	0.04	0.97	2.55	2.49	0.06
CRBH20	46.13	1.21	1.21	0.03	1.32	2.50	2.25	0.25
TMBH17	12.76	-0.60	2.60	0.20	3.07	5.77	5.57	0.20
LTMBH26	9.98	-1.00	1.71	0.17	2.19	6.94	6.80	0.14
BH76	18.61	-0.99	2.07	0.11	2.71	8.16	7.89	0.27
ISO34	14.57	0.85	0.85	0.06	1.20	4.44	4.37	0.07
ICONF	3.27	0.27	0.27	0.08	0.35	0.67	0.67	0.00
ACONF	1.83	0.62	0.62	0.34	0.68	1.34	1.15	0.19
TAUT15	3.05	0.95	0.95	0.31	1.15	2.52	2.50	0.02
Amino20x4	2.44	0.41	0.41	0.17	0.49	1.26	1.26	0.00
PCONF	1.62	2.01	2.01	1.24	2.50	4.00	3.96	0.04
MCONF	4.97	1.51	1.51	0.30	1.72	2.62	2.56	0.06
SCONF	4.60	1.08	1.08	0.24	1.22	2.48	2.39	0.09
PArel	4.63	0.07	0.78	0.17	1.05	2.67	2.62	0.05
BUT14DIOL	2.80	0.69	0.71	0.25	0.76	1.17	1.15	0.02
EIE22	5.44	0.31	0.32	0.06	0.40	0.96	0.95	0.01
Styrene45	62.64	-0.25	0.80	0.01	1.06	2.92	2.92	0.00
ISOMERIZATION20	31.84	-0.36	1.16	0.04	1.39	2.80	2.76	0.04
DIE60	4.71	0.27	0.34	0.07	0.46	1.35	1.34	0.01
IDISP	14.22	8.24	8.24	0.58	9.13	17.04	14.39	2.65
C20C24	30.77	-6.31	7.16	0.23	9.51	18.06	17.57	0.49
S66	5.47	-0.36	0.36	0.07	0.41	0.90	0.85	0.05
S10x8	6.59	-0.21	0.23	0.04	0.34	1.51	1.50	0.01
X40	3.76	0.21	0.21	0.05	0.30	1.05	1.05	0.00
HEAVY28	1.24	0.52	0.53	0.43	0.59	1.13	1.12	0.01
CHB6	26.79	0.32	0.75	0.03	0.77	1.32	0.93	0.39
AHB21	22.49	-0.67	0.72	0.03	0.95	2.40	2.33	0.07
IL16	109.04	-0.07	0.35	0.00	0.38	0.63	0.60	0.03
PNICO23	4.27	0.83	0.83	0.19	0.88	1.90	1.56	0.34
CT20	0.98	-0.06	0.07	0.07	0.09	0.19	0.18	0.01
CARBHB12	6.04	1.46	1.46	0.24	1.58	2.70	2.22	0.48
ADIM6	3.36	3.19	3.19	0.95	3.48	6.69	5.37	1.32
3B-69-TRIM	12.30	-0.57	0.57	0.05	0.69	1.35	1.34	0.01

Table S54: Statistical analysis for revDOD-SCAN-D4 for all testset in our databsase. The numbers given (all in kcal/mol) are average reaction energy ($|\overline{\Delta E}|$), mean deviation (MD), mean absolute deviation (MAD), MAD normalized with respect to $|\overline{\Delta E}|$ (NMAD), root-mean-square deviation (RMSD), deviation span (Δ_{err}), maximum (max) and minimum deviation (min).

Test set	$\lvert\overline{\Delta E}\rvert$	MD	MAD	NMAD	RMSD	Δ_{err}	max	min
FH51	31.01	2.57	2.57	0.08	3.44	12.59	12.56	0.03
YBDE18	49.28	1.70	2.48	0.05	3.23	9.07	8.75	0.32
AL2X6	35.88	2.69	2.69	0.07	3.17	6.06	5.34	0.72
DARC	32.47	-7.63	7.63	0.24	7.79	14.28	9.55	4.73
NBPRC	27.71	-2.03	2.99	0.11	3.40	8.26	6.97	1.29
HEAVYSB9	58.02	0.76	1.88	0.03	2.45	4.79	4.59	0.20
BSR36	16.20	4.55	4.59	0.28	5.38	14.34	13.86	0.48
RSE43	7.60	1.71	1.71	0.22	2.31	9.64	9.14	0.50
W4-11	306.91	-4.09	4.27	0.01	4.97	15.23	15.00	0.23
G21EA	33.62	-6.43	6.43	0.19	7.01	12.56	11.50	1.06
G21IP	257.61	-1.56	2.50	0.01	2.93	7.29	7.27	0.02
DIPCS10	654.26	-3.94	4.07	0.01	4.62	8.32	7.68	0.64
PA26	189.05	2.11	2.20	0.01	2.71	6.36	6.26	0.10
SIE4x4	33.72	3.06	3.06	0.09	3.57	7.65	7.03	0.62
ALKBDE10	100.69	-1.17	3.51	0.03	4.47	9.50	9.13	0.37
RC21	35.70	0.31	1.67	0.05	2.23	6.96	6.86	0.10
ALK8	62.60	4.90	4.93	0.08	7.52	19.40	19.28	0.12
DC13	54.98	-2.07	3.41	0.06	4.94	15.75	14.89	0.86
G2RC	51.26	-1.29	2.80	0.05	3.28	7.66	7.53	0.13
BH76RC	21.39	-0.19	1.36	0.06	1.72	5.19	4.80	0.39
MOR23	35.57	-6.86	7.95	0.22	9.25	16.69	16.60	0.09
WCPT18	34.99	2.81	2.81	0.08	3.57	6.72	6.50	0.22
BHROT27	6.37	0.20	0.20	0.03	0.25	0.59	0.56	0.03
BHPERI	20.87	3.54	3.54	0.17	4.16	9.00	8.92	0.08
BHDIV10	45.33	1.45	1.45	0.03	1.67	3.49	3.23	0.26
INV24	32.85	1.46	1.46	0.04	1.89	3.72	3.70	0.02
CR20	19.31	0.58	0.82	0.04	1.03	2.64	2.61	0.03
CRBH20	46.13	1.29	1.29	0.03	1.40	2.66	2.34	0.32
TMBH17	12.76	-0.57	2.57	0.20	3.06	5.78	5.59	0.19
LTMBH26	9.98	-0.97	1.70	0.17	2.19	6.97	6.89	0.08
BH76	18.61	-1.00	2.07	0.11	2.71	8.10	7.85	0.25
ISO34	14.57	0.85	0.85	0.06	1.22	4.48	4.43	0.05
ICONF	3.27	0.28	0.28	0.09	0.35	0.68	0.68	0.00
ACONF	1.83	0.63	0.63	0.34	0.70	1.37	1.17	0.20
TAUT15	3.05	0.96	0.96	0.32	1.17	2.51	2.50	0.01
Amino20x4	2.44	0.41	0.41	0.17	0.50	1.27	1.27	0.00
PCONF	1.62	2.04	2.04	1.26	2.53	4.07	4.03	0.04
MCONF	4.97	1.52	1.52	0.31	1.73	2.64	2.58	0.06

Continued on next page

Test set	$\|\overline{\Delta E}\|$	MD	MAD	NMAD	RMSD	Δ_{err}	max	min
SCONF	4.60	1.09	1.09	0.24	1.23	2.52	2.42	0.10
PArel	4.63	0.07	0.78	0.17	1.05	2.69	2.63	0.06
BUT14DIOL	2.80	0.70	0.71	0.26	0.77	1.17	1.16	0.01
EIE22	5.44	0.30	0.31	0.06	0.39	0.93	0.93	0.00
Styrene45	62.64	-0.30	0.82	0.01	1.10	2.86	2.86	0.00
ISOMERIZATION20	31.84	-0.35	1.14	0.04	1.37	2.78	2.74	0.04
DIE60	4.71	0.26	0.34	0.07	0.46	1.35	1.35	0.00
IDISP	14.22	8.38	8.38	0.59	9.29	17.35	14.66	2.69
C20C24	30.77	-6.40	6.91	0.22	9.28	17.13	17.08	0.05
S66	5.47	-0.38	0.38	0.07	0.44	0.97	0.91	0.06
S10x8	6.59	-0.23	0.25	0.04	0.36	1.58	1.58	0.00
X40	3.76	0.22	0.22	0.06	0.32	1.10	1.10	0.00
HEAVY28	1.24	0.53	0.54	0.43	0.60	1.14	1.12	0.02
CHB6	26.79	0.27	0.76	0.03	0.78	1.47	1.00	0.47
AHB21	22.49	-0.69	0.73	0.03	0.96	2.41	2.35	0.06
IL16	109.04	-0.11	0.34	0.00	0.39	0.70	0.63	0.07
PNICO23	4.27	0.85	0.85	0.20	0.90	1.95	1.60	0.35
CT20	0.98	-0.07	0.08	0.08	0.10	0.19	0.19	0.00
CARBHB12	6.04	1.47	1.47	0.24	1.59	2.73	2.24	0.49
ADIM6	3.36	3.24	3.24	0.96	3.52	6.78	5.44	1.34
3B-69-TRIM	12.30	-0.62	0.63	0.05	0.74	1.42	1.42	0.00
ISOL24	21.92	2.32	3.44	0.16	6.38	26.59	26.45	0.14
C60ISO	98.25	-4.68	5.09	0.05	7.00	14.23	13.95	0.28
L7	18.20	-2.30	2.30	0.13	2.93	5.55	5.40	0.15
UPU23	5.72	-0.45	1.29	0.23	1.78	5.39	5.32	0.07
ENZYMES23	15.32	-0.68	1.93	0.13	2.27	4.56	4.36	0.20

S5.3 Dispersion-Uncorrected Double Hybrids

Table S55: Statistical analysis for B2-PLYP for all testset in our databsase. The numbers given (all in kcal/mol) are average reaction energy ($|\overline{\Delta E}|$), mean deviation (MD), mean absolute deviation (MAD), MAD normalized with respect to $|\overline{\Delta E}|$ (NMAD), root-mean-square deviation (RMSD), deviation span (Δ_{err}), maximum (max) and minimum deviation (min).

Test set	$\|\overline{\Delta E}\|$	MD	MAD	NMAD	RMSD	Δ_{err}	max	min
FH51	31.01	1.89	1.89	0.06	2.48	7.83	7.71	0.12
YBDE18	49.28	-3.91	4.56	0.09	4.92	10.14	8.02	2.12
AL2X6	35.88	-4.50	4.50	0.13	4.86	9.43	6.95	2.48
DARC	32.47	6.96	6.96	0.21	7.04	12.86	8.30	4.56
NBPRC	27.71	2.20	3.26	0.12	4.40	10.50	10.00	0.50
HEAVYSB9	58.02	-4.19	4.19	0.07	4.52	10.07	7.58	2.49

Continued on next page

Test set	$\lvert\overline{\Delta E}\rvert$	MD	MAD	NMAD	RMSD	Δ_{err}	max	min
BSR36	16.20	-3.78	3.78	0.23	4.16	9.60	8.05	1.55
RSE43	7.60	-0.46	0.54	0.07	0.70	1.82	1.81	0.01
W4-11	306.91	-3.39	3.69	0.01	4.67	15.99	15.83	0.16
G21EA	33.62	-5.65	5.80	0.17	6.11	12.23	10.32	1.91
G21IP	257.61	-1.17	2.60	0.01	3.04	7.75	7.70	0.05
DIPCS10	654.26	-3.32	3.83	0.01	4.44	10.93	9.05	1.88
PA26	189.05	1.64	1.97	0.01	2.79	7.27	7.21	0.06
SIE4x4	33.72	9.29	9.29	0.28	10.85	21.69	21.40	0.29
ALKBDE10	100.69	0.41	3.66	0.04	4.78	9.19	9.19	0.00
RC21	35.70	-1.26	1.63	0.05	1.97	4.99	4.83	0.16
ALK8	62.60	-2.72	2.97	0.05	4.37	9.81	9.55	0.26
DC13	54.98	3.26	9.62	0.18	12.95	28.77	28.16	0.61
G2RC	51.26	0.16	1.86	0.04	2.43	6.13	6.00	0.13
BH76RC	21.39	-0.38	1.06	0.05	1.45	4.48	4.39	0.09
MOR23	35.57	1.60	4.17	0.12	5.57	12.62	12.59	0.03
WCPT18	34.99	1.94	1.94	0.06	2.38	4.47	4.47	0.00
BHROT27	6.37	0.25	0.25	0.04	0.35	1.09	1.08	0.01
BHPERI	20.87	1.35	1.35	0.06	1.62	4.62	4.41	0.21
BHDIV10	45.33	2.08	2.08	0.05	2.59	4.81	4.62	0.19
INV24	32.85	1.50	1.50	0.05	1.92	4.44	4.44	0.00
CR20	19.31	-8.06	8.06	0.42	8.12	15.64	9.94	5.70
CRBH20	46.13	-4.97	4.97	0.11	5.02	10.19	6.44	3.75
TMBH17	12.76	0.60	2.97	0.23	4.18	11.55	11.48	0.07
LTMBH26	9.98	-1.40	2.27	0.23	3.32	10.61	10.60	0.01
BH76	18.61	-2.64	2.90	0.16	3.41	7.34	7.21	0.13
ISO34	14.57	1.38	1.38	0.09	2.08	8.05	8.05	0.00
ICONF	3.27	0.41	0.41	0.12	0.49	1.16	1.09	0.07
ACONF	1.83	0.38	0.38	0.21	0.42	0.85	0.72	0.13
TAUT15	3.05	0.81	0.81	0.27	0.96	1.84	1.83	0.01
Amino20x4	2.44	0.28	0.28	0.11	0.35	0.97	0.97	0.00
PCONF	1.62	1.46	1.46	0.90	1.64	3.04	2.88	0.16
MCONF	4.97	0.75	0.75	0.15	0.81	1.45	1.33	0.12
SCONF	4.60	3.28	3.28	0.71	3.60	6.92	6.82	0.10
PArel	4.63	-0.03	0.74	0.16	1.13	2.89	2.88	0.01
BUT14DIOL	2.80	0.30	0.30	0.11	0.33	0.72	0.63	0.09
EIE22	5.44	0.98	0.98	0.18	1.07	2.09	2.02	0.07
Styrene45	62.64	2.83	4.11	0.07	5.27	14.21	14.21	0.00
ISOMERIZATION20	31.84	-0.41	1.47	0.05	2.03	4.54	4.45	0.09
DIE60	4.71	0.86	0.86	0.18	0.93	1.83	1.68	0.15
IDISP	14.22	4.56	4.56	0.32	4.89	7.47	6.30	1.17
C20C24	30.77	0.04	37.36	1.21	40.04	83.90	61.61	22.29
S66	5.47	-2.15	2.15	0.39	2.44	6.27	5.82	0.45
S10x8	6.59	1.12	1.12	0.17	1.51	6.05	5.99	0.06
X40	3.76	1.35	1.35	0.36	1.62	4.77	4.56	0.21

Continued on next page

Test set	$\lvert\overline{\Delta E}\rvert$	MD	MAD	NMAD	RMSD	Δ_{err}	max	min
HEAVY28	1.24	-0.62	0.64	0.51	0.71	1.63	1.59	0.04
CHB6	26.79	1.35	1.35	0.05	1.72	3.21	3.15	0.06
AHB21	22.49	-0.16	0.78	0.03	0.94	1.96	1.90	0.06
IL16	109.04	2.28	2.28	0.02	2.43	4.67	3.79	0.88
PNICO23	4.27	-0.97	0.97	0.23	1.08	2.51	2.22	0.29
CT20	0.98	0.47	0.47	0.48	0.49	0.91	0.72	0.19
CARBHB12	6.04	0.42	0.46	0.08	0.62	1.34	1.32	0.02
ADIM6	3.36	-1.66	1.66	0.50	1.81	3.37	2.70	0.67
3B-69-TRIM	12.30	3.88	3.88	0.32	4.27	9.65	8.97	0.68

Table S56: Statistical analysis for B2GP-PLYP for all testset in our databsase. The numbers given (all in kcal/mol) are average reaction energy ($\lvert\overline{\Delta E}\rvert$), mean deviation (MD), mean absolute deviation (MAD), MAD normalized with respect to $\lvert\overline{\Delta E}\rvert$ (NMAD), root-mean-square deviation (RMSD), deviation span (Δ_{err}), maximum (max) and minimum deviation (min).

Test set	$\lvert\overline{\Delta E}\rvert$	MD	MAD	NMAD	RMSD	Δ_{err}	max	min
FH51	31.01	1.27	1.27	0.04	1.71	4.66	4.58	0.08
YBDE18	49.28	-2.39	3.06	0.06	3.11	6.59	4.35	2.24
AL2X6	35.88	-2.86	2.86	0.08	3.29	5.14	4.73	0.41
DARC	32.47	3.00	3.00	0.09	3.09	5.74	4.08	1.66
NBPRC	27.71	1.27	2.52	0.09	3.10	6.66	6.27	0.39
HEAVYSB9	58.02	-3.04	3.04	0.05	3.47	7.20	5.98	1.22
BSR36	16.20	-1.98	1.98	0.12	2.08	4.42	3.55	0.87
RSE43	7.60	0.19	0.40	0.05	0.71	3.31	3.31	0.00
W4-11	306.91	-5.48	5.64	0.02	6.67	20.07	19.98	0.09
G21EA	33.62	-6.18	6.25	0.19	6.63	12.31	11.44	0.87
G21IP	257.61	-1.29	2.38	0.01	2.89	8.35	8.35	0.00
DIPCS10	654.26	-3.49	3.52	0.01	4.52	9.55	9.51	0.04
PA26	189.05	0.48	1.43	0.01	2.03	5.80	5.77	0.03
SIE4x4	33.72	5.97	6.00	0.18	7.11	14.76	14.51	0.25
ALKBDE10	100.69	-0.59	3.46	0.03	4.56	10.15	10.14	0.01
RC21	35.70	-1.14	1.36	0.04	1.69	3.46	3.46	0.00
ALK8	62.60	-1.49	2.30	0.04	3.32	7.23	6.98	0.25
DC13	54.98	2.00	6.55	0.12	9.93	25.05	24.93	0.12
G2RC	51.26	-1.15	2.03	0.04	2.56	8.24	7.90	0.34
BH76RC	21.39	-0.45	0.94	0.04	1.45	4.68	4.67	0.01
MOR23	35.57	-0.47	3.85	0.11	5.20	13.39	13.31	0.08
WCPT18	34.99	1.80	1.80	0.05	2.20	4.29	4.17	0.12
BHROT27	6.37	0.25	0.25	0.04	0.35	1.06	1.06	0.00
BHPERI	20.87	0.68	0.68	0.03	0.89	2.65	2.62	0.03
BHDIV10	45.33	1.70	1.70	0.04	2.10	4.62	4.29	0.33
INV24	32.85	1.73	1.73	0.05	2.08	4.53	4.41	0.12

Continued on next page

Test set	$\|\overline{\Delta E}\|$	MD	MAD	NMAD	RMSD	Δ_{err}	max	min
CR20	19.31	-6.13	6.13	0.32	6.20	12.03	7.92	4.11
CRBH20	46.13	-1.72	1.72	0.04	1.84	3.59	3.05	0.54
TMBH17	12.76	0.96	2.46	0.19	3.56	10.04	9.99	0.05
LTMBH26	9.98	-0.76	1.56	0.16	2.45	8.73	8.70	0.03
BH76	18.61	-1.16	1.84	0.10	2.38	8.34	8.24	0.10
ISO34	14.57	0.97	0.97	0.07	1.59	6.92	6.91	0.01
ICONF	3.27	0.37	0.37	0.11	0.46	0.90	0.89	0.01
ACONF	1.83	0.22	0.22	0.12	0.25	0.47	0.40	0.07
TAUT15	3.05	0.68	0.68	0.22	0.85	1.75	1.75	0.00
Amino20x4	2.44	0.18	0.18	0.08	0.24	0.67	0.67	0.00
PCONF	1.62	0.73	0.73	0.45	0.86	1.73	1.67	0.06
MCONF	4.97	0.27	0.27	0.05	0.30	0.58	0.55	0.03
SCONF	4.60	0.32	0.32	0.07	0.41	0.81	0.80	0.01
PArel	4.63	0.09	0.62	0.13	0.86	1.95	1.94	0.01
BUT14DIOL	2.80	0.42	0.42	0.15	0.43	0.77	0.68	0.09
EIE22	5.44	0.75	0.75	0.14	0.84	1.72	1.67	0.05
Styrene45	62.64	2.87	3.51	0.06	4.33	11.69	11.69	0.00
ISOMERIZATION20	31.84	-0.70	1.51	0.05	1.98	4.03	4.02	0.01
DIE60	4.71	0.70	0.70	0.15	0.78	1.54	1.43	0.11
IDISP	14.22	2.68	2.68	0.19	3.11	5.68	5.24	0.44
C20C24	30.77	-0.39	29.68	0.96	32.08	65.84	49.60	16.24
S66	5.47	-1.70	1.70	0.31	1.94	4.96	4.66	0.30
S10x8	6.59	0.84	0.84	0.13	1.16	4.76	4.72	0.04
X40	3.76	1.07	1.07	0.28	1.28	3.44	3.42	0.02
HEAVY28	1.24	-0.43	0.47	0.38	0.55	1.61	1.46	0.15
CHB6	26.79	1.16	1.16	0.04	1.49	2.72	2.69	0.03
AHB21	22.49	-0.37	0.84	0.04	1.06	2.56	2.47	0.09
IL16	109.04	2.02	2.02	0.02	2.17	4.07	3.37	0.70
PNICO23	4.27	-0.65	0.65	0.15	0.79	1.76	1.75	0.01
CT20	0.98	0.38	0.38	0.38	0.39	0.79	0.60	0.19
CARBHB12	6.04	0.66	0.66	0.11	0.80	1.50	1.49	0.01
ADIM6	3.36	-0.69	0.69	0.21	0.74	1.35	1.05	0.30
3B-69-TRIM	12.30	3.02	3.02	0.25	3.36	7.75	7.35	0.40

Table S57: Statistical analysis for B2K-PLYP for all testset in our databsase. The numbers given (all in kcal/mol) are average reaction energy ($|\overline{\Delta E}|$), mean deviation (MD), mean absolute deviation (MAD), MAD normalized with respect to $|\overline{\Delta E}|$ (NMAD), root-mean-square deviation (RMSD), deviation span (Δ_{err}), maximum (max) and minimum deviation (min).

Test set	$\|\overline{\Delta E}\|$	MD	MAD	NMAD	RMSD	Δ_{err}	max	min
FH51	31.01	1.39	1.39	0.04	1.81	4.23	4.17	0.06
YBDE18	49.28	-1.50	2.19	0.04	2.44	5.19	4.54	0.65

Continued on next page

Test set	$\lvert\overline{\Delta E}\rvert$	MD	MAD	NMAD	RMSD	Δ_{err}	max	min
AL2X6	35.88	-1.91	2.23	0.06	2.48	4.53	3.74	0.79
DARC	32.47	0.68	0.74	0.02	0.98	1.91	1.85	0.06
NBPRC	27.71	0.72	2.21	0.08	2.50	4.17	4.10	0.07
HEAVYSB9	58.02	-2.37	2.37	0.04	2.96	6.36	5.87	0.49
BSR36	16.20	-0.86	0.89	0.06	0.94	1.77	1.40	0.37
RSE43	7.60	0.59	0.61	0.08	1.15	4.90	4.89	0.01
W4-11	306.91	-6.68	6.94	0.02	8.05	22.42	22.38	0.04
G21EA	33.62	-6.51	6.53	0.19	6.98	12.33	12.13	0.20
G21IP	257.61	-1.41	2.37	0.01	2.95	9.01	8.83	0.18
DIPCS10	654.26	-3.73	3.90	0.01	4.80	9.95	9.86	0.09
PA26	189.05	0.49	1.43	0.01	2.03	5.53	5.49	0.04
SIE4x4	33.72	4.06	4.13	0.12	4.97	11.08	10.52	0.56
ALKBDE10	100.69	-1.10	3.43	0.03	4.67	10.71	10.70	0.01
RC21	35.70	-1.13	1.55	0.04	1.91	3.85	3.67	0.18
ALK8	62.60	-0.79	2.25	0.04	2.91	6.03	5.46	0.57
DC13	54.98	1.28	5.41	0.10	8.51	23.11	23.02	0.09
G2RC	51.26	-1.90	2.42	0.05	3.10	9.56	9.49	0.07
BH76RC	21.39	-0.49	1.12	0.05	1.64	4.86	4.84	0.02
MOR23	35.57	-1.71	4.25	0.12	5.59	14.02	13.92	0.10
WCPT18	34.99	1.89	1.89	0.05	2.23	4.59	4.07	0.52
BHROT27	6.37	0.27	0.27	0.04	0.37	1.05	1.05	0.00
BHPERI	20.87	0.70	0.70	0.03	0.84	1.97	1.96	0.01
BHDIV10	45.33	1.55	1.55	0.03	1.96	4.24	4.15	0.09
INV24	32.85	1.89	1.89	0.06	2.31	4.52	4.46	0.06
CR20	19.31	-4.99	4.99	0.26	5.09	9.91	6.74	3.17
CRBH20	46.13	0.17	0.54	0.01	0.68	1.35	1.34	0.01
TMBH17	12.76	2.21	3.58	0.28	5.68	18.75	18.66	0.09
LTMBH26	9.98	0.01	1.01	0.10	1.75	7.39	7.28	0.11
BH76	18.61	-0.29	1.51	0.08	2.26	9.22	9.17	0.05
ISO34	14.57	0.76	0.76	0.05	1.38	6.24	6.23	0.01
ICONF	3.27	0.37	0.37	0.11	0.45	0.83	0.79	0.04
ACONF	1.83	0.13	0.13	0.07	0.16	0.30	0.27	0.03
TAUT15	3.05	0.64	0.64	0.21	0.84	1.74	1.72	0.02
Amino20x4	2.44	0.15	0.15	0.06	0.20	0.52	0.52	0.00
PCONF	1.62	0.42	0.42	0.26	0.49	0.96	0.95	0.01
MCONF	4.97	0.23	0.23	0.05	0.28	0.68	0.67	0.01
SCONF	4.60	3.28	3.28	0.71	3.60	6.92	6.82	0.10
PArel	4.63	0.16	0.59	0.13	0.78	1.70	1.70	0.00
BUT14DIOL	2.80	0.50	0.50	0.18	0.51	0.81	0.72	0.09
EIE22	5.44	0.63	0.63	0.12	0.70	1.51	1.47	0.04
Styrene45	62.64	2.87	3.19	0.05	3.91	10.19	10.19	0.00
ISOMERIZATION20	31.84	-0.92	1.56	0.05	2.06	4.82	4.76	0.06
DIE60	4.71	0.61	0.61	0.13	0.70	1.39	1.29	0.10
IDISP	14.22	2.75	2.75	0.19	4.92	11.74	11.74	0.00

Continued on next page

Test set	$\|\overline{\Delta E}\|$	MD	MAD	NMAD	RMSD	Δ_{err}	max	min
C20C24	30.77	-0.70	25.16	0.82	27.44	55.18	42.63	12.55
S66	5.47	-1.44	1.44	0.26	1.65	4.21	4.00	0.21
S10x8	6.59	0.69	0.69	0.10	0.97	3.98	3.96	0.02
X40	3.76	0.91	0.91	0.24	1.09	2.75	2.75	0.00
HEAVY28	1.24	-0.31	0.38	0.31	0.47	1.41	1.41	0.00
CHB6	26.79	1.07	1.07	0.04	1.37	2.47	2.43	0.04
AHB21	22.49	-0.47	0.88	0.04	1.14	2.88	2.81	0.07
IL16	109.04	1.87	1.87	0.02	2.02	3.86	3.26	0.60
PNICO23	4.27	-0.46	0.49	0.11	0.63	1.53	1.51	0.02
CT20	0.98	0.33	0.33	0.33	0.34	0.74	0.57	0.17
CARBHB12	6.04	0.80	0.80	0.13	0.93	1.66	1.60	0.06
ADIM6	3.36	-0.11	0.11	0.03	0.12	0.22	0.17	0.05
3B-69-TRIM	12.30	2.54	2.54	0.21	2.85	6.68	6.43	0.25

Table S58: Statistical analysis for B2T-PLYP for all testset in our databsase. The numbers given (all in kcal/mol) are average reaction energy ($|\overline{\Delta E}|$), mean deviation (MD), mean absolute deviation (MAD), MAD normalized with respect to $|\overline{\Delta E}|$ (NMAD), root-mean-square deviation (RMSD), deviation span (Δ_{err}), maximum (max) and minimum deviation (min).

Test set	$\|\overline{\Delta E}\|$	MD	MAD	NMAD	RMSD	Δ_{err}	max	min
FH51	31.01	1.40	1.40	0.05	1.92	6.10	6.07	0.03
YBDE18	49.28	-3.24	3.77	0.08	3.91	8.40	6.08	2.32
AL2X6	35.88	-3.60	3.60	0.10	4.00	6.95	5.72	1.23
DARC	32.47	4.67	4.67	0.14	4.75	8.63	5.85	2.78
NBPRC	27.71	1.70	2.81	0.10	3.67	8.04	7.94	0.10
HEAVYSB9	58.02	-3.75	3.75	0.06	4.09	8.67	6.79	1.88
BSR36	16.20	-2.99	2.99	0.18	3.24	7.08	5.84	1.24
RSE43	7.60	-0.09	0.36	0.05	0.54	2.21	2.21	0.00
W4-11	306.91	-5.35	5.45	0.02	6.48	20.01	19.86	0.15
G21EA	33.62	-6.02	6.13	0.18	6.50	12.57	11.14	1.43
G21IP	257.61	-1.22	2.46	0.01	2.94	8.11	8.06	0.05
DIPCS10	654.26	-3.35	3.60	0.01	4.49	10.07	9.56	0.51
PA26	189.05	0.54	1.46	0.01	2.09	6.08	6.07	0.01
SIE4x4	33.72	7.21	7.22	0.21	8.54	17.39	17.36	0.03
ALKBDE10	100.69	-0.74	3.58	0.04	4.54	10.55	10.12	0.43
RC21	35.70	-1.12	1.36	0.04	1.64	3.19	3.13	0.06
ALK8	62.60	-2.04	2.46	0.04	3.86	8.40	8.37	0.03
DC13	54.98	2.43	7.69	0.14	11.26	26.74	26.59	0.15
G2RC	51.26	-0.73	1.84	0.04	2.35	7.07	6.81	0.26
BH76RC	21.39	-0.44	1.01	0.05	1.45	4.58	4.53	0.05
MOR23	35.57	0.65	3.67	0.10	5.01	12.21	12.16	0.05
WCPT18	34.99	1.68	1.68	0.05	2.08	4.05	4.01	0.04

Continued on next page

Test set	$\lvert\overline{\Delta E}\rvert$	MD	MAD	NMAD	RMSD	Δ_{err}	max	min
BHROT27	6.37	0.26	0.26	0.04	0.36	1.07	1.07	0.00
BHPERI	20.87	1.00	1.00	0.05	1.23	3.01	2.99	0.02
BHDIV10	45.33	1.79	1.79	0.04	2.23	4.64	4.43	0.21
INV24	32.85	1.62	1.62	0.05	1.95	4.51	4.27	0.24
CR20	19.31	-6.92	6.92	0.36	6.99	13.53	8.80	4.73
CRBH20	46.13	-2.96	2.96	0.06	3.04	6.11	4.34	1.77
TMBH17	12.76	1.03	2.60	0.20	3.82	10.96	10.89	0.07
LTMBH26	9.98	-0.95	1.79	0.18	2.82	9.93	9.92	0.01
BH76	18.61	-1.66	2.11	0.11	2.62	7.78	7.66	0.12
ISO34	14.57	1.13	1.13	0.08	1.78	7.47	7.46	0.01
ICONF	3.27	0.39	0.39	0.12	0.48	1.02	0.98	0.04
ACONF	1.83	0.31	0.31	0.17	0.34	0.66	0.56	0.10
TAUT15	3.05	0.72	0.72	0.24	0.87	1.78	1.77	0.01
Amino20x4	2.44	0.22	0.22	0.09	0.29	0.83	0.83	0.00
PCONF	1.62	1.08	1.08	0.67	1.23	2.37	2.28	0.09
MCONF	4.97	0.52	0.52	0.10	0.55	1.12	0.96	0.16
SCONF	4.60	3.28	3.28	0.71	3.60	6.92	6.82	0.10
PArel	4.63	0.06	0.63	0.14	0.92	2.32	2.32	0.00
BUT14DIOL	2.80	0.36	0.36	0.13	0.37	0.74	0.65	0.09
EIE22	5.44	0.84	0.84	0.15	0.93	1.86	1.80	0.06
Styrene45	62.64	2.95	3.82	0.06	4.74	12.77	12.77	0.00
ISOMERIZATION20	31.84	-0.55	1.46	0.05	1.98	4.40	4.39	0.01
DIE60	4.71	0.76	0.76	0.16	0.83	1.67	1.57	0.10
IDISP	14.22	2.85	2.85	0.20	3.43	5.35	5.26	0.09
C20C24	30.77	-0.07	33.07	1.07	35.50	73.95	54.61	19.34
S66	5.47	-1.89	1.89	0.35	2.16	5.55	5.19	0.36
S10x8	6.59	0.95	0.95	0.14	1.31	5.40	5.35	0.05
X40	3.76	1.19	1.19	0.32	1.44	4.04	3.97	0.07
HEAVY28	1.24	-0.52	0.56	0.45	0.63	1.68	1.53	0.15
CHB6	26.79	1.18	1.19	0.04	1.54	2.88	2.85	0.03
AHB21	22.49	-0.31	0.84	0.04	1.05	2.43	2.39	0.04
IL16	109.04	2.15	2.15	0.02	2.29	4.40	3.59	0.81
PNICO23	4.27	-0.81	0.81	0.19	0.93	2.11	1.98	0.13
CT20	0.98	0.41	0.41	0.42	0.43	0.83	0.64	0.19
CARBHB12	6.04	0.54	0.55	0.09	0.71	1.45	1.40	0.05
ADIM6	3.36	-1.18	1.18	0.35	1.28	2.36	1.88	0.48
3B-69-TRIM	12.30	3.38	3.38	0.27	3.76	8.59	8.12	0.47

Table S59: Statistical analysis for B2π-PLYP for all testset in our databsase. The numbers given (all in kcal/mol) are average reaction energy ($|\overline{\Delta E}|$), mean deviation (MD), mean absolute deviation (MAD), MAD normalized with respect to $|\overline{\Delta E}|$ (NMAD), root-mean-square deviation (RMSD), deviation span (Δ_{err}), maximum (max) and minimum deviation (min).

Test set	$\lvert\overline{\Delta E}\rvert$	MD	MAD	NMAD	RMSD	Δ_{err}	max	min
FH51	31.01	1.33	1.33	0.04	1.84	5.24	5.23	0.01
YBDE18	49.28	-2.53	2.91	0.06	3.12	7.06	5.57	1.49
AL2X6	35.88	-3.04	3.04	0.08	3.56	5.61	5.28	0.33
DARC	32.47	3.23	3.23	0.10	3.38	5.24	4.47	0.77
NBPRC	27.71	1.35	2.73	0.10	3.30	6.48	6.36	0.12
HEAVYSB9	58.02	-3.34	3.34	0.06	3.68	7.19	6.09	1.10
BSR36	16.20	-3.52	3.52	0.22	3.89	9.17	7.83	1.34
RSE43	7.60	-0.20	0.37	0.05	0.51	1.52	1.52	0.00
W4-11	306.91	-3.15	3.96	0.01	5.60	20.09	20.07	0.02
G21EA	33.62	-5.06	5.26	0.16	5.70	12.46	10.11	2.35
G21IP	257.61	0.09	2.24	0.01	2.84	6.77	6.71	0.06
DIPCS10	654.26	-0.69	2.75	0.00	3.57	7.84	7.80	0.04
PA26	189.05	0.70	1.57	0.01	2.21	6.39	6.34	0.05
SIE4x4	33.72	7.04	7.04	0.21	8.43	17.79	17.76	0.03
ALKBDE10	100.69	-0.98	3.52	0.03	4.43	10.23	9.99	0.24
RC21	35.70	-0.09	0.85	0.02	1.00	2.14	2.09	0.05
ALK8	62.60	-1.52	2.56	0.04	3.91	9.03	8.58	0.45
DC13	54.98	1.29	7.54	0.14	10.32	24.02	23.48	0.54
G2RC	51.26	-1.77	2.51	0.05	3.07	8.41	8.07	0.34
BH76RC	21.39	-0.69	1.44	0.07	1.91	4.86	4.76	0.10
MOR23	35.57	0.76	3.30	0.09	4.31	9.55	9.39	0.16
WCPT18	34.99	1.77	1.77	0.05	2.08	3.94	3.71	0.23
BHROT27	6.37	0.33	0.33	0.05	0.46	1.21	1.20	0.01
BHPERI	20.87	1.08	1.08	0.05	1.29	2.93	2.79	0.14
BHDIV10	45.33	1.69	1.69	0.04	2.12	4.79	4.63	0.16
INV24	32.85	1.71	1.71	0.05	1.98	3.99	3.69	0.30
CR20	19.31	-5.66	5.66	0.29	5.77	11.19	7.78	3.41
CRBH20	46.13	-1.69	1.69	0.04	1.85	3.82	3.21	0.61
TMBH17	12.76	1.65	2.60	0.20	3.93	11.57	11.23	0.34
LTMBH26	9.98	-0.63	1.61	0.16	2.78	11.15	11.13	0.02
BH76	18.61	-1.61	2.12	0.11	2.58	7.50	7.45	0.05
ISO34	14.57	1.00	1.00	0.07	1.68	7.39	7.39	0.00
ICONF	3.27	0.40	0.40	0.12	0.50	1.01	0.96	0.05
ACONF	1.83	0.30	0.30	0.16	0.34	0.64	0.54	0.10
TAUT15	3.05	0.71	0.71	0.23	0.90	1.61	1.60	0.01
Amino20x4	2.44	0.24	0.24	0.10	0.31	0.92	0.92	0.00
PCONF	1.62	1.08	1.08	0.67	1.25	2.26	2.26	0.00
MCONF	4.97	0.54	0.54	0.11	0.58	1.22	1.02	0.20

Continued on next page

Test set	$\|\overline{\Delta E}\|$	MD	MAD	NMAD	RMSD	Δ_{err}	max	min
SCONF	4.60	3.28	3.28	0.71	3.60	6.92	6.82	0.10
PArel	4.63	0.17	0.58	0.13	0.88	2.63	2.63	0.00
BUT14DIOL	2.80	0.41	0.41	0.15	0.42	0.80	0.71	0.09
EIE22	5.44	0.83	0.83	0.15	0.92	1.84	1.80	0.04
Styrene45	62.64	3.30	3.79	0.06	4.62	11.80	11.80	0.00
ISOMERIZATION20	31.84	-0.69	1.44	0.05	1.96	4.09	4.08	0.01
DIE60	4.71	0.75	0.75	0.16	0.85	1.66	1.64	0.02
IDISP	14.22	3.14	3.14	0.22	3.82	6.94	6.54	0.40
C20C24	30.77	-0.23	30.57	0.99	32.80	69.22	50.56	18.66
S66	5.47	-1.66	1.66	0.30	1.99	4.97	4.85	0.12
S10x8	6.59	0.72	0.73	0.11	1.15	5.17	5.16	0.01
X40	3.76	1.05	1.05	0.28	1.32	4.03	3.84	0.19
HEAVY28	1.24	-0.48	0.52	0.42	0.59	1.68	1.51	0.17
CHB6	26.79	0.70	0.87	0.03	1.23	2.51	2.44	0.07
AHB21	22.49	-0.78	1.06	0.05	1.43	3.71	3.63	0.08
IL16	109.04	1.74	1.74	0.02	1.91	3.67	3.19	0.48
PNICO23	4.27	-0.66	0.66	0.16	0.81	1.95	1.95	0.00
CT20	0.98	0.32	0.32	0.33	0.34	0.61	0.54	0.07
CARBHB12	6.04	0.70	0.70	0.12	0.86	1.67	1.66	0.01
ADIM6	3.36	-1.18	1.18	0.35	1.28	2.35	1.90	0.45
3B-69-TRIM	12.30	2.90	2.90	0.24	3.37	7.94	7.92	0.02

Table S60: Statistical analysis for B2NC-PLYP for all testset in our databsase. The numbers given (all in kcal/mol) are average reaction energy ($|\overline{\Delta E}|$), mean deviation (MD), mean absolute deviation (MAD), MAD normalized with respect to $|\overline{\Delta E}|$ (NMAD), root-mean-square deviation (RMSD), deviation span (Δ_{err}), maximum (max) and minimum deviation (min).

Test set	$\|\overline{\Delta E}\|$	MD	MAD	NMAD	RMSD	Δ_{err}	max	min
FH51	31.01	1.32	1.32	0.04	1.75	4.57	4.54	0.03
YBDE18	49.28	0.08	2.00	0.04	3.18	9.18	9.09	0.09
AL2X6	35.88	-2.06	2.06	0.06	2.35	4.29	3.82	0.47
DARC	32.47	2.22	2.22	0.07	2.36	4.56	3.28	1.28
NBPRC	27.71	0.67	2.04	0.07	2.37	4.90	4.50	0.40
HEAVYSB9	58.02	-0.57	1.31	0.02	1.83	3.78	3.74	0.04
BSR36	16.20	0.88	0.92	0.06	1.53	5.49	5.41	0.08
RSE43	7.60	0.37	0.64	0.08	1.27	5.78	5.77	0.01
W4-11	306.91	1.63	4.08	0.01	5.23	14.08	13.98	0.10
G21EA	33.62	-5.61	5.61	0.17	5.89	10.42	9.91	0.51
G21IP	257.61	-1.38	2.46	0.01	3.02	8.66	8.53	0.13
DIPCS10	654.26	-4.04	4.04	0.01	4.53	7.49	7.03	0.46
PA26	189.05	-0.18	1.43	0.01	1.82	4.79	4.49	0.30
SIE4x4	33.72	6.71	6.75	0.20	7.83	13.84	13.50	0.34

Continued on next page

Test set	$\lvert\overline{\Delta E}\rvert$	MD	MAD	NMAD	RMSD	Δ_{err}	max	min
ALKBDE10	100.69	4.71	6.50	0.06	7.80	12.92	12.88	0.04
RC21	35.70	-1.88	2.24	0.06	2.87	6.45	6.30	0.15
ALK8	62.60	-0.91	1.66	0.03	2.11	3.81	3.76	0.05
DC13	54.98	2.76	6.29	0.11	8.66	21.76	21.55	0.21
G2RC	51.26	-0.14	2.21	0.04	3.01	8.19	8.14	0.05
BH76RC	21.39	-0.27	1.61	0.08	2.01	5.23	4.93	0.30
MOR23	35.57	-3.30	6.42	0.18	8.96	23.85	23.35	0.50
WCPT18	34.99	3.63	3.63	0.10	4.02	7.73	6.82	0.91
BHROT27	6.37	0.19	0.19	0.03	0.28	0.97	0.96	0.01
BHPERI	20.87	3.04	3.04	0.15	3.23	7.20	5.89	1.31
BHDIV10	45.33	2.43	2.43	0.05	2.86	5.69	5.31	0.38
INV24	32.85	2.03	2.03	0.06	2.71	7.19	7.15	0.04
CR20	19.31	-5.96	5.96	0.31	6.02	11.54	7.23	4.31
CRBH20	46.13	-1.99	1.99	0.04	2.08	4.13	3.43	0.70
TMBH17	12.76	-1.16	3.22	0.25	3.85	7.23	6.98	0.25
LTMBH26	9.98	-1.41	1.93	0.19	2.47	6.92	6.84	0.08
BH76	18.61	-1.89	2.67	0.14	3.33	8.08	8.07	0.01
ISO34	14.57	0.96	0.96	0.07	1.50	6.00	5.98	0.02
ICONF	3.27	0.30	0.30	0.09	0.36	0.77	0.75	0.02
ACONF	1.83	0.04	0.04	0.02	0.04	0.08	0.08	0.00
TAUT15	3.05	0.79	0.79	0.26	1.01	1.86	1.86	0.00
Amino20x4	2.44	0.19	0.19	0.08	0.25	0.65	0.65	0.00
PCONF	1.62	0.51	0.51	0.31	0.58	1.18	1.10	0.08
MCONF	4.97	0.49	0.49	0.10	0.54	1.11	0.97	0.14
SCONF	4.60	3.28	3.28	0.71	3.60	6.92	6.82	0.10
PArel	4.63	-0.08	0.94	0.20	1.36	3.72	3.70	0.02
BUT14DIOL	2.80	0.60	0.60	0.21	0.61	0.81	0.74	0.07
EIE22	5.44	0.78	0.79	0.14	0.88	1.73	1.69	0.04
Styrene45	62.64	1.96	2.96	0.05	3.83	11.05	11.05	0.00
ISOMERIZATION20	31.84	-0.47	2.27	0.07	2.99	7.36	7.33	0.03
DIE60	4.71	0.73	0.73	0.15	0.77	1.78	1.44	0.34
IDISP	14.22	3.23	3.23	0.23	5.95	14.55	14.31	0.24
C20C24	30.77	-1.61	26.50	0.86	29.79	56.63	47.01	9.62
S66	5.47	-1.54	1.54	0.28	1.70	4.35	4.02	0.33
S10x8	6.59	0.85	0.85	0.13	1.09	3.49	3.47	0.02
X40	3.76	0.97	0.97	0.26	1.09	2.71	2.50	0.21
HEAVY28	1.24	-0.23	0.31	0.25	0.40	1.23	1.21	0.02
CHB6	26.79	1.69	1.69	0.06	1.89	3.57	2.83	0.74
AHB21	22.49	-0.09	0.61	0.03	0.74	1.57	1.53	0.04
IL16	109.04	1.79	1.79	0.02	1.98	3.60	3.21	0.39
PNICO23	4.27	-0.40	0.43	0.10	0.52	1.36	1.31	0.05
CT20	0.98	0.39	0.39	0.39	0.40	0.77	0.59	0.18
CARBHB12	6.04	0.88	0.88	0.15	1.02	1.75	1.69	0.06
ADIM6	3.36	0.27	0.27	0.08	0.34	0.65	0.63	0.02

Continued on next page

Test set	$\|\overline{\Delta E}\|$	MD	MAD	NMAD	RMSD	Δ_{err}	max	min
3B-69-TRIM	12.30	2.85	2.85	0.23	3.07	6.87	6.20	0.67

Table S61: Statistical analysis for mPW2-PLYP for all testset in our databsase. The numbers given (all in kcal/mol) are average reaction energy ($|\overline{\Delta E}|$), mean deviation (MD), mean absolute deviation (MAD), MAD normalized with respect to $|\overline{\Delta E}|$ (NMAD), root-mean-square deviation (RMSD), deviation span (Δ_{err}), maximum (max) and minimum deviation (min).

Test set	$\|\overline{\Delta E}\|$	MD	MAD	NMAD	RMSD	Δ_{err}	max	min
FH51	31.01	1.52	1.52	0.05	2.12	7.42	7.40	0.02
YBDE18	49.28	-3.89	4.27	0.09	4.58	8.76	7.42	1.34
AL2X6	35.88	-3.78	3.78	0.11	4.25	7.56	6.31	1.25
DARC	32.47	6.00	6.00	0.18	6.10	10.62	7.22	3.40
NBPRC	27.71	1.86	3.19	0.11	4.13	8.81	8.80	0.01
HEAVYSB9	58.02	-4.29	4.29	0.07	4.61	9.72	7.41	2.31
BSR36	16.20	-4.23	4.23	0.26	4.82	12.03	10.57	1.46
RSE43	7.60	-0.32	0.43	0.06	0.56	1.50	1.48	0.02
W4-11	306.91	-5.41	5.53	0.02	6.70	21.21	21.04	0.17
G21EA	33.62	-5.47	5.69	0.17	6.09	13.29	10.56	2.73
G21IP	257.61	-0.55	2.43	0.01	2.97	6.99	6.91	0.08
DIPCS10	654.26	-1.88	3.14	0.00	4.07	9.46	8.90	0.56
PA26	189.05	0.49	1.52	0.01	2.18	6.32	6.32	0.00
SIE4x4	33.72	8.44	8.44	0.25	9.93	20.18	20.10	0.08
ALKBDE10	100.69	-1.00	3.70	0.04	4.53	10.84	10.23	0.61
RC21	35.70	-0.76	1.21	0.03	1.51	4.28	4.13	0.15
ALK8	62.60	-1.97	2.60	0.04	4.27	9.78	9.61	0.17
DC13	54.98	2.37	9.08	0.17	12.93	27.63	27.35	0.28
G2RC	51.26	-0.70	1.96	0.04	2.45	6.38	6.26	0.12
BH76RC	21.39	-0.45	1.24	0.06	1.63	4.40	4.39	0.01
MOR23	35.57	1.44	3.77	0.11	4.87	11.15	10.93	0.22
WCPT18	34.99	1.70	1.70	0.05	2.06	4.17	3.89	0.28
BHROT27	6.37	0.29	0.29	0.05	0.39	1.14	1.12	0.02
BHPERI	20.87	1.30	1.30	0.06	1.60	3.40	3.35	0.05
BHDIV10	45.33	1.91	1.91	0.04	2.40	5.24	4.84	0.40
INV24	32.85	1.51	1.51	0.05	1.82	3.86	3.76	0.10
CR20	19.31	-7.21	7.21	0.37	7.29	14.10	9.27	4.83
CRBH20	46.13	-4.25	4.25	0.09	4.31	8.71	5.64	3.07
TMBH17	12.76	1.39	2.90	0.23	4.31	12.94	12.56	0.38
LTMBH26	9.98	-0.45	1.64	0.16	2.86	11.46	11.41	0.05
BH76	18.61	-2.30	2.59	0.14	3.12	7.14	7.02	0.12
ISO34	14.57	1.37	1.37	0.09	2.07	8.41	8.41	0.00
ICONF	3.27	0.40	0.40	0.12	0.49	1.04	1.03	0.01
ACONF	1.83	0.34	0.34	0.18	0.38	0.77	0.65	0.12

Continued on next page

Test set	$\|\overline{\Delta E}\|$	MD	MAD	NMAD	RMSD	Δ_{err}	max	min
TAUT15	3.05	0.73	0.73	0.24	0.88	1.74	1.73	0.01
Amino20x4	2.44	0.26	0.26	0.10	0.33	0.92	0.92	0.00
PCONF	1.62	1.19	1.19	0.73	1.35	2.51	2.43	0.08
MCONF	4.97	0.62	0.62	0.13	0.67	1.28	1.14	0.14
SCONF	4.60	3.28	3.28	0.71	3.60	6.92	6.82	0.10
PArel	4.63	0.03	0.68	0.15	0.97	2.84	2.81	0.03
BUT14DIOL	2.80	0.35	0.35	0.13	0.37	0.77	0.67	0.10
EIE22	5.44	0.90	0.90	0.16	0.98	1.93	1.86	0.07
Styrene45	62.64	3.50	4.48	0.07	5.61	15.00	15.00	0.00
ISOMERIZATION20	31.84	-0.52	1.33	0.04	1.85	3.97	3.90	0.07
DIE60	4.71	0.77	0.77	0.16	0.85	1.72	1.60	0.12
IDISP	14.22	4.43	4.43	0.31	4.88	8.20	7.25	0.95
C20C24	30.77	1.07	37.51	1.22	39.75	83.85	59.72	24.13
S66	5.47	-1.52	1.52	0.28	1.85	4.78	4.77	0.01
S10x8	6.59	0.65	0.66	0.10	1.11	5.33	5.32	0.01
X40	3.76	0.91	0.91	0.24	1.21	3.75	3.73	0.02
HEAVY28	1.24	-0.33	0.38	0.31	0.47	1.27	1.26	0.01
CHB6	26.79	0.58	0.86	0.03	1.21	2.49	2.44	0.05
AHB21	22.49	-0.70	0.93	0.04	1.24	2.94	2.92	0.02
IL16	109.04	1.68	1.68	0.02	1.84	3.52	3.07	0.45
PNICO23	4.27	-0.58	0.59	0.14	0.75	1.79	1.77	0.02
CT20	0.98	0.11	0.14	0.14	0.16	0.32	0.29	0.03
CARBHB12	6.04	0.75	0.75	0.12	0.88	1.77	1.64	0.13
ADIM6	3.36	-0.92	0.92	0.27	1.05	1.88	1.67	0.21
3B-69-TRIM	12.30	2.54	2.55	0.21	3.01	7.42	7.40	0.02

Table S62: Statistical analysis for mPW2NC-PLYP for all testset in our databsase. The numbers given (all in kcal/mol) are average reaction energy ($|\overline{\Delta E}|$), mean deviation (MD), mean absolute deviation (MAD), MAD normalized with respect to $|\overline{\Delta E}|$ (NMAD), root-mean-square deviation (RMSD), deviation span (Δ_{err}), maximum (max) and minimum deviation (min).

Test set	$\|\overline{\Delta E}\|$	MD	MAD	NMAD	RMSD	Δ_{err}	max	min
FH51	31.01	1.33	1.33	0.04	1.76	4.59	4.55	0.04
YBDE18	49.28	-2.45	3.41	0.07	3.50	7.35	5.41	1.94
AL2X6	35.88	-2.68	2.68	0.07	3.06	4.94	4.51	0.43
DARC	32.47	3.51	3.51	0.11	3.56	7.00	4.42	2.58
NBPRC	27.71	1.22	2.55	0.09	3.16	6.77	6.51	0.26
HEAVYSB9	58.02	-2.89	2.89	0.05	3.42	7.59	6.40	1.19
BSR36	16.20	-0.99	1.00	0.06	1.06	1.86	1.78	0.08
RSE43	7.60	0.48	0.55	0.07	1.07	4.79	4.79	0.00
W4-11	306.91	-7.46	7.68	0.03	8.95	23.35	23.05	0.30
G21EA	33.62	-6.89	6.89	0.21	7.28	12.29	12.29	0.00

Continued on next page

Test set	$\lvert\overline{\Delta E}\rvert$	MD	MAD	NMAD	RMSD	Δ_{err}	max	min
G21IP	257.61	-2.21	2.86	0.01	3.43	9.37	9.29	0.08
DIPCS10	654.26	-5.34	5.34	0.01	5.92	11.81	10.60	1.21
PA26	189.05	0.16	1.37	0.01	1.93	5.25	5.23	0.02
SIE4x4	33.72	5.66	5.72	0.17	6.72	13.21	12.78	0.43
ALKBDE10	100.69	-0.02	3.81	0.04	4.97	10.21	10.15	0.06
RC21	35.70	-2.05	2.22	0.06	2.70	5.64	5.61	0.03
ALK8	62.60	-1.32	2.13	0.03	2.90	6.01	5.84	0.17
DC13	54.98	2.70	7.15	0.13	10.61	27.01	26.58	0.43
G2RC	51.26	-0.47	1.77	0.03	2.43	7.52	7.47	0.05
BH76RC	21.39	-0.21	1.07	0.05	1.50	4.48	4.46	0.02
MOR23	35.57	-1.46	4.83	0.14	6.43	17.35	17.11	0.24
WCPT18	34.99	2.04	2.04	0.06	2.51	4.77	4.74	0.03
BHROT27	6.37	0.19	0.19	0.03	0.28	0.93	0.93	0.00
BHPERI	20.87	1.09	1.09	0.05	1.38	4.29	4.23	0.06
BHDIV10	45.33	1.80	1.80	0.04	2.24	4.25	4.18	0.07
INV24	32.85	1.82	1.82	0.06	2.27	5.18	5.16	0.02
CR20	19.31	-6.72	6.72	0.35	6.78	13.11	8.26	4.85
CRBH20	46.13	-2.52	2.52	0.05	2.60	5.10	3.87	1.23
TMBH17	12.76	0.36	2.74	0.21	3.63	9.84	9.63	0.21
LTMBH26	9.98	-1.01	1.71	0.17	2.42	6.70	6.64	0.06
BH76	18.61	-1.13	1.94	0.10	2.61	8.54	8.51	0.03
ISO34	14.57	1.10	1.10	0.08	1.68	6.99	6.98	0.01
ICONF	3.27	0.34	0.34	0.10	0.41	0.88	0.83	0.05
ACONF	1.83	0.15	0.15	0.08	0.17	0.31	0.27	0.04
TAUT15	3.05	0.66	0.66	0.22	0.83	1.89	1.89	0.00
Amino20x4	2.44	0.15	0.15	0.06	0.20	0.57	0.57	0.00
PCONF	1.62	0.45	0.45	0.28	0.53	1.06	1.04	0.02
MCONF	4.97	0.22	0.22	0.04	0.27	0.65	0.64	0.01
SCONF	4.60	3.28	3.28	0.71	3.60	6.92	6.82	0.10
PArel	4.63	-0.03	0.66	0.14	0.95	2.53	2.52	0.01
BUT14DIOL	2.80	0.45	0.45	0.16	0.46	0.76	0.67	0.09
EIE22	5.44	0.71	0.71	0.13	0.78	1.61	1.55	0.06
Styrene45	62.64	2.76	3.65	0.06	4.61	13.04	13.04	0.00
ISOMERIZATION20	31.84	-0.52	1.76	0.06	2.34	6.43	6.40	0.03
DIE60	4.71	0.65	0.65	0.14	0.71	1.37	1.21	0.16
IDISP	14.22	2.16	2.16	0.15	3.60	8.41	8.40	0.01
C20C24	30.77	0.20	31.41	1.02	33.88	67.99	51.53	16.46
S66	5.47	-1.42	1.42	0.26	1.62	4.30	4.07	0.23
S10x8	6.59	0.73	0.74	0.11	1.03	4.09	4.08	0.01
X40	3.76	0.87	0.87	0.23	1.04	2.74	2.69	0.05
HEAVY28	1.24	-0.20	0.29	0.23	0.38	1.19	1.19	0.00
CHB6	26.79	1.22	1.22	0.05	1.51	2.77	2.62	0.15
AHB21	22.49	-0.22	0.67	0.03	0.82	1.93	1.81	0.12
IL16	109.04	1.93	1.93	0.02	2.08	3.97	3.32	0.65

Continued on next page

Test set	$\lvert\overline{\Delta E}\rvert$	MD	MAD	NMAD	RMSD	Δ_{err}	max	min
PNICO23	4.27	-0.44	0.48	0.11	0.62	1.49	1.49	0.00
CT20	0.98	0.21	0.21	0.22	0.23	0.49	0.42	0.07
CARBHB12	6.04	0.80	0.80	0.13	0.91	1.66	1.51	0.15
ADIM6	3.36	0.12	0.12	0.04	0.13	0.26	0.19	0.07
3B-69-TRIM	12.30	2.49	2.49	0.20	2.76	6.66	6.22	0.44

Table S63: Statistical analysis for mPW2K-PLYP for all testset in our databsase. The numbers given (all in kcal/mol) are average reaction energy ($\lvert\overline{\Delta E}\rvert$), mean deviation (MD), mean absolute deviation (MAD), MAD normalized with respect to $\lvert\overline{\Delta E}\rvert$ (NMAD), root-mean-square deviation (RMSD), deviation span (Δ_{err}), maximum (max) and minimum deviation (min).

Test set	$\lvert\overline{\Delta E}\rvert$	MD	MAD	NMAD	RMSD	Δ_{err}	max	min
FH51	31.01	1.41	1.41	0.05	1.83	4.15	4.11	0.04
YBDE18	49.28	-1.19	1.98	0.04	2.29	5.35	4.94	0.41
AL2X6	35.88	-1.52	2.02	0.06	2.19	4.59	3.34	1.25
DARC	32.47	0.49	0.63	0.02	0.85	1.73	1.73	0.00
NBPRC	27.71	0.51	2.26	0.08	2.49	4.05	3.79	0.26
HEAVYSB9	58.02	-2.07	2.07	0.04	2.75	5.95	5.73	0.22
BSR36	16.20	-0.82	0.85	0.05	0.94	2.07	1.77	0.30
RSE43	7.60	0.60	0.61	0.08	1.15	4.88	4.88	0.00
W4-11	306.91	-6.13	6.48	0.02	7.57	22.10	21.83	0.27
G21EA	33.62	-6.20	6.25	0.19	6.69	12.36	11.77	0.59
G21IP	257.61	-1.02	2.21	0.01	2.79	8.29	8.24	0.05
DIPCS10	654.26	-2.89	3.37	0.01	4.23	9.19	9.16	0.03
PA26	189.05	0.37	1.43	0.01	2.01	5.48	5.38	0.10
SIE4x4	33.72	4.17	4.24	0.13	5.04	11.11	10.48	0.63
ALKBDE10	100.69	-0.72	3.48	0.03	4.63	10.74	10.44	0.30
RC21	35.70	-0.94	1.46	0.04	1.79	3.73	3.67	0.06
ALK8	62.60	-0.36	2.27	0.04	2.90	5.45	5.10	0.35
DC13	54.98	1.05	5.51	0.10	8.54	22.26	22.19	0.07
G2RC	51.26	-2.03	2.60	0.05	3.28	9.92	9.77	0.15
BH76RC	21.39	-0.49	1.16	0.05	1.65	4.83	4.82	0.01
MOR23	35.57	-2.13	4.27	0.12	5.70	14.18	14.11	0.07
WCPT18	34.99	2.03	2.03	0.06	2.40	4.92	4.31	0.61
BHROT27	6.37	0.27	0.27	0.04	0.37	1.07	1.06	0.01
BHPERI	20.87	0.80	0.80	0.04	0.97	2.54	2.39	0.15
BHDIV10	45.33	1.57	1.57	0.03	2.00	4.51	4.28	0.23
INV24	32.85	1.88	1.88	0.06	2.29	4.38	4.32	0.06
CR20	19.31	-4.70	4.70	0.24	4.80	9.36	6.46	2.90
CRBH20	46.13	0.01	0.52	0.01	0.66	1.40	1.39	0.01
TMBH17	12.76	2.23	3.53	0.28	5.47	17.50	17.38	0.12
LTMBH26	9.98	-0.44	1.26	0.13	1.98	7.14	7.12	0.02

Continued on next page

Test set	$\|\overline{\Delta E}\|$	MD	MAD	NMAD	RMSD	Δ_{err}	max	min
BH76	18.61	-0.48	1.62	0.09	2.33	8.92	8.92	0.00
ISO34	14.57	0.78	0.78	0.05	1.41	6.42	6.41	0.01
ICONF	3.27	0.36	0.36	0.11	0.44	0.83	0.79	0.04
ACONF	1.83	0.09	0.09	0.05	0.11	0.21	0.20	0.01
TAUT15	3.05	0.64	0.64	0.21	0.83	1.71	1.70	0.01
Amino20x4	2.44	0.15	0.15	0.06	0.19	0.53	0.53	0.00
PCONF	1.62	0.36	0.36	0.22	0.42	0.81	0.80	0.01
MCONF	4.97	0.31	0.31	0.06	0.35	0.68	0.64	0.04
SCONF	4.60	3.28	3.28	0.71	3.60	6.92	6.82	0.10
PArel	4.63	0.14	0.58	0.13	0.77	1.65	1.65	0.00
BUT14DIOL	2.80	0.55	0.55	0.20	0.56	0.84	0.75	0.09
EIE22	5.44	0.62	0.62	0.11	0.69	1.47	1.43	0.04
Styrene45	62.64	3.08	3.37	0.05	4.14	10.92	10.92	0.00
ISOMERIZATION20	31.84	-0.95	1.56	0.05	2.03	4.72	4.66	0.06
DIE60	4.71	0.59	0.59	0.12	0.67	1.31	1.22	0.09
IDISP	14.22	2.86	2.86	0.20	5.06	12.33	12.11	0.22
C20C24	30.77	-0.27	25.71	0.84	27.88	55.96	42.74	13.22
S66	5.47	-1.07	1.07	0.20	1.29	3.40	3.37	0.03
S10x8	6.59	0.43	0.46	0.07	0.74	3.47	3.46	0.01
X40	3.76	0.66	0.66	0.17	0.83	2.21	2.20	0.01
HEAVY28	1.24	-0.11	0.26	0.21	0.36	1.18	1.17	0.01
CHB6	26.79	0.72	0.82	0.03	1.10	2.24	2.08	0.16
AHB21	22.49	-0.73	0.92	0.04	1.26	3.08	3.06	0.02
IL16	109.04	1.47	1.47	0.01	1.64	3.10	2.81	0.29
PNICO23	4.27	-0.20	0.34	0.08	0.47	1.30	1.26	0.04
CT20	0.98	0.11	0.12	0.12	0.14	0.32	0.31	0.01
CARBHB12	6.04	1.03	1.03	0.17	1.14	2.04	1.81	0.23
ADIM6	3.36	0.42	0.42	0.12	0.45	0.93	0.71	0.22
3B-69-TRIM	12.30	1.78	1.79	0.15	2.12	5.37	5.36	0.01

Table S64: Statistical analysis for PBE0-DH for all testset in our databsase. The numbers given (all in kcal/mol) are average reaction energy ($|\overline{\Delta E}|$), mean deviation (MD), mean absolute deviation (MAD), MAD normalized with respect to $|\overline{\Delta E}|$ (NMAD), root-mean-square deviation (RMSD), deviation span (Δ_{err}), maximum (max) and minimum deviation (min).

Test set	$\|\overline{\Delta E}\|$	MD	MAD	NMAD	RMSD	Δ_{err}	max	min
FH51	31.01	2.86	2.86	0.09	3.62	10.66	10.65	0.01
YBDE18	49.28	-1.55	1.98	0.04	2.49	5.78	5.66	0.12
AL2X6	35.88	-0.72	1.06	0.03	1.30	2.34	2.32	0.02
DARC	32.47	-4.94	4.94	0.15	5.31	12.00	9.34	2.66
NBPRC	27.71	0.10	2.56	0.09	2.92	5.68	5.34	0.34
HEAVYSB9	58.02	-3.25	3.25	0.06	3.40	6.96	5.22	1.74

Continued on next page

Test set	$\lvert\overline{\Delta E}\rvert$	MD	MAD	NMAD	RMSD	Δ_{err}	max	min
BSR36	16.20	-4.70	4.70	0.29	5.22	12.85	10.90	1.95
RSE43	7.60	-0.44	0.49	0.06	0.64	1.87	1.83	0.04
W4-11	306.91	-7.45	7.53	0.02	9.11	28.92	28.76	0.16
G21EA	33.62	-4.90	5.16	0.15	6.61	12.48	12.39	0.09
G21IP	257.61	0.49	3.06	0.01	3.79	8.27	8.19	0.08
DIPCS10	654.26	0.30	2.93	0.00	3.41	6.52	6.03	0.49
PA26	189.05	1.42	1.91	0.01	2.52	6.91	6.81	0.10
SIE4x4	33.72	6.76	6.76	0.20	8.12	18.65	18.00	0.65
ALKBDE10	100.69	-5.82	6.25	0.06	7.52	15.35	15.32	0.03
RC21	35.70	3.33	3.81	0.11	4.79	13.66	13.11	0.55
ALK8	62.60	2.62	2.62	0.04	3.32	6.28	5.82	0.46
DC13	54.98	-2.04	8.20	0.15	10.11	20.80	20.34	0.46
G2RC	51.26	-4.34	5.96	0.12	7.76	19.52	19.44	0.08
BH76RC	21.39	-0.53	2.06	0.10	2.61	6.46	6.37	0.09
MOR23	35.57	0.54	2.64	0.07	3.18	6.58	6.51	0.07
WCPT18	34.99	2.52	2.52	0.07	3.11	6.00	5.83	0.17
BHROT27	6.37	0.53	0.53	0.08	0.72	1.40	1.39	0.01
BHPERI	20.87	1.42	1.42	0.07	1.71	4.36	4.20	0.16
BHDIV10	45.33	2.28	2.28	0.05	2.57	5.45	4.64	0.81
INV24	32.85	1.69	1.69	0.05	2.30	6.78	6.55	0.23
CR20	19.31	0.84	1.09	0.06	1.29	2.37	2.34	0.03
CRBH20	46.13	4.15	4.15	0.09	4.24	8.13	5.59	2.54
TMBH17	12.76	1.99	3.70	0.29	4.27	7.78	7.32	0.46
LTMBH26	9.98	-0.05	1.75	0.18	3.00	13.25	13.03	0.22
BH76	18.61	-1.45	2.15	0.12	2.47	7.28	6.97	0.31
ISO34	14.57	1.46	1.46	0.10	1.98	4.19	4.16	0.03
ICONF	3.27	0.44	0.44	0.13	0.58	1.37	1.30	0.07
ACONF	1.83	0.40	0.40	0.22	0.45	0.92	0.77	0.15
TAUT15	3.05	0.86	0.86	0.28	1.17	2.43	2.41	0.02
Amino20x4	2.44	0.32	0.32	0.13	0.41	1.28	1.28	0.00
PCONF	1.62	1.83	1.83	1.13	2.07	3.36	3.35	0.01
MCONF	4.97	0.84	0.84	0.17	0.95	1.61	1.59	0.02
SCONF	4.60	3.28	3.28	0.71	3.60	6.92	6.82	0.10
PArel	4.63	0.74	1.17	0.25	1.50	3.72	3.68	0.04
BUT14DIOL	2.80	0.15	0.16	0.06	0.21	0.53	0.53	0.00
EIE22	5.44	0.91	0.93	0.17	1.07	2.13	2.09	0.04
Styrene45	62.64	0.54	2.95	0.05	3.66	8.60	8.60	0.00
ISOMERIZATION20	31.84	-0.59	1.74	0.05	2.19	4.64	4.61	0.03
DIE60	4.71	1.08	1.10	0.23	1.31	2.77	2.70	0.07
IDISP	14.22	4.40	4.40	0.31	5.12	9.41	8.30	1.11
C20C24	30.77	-10.55	10.55	0.34	12.80	27.70	23.41	4.29
S66	5.47	-1.75	1.75	0.32	2.12	5.36	5.33	0.03
S10x8	6.59	0.70	0.75	0.11	1.17	5.05	5.04	0.01
X40	3.76	1.09	1.09	0.29	1.38	4.51	4.43	0.08

Continued on next page

Test set	$\|\overline{\Delta E}\|$	MD	MAD	NMAD	RMSD	Δ_{err}	max	min
HEAVY28	1.24	-0.42	0.48	0.39	0.54	1.31	1.26	0.05
CHB6	26.79	0.55	0.87	0.03	1.04	1.91	1.90	0.01
AHB21	22.49	-1.48	1.61	0.07	2.28	5.69	5.68	0.01
IL16	109.04	1.15	1.28	0.01	1.49	3.19	3.07	0.12
PNICO23	4.27	-0.22	0.39	0.09	0.57	1.86	1.85	0.01
CT20	0.98	0.43	0.43	0.44	0.46	0.85	0.69	0.16
CARBHB12	6.04	0.95	0.95	0.16	1.19	2.57	2.47	0.10
ADIM6	3.36	-2.10	2.10	0.63	2.32	4.27	3.54	0.73
3B-69-TRIM	12.30	3.26	3.27	0.27	3.82	9.01	8.92	0.09

Table S65: Statistical analysis for PBE0-2 for all testset in our databsase. The numbers given (all in kcal/mol) are average reaction energy ($|\overline{\Delta E}|$), mean deviation (MD), mean absolute deviation (MAD), MAD normalized with respect to $|\overline{\Delta E}|$ (NMAD), root-mean-square deviation (RMSD), deviation span (Δ_{err}), maximum (max) and minimum deviation (min).

Test set	$\|\overline{\Delta E}\|$	MD	MAD	NMAD	RMSD	Δ_{err}	max	min
FH51	31.01	3.14	3.14	0.10	3.98	11.44	11.41	0.03
YBDE18	49.28	2.40	3.48	0.07	4.27	8.24	8.15	0.09
AL2X6	35.88	1.64	1.64	0.05	1.92	2.95	2.80	0.15
DARC	32.47	-8.90	8.90	0.27	8.97	17.53	10.33	7.20
NBPRC	27.71	-1.40	2.91	0.11	3.20	7.82	6.19	1.63
HEAVYSB9	58.02	0.65	1.40	0.02	1.73	3.10	3.08	0.02
BSR36	16.20	2.06	2.08	0.13	2.93	9.13	9.04	0.09
RSE43	7.60	1.13	1.14	0.15	1.99	7.99	7.88	0.11
W4-11	306.91	-5.08	5.89	0.02	6.97	17.56	17.49	0.07
G21EA	33.62	-5.99	6.00	0.18	6.78	12.99	12.87	0.12
G21IP	257.61	-0.66	2.31	0.01	3.02	9.82	9.75	0.07
DIPCS10	654.26	-2.34	3.46	0.01	4.22	8.85	8.12	0.73
PA26	189.05	0.68	1.56	0.01	2.01	4.79	4.78	0.01
SIE4x4	33.72	1.13	1.49	0.04	1.83	3.64	3.59	0.05
ALKBDE10	100.69	-1.05	4.03	0.04	4.77	9.89	9.12	0.77
RC21	35.70	0.77	2.78	0.08	3.76	11.01	10.43	0.58
ALK8	62.60	3.23	3.23	0.05	4.17	8.84	8.19	0.65
DC13	54.98	-1.74	7.83	0.14	8.91	17.21	16.79	0.42
G2RC	51.26	-4.25	5.19	0.10	6.32	16.24	15.50	0.74
BH76RC	21.39	-0.45	2.15	0.10	2.73	6.45	6.31	0.14
MOR23	35.57	-5.92	7.48	0.21	9.26	19.58	19.52	0.06
WCPT18	34.99	3.06	3.06	0.09	3.74	6.60	6.58	0.02
BHROT27	6.37	0.37	0.37	0.06	0.47	1.14	1.13	0.01
BHPERI	20.87	3.66	3.66	0.18	4.05	8.49	7.53	0.96
BHDIV10	45.33	1.71	1.71	0.04	1.96	2.77	2.69	0.08
INV24	32.85	1.93	1.93	0.06	2.45	5.14	5.07	0.07

Continued on next page

Test set	$\lvert\overline{\Delta E}\rvert$	MD	MAD	NMAD	RMSD	Δ_{err}	max	min
CR20	19.31	1.12	1.24	0.06	1.38	2.30	2.19	0.11
CRBH20	46.13	6.55	6.55	0.14	6.58	12.71	7.42	5.29
TMBH17	12.76	0.31	2.64	0.21	3.17	6.34	6.14	0.20
LTMBH26	9.98	0.13	1.19	0.12	1.62	4.96	4.86	0.10
BH76	18.61	0.64	1.94	0.10	2.91	11.24	11.22	0.02
ISO34	14.57	1.17	1.17	0.08	1.71	4.26	4.26	0.00
ICONF	3.27	0.43	0.43	0.13	0.52	1.09	0.99	0.10
ACONF	1.83	0.08	0.08	0.04	0.11	0.27	0.27	0.00
TAUT15	3.05	0.90	0.90	0.29	1.09	1.90	1.84	0.06
Amino20x4	2.44	0.24	0.24	0.10	0.31	0.77	0.76	0.01
PCONF	1.62	0.58	0.58	0.36	0.77	1.80	1.77	0.03
MCONF	4.97	0.82	0.82	0.16	0.90	1.57	1.47	0.10
SCONF	4.60	3.28	3.28	0.71	3.60	6.92	6.82	0.10
PArel	4.63	0.54	1.10	0.24	1.39	3.98	3.91	0.07
BUT14DIOL	2.80	0.61	0.61	0.22	0.63	0.79	0.77	0.02
EIE22	5.44	0.50	0.54	0.10	0.61	1.39	1.35	0.04
Styrene45	62.64	0.73	2.78	0.04	3.52	8.01	8.01	0.00
ISOMERIZATION20	31.84	-1.25	2.00	0.06	2.84	9.78	9.59	0.19
DIE60	4.71	0.68	0.68	0.14	0.83	1.76	1.75	0.01
IDISP	14.22	7.09	7.09	0.50	12.30	30.33	29.43	0.90
C20C24	30.77	-8.35	8.35	0.27	9.42	14.99	12.70	2.29
S66	5.47	-0.99	0.99	0.18	1.13	2.89	2.81	0.08
S10x8	6.59	0.41	0.42	0.06	0.56	1.90	1.89	0.01
X40	3.76	0.64	0.64	0.17	0.71	1.56	1.51	0.05
HEAVY28	1.24	-0.01	0.24	0.19	0.37	1.10	1.09	0.01
CHB6	26.79	1.10	1.10	0.04	1.22	2.25	1.82	0.43
AHB21	22.49	-1.01	1.15	0.05	1.64	3.87	3.87	0.00
IL16	109.04	1.04	1.12	0.01	1.32	2.35	2.28	0.07
PNICO23	4.27	0.26	0.33	0.08	0.42	1.04	1.00	0.04
CT20	0.98	0.35	0.35	0.36	0.36	0.82	0.58	0.24
CARBHB12	6.04	1.34	1.34	0.22	1.51	2.66	2.40	0.26
ADIM6	3.36	0.75	0.75	0.22	0.86	1.69	1.46	0.23
3B-69-TRIM	12.30	1.89	1.91	0.16	2.15	4.97	4.89	0.08

Table S66: Statistical analysis for LS1TPSS for all testset in our databsase. The numbers given (all in kcal/mol) are average reaction energy ($\lvert\overline{\Delta E}\rvert$), mean deviation (MD), mean absolute deviation (MAD), MAD normalized with respect to $\lvert\overline{\Delta E}\rvert$ (NMAD), root-mean-square deviation (RMSD), deviation span (Δ_{err}), maximum (max) and minimum deviation (min).

Test set	$\lvert\overline{\Delta E}\rvert$	MD	MAD	NMAD	RMSD	Δ_{err}	max	min
FH51	31.01	-2.31	2.72	0.09	3.50	8.57	8.54	0.03
YBDE18	49.28	2.53	4.03	0.08	4.87	10.51	10.17	0.34

Continued on next page

Test set	$\lvert\overline{\Delta E}\rvert$	MD	MAD	NMAD	RMSD	Δ_{err}	max	min
AL2X6	35.88	1.88	1.88	0.05	2.25	3.80	3.42	0.38
DARC	32.47	-7.89	7.89	0.24	8.01	15.64	9.83	5.81
NBPRC	27.71	-1.50	2.58	0.09	2.88	6.53	5.60	0.93
HEAVYSB9	58.02	1.34	2.11	0.04	2.45	4.76	4.27	0.49
BSR36	16.20	3.33	3.33	0.21	4.50	13.04	13.00	0.04
RSE43	7.60	1.84	1.85	0.24	3.27	13.08	13.06	0.02
W4-11	306.91	-4.95	6.06	0.02	7.42	20.78	20.62	0.16
G21EA	33.62	-6.52	6.77	0.20	7.35	16.15	13.62	2.53
G21IP	257.61	-1.23	2.57	0.01	3.37	9.62	9.62	0.00
DIPCS10	654.26	-3.65	4.09	0.01	5.28	9.72	9.49	0.23
PA26	189.05	0.54	1.82	0.01	2.30	5.36	5.21	0.15
SIE4x4	33.72	0.39	0.98	0.03	1.19	2.44	2.44	0.00
ALKBDE10	100.69	-0.26	3.76	0.04	4.65	9.80	9.34	0.46
RC21	35.70	-1.14	3.07	0.09	3.67	8.92	8.76	0.16
ALK8	62.60	2.91	2.91	0.05	3.97	8.80	8.32	0.48
DC13	54.98	-0.85	6.94	0.13	8.22	15.46	14.69	0.77
G2RC	51.26	-3.44	4.14	0.08	5.09	12.21	11.71	0.50
BH76RC	21.39	-0.52	2.02	0.09	2.51	5.98	5.83	0.15
MOR23	35.57	-7.14	9.18	0.26	11.15	22.67	22.26	0.41
WCPT18	34.99	2.82	2.82	0.08	3.45	6.21	6.13	0.08
BHROT27	6.37	0.30	0.32	0.05	0.40	1.04	1.01	0.03
BHPERI	20.87	-3.74	3.92	0.19	4.40	9.11	8.86	0.25
BHDIV10	45.33	-0.20	1.45	0.03	1.66	3.37	2.99	0.38
INV24	32.85	-0.20	2.24	0.07	2.81	5.33	5.32	0.01
CR20	19.31	-0.05	0.82	0.04	1.04	2.66	2.63	0.03
CRBH20	46.13	6.61	6.61	0.14	6.65	12.68	7.47	5.21
TMBH17	12.76	0.25	2.72	0.21	3.50	7.29	7.27	0.02
LTMBH26	9.98	0.28	1.30	0.13	1.67	3.18	3.17	0.01
BH76	18.61	1.12	2.15	0.12	3.48	14.58	14.57	0.01
ISO34	14.57	1.14	1.14	0.08	1.61	4.65	4.65	0.00
ICONF	3.27	0.35	0.41	0.12	0.47	0.99	0.92	0.07
ACONF	1.83	-0.15	0.15	0.08	0.18	0.44	0.42	0.02
TAUT15	3.05	0.94	0.94	0.31	1.13	2.19	2.11	0.08
Amino20x4	2.44	0.09	0.27	0.11	0.34	0.87	0.87	0.00
PCONF	1.62	0.90	0.90	0.56	1.16	2.49	2.45	0.04
MCONF	4.97	1.08	1.08	0.22	1.18	1.99	1.87	0.12
SCONF	4.60	0.99	0.99	0.22	1.09	1.91	1.86	0.05
PArel	4.63	0.39	1.01	0.22	1.39	4.23	4.21	0.02
BUT14DIOL	2.80	0.66	0.66	0.24	0.69	0.92	0.89	0.03
EIE22	5.44	0.43	0.46	0.08	0.52	1.17	1.16	0.01
Styrene45	62.64	1.00	2.48	0.04	3.15	7.97	7.97	0.00
ISOMERIZATION20	31.84	-1.44	2.14	0.07	3.10	10.74	10.64	0.10
DIE60	4.71	0.62	0.62	0.13	0.76	1.54	1.48	0.06
IDISP	14.22	9.12	9.12	0.64	14.63	35.78	34.44	1.34

Continued on next page

Test set	$\|\overline{\Delta E}\|$	MD	MAD	NMAD	RMSD	Δ_{err}	max	min
C20C24	30.77	-7.20	8.98	0.29	10.17	17.77	15.65	2.12
S66	5.47	-1.05	1.05	0.19	1.16	2.96	2.74	0.22
S10x8	6.59	0.52	0.52	0.08	0.64	1.60	1.60	0.00
X40	3.76	0.68	0.68	0.18	0.74	1.23	1.19	0.04
HEAVY28	1.24	0.01	0.24	0.19	0.37	1.09	1.09	0.00
CHB6	26.79	1.42	1.42	0.05	1.52	3.24	2.27	0.97
AHB21	22.49	-0.62	0.92	0.04	1.29	3.08	3.04	0.04
IL16	109.04	1.41	1.41	0.01	1.61	2.81	2.80	0.01
PNICO23	4.27	0.19	0.27	0.06	0.35	0.71	0.69	0.02
CT20	0.98	0.40	0.40	0.40	0.41	0.97	0.70	0.27
CARBHB12	6.04	1.25	1.25	0.21	1.41	2.36	2.14	0.22
ADIM6	3.36	1.19	1.19	0.35	1.34	2.60	2.24	0.36
3B-69-TRIM	12.30	2.03	2.04	0.17	2.22	4.95	4.68	0.27

Table S67: Statistical analysis for LS1DH for all testset in our databsase. The numbers given (all in kcal/mol) are average reaction energy ($|\overline{\Delta E}|$), mean deviation (MD), mean absolute deviation (MAD), MAD normalized with respect to $|\overline{\Delta E}|$ (NMAD), root-mean-square deviation (RMSD), deviation span (Δ_{err}), maximum (max) and minimum deviation (min).

Test set	$\|\overline{\Delta E}\|$	MD	MAD	NMAD	RMSD	Δ_{err}	max	min
FH51	31.01	2.83	2.83	0.09	3.59	9.47	9.46	0.01
YBDE18	49.28	2.08	2.30	0.05	2.85	6.69	6.58	0.11
AL2X6	35.88	0.67	1.07	0.03	1.38	3.12	2.77	0.35
DARC	32.47	-5.53	5.53	0.17	5.63	11.03	7.03	4.00
NBPRC	27.71	-0.98	2.45	0.09	2.64	4.02	3.58	0.44
HEAVYSB9	58.02	0.58	1.27	0.02	1.51	2.69	2.62	0.07
BSR36	16.20	0.44	0.70	0.04	1.03	4.10	4.02	0.08
RSE43	7.60	0.43	0.60	0.08	1.11	4.70	4.70	0.00
W4-11	306.91	3.01	5.83	0.02	7.66	21.70	21.70	0.00
G21EA	33.62	-1.84	2.41	0.07	3.15	7.95	7.95	0.00
G21IP	257.61	3.44	3.78	0.01	4.44	9.12	9.05	0.07
DIPCS10	654.26	4.86	5.15	0.01	6.06	10.52	9.93	0.59
PA26	189.05	0.38	1.44	0.01	1.96	4.79	4.74	0.05
SIE4x4	33.72	3.52	3.65	0.11	4.39	10.29	9.69	0.60
ALKBDE10	100.69	2.02	4.58	0.05	5.30	10.38	9.00	1.38
RC21	35.70	1.45	2.25	0.06	3.28	9.33	9.12	0.21
ALK8	62.60	1.89	1.89	0.03	2.77	7.51	7.03	0.48
DC13	54.98	-1.49	5.57	0.10	6.40	9.98	9.84	0.14
G2RC	51.26	-4.37	5.09	0.10	6.19	13.42	12.96	0.46
BH76RC	21.39	-1.17	1.71	0.08	2.29	6.35	6.19	0.16
MOR23	35.57	-4.26	5.39	0.15	6.92	15.76	15.71	0.05
WCPT18	34.99	2.88	2.88	0.08	3.59	6.13	6.06	0.07

Continued on next page

Test set	$\|\overline{\Delta E}\|$	MD	MAD	NMAD	RMSD	Δ_{err}	max	min
BHROT27	6.37	0.45	0.45	0.07	0.61	1.48	1.47	0.01
BHPERI	20.87	2.04	2.04	0.10	2.33	4.89	4.63	0.26
BHDIV10	45.33	1.57	1.57	0.03	1.90	3.81	3.69	0.12
INV24	32.85	2.00	2.00	0.06	2.41	5.33	5.13	0.20
CR20	19.31	-0.85	1.03	0.05	1.30	2.85	2.79	0.06
CRBH20	46.13	4.76	4.76	0.10	4.81	8.94	5.66	3.28
TMBH17	12.76	1.05	2.60	0.20	3.22	6.36	6.02	0.34
LTMBH26	9.98	0.12	1.06	0.11	1.73	7.32	7.22	0.10
BH76	18.61	-0.36	1.78	0.10	2.43	9.48	9.40	0.08
ISO34	14.57	0.90	0.90	0.06	1.38	4.11	4.10	0.01
ICONF	3.27	0.43	0.43	0.13	0.54	1.11	1.11	0.00
ACONF	1.83	0.05	0.05	0.03	0.06	0.14	0.13	0.01
TAUT15	3.05	0.84	0.84	0.28	1.16	2.34	2.32	0.02
Amino20x4	2.44	0.23	0.23	0.10	0.31	0.86	0.86	0.00
PCONF	1.62	0.46	0.46	0.28	0.61	1.34	1.33	0.01
MCONF	4.97	0.59	0.59	0.12	0.66	1.13	1.12	0.01
SCONF	4.60	3.28	3.28	0.71	3.60	6.92	6.82	0.10
PArel	4.63	0.47	1.01	0.22	1.26	3.44	3.38	0.06
BUT14DIOL	2.80	0.72	0.72	0.26	0.73	0.93	0.88	0.05
EIE22	5.44	0.61	0.63	0.12	0.72	1.55	1.51	0.04
Styrene45	62.64	2.07	2.46	0.04	2.97	8.22	8.22	0.00
ISOMERIZATION20	31.84	-1.51	1.97	0.06	2.75	8.49	8.47	0.02
DIE60	4.71	0.69	0.70	0.15	0.83	1.73	1.73	0.00
IDISP	14.22	4.39	4.39	0.31	8.39	20.74	20.24	0.50
C20C24	30.77	-4.54	10.21	0.33	13.75	24.73	24.71	0.02
S66	5.47	-0.78	0.78	0.14	0.98	2.61	2.60	0.01
S10x8	6.59	0.11	0.36	0.05	0.56	2.25	2.25	0.00
X40	3.76	0.53	0.53	0.14	0.63	1.66	1.60	0.06
HEAVY28	1.24	0.05	0.23	0.19	0.35	1.07	1.07	0.00
CHB6	26.79	0.77	0.77	0.03	1.00	1.74	1.65	0.09
AHB21	22.49	-1.52	1.56	0.07	2.08	5.03	4.98	0.05
IL16	109.04	0.74	0.88	0.01	1.05	2.02	1.95	0.07
PNICO23	4.27	0.25	0.33	0.08	0.41	0.85	0.82	0.03
CT20	0.98	0.18	0.18	0.19	0.20	0.33	0.33	0.00
CARBHB12	6.04	1.47	1.47	0.24	1.63	2.89	2.56	0.33
ADIM6	3.36	0.66	0.66	0.20	0.73	1.47	1.21	0.26
3B-69-TRIM	12.30	1.24	1.41	0.11	1.72	4.76	4.64	0.12

Table S68: Statistical analysis for DS1TPSS for all testset in our databsase. The numbers given (all in kcal/mol) are average reaction energy ($|\overline{\Delta E}|$), mean deviation (MD), mean absolute deviation (MAD), MAD normalized with respect to $|\overline{\Delta E}|$ (NMAD), root-mean-square deviation (RMSD), deviation span (Δ_{err}), maximum (max) and minimum deviation (min).

Test set	$\|\overline{\Delta E}\|$	MD	MAD	NMAD	RMSD	Δ_{err}	max	min
FH51	31.01	-1.27	1.87	0.06	2.41	5.52	5.47	0.05
YBDE18	49.28	1.84	2.99	0.06	3.72	10.12	10.12	0.00
AL2X6	35.88	1.24	1.29	0.04	1.49	2.47	2.36	0.11
DARC	32.47	-5.20	5.20	0.16	5.32	10.55	6.66	3.89
NBPRC	27.71	-1.15	2.07	0.07	2.29	3.99	3.76	0.23
HEAVYSB9	58.02	1.40	2.07	0.04	2.19	4.76	3.79	0.97
BSR36	16.20	2.29	2.30	0.14	3.28	9.77	9.74	0.03
RSE43	7.60	0.96	0.97	0.13	1.95	8.26	8.26	0.00
W4-11	306.91	-0.41	3.02	0.01	3.92	12.55	12.55	0.00
G21EA	33.62	-5.65	5.65	0.17	6.24	13.50	12.15	1.35
G21IP	257.61	-0.82	2.15	0.01	2.78	8.45	8.34	0.11
DIPCS10	654.26	-2.79	3.29	0.01	4.15	8.11	7.63	0.48
PA26	189.05	0.60	1.58	0.01	2.11	5.16	5.09	0.07
SIE4x4	33.72	3.82	3.88	0.12	4.63	9.15	8.63	0.52
ALKBDE10	100.69	2.00	5.15	0.05	6.09	10.35	10.20	0.15
RC21	35.70	-0.32	2.39	0.07	2.78	6.09	6.00	0.09
ALK8	62.60	2.64	2.90	0.05	3.93	7.21	6.84	0.37
DC13	54.98	-0.01	5.17	0.09	6.85	12.69	12.66	0.03
G2RC	51.26	-1.95	3.25	0.06	4.11	9.57	9.31	0.26
BH76RC	21.39	-0.42	1.53	0.07	1.91	5.55	5.53	0.02
MOR23	35.57	-6.41	8.73	0.25	11.11	24.51	23.95	0.56
WCPT18	34.99	3.86	3.86	0.11	4.37	7.51	7.46	0.05
BHROT27	6.37	0.23	0.25	0.04	0.34	1.04	1.04	0.00
BHPERI	20.87	-4.51	4.56	0.22	4.91	9.52	8.82	0.70
BHDIV10	45.33	-1.63	2.44	0.05	2.63	5.87	4.41	1.46
INV24	32.85	-0.78	1.87	0.06	2.42	5.46	5.44	0.02
CR20	19.31	-1.01	1.08	0.06	1.34	2.87	2.75	0.12
CRBH20	46.13	3.10	3.10	0.07	3.14	5.77	3.93	1.84
TMBH17	12.76	-1.18	2.65	0.21	3.09	6.56	6.14	0.42
LTMBH26	9.98	-0.67	1.35	0.14	1.78	4.45	4.30	0.15
BH76	18.61	-1.04	2.28	0.12	2.94	8.93	8.81	0.12
ISO34	14.57	1.09	1.09	0.07	1.42	4.37	4.36	0.01
ICONF	3.27	0.30	0.33	0.10	0.39	0.71	0.69	0.02
ACONF	1.83	-0.10	0.10	0.06	0.12	0.29	0.28	0.01
TAUT15	3.05	0.83	0.83	0.27	1.14	2.13	2.11	0.02
Amino20x4	2.44	0.07	0.25	0.10	0.31	0.77	0.77	0.00
PCONF	1.62	0.64	0.64	0.39	0.79	1.79	1.77	0.02
MCONF	4.97	0.86	0.86	0.17	0.95	1.67	1.63	0.04

Continued on next page

Test set	$\lvert\overline{\Delta E}\rvert$	MD	MAD	NMAD	RMSD	Δ_{err}	max	min
SCONF	4.60	1.02	1.02	0.22	1.12	1.95	1.95	0.00
PArel	4.63	0.29	1.14	0.25	1.47	4.15	4.09	0.06
BUT14DIOL	2.80	0.58	0.58	0.21	0.60	0.76	0.73	0.03
EIE22	5.44	0.70	0.72	0.13	0.80	1.60	1.60	0.00
Styrene45	62.64	0.39	2.11	0.03	2.58	5.93	5.93	0.00
ISOMERIZATION20	31.84	-0.79	2.15	0.07	2.76	7.09	7.00	0.09
DIE60	4.71	0.80	0.80	0.17	0.88	1.79	1.56	0.23
IDISP	14.22	6.96	6.96	0.49	11.15	27.23	26.22	1.01
C20C24	30.77	-8.52	11.78	0.38	13.81	29.77	25.00	4.77
S66	5.47	-1.26	1.26	0.23	1.38	3.57	3.31	0.26
S10x8	6.59	0.65	0.65	0.10	0.80	2.08	2.07	0.01
X40	3.76	0.78	0.78	0.21	0.86	1.80	1.76	0.04
HEAVY28	1.24	-0.03	0.22	0.18	0.34	1.03	1.02	0.01
CHB6	26.79	1.60	1.60	0.06	1.69	3.55	2.44	1.11
AHB21	22.49	-0.51	0.85	0.04	1.11	2.63	2.55	0.08
IL16	109.04	1.32	1.35	0.01	1.55	2.52	2.46	0.06
PNICO23	4.27	0.14	0.25	0.06	0.35	0.97	0.96	0.01
CT20	0.98	0.42	0.42	0.42	0.43	0.91	0.62	0.29
CARBHB12	6.04	1.20	1.20	0.20	1.37	2.46	2.26	0.20
ADIM6	3.36	0.70	0.70	0.21	0.79	1.55	1.34	0.21
3B-69-TRIM	12.30	2.44	2.44	0.20	2.65	5.39	5.39	0.00

Table S69: Statistical analysis for DH-BLYP for all testset in our databsase. The numbers given (all in kcal/mol) are average reaction energy ($\lvert\overline{\Delta E}\rvert$), mean deviation (MD), mean absolute deviation (MAD), MAD normalized with respect to $\lvert\overline{\Delta E}\rvert$ (NMAD), root-mean-square deviation (RMSD), deviation span (Δ_{err}), maximum (max) and minimum deviation (min).

Test set	$\lvert\overline{\Delta E}\rvert$	MD	MAD	NMAD	RMSD	Δ_{err}	max	min
FH51	31.01	1.30	1.30	0.04	1.71	3.82	3.76	0.06
YBDE18	49.28	-1.15	2.54	0.05	2.93	7.01	6.50	0.51
AL2X6	35.88	-2.56	2.56	0.07	2.89	5.01	4.38	0.63
DARC	32.47	2.94	2.94	0.09	3.02	5.98	4.03	1.95
NBPRC	27.71	1.02	2.28	0.08	2.78	6.03	5.63	0.40
HEAVYSB9	58.02	-1.77	1.77	0.03	2.44	4.91	4.74	0.17
BSR36	16.20	-0.55	0.67	0.04	0.71	1.52	1.32	0.20
RSE43	7.60	0.21	0.50	0.07	0.93	4.33	4.33	0.00
W4-11	306.91	-1.30	3.05	0.01	3.85	11.80	11.79	0.01
G21EA	33.62	-5.81	5.82	0.17	6.12	10.68	10.47	0.21
G21IP	257.61	-1.32	2.38	0.01	2.86	8.36	8.36	0.00
DIPCS10	654.26	-3.75	3.75	0.01	4.34	8.44	8.05	0.39
PA26	189.05	0.11	1.34	0.01	1.87	5.16	5.11	0.05
SIE4x4	33.72	6.70	6.73	0.20	7.83	14.85	14.60	0.25

Continued on next page

Test set	$\lvert\overline{\Delta E}\rvert$	MD	MAD	NMAD	RMSD	Δ_{err}	max	min
ALKBDE10	100.69	2.47	5.05	0.05	5.89	11.02	9.59	1.43
RC21	35.70	-1.53	1.83	0.05	2.27	5.08	4.99	0.09
ALK8	62.60	-1.28	1.95	0.03	2.60	5.33	5.24	0.09
DC13	54.98	2.54	6.61	0.12	9.32	23.58	23.32	0.26
G2RC	51.26	-0.46	1.94	0.04	2.65	7.92	7.77	0.15
BH76RC	21.39	-0.35	1.16	0.05	1.53	4.93	4.78	0.15
MOR23	35.57	-1.86	5.17	0.15	7.05	18.97	18.90	0.07
WCPT18	34.99	2.68	2.68	0.08	3.17	5.73	5.68	0.05
BHROT27	6.37	0.19	0.19	0.03	0.29	1.01	1.00	0.01
BHPERI	20.87	1.69	1.69	0.08	1.93	4.79	4.61	0.18
BHDIV10	45.33	2.00	2.00	0.04	2.45	4.53	4.51	0.02
INV24	32.85	1.77	1.77	0.05	2.30	5.23	5.20	0.03
CR20	19.31	-6.22	6.22	0.32	6.28	12.09	7.72	4.37
CRBH20	46.13	-2.22	2.22	0.05	2.30	4.60	3.62	0.98
TMBH17	12.76	-0.28	2.90	0.23	3.56	8.50	8.45	0.05
LTMBH26	9.98	-1.19	1.82	0.18	2.48	6.73	6.58	0.15
BH76	18.61	-1.73	2.36	0.13	2.93	8.14	7.94	0.20
ISO34	14.57	1.00	1.00	0.07	1.55	6.51	6.49	0.02
ICONF	3.27	0.33	0.33	0.10	0.40	0.87	0.83	0.04
ACONF	1.83	0.13	0.13	0.07	0.15	0.26	0.24	0.02
TAUT15	3.05	0.75	0.75	0.25	0.94	1.83	1.82	0.01
Amino20x4	2.44	0.17	0.17	0.07	0.23	0.62	0.62	0.00
PCONF	1.62	0.55	0.55	0.34	0.65	1.17	1.14	0.03
MCONF	4.97	0.21	0.21	0.04	0.27	0.65	0.64	0.01
SCONF	4.60	3.28	3.28	0.71	3.60	6.92	6.82	0.10
PArel	4.63	-0.02	0.80	0.17	1.13	2.86	2.85	0.01
BUT14DIOL	2.80	0.51	0.51	0.18	0.52	0.79	0.71	0.08
EIE22	5.44	0.79	0.79	0.15	0.88	1.71	1.70	0.01
Styrene45	62.64	2.35	3.23	0.05	4.10	11.57	11.57	0.00
ISOMERIZATION20	31.84	-0.52	1.94	0.06	2.54	6.31	6.27	0.04
DIE60	4.71	0.73	0.73	0.15	0.78	1.51	1.26	0.25
IDISP	14.22	2.04	2.04	0.14	3.84	9.25	9.24	0.01
C20C24	30.77	-1.04	28.64	0.93	31.50	62.48	49.34	13.14
S66	5.47	-1.65	1.65	0.30	1.84	4.73	4.40	0.33
S10x8	6.59	0.87	0.87	0.13	1.14	4.17	4.14	0.03
X40	3.76	1.04	1.04	0.28	1.20	3.22	3.02	0.20
HEAVY28	1.24	-0.33	0.39	0.32	0.47	1.36	1.33	0.03
CHB6	26.79	1.47	1.47	0.05	1.72	3.23	2.81	0.42
AHB21	22.49	-0.20	0.70	0.03	0.85	1.91	1.86	0.05
IL16	109.04	1.91	1.91	0.02	2.07	3.81	3.28	0.53
PNICO23	4.27	-0.54	0.55	0.13	0.65	1.57	1.55	0.02
CT20	0.98	0.39	0.39	0.40	0.40	0.78	0.60	0.18
CARBHB12	6.04	0.76	0.76	0.13	0.90	1.59	1.57	0.02
ADIM6	3.36	-0.25	0.25	0.07	0.26	0.47	0.32	0.15

Continued on next page

Test set	$\|\overline{\Delta E}\|$	MD	MAD	NMAD	RMSD	Δ_{err}	max	min
3B-69-TRIM	12.30	3.00	3.00	0.24	3.27	7.48	6.85	0.63

Table S70: Statistical analysis for PBE-QIDH for all testset in our databsase. The numbers given (all in kcal/mol) are average reaction energy ($|\overline{\Delta E}|$), mean deviation (MD), mean absolute deviation (MAD), MAD normalized with respect to $|\overline{\Delta E}|$ (NMAD), root-mean-square deviation (RMSD), deviation span (Δ_{err}), maximum (max) and minimum deviation (min).

Test set	$\|\overline{\Delta E}\|$	MD	MAD	NMAD	RMSD	Δ_{err}	max	min
FH51	31.01	-2.66	3.12	0.10	3.96	12.26	12.25	0.01
YBDE18	49.28	0.79	2.82	0.06	3.08	5.44	5.29	0.15
AL2X6	35.88	0.88	1.13	0.03	1.17	2.04	1.42	0.62
DARC	32.47	-7.79	7.79	0.24	7.92	15.94	9.65	6.29
NBPRC	27.71	-2.21	2.21	0.08	2.38	4.47	3.65	0.82
HEAVYSB9	58.02	-0.96	1.16	0.02	1.53	3.10	2.98	0.12
BSR36	16.20	-0.74	0.81	0.05	0.85	1.32	1.31	0.01
RSE43	7.60	0.44	0.53	0.07	0.88	3.86	3.83	0.03
W4-11	306.91	-6.79	6.89	0.02	8.32	23.65	23.57	0.08
G21EA	33.62	-5.59	5.59	0.17	6.68	13.03	12.87	0.16
G21IP	257.61	-0.08	2.49	0.01	3.13	8.99	8.92	0.07
DIPCS10	654.26	-0.89	3.15	0.00	3.64	7.00	6.21	0.79
PA26	189.05	1.10	1.73	0.01	2.25	5.89	5.79	0.10
SIE4x4	33.72	2.75	2.78	0.08	3.54	8.44	8.22	0.22
ALKBDE10	100.69	41.95	41.95	0.42	47.63	90.59	75.01	15.58
RC21	35.70	2.25	2.96	0.08	4.16	12.49	12.09	0.40
ALK8	62.60	3.53	3.53	0.06	4.18	7.85	7.07	0.78
DC13	54.98	-2.18	7.87	0.14	9.33	17.19	17.14	0.05
G2RC	51.26	-4.56	5.74	0.11	7.07	18.37	17.83	0.54
BH76RC	21.39	-0.52	1.95	0.09	2.54	6.49	6.36	0.13
MOR23	35.57	-3.43	4.35	0.12	5.82	13.60	13.58	0.02
WCPT18	34.99	-2.31	2.63	0.08	3.30	5.86	5.84	0.02
BHROT27	6.37	0.44	0.45	0.07	0.59	1.25	1.25	0.00
BHPERI	20.87	-1.82	2.40	0.12	2.74	4.45	4.36	0.09
BHDIV10	45.33	-0.78	1.71	0.04	1.96	3.59	3.24	0.35
INV24	32.85	0.04	1.84	0.06	2.36	6.22	6.16	0.06
CR20	19.31	1.34	1.36	0.07	1.54	2.42	2.31	0.11
CRBH20	46.13	5.83	5.83	0.13	5.87	11.34	6.87	4.47
TMBH17	12.76	-7.54	13.21	1.04	17.26	46.82	46.59	0.23
LTMBH26	9.98	0.15	1.30	0.13	2.13	8.93	8.91	0.02
BH76	18.61	-0.02	1.56	0.08	2.23	8.88	8.87	0.01
ISO34	14.57	0.18	1.25	0.09	1.82	4.03	4.01	0.02
ICONF	3.27	0.39	0.44	0.14	0.55	1.28	1.16	0.12
ACONF	1.83	0.12	0.12	0.07	0.15	0.34	0.31	0.03

Continued on next page

Test set	$\lvert\overline{\Delta E}\rvert$	MD	MAD	NMAD	RMSD	Δ_{err}	max	min
TAUT15	3.05	0.69	0.81	0.27	1.05	1.97	1.96	0.01
Amino20x4	2.44	0.09	0.21	0.09	0.27	0.71	0.70	0.01
PCONF	1.62	0.11	0.59	0.36	0.71	1.16	1.13	0.03
MCONF	4.97	0.03	0.20	0.04	0.24	0.58	0.58	0.00
SCONF	4.60	0.38	0.54	0.12	0.61	0.97	0.96	0.01
PArel	4.63	0.67	1.14	0.25	1.40	3.37	3.28	0.09
BUT14DIOL	2.80	0.41	0.41	0.15	0.42	0.54	0.52	0.02
EIE22	5.44	0.64	0.67	0.12	0.77	1.67	1.62	0.05
Styrene45	62.64	0.62	2.97	0.05	3.73	8.59	8.59	0.00
ISOMERIZATION20	31.84	-0.93	1.88	0.06	2.59	7.68	7.68	0.00
DIE60	4.71	0.82	0.83	0.18	1.00	2.18	2.17	0.01
IDISP	14.22	-3.19	3.56	0.25	7.48	18.28	18.21	0.07
C20C24	30.77	-8.47	8.47	0.28	9.86	18.85	16.85	2.00
S66	5.47	-1.27	1.27	0.23	1.51	3.80	3.75	0.05
S10x8	6.59	0.49	0.53	0.08	0.78	3.13	3.12	0.01
X40	3.76	0.76	0.81	0.22	0.96	2.74	2.68	0.06
HEAVY28	1.24	-0.18	0.30	0.24	0.39	1.17	1.16	0.01
CHB6	26.79	-1.02	1.02	0.04	1.35	2.92	2.72	0.20
AHB21	22.49	-3.27	3.27	0.15	4.03	9.74	9.08	0.66
IL16	109.04	-1.18	1.19	0.01	1.39	2.41	2.32	0.09
PNICO23	4.27	0.07	0.25	0.06	0.40	1.19	1.19	0.00
CT20	0.98	0.39	0.39	0.40	0.41	0.86	0.62	0.24
CARBHB12	6.04	1.18	1.18	0.20	1.37	2.62	2.43	0.19
ADIM6	3.36	-0.44	0.44	0.13	0.48	0.80	0.63	0.17
3B-69-TRIM	12.30	2.39	2.40	0.19	2.77	6.38	6.38	0.00

Table S71: Statistical analysis for SOS1-PBE-QIDH for all testset in our databsase. The numbers given (all in kcal/mol) are average reaction energy ($\lvert\overline{\Delta E}\rvert$), mean deviation (MD), mean absolute deviation (MAD), MAD normalized with respect to $\lvert\overline{\Delta E}\rvert$ (NMAD), root-mean-square deviation (RMSD), deviation span (Δ_{err}), maximum (max) and minimum deviation (min).

Test set	$\lvert\overline{\Delta E}\rvert$	MD	MAD	NMAD	RMSD	Δ_{err}	max	min
FH51	31.01	-1.94	2.62	0.08	3.50	13.62	13.61	0.01
YBDE18	49.28	-0.75	1.86	0.04	2.14	3.62	3.52	0.10
AL2X6	35.88	-0.37	0.77	0.02	1.05	2.09	2.00	0.09
DARC	32.47	-6.06	6.06	0.19	6.13	12.71	7.61	5.10
NBPRC	27.71	-1.47	1.83	0.07	1.96	3.38	2.56	0.82
HEAVYSB9	58.02	-2.38	2.38	0.04	2.75	4.64	4.49	0.15
BSR36	16.20	-3.48	3.48	0.21	3.76	8.38	6.83	1.55
RSE43	7.60	0.81	0.83	0.11	1.15	4.18	4.16	0.02
W4-11	306.91	-8.53	8.56	0.03	10.37	28.80	28.78	0.02
G21EA	33.62	-6.23	6.23	0.19	7.13	13.92	12.74	1.18

Continued on next page

Test set	$\|\overline{\Delta E}\|$	MD	MAD	NMAD	RMSD	Δ_{err}	max	min
G21IP	257.61	-0.41	2.21	0.01	2.79	6.75	6.73	0.02
DIPCS10	654.26	-1.42	3.16	0.00	3.79	7.33	7.32	0.01
PA26	189.05	2.42	2.69	0.01	3.12	7.59	6.88	0.71
SIE4x4	33.72	2.23	2.49	0.07	3.54	9.32	9.27	0.05
ALKBDE10	100.69	39.27	39.27	0.39	44.85	84.09	70.77	13.32
RC21	35.70	1.24	1.80	0.05	2.80	8.45	8.25	0.20
ALK8	62.60	2.27	2.27	0.04	2.76	6.71	5.78	0.93
DC13	54.98	-2.90	6.05	0.11	7.40	14.66	14.23	0.43
G2RC	51.26	-3.67	4.46	0.09	5.70	12.67	12.54	0.13
BH76RC	21.39	-0.39	1.85	0.09	2.36	5.60	5.60	0.00
MOR23	35.57	-0.10	1.82	0.05	2.38	6.18	6.16	0.02
WCPT18	34.99	-0.33	1.67	0.05	1.98	3.94	3.86	0.08
BHROT27	6.37	0.27	0.32	0.05	0.40	0.73	0.73	0.00
BHPERI	20.87	1.56	1.60	0.08	2.17	6.30	6.25	0.05
BHDIV10	45.33	0.64	1.17	0.03	1.29	2.21	1.82	0.39
INV24	32.85	0.38	1.97	0.06	2.82	8.98	8.82	0.16
CR20	19.31	0.51	0.79	0.04	0.94	1.88	1.88	0.00
CRBH20	46.13	5.58	5.58	0.12	5.65	11.13	6.96	4.17
TMBH17	12.76	-0.50	9.12	0.71	14.81	38.25	38.22	0.03
LTMBH26	9.98	0.71	1.25	0.13	2.44	11.19	11.11	0.08
BH76	18.61	0.84	1.46	0.08	2.33	9.20	9.18	0.02
ISO34	14.57	-0.16	1.05	0.07	1.48	3.34	3.28	0.06
ICONF	3.27	0.28	0.36	0.11	0.44	0.98	0.92	0.06
ACONF	1.83	0.25	0.25	0.14	0.28	0.60	0.51	0.09
TAUT15	3.05	0.14	0.58	0.19	0.74	1.91	1.87	0.04
Amino20x4	2.44	0.02	0.18	0.07	0.23	0.69	0.69	0.00
PCONF	1.62	0.09	0.79	0.49	0.96	1.75	1.74	0.01
MCONF	4.97	-0.44	0.44	0.09	0.46	0.75	0.66	0.09
SCONF	4.60	0.10	0.22	0.05	0.26	0.48	0.45	0.03
PArel	4.63	0.44	0.79	0.17	1.05	2.57	2.56	0.01
BUT14DIOL	2.80	0.20	0.20	0.07	0.22	0.42	0.40	0.02
EIE22	5.44	0.36	0.39	0.07	0.45	1.10	1.08	0.02
Styrene45	62.64	-0.71	2.09	0.03	2.87	8.02	8.02	0.00
ISOMERIZATION20	31.84	-0.23	1.39	0.04	2.04	6.63	6.48	0.15
DIE60	4.71	0.51	0.57	0.12	0.81	1.94	1.94	0.00
IDISP	14.22	-2.25	4.19	0.29	5.38	11.67	11.02	0.65
C20C24	30.77	-7.23	7.23	0.23	11.09	21.63	21.09	0.54
S66	5.47	-1.88	1.88	0.34	2.14	5.67	5.23	0.44
S10x8	6.59	0.96	0.96	0.15	1.28	4.85	4.82	0.03
X40	3.76	1.18	1.18	0.31	1.40	3.96	3.95	0.01
HEAVY28	1.24	-0.41	0.46	0.37	0.53	1.56	1.42	0.14
CHB6	26.79	-0.88	0.88	0.03	1.28	2.71	2.66	0.05
AHB21	22.49	-2.66	2.66	0.12	3.47	8.54	8.35	0.19
IL16	109.04	-0.25	0.69	0.01	0.78	1.48	1.32	0.16

Continued on next page

Test set	$\lvert\overline{\Delta E}\rvert$	MD	MAD	NMAD	RMSD	Δ_{err}	max	min
PNICO23	4.27	-0.38	0.40	0.09	0.52	1.78	1.70	0.08
CT20	0.98	0.53	0.53	0.54	0.55	1.31	0.97	0.34
CARBHB12	6.04	0.72	0.72	0.12	0.86	1.77	1.69	0.08
ADIM6	3.36	-1.02	1.02	0.30	1.13	2.02	1.68	0.34
3B-69-TRIM	12.30	3.54	3.54	0.29	3.91	8.88	8.23	0.65
ISOL24	21.92	2.31	5.20	0.24	7.16	16.52	16.00	0.52
C60ISO	98.25	4.24	4.24	0.04	4.75	9.55	8.61	0.94
L7	18.20	5.22	6.40	0.35	8.11	14.44	13.75	0.69
UPU23	5.72	0.30	0.57	0.10	0.72	1.65	1.63	0.02
ENZYMES23	15.32	1.11	2.00	0.13	2.85	9.40	9.34	0.06

Table S72: Statistical analysis for SDSCAN69 for all testset in our databsase. The numbers given (all in kcal/mol) are average reaction energy ($\lvert\overline{\Delta E}\rvert$), mean deviation (MD), mean absolute deviation (MAD), MAD normalized with respect to $\lvert\overline{\Delta E}\rvert$ (NMAD), root-mean-square deviation (RMSD), deviation span (Δ_{err}), maximum (max) and minimum deviation (min).

Test set	$\lvert\overline{\Delta E}\rvert$	MD	MAD	NMAD	RMSD	Δ_{err}	max	min
FH51	31.01	1.73	1.73	0.06	2.27	6.96	6.93	0.03
YBDE18	49.28	1.11	2.33	0.05	3.09	9.59	9.47	0.12
AL2X6	35.88	1.15	1.38	0.04	1.65	2.99	2.64	0.35
DARC	32.47	-4.62	4.62	0.14	4.71	9.01	5.96	3.05
NBPRC	27.71	-0.86	1.64	0.06	1.89	3.54	3.43	0.11
HEAVYSB9	58.02	0.11	1.37	0.02	1.81	3.91	3.87	0.04
BSR36	16.20	2.15	2.18	0.13	2.84	8.82	8.76	0.06
RSE43	7.60	1.47	1.47	0.19	2.35	9.87	9.66	0.21
W4-11	306.91	-2.81	3.57	0.01	4.37	10.49	10.49	0.00
G21EA	33.62	-6.25	6.25	0.19	6.72	12.17	11.49	0.68
G21IP	257.61	-1.55	2.48	0.01	3.04	8.74	8.70	0.04
DIPCS10	654.26	-4.26	4.26	0.01	5.03	9.73	9.23	0.50
PA26	189.05	0.98	1.47	0.01	1.96	5.37	5.31	0.06
SIE4x4	33.72	3.76	3.76	0.11	4.64	9.43	9.14	0.29
ALKBDE10	100.69	1.02	4.62	0.05	5.46	7.94	7.85	0.09
RC21	35.70	-0.93	2.00	0.06	2.56	5.60	5.59	0.01
ALK8	62.60	1.90	2.36	0.04	3.24	6.59	6.41	0.18
DC13	54.98	-0.46	3.22	0.06	4.39	9.62	9.07	0.55
G2RC	51.26	-1.37	2.66	0.05	3.16	6.90	6.80	0.10
BH76RC	21.39	-0.20	1.34	0.06	1.77	5.16	5.12	0.04
MOR23	35.57	-5.25	7.07	0.20	8.98	20.41	20.07	0.34
WCPT18	34.99	3.28	3.28	0.09	3.88	7.13	6.92	0.21
BHROT27	6.37	0.18	0.18	0.03	0.26	0.81	0.81	0.00
BHPERI	20.87	3.17	3.17	0.15	3.46	8.07	7.03	1.04
BHDIV10	45.33	1.74	1.74	0.04	1.98	3.34	3.16	0.18

Continued on next page

Test set	$\lvert\overline{\Delta E}\rvert$	MD	MAD	NMAD	RMSD	Δ_{err}	max	min
INV24	32.85	1.64	1.64	0.05	2.10	4.35	4.31	0.04
CR20	19.31	-1.15	1.19	0.06	1.41	2.71	2.71	0.00
CRBH20	46.13	1.70	1.70	0.04	1.78	3.38	2.81	0.57
TMBH17	12.76	-0.75	2.51	0.20	2.90	5.25	5.05	0.20
LTMBH26	9.98	-0.87	1.44	0.14	1.88	4.99	4.97	0.02
BH76	18.61	-0.79	2.07	0.11	2.79	8.99	8.98	0.01
ISO34	14.57	0.74	0.74	0.05	0.93	2.19	2.16	0.03
ICONF	3.27	0.27	0.27	0.08	0.32	0.61	0.60	0.01
ACONF	1.83	0.13	0.13	0.07	0.15	0.33	0.32	0.01
TAUT15	3.05	0.78	0.78	0.26	0.95	2.18	2.16	0.02
Amino20x4	2.44	0.21	0.21	0.09	0.26	0.71	0.70	0.01
PCONF	1.62	0.90	0.90	0.56	1.20	2.35	2.33	0.02
MCONF	4.97	1.01	1.01	0.20	1.14	1.80	1.80	0.00
SCONF	4.60	0.84	0.84	0.18	0.92	1.63	1.63	0.00
PArel	4.63	0.10	0.89	0.19	1.23	3.48	3.47	0.01
BUT14DIOL	2.80	0.60	0.60	0.21	0.62	0.82	0.81	0.01
EIE22	5.44	0.52	0.53	0.10	0.60	1.23	1.22	0.01
Styrene45	62.64	0.01	1.14	0.02	1.42	3.95	3.95	0.00
ISOMERIZATION20	31.84	-0.76	1.69	0.05	2.02	4.22	4.10	0.12
DIE60	4.71	0.52	0.52	0.11	0.61	1.27	1.18	0.09
IDISP	14.22	6.46	6.46	0.45	9.65	23.39	22.22	1.17
C20C24	30.77	-5.47	10.96	0.36	13.71	27.82	25.05	2.77
S66	5.47	-1.00	1.00	0.18	1.09	2.68	2.42	0.26
S10x8	6.59	0.59	0.59	0.09	0.75	1.75	1.75	0.00
X40	3.76	0.59	0.59	0.16	0.64	1.35	1.22	0.13
HEAVY28	1.24	0.02	0.19	0.15	0.28	0.80	0.80	0.00
CHB6	26.79	1.75	1.75	0.07	1.82	3.48	2.39	1.09
AHB21	22.49	-0.40	0.70	0.03	0.91	2.19	2.18	0.01
IL16	109.04	1.26	1.26	0.01	1.44	2.39	2.24	0.15
PNICO23	4.27	0.20	0.26	0.06	0.33	0.68	0.68	0.00
CT20	0.98	0.28	0.28	0.29	0.29	0.60	0.42	0.18
CARBHB12	6.04	1.12	1.12	0.19	1.25	2.19	1.95	0.24
ADIM6	3.36	1.26	1.26	0.38	1.39	2.71	2.21	0.50
3B-69-TRIM	12.30	1.94	1.94	0.16	2.06	4.33	3.75	0.58

S5.4 D3-Dispersion-Corrected Hybrids

Table S73: Statistical analysis for B3LYP-D3(BJ) for all testset in our databsase. The numbers given (all in kcal/mol) are average reaction energy ($|\overline{\Delta E}|$), mean deviation (MD), mean absolute deviation (MAD), MAD normalized with respect to $|\overline{\Delta E}|$ (NMAD), root-mean-square deviation (RMSD), deviation span (Δ_{err}), maximum (max) and minimum deviation (min).

Test set	$\lvert\overline{\Delta E}\rvert$	MD	MAD	NMAD	RMSD	Δ_{err}	max	min
FH51	31.01	2.61	2.61	0.08	3.48	8.84	8.77	0.07
YBDE18	49.28	-5.19	5.50	0.11	6.42	13.16	11.96	1.20
AL2X6	35.88	-2.84	2.84	0.08	2.92	5.73	3.71	2.02
DARC	32.47	7.95	7.95	0.24	8.11	12.51	9.07	3.44
NBPRC	27.71	1.61	2.57	0.09	3.73	9.11	9.10	0.01
HEAVYSB9	58.02	-3.69	3.73	0.06	4.28	7.06	6.90	0.16
BSR36	16.20	-3.22	3.22	0.20	3.79	10.07	9.00	1.07
RSE43	7.60	-1.50	1.50	0.20	1.79	4.27	4.22	0.05
W4-11	306.91	-2.63	3.96	0.01	5.68	28.17	28.11	0.06
G21EA	33.62	-3.82	4.40	0.13	5.15	11.21	10.52	0.69
G21IP	257.61	0.37	3.51	0.01	4.41	10.19	10.18	0.01
DIPCS10	654.26	-1.15	4.09	0.01	5.14	9.94	9.15	0.79
PA26	189.05	1.80	2.19	0.01	3.09	8.57	8.57	0.00
SIE4x4	33.72	17.51	17.51	0.52	20.24	42.49	40.56	1.93
ALKBDE10	100.69	45.44	45.44	0.45	51.49	95.72	79.07	16.65
RC21	35.70	1.31	2.33	0.07	2.85	5.68	5.64	0.04
ALK8	62.60	0.74	2.85	0.05	3.69	7.24	7.17	0.07
DC13	54.98	2.31	11.62	0.21	14.70	30.61	30.18	0.43
G2RC	51.26	0.66	2.64	0.05	3.34	8.57	8.40	0.17
BH76RC	21.39	-0.59	2.21	0.10	2.85	7.37	7.37	0.00
MOR23	35.57	2.31	6.62	0.19	14.58	63.05	62.97	0.08
WCPT18	34.99	2.51	2.51	0.07	3.19	6.44	6.40	0.04
BHROT27	6.37	0.45	0.45	0.07	0.60	1.30	1.30	0.00
BHPERI	20.87	1.17	1.17	0.06	1.56	4.56	4.51	0.05
BHDIV10	45.33	3.17	3.17	0.07	3.82	5.88	5.69	0.19
INV24	32.85	1.02	1.02	0.03	1.35	3.25	3.13	0.12
CR20	19.31	-7.29	7.29	0.38	7.36	14.46	8.97	5.49
CRBH20	46.13	-8.69	8.69	0.19	8.73	18.09	10.46	7.63
TMBH17	12.76	1.43	3.42	0.27	5.15	14.56	14.31	0.25
LTMBH26	9.98	-2.68	4.02	0.40	5.31	16.11	15.91	0.20
BH76	18.61	-5.70	5.83	0.31	6.44	12.14	11.75	0.39
ISO34	14.57	1.89	1.89	0.13	2.76	10.65	10.64	0.01
ICONF	3.27	0.31	0.31	0.09	0.43	1.08	1.04	0.04
ACONF	1.83	0.07	0.07	0.04	0.09	0.24	0.24	0.00
TAUT15	3.05	1.18	1.18	0.39	1.37	2.36	2.31	0.05
Amino20x4	2.44	0.22	0.22	0.09	0.31	1.24	1.24	0.00
PCONF	1.62	0.55	0.55	0.34	0.63	1.17	1.14	0.03
MCONF	4.97	0.25	0.25	0.05	0.29	0.63	0.63	0.00

Continued on next page

Test set	$\lvert\overline{\Delta E}\rvert$	MD	MAD	NMAD	RMSD	Δ_{err}	max	min
SCONF	4.60	0.40	0.40	0.09	0.63	2.10	2.09	0.01
PArel	4.63	-0.10	1.16	0.25	1.67	5.01	5.00	0.01
BUT14DIOL	2.80	0.40	0.40	0.14	0.43	1.00	0.94	0.06
EIE22	5.44	1.25	1.27	0.23	1.41	2.70	2.58	0.12
Styrene45	62.64	4.92	5.93	0.09	7.55	18.72	18.72	0.00
ISOMERIZATION20	31.84	0.21	2.22	0.07	2.92	7.41	7.34	0.07
DIE60	4.71	1.11	1.13	0.24	1.23	2.31	2.11	0.20
IDISP	14.22	3.66	3.66	0.26	5.51	12.78	12.42	0.36
C20C24	30.77	1.94	41.19	1.34	43.17	92.20	62.46	29.74
S66	5.47	-0.15	0.15	0.03	0.24	0.87	0.87	0.00
S10x8	6.59	-0.25	0.31	0.05	0.47	1.44	1.44	0.00
X40	3.76	0.19	0.19	0.05	0.27	0.96	0.95	0.01
HEAVY28	1.24	0.39	0.40	0.32	0.46	1.15	1.14	0.01
CHB6	26.79	0.11	0.71	0.03	0.99	2.06	2.02	0.04
AHB21	22.49	-1.06	1.08	0.05	1.34	2.76	2.69	0.07
IL16	109.04	12.77	12.77	0.12	14.14	20.02	19.50	0.52
PNICO23	4.27	0.38	0.39	0.09	0.47	0.98	0.97	0.01
CT20	0.98	0.10	0.21	0.21	0.25	0.48	0.44	0.04
CARBHB12	6.04	1.12	1.12	0.19	1.29	2.76	2.43	0.33
ADIM6	3.36	0.29	0.29	0.09	0.33	0.57	0.48	0.09
3B-69-TRIM	12.30	-0.11	0.47	0.04	0.63	1.61	1.61	0.00
ISOL24	21.92	-2.33	5.84	0.27	8.22	20.14	20.12	0.02
C60ISO	98.25	0.92	2.35	0.02	2.52	3.54	3.44	0.10
L7	18.20	0.47	1.03	0.06	1.23	1.86	1.81	0.05
UPU23	5.72	0.55	0.72	0.13	0.84	1.69	1.64	0.05
ENZYMES23	15.32	-2.79	3.83	0.25	4.57	8.51	8.51	0.00

Table S74: Statistical analysis for CAMB3LYP-D3(BJ) for all testset in our databsase. The numbers given (all in kcal/mol) are average reaction energy ($\lvert\overline{\Delta E}\rvert$), mean deviation (MD), mean absolute deviation (MAD), MAD normalized with respect to $\lvert\overline{\Delta E}\rvert$ (NMAD), root-mean-square deviation (RMSD), deviation span (Δ_{err}), maximum (max) and minimum deviation (min).

Test set	$\lvert\overline{\Delta E}\rvert$	MD	MAD	NMAD	RMSD	Δ_{err}	max	min
FH51	31.01	2.61	2.61	0.08	3.48	8.84	8.77	0.07
YBDE18	49.28	-5.19	5.50	0.11	6.42	13.16	11.96	1.20
AL2X6	35.88	-2.84	2.84	0.08	2.92	5.73	3.71	2.02
DARC	32.47	7.54	7.54	0.23	7.78	12.51	9.07	3.44
NBPRC	27.71	1.61	2.57	0.09	3.73	9.11	9.10	0.01
HEAVYSB9	58.02	-3.69	3.73	0.06	4.28	7.06	6.90	0.16
BSR36	16.20	-3.22	3.22	0.20	3.79	10.07	9.00	1.07
RSE43	7.60	-1.50	1.50	0.20	1.79	4.27	4.22	0.05
W4-11	306.91	-2.62	3.95	0.01	5.68	28.16	28.11	0.05

Continued on next page

Test set	$\lvert\overline{\Delta E}\rvert$	MD	MAD	NMAD	RMSD	Δ_{err}	max	min
G21EA	33.62	-3.82	4.40	0.13	5.15	11.21	10.52	0.69
G21IP	257.61	0.31	3.46	0.01	4.36	10.19	10.18	0.01
DIPCS10	654.26	-1.15	4.09	0.01	5.14	9.94	9.15	0.79
PA26	189.05	1.80	2.19	0.01	3.09	8.57	8.57	0.00
SIE4x4	33.72	17.51	17.51	0.52	20.24	42.49	40.56	1.93
ALKBDE10	100.69	45.44	45.44	0.45	51.49	95.72	79.07	16.65
RC21	35.70	1.31	2.33	0.07	2.85	5.68	5.64	0.04
ALK8	62.60	0.74	2.85	0.05	3.69	7.24	7.17	0.07
DC13	54.98	2.31	11.62	0.21	14.70	30.61	30.18	0.43
G2RC	51.26	0.66	2.64	0.05	3.34	8.57	8.40	0.17
BH76RC	21.39	-0.59	2.21	0.10	2.85	7.37	7.37	0.00
MOR23	35.57	2.31	6.62	0.19	14.58	63.05	62.97	0.08
WCPT18	34.99	2.51	2.51	0.07	3.19	6.44	6.40	0.04
BHROT27	6.37	0.45	0.45	0.07	0.60	1.30	1.30	0.00
BHPERI	20.87	1.17	1.17	0.06	1.56	4.56	4.51	0.05
BHDIV10	45.33	3.17	3.17	0.07	3.82	5.88	5.69	0.19
INV24	32.85	1.02	1.02	0.03	1.35	3.25	3.13	0.12
CR20	19.31	-7.29	7.29	0.38	7.36	14.46	8.97	5.49
CRBH20	46.13	-8.69	8.69	0.19	8.73	18.09	10.46	7.63
TMBH17	12.76	1.43	3.42	0.27	5.15	14.56	14.31	0.25
LTMBH26	9.98	-2.68	4.02	0.40	5.31	16.11	15.91	0.20
BH76	18.61	-5.70	5.83	0.31	6.44	12.14	11.75	0.39
ISO34	14.57	1.89	1.89	0.13	2.76	10.65	10.64	0.01
ICONF	3.27	0.31	0.31	0.09	0.43	1.08	1.04	0.04
ACONF	1.83	0.07	0.07	0.04	0.09	0.24	0.24	0.00
TAUT15	3.05	1.18	1.18	0.39	1.37	2.36	2.31	0.05
Amino20x4	2.44	0.22	0.22	0.09	0.31	1.24	1.24	0.00
PCONF	1.62	0.55	0.55	0.34	0.63	1.17	1.14	0.03
MCONF	4.97	0.25	0.25	0.05	0.29	0.63	0.63	0.00
SCONF	4.60	0.40	0.40	0.09	0.63	2.10	2.09	0.01
PArel	4.63	-0.10	1.16	0.25	1.67	5.01	5.00	0.01
BUT14DIOL	2.80	0.40	0.40	0.14	0.43	1.00	0.94	0.06
EIE22	5.44	1.25	1.27	0.23	1.41	2.70	2.58	0.12
Styrene45	62.64	4.92	5.93	0.09	7.55	18.72	18.72	0.00
ISOMERIZATION20	31.84	0.21	2.22	0.07	2.92	7.41	7.34	0.07
DIE60	4.71	1.11	1.13	0.24	1.23	2.31	2.11	0.20
IDISP	14.22	3.14	3.65	0.26	5.49	12.74	12.38	0.36
C20C24	30.77	1.96	41.36	1.34	43.34	92.59	62.69	29.90
S66	5.47	-0.22	0.22	0.04	0.59	4.41	4.41	0.00
S10x8	6.59	-0.25	0.31	0.05	0.47	1.44	1.44	0.00
X40	3.76	0.19	0.19	0.05	0.27	0.96	0.95	0.01
HEAVY28	1.24	0.39	0.40	0.32	0.46	1.15	1.14	0.01
CHB6	26.79	0.11	0.71	0.03	0.99	2.06	2.02	0.04
AHB21	22.49	-1.06	1.08	0.05	1.34	2.76	2.69	0.07

Continued on next page

Test set	$\|\overline{\Delta E}\|$	MD	MAD	NMAD	RMSD	Δ_{err}	max	min
IL16	109.04	-0.10	0.40	0.00	0.47	0.99	0.98	0.01
PNICO23	4.27	0.38	0.39	0.09	0.47	0.98	0.97	0.01
CT20	0.98	0.10	0.21	0.21	0.25	0.48	0.44	0.04
CARBHB12	6.04	1.12	1.12	0.19	1.29	2.76	2.43	0.33
ADIM6	3.36	0.29	0.29	0.09	0.33	0.57	0.48	0.09
3B-69-TRIM	12.30	-0.11	0.47	0.04	0.63	1.61	1.61	0.00
ISOL24	21.92	-2.33	5.84	0.27	8.22	20.14	20.12	0.02
C60ISO	98.25	0.92	2.35	0.02	2.52	3.54	3.44	0.10
L7	18.20	0.47	1.03	0.06	1.23	1.86	1.81	0.05
UPU23	5.72	0.55	0.71	0.12	0.83	1.69	1.64	0.05
ENZYMES23	15.32	-2.79	3.83	0.25	4.57	8.51	8.51	0.00

Table S75: Statistical analysis for B97-D3(0) for all testset in our databsase. The numbers given (all in kcal/mol) are average reaction energy ($|\overline{\Delta E}|$), mean deviation (MD), mean absolute deviation (MAD), MAD normalized with respect to $|\overline{\Delta E}|$ (NMAD), root-mean-square deviation (RMSD), deviation span (Δ_{err}), maximum (max) and minimum deviation (min).

Test set	$\|\overline{\Delta E}\|$	MD	MAD	NMAD	RMSD	Δ_{err}	max	min
FH51	31.01	1.11	2.36	0.08	3.09	8.62	8.59	0.03
YBDE18	49.28	-3.17	5.19	0.11	6.34	12.13	11.57	0.56
AL2X6	35.88	-4.53	4.53	0.13	4.89	8.83	6.54	2.29
DARC	32.47	5.56	5.56	0.17	6.02	8.76	7.68	1.08
NBPRC	27.71	2.25	2.78	0.10	3.75	8.30	8.21	0.09
HEAVYSB9	58.02	-3.88	3.88	0.07	4.23	9.08	6.83	2.25
BSR36	16.20	-6.89	6.89	0.43	8.10	21.05	18.66	2.39
RSE43	7.60	-1.82	1.82	0.24	2.08	5.34	4.97	0.37
W4-11	306.91	-2.59	3.47	0.01	4.88	26.16	26.15	0.01
G21EA	33.62	-3.95	4.26	0.13	5.17	10.36	10.23	0.13
G21IP	257.61	0.41	2.80	0.01	3.51	8.13	8.12	0.01
DIPCS10	654.26	-1.87	3.24	0.00	4.24	9.15	8.61	0.54
PA26	189.05	2.99	3.05	0.02	3.85	10.05	9.81	0.24
SIE4x4	33.72	16.85	16.85	0.50	19.18	40.13	37.40	2.73
ALKBDE10	100.69	-1.48	3.90	0.04	5.05	9.60	9.43	0.17
RC21	35.70	2.01	2.74	0.08	3.26	6.39	6.27	0.12
ALK8	62.60	-0.80	1.66	0.03	1.99	3.41	3.35	0.06
DC13	54.98	1.39	9.29	0.17	11.93	26.90	26.30	0.60
G2RC	51.26	0.13	3.34	0.07	4.14	9.02	8.90	0.12
BH76RC	21.39	-0.15	1.58	0.07	2.14	5.35	5.34	0.01
MOR23	35.57	6.98	8.07	0.23	14.71	64.29	63.92	0.37
WCPT18	34.99	-2.49	2.52	0.07	2.98	6.63	6.41	0.22
BHROT27	6.37	0.24	0.34	0.05	0.47	1.15	1.14	0.01
BHPERI	20.87	-0.23	1.19	0.06	1.63	5.60	5.60	0.00

Continued on next page

Test set	$\|\overline{\Delta E}\|$	MD	MAD	NMAD	RMSD	Δ_{err}	max	min
BHDIV10	45.33	-2.25	3.22	0.07	3.59	6.23	5.83	0.40
INV24	32.85	-1.11	1.34	0.04	1.62	3.79	3.75	0.04
CR20	19.31	-6.14	6.14	0.32	6.24	12.44	8.40	4.04
CRBH20	46.13	-6.33	6.33	0.14	6.43	13.45	8.62	4.83
TMBH17	12.76	2.14	3.19	0.25	4.22	10.21	10.12	0.09
LTMBH26	9.98	-1.90	3.29	0.33	4.64	15.83	15.67	0.16
BH76	18.61	-4.38	4.58	0.25	5.15	10.71	10.62	0.09
ISO34	14.57	-0.42	1.37	0.09	2.03	6.72	6.70	0.02
ICONF	3.27	0.13	0.47	0.14	0.65	1.57	1.56	0.01
ACONF	1.83	0.30	0.30	0.16	0.34	0.81	0.72	0.09
TAUT15	3.05	-0.38	0.96	0.31	1.14	1.91	1.83	0.08
Amino20x4	2.44	-0.12	0.32	0.13	0.41	1.17	1.16	0.01
PCONF	1.62	-0.22	1.02	0.63	1.09	2.37	2.00	0.37
MCONF	4.97	-0.37	0.39	0.08	0.45	0.96	0.95	0.01
SCONF	4.60	-0.42	0.52	0.11	0.63	1.17	1.15	0.02
PArel	4.63	0.10	1.03	0.22	1.56	4.93	4.89	0.04
BUT14DIOL	2.80	-0.13	0.26	0.09	0.30	0.65	0.64	0.01
EIE22	5.44	1.14	1.18	0.22	1.31	2.75	2.55	0.20
Styrene45	62.64	1.63	3.03	0.05	3.86	9.36	9.36	0.00
ISOMERIZATION20	31.84	0.35	1.93	0.06	2.50	5.49	5.46	0.03
DIE60	4.71	1.15	1.16	0.25	1.28	2.43	2.39	0.04
IDISP	14.22	3.15	7.20	0.51	8.61	16.10	15.53	0.57
C20C24	30.77	-5.72	30.62	1.00	35.21	74.04	59.77	14.27
S66	5.47	-0.51	0.53	0.10	0.68	2.16	2.16	0.00
S10x8	6.59	0.27	0.39	0.06	0.67	3.73	3.73	0.00
X40	3.76	0.49	0.54	0.14	0.67	2.07	2.04	0.03
HEAVY28	1.24	-0.05	0.20	0.16	0.26	0.68	0.67	0.01
CHB6	26.79	1.38	1.38	0.05	1.59	3.52	2.92	0.60
AHB21	22.49	-0.64	0.89	0.04	1.13	2.47	2.46	0.01
IL16	109.04	0.79	0.84	0.01	0.99	1.72	1.63	0.09
PNICO23	4.27	-0.39	0.42	0.10	0.47	0.93	0.88	0.05
CT20	0.98	0.07	0.15	0.15	0.19	0.39	0.39	0.00
CARBHB12	6.04	0.89	0.89	0.15	1.06	2.34	2.07	0.27
ADIM6	3.36	0.12	0.18	0.05	0.18	0.35	0.23	0.12
3B-69-TRIM	12.30	0.77	0.80	0.06	1.04	3.78	3.77	0.01
ISOL24	21.92	-2.67	5.17	0.24	7.68	24.31	24.23	0.08
C60ISO	98.25	-1.08	2.24	0.02	2.94	6.26	6.08	0.18
L7	18.20	5.29	5.83	0.32	7.10	14.13	12.26	1.87
UPU23	5.72	0.44	0.65	0.11	0.77	1.56	1.54	0.02
ENZYMES23	15.32	-2.76	4.24	0.28	5.05	13.05	12.50	0.55

Table S76: Statistical analysis for WB97X-D3(0) for all testset in our databsase. The numbers given (all in kcal/mol) are average reaction energy ($|\overline{\Delta E}|$), mean deviation (MD), mean absolute deviation (MAD), MAD normalized with respect to $|\overline{\Delta E}|$ (NMAD), root-mean-square deviation (RMSD), deviation span (Δ_{err}), maximum (max) and minimum deviation (min).

Test set	$\lvert\overline{\Delta E}\rvert$	MD	MAD	NMAD	RMSD	Δ_{err}	max	min
FH51	31.01	-1.74	2.67	0.09	3.66	13.53	13.49	0.04
YBDE18	49.28	39.21	39.21	0.80	52.33	78.23	75.38	2.85
AL2X6	35.88	4.11	4.11	0.11	4.33	8.80	6.45	2.35
DARC	32.47	-11.37	11.37	0.35	11.43	23.92	13.54	10.38
NBPRC	27.71	-3.11	5.72	0.21	6.11	14.64	11.14	3.50
HEAVYSB9	58.02	-0.77	2.52	0.04	2.88	4.27	4.06	0.21
BSR36	16.20	-2.56	2.61	0.16	4.03	13.58	13.56	0.02
RSE43	7.60	-0.19	1.57	0.21	4.67	30.00	29.84	0.16
W4-11	306.91	-3.78	3.95	0.01	6.41	37.84	37.81	0.03
G21EA	33.62	-3.97	4.21	0.13	5.17	10.21	9.83	0.38
G21IP	257.61	0.22	2.94	0.01	3.86	10.58	10.51	0.07
DIPCS10	654.26	-3.74	5.26	0.01	7.15	17.44	16.72	0.72
PA26	189.05	2.00	2.07	0.01	2.78	6.45	6.40	0.05
SIE4x4	33.72	11.28	11.28	0.33	13.45	32.16	29.99	2.17
ALKBDE10	100.69	-2.49	4.67	0.05	6.45	14.14	14.00	0.14
RC21	35.70	3.22	3.58	0.10	4.11	5.92	5.87	0.05
ALK8	62.60	-0.56	2.35	0.04	2.66	5.69	4.50	1.19
DC13	54.98	-2.60	7.34	0.13	9.36	20.18	19.93	0.25
G2RC	51.26	-2.47	4.39	0.09	5.20	10.30	10.06	0.24
BH76RC	21.39	-0.13	1.84	0.09	2.25	4.80	4.80	0.00
MOR23	35.57	3.10	6.25	0.18	14.35	66.34	66.02	0.32
WCPT18	34.99	-0.35	2.28	0.07	2.79	7.41	7.18	0.23
BHROT27	6.37	0.25	0.37	0.06	0.49	1.13	1.11	0.02
BHPERI	20.87	2.26	2.49	0.12	3.10	9.72	9.55	0.17
BHDIV10	45.33	1.15	1.27	0.03	1.67	3.46	3.40	0.06
INV24	32.85	-0.08	1.30	0.04	2.09	7.11	7.11	0.00
CR20	19.31	7.53	7.53	0.39	7.55	15.02	8.63	6.39
CRBH20	46.13	5.19	5.19	0.11	5.28	10.81	6.83	3.98
TMBH17	12.76	4.63	5.07	0.40	6.32	12.50	12.28	0.22
LTMBH26	9.98	0.72	2.13	0.21	3.78	17.13	17.07	0.06
BH76	18.61	-1.08	2.38	0.13	2.79	6.15	6.08	0.07
ISO34	14.57	-0.50	0.87	0.06	1.23	3.10	3.03	0.07
ICONF	3.27	0.22	0.45	0.14	0.62	1.33	1.30	0.03
ACONF	1.83	-0.43	0.43	0.24	0.49	1.02	0.89	0.13
TAUT15	3.05	-0.56	1.29	0.42	1.46	2.60	2.51	0.09
Amino20x4	2.44	0.01	0.33	0.13	0.41	1.07	1.06	0.01
PCONF	1.62	0.43	1.76	1.08	2.09	3.99	3.70	0.29
MCONF	4.97	1.45	1.47	0.30	1.63	2.43	2.32	0.11

Continued on next page

Test set	$\|\overline{\Delta E}\|$	MD	MAD	NMAD	RMSD	Δ_{err}	max	min
SCONF	4.60	-0.04	0.43	0.09	0.67	2.08	2.05	0.03
PArel	4.63	0.05	0.69	0.15	0.89	1.93	1.89	0.04
BUT14DIOL	2.80	0.50	0.50	0.18	0.55	0.93	0.93	0.00
EIE22	5.44	-0.06	0.28	0.05	0.38	0.87	0.87	0.00
Styrene45	62.64	-0.09	2.06	0.03	2.83	8.93	8.93	0.00
ISOMERIZATION20	31.84	1.72	2.81	0.09	6.04	25.79	25.69	0.10
DIE60	4.71	-0.05	0.72	0.15	0.93	2.20	2.19	0.01
IDISP	14.22	0.06	3.79	0.27	5.28	9.57	9.54	0.03
C20C24	30.77	-2.09	8.23	0.27	10.36	24.58	21.64	2.94
S66	5.47	-1.97	1.97	0.36	2.10	4.27	3.73	0.54
S10x8	6.59	-1.29	1.29	0.20	1.52	2.87	2.87	0.00
X40	3.76	-1.18	1.18	0.31	1.28	2.95	2.74	0.21
HEAVY28	1.24	1.81	1.81	1.46	1.88	3.29	2.62	0.67
CHB6	26.79	1.43	1.43	0.05	1.68	3.10	2.95	0.15
AHB21	22.49	-2.10	2.10	0.09	2.27	5.02	4.06	0.96
IL16	109.04	-1.47	1.47	0.01	1.57	3.26	2.51	0.75
PNICO23	4.27	0.93	1.01	0.24	1.04	1.71	1.48	0.23
CT20	0.98	-0.34	0.34	0.35	0.40	0.94	0.93	0.01
CARBHB12	6.04	1.79	1.79	0.30	1.86	3.40	2.55	0.85
ADIM6	3.36	3.48	3.48	1.04	3.76	7.06	5.57	1.49
3B-69-TRIM	12.30	-3.58	3.58	0.29	3.75	7.13	6.02	1.11
ISOL24	21.92	-0.36	2.80	0.13	3.93	12.70	12.51	0.19
C60ISO	98.25	15.60	15.60	0.16	17.13	30.59	25.34	5.25
L7	18.20	-6.76	6.76	0.37	7.23	12.74	10.28	2.46
UPU23	5.72	-0.02	1.26	0.22	1.71	5.82	5.78	0.04
ENZYMES23	15.32	-0.99	2.28	0.15	2.90	8.71	8.71	0.00

Table S77: Statistical analysis for PBE0-D3(BJ) for all testset in our databsase. The numbers given (all in kcal/mol) are average reaction energy ($|\overline{\Delta E}|$), mean deviation (MD), mean absolute deviation (MAD), MAD normalized with respect to $|\overline{\Delta E}|$ (NMAD), root-mean-square deviation (RMSD), deviation span (Δ_{err}), maximum (max) and minimum deviation (min).

Test set	$\|\overline{\Delta E}\|$	MD	MAD	NMAD	RMSD	Δ_{err}	max	min
FH51	31.01	2.86	2.86	0.09	3.76	9.73	9.64	0.09
YBDE18	49.28	-1.20	1.29	0.03	1.69	4.14	4.10	0.04
AL2X6	35.88	0.50	1.43	0.04	1.62	2.38	2.33	0.05
DARC	32.47	-4.13	4.13	0.13	4.94	10.72	9.08	1.64
NBPRC	27.71	-0.46	2.84	0.10	3.11	5.21	4.66	0.55
HEAVYSB9	58.02	-1.86	1.86	0.03	2.17	3.81	3.50	0.31
BSR36	16.20	-3.30	3.30	0.20	3.83	9.96	8.78	1.18
RSE43	7.60	-1.23	1.23	0.16	1.48	3.88	3.80	0.08
W4-11	306.91	-2.31	3.60	0.01	5.24	27.12	27.09	0.03

Continued on next page

Test set	$\|\overline{\Delta E}\|$	MD	MAD	NMAD	RMSD	Δ_{err}	max	min
G21EA	33.62	-3.98	4.61	0.14	6.00	12.01	12.01	0.00
G21IP	257.61	0.61	3.60	0.01	4.29	8.92	8.85	0.07
DIPCS10	654.26	0.03	2.80	0.00	3.04	5.15	4.68	0.47
PA26	189.05	1.57	1.81	0.01	2.58	7.39	7.37	0.02
SIE4x4	33.72	13.79	13.79	0.41	15.81	33.62	31.79	1.83
ALKBDE10	100.69	42.82	42.82	0.43	49.02	92.40	74.95	17.45
RC21	35.70	4.76	5.31	0.15	6.16	12.50	12.48	0.02
ALK8	62.60	4.83	4.85	0.08	6.18	9.69	9.57	0.12
DC13	54.98	-1.95	7.80	0.14	10.19	22.20	21.72	0.48
G2RC	51.26	-3.12	6.55	0.13	8.27	21.84	21.64	0.20
BH76RC	21.39	-0.37	2.09	0.10	2.80	7.06	6.85	0.21
MOR23	35.57	1.13	5.53	0.16	13.24	60.48	60.46	0.02
WCPT18	34.99	4.49	4.49	0.13	5.19	10.92	9.97	0.95
BHROT27	6.37	0.55	0.55	0.09	0.74	1.45	1.45	0.00
BHPERI	20.87	3.22	3.22	0.15	3.52	6.58	5.93	0.65
BHDIV10	45.33	4.62	4.62	0.10	5.15	7.84	7.70	0.14
INV24	32.85	1.19	1.19	0.04	1.71	4.57	4.54	0.03
CR20	19.31	1.45	1.54	0.08	1.80	3.15	3.06	0.09
CRBH20	46.13	0.74	0.97	0.02	1.18	2.32	2.32	0.00
TMBH17	12.76	1.17	3.37	0.26	4.19	6.97	6.85	0.12
LTMBH26	9.98	-1.42	2.82	0.28	3.99	14.27	14.26	0.01
BH76	18.61	-11.11	11.40	0.61	18.54	60.92	60.91	0.01
ISO34	14.57	1.39	1.39	0.10	1.78	3.87	3.83	0.04
ICONF	3.27	0.32	0.32	0.10	0.48	1.36	1.32	0.04
ACONF	1.83	0.03	0.03	0.02	0.05	0.13	0.13	0.00
TAUT15	3.05	1.15	1.15	0.38	1.49	3.26	3.12	0.14
Amino20x4	2.44	0.29	0.29	0.12	0.36	0.93	0.93	0.00
PCONF	1.62	0.86	0.86	0.53	1.02	1.86	1.81	0.05
MCONF	4.97	0.25	0.25	0.05	0.30	0.60	0.60	0.00
SCONF	4.60	0.39	0.39	0.08	0.53	1.54	1.53	0.01
PArel	4.63	0.59	1.21	0.26	1.68	5.13	5.02	0.11
BUT14DIOL	2.80	0.29	0.30	0.11	0.34	0.92	0.84	0.08
EIE22	5.44	1.19	1.23	0.23	1.39	2.71	2.56	0.15
Styrene45	62.64	0.93	2.69	0.04	3.29	9.19	9.19	0.00
ISOMERIZATION20	31.84	0.12	1.93	0.06	2.51	5.75	5.69	0.06
DIE60	4.71	1.33	1.34	0.29	1.54	2.86	2.85	0.01
IDISP	14.22	1.61	1.61	0.11	2.08	3.75	3.52	0.23
C20C24	30.77	-10.83	10.83	0.35	14.35	28.78	26.24	2.54
S66	5.47	-0.24	0.24	0.04	0.36	1.32	1.32	0.00
S10x8	6.59	-0.36	0.40	0.06	0.63	2.43	2.43	0.00
X40	3.76	0.25	0.25	0.07	0.43	1.89	1.89	0.00
HEAVY28	1.24	0.36	0.36	0.29	0.46	1.35	1.34	0.01
CHB6	26.79	0.01	0.60	0.02	0.81	1.41	1.36	0.05
AHB21	22.49	-2.02	2.02	0.09	2.49	5.04	4.99	0.05

Continued on next page

Test set	$\lvert\overline{\Delta E}\rvert$	MD	MAD	NMAD	RMSD	Δ_{err}	max	min
IL16	109.04	-0.79	0.81	0.01	1.04	2.17	2.12	0.05
PNICO23	4.27	0.88	0.88	0.21	1.19	3.81	3.78	0.03
CT20	0.98	0.03	0.14	0.14	0.18	0.43	0.43	0.00
CARBHB12	6.04	1.66	1.66	0.28	1.91	4.07	3.52	0.55
ADIM6	3.36	0.22	0.22	0.07	0.22	0.47	0.28	0.19
3B-69-TRIM	12.30	-0.10	0.67	0.05	0.89	2.48	2.44	0.04
ISOL24	21.92	0.23	1.97	0.09	2.78	7.68	7.59	0.09
C60ISO	98.25	2.38	2.94	0.03	3.15	5.98	4.78	1.20
L7	18.20	2.42	2.73	0.15	3.38	6.32	6.01	0.31
UPU23	5.72	0.47	0.60	0.10	0.71	1.63	1.58	0.05
ENZYMES23	15.32	-0.99	2.28	0.15	2.90	8.71	8.71	0.00

Table S78: Statistical analysis for TPSSH-D3(BJ) for all testset in our databsase. The numbers given (all in kcal/mol) are average reaction energy ($\lvert\overline{\Delta E}\rvert$), mean deviation (MD), mean absolute deviation (MAD), MAD normalized with respect to $\lvert\overline{\Delta E}\rvert$ (NMAD), root-mean-square deviation (RMSD), deviation span (Δ_{err}), maximum (max) and minimum deviation (min).

Test set	$\lvert\overline{\Delta E}\rvert$	MD	MAD	NMAD	RMSD	Δ_{err}	max	min
FH51	31.01	3.26	3.26	0.11	4.44	12.32	12.29	0.03
YBDE18	49.28	-5.00	5.02	0.10	5.40	8.28	8.06	0.22
AL2X6	35.88	0.83	2.24	0.06	2.36	4.86	3.53	1.33
DARC	32.47	3.00	3.30	0.10	3.74	5.52	5.12	0.40
NBPRC	27.71	-0.46	1.55	0.06	2.01	4.63	4.61	0.02
HEAVYSB9	58.02	-1.63	2.29	0.04	2.70	5.31	4.99	0.32
BSR36	16.20	-4.13	4.13	0.25	4.60	11.85	10.48	1.37
RSE43	7.60	-1.33	1.33	0.17	1.61	4.71	4.50	0.21
W4-11	306.91	-2.19	4.89	0.02	6.60	26.97	26.92	0.05
G21EA	33.62	-4.39	4.88	0.15	6.38	12.69	12.61	0.08
G21IP	257.61	-0.08	3.70	0.01	4.45	11.40	11.05	0.35
DIPCS10	654.26	-1.49	2.96	0.00	3.73	8.72	7.93	0.79
PA26	189.05	3.62	3.62	0.02	4.37	11.32	10.81	0.51
SIE4x4	33.72	17.79	17.79	0.53	20.56	43.29	40.67	2.62
ALKBDE10	100.69	-2.81	5.78	0.06	7.11	14.24	13.83	0.41
RC21	35.70	3.01	3.78	0.11	4.13	8.71	7.27	1.44
ALK8	62.60	4.33	4.33	0.07	5.58	10.23	9.33	0.90
DC13	54.98	-0.08	8.45	0.15	10.89	24.01	23.09	0.92
G2RC	51.26	1.49	5.76	0.11	7.24	16.49	16.49	0.00
BH76RC	21.39	-0.26	3.06	0.14	4.09	9.16	9.15	0.01
MOR23	35.57	-0.29	6.57	0.18	13.55	60.10	59.86	0.24
WCPT18	34.99	4.65	4.65	0.13	5.12	10.07	8.79	1.28
BHROT27	6.37	0.53	0.53	0.08	0.70	1.53	1.51	0.02
BHPERI	20.87	3.96	3.96	0.19	4.20	8.26	6.71	1.55

Continued on next page

Test set	$\|\overline{\Delta E}\|$	MD	MAD	NMAD	RMSD	Δ_{err}	max	min
BHDIV10	45.33	5.25	5.25	0.12	5.68	11.25	9.33	1.92
INV24	32.85	1.22	1.22	0.04	1.56	4.09	3.87	0.22
CR20	19.31	-1.97	2.19	0.11	2.41	4.53	3.88	0.65
CRBH20	46.13	-6.63	6.63	0.14	6.67	13.38	8.11	5.27
TMBH17	12.76	-0.41	3.47	0.27	4.31	8.04	8.03	0.01
LTMBH26	9.98	-2.82	4.01	0.40	5.14	15.47	15.47	0.00
BH76	18.61	-7.39	7.44	0.40	8.22	17.29	17.21	0.08
ISO34	14.57	1.99	1.99	0.14	2.74	9.75	9.66	0.09
ICONF	3.27	0.18	0.18	0.05	0.25	0.65	0.64	0.01
ACONF	1.83	0.08	0.08	0.04	0.09	0.18	0.18	0.00
TAUT15	3.05	1.36	1.36	0.45	1.57	2.75	2.73	0.02
Amino20x4	2.44	0.30	0.30	0.12	0.36	0.87	0.86	0.01
PCONF	1.62	0.92	0.92	0.57	1.10	2.15	1.95	0.20
MCONF	4.97	0.36	0.36	0.07	0.44	0.86	0.85	0.01
SCONF	4.60	0.83	0.83	0.18	1.05	2.71	2.65	0.06
PArel	4.63	0.21	1.32	0.28	1.78	4.80	4.67	0.13
BUT14DIOL	2.80	0.19	0.21	0.07	0.26	0.76	0.74	0.02
EIE22	5.44	1.64	1.65	0.30	1.82	3.30	3.20	0.10
Styrene45	62.64	1.07	2.37	0.04	3.05	8.53	8.53	0.00
ISOMERIZATION20	31.84	1.17	2.88	0.09	3.70	8.48	8.48	0.00
DIE60	4.71	1.59	1.59	0.34	1.71	3.05	2.85	0.20
IDISP	14.22	2.17	2.17	0.15	3.31	7.66	7.50	0.16
C20C24	30.77	-11.41	20.02	0.65	28.04	54.87	53.03	1.84
S66	5.47	-0.29	0.29	0.05	0.35	0.99	0.99	0.00
S10x8	6.59	-0.12	0.26	0.04	0.36	1.44	1.44	0.00
X40	3.76	0.28	0.28	0.07	0.39	1.50	1.49	0.01
HEAVY28	1.24	0.36	0.37	0.30	0.47	1.31	1.30	0.01
CHB6	26.79	0.66	0.82	0.03	1.06	2.25	2.20	0.05
AHB21	22.49	-1.21	1.29	0.06	1.59	3.14	3.02	0.12
IL16	109.04	-0.27	0.43	0.00	0.55	1.36	1.32	0.04
PNICO23	4.27	0.88	0.95	0.22	1.22	3.55	3.53	0.02
CT20	0.98	0.17	0.23	0.23	0.27	0.54	0.53	0.01
CARBHB12	6.04	1.40	1.40	0.23	1.67	3.66	3.21	0.45
ADIM6	3.36	0.06	0.09	0.03	0.10	0.18	0.15	0.03
3B-69-TRIM	12.30	0.44	0.69	0.06	0.93	2.75	2.74	0.01
ISOL24	21.92	-2.31	4.41	0.20	5.95	15.02	14.83	0.19
C60ISO	98.25	-3.18	3.88	0.04	5.15	10.95	10.52	0.43
L7	18.20	1.52	1.85	0.10	2.03	4.60	3.56	1.04
UPU23	5.72	0.40	0.53	0.09	0.66	1.38	1.37	0.01
ENZYMES23	15.32	-3.49	4.51	0.29	5.51	10.25	10.02	0.23

Table S79: Statistical analysis for PW6B95-D3(0) for all testset in our databsase. The numbers given (all in kcal/mol) are average reaction energy ($|\overline{\Delta E}|$), mean deviation (MD), mean absolute deviation (MAD), MAD normalized with respect to $|\overline{\Delta E}|$ (NMAD), root-mean-square deviation (RMSD), deviation span (Δ_{err}), maximum (max) and minimum deviation (min).

Test set	$\lvert\overline{\Delta E}\rvert$	MD	MAD	NMAD	RMSD	Δ_{err}	max	min
FH51	31.01	0.41	1.64	0.05	2.21	6.79	6.72	0.07
YBDE18	49.28	34.27	34.48	0.70	48.16	69.94	69.61	0.33
AL2X6	35.88	4.62	4.62	0.13	4.77	10.60	6.87	3.73
DARC	32.47	-4.52	4.52	0.14	4.91	10.11	7.62	2.49
NBPRC	27.71	-2.76	3.96	0.14	4.32	8.68	8.04	0.64
HEAVYSB9	58.02	-2.77	2.77	0.05	3.11	7.18	6.03	1.15
BSR36	16.20	-3.38	3.38	0.21	3.86	9.93	8.89	1.04
RSE43	7.60	-1.80	1.80	0.24	2.09	5.27	4.83	0.44
W4-11	306.91	-2.36	2.92	0.01	4.97	31.55	31.52	0.03
G21EA	33.62	-3.88	4.15	0.12	5.12	10.85	10.21	0.64
G21IP	257.61	1.38	2.81	0.01	3.65	9.23	9.17	0.06
DIPCS10	654.26	1.34	2.79	0.00	3.27	5.33	5.24	0.09
PA26	189.05	1.49	1.74	0.01	2.51	7.36	7.36	0.00
SIE4x4	33.72	14.86	14.86	0.44	17.19	35.97	34.70	1.27
ALKBDE10	100.69	-2.28	4.15	0.04	5.32	12.36	11.58	0.78
RC21	35.70	2.22	2.73	0.08	3.25	6.89	6.79	0.10
ALK8	62.60	2.89	3.08	0.05	4.25	7.79	7.64	0.15
DC13	54.98	-0.07	8.08	0.15	10.06	18.53	17.87	0.66
G2RC	51.26	-0.66	2.92	0.06	3.57	7.52	7.47	0.05
BH76RC	21.39	-0.18	1.52	0.07	2.03	4.97	4.86	0.11
MOR23	35.57	4.50	5.29	0.15	13.80	64.64	64.57	0.07
WCPT18	34.99	-1.66	1.71	0.05	2.06	4.57	4.56	0.01
BHROT27	6.37	0.46	0.52	0.08	0.70	1.46	1.45	0.01
BHPERI	20.87	-0.12	0.96	0.05	1.20	2.80	2.80	0.00
BHDIV10	45.33	-2.04	2.30	0.05	2.50	4.74	3.86	0.88
INV24	32.85	1.28	1.28	0.04	1.64	3.70	3.63	0.07
CR20	19.31	2.16	2.16	0.11	2.36	4.22	4.13	0.09
CRBH20	46.13	-0.51	0.67	0.01	0.82	1.64	1.55	0.09
TMBH17	12.76	2.04	2.76	0.22	4.15	10.25	10.18	0.07
LTMBH26	9.98	-0.96	2.49	0.25	4.02	15.67	15.66	0.01
BH76	18.61	-3.74	3.90	0.21	4.37	9.32	9.10	0.22
ISO34	14.57	-0.64	1.26	0.09	1.58	4.22	4.15	0.07
ICONF	3.27	0.12	0.30	0.09	0.46	1.23	1.20	0.03
ACONF	1.83	-0.14	0.14	0.08	0.18	0.38	0.37	0.01
TAUT15	3.05	0.06	0.83	0.27	1.04	2.16	2.13	0.03
Amino20x4	2.44	0.05	0.28	0.12	0.40	1.17	1.17	0.00
PCONF	1.62	0.42	0.52	0.32	0.67	1.39	1.38	0.01
MCONF	4.97	0.22	0.37	0.07	0.45	1.08	1.06	0.02

Continued on next page

Test set	$\|\overline{\Delta E}\|$	MD	MAD	NMAD	RMSD	Δ_{err}	max	min
SCONF	4.60	0.09	0.33	0.07	0.46	1.42	1.41	0.01
PArel	4.63	0.45	0.92	0.20	1.44	4.97	4.93	0.04
BUT14DIOL	2.80	-0.27	0.27	0.10	0.30	0.63	0.59	0.04
EIE22	5.44	1.09	1.11	0.20	1.24	2.28	2.25	0.03
Styrene45	62.64	0.49	2.12	0.03	2.53	6.96	6.96	0.00
ISOMERIZATION20	31.84	2.26	4.27	0.13	9.74	41.95	41.82	0.13
DIE60	4.71	1.17	1.17	0.25	1.32	2.44	2.42	0.02
IDISP	14.22	2.12	3.51	0.25	4.92	11.34	10.61	0.73
C20C24	30.77	-12.58	12.58	0.41	15.45	32.93	28.46	4.47
S66	5.47	-0.15	0.27	0.05	0.35	1.00	0.99	0.01
S10x8	6.59	1.78	1.84	0.28	10.68	83.71	83.71	0.00
X40	3.76	0.15	0.25	0.07	0.32	0.88	0.88	0.00
HEAVY28	1.24	0.77	0.78	0.63	0.83	1.54	1.40	0.14
CHB6	26.79	-0.18	0.65	0.02	0.91	2.10	2.00	0.10
AHB21	22.49	-0.95	1.03	0.05	1.40	3.48	3.46	0.02
IL16	109.04	0.17	0.37	0.00	0.45	0.95	0.93	0.02
PNICO23	4.27	-0.10	0.16	0.04	0.24	0.88	0.87	0.01
CT20	0.98	0.01	0.09	0.09	0.11	0.23	0.23	0.00
CARBHB12	6.04	0.65	0.65	0.11	0.76	1.78	1.58	0.20
ADIM6	3.36	0.66	0.66	0.20	0.70	1.39	1.00	0.39
3B-69-TRIM	12.30	0.08	2.45	0.20	9.76	73.81	73.80	0.01
ISOL24	21.92	-1.91	3.76	0.17	4.82	11.30	10.89	0.41
C60ISO	98.25	1.63	2.35	0.02	2.47	4.73	3.60	1.13
L7	18.20	3.87	3.87	0.21	4.45	7.74	6.71	1.03
UPU23	5.72	0.40	0.56	0.10	0.66	1.23	1.15	0.08
ENZYMES23	15.32	-2.66	2.67	0.17	2.99	3.78	3.75	0.03

Table S80: Statistical analysis for mPWB1K-D3(BJ) for all testset in our databsase. The numbers given (all in kcal/mol) are average reaction energy ($|\overline{\Delta E}|$), mean deviation (MD), mean absolute deviation (MAD), MAD normalized with respect to $|\overline{\Delta E}|$ (NMAD), root-mean-square deviation (RMSD), deviation span (Δ_{err}), maximum (max) and minimum deviation (min).

Test set	$\|\overline{\Delta E}\|$	MD	MAD	NMAD	RMSD	Δ_{err}	max	min
FH51	31.01	-2.01	2.54	0.08	3.21	7.42	7.38	0.04
YBDE18	49.28	23.20	23.36	0.47	30.44	46.90	45.43	1.47
AL2X6	35.88	6.30	6.30	0.18	6.56	14.31	9.73	4.58
DARC	32.47	-5.93	5.93	0.18	6.14	12.88	8.57	4.31
NBPRC	27.71	-3.57	5.49	0.20	6.28	14.90	13.28	1.62
HEAVYSB9	58.02	-0.74	0.90	0.02	1.10	2.39	2.12	0.27
BSR36	16.20	-0.59	0.67	0.04	0.74	1.77	1.63	0.14
RSE43	7.60	-1.14	1.14	0.15	1.40	3.53	3.51	0.02
W4-11	306.91	-7.77	8.05	0.03	10.66	37.81	37.77	0.04

Continued on next page

Test set	$\|\overline{\Delta E}\|$	MD	MAD	NMAD	RMSD	Δ_{err}	max	min
G21EA	33.62	-6.47	6.47	0.19	7.84	14.66	14.65	0.01
G21IP	257.61	-0.43	2.89	0.01	3.72	9.19	9.12	0.07
DIPCS10	654.26	-2.21	3.46	0.01	4.53	9.03	9.03	0.00
PA26	189.05	1.63	2.05	0.01	2.69	7.00	6.95	0.05
SIE4x4	33.72	9.26	9.26	0.27	11.11	25.41	24.77	0.64
ALKBDE10	100.69	46.14	46.14	0.46	55.04	108.17	99.58	8.59
RC21	35.70	4.21	4.58	0.13	5.54	14.04	13.71	0.33
ALK8	62.60	6.14	6.14	0.10	8.14	15.00	14.66	0.34
DC13	54.98	-3.62	9.35	0.17	11.61	24.14	24.09	0.05
G2RC	51.26	-3.99	5.60	0.11	7.20	18.83	18.64	0.19
BH76RC	21.39	-0.12	2.67	0.13	3.01	6.68	6.21	0.47
MOR23	35.57	1.19	6.02	0.17	14.07	64.66	64.62	0.04
WCPT18	34.99	-0.38	1.31	0.04	1.69	4.21	4.20	0.01
BHROT27	6.37	0.65	0.68	0.11	0.93	1.95	1.91	0.04
BHPERI	20.87	-0.29	2.14	0.10	2.57	7.06	6.97	0.09
BHDIV10	45.33	-1.13	1.62	0.04	1.91	4.05	3.54	0.51
INV24	32.85	0.66	2.03	0.06	3.11	10.22	10.12	0.10
CR20	19.31	5.53	5.53	0.29	5.59	10.41	6.73	3.68
CRBH20	46.13	5.47	5.47	0.12	5.53	10.85	6.72	4.13
TMBH17	12.76	3.22	4.55	0.36	5.65	11.59	11.41	0.18
LTMBH26	9.98	0.32	1.85	0.19	3.43	15.50	15.49	0.01
BH76	18.61	-1.21	1.89	0.10	2.25	6.03	5.94	0.09
ISO34	14.57	-0.28	1.43	0.10	1.92	4.16	4.14	0.02
ICONF	3.27	0.20	0.57	0.18	0.70	1.61	1.49	0.12
ACONF	1.83	-0.40	0.40	0.22	0.46	0.99	0.89	0.10
TAUT15	3.05	0.39	0.74	0.24	1.01	2.11	2.09	0.02
Amino20x4	2.44	0.12	0.48	0.20	0.63	2.03	2.02	0.01
PCONF	1.62	0.70	1.15	0.71	1.44	2.97	2.88	0.09
MCONF	4.97	-0.81	0.88	0.18	1.03	1.97	1.94	0.03
SCONF	4.60	0.17	0.48	0.10	0.60	1.68	1.68	0.00
PArel	4.63	0.86	1.12	0.24	1.57	4.29	4.27	0.02
BUT14DIOL	2.80	-0.12	0.15	0.06	0.22	0.76	0.76	0.00
EIE22	5.44	0.72	0.76	0.14	0.89	1.72	1.70	0.02
Styrene45	62.64	0.59	3.45	0.06	4.45	11.82	11.82	0.00
ISOMERIZATION20	31.84	1.36	2.92	0.09	6.57	28.03	28.02	0.01
DIE60	4.71	0.97	1.01	0.21	1.26	2.90	2.83	0.07
IDISP	14.22	-0.24	3.27	0.23	4.17	7.53	7.48	0.05
C20C24	30.77	-12.97	13.27	0.43	16.43	27.00	26.11	0.89
S66	5.47	0.37	1.02	0.19	2.35	11.64	11.63	0.01
S10x8	6.59	0.41	1.22	0.18	5.30	42.10	42.09	0.01
X40	3.76	-0.13	0.26	0.07	0.37	1.21	1.21	0.00
HEAVY28	1.24	0.58	0.60	0.48	0.66	1.41	1.27	0.14
CHB6	26.79	-0.72	0.97	0.04	1.41	3.35	3.18	0.17
AHB21	22.49	-1.50	1.54	0.07	2.14	6.03	5.86	0.17

Continued on next page

Test set	$\lvert\overline{\Delta E}\rvert$	MD	MAD	NMAD	RMSD	Δ_{err}	max	min
IL16	109.04	-0.21	0.35	0.00	0.45	0.96	0.95	0.01
PNICO23	4.27	0.54	0.54	0.13	0.66	1.46	1.41	0.05
CT20	0.98	-3.35	3.61	3.68	10.48	34.80	34.78	0.02
CARBHB12	6.04	0.86	0.86	0.14	0.96	2.06	1.78	0.28
ADIM6	3.36	1.34	1.34	0.40	1.47	2.67	2.12	0.55
3B-69-TRIM	12.30	-1.09	1.21	0.10	4.45	34.98	34.98	0.00
ISOL24	21.92	1.09	2.86	0.13	3.99	11.97	11.89	0.08
C60ISO	98.25	8.99	8.99	0.09	9.45	19.09	14.42	4.67
L7	18.20	-1.97	1.97	0.11	2.38	3.32	3.21	0.11
UPU23	5.72	0.24	0.69	0.12	0.91	2.48	2.47	0.01
ENZYMES23	15.32	0.58	1.81	0.12	2.91	10.59	10.42	0.17

Table S81: Statistical analysis for mPW1B95-D3(BJ) for all testset in our databsase. The numbers given (all in kcal/mol) are average reaction energy ($\lvert\overline{\Delta E}\rvert$), mean deviation (MD), mean absolute deviation (MAD), MAD normalized with respect to $\lvert\overline{\Delta E}\rvert$ (NMAD), root-mean-square deviation (RMSD), deviation span (Δ_{err}), maximum (max) and minimum deviation (min).

Test set	$\lvert\overline{\Delta E}\rvert$	MD	MAD	NMAD	RMSD	Δ_{err}	max	min
FH51	31.01	-0.30	1.51	0.05	2.16	6.25	6.21	0.04
YBDE18	49.28	32.74	32.74	0.66	44.72	65.53	64.78	0.75
AL2X6	35.88	6.47	6.47	0.18	6.69	14.06	9.44	4.62
DARC	32.47	-8.27	8.27	0.25	8.55	17.61	11.69	5.92
NBPRC	27.71	-3.24	4.78	0.17	5.45	13.16	11.21	1.95
HEAVYSB9	58.02	-0.35	0.96	0.02	1.12	2.42	2.29	0.13
BSR36	16.20	-1.92	1.92	0.12	2.09	4.56	4.41	0.15
RSE43	7.60	-1.89	1.89	0.25	2.19	5.30	4.99	0.31
W4-11	306.91	-1.40	2.60	0.01	4.24	26.95	26.95	0.00
G21EA	33.62	-5.65	5.67	0.17	6.68	12.87	12.59	0.28
G21IP	257.61	-0.47	2.67	0.01	3.26	9.01	8.86	0.15
DIPCS10	654.26	-2.71	2.95	0.00	3.87	7.75	7.42	0.33
PA26	189.05	1.02	1.56	0.01	2.21	6.46	6.43	0.03
SIE4x4	33.72	13.70	13.70	0.41	15.89	33.56	32.44	1.12
ALKBDE10	100.69	42.84	42.84	0.43	48.67	92.30	74.71	17.59
RC21	35.70	3.23	3.67	0.10	4.31	8.36	8.22	0.14
ALK8	62.60	5.76	5.90	0.09	8.24	15.41	15.18	0.23
DC13	54.98	-1.49	7.56	0.14	9.56	22.44	22.05	0.39
G2RC	51.26	-1.72	4.12	0.08	5.15	12.59	12.50	0.09
BH76RC	21.39	-0.18	1.76	0.08	2.22	4.93	4.85	0.08
MOR23	35.57	2.05	5.01	0.14	13.63	64.01	63.94	0.07
WCPT18	34.99	-2.20	2.25	0.06	2.55	5.77	5.35	0.42
BHROT27	6.37	0.52	0.58	0.09	0.78	1.47	1.47	0.00
BHPERI	20.87	-0.69	1.64	0.08	1.86	3.80	3.59	0.21

Continued on next page

Test set	$\lvert\overline{\Delta E}\rvert$	MD	MAD	NMAD	RMSD	Δ_{err}	max	min
BHDIV10	45.33	-2.68	2.87	0.06	3.12	5.71	4.77	0.94
INV24	32.85	-0.49	1.39	0.04	1.85	5.17	4.93	0.24
CR20	19.31	4.85	4.85	0.25	4.93	9.36	6.33	3.03
CRBH20	46.13	2.91	2.91	0.06	2.97	6.08	4.12	1.96
TMBH17	12.76	1.99	3.44	0.27	4.31	8.43	8.39	0.04
LTMBH26	9.98	-0.59	2.07	0.21	3.58	14.85	14.85	0.00
BH76	18.61	-3.27	3.47	0.19	3.90	10.18	9.98	0.20
ISO34	14.57	-0.59	1.35	0.09	1.68	3.97	3.91	0.06
ICONF	3.27	0.14	0.37	0.11	0.49	1.28	1.20	0.08
ACONF	1.83	-0.15	0.15	0.08	0.18	0.37	0.37	0.00
TAUT15	3.05	0.39	0.89	0.29	1.24	2.76	2.75	0.01
Amino20x4	2.44	0.07	0.36	0.15	0.47	1.51	1.50	0.01
PCONF	1.62	0.46	0.60	0.37	0.71	1.49	1.45	0.04
MCONF	4.97	0.30	0.43	0.09	0.53	1.07	1.07	0.00
SCONF	4.60	0.14	0.36	0.08	0.45	1.24	1.22	0.02
PArel	4.63	0.66	1.00	0.22	1.55	4.87	4.86	0.01
BUT14DIOL	2.80	-0.21	0.21	0.08	0.24	0.52	0.52	0.00
EIE22	5.44	1.07	1.10	0.20	1.25	2.37	2.26	0.11
Styrene45	62.64	-0.18	2.55	0.04	3.31	9.28	9.28	0.00
ISOMERIZATION20	31.84	2.29	4.17	0.13	9.78	42.31	42.15	0.16
DIE60	4.71	1.25	1.27	0.27	1.47	2.89	2.83	0.06
IDISP	14.22	0.93	1.55	0.11	1.90	3.06	2.98	0.08
C20C24	30.77	-14.41	14.41	0.47	15.43	28.27	22.69	5.58
S66	5.47	-0.37	0.37	0.07	0.45	1.01	1.00	0.01
S10x8	6.59	0.14	0.42	0.06	1.07	8.75	8.75	0.00
X40	3.76	0.28	0.31	0.08	0.37	0.81	0.79	0.02
HEAVY28	1.24	0.86	0.88	0.71	0.97	1.77	1.59	0.18
CHB6	26.79	-0.50	0.73	0.03	1.14	2.59	2.59	0.00
AHB21	22.49	-0.97	1.14	0.05	1.58	4.06	4.03	0.03
IL16	109.04	0.47	0.63	0.01	0.73	1.33	1.19	0.14
PNICO23	4.27	0.21	0.32	0.08	0.47	1.69	1.65	0.04
CT20	0.98	0.25	0.26	0.26	0.29	0.51	0.48	0.03
CARBHB12	6.04	0.60	0.60	0.10	0.75	1.80	1.64	0.16
ADIM6	3.36	0.15	0.15	0.04	0.19	0.43	0.39	0.04
3B-69-TRIM	12.30	-2.15	4.71	0.38	16.27	84.88	84.70	0.18
ISOL24	21.92	-0.76	2.28	0.10	2.93	6.92	6.78	0.14
C60ISO	98.25	3.14	3.14	0.03	3.56	5.84	5.61	0.23
L7	18.20	1.79	1.79	0.10	1.82	3.36	2.09	1.27
UPU23	5.72	0.48	0.62	0.11	0.72	1.43	1.34	0.09
ENZYMES23	15.32	-0.88	2.36	0.15	3.31	11.43	11.15	0.28

Table S82: Statistical analysis for mPW1PW-D3(BJ) for all testset in our databsase. The numbers given (all in kcal/mol) are average reaction energy ($|\overline{\Delta E}|$), mean deviation (MD), mean absolute deviation (MAD), MAD normalized with respect to $|\overline{\Delta E}|$ (NMAD), root-mean-square deviation (RMSD), deviation span (Δ_{err}), maximum (max) and minimum deviation (min).

Test set	$\lvert\overline{\Delta E}\rvert$	MD	MAD	NMAD	RMSD	Δ_{err}	max	min
FH51	31.01	-0.51	2.22	0.07	2.95	7.48	7.43	0.05
YBDE18	49.28	-3.59	3.59	0.07	3.82	8.09	6.69	1.40
AL2X6	35.88	-1.21	1.37	0.04	1.90	3.80	3.73	0.07
DARC	32.47	-0.85	2.38	0.07	2.83	6.75	5.91	0.84
NBPRC	27.71	-1.09	1.67	0.06	1.88	3.35	2.94	0.41
HEAVYSB9	58.02	-3.68	3.68	0.06	3.87	7.71	5.70	2.01
BSR36	16.20	-4.58	4.58	0.28	5.14	13.36	11.49	1.87
RSE43	7.60	-1.07	1.07	0.14	1.32	3.60	3.55	0.05
W4-11	306.91	-6.41	6.57	0.02	8.04	31.11	30.98	0.13
G21EA	33.62	-3.49	4.28	0.13	5.65	11.49	11.48	0.01
G21IP	257.61	1.73	3.98	0.02	4.77	10.57	10.50	0.07
DIPCS10	654.26	2.41	3.70	0.01	4.18	8.45	7.55	0.90
PA26	189.05	2.13	2.18	0.01	2.97	8.36	8.16	0.20
SIE4x4	33.72	15.78	15.78	0.47	17.54	37.17	32.28	4.89
ALKBDE10	100.69	42.27	42.27	0.42	48.58	90.22	73.76	16.46
RC21	35.70	3.38	3.76	0.11	4.34	9.58	9.03	0.55
ALK8	62.60	3.28	3.28	0.05	4.00	6.39	5.93	0.46
DC13	54.98	-0.81	7.48	0.14	9.47	17.46	17.45	0.01
G2RC	51.26	-2.28	5.11	0.10	6.47	16.79	16.77	0.02
BH76RC	21.39	-0.38	1.86	0.09	2.53	6.72	6.56	0.16
MOR23	35.57	-0.24	2.63	0.07	3.64	11.17	11.07	0.10
WCPT18	34.99	-3.15	3.15	0.09	3.83	7.86	7.81	0.05
BHROT27	6.37	0.47	0.52	0.08	0.71	1.42	1.40	0.02
BHPERI	20.87	-1.67	1.82	0.09	2.12	4.05	4.03	0.02
BHDIV10	45.33	-3.11	3.81	0.08	4.23	6.47	6.36	0.11
INV24	32.85	-0.44	1.17	0.04	1.69	4.78	4.73	0.05
CR20	19.31	-1.70	1.72	0.09	2.05	3.88	3.70	0.18
CRBH20	46.13	-1.15	1.20	0.03	1.52	3.18	3.16	0.02
TMBH17	12.76	1.62	3.15	0.25	4.06	7.78	7.59	0.19
LTMBH26	9.98	-1.52	2.86	0.29	4.09	14.88	14.75	0.13
BH76	18.61	-3.92	4.21	0.23	4.58	11.55	11.18	0.37
ISO34	14.57	-0.23	1.23	0.08	1.68	5.30	5.29	0.01
ICONF	3.27	0.22	0.31	0.10	0.46	1.28	1.27	0.01
ACONF	1.83	0.18	0.18	0.10	0.20	0.44	0.38	0.06
TAUT15	3.05	0.21	1.12	0.37	1.34	2.69	2.54	0.15
Amino20x4	2.44	0.01	0.26	0.11	0.34	0.99	0.99	0.00
PCONF	1.62	-0.24	1.36	0.84	1.50	2.46	2.27	0.19
MCONF	4.97	-0.38	0.39	0.08	0.45	0.87	0.83	0.04

Continued on next page

Test set	$\|\overline{\Delta E}\|$	MD	MAD	NMAD	RMSD	Δ_{err}	max	min
SCONF	4.60	0.04	0.31	0.07	0.42	1.26	1.22	0.04
PArel	4.63	0.45	1.04	0.22	1.52	4.95	4.88	0.07
BUT14DIOL	2.80	0.10	0.17	0.06	0.23	0.74	0.74	0.00
EIE22	5.44	1.22	1.25	0.23	1.41	2.78	2.63	0.15
Styrene45	62.64	1.76	2.27	0.04	2.88	7.81	7.81	0.00
ISOMERIZATION20	31.84	0.07	1.93	0.06	2.46	5.29	5.09	0.20
DIE60	4.71	1.31	1.32	0.28	1.51	2.85	2.81	0.04
IDISP	14.22	1.17	3.73	0.26	4.16	6.13	5.76	0.37
C20C24	30.77	-7.70	14.49	0.47	19.85	37.20	36.39	0.81
S66	5.47	-0.78	0.80	0.15	0.96	2.55	2.53	0.02
S10x8	6.59	0.13	0.35	0.05	0.53	2.03	2.02	0.01
X40	3.76	0.57	0.67	0.18	0.76	2.15	2.05	0.10
HEAVY28	1.24	-0.11	0.26	0.21	0.32	0.84	0.83	0.01
CHB6	26.79	0.80	0.90	0.03	1.22	2.56	2.53	0.03
AHB21	22.49	-1.37	1.49	0.07	2.00	4.44	4.38	0.06
IL16	109.04	0.27	0.67	0.01	0.79	1.60	1.56	0.04
PNICO23	4.27	0.20	0.43	0.10	0.65	2.37	2.35	0.02
CT20	0.98	0.40	0.40	0.40	0.44	0.78	0.78	0.00
CARBHB12	6.04	1.11	1.11	0.18	1.38	2.96	2.75	0.21
ADIM6	3.36	-1.03	1.03	0.31	1.14	2.13	1.76	0.37
3B-69-TRIM	12.30	1.48	1.57	0.13	1.87	4.97	4.94	0.03
ISOL24	21.92	-0.46	2.64	0.12	3.69	8.23	8.18	0.05
C60ISO	98.25	2.77	3.09	0.03	3.43	6.25	5.59	0.66
L7	18.20	5.88	5.88	0.32	6.70	13.97	11.36	2.61
UPU23	5.72	0.89	0.95	0.17	1.19	2.53	2.47	0.06
ENZYMES23	15.32	-1.33	2.77	0.18	3.64	10.92	10.82	0.10

Table S83: Statistical analysis for PWB6K-D3(0) for all testset in our databsase. The numbers given (all in kcal/mol) are average reaction energy ($|\overline{\Delta E}|$), mean deviation (MD), mean absolute deviation (MAD), MAD normalized with respect to $|\overline{\Delta E}|$ (NMAD), root-mean-square deviation (RMSD), deviation span (Δ_{err}), maximum (max) and minimum deviation (min).

Test set	$\|\overline{\Delta E}\|$	MD	MAD	NMAD	RMSD	Δ_{err}	max	min
FH51	31.01	-1.64	2.24	0.07	2.83	6.64	6.61	0.03
YBDE18	49.28	-2.60	2.60	0.05	2.92	6.17	5.56	0.61
AL2X6	35.88	0.58	0.84	0.02	0.85	1.75	1.11	0.64
DARC	32.47	-5.89	5.89	0.18	5.98	12.49	8.18	4.31
NBPRC	27.71	-1.90	2.34	0.08	2.57	5.54	4.23	1.31
HEAVYSB9	58.02	-2.86	2.86	0.05	2.95	5.65	4.10	1.55
BSR36	16.20	-0.28	0.43	0.03	0.50	1.02	0.98	0.04
RSE43	7.60	-1.01	1.01	0.13	1.25	3.19	3.18	0.01
W4-11	306.91	-10.70	10.74	0.04	13.28	40.52	40.48	0.04

Continued on next page

Test set	$\lvert\overline{\Delta E}\rvert$	MD	MAD	NMAD	RMSD	Δ_{err}	max	min
G21EA	33.62	-5.76	5.89	0.18	7.32	15.48	14.26	1.22
G21IP	257.61	0.73	2.91	0.01	3.96	10.79	10.67	0.12
DIPCS10	654.26	0.23	3.47	0.01	4.46	9.29	9.19	0.10
PA26	189.05	1.24	1.80	0.01	2.46	6.49	6.41	0.08
SIE4x4	33.72	9.77	9.77	0.29	11.37	26.09	23.01	3.08
ALKBDE10	100.69	45.34	45.34	0.45	54.52	106.99	99.76	7.23
RC21	35.70	3.06	3.34	0.09	4.15	11.55	10.96	0.59
ALK8	62.60	4.98	4.98	0.08	6.14	9.57	9.29	0.28
DC13	54.98	-3.56	8.81	0.16	10.99	21.94	21.80	0.14
G2RC	51.26	-3.85	5.09	0.10	6.22	14.71	14.04	0.67
BH76RC	21.39	-0.32	2.82	0.13	3.20	7.74	7.00	0.74
MOR23	35.57	-2.49	4.21	0.12	5.17	9.11	9.07	0.04
WCPT18	34.99	0.88	1.21	0.03	1.69	5.55	5.35	0.20
BHROT27	6.37	0.65	0.68	0.11	0.94	1.92	1.90	0.02
BHPERI	20.87	0.01	2.10	0.10	2.52	7.38	7.12	0.26
BHDIV10	45.33	-0.40	1.33	0.03	1.50	3.13	2.72	0.41
INV24	32.85	0.37	1.98	0.06	3.22	10.91	10.89	0.02
CR20	19.31	-1.83	1.89	0.10	2.28	4.70	4.51	0.19
CRBH20	46.13	1.55	1.74	0.04	1.97	3.77	3.41	0.36
TMBH17	12.76	4.20	4.71	0.37	6.24	12.92	12.51	0.41
LTMBH26	9.98	0.81	1.95	0.20	3.76	16.15	16.07	0.08
BH76	18.61	-0.93	1.73	0.09	2.16	6.15	6.09	0.06
ISO34	14.57	-0.36	1.39	0.10	1.73	3.57	3.56	0.01
ICONF	3.27	0.23	0.43	0.13	0.63	1.59	1.50	0.09
ACONF	1.83	-0.04	0.05	0.03	0.08	0.27	0.27	0.00
TAUT15	3.05	0.25	0.68	0.22	0.90	2.11	2.06	0.05
Amino20x4	2.44	0.10	0.35	0.14	0.49	1.28	1.27	0.01
PCONF	1.62	0.61	0.62	0.38	0.72	1.35	1.31	0.04
MCONF	4.97	0.05	0.27	0.05	0.32	0.71	0.70	0.01
SCONF	4.60	-0.02	0.12	0.03	0.19	0.63	0.62	0.01
PArel	4.63	0.81	0.99	0.21	1.50	4.40	4.37	0.03
BUT14DIOL	2.80	-0.27	0.27	0.10	0.29	0.67	0.58	0.09
EIE22	5.44	0.74	0.76	0.14	0.88	1.73	1.70	0.03
Styrene45	62.64	0.75	2.70	0.04	3.47	9.42	9.42	0.00
ISOMERIZATION20	31.84	-0.38	1.80	0.06	2.17	4.28	4.27	0.01
DIE60	4.71	0.95	0.99	0.21	1.23	2.76	2.75	0.01
IDISP	14.22	0.60	2.99	0.21	3.76	7.53	7.18	0.35
C20C24	30.77	-10.04	12.55	0.41	18.03	34.57	34.51	0.06
S66	5.47	-0.06	0.23	0.04	0.32	1.13	1.13	0.00
S10x8	6.59	-0.11	0.28	0.04	0.54	3.23	3.22	0.01
X40	3.76	0.03	0.27	0.07	0.37	1.04	1.02	0.02
HEAVY28	1.24	0.05	0.13	0.10	0.17	0.55	0.54	0.01
CHB6	26.79	-1.23	1.27	0.05	1.75	3.66	3.56	0.10
AHB21	22.49	-1.57	1.61	0.07	2.24	6.44	6.31	0.13

Continued on next page

Test set	$\overline{\lvert\Delta E\rvert}$	MD	MAD	NMAD	RMSD	Δ_{err}	max	min
IL16	109.04	0.31	0.47	0.00	0.53	0.93	0.89	0.04
PNICO23	4.27	0.04	0.23	0.05	0.33	1.00	1.00	0.00
CT20	0.98	1.44	1.75	1.78	6.33	25.30	25.30	0.00
CARBHB12	6.04	0.75	0.75	0.12	0.85	1.97	1.67	0.30
ADIM6	3.36	2.20	2.20	0.65	2.34	4.36	3.27	1.09
3B-69-TRIM	12.30	0.40	2.11	0.17	8.69	49.42	49.42	0.00
ISOL24	21.92	-0.36	2.31	0.11	2.93	6.83	6.61	0.22
C60ISO	98.25	9.69	9.69	0.10	10.30	20.66	16.02	4.64
L7	18.20	11.86	11.86	0.65	17.93	35.72	34.56	1.16
UPU23	5.72	0.58	0.64	0.11	0.80	1.61	1.55	0.06
ENZYMES23	15.32	0.47	2.38	0.16	3.78	12.47	12.42	0.05

Table S84: Statistical analysis for SOGGA11-X-D3(BJ) for all testset in our databsase. The numbers given (all in kcal/mol) are average reaction energy ($\overline{\lvert\Delta E\rvert}$), mean deviation (MD), mean absolute deviation (MAD), MAD normalized with respect to $\overline{\lvert\Delta E\rvert}$ (NMAD), root-mean-square deviation (RMSD), deviation span (Δ_{err}), maximum (max) and minimum deviation (min).

Test set	$\overline{\lvert\Delta E\rvert}$	MD	MAD	NMAD	RMSD	Δ_{err}	max	min
FH51	31.01	-1.19	2.25	0.07	2.91	8.10	8.09	0.01
YBDE18	49.28	3.40	5.36	0.11	6.55	11.32	10.54	0.78
AL2X6	35.88	-1.00	1.00	0.03	1.07	2.33	1.67	0.66
DARC	32.47	-4.20	4.20	0.13	4.53	10.75	7.92	2.83
NBPRC	27.71	0.10	2.22	0.08	2.72	5.44	5.29	0.15
HEAVYSB9	58.02	-1.54	1.55	0.03	1.79	3.45	3.40	0.05
BSR36	16.20	-4.51	4.51	0.28	5.31	14.62	12.87	1.75
RSE43	7.60	-1.07	1.07	0.14	1.27	2.97	2.82	0.15
W4-11	306.91	-5.15	5.35	0.02	7.47	32.19	32.18	0.01
G21EA	33.62	-2.77	3.32	0.10	4.77	10.65	10.63	0.02
G21IP	257.61	2.39	3.28	0.01	4.26	9.40	9.22	0.18
DIPCS10	654.26	1.92	4.81	0.01	5.71	9.87	9.82	0.05
PA26	189.05	2.82	2.91	0.02	3.79	9.56	9.49	0.07
SIE4x4	33.72	9.93	9.93	0.29	11.71	25.76	25.12	0.64
ALKBDE10	100.69	-6.81	6.81	0.07	8.31	20.24	19.05	1.19
RC21	35.70	3.54	3.88	0.11	4.66	9.98	9.69	0.29
ALK8	62.60	3.08	3.08	0.05	3.79	7.49	7.00	0.49
DC13	54.98	-1.88	6.81	0.12	8.45	20.01	19.70	0.31
G2RC	51.26	-2.89	4.66	0.09	6.30	17.42	17.42	0.00
BH76RC	21.39	0.16	2.44	0.11	2.78	5.32	5.12	0.20
MOR23	35.57	4.41	6.61	0.19	14.45	66.21	65.78	0.43
WCPT18	34.99	0.25	1.37	0.04	1.71	4.18	3.99	0.19
BHROT27	6.37	0.35	0.38	0.06	0.50	1.25	1.23	0.02
BHPERI	20.87	0.77	1.09	0.05	1.58	4.97	4.97	0.00

Continued on next page

Test set	$\lvert\overline{\Delta E}\rvert$	MD	MAD	NMAD	RMSD	Δ_{err}	max	min
BHDIV10	45.33	-0.56	1.22	0.03	1.71	3.30	3.30	0.00
INV24	32.85	0.73	1.61	0.05	2.85	9.96	9.82	0.14
CR20	19.31	0.83	0.95	0.05	1.12	2.24	2.21	0.03
CRBH20	46.13	0.46	0.92	0.02	1.15	2.50	2.37	0.13
TMBH17	12.76	3.51	4.07	0.32	5.24	9.68	9.62	0.06
LTMBH26	9.98	-0.33	2.30	0.23	3.87	16.89	16.70	0.19
BH76	18.61	-1.75	2.09	0.11	2.50	6.18	6.18	0.00
ISO34	14.57	-0.34	1.16	0.08	1.52	4.14	4.03	0.11
ICONF	3.27	0.18	0.31	0.09	0.43	1.12	1.08	0.04
ACONF	1.83	-0.17	0.17	0.09	0.20	0.50	0.45	0.05
TAUT15	3.05	0.51	0.51	0.17	0.75	1.87	1.85	0.02
Amino20x4	2.44	-0.03	0.23	0.09	0.28	0.78	0.78	0.00
PCONF	1.62	0.50	0.50	0.31	0.68	1.55	1.55	0.00
MCONF	4.97	0.16	0.25	0.05	0.29	0.64	0.63	0.01
SCONF	4.60	0.10	0.51	0.11	0.70	1.98	1.97	0.01
PArel	4.63	0.53	0.81	0.18	1.18	3.35	3.34	0.01
BUT14DIOL	2.80	0.43	0.43	0.15	0.45	0.86	0.84	0.02
EIE22	5.44	0.81	0.82	0.15	0.94	1.87	1.80	0.07
Styrene45	62.64	1.79	2.78	0.04	3.22	7.34	7.34	0.00
ISOMERIZATION20	31.84	0.21	1.68	0.05	1.96	3.70	3.66	0.04
DIE60	4.71	0.91	0.95	0.20	1.19	2.63	2.63	0.00
IDISP	14.22	1.21	2.60	0.18	2.85	4.49	4.15	0.34
C20C24	30.77	-8.52	10.37	0.34	13.92	25.66	24.50	1.16
S66	5.47	-0.31	0.42	0.08	0.47	1.03	0.99	0.04
S10x8	6.59	0.08	0.43	0.06	0.55	1.47	1.47	0.00
X40	3.76	0.47	0.52	0.14	0.59	1.13	1.12	0.01
HEAVY28	1.24	-0.04	0.23	0.19	0.31	0.85	0.84	0.01
CHB6	26.79	0.55	0.97	0.04	1.16	2.69	2.27	0.42
AHB21	22.49	-1.17	1.22	0.05	1.70	4.53	4.49	0.04
IL16	109.04	0.26	0.41	0.00	0.49	1.00	0.98	0.02
PNICO23	4.27	0.09	0.22	0.05	0.26	0.54	0.52	0.02
CT20	0.98	0.79	0.79	0.80	0.85	1.54	1.38	0.16
CARBHB12	6.04	0.78	0.79	0.13	0.95	1.97	1.94	0.03
ADIM6	3.36	-0.75	0.75	0.22	0.76	1.38	0.87	0.51
3B-69-TRIM	12.30	1.33	1.43	0.12	1.69	3.33	3.31	0.02
ISOL24	21.92	-0.12	1.47	0.07	1.92	4.35	4.32	0.03
C60ISO	98.25	7.89	7.89	0.08	8.34	17.52	13.20	4.32
L7	18.20	2.23	2.23	0.12	2.61	4.97	4.88	0.09
UPU23	5.72	0.86	0.93	0.16	1.09	2.18	2.14	0.04
ENZYMES23	15.32	0.13	2.11	0.14	3.49	12.21	12.20	0.01

Table S85: Statistical analysis for M05-2X-D3(0) for all testset in our databsase. The numbers given (all in kcal/mol) are average reaction energy ($|\overline{\Delta E}|$), mean deviation (MD), mean absolute deviation (MAD), MAD normalized with respect to $|\overline{\Delta E}|$ (NMAD), root-mean-square deviation (RMSD), deviation span (Δ_{err}), maximum (max) and minimum deviation (min).

Test set	$\lvert\overline{\Delta E}\rvert$	MD	MAD	NMAD	RMSD	Δ_{err}	max	min
FH51	31.01	-1.77	2.25	0.07	2.83	8.06	8.03	0.03
YBDE18	49.28	15.72	15.72	0.32	20.33	32.81	30.93	1.88
AL2X6	35.88	4.33	4.33	0.12	4.66	9.28	7.18	2.10
DARC	32.47	-6.53	6.53	0.20	6.55	13.20	7.36	5.84
NBPRC	27.71	-1.60	2.28	0.08	2.98	5.96	5.94	0.02
HEAVYSB9	58.02	-2.78	2.79	0.05	3.85	7.98	7.97	0.01
BSR36	16.20	1.34	1.49	0.09	1.80	4.51	4.50	0.01
RSE43	7.60	-0.29	0.53	0.07	0.69	2.09	2.05	0.04
W4-11	306.91	-4.87	6.70	0.02	9.63	36.00	35.60	0.40
G21EA	33.62	-2.37	3.31	0.10	4.13	8.70	8.68	0.02
G21IP	257.61	1.46	3.46	0.01	4.65	13.45	13.43	0.02
DIPCS10	654.26	0.72	3.91	0.01	4.57	10.21	9.53	0.68
PA26	189.05	-0.59	1.54	0.01	1.84	4.62	4.36	0.26
SIE4x4	33.72	6.37	6.37	0.19	6.60	12.92	8.99	3.93
ALKBDE10	100.69	-2.09	5.10	0.05	7.44	18.32	18.15	0.17
RC21	35.70	2.72	3.03	0.08	3.51	5.89	5.81	0.08
ALK8	62.60	2.30	2.92	0.05	4.30	9.51	9.24	0.27
DC13	54.98	-2.58	9.48	0.17	12.25	27.37	26.88	0.49
G2RC	51.26	-3.84	4.48	0.09	5.44	11.07	10.32	0.75
BH76RC	21.39	-0.96	1.51	0.07	2.05	5.40	5.32	0.08
MOR23	35.57	1.41	7.66	0.22	15.11	66.03	65.62	0.41
WCPT18	34.99	-2.15	3.16	0.09	3.96	6.62	6.55	0.07
BHROT27	6.37	0.36	0.42	0.07	0.55	1.20	1.18	0.02
BHPERI	20.87	-2.58	2.82	0.14	3.36	6.05	5.67	0.38
BHDIV10	45.33	-0.44	1.54	0.03	1.79	3.15	3.12	0.03
INV24	32.85	0.38	1.67	0.05	2.53	7.18	7.17	0.01
CR20	19.31	3.24	3.24	0.17	3.35	6.12	4.72	1.40
CRBH20	46.13	3.76	3.76	0.08	3.86	6.94	5.00	1.94
TMBH17	12.76	3.41	5.04	0.40	6.37	13.57	12.96	0.61
LTMBH26	9.98	0.14	1.48	0.15	2.91	13.21	13.13	0.08
BH76	18.61	-1.02	2.28	0.12	2.84	7.23	6.99	0.24
ISO34	14.57	0.16	1.13	0.08	1.57	4.99	4.98	0.01
ICONF	3.27	0.12	0.46	0.14	0.60	1.22	1.16	0.06
ACONF	1.83	-0.48	0.48	0.26	0.54	1.10	0.94	0.16
TAUT15	3.05	0.47	0.85	0.28	1.07	2.86	2.71	0.15
Amino20x4	2.44	0.13	0.45	0.18	0.56	1.34	1.34	0.00
PCONF	1.62	0.45	2.16	1.34	2.56	4.21	3.92	0.29
MCONF	4.97	1.59	1.64	0.33	1.87	2.88	2.80	0.08

Continued on next page

Test set	$\lvert\overline{\Delta E}\rvert$	MD	MAD	NMAD	RMSD	Δ_{err}	max	min
SCONF	4.60	0.22	0.70	0.15	0.87	2.12	2.10	0.02
PArel	4.63	0.23	0.99	0.21	1.45	3.66	3.64	0.02
BUT14DIOL	2.80	0.11	0.27	0.10	0.33	0.76	0.76	0.00
EIE22	5.44	-0.08	0.28	0.05	0.37	1.08	1.08	0.00
Styrene45	62.64	3.78	3.84	0.06	4.58	10.65	10.65	0.00
ISOMERIZATION20	31.84	-0.16	1.48	0.05	1.80	3.54	3.41	0.13
DIE60	4.71	0.08	0.47	0.10	0.70	2.07	2.04	0.03
IDISP	14.22	-0.99	5.76	0.41	6.41	11.93	9.73	2.20
C20C24	30.77	10.48	15.44	0.50	16.87	29.89	23.67	6.22
S66	5.47	1.79	1.79	0.33	2.00	4.93	4.61	0.32
S10x8	6.59	-1.09	1.09	0.17	1.30	3.36	3.35	0.01
X40	3.76	-0.90	0.90	0.24	1.09	3.73	3.42	0.31
HEAVY28	1.24	0.80	0.80	0.64	0.88	1.52	1.52	0.00
CHB6	26.79	-0.84	0.86	0.03	1.28	2.88	2.83	0.05
AHB21	22.49	-2.11	2.11	0.09	2.76	7.28	7.26	0.02
IL16	109.04	-0.54	0.63	0.01	0.79	1.86	1.84	0.02
PNICO23	4.27	0.87	0.87	0.20	0.95	2.41	2.05	0.36
CT20	0.98	-0.34	0.35	0.35	0.38	0.56	0.55	0.01
CARBHB12	6.04	1.17	1.17	0.19	1.30	2.12	1.82	0.30
ADIM6	3.36	3.07	3.07	0.91	3.34	6.30	5.02	1.28
3B-69-TRIM	12.30	-3.19	3.19	0.26	3.40	6.48	5.51	0.97
ISOL24	21.92	1.08	2.90	0.13	4.49	13.21	13.00	0.21
C60ISO	98.25	10.68	10.68	0.11	11.32	20.56	15.76	4.80
L7	18.20	-7.27	7.27	0.40	7.78	14.41	11.82	2.59
UPU23	5.72	-0.31	1.28	0.22	1.79	6.13	6.13	0.00
ENZYMES23	15.32	-0.45	1.62	0.11	2.20	5.90	5.87	0.03

Table S86: Statistical analysis for M06-D3(0) for all testset in our databsase. The numbers given (all in kcal/mol) are average reaction energy ($\lvert\overline{\Delta E}\rvert$), mean deviation (MD), mean absolute deviation (MAD), MAD normalized with respect to $\lvert\overline{\Delta E}\rvert$ (NMAD), root-mean-square deviation (RMSD), deviation span (Δ_{err}), maximum (max) and minimum deviation (min).

Test set	$\lvert\overline{\Delta E}\rvert$	MD	MAD	NMAD	RMSD	Δ_{err}	max	min
FH51	31.01	-0.74	1.98	0.06	2.68	5.95	5.91	0.04
YBDE18	49.28	35.24	35.31	0.72	49.18	71.15	71.08	0.07
AL2X6	35.88	5.06	5.06	0.14	5.87	13.31	10.88	2.43
DARC	32.47	-7.52	7.52	0.23	7.58	15.52	9.25	6.27
NBPRC	27.71	-2.76	3.87	0.14	4.59	10.11	9.28	0.83
HEAVYSB9	58.02	1.02	1.81	0.03	2.18	4.98	4.25	0.73
BSR36	16.20	1.57	1.72	0.11	2.05	4.95	4.90	0.05
RSE43	7.60	-1.23	1.23	0.16	1.65	6.09	6.09	0.00
W4-11	306.91	-2.14	4.48	0.01	5.99	20.98	20.89	0.09

Continued on next page

Test set	$\lvert\overline{\Delta E}\rvert$	MD	MAD	NMAD	RMSD	Δ_{err}	max	min
G21EA	33.62	-5.16	5.31	0.16	6.02	11.73	10.94	0.79
G21IP	257.61	-0.93	3.04	0.01	3.87	8.89	8.89	0.00
DIPCS10	654.26	-5.21	5.38	0.01	7.01	13.14	12.96	0.18
PA26	189.05	1.94	2.25	0.01	3.17	8.79	8.52	0.27
SIE4x4	33.72	12.33	12.33	0.37	13.62	24.68	21.55	3.13
ALKBDE10	100.69	-0.22	3.84	0.04	4.97	11.52	11.33	0.19
RC21	35.70	1.26	2.31	0.06	2.66	5.01	4.93	0.08
ALK8	62.60	1.35	3.52	0.06	4.59	11.10	10.18	0.92
DC13	54.98	-1.93	7.10	0.13	9.22	19.86	18.65	1.21
G2RC	51.26	-0.47	3.91	0.08	5.35	14.33	14.20	0.13
BH76RC	21.39	-0.73	1.64	0.08	2.23	6.07	6.07	0.00
MOR23	35.57	-0.20	3.94	0.11	5.19	13.11	13.10	0.01
WCPT18	34.99	-2.30	2.40	0.07	3.02	6.59	6.38	0.21
BHROT27	6.37	0.63	0.64	0.10	0.86	2.25	2.25	0.00
BHPERI	20.87	-0.08	1.76	0.08	2.08	4.92	4.67	0.25
BHDIV10	45.33	-1.43	1.89	0.04	2.47	4.99	4.87	0.12
INV24	32.85	-0.44	1.33	0.04	2.15	7.30	7.30	0.00
CR20	19.31	-1.82	1.82	0.09	2.16	4.74	4.46	0.28
CRBH20	46.13	-3.96	3.96	0.09	4.01	8.09	5.17	2.92
TMBH17	12.76	1.74	2.72	0.21	4.24	11.58	11.48	0.10
LTMBH26	9.98	-1.51	3.59	0.36	4.97	18.27	17.75	0.52
BH76	18.61	-2.52	3.03	0.16	3.60	10.66	10.48	0.18
ISO34	14.57	-0.57	1.45	0.10	1.90	4.54	4.52	0.02
ICONF	3.27	-0.06	0.51	0.16	0.66	1.44	1.40	0.04
ACONF	1.83	-0.73	0.73	0.40	0.83	1.59	1.40	0.19
TAUT15	3.05	-0.57	0.99	0.33	1.19	2.35	2.32	0.03
Amino20x4	2.44	-0.01	0.54	0.22	0.67	2.05	2.05	0.00
PCONF	1.62	0.56	2.78	1.72	3.18	5.42	4.92	0.50
MCONF	4.97	1.56	1.65	0.33	1.88	3.07	3.06	0.01
SCONF	4.60	-0.11	0.51	0.11	0.75	2.20	2.18	0.02
PArel	4.63	0.52	1.04	0.22	1.68	5.66	5.65	0.01
BUT14DIOL	2.80	0.42	0.46	0.16	0.53	1.01	0.99	0.02
EIE22	5.44	0.88	0.93	0.17	1.12	2.29	2.28	0.01
Styrene45	62.64	-2.51	3.56	0.06	4.76	12.16	12.16	0.00
ISOMERIZATION20	31.84	1.96	4.39	0.14	10.38	44.45	44.39	0.06
DIE60	4.71	1.07	1.11	0.24	1.40	3.28	3.27	0.01
IDISP	14.22	1.78	5.27	0.37	6.93	15.56	14.58	0.98
C20C24	30.77	-15.63	15.63	0.51	23.28	45.35	44.61	0.74
S66	5.47	1.35	1.38	0.25	1.75	4.33	4.32	0.01
S10x8	6.59	-0.49	0.51	0.08	0.79	2.77	2.77	0.00
X40	3.76	-0.54	0.63	0.17	0.92	2.73	2.71	0.02
HEAVY28	1.24	1.43	1.44	1.16	1.56	2.54	2.46	0.08
CHB6	26.79	2.09	2.09	0.08	2.33	4.45	3.49	0.96
AHB21	22.49	-1.11	1.12	0.05	1.39	3.20	3.20	0.00

Continued on next page

Test set	$\lvert\overline{\Delta E}\rvert$	MD	MAD	NMAD	RMSD	Δ_{err}	max	min
IL16	109.04	-1.64	1.64	0.02	1.84	3.60	3.50	0.10
PNICO23	4.27	0.59	0.60	0.14	0.69	1.40	1.31	0.09
CT20	0.98	0.29	0.34	0.35	0.38	0.75	0.66	0.09
CARBHB12	6.04	0.80	0.80	0.13	0.88	1.56	1.36	0.20
ADIM6	3.36	3.76	3.76	1.12	4.23	7.92	6.83	1.09
3B-69-TRIM	12.30	-1.79	1.82	0.15	2.25	5.13	5.12	0.01
ISOL24	21.92	-1.67	2.96	0.14	4.80	18.76	18.71	0.05
C60ISO	98.25	-0.32	1.91	0.02	2.34	4.96	4.79	0.17
L7	18.20	-8.24	8.24	0.45	8.99	16.75	14.30	2.45
UPU23	5.72	-0.97	1.68	0.29	2.44	8.68	8.60	0.08
ENZYMES23	15.32	-2.68	3.85	0.25	4.74	13.33	13.06	0.27

Table S87: Statistical analysis for M06-2X-D3(BJ) for all testset in our databsase. The numbers given (all in kcal/mol) are average reaction energy ($\lvert\overline{\Delta E}\rvert$), mean deviation (MD), mean absolute deviation (MAD), MAD normalized with respect to $\lvert\overline{\Delta E}\rvert$ (NMAD), root-mean-square deviation (RMSD), deviation span (Δ_{err}), maximum (max) and minimum deviation (min).

Test set	$\lvert\overline{\Delta E}\rvert$	MD	MAD	NMAD	RMSD	Δ_{err}	max	min
FH51	31.01	1.75	1.75	0.06	2.19	5.61	5.59	0.02
YBDE18	49.28	-2.05	2.23	0.05	2.70	5.49	5.20	0.29
AL2X6	35.88	2.59	2.59	0.07	2.90	5.73	4.43	1.30
DARC	32.47	-1.06	1.08	0.03	1.27	2.64	2.53	0.11
NBPRC	27.71	-1.33	1.54	0.06	1.87	4.03	3.98	0.05
HEAVYSB9	58.02	-8.40	8.40	0.14	9.48	16.10	14.69	1.41
BSR36	16.20	0.26	1.42	0.09	1.84	5.96	5.96	0.00
RSE43	7.60	-0.01	0.41	0.05	0.53	1.09	1.09	0.00
W4-11	306.91	-3.94	4.27	0.01	6.13	30.39	30.39	0.00
G21EA	33.62	-3.64	3.85	0.11	4.76	9.94	9.58	0.36
G21IP	257.61	0.47	2.70	0.01	3.71	11.01	10.83	0.18
DIPCS10	654.26	0.13	2.81	0.00	3.37	7.51	6.66	0.85
PA26	189.05	-1.02	1.62	0.01	1.80	3.50	3.37	0.13
SIE4x4	33.72	8.38	8.38	0.25	10.03	22.97	22.63	0.34
ALKBDE10	100.69	-4.75	5.54	0.05	7.83	19.06	18.65	0.41
RC21	35.70	1.67	2.06	0.06	2.60	5.94	5.91	0.03
ALK8	62.60	2.64	3.38	0.05	4.66	10.10	9.78	0.32
DC13	54.98	-4.17	8.44	0.15	11.59	28.43	27.98	0.45
G2RC	51.26	-1.46	2.15	0.04	2.69	7.20	7.07	0.13
BH76RC	21.39	-0.48	1.45	0.07	1.99	5.95	5.90	0.05
MOR23	35.57	3.84	8.26	0.23	15.20	65.43	65.38	0.05
WCPT18	34.99	-1.67	2.48	0.07	3.14	6.10	5.96	0.14
BHROT27	6.37	0.37	0.37	0.06	0.48	1.26	1.23	0.03
BHPERI	20.87	-1.18	2.18	0.10	2.46	4.54	4.36	0.18

Continued on next page

Test set	$\lvert\overline{\Delta E}\rvert$	MD	MAD	NMAD	RMSD	Δ_{err}	max	min
BHDIV10	45.33	1.50	1.50	0.03	1.74	3.00	2.88	0.12
INV24	32.85	1.38	1.38	0.04	2.23	8.18	8.07	0.11
CR20	19.31	-0.96	1.17	0.06	1.51	2.93	2.93	0.00
CRBH20	46.13	0.58	1.18	0.03	1.35	2.55	2.51	0.04
TMBH17	12.76	3.85	5.52	0.43	6.98	14.81	14.72	0.09
LTMBH26	9.98	0.92	1.71	0.17	3.12	13.72	13.70	0.02
BH76	18.61	-0.95	1.72	0.09	2.15	7.34	7.28	0.06
ISO34	14.57	1.24	1.24	0.09	1.65	4.13	4.04	0.09
ICONF	3.27	0.01	0.49	0.15	0.66	1.33	1.30	0.03
ACONF	1.83	-0.71	0.71	0.39	0.81	1.55	1.34	0.21
TAUT15	3.05	0.42	0.42	0.14	0.50	1.11	1.10	0.01
Amino20x4	2.44	0.54	0.54	0.22	0.70	1.84	1.83	0.01
PCONF	1.62	0.85	3.31	2.04	3.82	6.19	5.71	0.48
MCONF	4.97	1.80	1.90	0.38	2.15	3.16	3.14	0.02
SCONF	4.60	0.03	0.64	0.14	0.94	2.61	2.60	0.01
PArel	4.63	0.19	0.93	0.20	1.34	3.27	3.24	0.03
BUT14DIOL	2.80	0.23	0.38	0.14	0.44	0.90	0.90	0.00
EIE22	5.44	-0.15	0.24	0.04	0.31	0.77	0.77	0.00
Styrene45	62.64	0.15	2.27	0.04	2.83	8.51	8.51	0.00
ISOMERIZATION20	31.84	0.85	1.23	0.04	1.69	3.91	3.85	0.06
DIE60	4.71	0.26	0.53	0.11	0.85	2.54	2.52	0.02
IDISP	14.22	7.67	7.67	0.54	8.42	17.00	13.82	3.18
C20C24	30.77	3.58	28.29	0.92	29.50	52.21	39.65	12.56
S66	5.47	-1.90	1.90	0.35	2.25	5.74	5.64	0.10
S10x8	6.59	-1.02	1.02	0.15	1.35	4.94	4.91	0.03
X40	3.76	1.05	1.05	0.28	1.37	4.72	4.63	0.09
HEAVY28	1.24	0.51	0.52	0.42	0.63	1.42	1.40	0.02
CHB6	26.79	-0.49	0.52	0.02	0.75	1.76	1.67	0.09
AHB21	22.49	-2.00	2.00	0.09	2.48	5.94	5.90	0.04
IL16	109.04	-1.61	1.61	0.01	1.70	3.40	2.70	0.70
PNICO23	4.27	0.85	0.87	0.20	0.93	1.99	1.81	0.18
CT20	0.98	-0.21	0.22	0.23	0.26	0.40	0.39	0.01
CARBHB12	6.04	1.16	1.16	0.19	1.27	2.08	1.78	0.30
ADIM6	3.36	3.31	3.31	0.99	3.65	7.05	5.76	1.29
3B-69-TRIM	12.30	-3.14	3.14	0.26	3.47	7.80	7.07	0.73
ISOL24	21.92	-0.28	2.89	0.13	4.38	16.56	16.11	0.45
C60ISO	98.25	8.43	8.43	0.09	8.89	15.81	11.97	3.84
L7	18.20	-10.06	10.06	0.55	11.18	22.53	18.99	3.54
UPU23	5.72	-0.65	1.53	0.27	2.17	7.74	7.54	0.20
ENZYMES23	15.32	-0.81	1.46	0.10	1.96	5.10	5.03	0.07

Table S88: Statistical analysis for M08-SO-D3(0) for all testset in our databsase. The numbers given (all in kcal/mol) are average reaction energy ($|\overline{\Delta E}|$), mean deviation (MD), mean absolute deviation (MAD), MAD normalized with respect to $|\overline{\Delta E}|$ (NMAD), root-mean-square deviation (RMSD), deviation span (Δ_{err}), maximum (max) and minimum deviation (min).

Test set	$\lvert\overline{\Delta E}\rvert$	MD	MAD	NMAD	RMSD	Δ_{err}	max	min
FH51	31.01	-0.66	1.77	0.06	2.30	6.80	6.79	0.01
YBDE18	49.28	-2.95	2.97	0.06	3.44	6.83	6.57	0.26
AL2X6	35.88	2.22	2.22	0.06	2.72	5.13	4.91	0.22
DARC	32.47	-4.72	4.72	0.15	4.80	9.43	6.20	3.23
NBPRC	27.71	-2.01	2.46	0.09	2.71	5.55	4.20	1.35
HEAVYSB9	58.02	-2.00	2.55	0.04	3.41	8.38	8.09	0.29
BSR36	16.20	2.38	2.40	0.15	2.95	6.33	6.32	0.01
RSE43	7.60	-0.06	0.60	0.08	0.74	1.61	1.61	0.00
W4-11	306.91	-4.47	4.79	0.02	6.97	33.50	33.49	0.01
G21EA	33.62	-0.77	2.78	0.08	3.47	8.81	8.40	0.41
G21IP	257.61	3.38	4.05	0.02	5.37	12.88	12.87	0.01
DIPCS10	654.26	3.03	4.01	0.01	4.95	9.92	9.44	0.48
PA26	189.05	-1.38	2.01	0.01	2.20	4.74	4.17	0.57
SIE4x4	33.72	10.38	10.38	0.31	12.12	27.77	25.07	2.70
ALKBDE10	100.69	40.15	40.15	0.40	45.67	85.80	71.78	14.02
RC21	35.70	1.49	1.97	0.06	2.36	4.51	4.51	0.00
ALK8	62.60	4.61	4.61	0.07	6.10	10.13	9.99	0.14
DC13	54.98	-3.49	7.53	0.14	9.50	18.27	17.87	0.40
G2RC	51.26	-1.80	2.59	0.05	3.47	12.11	11.39	0.72
BH76RC	21.39	-0.46	1.25	0.06	1.70	4.01	4.01	0.00
MOR23	35.57	-2.26	4.88	0.14	5.85	10.65	10.21	0.44
WCPT18	34.99	-0.19	1.52	0.04	1.87	3.68	3.59	0.09
BHROT27	6.37	0.15	0.32	0.05	0.41	1.00	0.98	0.02
BHPERI	20.87	-1.82	2.91	0.14	3.43	7.05	6.93	0.12
BHDIV10	45.33	-0.78	0.94	0.02	1.43	3.45	3.37	0.08
INV24	32.85	-0.13	2.14	0.07	3.15	8.04	8.03	0.01
CR20	19.31	-2.04	2.04	0.11	2.11	3.67	2.91	0.76
CRBH20	46.13	-1.66	1.66	0.04	1.78	2.58	2.48	0.10
TMBH17	12.76	3.66	5.03	0.39	6.35	12.70	12.45	0.25
LTMBH26	9.98	0.20	2.06	0.21	3.70	15.55	15.40	0.15
BH76	18.61	-1.06	1.63	0.09	2.07	8.03	7.93	0.10
ISO34	14.57	-0.57	0.98	0.07	1.42	3.70	3.68	0.02
ICONF	3.27	-0.03	0.69	0.21	0.91	2.34	2.26	0.08
ACONF	1.83	-0.87	0.87	0.48	0.98	1.86	1.62	0.24
TAUT15	3.05	-0.15	0.60	0.20	0.79	1.90	1.88	0.02
Amino20x4	2.44	0.02	0.62	0.25	0.82	2.16	2.16	0.00
PCONF	1.62	0.80	3.11	1.92	3.65	5.98	5.71	0.27
MCONF	4.97	1.64	1.79	0.36	2.03	3.25	3.18	0.07

Continued on next page

Test set	$\lvert\overline{\Delta E}\rvert$	MD	MAD	NMAD	RMSD	Δ_{err}	max	min
SCONF	4.60	0.23	1.04	0.23	1.35	3.91	3.66	0.25
PArel	4.63	0.27	0.91	0.20	1.32	3.41	3.38	0.03
BUT14DIOL	2.80	0.17	0.36	0.13	0.43	1.01	1.00	0.01
EIE22	5.44	-0.22	0.26	0.05	0.32	0.58	0.56	0.02
Styrene45	62.64	-0.66	1.65	0.03	2.36	9.10	9.10	0.00
ISOMERIZATION20	31.84	1.46	1.58	0.05	2.10	4.86	4.77	0.09
DIE60	4.71	0.23	0.58	0.12	0.93	2.72	2.71	0.01
IDISP	14.22	-0.27	7.94	0.56	8.75	17.21	13.92	3.29
C20C24	30.77	2.92	20.25	0.66	21.19	36.61	28.01	8.60
S66	5.47	1.83	1.83	0.34	2.20	4.97	4.81	0.16
S10x8	6.59	-0.82	0.82	0.12	1.21	5.23	5.21	0.02
X40	3.76	-0.91	0.94	0.25	1.25	4.15	4.02	0.13
HEAVY28	1.24	0.68	0.68	0.55	0.77	1.43	1.43	0.00
CHB6	26.79	-0.83	0.85	0.03	1.20	2.60	2.54	0.06
AHB21	22.49	-1.21	1.21	0.05	1.45	3.22	3.20	0.02
IL16	109.04	-0.48	0.69	0.01	0.95	2.71	2.71	0.00
PNICO23	4.27	1.05	1.14	0.27	1.22	2.81	2.32	0.49
CT20	0.98	-0.07	0.14	0.15	0.19	0.45	0.45	0.00
CARBHB12	6.04	0.89	0.89	0.15	0.95	1.66	1.34	0.32
ADIM6	3.36	2.96	2.96	0.88	3.27	6.24	5.10	1.14
3B-69-TRIM	12.30	-2.67	2.67	0.22	3.02	6.55	6.19	0.36
ISOL24	21.92	0.29	2.96	0.14	4.59	15.64	15.47	0.17
C60ISO	98.25	7.85	7.85	0.08	8.29	14.76	11.16	3.60
L7	18.20	-9.39	9.39	0.52	10.43	20.62	17.62	3.00
UPU23	5.72	-0.49	1.45	0.25	2.08	7.56	7.33	0.23
ENZYMES23	15.32	-0.53	1.51	0.10	2.12	5.99	5.98	0.01

Table S89: Statistical analysis for M08-HX-D3(0) for all testset in our databsase. The numbers given (all in kcal/mol) are average reaction energy ($\lvert\overline{\Delta E}\rvert$), mean deviation (MD), mean absolute deviation (MAD), MAD normalized with respect to $\lvert\overline{\Delta E}\rvert$ (NMAD), root-mean-square deviation (RMSD), deviation span (Δ_{err}), maximum (max) and minimum deviation (min).

Test set	$\lvert\overline{\Delta E}\rvert$	MD	MAD	NMAD	RMSD	Δ_{err}	max	min
FH51	31.01	-0.99	1.95	0.06	2.45	7.79	7.76	0.03
YBDE18	49.28	16.71	16.77	0.34	21.85	34.15	33.65	0.50
AL2X6	35.88	6.80	6.80	0.19	6.93	14.54	9.24	5.30
DARC	32.47	-6.84	6.84	0.21	6.93	13.96	8.42	5.54
NBPRC	27.71	-2.40	4.25	0.15	4.70	10.02	8.58	1.44
HEAVYSB9	58.02	-2.16	2.36	0.04	3.97	10.75	10.67	0.08
BSR36	16.20	0.86	1.30	0.08	1.67	5.31	5.27	0.04
RSE43	7.60	0.19	0.57	0.08	0.70	1.48	1.48	0.00
W4-11	306.91	-3.90	4.25	0.01	6.48	36.60	36.57	0.03

Continued on next page

Test set	$\|\overline{\Delta E}\|$	MD	MAD	NMAD	RMSD	Δ_{err}	max	min
G21EA	33.62	-1.40	2.88	0.09	3.62	8.68	8.18	0.50
G21IP	257.61	3.46	3.69	0.01	4.94	14.97	14.85	0.12
DIPCS10	654.26	3.86	4.03	0.01	4.55	9.50	8.64	0.86
PA26	189.05	-0.55	0.99	0.01	1.33	3.53	3.40	0.13
SIE4x4	33.72	8.36	8.36	0.25	9.82	24.26	21.98	2.28
ALKBDE10	100.69	-4.15	4.25	0.04	6.00	15.72	15.22	0.50
RC21	35.70	1.55	1.93	0.05	2.36	5.15	5.00	0.15
ALK8	62.60	3.41	3.71	0.06	5.26	12.72	12.19	0.53
DC13	54.98	-2.37	8.65	0.16	11.12	25.29	25.11	0.18
G2RC	51.26	-2.44	3.67	0.07	4.72	14.87	14.52	0.35
BH76RC	21.39	-0.25	1.19	0.06	1.59	3.92	3.88	0.04
MOR23	35.57	0.06	6.00	0.17	7.08	13.04	13.01	0.03
WCPT18	34.99	0.53	1.85	0.05	2.09	3.67	3.59	0.08
BHROT27	6.37	0.29	0.39	0.06	0.50	1.21	1.19	0.02
BHPERI	20.87	-1.57	2.67	0.13	3.06	5.67	5.30	0.37
BHDIV10	45.33	-0.30	1.06	0.02	1.37	2.56	2.50	0.06
INV24	32.85	0.72	1.92	0.06	3.26	11.39	11.34	0.05
CR20	19.31	2.25	2.25	0.12	2.32	4.13	3.21	0.92
CRBH20	46.13	2.52	2.52	0.05	2.61	4.61	3.57	1.04
TMBH17	12.76	4.26	5.78	0.45	7.27	15.27	14.29	0.98
LTMBH26	9.98	0.77	2.00	0.20	3.47	14.10	14.07	0.03
BH76	18.61	-0.46	1.25	0.07	1.86	9.50	9.50	0.00
ISO34	14.57	0.94	0.94	0.06	1.36	3.80	3.79	0.01
ICONF	3.27	-0.07	0.71	0.22	0.91	2.24	2.18	0.06
ACONF	1.83	-0.90	0.90	0.49	1.01	1.94	1.63	0.31
TAUT15	3.05	-0.12	0.47	0.16	0.70	1.95	1.93	0.02
Amino20x4	2.44	0.05	0.57	0.23	0.78	2.31	2.31	0.00
PCONF	1.62	1.01	3.18	1.96	3.77	5.98	5.93	0.05
MCONF	4.97	1.85	1.92	0.39	2.18	3.42	3.38	0.04
SCONF	4.60	0.15	0.67	0.15	0.91	2.69	2.62	0.07
PArel	4.63	0.25	0.88	0.19	1.28	3.10	3.07	0.03
BUT14DIOL	2.80	0.01	0.28	0.10	0.37	1.01	1.01	0.00
EIE22	5.44	-0.05	0.25	0.05	0.29	0.56	0.55	0.01
Styrene45	62.64	0.72	1.87	0.03	2.32	6.41	6.41	0.00
ISOMERIZATION20	31.84	1.46	1.88	0.06	2.99	11.24	11.11	0.13
DIE60	4.71	0.37	0.60	0.13	0.93	2.59	2.58	0.01
IDISP	14.22	-0.16	7.23	0.51	8.35	17.65	14.89	2.76
C20C24	30.77	5.00	15.32	0.50	16.01	26.74	20.33	6.41
S66	5.47	1.91	1.91	0.35	2.26	5.33	5.03	0.30
S10x8	6.59	-0.91	0.91	0.14	1.27	5.14	5.13	0.01
X40	3.76	-0.81	0.82	0.22	1.13	3.92	3.87	0.05
HEAVY28	1.24	1.44	1.44	1.16	1.56	2.69	2.63	0.06
CHB6	26.79	-1.12	1.12	0.04	1.54	3.25	3.23	0.02
AHB21	22.49	-1.35	1.35	0.06	1.62	4.10	3.87	0.23

Continued on next page

Test set	$\lvert\overline{\Delta E}\rvert$	MD	MAD	NMAD	RMSD	Δ_{err}	max	min
IL16	109.04	-1.02	1.02	0.01	1.17	2.17	2.04	0.13
PNICO23	4.27	1.08	1.14	0.27	1.21	2.86	2.24	0.62
CT20	0.98	-0.06	0.10	0.10	0.14	0.45	0.43	0.02
CARBHB12	6.04	1.06	1.06	0.18	1.13	2.23	1.72	0.51
ADIM6	3.36	3.16	3.16	0.94	3.52	6.77	5.61	1.16
3B-69-TRIM	12.30	-2.63	2.63	0.21	2.92	6.43	5.78	0.65
ISOL24	21.92	0.79	2.92	0.13	4.63	15.42	15.31	0.11
C60ISO	98.25	8.91	8.91	0.09	9.44	17.37	13.37	4.00
L7	18.20	-8.87	8.87	0.49	10.09	20.22	17.52	2.70
UPU23	5.72	-0.48	1.26	0.22	1.86	6.68	6.63	0.05
ENZYMES23	15.32	0.07	1.22	0.08	1.57	4.15	4.12	0.03

Table S90: Statistical analysis for MN12-SX-D3(BJ) for all testset in our databsase. The numbers given (all in kcal/mol) are average reaction energy ($\lvert\overline{\Delta E}\rvert$), mean deviation (MD), mean absolute deviation (MAD), MAD normalized with respect to $\lvert\overline{\Delta E}\rvert$ (NMAD), root-mean-square deviation (RMSD), deviation span (Δ_{err}), maximum (max) and minimum deviation (min).

Test set	$\lvert\overline{\Delta E}\rvert$	MD	MAD	NMAD	RMSD	Δ_{err}	max	min
FH51	31.01	0.50	1.74	0.06	2.61	10.11	10.11	0.00
YBDE18	49.28	-1.11	2.55	0.05	2.81	4.87	4.79	0.08
AL2X6	35.88	0.18	0.83	0.02	1.03	1.79	1.68	0.11
DARC	32.47	0.18	1.11	0.03	1.20	1.90	1.66	0.24
NBPRC	27.71	-0.88	1.48	0.05	1.82	3.14	3.11	0.03
HEAVYSB9	58.02	26.38	26.38	0.45	27.93	56.50	44.23	12.27
BSR36	16.20	0.97	0.98	0.06	1.23	3.46	3.43	0.03
RSE43	7.60	-1.49	1.49	0.20	1.76	5.39	5.20	0.19
W4-11	306.91	-0.26	3.17	0.01	5.26	29.76	29.75	0.01
G21EA	33.62	-4.48	4.74	0.14	6.93	24.51	24.05	0.46
G21IP	257.61	0.00	3.87	0.02	4.93	10.74	10.70	0.04
DIPCS10	654.26	-6.23	6.69	0.01	9.23	20.13	19.70	0.43
PA26	189.05	-0.54	1.60	0.01	1.83	4.03	3.92	0.11
SIE4x4	33.72	8.45	8.87	0.26	10.68	20.19	19.57	0.62
ALKBDE10	100.69	46.03	46.03	0.46	52.22	103.53	84.63	18.90
RC21	35.70	1.96	2.21	0.06	2.75	6.74	6.51	0.23
ALK8	62.60	-1.12	4.96	0.08	6.91	15.97	14.97	1.00
DC13	54.98	-2.39	8.20	0.15	10.50	21.40	20.85	0.55
G2RC	51.26	-0.29	2.70	0.05	3.40	8.60	8.56	0.04
BH76RC	21.39	-0.16	1.71	0.08	2.35	6.45	6.44	0.01
MOR23	35.57	-2.39	4.41	0.12	5.66	13.61	12.72	0.89
WCPT18	34.99	-0.19	0.90	0.03	1.27	3.34	3.25	0.09
BHROT27	6.37	0.59	0.61	0.10	0.90	2.50	2.49	0.01
BHPERI	20.87	-0.34	1.60	0.08	1.97	4.35	4.30	0.05

Continued on next page

Test set	$\lvert\overline{\Delta E}\rvert$	MD	MAD	NMAD	RMSD	Δ_{err}	max	min
BHDIV10	45.33	-0.32	1.19	0.03	1.47	3.16	3.10	0.06
INV24	32.85	0.39	1.50	0.05	2.18	7.81	7.65	0.16
CR20	19.31	-1.30	1.73	0.09	2.19	4.48	4.45	0.03
CRBH20	46.13	-3.51	3.51	0.08	3.68	7.68	5.98	1.70
TMBH17	12.76	3.89	5.02	0.39	7.91	25.10	25.02	0.08
LTMBH26	9.98	1.15	4.88	0.49	14.64	73.39	73.32	0.07
BH76	18.61	-0.97	1.55	0.08	2.16	8.45	8.43	0.02
ISO34	14.57	-0.11	1.11	0.08	1.50	3.91	3.85	0.06
ICONF	3.27	-0.08	0.56	0.17	0.74	1.77	1.77	0.00
ACONF	1.83	-0.61	0.61	0.34	0.69	1.40	1.20	0.20
TAUT15	3.05	0.69	1.34	0.44	1.66	3.43	3.38	0.05
Amino20x4	2.44	0.07	0.58	0.24	0.72	1.87	1.86	0.01
PCONF	1.62	1.05	2.92	1.80	3.32	5.43	5.02	0.41
MCONF	4.97	1.41	1.55	0.31	1.75	3.01	3.00	0.01
SCONF	4.60	-0.08	0.27	0.06	0.44	1.53	1.52	0.01
PArel	4.63	1.03	1.36	0.29	2.20	6.60	6.58	0.02
BUT14DIOL	2.80	-0.13	0.19	0.07	0.26	0.74	0.74	0.00
EIE22	5.44	1.06	1.10	0.20	1.21	2.58	2.27	0.31
Styrene45	62.64	1.14	1.86	0.03	2.44	7.37	7.37	0.00
ISOMERIZATION20	31.84	0.20	3.24	0.10	4.33	11.82	11.74	0.08
DIE60	4.71	1.37	1.39	0.29	1.59	3.16	3.07	0.09
IDISP	14.22	2.04	5.39	0.38	6.36	13.14	11.38	1.76
C20C24	30.77	-10.92	16.36	0.53	24.11	47.64	46.51	1.13
S66	5.47	1.04	1.18	0.22	1.56	4.28	4.28	0.00
S10x8	6.59	-0.26	0.41	0.06	0.82	3.99	3.99	0.00
X40	3.76	-0.34	0.60	0.16	0.95	3.35	3.34	0.01
HEAVY28	1.24	0.46	0.48	0.39	0.60	1.40	1.37	0.03
CHB6	26.79	0.93	0.93	0.03	1.07	2.16	1.77	0.39
AHB21	22.49	-0.39	0.66	0.03	0.86	2.09	2.05	0.04
IL16	109.04	-0.17	0.36	0.00	0.46	1.24	1.23	0.01
PNICO23	4.27	0.78	0.78	0.18	0.88	1.71	1.57	0.14
CT20	0.98	0.41	0.42	0.42	0.45	0.75	0.69	0.06
CARBHB12	6.04	0.18	0.19	0.03	0.25	0.58	0.54	0.04
ADIM6	3.36	2.27	2.27	0.67	2.58	4.92	4.26	0.66
3B-69-TRIM	12.30	-1.04	1.25	0.10	1.59	4.10	4.10	0.00
ISOL24	21.92	-0.72	2.56	0.12	3.32	9.34	9.13	0.21
C60ISO	98.25	1.28	2.65	0.03	2.84	4.01	3.76	0.25
L7	18.20	-7.00	7.00	0.38	8.19	15.68	14.01	1.67
UPU23	5.72	-0.86	1.20	0.21	1.83	6.54	6.51	0.03
ENZYMES23	15.32	-3.51	3.96	0.26	7.61	25.38	25.12	0.26

S5.5 D4-Dispersion-Corrected Hybrids

Table S91: Statistical analysis for B3LYP-D4 for all testset in our databsase. The numbers given (all in kcal/mol) are average reaction energy ($|\overline{\Delta E}|$), mean deviation (MD), mean absolute deviation (MAD), MAD normalized with respect to $|\overline{\Delta E}|$ (NMAD), root-mean-square deviation (RMSD), deviation span (Δ_{err}), maximum (max) and minimum deviation (min).

Test set	$\lvert\overline{\Delta E}\rvert$	MD	MAD	NMAD	RMSD	Δ_{err}	max	min
FH51	31.01	2.80	4.02	0.13	5.23	16.39	16.21	0.18
YBDE18	49.28	-9.20	9.31	0.19	10.63	19.19	18.19	1.00
AL2X6	35.88	-8.92	8.92	0.25	9.49	19.12	14.01	5.11
DARC	32.47	14.82	14.82	0.46	15.17	26.29	17.98	8.31
NBPRC	27.71	2.85	3.73	0.13	5.14	11.56	11.18	0.38
HEAVYSB9	58.02	-8.99	8.99	0.15	9.37	19.52	13.78	5.74
BSR36	16.20	-10.58	10.58	0.65	12.09	30.65	27.56	3.09
RSE43	7.60	-1.74	1.74	0.23	2.02	5.03	4.75	0.28
W4-11	306.91	-4.34	5.09	0.02	7.10	28.96	28.90	0.06
G21EA	33.62	-3.81	4.40	0.13	5.16	11.21	10.52	0.69
G21IP	257.61	0.32	3.46	0.01	4.35	10.18	10.17	0.01
DIPCS10	654.26	-1.18	4.12	0.01	5.16	9.94	9.15	0.79
PA26	189.05	1.16	1.86	0.01	2.74	8.14	7.99	0.15
SIE4x4	33.72	17.08	17.08	0.51	19.97	42.24	40.51	1.73
ALKBDE10	100.69	44.50	44.50	0.44	50.79	92.94	78.45	14.49
RC21	35.70	-0.85	2.18	0.06	3.22	10.88	10.82	0.06
ALK8	62.60	-6.35	6.35	0.10	9.34	18.54	18.29	0.25
DC13	54.98	4.71	16.06	0.29	21.23	39.31	38.82	0.49
G2RC	51.26	1.17	2.60	0.05	3.46	8.06	8.02	0.04
BH76RC	21.39	-0.59	2.24	0.10	2.85	7.27	7.24	0.03
MOR23	35.57	9.62	10.98	0.31	12.78	22.36	21.65	0.71
WCPT18	34.99	-1.21	1.35	0.04	1.71	3.64	3.60	0.04
BHROT27	6.37	0.27	0.44	0.07	0.58	1.36	1.33	0.03
BHPERI	20.87	3.74	4.44	0.21	5.16	9.56	9.31	0.25
BHDIV10	45.33	-0.75	2.80	0.06	3.53	6.13	6.10	0.03
INV24	32.85	-1.70	1.88	0.06	2.18	4.75	4.32	0.43
CR20	19.31	-11.58	11.58	0.60	11.73	23.82	15.87	7.95
CRBH20	46.13	-8.83	8.83	0.19	8.92	19.09	11.68	7.41
TMBH17	12.76	1.29	9.16	0.72	14.66	39.39	39.35	0.04
LTMBH26	9.98	-0.89	2.93	0.29	4.52	17.21	16.92	0.29
BH76	18.61	-4.91	5.07	0.27	5.81	11.72	11.55	0.17
ISO34	14.57	-0.45	2.38	0.16	3.47	11.71	11.71	0.00
ICONF	3.27	0.10	0.58	0.18	0.78	1.88	1.84	0.04
ACONF	1.83	0.92	0.92	0.50	1.03	2.14	1.80	0.34
TAUT15	3.05	-0.03	1.14	0.37	1.35	2.12	2.10	0.02
Amino20x4	2.44	-0.09	0.66	0.27	0.83	2.36	2.35	0.01
PCONF	1.62	-0.40	3.80	2.35	4.34	7.23	6.99	0.24
MCONF	4.97	-2.36	2.41	0.48	2.74	4.18	4.14	0.04

Continued on next page

Test set	$\lvert\overline{\Delta E}\rvert$	MD	MAD	NMAD	RMSD	Δ_{err}	max	min
SCONF	4.60	-0.27	0.60	0.13	0.75	1.77	1.75	0.02
PArel	4.63	-0.08	1.17	0.25	1.73	5.32	5.31	0.01
BUT14DIOL	2.80	-0.09	0.33	0.12	0.40	0.86	0.85	0.01
EIE22	5.44	1.49	1.49	0.27	1.65	2.98	2.95	0.03
Styrene45	62.64	3.67	5.96	0.10	7.69	18.59	18.59	0.00
ISOMERIZATION20	31.84	0.21	2.18	0.07	2.91	7.53	7.50	0.03
DIE60	4.71	1.32	1.34	0.28	1.49	2.85	2.85	0.00
IDISP	14.22	4.13	16.38	1.15	18.96	38.49	34.77	3.72
C20C24	30.77	1.30	54.49	1.77	57.65	125.91	87.04	38.87
S66	5.47	-0.03	0.27	0.05	0.34	0.94	0.94	0.00
S10x8	6.59	-0.23	0.38	0.06	0.53	1.53	1.53	0.00
X40	3.76	2.14	2.14	0.57	2.65	8.43	8.14	0.29
HEAVY28	1.24	-1.24	1.24	1.00	1.33	2.78	2.28	0.50
CHB6	26.79	0.15	0.61	0.02	0.78	1.94	1.64	0.30
AHB21	22.49	-0.77	0.98	0.04	1.23	2.59	2.58	0.01
PNICO23	4.27	-1.87	1.87	0.44	1.99	4.91	3.78	1.13
CT20	0.98	0.07	0.16	0.16	0.20	0.41	0.41	0.00
CARBHB12	6.04	-0.22	0.56	0.09	0.69	1.30	1.22	0.08
ADIM6	3.36	-4.86	4.86	1.44	5.32	10.00	8.14	1.86
3B-69-TRIM	12.30	-0.21	0.48	0.04	0.67	2.18	2.18	0.00
ISOL24	21.92	-2.42	5.52	0.25	7.73	19.77	19.63	0.14
C60ISO	98.25	-0.73	2.30	0.02	2.85	6.22	5.81	0.41
L7	18.20	-0.12	0.45	0.02	0.70	1.67	1.67	0.00
UPU23	5.72	0.53	0.70	0.12	0.82	1.72	1.69	0.03
ENZYMES23	15.32	-3.00	4.03	0.26	4.81	9.65	9.63	0.02

Table S92: Statistical analysis for CAMB3LYP-D4 for all testset in our databsase. The numbers given (all in kcal/mol) are average reaction energy ($\lvert\overline{\Delta E}\rvert$), mean deviation (MD), mean absolute deviation (MAD), MAD normalized with respect to $\lvert\overline{\Delta E}\rvert$ (NMAD), root-mean-square deviation (RMSD), deviation span (Δ_{err}), maximum (max) and minimum deviation (min).

Test set	$\lvert\overline{\Delta E}\rvert$	MD	MAD	NMAD	RMSD	Δ_{err}	max	min
FH51	31.01	2.06	3.19	0.10	4.15	12.10	12.06	0.04
YBDE18	49.28	-7.12	7.33	0.15	8.38	16.28	14.39	1.89
AL2X6	35.88	-5.64	5.64	0.16	5.86	12.02	8.20	3.82
DARC	32.47	10.59	10.59	0.33	10.85	18.14	12.53	5.61
NBPRC	27.71	1.26	2.04	0.07	2.60	6.43	5.48	0.95
HEAVYSB9	58.02	-6.22	6.22	0.11	6.54	12.48	9.31	3.17
BSR36	16.20	-5.53	5.53	0.34	6.28	16.23	14.17	2.06
RSE43	7.60	-1.64	1.64	0.22	1.92	4.80	4.55	0.25
W4-11	306.91	-3.58	4.53	0.01	6.35	28.66	28.62	0.04
G21EA	33.62	-0.78	7.04	0.21	12.29	48.99	48.39	0.60

Continued on next page

Test set	$\lvert\overline{\Delta E}\rvert$	MD	MAD	NMAD	RMSD	Δ_{err}	max	min
G21IP	257.61	0.38	3.43	0.01	4.32	10.19	10.18	0.01
DIPCS10	654.26	-0.87	3.90	0.01	4.90	9.94	9.15	0.79
PA26	189.05	0.98	1.72	0.01	2.56	7.66	7.59	0.07
SIE4x4	33.72	17.24	17.24	0.51	20.08	42.25	40.51	1.74
ALKBDE10	100.69	-1.97	5.91	0.06	6.69	11.56	10.05	1.51
RC21	35.70	0.13	2.15	0.06	2.73	8.41	8.26	0.15
ALK8	62.60	-3.80	4.21	0.07	6.49	14.61	14.32	0.29
DC13	54.98	3.70	12.94	0.24	16.51	30.54	29.99	0.55
G2RC	51.26	0.96	2.43	0.05	3.27	8.32	8.28	0.04
BH76RC	21.39	-0.60	2.30	0.11	2.90	7.20	7.12	0.08
MOR23	35.57	6.25	8.13	0.23	15.04	64.70	64.64	0.06
WCPT18	34.99	-1.77	1.89	0.05	2.39	5.00	4.95	0.05
BHROT27	6.37	0.28	0.43	0.07	0.58	1.36	1.33	0.03
BHPERI	20.87	1.23	2.13	0.10	2.44	4.90	4.67	0.23
BHDIV10	45.33	-1.32	2.90	0.06	3.55	6.02	5.92	0.10
INV24	32.85	-1.12	1.31	0.04	1.61	3.63	3.46	0.17
CR20	19.31	-9.09	9.09	0.47	9.18	18.17	11.61	6.56
CRBH20	46.13	-8.46	8.46	0.18	8.52	17.72	10.46	7.26
TMBH17	12.76	2.82	4.14	0.32	6.04	15.68	15.65	0.03
LTMBH26	9.98	-1.37	3.14	0.31	4.64	16.61	16.46	0.15
BH76	18.61	-5.30	5.44	0.29	6.12	11.96	11.62	0.34
ISO34	14.57	-0.32	2.10	0.14	3.05	11.21	11.18	0.03
ICONF	3.27	0.03	0.38	0.12	0.55	1.49	1.49	0.00
ACONF	1.83	0.33	0.33	0.18	0.37	0.90	0.78	0.12
TAUT15	3.05	-0.04	1.12	0.37	1.33	2.21	2.18	0.03
Amino20x4	2.44	-0.04	0.32	0.13	0.42	1.38	1.37	0.01
PCONF	1.62	-0.11	1.33	0.82	1.47	2.92	2.61	0.31
MCONF	4.97	-0.65	0.83	0.17	1.21	5.45	5.26	0.19
SCONF	4.60	-0.14	0.29	0.06	0.38	1.14	1.10	0.04
PArel	4.63	-0.05	1.15	0.25	1.70	5.31	5.30	0.01
BUT14DIOL	2.80	0.29	0.30	0.11	0.35	0.85	0.81	0.04
EIE22	5.44	1.36	1.37	0.25	1.52	2.86	2.76	0.10
Styrene45	62.64	4.16	5.76	0.09	7.38	18.44	18.44	0.00
ISOMERIZATION20	31.84	0.21	2.19	0.07	2.91	7.47	7.44	0.03
DIE60	4.71	1.23	1.24	0.26	1.37	2.47	2.46	0.01
IDISP	14.22	3.36	7.69	0.54	9.37	19.30	18.29	1.01
C20C24	30.77	-1.16	43.10	1.40	46.33	99.88	71.90	27.98
S66	5.47	-0.98	1.08	0.20	1.27	3.33	3.28	0.05
S10x8	6.59	0.39	0.43	0.06	0.76	3.82	3.82	0.00
X40	3.76	0.78	0.82	0.22	0.96	2.34	2.32	0.02
HEAVY28	1.24	-0.34	0.38	0.31	0.46	1.00	0.98	0.02
CHB6	26.79	0.74	0.92	0.03	1.25	2.71	2.70	0.01
AHB21	22.49	-0.49	0.91	0.04	1.11	2.38	2.35	0.03
IL16	109.04	1.38	1.38	0.01	1.50	2.82	2.49	0.33

Continued on next page

Test set	$\lvert\overline{\Delta E}\rvert$	MD	MAD	NMAD	RMSD	Δ_{err}	max	min
PNICO23	4.27	-0.59	0.59	0.14	0.63	1.05	1.04	0.01
CT20	0.98	0.29	0.31	0.31	0.34	0.64	0.57	0.07
CARBHB12	6.04	0.53	0.54	0.09	0.75	1.68	1.67	0.01
ADIM6	3.36	-1.42	1.42	0.42	1.54	2.99	2.37	0.62
3B-69-TRIM	12.30	1.66	1.66	0.13	1.91	5.00	4.95	0.05
ISOL24	21.92	-3.56	7.09	0.32	10.41	31.72	31.67	0.05
C60ISO	98.25	-0.34	2.12	0.02	2.58	5.13	5.04	0.09
L7	18.20	5.76	5.76	0.32	6.57	13.65	11.28	2.37
UPU23	5.72	0.93	1.02	0.18	1.27	2.38	2.35	0.03
ENZYMES23	15.32	-3.08	4.43	0.29	5.20	11.56	11.25	0.31

Table S93: Statistical analysis for B97-D4 for all testset in our databsase. The numbers given (all in kcal/mol) are average reaction energy ($\lvert\overline{\Delta E}\rvert$), mean deviation (MD), mean absolute deviation (MAD), MAD normalized with respect to $\lvert\overline{\Delta E}\rvert$ (NMAD), root-mean-square deviation (RMSD), deviation span (Δ_{err}), maximum (max) and minimum deviation (min).

Test set	$\lvert\overline{\Delta E}\rvert$	MD	MAD	NMAD	RMSD	Δ_{err}	max	min
FH51	31.01	0.41	2.06	0.07	2.82	8.79	8.76	0.03
YBDE18	49.28	-0.14	4.18	0.08	4.49	7.60	6.87	0.73
AL2X6	35.88	-0.70	1.03	0.03	1.89	4.22	4.19	0.03
DARC	32.47	0.43	1.92	0.06	2.14	3.86	3.48	0.38
NBPRC	27.71	-0.70	2.01	0.07	2.06	3.93	2.70	1.23
HEAVYSB9	58.02	-0.41	1.47	0.03	1.89	4.77	4.12	0.65
BSR36	16.20	-2.04	2.04	0.13	2.32	6.32	5.44	0.88
RSE43	7.60	-1.88	1.88	0.25	2.12	5.25	4.80	0.45
W4-11	306.91	-0.87	2.64	0.01	3.91	25.38	25.37	0.01
G21EA	33.62	-3.50	3.90	0.12	4.95	10.16	10.09	0.07
G21IP	257.61	0.58	2.74	0.01	3.44	8.36	8.24	0.12
DIPCS10	654.26	-1.07	2.70	0.00	3.60	7.13	7.05	0.08
PA26	189.05	2.32	2.39	0.01	3.18	8.76	8.68	0.08
SIE4x4	33.72	16.88	16.88	0.50	19.17	40.06	37.31	2.75
ALKBDE10	100.69	-0.53	3.77	0.04	5.03	11.72	11.62	0.10
RC21	35.70	3.34	3.72	0.10	4.34	7.61	7.51	0.10
ALK8	62.60	1.86	2.02	0.03	2.46	4.89	4.47	0.42
DC13	54.98	0.24	5.27	0.10	6.91	17.77	17.15	0.62
G2RC	51.26	-0.23	4.09	0.08	5.15	13.58	13.46	0.12
BH76RC	21.39	-0.09	1.65	0.08	2.20	5.64	5.61	0.03
MOR23	35.57	0.96	5.89	0.17	13.50	60.89	60.75	0.14
WCPT18	34.99	-3.30	3.30	0.09	3.89	8.06	8.01	0.05
BHROT27	6.37	0.31	0.42	0.07	0.55	1.35	1.35	0.00
BHPERI	20.87	-3.35	3.35	0.16	3.67	6.90	6.03	0.87
BHDIV10	45.33	-3.59	4.27	0.09	4.61	9.69	7.65	2.04

Continued on next page

Test set	$\lvert\overline{\Delta E}\rvert$	MD	MAD	NMAD	RMSD	Δ_{err}	max	min
INV24	32.85	-0.68	1.03	0.03	1.34	3.28	3.25	0.03
CR20	19.31	-1.94	1.94	0.10	2.22	4.05	3.97	0.08
CRBH20	46.13	-4.77	4.77	0.10	4.86	10.32	6.75	3.57
TMBH17	12.76	1.37	2.95	0.23	4.16	8.65	8.50	0.15
LTMBH26	9.98	-2.06	3.46	0.35	4.65	14.73	14.59	0.14
BH76	18.61	-4.90	5.07	0.27	5.64	11.72	11.58	0.14
ISO34	14.57	-0.22	1.02	0.07	1.55	5.55	5.51	0.04
ICONF	3.27	-0.00	0.27	0.08	0.34	0.86	0.83	0.03
ACONF	1.83	-0.12	0.12	0.06	0.14	0.31	0.27	0.04
TAUT15	3.05	-0.05	1.02	0.33	1.17	2.00	1.89	0.11
Amino20x4	2.44	-0.06	0.21	0.08	0.27	0.98	0.98	0.00
PCONF	1.62	-0.18	0.31	0.19	0.38	0.96	0.84	0.12
MCONF	4.97	-0.01	0.20	0.04	0.25	0.57	0.57	0.00
SCONF	4.60	-0.07	0.30	0.07	0.48	1.62	1.61	0.01
PArel	4.63	0.23	0.93	0.20	1.50	4.99	4.93	0.06
BUT14DIOL	2.80	0.29	0.29	0.10	0.32	0.66	0.64	0.02
EIE22	5.44	1.19	1.22	0.22	1.37	2.79	2.62	0.17
Styrene45	62.64	2.72	3.06	0.05	3.78	9.11	9.11	0.00
ISOMERIZATION20	31.84	0.38	1.95	0.06	2.51	5.64	5.55	0.09
DIE60	4.71	1.14	1.14	0.24	1.24	2.35	2.23	0.12
IDISP	14.22	1.44	1.76	0.12	2.40	4.90	4.81	0.09
C20C24	30.77	-9.94	11.39	0.37	18.15	34.52	34.13	0.39
S66	5.47	0.04	0.17	0.03	0.22	0.59	0.59	0.00
S10x8	6.59	-0.21	0.27	0.04	0.34	0.97	0.97	0.00
X40	3.76	-0.19	0.36	0.09	0.49	1.33	1.33	0.00
HEAVY28	1.24	0.67	0.69	0.56	0.75	1.41	1.22	0.19
CHB6	26.79	0.03	0.41	0.02	0.57	1.23	1.17	0.06
AHB21	22.49	-0.83	1.13	0.05	1.40	3.02	2.91	0.11
IL16	109.04	0.02	0.52	0.00	0.64	1.17	1.14	0.03
PNICO23	4.27	0.94	0.95	0.22	1.16	3.10	3.06	0.04
CT20	0.98	-0.09	0.12	0.12	0.17	0.54	0.54	0.00
CARBHB12	6.04	1.24	1.24	0.21	1.44	3.27	2.76	0.51
ADIM6	3.36	0.26	0.26	0.08	0.28	0.42	0.34	0.08
3B-69-TRIM	12.30	-0.40	0.48	0.04	0.64	1.99	1.98	0.01
ISOL24	21.92	-0.69	3.14	0.14	4.37	10.45	10.35	0.10
C60ISO	98.25	-1.87	2.86	0.03	3.65	8.50	7.62	0.88
L7	18.20	2.20	2.43	0.13	3.24	6.05	5.73	0.32
UPU23	5.72	0.38	0.58	0.10	0.69	1.58	1.51	0.07
ENZYMES23	15.32	-2.53	3.99	0.26	4.73	11.52	10.47	1.05

Table S94: Statistical analysis for WB97X-D4 for all testset in our databsase. The numbers given (all in kcal/mol) are average reaction energy ($|\overline{\Delta E}|$), mean deviation (MD), mean absolute deviation (MAD), MAD normalized with respect to $|\overline{\Delta E}|$ (NMAD), root-mean-square deviation (RMSD), deviation span (Δ_{err}), maximum (max) and minimum deviation (min).

Test set	$\lvert\overline{\Delta E}\rvert$	MD	MAD	NMAD	RMSD	Δ_{err}	max	min
FH51	31.01	-1.03	2.32	0.07	3.05	10.17	10.13	0.04
YBDE18	49.28	-2.77	2.79	0.06	3.13	6.17	6.06	0.11
AL2X6	35.88	-3.18	3.18	0.09	3.43	6.53	4.98	1.55
DARC	32.47	-0.64	1.62	0.05	2.16	4.88	4.42	0.46
NBPRC	27.71	-1.14	1.80	0.06	2.08	3.92	3.62	0.30
HEAVYSB9	58.02	-2.50	3.48	0.06	3.68	7.17	5.47	1.70
BSR36	16.20	-5.97	5.97	0.37	7.32	20.85	19.03	1.82
RSE43	7.60	-0.42	1.80	0.24	4.69	30.04	29.66	0.38
W4-11	306.91	-3.96	4.09	0.01	6.51	37.84	37.81	0.03
G21EA	33.62	-3.97	4.20	0.12	5.16	10.20	9.83	0.37
G21IP	257.61	0.22	2.94	0.01	3.86	10.58	10.51	0.07
DIPCS10	654.26	-3.71	5.26	0.01	7.15	17.45	16.72	0.73
PA26	189.05	1.78	1.87	0.01	2.63	6.23	6.21	0.02
SIE4x4	33.72	11.00	11.00	0.33	13.29	32.05	29.89	2.16
ALKBDE10	100.69	42.85	42.85	0.43	48.90	89.31	75.41	13.90
RC21	35.70	2.31	2.83	0.08	3.20	5.78	5.19	0.59
ALK8	62.60	-2.08	2.53	0.04	3.21	6.63	6.05	0.58
DC13	54.98	-1.71	7.58	0.14	10.08	21.60	21.34	0.26
G2RC	51.26	-2.33	4.10	0.08	4.86	9.97	9.76	0.21
BH76RC	21.39	-0.11	1.80	0.08	2.18	4.80	4.80	0.00
MOR23	35.57	4.46	5.34	0.15	6.13	13.24	12.36	0.88
WCPT18	34.99	-0.01	1.88	0.05	2.46	7.22	7.17	0.05
BHROT27	6.37	0.25	0.36	0.06	0.53	1.28	1.28	0.00
BHPERI	20.87	4.12	4.20	0.20	4.67	10.79	9.70	1.09
BHDIV10	45.33	1.29	1.34	0.03	1.77	3.43	3.42	0.01
INV24	32.85	-0.57	1.36	0.04	2.13	7.26	7.18	0.08
CR20	19.31	-1.81	1.84	0.10	2.08	3.85	3.57	0.28
CRBH20	46.13	0.84	1.31	0.03	1.74	3.44	3.43	0.01
TMBH17	12.76	1.86	10.64	0.83	15.45	41.04	39.83	1.21
LTMBH26	9.98	1.49	2.23	0.22	4.09	17.34	17.26	0.08
BH76	18.61	-0.76	2.21	0.12	2.59	6.08	5.94	0.14
ISO34	14.57	-0.59	1.06	0.07	1.44	3.58	3.56	0.02
ICONF	3.27	0.22	0.40	0.12	0.54	1.17	1.14	0.03
ACONF	1.83	0.09	0.09	0.05	0.11	0.24	0.24	0.00
TAUT15	3.05	-0.32	1.09	0.36	1.26	2.46	2.44	0.02
Amino20x4	2.44	-0.02	0.23	0.09	0.30	0.86	0.86	0.00
PCONF	1.62	0.17	0.75	0.47	0.90	1.98	1.74	0.24
MCONF	4.97	-0.23	0.36	0.07	0.42	0.84	0.83	0.01

Continued on next page

Test set	$\vert\overline{\Delta E}\vert$	MD	MAD	NMAD	RMSD	Δ_{err}	max	min
SCONF	4.60	-0.08	0.15	0.03	0.19	0.56	0.54	0.02
PArel	4.63	0.08	0.64	0.14	0.84	1.71	1.69	0.02
BUT14DIOL	2.80	0.26	0.26	0.09	0.28	0.56	0.54	0.02
EIE22	5.44	0.17	0.40	0.07	0.45	0.89	0.86	0.03
Styrene45	62.64	-0.19	2.02	0.03	2.82	8.92	8.92	0.00
ISOMERIZATION20	31.84	-0.06	1.40	0.04	1.67	3.50	3.44	0.06
DIE60	4.71	0.09	0.65	0.14	0.91	2.19	2.19	0.00
IDISP	14.22	0.04	4.17	0.29	5.29	10.99	10.34	0.65
C20C24	30.77	1.81	17.40	0.57	19.03	25.66	23.95	1.71
S66	5.47	-0.38	0.56	0.10	0.74	2.45	2.45	0.00
S10x8	6.59	-0.33	0.42	0.06	0.61	1.70	1.70	0.00
X40	3.76	0.11	0.37	0.10	0.59	2.39	2.37	0.02
HEAVY28	1.24	0.29	0.29	0.23	0.36	0.80	0.79	0.01
CHB6	26.79	1.65	1.65	0.06	1.86	3.38	3.00	0.38
AHB21	22.49	-1.70	1.70	0.08	1.96	4.59	4.01	0.58
IL16	109.04	0.57	0.61	0.01	0.76	1.56	1.54	0.02
PNICO23	4.27	0.10	0.40	0.09	0.49	1.67	1.59	0.08
CT20	0.98	0.03	0.15	0.15	0.19	0.48	0.48	0.00
CARBHB12	6.04	1.03	1.03	0.17	1.13	2.53	2.06	0.47
ADIM6	3.36	-0.45	0.45	0.13	0.55	0.95	0.93	0.02
3B-69-TRIM	12.30	-0.37	0.69	0.06	0.92	2.26	2.24	0.02
ISOL24	21.92	-1.00	2.86	0.13	3.86	8.59	8.53	0.06
C60ISO	98.25	15.64	15.64	0.16	17.21	30.66	25.48	5.18
L7	18.20	3.53	3.79	0.21	5.00	9.31	8.98	0.33
UPU23	5.72	0.84	0.90	0.16	1.04	2.04	1.93	0.11
ENZYMES23	15.32	0.70	2.70	0.18	3.91	12.79	12.78	0.01

Table S95: Statistical analysis for PBE0-D4 for all testset in our databsase. The numbers given (all in kcal/mol) are average reaction energy ($|\overline{\Delta E}|$), mean deviation (MD), mean absolute deviation (MAD), MAD normalized with respect to $|\overline{\Delta E}|$ (NMAD), root-mean-square deviation (RMSD), deviation span (Δ_{err}), maximum (max) and minimum deviation (min).

Test set	$\vert\overline{\Delta E}\vert$	MD	MAD	NMAD	RMSD	Δ_{err}	max	min
FH51	31.01	-0.56	2.54	0.08	3.33	8.19	8.16	0.03
YBDE18	49.28	-3.27	3.27	0.07	3.78	8.51	7.92	0.59
AL2X6	35.88	-2.77	2.86	0.08	3.33	4.78	4.54	0.24
DARC	32.47	-0.18	3.23	0.10	3.57	7.06	6.56	0.50
NBPRC	27.71	-0.59	2.19	0.08	2.43	4.80	3.62	1.18
HEAVYSB9	58.02	-4.76	4.76	0.08	5.13	9.18	7.63	1.55
BSR36	16.20	-7.64	7.64	0.47	8.72	22.20	19.75	2.45
RSE43	7.60	-1.36	1.36	0.18	1.60	4.13	4.07	0.06
W4-11	306.91	-3.12	3.92	0.01	5.63	27.45	27.44	0.01

Continued on next page

Test set	$\lvert\overline{\Delta E}\rvert$	MD	MAD	NMAD	RMSD	Δ_{err}	max	min
G21EA	33.62	-3.98	4.62	0.14	6.00	12.02	12.01	0.01
G21IP	257.61	0.61	3.60	0.01	4.28	8.92	8.85	0.07
DIPCS10	654.26	0.02	2.80	0.00	3.05	5.18	4.72	0.46
PA26	189.05	1.24	1.69	0.01	2.41	7.07	7.06	0.01
SIE4x4	33.72	13.54	13.54	0.40	15.64	33.50	31.77	1.73
ALKBDE10	100.69	42.40	42.40	0.42	48.69	91.38	74.66	16.72
RC21	35.70	3.68	4.16	0.12	4.85	10.85	10.35	0.50
ALK8	62.60	1.76	1.99	0.03	2.98	6.59	6.54	0.05
DC13	54.98	-0.66	9.09	0.17	11.27	22.08	21.80	0.28
G2RC	51.26	-2.86	6.01	0.12	7.52	17.79	17.77	0.02
BH76RC	21.39	-0.36	2.08	0.10	2.78	7.05	6.80	0.25
MOR23	35.57	3.94	5.09	0.14	6.16	10.15	9.74	0.41
WCPT18	34.99	-3.88	3.88	0.11	4.39	9.43	8.50	0.93
BHROT27	6.37	0.49	0.56	0.09	0.74	1.47	1.47	0.00
BHPERI	20.87	-0.56	1.26	0.06	1.59	3.90	3.90	0.00
BHDIV10	45.33	-3.45	4.08	0.09	4.58	6.91	6.81	0.10
INV24	32.85	-1.09	1.56	0.05	1.95	5.24	4.93	0.31
CR20	19.31	-0.75	1.17	0.06	1.56	3.54	3.47	0.07
CRBH20	46.13	0.72	1.11	0.02	1.30	2.56	2.45	0.11
TMBH17	12.76	-0.45	8.82	0.69	13.93	35.57	35.48	0.09
LTMBH26	9.98	-0.73	2.47	0.25	3.90	14.77	14.72	0.05
BH76	18.61	-4.16	4.48	0.24	4.96	14.07	14.01	0.06
ISO34	14.57	-0.42	1.60	0.11	2.14	6.51	6.49	0.02
ICONF	3.27	0.30	0.45	0.14	0.60	1.48	1.40	0.08
ACONF	1.83	0.60	0.60	0.33	0.67	1.34	1.12	0.22
TAUT15	3.05	0.44	1.15	0.38	1.54	3.29	3.26	0.03
Amino20x4	2.44	0.03	0.47	0.19	0.59	1.78	1.78	0.00
PCONF	1.62	-0.40	3.00	1.85	3.36	5.25	5.21	0.04
MCONF	4.97	-1.50	1.57	0.32	1.79	2.99	2.98	0.01
SCONF	4.60	0.03	0.28	0.06	0.36	0.90	0.89	0.01
PArel	4.63	0.61	1.23	0.26	1.71	5.22	5.18	0.04
BUT14DIOL	2.80	0.02	0.21	0.07	0.27	0.72	0.72	0.00
EIE22	5.44	1.33	1.35	0.25	1.53	2.86	2.77	0.09
Styrene45	62.64	0.32	2.56	0.04	3.16	7.83	7.83	0.00
ISOMERIZATION20	31.84	0.13	1.92	0.06	2.51	5.73	5.69	0.04
DIE60	4.71	1.46	1.47	0.31	1.68	3.26	3.25	0.01
IDISP	14.22	1.10	9.46	0.67	10.29	17.13	14.63	2.50
C20C24	30.77	-11.33	12.98	0.42	20.12	39.60	38.94	0.66
S66	5.47	0.05	0.26	0.05	0.38	1.41	1.41	0.00
S10x8	6.59	-0.37	0.43	0.07	0.67	2.54	2.53	0.01
X40	3.76	1.25	1.32	0.35	1.74	5.98	5.93	0.05
HEAVY28	1.24	-0.53	0.58	0.47	0.65	1.34	1.31	0.03
CHB6	26.79	-0.04	0.58	0.02	0.70	1.38	1.23	0.15
AHB21	22.49	-1.90	1.93	0.09	2.43	5.02	4.98	0.04

Continued on next page

Test set	$\|\overline{\Delta E}\|$	MD	MAD	NMAD	RMSD	Δ_{err}	max	min
IL16	109.04	1.15	1.37	0.01	1.57	3.61	3.34	0.27
PNICO23	4.27	-0.39	0.62	0.15	0.83	2.40	2.36	0.04
CT20	0.98	-0.00	0.12	0.12	0.16	0.41	0.41	0.00
CARBHB12	6.04	0.87	0.88	0.15	1.20	2.75	2.69	0.06
ADIM6	3.36	-3.27	3.27	0.97	3.63	6.74	5.65	1.09
3B-69-TRIM	12.30	-0.22	0.63	0.05	0.86	2.40	2.39	0.01
ISOL24	21.92	0.23	1.95	0.09	2.80	7.78	7.67	0.11
C60ISO	98.25	1.43	2.51	0.03	2.62	4.50	3.62	0.88
L7	18.20	1.98	2.02	0.11	2.71	5.11	4.97	0.14
UPU23	5.72	0.47	0.59	0.10	0.70	1.60	1.59	0.01
ENZYMES23	15.32	-1.18	2.43	0.16	3.09	9.04	9.00	0.04

Table S96: Statistical analysis for TPSSH-D4 for all testset in our databsase. The numbers given (all in kcal/mol) are average reaction energy ($|\overline{\Delta E}|$), mean deviation (MD), mean absolute deviation (MAD), MAD normalized with respect to $|\overline{\Delta E}|$ (NMAD), root-mean-square deviation (RMSD), deviation span (Δ_{err}), maximum (max) and minimum deviation (min).

Test set	$\|\overline{\Delta E}\|$	MD	MAD	NMAD	RMSD	Δ_{err}	max	min
FH51	31.01	1.53	3.22	0.10	4.44	12.33	12.31	0.02
YBDE18	49.28	-4.82	4.89	0.10	5.24	8.60	8.00	0.60
AL2X6	35.88	0.72	2.13	0.06	2.28	4.69	3.53	1.16
DARC	32.47	2.35	2.96	0.09	3.23	5.63	4.49	1.14
NBPRC	27.71	-1.80	1.93	0.07	2.46	4.63	4.57	0.06
HEAVYSB9	58.02	-2.05	2.42	0.04	2.83	5.04	4.69	0.35
BSR36	16.20	-3.14	3.14	0.19	3.41	8.68	7.71	0.97
RSE43	7.60	-1.36	1.36	0.18	1.64	4.84	4.57	0.27
W4-11	306.91	-2.16	4.84	0.02	6.51	26.98	26.97	0.01
G21EA	33.62	-1.66	7.23	0.22	11.70	41.93	41.84	0.09
G21IP	257.61	0.02	3.63	0.01	4.40	11.40	11.05	0.35
DIPCS10	654.26	-1.03	2.81	0.00	3.39	7.31	6.73	0.58
PA26	189.05	2.90	2.90	0.02	3.71	9.94	9.81	0.13
SIE4x4	33.72	17.70	17.70	0.52	20.50	43.15	40.64	2.51
ALKBDE10	100.69	-2.75	5.81	0.06	7.18	14.52	14.22	0.30
RC21	35.70	2.96	3.74	0.10	4.08	8.82	7.39	1.43
ALK8	62.60	2.63	2.63	0.04	3.29	6.00	5.44	0.56
DC13	54.98	0.26	7.56	0.14	10.33	22.15	21.90	0.25
G2RC	51.26	1.50	5.88	0.11	7.35	16.91	16.63	0.28
BH76RC	21.39	-0.25	3.08	0.14	4.10	9.16	9.10	0.06
MOR23	35.57	-0.63	6.51	0.18	13.44	59.47	59.40	0.07
WCPT18	34.99	-4.67	4.67	0.13	5.14	10.11	8.82	1.29
BHROT27	6.37	0.39	0.54	0.08	0.70	1.55	1.52	0.03
BHPERI	20.87	-4.26	4.26	0.20	4.53	8.69	7.15	1.54

Continued on next page

Test set	$\lvert\overline{\Delta E}\rvert$	MD	MAD	NMAD	RMSD	Δ_{err}	max	min
BHDIV10	45.33	-4.98	5.34	0.12	5.77	11.44	9.64	1.80
INV24	32.85	-1.10	1.32	0.04	1.64	4.03	3.95	0.08
CR20	19.31	-1.31	1.62	0.08	1.83	3.46	3.19	0.27
CRBH20	46.13	-6.07	6.07	0.13	6.11	12.14	7.30	4.84
TMBH17	12.76	-0.36	4.15	0.33	4.78	8.61	8.05	0.56
LTMBH26	9.98	-2.36	3.79	0.38	4.96	15.76	15.38	0.38
BH76	18.61	-7.42	7.47	0.40	8.25	17.27	17.21	0.06
ISO34	14.57	-1.35	2.02	0.14	2.78	9.99	9.88	0.11
ICONF	3.27	0.03	0.18	0.05	0.23	0.57	0.55	0.02
ACONF	1.83	-0.13	0.13	0.07	0.14	0.30	0.27	0.03
TAUT15	3.05	0.22	1.34	0.44	1.57	2.92	2.85	0.07
Amino20x4	2.44	0.05	0.30	0.12	0.37	1.01	1.01	0.00
PCONF	1.62	-0.29	0.68	0.42	0.83	1.70	1.63	0.07
MCONF	4.97	0.17	0.60	0.12	1.19	5.83	5.83	0.00
SCONF	4.60	0.36	0.99	0.22	1.22	3.04	3.01	0.03
PArel	4.63	0.27	1.30	0.28	1.76	4.98	4.87	0.11
BUT14DIOL	2.80	0.37	0.37	0.13	0.39	0.72	0.71	0.01
EIE22	5.44	1.64	1.66	0.30	1.83	3.46	3.32	0.14
Styrene45	62.64	0.97	2.28	0.04	3.00	8.07	8.07	0.00
ISOMERIZATION20	31.84	1.18	2.89	0.09	3.72	8.52	8.51	0.01
DIE60	4.71	1.62	1.62	0.34	1.74	3.11	2.88	0.23
IDISP	14.22	1.68	1.78	0.13	2.44	5.59	5.29	0.30
C20C24	30.77	-15.61	15.81	0.51	25.18	49.70	49.11	0.59
S66	5.47	-0.18	0.34	0.06	0.39	0.93	0.92	0.01
S10x8	6.59	-0.17	0.30	0.04	0.44	1.72	1.71	0.01
X40	3.76	0.12	0.33	0.09	0.50	1.73	1.72	0.01
HEAVY28	1.24	0.28	0.35	0.28	0.44	1.18	1.10	0.08
CHB6	26.79	0.56	0.64	0.02	0.86	1.88	1.75	0.13
AHB21	22.49	-1.07	1.24	0.06	1.55	3.09	3.00	0.09
IL16	109.04	-0.12	0.38	0.00	0.47	1.10	1.08	0.02
PNICO23	4.27	0.86	0.93	0.22	1.19	3.53	3.47	0.06
CT20	0.98	0.12	0.19	0.19	0.22	0.41	0.40	0.01
CARBHB12	6.04	1.33	1.33	0.22	1.60	3.51	3.11	0.40
ADIM6	3.36	-0.19	0.21	0.06	0.27	0.54	0.52	0.02
3B-69-TRIM	12.30	0.23	0.61	0.05	0.81	2.22	2.22	0.00
ISOL24	21.92	-2.16	3.95	0.18	5.34	13.09	12.92	0.17
C60ISO	98.25	-4.44	4.65	0.05	6.33	12.49	12.37	0.12
L7	18.20	1.00	1.00	0.05	1.28	2.27	2.23	0.04
UPU23	5.72	0.38	0.51	0.09	0.64	1.38	1.37	0.01
ENZYMES23	15.32	-3.65	4.61	0.30	5.67	11.63	11.13	0.50

Table S97: Statistical analysis for PW6B95-D4 for all testset in our databsase. The numbers given (all in kcal/mol) are average reaction energy ($|\overline{\Delta E}|$), mean deviation (MD), mean absolute deviation (MAD), MAD normalized with respect to $|\overline{\Delta E}|$ (NMAD), root-mean-square deviation (RMSD), deviation span (Δ_{err}), maximum (max) and minimum deviation (min).

Test set	$\lvert\overline{\Delta E}\rvert$	MD	MAD	NMAD	RMSD	Δ_{err}	max	min
FH51	31.01	0.33	1.57	0.05	2.18	6.84	6.82	0.02
YBDE18	49.28	35.09	35.09	0.71	48.68	70.37	70.36	0.01
AL2X6	35.88	5.19	5.19	0.14	5.36	11.86	7.77	4.09
DARC	32.47	-6.10	6.10	0.19	6.46	13.24	9.41	3.83
NBPRC	27.71	-3.88	3.88	0.14	4.08	8.67	6.28	2.39
HEAVYSB9	58.02	-2.72	2.72	0.05	3.50	8.00	7.66	0.34
BSR36	16.20	-1.93	1.93	0.12	2.12	5.35	5.00	0.35
RSE43	7.60	-1.90	1.90	0.25	2.18	5.39	4.95	0.44
W4-11	306.91	-1.71	2.65	0.01	4.75	31.16	31.13	0.03
G21EA	33.62	5.12	5.99	0.18	9.75	33.00	32.25	0.75
G21IP	257.61	1.46	2.81	0.01	3.65	9.23	9.22	0.01
DIPCS10	654.26	1.70	2.85	0.00	3.29	5.81	5.63	0.18
PA26	189.05	1.11	1.55	0.01	2.23	6.82	6.79	0.03
SIE4x4	33.72	14.81	14.81	0.44	17.15	35.89	34.61	1.28
ALKBDE10	100.69	-1.88	4.10	0.04	5.17	11.73	11.02	0.71
RC21	35.70	2.53	2.90	0.08	3.48	7.09	7.06	0.03
ALK8	62.60	2.77	2.86	0.05	3.98	7.17	7.13	0.04
DC13	54.98	-0.38	7.28	0.13	9.06	17.85	17.11	0.74
G2RC	51.26	-0.79	3.06	0.06	3.91	9.14	9.10	0.04
BH76RC	21.39	-0.15	1.54	0.07	2.03	4.96	4.90	0.06
MOR23	35.57	4.21	5.16	0.15	13.65	63.96	63.80	0.16
WCPT18	34.99	-1.86	1.90	0.05	2.25	5.06	4.88	0.18
BHROT27	6.37	0.47	0.56	0.09	0.74	1.52	1.50	0.02
BHPERI	20.87	-0.93	1.39	0.07	1.63	3.16	3.08	0.08
BHDIV10	45.33	-2.46	2.74	0.06	2.90	5.36	4.04	1.32
INV24	32.85	-0.79	1.40	0.04	1.73	3.74	3.66	0.08
CR20	19.31	3.59	3.59	0.19	3.68	6.97	5.05	1.92
CRBH20	46.13	0.46	0.61	0.01	0.71	1.43	1.39	0.04
TMBH17	12.76	2.52	3.27	0.26	4.51	9.40	9.07	0.33
LTMBH26	9.98	-0.47	2.16	0.22	3.78	15.35	15.20	0.15
BH76	18.61	-3.86	4.01	0.22	4.46	9.66	9.41	0.25
ISO34	14.57	-0.56	1.20	0.08	1.51	3.82	3.74	0.08
ICONF	3.27	0.09	0.28	0.09	0.41	1.02	1.01	0.01
ACONF	1.83	-0.22	0.22	0.12	0.26	0.52	0.47	0.05
TAUT15	3.05	0.28	0.86	0.28	1.16	2.55	2.52	0.03
Amino20x4	2.44	0.06	0.29	0.12	0.39	1.13	1.13	0.00
PCONF	1.62	0.40	0.49	0.30	0.59	1.04	1.03	0.01
MCONF	4.97	0.14	0.35	0.07	0.42	0.96	0.95	0.01

Continued on next page

Test set	$\lvert\overline{\Delta E}\rvert$	MD	MAD	NMAD	RMSD	Δ_{err}	max	min
SCONF	4.60	0.13	0.41	0.09	0.53	1.52	1.48	0.04
PArel	4.63	0.49	0.91	0.20	1.45	4.95	4.95	0.00
BUT14DIOL	2.80	-0.11	0.12	0.04	0.17	0.47	0.47	0.00
EIE22	5.44	1.13	1.15	0.21	1.30	2.33	2.30	0.03
Styrene45	62.64	0.91	2.02	0.03	2.48	5.72	5.72	0.00
ISOMERIZATION20	31.84	2.25	4.28	0.13	9.75	41.94	41.81	0.13
DIE60	4.71	1.22	1.22	0.26	1.38	2.50	2.49	0.01
IDISP	14.22	1.49	2.35	0.17	3.48	8.14	7.71	0.43
C20C24	30.77	-13.24	13.24	0.43	13.55	28.36	18.09	10.27
S66	5.47	-0.18	0.29	0.05	0.52	2.83	2.83	0.00
S10x8	6.59	1.72	1.77	0.27	10.65	83.57	83.57	0.00
X40	3.76	0.23	0.31	0.08	0.39	1.14	1.12	0.02
HEAVY28	1.24	0.61	0.64	0.52	0.68	1.46	1.20	0.26
CHB6	26.79	-0.35	0.81	0.03	1.04	2.19	2.15	0.04
AHB21	22.49	-0.89	1.07	0.05	1.43	3.53	3.50	0.03
IL16	109.04	0.36	0.52	0.00	0.61	1.10	1.08	0.02
PNICO23	4.27	0.20	0.28	0.06	0.37	1.13	1.12	0.01
CT20	0.98	0.01	0.09	0.09	0.12	0.22	0.22	0.00
CARBHB12	6.04	0.65	0.65	0.11	0.76	1.83	1.61	0.22
ADIM6	3.36	0.43	0.43	0.13	0.45	1.01	0.67	0.34
3B-69-TRIM	12.30	-0.10	2.28	0.19	9.80	74.31	74.30	0.01
ISOL24	21.92	-1.38	3.18	0.15	4.09	9.77	9.44	0.33
C60ISO	98.25	1.30	2.22	0.02	2.33	3.84	3.23	0.61
L7	18.20	3.94	3.94	0.22	4.59	8.14	7.01	1.13
UPU23	5.72	0.59	0.68	0.12	0.81	1.51	1.49	0.02
ENZYMES23	15.32	-1.74	3.19	0.21	3.93	12.00	11.33	0.67

Table S98: Statistical analysis for mPWB1K-D4 for all testset in our databsase. The numbers given (all in kcal/mol) are average reaction energy ($\lvert\overline{\Delta E}\rvert$), mean deviation (MD), mean absolute deviation (MAD), MAD normalized with respect to $\lvert\overline{\Delta E}\rvert$ (NMAD), root-mean-square deviation (RMSD), deviation span (Δ_{err}), maximum (max) and minimum deviation (min).

Test set	$\lvert\overline{\Delta E}\rvert$	MD	MAD	NMAD	RMSD	Δ_{err}	max	min
FH51	31.01	-1.61	2.23	0.07	2.79	6.85	6.76	0.09
YBDE18	49.28	22.07	22.34	0.45	29.78	46.17	44.66	1.51
AL2X6	35.88	4.75	4.75	0.13	4.89	10.84	7.04	3.80
DARC	32.47	-4.08	4.08	0.13	4.49	9.57	7.28	2.29
NBPRC	27.71	-3.62	3.62	0.13	3.73	8.06	5.32	2.74
HEAVYSB9	58.02	-1.89	1.89	0.03	2.02	4.53	3.34	1.19
BSR36	16.20	-2.04	2.04	0.13	2.18	4.94	4.46	0.48
RSE43	7.60	-1.23	1.23	0.16	1.48	3.76	3.62	0.14
W4-11	306.91	-8.32	8.50	0.03	10.99	38.13	38.04	0.09

Continued on next page

Test set	$\lvert\overline{\Delta E}\rvert$	MD	MAD	NMAD	RMSD	Δ_{err}	max	min
G21EA	33.62	5.02	5.92	0.18	9.69	32.62	31.85	0.77
G21IP	257.61	-0.39	2.87	0.01	3.70	9.25	9.12	0.13
DIPCS10	654.26	-2.04	3.36	0.01	4.41	8.69	8.59	0.10
PA26	189.05	1.11	1.76	0.01	2.40	6.37	6.33	0.04
SIE4x4	33.72	9.08	9.08	0.27	11.01	25.25	24.73	0.52
ALKBDE10	100.69	-6.61	10.42	0.10	11.83	27.47	22.50	4.97
RC21	35.70	3.48	3.83	0.11	4.73	13.03	12.43	0.60
ALK8	62.60	3.94	3.94	0.06	5.35	8.83	8.78	0.05
DC13	54.98	-2.81	9.29	0.17	11.55	24.56	24.43	0.13
G2RC	51.26	-3.79	5.37	0.10	6.74	16.68	16.52	0.16
BH76RC	21.39	-0.11	2.60	0.12	2.94	6.61	6.20	0.41
MOR23	35.57	3.49	4.94	0.14	14.03	65.69	65.58	0.11
WCPT18	34.99	0.01	0.94	0.03	1.37	4.26	4.24	0.02
BHROT27	6.37	0.63	0.68	0.11	0.92	1.95	1.91	0.04
BHPERI	20.87	0.98	1.67	0.08	2.40	6.93	6.89	0.04
BHDIV10	45.33	-0.78	1.37	0.03	1.59	3.74	3.20	0.54
INV24	32.85	0.25	1.96	0.06	3.05	10.23	10.11	0.12
CR20	19.31	4.49	4.49	0.23	4.60	8.34	6.10	2.24
CRBH20	46.13	5.62	5.62	0.12	5.69	10.97	7.02	3.95
TMBH17	12.76	4.67	5.29	0.41	6.50	12.55	12.33	0.22
LTMBH26	9.98	0.91	1.88	0.19	3.68	15.82	15.78	0.04
BH76	18.61	-0.96	1.67	0.09	2.10	6.40	6.33	0.07
ISO34	14.57	-0.39	1.51	0.10	1.94	4.22	4.16	0.06
ICONF	3.27	0.20	0.45	0.14	0.60	1.43	1.39	0.04
ACONF	1.83	-0.14	0.14	0.07	0.17	0.43	0.43	0.00
TAUT15	3.05	0.47	0.80	0.26	1.07	2.06	2.02	0.04
Amino20x4	2.44	0.10	0.39	0.16	0.51	1.42	1.42	0.00
PCONF	1.62	0.65	0.70	0.43	0.84	1.61	1.51	0.10
MCONF	4.97	0.28	0.39	0.08	0.48	0.97	0.93	0.04
SCONF	4.60	0.10	0.24	0.05	0.33	0.98	0.96	0.02
PArel	4.63	0.89	1.12	0.24	1.60	4.45	4.45	0.00
BUT14DIOL	2.80	-0.20	0.20	0.07	0.22	0.59	0.57	0.02
EIE22	5.44	0.79	0.83	0.15	0.97	1.90	1.84	0.06
Styrene45	62.64	0.16	3.25	0.05	4.32	12.28	12.28	0.00
ISOMERIZATION20	31.84	1.36	2.90	0.09	6.57	28.06	28.03	0.03
DIE60	4.71	1.05	1.10	0.23	1.34	2.98	2.96	0.02
IDISP	14.22	-0.15	1.59	0.11	2.02	3.61	3.34	0.27
C20C24	30.77	-14.66	14.66	0.48	17.05	29.18	27.02	2.16
S66	5.47	-0.37	0.78	0.14	2.38	13.22	13.20	0.02
S10x8	6.59	0.86	1.11	0.17	5.33	42.21	42.20	0.01
X40	3.76	0.41	0.45	0.12	0.54	1.24	1.24	0.00
HEAVY28	1.24	0.21	0.25	0.20	0.32	0.84	0.82	0.02
CHB6	26.79	-0.49	0.92	0.03	1.30	3.05	2.92	0.13
AHB21	22.49	-1.15	1.36	0.06	1.98	5.82	5.76	0.06

Continued on next page

Test set	$\|\overline{\Delta E}\|$	MD	MAD	NMAD	RMSD	Δ_{err}	max	min
IL16	109.04	0.74	0.76	0.01	0.89	1.56	1.55	0.01
PNICO23	4.27	-0.04	0.19	0.05	0.27	0.76	0.75	0.01
CT20	0.98	-3.22	3.70	3.76	10.44	34.64	34.61	0.03
CARBHB12	6.04	0.49	0.49	0.08	0.62	1.52	1.44	0.08
ADIM6	3.36	0.10	0.10	0.03	0.14	0.32	0.31	0.01
3B-69-TRIM	12.30	0.13	1.31	0.11	4.26	33.39	33.31	0.08
ISOL24	21.92	0.35	2.46	0.11	3.15	7.45	7.42	0.03
C60ISO	98.25	8.22	8.22	0.08	8.65	17.55	13.23	4.32
L7	18.20	1.65	1.65	0.09	1.77	3.56	2.53	1.03
UPU23	5.72	0.53	0.63	0.11	0.76	1.48	1.40	0.08
ENZYMES23	15.32	0.35	2.21	0.14	3.58	12.29	12.27	0.02

Table S99: Statistical analysis for mPW1PW-D4 for all testset in our databsase. The numbers given (all in kcal/mol) are average reaction energy ($|\overline{\Delta E}|$), mean deviation (MD), mean absolute deviation (MAD), MAD normalized with respect to $|\overline{\Delta E}|$ (NMAD), root-mean-square deviation (RMSD), deviation span (Δ_{err}), maximum (max) and minimum deviation (min).

Test set	$\|\overline{\Delta E}\|$	MD	MAD	NMAD	RMSD	Δ_{err}	max	min
FH51	31.01	-0.68	2.23	0.07	2.99	8.16	8.05	0.11
YBDE18	49.28	-3.04	3.04	0.06	3.30	7.07	6.31	0.76
AL2X6	35.88	-0.36	1.10	0.03	1.56	3.66	3.29	0.37
DARC	32.47	-2.22	2.25	0.07	3.30	6.84	6.82	0.02
NBPRC	27.71	-1.35	1.94	0.07	2.21	3.22	3.19	0.03
HEAVYSB9	58.02	-2.94	2.94	0.05	3.15	5.45	4.35	1.10
BSR36	16.20	-2.55	2.55	0.16	2.76	7.17	5.94	1.23
RSE43	7.60	-1.09	1.09	0.14	1.34	3.60	3.57	0.03
W4-11	306.91	-6.30	6.46	0.02	7.89	31.21	30.99	0.22
G21EA	33.62	-2.93	4.15	0.12	5.40	11.31	11.16	0.15
G21IP	257.61	1.83	3.94	0.02	4.76	10.79	10.57	0.22
DIPCS10	654.26	2.85	3.77	0.01	4.33	8.82	7.92	0.90
PA26	189.05	2.09	2.14	0.01	2.92	8.22	8.06	0.16
SIE4x4	33.72	15.74	15.74	0.47	17.52	36.99	32.25	4.74
ALKBDE10	100.69	-4.48	6.84	0.07	8.00	15.67	14.23	1.44
RC21	35.70	3.43	3.83	0.11	4.50	9.95	9.54	0.41
ALK8	62.60	2.58	2.58	0.04	3.12	5.42	5.18	0.24
DC13	54.98	-0.74	6.60	0.12	8.67	17.79	17.73	0.06
G2RC	51.26	-2.26	5.20	0.10	6.62	18.06	18.01	0.05
BH76RC	21.39	-0.37	1.86	0.09	2.52	6.63	6.51	0.12
MOR23	35.57	-2.23	3.35	0.09	4.38	11.79	11.75	0.04
WCPT18	34.99	-3.24	3.24	0.09	3.94	8.01	8.00	0.01
BHROT27	6.37	0.47	0.52	0.08	0.70	1.44	1.42	0.02
BHPERI	20.87	-2.39	2.51	0.12	2.89	5.31	5.07	0.24

Continued on next page

Test set	$\lvert\overline{\Delta E}\rvert$	MD	MAD	NMAD	RMSD	Δ_{err}	max	min
BHDIV10	45.33	-3.29	3.96	0.09	4.39	6.97	6.81	0.16
INV24	32.85	-0.44	1.19	0.04	1.67	4.78	4.73	0.05
CR20	19.31	-0.70	0.93	0.05	1.20	2.79	2.62	0.17
CRBH20	46.13	-0.65	0.86	0.02	1.10	2.49	2.42	0.07
TMBH17	12.76	1.97	3.32	0.26	4.21	7.68	7.56	0.12
LTMBH26	9.98	-1.37	3.01	0.30	4.22	14.80	14.62	0.18
BH76	18.61	-3.99	4.29	0.23	4.64	11.62	11.21	0.41
ISO34	14.57	-0.23	1.18	0.08	1.57	5.05	5.04	0.01
ICONF	3.27	0.16	0.28	0.09	0.43	1.22	1.19	0.03
ACONF	1.83	0.00	0.02	0.01	0.03	0.07	0.07	0.00
TAUT15	3.05	0.28	1.09	0.36	1.35	2.79	2.66	0.13
Amino20x4	2.44	0.03	0.24	0.10	0.33	1.17	1.17	0.00
PCONF	1.62	-0.07	0.60	0.37	0.68	1.25	1.13	0.12
MCONF	4.97	0.09	0.28	0.06	0.33	0.70	0.69	0.01
SCONF	4.60	0.11	0.44	0.10	0.62	1.88	1.88	0.00
PArel	4.63	0.51	1.01	0.22	1.53	5.07	5.06	0.01
BUT14DIOL	2.80	0.34	0.34	0.12	0.36	0.76	0.73	0.03
EIE22	5.44	1.19	1.23	0.23	1.40	2.77	2.60	0.17
Styrene45	62.64	1.74	2.34	0.04	2.95	8.51	8.51	0.00
ISOMERIZATION20	31.84	0.08	1.92	0.06	2.46	5.32	5.12	0.20
DIE60	4.71	1.32	1.33	0.28	1.52	2.85	2.76	0.09
IDISP	14.22	0.58	1.32	0.09	1.74	3.48	3.38	0.10
C20C24	30.77	-11.79	11.79	0.38	16.16	31.94	29.89	2.05
S66	5.47	-0.24	0.37	0.07	0.44	1.04	1.04	0.00
S10x8	6.59	-0.21	0.32	0.05	0.51	2.19	2.19	0.00
X40	3.76	0.20	0.38	0.10	0.53	1.86	1.85	0.01
HEAVY28	1.24	0.16	0.28	0.22	0.36	0.98	0.98	0.00
CHB6	26.79	0.43	0.56	0.02	0.75	1.53	1.51	0.02
AHB21	22.49	-1.33	1.49	0.07	2.00	4.42	4.39	0.03
IL16	109.04	-0.02	0.58	0.01	0.67	1.36	1.26	0.10
PNICO23	4.27	0.54	0.64	0.15	0.85	2.79	2.71	0.08
CT20	0.98	0.26	0.26	0.27	0.30	0.57	0.55	0.02
CARBHB12	6.04	1.22	1.22	0.20	1.49	3.25	2.95	0.30
ADIM6	3.36	-0.51	0.51	0.15	0.56	1.07	0.89	0.18
3B-69-TRIM	12.30	0.27	0.64	0.05	0.86	2.16	2.16	0.00
ISOL24	21.92	-0.10	1.88	0.09	2.71	7.31	7.22	0.09
C60ISO	98.25	1.56	2.55	0.03	2.66	4.75	3.70	1.05
L7	18.20	0.98	0.98	0.05	1.14	1.81	1.81	0.00
UPU23	5.72	0.59	0.68	0.12	0.81	1.73	1.69	0.04
ENZYMES23	15.32	-1.32	2.79	0.18	3.58	10.32	10.23	0.09

Table S100: Statistical analysis for M06-D4 for all testset in our databsase. The numbers given (all in kcal/mol) are average reaction energy ($|\overline{\Delta E}|$), mean deviation (MD), mean absolute deviation (MAD), MAD normalized with respect to $|\overline{\Delta E}|$ (NMAD), root-mean-square deviation (RMSD), deviation span (Δ_{err}), maximum (max) and minimum deviation (min).

Test set	$\|\overline{\Delta E}\|$	MD	MAD	NMAD	RMSD	Δ_{err}	max	min
FH51	31.01	-0.20	1.89	0.06	2.44	5.80	5.79	0.01
YBDE18	49.28	34.31	34.84	0.71	48.78	70.65	70.56	0.09
AL2X6	35.88	3.65	3.65	0.10	4.22	9.80	7.78	2.02
DARC	32.47	-5.61	5.61	0.17	5.75	12.23	7.83	4.40
NBPRC	27.71	-2.62	2.62	0.09	3.13	5.46	5.19	0.27
HEAVYSB9	58.02	-0.10	1.78	0.03	1.92	3.81	2.98	0.83
BSR36	16.20	-0.51	0.70	0.04	0.98	3.67	3.65	0.02
RSE43	7.60	-1.43	1.43	0.19	1.82	6.59	6.31	0.28
W4-11	306.91	-2.11	4.49	0.01	6.01	20.92	20.83	0.09
G21EA	33.62	-5.13	5.27	0.16	5.99	11.69	10.93	0.76
G21IP	257.61	-0.91	3.03	0.01	3.86	8.96	8.89	0.07
DIPCS10	654.26	-5.12	5.35	0.01	6.97	13.30	12.96	0.34
PA26	189.05	1.68	2.10	0.01	3.00	8.34	8.25	0.09
SIE4x4	33.72	11.89	11.89	0.35	13.29	23.77	21.37	2.40
ALKBDE10	100.69	-0.18	3.84	0.04	4.95	11.49	11.26	0.23
RC21	35.70	0.50	1.59	0.04	2.11	4.28	4.27	0.01
ALK8	62.60	0.23	3.28	0.05	3.89	7.11	6.12	0.99
DC13	54.98	-1.29	7.15	0.13	9.56	20.17	19.19	0.98
G2RC	51.26	-0.39	3.88	0.08	5.30	13.84	13.78	0.06
BH76RC	21.39	-0.68	1.60	0.07	2.20	6.18	6.05	0.13
MOR23	35.57	2.86	4.07	0.11	5.30	12.28	12.12	0.16
WCPT18	34.99	-1.99	2.15	0.06	2.68	5.50	5.41	0.09
BHROT27	6.37	0.63	0.65	0.10	0.88	2.24	2.23	0.01
BHPERI	20.87	1.23	1.80	0.09	2.28	5.00	4.76	0.24
BHDIV10	45.33	-1.38	1.89	0.04	2.44	4.55	4.44	0.11
INV24	32.85	-0.78	1.42	0.04	2.24	7.34	7.34	0.00
CR20	19.31	-2.54	2.54	0.13	2.77	5.46	4.80	0.66
CRBH20	46.13	-3.57	3.57	0.08	3.62	7.29	4.79	2.50
TMBH17	12.76	2.71	3.17	0.25	4.78	11.84	11.73	0.11
LTMBH26	9.98	-0.33	2.84	0.28	4.50	17.98	17.87	0.11
BH76	18.61	-2.24	2.78	0.15	3.38	10.37	10.33	0.04
ISO34	14.57	-0.64	1.40	0.10	1.85	4.64	4.63	0.01
ICONF	3.27	-0.06	0.43	0.13	0.57	1.24	1.24	0.00
ACONF	1.83	-0.35	0.35	0.19	0.40	0.72	0.67	0.05
TAUT15	3.05	-0.34	0.83	0.27	1.05	2.28	2.24	0.04
Amino20x4	2.44	-0.03	0.35	0.14	0.43	1.24	1.24	0.00
PCONF	1.62	0.33	1.06	0.65	1.19	1.86	1.84	0.02
MCONF	4.97	0.43	0.54	0.11	0.63	1.34	1.30	0.04

Continued on next page

Test set	$\lvert\overline{\Delta E}\rvert$	MD	MAD	NMAD	RMSD	Δ_{err}	max	min
SCONF	4.60	-0.13	0.29	0.06	0.37	1.00	1.00	0.00
PArel	4.63	0.56	0.97	0.21	1.62	5.73	5.71	0.02
BUT14DIOL	2.80	0.25	0.26	0.09	0.30	0.59	0.56	0.03
EIE22	5.44	1.08	1.11	0.20	1.31	2.61	2.51	0.10
Styrene45	62.64	-2.51	3.68	0.06	4.91	12.50	12.50	0.00
ISOMERIZATION20	31.84	1.97	4.41	0.14	10.39	44.45	44.42	0.03
DIE60	4.71	1.19	1.24	0.26	1.51	3.19	3.12	0.07
IDISP	14.22	1.64	3.70	0.26	4.43	8.75	7.13	1.62
C20C24	30.77	-15.53	15.53	0.50	23.69	46.49	45.76	0.73
S66	5.47	-0.29	0.45	0.08	0.51	1.11	1.11	0.00
S10x8	6.59	0.39	0.44	0.07	0.51	1.01	1.01	0.00
X40	3.76	0.34	0.44	0.12	0.50	0.90	0.90	0.00
HEAVY28	1.24	0.95	0.99	0.80	1.10	2.23	1.87	0.36
CHB6	26.79	2.29	2.29	0.09	2.54	4.49	3.53	0.96
AHB21	22.49	-0.73	0.82	0.04	1.16	3.19	3.15	0.04
IL16	109.04	-0.10	0.62	0.01	0.77	2.07	1.95	0.12
PNICO23	4.27	0.07	0.21	0.05	0.26	0.52	0.52	0.00
CT20	0.98	0.63	0.63	0.64	0.68	1.03	1.01	0.02
CARBHB12	6.04	0.25	0.34	0.06	0.43	1.01	0.98	0.03
ADIM6	3.36	1.08	1.08	0.32	1.34	2.48	2.47	0.01
3B-69-TRIM	12.30	1.12	1.13	0.09	1.31	3.03	2.96	0.07
ISOL24	21.92	-2.23	3.27	0.15	5.14	19.40	19.20	0.20
C60ISO	98.25	-0.33	1.84	0.02	2.30	4.80	4.73	0.07
L7	18.20	0.95	1.15	0.06	1.43	2.74	2.53	0.21
UPU23	5.72	-0.20	0.66	0.12	0.98	3.72	3.70	0.02
ENZYMES23	15.32	-2.64	4.42	0.29	5.51	16.07	15.72	0.35

S5.6 Dispersion-Uncorrected Hybrids

Table S101: Statistical analysis for B3LYP for all testset in our databsase. The numbers given (all in kcal/mol) are average reaction energy ($\lvert\overline{\Delta E}\rvert$), mean deviation (MD), mean absolute deviation (MAD), MAD normalized with respect to $\lvert\overline{\Delta E}\rvert$ (NMAD), root-mean-square deviation (RMSD), deviation span (Δ_{err}), maximum (max) and minimum deviation (min).

Test set	$\lvert\overline{\Delta E}\rvert$	MD	MAD	NMAD	RMSD	Δ_{err}	max	min
FH51	31.01	4.02	4.02	0.13	5.23	16.39	16.21	0.18
YBDE18	49.28	-9.20	9.31	0.19	10.62	19.19	18.19	1.00
AL2X6	35.88	-8.92	8.92	0.25	9.49	19.12	14.01	5.11
DARC	32.47	15.67	15.67	0.48	15.97	27.04	18.73	8.31
NBPRC	27.71	4.70	5.80	0.21	8.04	19.52	19.14	0.38
HEAVYSB9	58.02	-8.99	8.99	0.15	9.37	19.52	13.78	5.74

Continued on next page

Test set	$\vert\overline{\Delta E}\vert$	MD	MAD	NMAD	RMSD	Δ_{err}	max	min
BSR36	16.20	-10.58	10.58	0.65	12.09	30.65	27.56	3.09
RSE43	7.60	-1.74	1.74	0.23	2.02	5.03	4.75	0.28
W4-11	306.91	-4.33	5.09	0.02	7.09	28.96	28.90	0.06
G21EA	33.62	-3.81	4.40	0.13	5.16	11.21	10.52	0.69
G21IP	257.61	0.38	3.51	0.01	4.41	10.19	10.18	0.01
DIPCS10	654.26	-1.18	4.12	0.01	5.16	9.94	9.15	0.79
PA26	189.05	1.16	1.86	0.01	2.74	8.16	8.01	0.15
SIE4x4	33.72	17.08	17.08	0.51	19.97	42.24	40.51	1.73
ALKBDE10	100.69	44.50	44.50	0.44	50.79	92.95	78.46	14.49
RC21	35.70	-0.98	2.21	0.06	3.28	10.88	10.82	0.06
ALK8	62.60	-5.87	5.87	0.09	8.78	18.54	18.29	0.25
DC13	54.98	4.72	16.07	0.29	21.23	39.30	38.81	0.49
G2RC	51.26	1.17	2.60	0.05	3.45	8.06	8.02	0.04
BH76RC	21.39	-0.60	2.38	0.11	2.98	7.20	7.01	0.19
MOR23	35.57	12.51	13.87	0.39	18.98	68.01	67.29	0.72
WCPT18	34.99	1.35	1.35	0.04	1.71	3.64	3.60	0.04
BHROT27	6.37	0.44	0.44	0.07	0.58	1.36	1.33	0.03
BHPERI	20.87	4.44	4.44	0.21	5.16	9.57	9.32	0.25
BHDIV10	45.33	2.80	2.80	0.06	3.53	6.13	6.10	0.03
INV24	32.85	1.88	1.88	0.06	2.18	4.75	4.32	0.43
CR20	19.31	-11.58	11.58	0.60	11.73	23.82	15.87	7.95
CRBH20	46.13	-8.83	8.83	0.19	8.92	19.09	11.68	7.41
TMBH17	12.76	2.97	4.05	0.32	5.82	17.03	16.99	0.04
LTMBH26	9.98	-1.77	3.49	0.35	4.98	17.21	16.92	0.29
BH76	18.61	-4.91	5.07	0.27	5.82	11.72	11.55	0.17
ISO34	14.57	2.38	2.38	0.16	3.47	11.71	11.71	0.00
ICONF	3.27	0.58	0.58	0.18	0.78	1.88	1.84	0.04
ACONF	1.83	0.92	0.92	0.50	1.03	2.14	1.80	0.34
TAUT15	3.05	1.14	1.14	0.37	1.35	2.12	2.10	0.02
Amino20x4	2.44	0.66	0.66	0.27	0.83	2.36	2.35	0.01
PCONF	1.62	3.80	3.80	2.35	4.34	7.23	6.99	0.24
MCONF	4.97	2.50	2.50	0.50	2.79	4.22	4.14	0.08
SCONF	4.60	0.60	0.60	0.13	0.75	1.77	1.75	0.02
PArel	4.63	-0.08	1.17	0.25	1.73	5.32	5.31	0.01
BUT14DIOL	2.80	-0.09	0.33	0.12	0.40	0.86	0.85	0.01
EIE22	5.44	1.49	1.49	0.27	1.65	2.98	2.95	0.03
Styrene45	62.64	3.67	5.96	0.10	7.69	18.59	18.59	0.00
ISOMERIZATION20	31.84	0.21	2.18	0.07	2.91	7.53	7.50	0.03
DIE60	4.71	1.32	1.34	0.28	1.49	2.85	2.85	0.00
IDISP	14.22	16.38	16.38	1.15	18.96	38.49	34.77	3.72
C20C24	30.77	1.27	54.32	1.77	57.48	125.56	86.82	38.74
S66	5.47	-3.41	3.41	0.62	3.93	10.01	9.39	0.62
S10x8	6.59	1.76	1.76	0.27	2.44	10.25	10.15	0.10
X40	3.76	2.14	2.14	0.57	2.65	8.43	8.14	0.29

Continued on next page

Test set	$\lvert\overline{\Delta E}\rvert$	MD	MAD	NMAD	RMSD	Δ_{err}	max	min
HEAVY28	1.24	-1.24	1.24	1.00	1.33	2.79	2.28	0.51
CHB6	26.79	1.46	1.56	0.06	2.06	4.42	4.10	0.32
AHB21	22.49	-0.05	0.96	0.04	1.16	2.47	2.40	0.07
IL16	109.04	16.23	16.23	0.15	17.73	26.57	24.80	1.77
PNICO23	4.27	-1.87	1.87	0.44	1.99	4.91	3.78	1.13
CT20	0.98	0.74	0.74	0.75	0.77	1.34	1.11	0.23
CARBHB12	6.04	-0.22	0.56	0.09	0.69	1.30	1.22	0.08
ADIM6	3.36	-4.86	4.86	1.44	5.32	10.00	8.14	1.86
3B-69-TRIM	12.30	6.21	6.21	0.50	6.88	15.77	14.79	0.98
ISOL24	21.92	-4.95	9.57	0.44	14.96	51.08	51.07	0.01
C60ISO	98.25	0.34	2.14	0.02	2.33	4.51	3.70	0.81
L7	18.20	23.56	23.56	1.29	26.48	53.50	44.22	9.28
UPU23	5.72	2.14	2.49	0.43	3.71	9.59	9.58	0.01
ENZYMES23	15.32	-2.97	5.30	0.35	6.29	15.15	15.02	0.13

Table S102: Statistical analysis for CAMB3LYP for all testset in our databsase. The numbers given (all in kcal/mol) are average reaction energy ($\lvert\overline{\Delta E}\rvert$), mean deviation (MD), mean absolute deviation (MAD), MAD normalized with respect to $\lvert\overline{\Delta E}\rvert$ (NMAD), root-mean-square deviation (RMSD), deviation span (Δ_{err}), maximum (max) and minimum deviation (min).

Test set	$\lvert\overline{\Delta E}\rvert$	MD	MAD	NMAD	RMSD	Δ_{err}	max	min
FH51	31.01	4.02	4.02	0.13	5.23	16.39	16.21	0.18
YBDE18	49.28	-9.20	9.31	0.19	10.62	19.19	18.19	1.00
AL2X6	35.88	-8.92	8.92	0.25	9.49	19.12	14.01	5.11
DARC	32.47	14.82	14.82	0.46	15.17	26.29	17.98	8.31
NBPRC	27.71	4.70	5.80	0.21	8.04	19.52	19.14	0.38
HEAVYSB9	58.02	-8.99	8.99	0.15	9.37	19.52	13.78	5.74
BSR36	16.20	-10.58	10.58	0.65	12.09	30.65	27.56	3.09
RSE43	7.60	-1.74	1.74	0.23	2.02	5.03	4.75	0.28
W4-11	306.91	-4.33	5.08	0.02	7.09	28.95	28.90	0.05
G21EA	33.62	-3.81	4.40	0.13	5.16	11.21	10.52	0.69
G21IP	257.61	0.32	3.46	0.01	4.35	10.19	10.18	0.01
DIPCS10	654.26	-1.18	4.12	0.01	5.16	9.94	9.15	0.79
PA26	189.05	1.16	1.86	0.01	2.74	8.16	8.01	0.15
SIE4x4	33.72	17.08	17.08	0.51	19.97	42.24	40.51	1.73
ALKBDE10	100.69	44.50	44.50	0.44	50.79	92.95	78.46	14.49
RC21	35.70	-0.98	2.21	0.06	3.28	10.88	10.82	0.06
ALK8	62.60	-5.87	5.87	0.09	8.78	18.54	18.29	0.25
DC13	54.98	4.72	16.07	0.29	21.23	39.30	38.81	0.49
G2RC	51.26	1.17	2.60	0.05	3.45	8.06	8.02	0.04
BH76RC	21.39	-0.60	2.38	0.11	2.98	7.20	7.01	0.19
MOR23	35.57	12.51	13.87	0.39	18.98	68.01	67.29	0.72

Continued on next page

Test set	$\lvert\overline{\Delta E}\rvert$	MD	MAD	NMAD	RMSD	Δ_{err}	max	min
WCPT18	34.99	1.35	1.35	0.04	1.71	3.64	3.60	0.04
BHROT27	6.37	0.44	0.44	0.07	0.58	1.36	1.33	0.03
BHPERI	20.87	4.44	4.44	0.21	5.16	9.57	9.32	0.25
BHDIV10	45.33	2.80	2.80	0.06	3.53	6.13	6.10	0.03
INV24	32.85	1.88	1.88	0.06	2.18	4.75	4.32	0.43
CR20	19.31	-11.58	11.58	0.60	11.73	23.82	15.87	7.95
CRBH20	46.13	-8.83	8.83	0.19	8.92	19.09	11.68	7.41
TMBH17	12.76	2.97	4.05	0.32	5.82	17.03	16.99	0.04
LTMBH26	9.98	-1.77	3.49	0.35	4.98	17.21	16.92	0.29
BH76	18.61	-4.91	5.07	0.27	5.81	11.72	11.55	0.17
ISO34	14.57	2.38	2.38	0.16	3.47	11.71	11.71	0.00
ICONF	3.27	0.58	0.58	0.18	0.78	1.88	1.84	0.04
ACONF	1.83	0.92	0.92	0.50	1.03	2.14	1.80	0.34
TAUT15	3.05	1.14	1.14	0.37	1.35	2.12	2.10	0.02
Amino20x4	2.44	0.66	0.66	0.27	0.83	2.36	2.35	0.01
PCONF	1.62	3.80	3.80	2.35	4.34	7.23	6.99	0.24
MCONF	4.97	2.50	2.50	0.50	2.79	4.22	4.14	0.08
SCONF	4.60	0.60	0.60	0.13	0.75	1.77	1.75	0.02
PArel	4.63	-0.08	1.17	0.25	1.73	5.32	5.31	0.01
BUT14DIOL	2.80	-0.09	0.33	0.12	0.40	0.86	0.85	0.01
EIE22	5.44	1.49	1.49	0.27	1.65	2.98	2.95	0.03
Styrene45	62.64	3.67	5.96	0.10	7.69	18.59	18.59	0.00
ISOMERIZATION20	31.84	0.21	2.18	0.07	2.91	7.53	7.50	0.03
DIE60	4.71	1.32	1.34	0.28	1.49	2.85	2.85	0.00
IDISP	14.22	4.12	16.36	1.15	18.94	38.44	34.72	3.72
C20C24	30.77	1.30	54.49	1.77	57.65	125.91	87.04	38.87
S66	5.47	-3.37	3.37	0.62	3.91	10.01	9.39	0.62
S10x8	6.59	1.76	1.76	0.27	2.44	10.25	10.15	0.10
X40	3.76	2.14	2.14	0.57	2.65	8.43	8.14	0.29
HEAVY28	1.24	-1.24	1.24	1.00	1.33	2.79	2.28	0.51
CHB6	26.79	1.46	1.56	0.06	2.06	4.42	4.10	0.32
AHB21	22.49	-0.05	0.96	0.04	1.16	2.47	2.40	0.07
IL16	109.04	3.22	3.22	0.03	3.38	7.02	5.51	1.51
PNICO23	4.27	-1.87	1.87	0.44	1.99	4.91	3.78	1.13
CT20	0.98	0.74	0.74	0.75	0.77	1.34	1.11	0.23
CARBHB12	6.04	-0.22	0.56	0.09	0.69	1.30	1.22	0.08
ADIM6	3.36	-4.86	4.86	1.44	5.32	10.00	8.14	1.86
3B-69-TRIM	12.30	6.21	6.21	0.50	6.88	15.77	14.79	0.98
ISOL24	21.92	-4.95	9.57	0.44	14.96	51.08	51.07	0.01
C60ISO	98.25	0.34	2.14	0.02	2.33	4.51	3.70	0.81
L7	18.20	23.56	23.56	1.29	26.48	53.50	44.22	9.28
UPU23	5.72	2.15	2.48	0.43	3.66	9.59	9.58	0.01
ENZYMES23	15.32	-2.74	5.20	0.34	6.21	15.15	15.02	0.13

Table S103: Statistical analysis for B97 for all testset in our databsase. The numbers given (all in kcal/mol) are average reaction energy ($|\overline{\Delta E}|$), mean deviation (MD), mean absolute deviation (MAD), MAD normalized with respect to $|\overline{\Delta E}|$ (NMAD), root-mean-square deviation (RMSD), deviation span (Δ_{err}), maximum (max) and minimum deviation (min).

Test set	$\lvert\overline{\Delta E}\rvert$	MD	MAD	NMAD	RMSD	Δ_{err}	max	min
FH51	31.01	1.82	2.99	0.10	3.85	10.00	9.85	0.15
YBDE18	49.28	-4.54	6.06	0.12	7.65	15.43	14.90	0.53
AL2X6	35.88	-6.64	6.64	0.19	7.09	12.48	9.56	2.92
DARC	32.47	8.45	8.45	0.26	9.04	13.92	11.57	2.35
NBPRC	27.71	3.64	4.05	0.15	5.49	12.96	12.38	0.58
HEAVYSB9	58.02	-5.61	5.61	0.10	6.12	12.02	9.45	2.57
BSR36	16.20	-10.29	10.29	0.64	11.78	29.68	26.93	2.75
RSE43	7.60	-2.05	2.05	0.27	2.30	5.74	5.24	0.50
W4-11	306.91	-2.77	3.62	0.01	5.06	26.26	26.15	0.11
G21EA	33.62	-3.95	4.26	0.13	5.17	10.36	10.23	0.13
G21IP	257.61	0.41	2.80	0.01	3.50	8.13	8.12	0.01
DIPCS10	654.26	-1.84	3.24	0.00	4.25	9.15	8.62	0.53
PA26	189.05	2.76	2.83	0.01	3.66	9.85	9.62	0.23
SIE4x4	33.72	16.58	16.58	0.49	18.97	40.04	37.31	2.73
ALKBDE10	100.69	-1.49	3.90	0.04	5.05	9.60	9.43	0.17
RC21	35.70	1.05	2.02	0.06	2.61	5.29	5.29	0.00
ALK8	62.60	-2.30	2.90	0.05	3.76	6.86	6.82	0.04
DC13	54.98	2.28	10.72	0.19	13.93	30.04	29.62	0.42
G2RC	51.26	0.27	3.14	0.06	3.95	9.02	8.83	0.19
BH76RC	21.39	-0.13	1.58	0.07	2.15	5.39	5.32	0.07
MOR23	35.57	11.23	12.01	0.34	17.36	66.08	65.63	0.45
WCPT18	34.99	-2.06	2.07	0.06	2.47	5.21	5.15	0.06
BHROT27	6.37	0.24	0.38	0.06	0.49	1.11	1.11	0.00
BHPERI	20.87	1.61	2.71	0.13	3.03	6.33	6.06	0.27
BHDIV10	45.33	-2.11	3.19	0.07	3.57	6.23	6.15	0.08
INV24	32.85	-1.61	1.83	0.06	2.15	4.72	4.72	0.00
CR20	19.31	-7.38	7.38	0.38	7.54	15.43	10.75	4.68
CRBH20	46.13	-5.99	5.99	0.13	6.14	13.00	8.61	4.39
TMBH17	12.76	2.66	3.58	0.28	4.56	11.02	10.58	0.44
LTMBH26	9.98	-1.61	3.06	0.31	4.51	15.89	15.85	0.04
BH76	18.61	-4.06	4.30	0.23	4.84	10.43	10.37	0.06
ISO34	14.57	-0.52	1.59	0.11	2.47	8.87	8.78	0.09
ICONF	3.27	0.13	0.50	0.15	0.71	1.85	1.80	0.05
ACONF	1.83	0.82	0.82	0.45	0.92	1.91	1.62	0.29
TAUT15	3.05	-0.14	0.99	0.32	1.12	1.82	1.73	0.09
Amino20x4	2.44	-0.15	0.61	0.25	0.75	2.00	2.00	0.00
PCONF	1.62	-0.48	3.44	2.13	3.90	6.74	6.42	0.32
MCONF	4.97	-2.05	2.11	0.42	2.39	3.67	3.63	0.04

Continued on next page

Test set	$\|\overline{\Delta E}\|$	MD	MAD	NMAD	RMSD	Δ_{err}	max	min
SCONF	4.60	-0.46	0.85	0.18	1.01	1.98	1.96	0.02
PArel	4.63	0.13	1.00	0.22	1.55	5.07	4.96	0.11
BUT14DIOL	2.80	-0.37	0.51	0.18	0.56	1.04	1.01	0.03
EIE22	5.44	1.37	1.37	0.25	1.54	2.82	2.81	0.01
Styrene45	62.64	1.53	3.28	0.05	4.11	10.11	10.11	0.00
ISOMERIZATION20	31.84	0.36	1.94	0.06	2.50	5.55	5.49	0.06
DIE60	4.71	1.29	1.30	0.28	1.45	2.76	2.72	0.04
IDISP	14.22	3.14	14.15	1.00	15.88	30.02	26.78	3.24
C20C24	30.77	-5.50	33.30	1.08	37.96	81.50	64.27	17.23
S66	5.47	-2.93	2.93	0.54	3.35	8.94	8.44	0.50
S10x8	6.59	1.60	1.60	0.24	2.21	9.10	9.04	0.06
X40	3.76	1.77	1.77	0.47	2.21	7.39	7.09	0.30
HEAVY28	1.24	-0.74	0.74	0.60	0.83	1.52	1.52	0.00
CHB6	26.79	1.69	1.69	0.06	1.98	4.30	3.65	0.65
AHB21	22.49	-0.16	0.98	0.04	1.21	2.30	2.29	0.01
IL16	109.04	2.84	2.84	0.03	3.06	6.17	5.32	0.85
PNICO23	4.27	-1.22	1.22	0.29	1.33	3.22	2.82	0.40
CT20	0.98	0.54	0.54	0.55	0.58	1.11	0.86	0.25
CARBHB12	6.04	0.13	0.51	0.08	0.66	1.68	1.58	0.10
ADIM6	3.36	-3.81	3.81	1.13	4.24	7.94	6.69	1.25
3B-69-TRIM	12.30	5.29	5.29	0.43	5.84	13.68	12.61	1.07
ISOL24	21.92	-3.56	6.89	0.31	11.19	39.68	39.59	0.09
C60ISO	98.25	-0.95	2.12	0.02	2.80	6.18	5.76	0.42
L7	18.20	21.16	21.16	1.16	23.98	48.63	40.68	7.95
UPU23	5.72	1.70	2.05	0.36	2.99	7.71	7.64	0.07
ENZYMES23	15.32	-2.43	4.93	0.32	6.13	16.33	16.27	0.06

Table S104: Statistical analysis for WB97X for all testset in our databsase. The numbers given (all in kcal/mol) are average reaction energy ($|\overline{\Delta E}|$), mean deviation (MD), mean absolute deviation (MAD), MAD normalized with respect to $|\overline{\Delta E}|$ (NMAD), root-mean-square deviation (RMSD), deviation span (Δ_{err}), maximum (max) and minimum deviation (min).

Test set	$\|\overline{\Delta E}\|$	MD	MAD	NMAD	RMSD	Δ_{err}	max	min
FH51	31.01	-1.03	2.32	0.07	3.05	10.17	10.13	0.04
YBDE18	49.28	37.84	37.84	0.77	51.68	75.90	74.66	1.24
AL2X6	35.88	2.00	2.00	0.06	2.18	4.95	3.65	1.30
DARC	32.47	-8.48	8.48	0.26	8.71	19.00	12.27	6.73
NBPRC	27.71	-1.73	4.43	0.16	4.57	9.88	6.96	2.92
HEAVYSB9	58.02	-2.51	3.49	0.06	3.69	7.17	5.47	1.70
BSR36	16.20	-5.95	5.95	0.37	7.30	20.80	18.99	1.81
RSE43	7.60	-0.41	1.79	0.24	4.69	30.04	29.67	0.37
W4-11	306.91	-3.96	4.10	0.01	6.52	37.84	37.81	0.03

Continued on next page

Test set	$\lvert\overline{\Delta E}\rvert$	MD	MAD	NMAD	RMSD	Δ_{err}	max	min
G21EA	33.62	-3.97	4.20	0.12	5.16	10.21	9.83	0.38
G21IP	257.61	0.22	2.93	0.01	3.86	10.58	10.51	0.07
DIPCS10	654.26	-3.71	5.26	0.01	7.15	17.45	16.72	0.73
PA26	189.05	1.78	1.87	0.01	2.63	6.27	6.22	0.05
SIE4x4	33.72	11.00	11.00	0.33	13.29	32.05	29.89	2.16
ALKBDE10	100.69	-2.50	4.66	0.05	6.45	14.14	14.00	0.14
RC21	35.70	2.26	2.81	0.08	3.19	5.78	5.19	0.59
ALK8	62.60	-2.06	2.45	0.04	3.07	6.63	6.05	0.58
DC13	54.98	-1.71	7.59	0.14	10.08	21.52	21.26	0.26
G2RC	51.26	-2.33	4.10	0.08	4.87	9.97	9.76	0.21
BH76RC	21.39	-0.10	1.79	0.08	2.17	4.86	4.85	0.01
MOR23	35.57	7.35	8.21	0.23	15.39	68.61	67.73	0.88
WCPT18	34.99	0.08	1.81	0.05	2.41	7.29	7.17	0.12
BHROT27	6.37	0.25	0.36	0.06	0.53	1.28	1.28	0.00
BHPERI	20.87	4.11	4.20	0.20	4.66	10.72	9.63	1.09
BHDIV10	45.33	1.29	1.34	0.03	1.78	3.45	3.44	0.01
INV24	32.85	-0.57	1.36	0.04	2.13	7.23	7.15	0.08
CR20	19.31	6.29	6.29	0.33	6.33	12.78	7.83	4.95
CRBH20	46.13	5.53	5.53	0.12	5.64	11.69	7.42	4.27
TMBH17	12.76	5.15	5.53	0.43	6.82	15.15	13.94	1.21
LTMBH26	9.98	1.01	2.14	0.21	3.83	17.34	17.26	0.08
BH76	18.61	-0.76	2.21	0.12	2.59	6.08	5.94	0.14
ISO34	14.57	-0.59	1.06	0.07	1.44	3.58	3.56	0.02
ICONF	3.27	0.22	0.40	0.12	0.55	1.17	1.14	0.03
ACONF	1.83	0.09	0.09	0.05	0.11	0.24	0.24	0.00
TAUT15	3.05	-0.32	1.09	0.36	1.26	2.46	2.44	0.02
Amino20x4	2.44	-0.02	0.23	0.09	0.30	0.86	0.86	0.00
PCONF	1.62	0.17	0.75	0.47	0.90	1.98	1.74	0.24
MCONF	4.97	-0.23	0.36	0.07	0.42	0.84	0.83	0.01
SCONF	4.60	-0.08	0.15	0.03	0.19	0.56	0.54	0.02
PArel	4.63	0.08	0.64	0.14	0.84	1.71	1.69	0.02
BUT14DIOL	2.80	0.26	0.26	0.09	0.28	0.56	0.54	0.02
EIE22	5.44	0.17	0.40	0.07	0.45	0.89	0.86	0.03
Styrene45	62.64	-0.19	2.02	0.03	2.82	8.93	8.93	0.00
ISOMERIZATION20	31.84	1.73	2.83	0.09	6.05	25.84	25.72	0.12
DIE60	4.71	0.09	0.65	0.14	0.91	2.19	2.19	0.00
IDISP	14.22	0.05	4.17	0.29	5.28	10.96	10.32	0.64
C20C24	30.77	-1.87	6.33	0.21	8.73	18.72	18.68	0.04
S66	5.47	-0.56	0.56	0.10	0.74	2.45	2.45	0.00
S10x8	6.59	0.04	0.39	0.06	0.64	3.01	3.01	0.00
X40	3.76	0.11	0.37	0.10	0.58	2.38	2.36	0.02
HEAVY28	1.24	1.13	1.13	0.91	1.19	2.20	1.90	0.30
CHB6	26.79	1.74	1.74	0.06	1.95	3.47	3.04	0.43
AHB21	22.49	-1.62	1.62	0.07	1.90	4.48	3.99	0.49

Continued on next page

Test set	$\lvert\overline{\Delta E}\rvert$	MD	MAD	NMAD	RMSD	Δ_{err}	max	min
IL16	109.04	0.57	0.61	0.01	0.76	1.56	1.54	0.02
PNICO23	4.27	0.10	0.39	0.09	0.49	1.67	1.59	0.08
CT20	0.98	0.13	0.18	0.19	0.23	0.51	0.49	0.02
CARBHB12	6.04	1.03	1.03	0.17	1.13	2.53	2.06	0.47
ADIM6	3.36	-0.45	0.45	0.13	0.55	0.94	0.93	0.01
3B-69-TRIM	12.30	0.94	1.23	0.10	1.61	5.14	5.09	0.05
ISOL24	21.92	-1.25	3.17	0.14	4.35	12.91	12.88	0.03
C60ISO	98.25	15.73	15.73	0.16	17.32	30.85	25.67	5.18
L7	18.20	9.11	9.11	0.50	10.93	21.72	19.14	2.58
UPU23	5.72	1.25	1.26	0.22	1.56	3.09	3.04	0.05
ENZYMES23	15.32	0.97	2.79	0.18	4.15	13.74	13.66	0.08

Table S105: Statistical analysis for PBE0 for all testset in our databsase. The numbers given (all in kcal/mol) are average reaction energy ($\lvert\overline{\Delta E}\rvert$), mean deviation (MD), mean absolute deviation (MAD), MAD normalized with respect to $\lvert\overline{\Delta E}\rvert$ (NMAD), root-mean-square deviation (RMSD), deviation span (Δ_{err}), maximum (max) and minimum deviation (min).

Test set	$\lvert\overline{\Delta E}\rvert$	MD	MAD	NMAD	RMSD	Δ_{err}	max	min
FH51	31.01	2.55	2.55	0.08	3.33	8.18	8.16	0.02
YBDE18	49.28	-3.27	3.27	0.07	3.78	8.51	7.92	0.59
AL2X6	35.88	-2.76	2.86	0.08	3.33	4.78	4.54	0.24
DARC	32.47	-0.18	3.23	0.10	3.57	7.06	6.56	0.50
NBPRC	27.71	1.21	2.48	0.09	3.00	6.80	6.54	0.26
HEAVYSB9	58.02	-4.76	4.76	0.08	5.13	9.18	7.63	1.55
BSR36	16.20	-7.64	7.64	0.47	8.72	22.20	19.75	2.45
RSE43	7.60	-1.36	1.36	0.18	1.60	4.13	4.07	0.06
W4-11	306.91	-3.11	3.91	0.01	5.63	27.47	27.45	0.02
G21EA	33.62	-3.98	4.62	0.14	6.00	12.01	12.01	0.00
G21IP	257.61	0.61	3.60	0.01	4.28	8.92	8.85	0.07
DIPCS10	654.26	0.02	2.80	0.00	3.05	5.18	4.72	0.46
PA26	189.05	1.24	1.69	0.01	2.41	7.08	7.07	0.01
SIE4x4	33.72	13.54	13.54	0.40	15.64	33.50	31.77	1.73
ALKBDE10	100.69	42.40	42.40	0.42	48.70	91.39	74.67	16.72
RC21	35.70	3.54	4.04	0.11	4.76	10.85	10.35	0.50
ALK8	62.60	1.57	1.77	0.03	2.79	6.59	6.54	0.05
DC13	54.98	-0.65	9.09	0.17	11.28	22.10	21.82	0.28
G2RC	51.26	-2.86	6.01	0.12	7.53	17.81	17.79	0.02
BH76RC	21.39	-0.36	2.02	0.09	2.71	6.88	6.68	0.20
MOR23	35.57	6.83	7.68	0.22	14.46	63.18	62.79	0.39
WCPT18	34.99	3.88	3.88	0.11	4.39	9.43	8.50	0.93
BHROT27	6.37	0.56	0.56	0.09	0.74	1.47	1.47	0.00
BHPERI	20.87	1.26	1.26	0.06	1.59	3.90	3.90	0.00

Continued on next page

Test set	$\lvert\overline{\Delta E}\rvert$	MD	MAD	NMAD	RMSD	Δ_{err}	max	min
BHDIV10	45.33	4.08	4.08	0.09	4.58	6.91	6.81	0.10
INV24	32.85	1.56	1.56	0.05	1.95	5.24	4.93	0.31
CR20	19.31	-0.75	1.17	0.06	1.56	3.54	3.47	0.07
CRBH20	46.13	0.72	1.11	0.02	1.30	2.56	2.45	0.11
TMBH17	12.76	1.90	3.66	0.29	4.37	7.18	7.09	0.09
LTMBH26	9.98	-0.97	2.44	0.24	3.78	14.77	14.72	0.05
BH76	18.61	-10.70	11.03	0.59	18.30	60.54	60.48	0.06
ISO34	14.57	1.60	1.60	0.11	2.14	6.50	6.48	0.02
ICONF	3.27	0.45	0.45	0.14	0.60	1.48	1.40	0.08
ACONF	1.83	0.60	0.60	0.33	0.67	1.34	1.12	0.22
TAUT15	3.05	1.15	1.15	0.38	1.54	3.29	3.26	0.03
Amino20x4	2.44	0.47	0.47	0.19	0.59	1.78	1.78	0.00
PCONF	1.62	3.00	3.00	1.85	3.36	5.25	5.21	0.04
MCONF	4.97	1.63	1.63	0.33	1.83	2.99	2.98	0.01
SCONF	4.60	0.28	0.28	0.06	0.36	0.90	0.89	0.01
PArel	4.63	0.61	1.23	0.26	1.71	5.22	5.18	0.04
BUT14DIOL	2.80	0.02	0.21	0.07	0.27	0.72	0.72	0.00
EIE22	5.44	1.33	1.35	0.25	1.53	2.86	2.77	0.09
Styrene45	62.64	0.32	2.56	0.04	3.16	7.83	7.83	0.00
ISOMERIZATION20	31.84	0.13	1.92	0.06	2.51	5.73	5.69	0.04
DIE60	4.71	1.46	1.47	0.31	1.68	3.26	3.25	0.01
IDISP	14.22	9.47	9.47	0.67	10.29	17.13	14.63	2.50
C20C24	30.77	-11.33	12.98	0.42	20.12	39.60	38.94	0.66
S66	5.47	-2.19	2.19	0.40	2.67	6.91	6.87	0.04
S10x8	6.59	0.94	0.97	0.15	1.52	6.75	6.75	0.00
X40	3.76	1.32	1.32	0.35	1.74	5.98	5.93	0.05
HEAVY28	1.24	-0.53	0.58	0.47	0.66	1.34	1.31	0.03
CHB6	26.79	0.63	0.94	0.03	1.17	2.33	2.30	0.03
AHB21	22.49	-1.47	1.66	0.07	2.20	4.99	4.86	0.13
IL16	109.04	1.15	1.37	0.01	1.57	3.61	3.34	0.27
PNICO23	4.27	-0.39	0.62	0.15	0.83	2.40	2.36	0.04
CT20	0.98	0.42	0.43	0.44	0.46	0.79	0.73	0.06
CARBHB12	6.04	0.87	0.88	0.15	1.20	2.75	2.69	0.06
ADIM6	3.36	-3.27	3.27	0.97	3.63	6.74	5.65	1.09
3B-69-TRIM	12.30	4.10	4.11	0.33	4.77	11.01	10.98	0.03
ISOL24	21.92	-1.15	3.82	0.17	5.75	19.18	18.96	0.22
C60ISO	98.25	2.09	2.75	0.03	2.93	6.27	4.84	1.43
L7	18.20	18.49	18.49	1.02	21.00	42.61	35.44	7.17
UPU23	5.72	1.60	1.85	0.32	2.72	7.09	7.00	0.09
ENZYMES23	15.32	-0.87	2.89	0.19	3.94	13.11	12.64	0.47

Table S106: Statistical analysis for TPSSH for all testset in our databsase. The numbers given (all in kcal/mol) are average reaction energy ($|\overline{\Delta E}|$), mean deviation (MD), mean absolute deviation (MAD), MAD normalized with respect to $|\overline{\Delta E}|$ (NMAD), root-mean-square deviation (RMSD), deviation span (Δ_{err}), maximum (max) and minimum deviation (min).

Test set	$\lvert\overline{\Delta E}\rvert$	MD	MAD	NMAD	RMSD	Δ_{err}	max	min
FH51	31.01	3.99	3.99	0.13	5.09	12.20	12.12	0.08
YBDE18	49.28	-7.76	7.76	0.16	8.26	14.32	12.13	2.19
AL2X6	35.88	-3.52	3.61	0.10	4.31	6.12	5.91	0.21
DARC	32.47	8.27	8.27	0.25	8.89	13.97	11.37	2.60
NBPRC	27.71	1.76	2.92	0.11	3.94	7.86	7.82	0.04
HEAVYSB9	58.02	-5.49	5.49	0.09	6.17	11.99	10.66	1.33
BSR36	16.20	-9.83	9.83	0.61	11.07	27.63	25.29	2.34
RSE43	7.60	-1.51	1.51	0.20	1.78	5.24	4.87	0.37
W4-11	306.91	-3.27	5.20	0.02	6.95	27.48	27.41	0.07
G21EA	33.62	-4.39	4.88	0.15	6.38	12.69	12.61	0.08
G21IP	257.61	-0.07	3.69	0.01	4.45	11.40	11.05	0.35
DIPCS10	654.26	-1.50	2.97	0.00	3.74	8.76	7.98	0.78
PA26	189.05	3.17	3.17	0.02	4.01	10.73	10.42	0.31
SIE4x4	33.72	17.47	17.47	0.52	20.35	43.13	40.64	2.49
ALKBDE10	100.69	-3.22	6.01	0.06	7.24	13.83	13.17	0.66
RC21	35.70	1.38	2.51	0.07	2.97	6.19	5.83	0.36
ALK8	62.60	-0.01	0.98	0.02	1.12	2.28	2.14	0.14
DC13	54.98	1.63	11.46	0.21	14.76	31.80	31.60	0.20
G2RC	51.26	1.83	5.25	0.10	6.74	16.25	16.24	0.01
BH76RC	21.39	-0.25	3.10	0.14	4.11	9.05	8.92	0.13
MOR23	35.57	7.32	8.57	0.24	14.93	63.26	62.97	0.29
WCPT18	34.99	3.84	3.84	0.11	4.14	8.10	6.83	1.27
BHROT27	6.37	0.54	0.54	0.09	0.70	1.52	1.50	0.02
BHPERI	20.87	1.44	1.44	0.07	1.80	4.32	4.27	0.05
BHDIV10	45.33	4.53	4.53	0.10	4.96	8.92	7.39	1.53
INV24	32.85	2.10	2.10	0.06	2.43	6.00	5.49	0.51
CR20	19.31	-4.90	4.90	0.25	5.28	8.64	8.58	0.06
CRBH20	46.13	-6.66	6.66	0.14	6.73	13.66	8.62	5.04
TMBH17	12.76	0.57	3.07	0.24	4.03	8.80	8.77	0.03
LTMBH26	9.98	-2.22	3.48	0.35	4.81	16.12	16.09	0.03
BH76	18.61	-6.85	6.92	0.37	7.77	16.46	16.45	0.01
ISO34	14.57	2.28	2.28	0.16	3.17	10.34	10.27	0.07
ICONF	3.27	0.37	0.37	0.11	0.48	1.31	1.30	0.01
ACONF	1.83	0.69	0.69	0.38	0.78	1.54	1.32	0.22
TAUT15	3.05	1.35	1.35	0.44	1.58	2.64	2.56	0.08
Amino20x4	2.44	0.52	0.52	0.21	0.66	1.90	1.90	0.00
PCONF	1.62	3.71	3.71	2.29	4.09	6.39	5.96	0.43
MCONF	4.97	2.08	2.08	0.42	2.33	3.74	3.71	0.03

Continued on next page

Test set	$\lvert\overline{\Delta E}\rvert$	MD	MAD	NMAD	RMSD	Δ_{err}	max	min
SCONF	4.60	0.32	0.32	0.07	0.41	0.87	0.85	0.02
PArel	4.63	0.23	1.33	0.29	1.80	4.97	4.89	0.08
BUT14DIOL	2.80	-0.18	0.32	0.11	0.36	0.71	0.69	0.02
EIE22	5.44	1.83	1.83	0.34	2.00	3.52	3.49	0.03
Styrene45	62.64	0.26	2.85	0.05	3.60	11.16	11.16	0.00
ISOMERIZATION20	31.84	1.17	2.89	0.09	3.72	8.50	8.48	0.02
DIE60	4.71	1.75	1.75	0.37	1.90	3.52	3.30	0.22
IDISP	14.22	12.24	12.24	0.86	14.16	28.84	25.94	2.90
C20C24	30.77	-11.93	29.11	0.95	37.52	78.59	70.07	8.52
S66	5.47	-3.13	3.13	0.57	3.62	9.49	8.96	0.53
S10x8	6.59	1.55	1.55	0.24	2.12	8.31	8.23	0.08
X40	3.76	1.87	1.87	0.50	2.33	7.47	7.46	0.01
HEAVY28	1.24	-0.84	0.84	0.68	0.94	1.72	1.65	0.07
CHB6	26.79	1.51	1.51	0.06	1.87	3.79	3.54	0.25
AHB21	22.49	-0.48	1.15	0.05	1.36	2.73	2.55	0.18
IL16	109.04	2.30	2.30	0.02	2.50	5.00	4.49	0.51
PNICO23	4.27	-0.81	0.92	0.22	1.09	2.98	2.97	0.01
CT20	0.98	0.69	0.69	0.70	0.72	1.30	1.05	0.25
CARBHB12	6.04	0.35	0.59	0.10	0.87	2.36	2.25	0.11
ADIM6	3.36	-4.40	4.40	1.31	4.87	9.09	7.55	1.54
3B-69-TRIM	12.30	5.86	5.86	0.48	6.50	14.49	13.63	0.86
ISOL24	21.92	-4.14	7.29	0.33	11.27	38.67	38.60	0.07
C60ISO	98.25	-3.55	3.78	0.04	5.29	10.62	10.62	0.00
L7	18.20	22.15	22.15	1.22	24.81	50.03	41.47	8.56
UPU23	5.72	1.83	2.23	0.39	3.35	9.34	9.32	0.02
ENZYMES23	15.32	-3.34	5.60	0.37	6.55	14.76	14.05	0.71

Table S107: Statistical analysis for PW6B95 for all testset in our databsase. The numbers given (all in kcal/mol) are average reaction energy ($\lvert\overline{\Delta E}\rvert$), mean deviation (MD), mean absolute deviation (MAD), MAD normalized with respect to $\lvert\overline{\Delta E}\rvert$ (NMAD), root-mean-square deviation (RMSD), deviation span (Δ_{err}), maximum (max) and minimum deviation (min).

Test set	$\lvert\overline{\Delta E}\rvert$	MD	MAD	NMAD	RMSD	Δ_{err}	max	min
FH51	31.01	0.99	2.08	0.07	2.70	6.81	6.80	0.01
YBDE18	49.28	33.14	34.32	0.70	47.72	69.48	69.30	0.18
AL2X6	35.88	2.98	2.98	0.08	3.04	5.41	3.59	1.82
DARC	32.47	-2.38	2.49	0.08	3.56	6.93	6.78	0.15
NBPRC	27.71	-1.61	2.77	0.10	3.13	4.93	4.77	0.16
HEAVYSB9	58.02	-4.21	4.21	0.07	4.64	9.45	7.98	1.47
BSR36	16.20	-5.93	5.93	0.37	6.65	16.60	15.39	1.21
RSE43	7.60	-1.98	1.98	0.26	2.27	5.52	5.03	0.49
W4-11	306.91	-2.53	3.04	0.01	5.07	31.67	31.64	0.03

Continued on next page

Test set	$\|\overline{\Delta E}\|$	MD	MAD	NMAD	RMSD	Δ_{err}	max	min
G21EA	33.62	-3.88	4.14	0.12	5.12	10.83	10.21	0.62
G21IP	257.61	1.38	2.81	0.01	3.64	9.23	9.17	0.06
DIPCS10	654.26	1.36	2.82	0.00	3.30	5.51	5.36	0.15
PA26	189.05	1.31	1.69	0.01	2.42	7.24	7.22	0.02
SIE4x4	33.72	14.67	14.67	0.44	17.05	35.88	34.61	1.27
ALKBDE10	100.69	-2.29	4.16	0.04	5.33	12.36	11.58	0.78
RC21	35.70	1.47	2.10	0.06	2.59	6.19	6.01	0.18
ALK8	62.60	1.54	2.12	0.03	2.71	4.87	4.76	0.11
DC13	54.98	0.51	9.22	0.17	11.44	21.23	20.80	0.43
G2RC	51.26	-0.55	2.79	0.05	3.38	7.62	7.40	0.22
BH76RC	21.39	-0.16	1.49	0.07	2.02	4.94	4.91	0.03
MOR23	35.57	7.94	8.33	0.23	15.17	67.52	65.95	1.57
WCPT18	34.99	-1.31	1.37	0.04	1.67	3.71	3.58	0.13
BHROT27	6.37	0.44	0.54	0.09	0.71	1.42	1.40	0.02
BHPERI	20.87	1.31	1.83	0.09	2.07	3.57	3.46	0.11
BHDIV10	45.33	-1.85	2.17	0.05	2.40	4.29	3.81	0.48
INV24	32.85	1.53	1.53	0.05	1.93	4.10	4.05	0.05
CR20	19.31	1.14	1.41	0.07	1.67	3.62	3.56	0.06
CRBH20	46.13	-0.09	0.57	0.01	0.70	1.30	1.25	0.05
TMBH17	12.76	2.57	3.07	0.24	4.41	10.52	10.49	0.03
LTMBH26	9.98	-0.72	2.41	0.24	3.95	15.81	15.75	0.06
BH76	18.61	-3.48	3.65	0.20	4.13	8.90	8.87	0.03
ISO34	14.57	-0.69	1.40	0.10	1.81	4.48	4.48	0.00
ICONF	3.27	0.12	0.27	0.08	0.39	1.02	1.01	0.01
ACONF	1.83	0.22	0.22	0.12	0.23	0.46	0.38	0.08
TAUT15	3.05	0.24	0.85	0.28	1.13	2.30	2.27	0.03
Amino20x4	2.44	0.01	0.37	0.15	0.47	1.23	1.23	0.00
PCONF	1.62	0.29	1.42	0.88	1.72	3.21	3.18	0.03
MCONF	4.97	-0.88	0.96	0.19	1.12	2.07	2.07	0.00
SCONF	4.60	-0.02	0.18	0.04	0.21	0.37	0.37	0.00
PArel	4.63	0.45	0.92	0.20	1.45	4.97	4.96	0.01
BUT14DIOL	2.80	-0.42	0.42	0.15	0.43	0.66	0.59	0.07
EIE22	5.44	1.22	1.23	0.23	1.38	2.47	2.43	0.04
Styrene45	62.64	0.39	2.30	0.04	2.76	7.55	7.55	0.00
ISOMERIZATION20	31.84	2.25	4.29	0.13	9.75	41.95	41.83	0.12
DIE60	4.71	1.29	1.30	0.28	1.47	2.67	2.64	0.03
IDISP	14.22	2.13	7.15	0.50	9.08	19.60	18.40	1.20
C20C24	30.77	-11.79	11.79	0.38	16.33	34.07	31.65	2.42
S66	5.47	-1.80	1.80	0.33	2.00	5.47	5.09	0.38
S10x8	6.59	2.71	2.71	0.41	10.94	84.86	84.84	0.02
X40	3.76	1.14	1.14	0.30	1.42	4.31	4.21	0.10
HEAVY28	1.24	0.13	0.17	0.14	0.24	0.77	0.77	0.00
CHB6	26.79	0.19	0.86	0.03	1.08	1.99	1.82	0.17
AHB21	22.49	-0.57	1.03	0.05	1.31	3.53	3.38	0.15

Continued on next page

Test set	$\|\overline{\Delta E}\|$	MD	MAD	NMAD	RMSD	Δ_{err}	max	min
IL16	109.04	1.76	1.76	0.02	1.93	3.30	3.16	0.14
PNICO23	4.27	-0.75	0.75	0.18	0.85	2.63	2.34	0.29
CT20	0.98	0.34	0.34	0.34	0.37	0.69	0.61	0.08
CARBHB12	6.04	0.10	0.39	0.06	0.50	1.22	1.17	0.05
ADIM6	3.36	-1.90	1.90	0.57	2.14	4.05	3.51	0.54
3B-69-TRIM	12.30	3.15	5.44	0.44	10.54	72.70	71.91	0.79
ISOL24	21.92	-2.68	5.07	0.23	7.25	22.97	22.91	0.06
C60ISO	98.25	1.76	2.40	0.02	2.54	5.34	3.99	1.35
L7	18.20	15.03	15.03	0.83	16.43	30.79	25.38	5.41
UPU23	5.72	1.30	1.45	0.25	2.05	4.88	4.81	0.07
ENZYMES23	15.32	-3.18	3.46	0.23	4.06	6.98	6.26	0.72

Table S108: Statistical analysis for mPWB1K for all testset in our databsase. The numbers given (all in kcal/mol) are average reaction energy ($|\overline{\Delta E}|$), mean deviation (MD), mean absolute deviation (MAD), MAD normalized with respect to $|\overline{\Delta E}|$ (NMAD), root-mean-square deviation (RMSD), deviation span (Δ_{err}), maximum (max) and minimum deviation (min).

Test set	$\|\overline{\Delta E}\|$	MD	MAD	NMAD	RMSD	Δ_{err}	max	min
FH51	31.01	-1.16	2.03	0.07	2.51	5.94	5.93	0.01
YBDE18	49.28	20.89	21.28	0.43	29.20	44.21	43.98	0.23
AL2X6	35.88	2.96	2.96	0.08	3.02	5.67	3.71	1.96
DARC	32.47	-1.56	2.25	0.07	2.90	6.28	5.71	0.57
NBPRC	27.71	-1.73	3.53	0.13	4.08	7.74	7.21	0.53
HEAVYSB9	58.02	-3.61	3.61	0.06	3.83	7.05	5.34	1.71
BSR36	16.20	-5.10	5.10	0.31	5.61	13.68	12.61	1.07
RSE43	7.60	-1.30	1.30	0.17	1.54	3.85	3.68	0.17
W4-11	306.91	-8.73	8.85	0.03	11.29	38.20	38.20	0.00
G21EA	33.62	-6.47	6.47	0.19	7.84	14.68	14.65	0.03
G21IP	257.61	-0.42	2.89	0.01	3.72	9.20	9.12	0.08
DIPCS10	654.26	-2.23	3.47	0.01	4.55	9.11	9.08	0.03
PA26	189.05	1.22	1.82	0.01	2.49	6.63	6.58	0.05
SIE4x4	33.72	8.98	8.98	0.27	10.96	25.24	24.73	0.51
ALKBDE10	100.69	45.73	45.73	0.45	54.77	107.61	99.43	8.18
RC21	35.70	2.81	3.15	0.09	4.03	11.49	11.22	0.27
ALK8	62.60	2.88	3.25	0.05	4.20	7.33	6.97	0.36
DC13	54.98	-2.21	9.47	0.17	11.89	24.73	24.60	0.13
G2RC	51.26	-3.65	5.20	0.10	6.37	14.47	14.22	0.25
BH76RC	21.39	-0.09	2.54	0.12	2.88	6.58	6.24	0.34
MOR23	35.57	6.91	7.24	0.20	15.08	67.15	67.09	0.06
WCPT18	34.99	0.34	0.81	0.02	1.22	4.26	4.24	0.02
BHROT27	6.37	0.63	0.68	0.11	0.93	1.98	1.94	0.04
BHPERI	20.87	2.45	2.66	0.13	3.14	6.84	6.82	0.02

Continued on next page

Test set	$\vert\overline{\Delta E}\vert$	MD	MAD	NMAD	RMSD	Δ_{err}	max	min
BHDIV10	45.33	-0.48	1.24	0.03	1.42	3.48	2.90	0.58
INV24	32.85	-0.10	2.02	0.06	3.07	10.22	10.10	0.12
CR20	19.31	3.04	3.04	0.16	3.32	5.68	5.28	0.40
CRBH20	46.13	5.42	5.42	0.12	5.51	10.42	6.94	3.48
TMBH17	12.76	4.05	4.70	0.37	6.16	13.17	13.12	0.05
LTMBH26	9.98	0.84	1.82	0.18	3.47	16.14	16.04	0.10
BH76	18.61	-0.74	1.49	0.08	1.99	6.65	6.63	0.02
ISO34	14.57	-0.46	1.62	0.11	2.06	4.20	4.20	0.00
ICONF	3.27	0.23	0.40	0.12	0.57	1.43	1.38	0.05
ACONF	1.83	0.22	0.22	0.12	0.24	0.54	0.45	0.09
TAUT15	3.05	0.48	0.81	0.26	1.10	2.14	2.11	0.03
Amino20x4	2.44	0.07	0.43	0.18	0.56	1.28	1.28	0.00
PCONF	1.62	0.50	1.29	0.80	1.67	3.62	3.45	0.17
MCONF	4.97	-0.81	0.88	0.18	1.03	1.97	1.94	0.03
SCONF	4.60	0.03	0.16	0.04	0.24	0.78	0.76	0.02
PArel	4.63	0.88	1.14	0.25	1.60	4.46	4.46	0.00
BUT14DIOL	2.80	-0.42	0.42	0.15	0.43	0.66	0.59	0.07
EIE22	5.44	0.87	0.90	0.16	1.05	1.99	1.94	0.05
Styrene45	62.64	-0.12	3.10	0.05	4.19	12.41	12.41	0.00
ISOMERIZATION20	31.84	1.36	2.90	0.09	6.57	28.06	28.03	0.03
DIE60	4.71	1.10	1.17	0.25	1.40	3.11	3.04	0.07
IDISP	14.22	0.25	5.12	0.36	5.96	10.82	9.61	1.21
C20C24	30.77	-13.50	13.50	0.44	14.18	29.76	21.01	8.75
S66	5.47	-1.97	2.23	0.41	3.26	17.29	16.87	0.42
S10x8	6.59	1.77	1.87	0.28	5.57	42.65	42.61	0.04
X40	3.76	1.21	1.21	0.32	1.46	4.15	4.12	0.03
HEAVY28	1.24	-0.27	0.31	0.25	0.35	0.85	0.83	0.02
CHB6	26.79	-0.15	0.92	0.03	1.28	2.84	2.75	0.09
AHB21	22.49	-0.89	1.34	0.06	1.91	5.74	5.69	0.05
IL16	109.04	1.89	1.89	0.02	2.03	3.93	3.42	0.51
PNICO23	4.27	-0.77	0.77	0.18	0.88	2.57	2.47	0.10
CT20	0.98	-2.93	3.91	3.98	10.34	34.50	34.21	0.29
CARBHB12	6.04	0.03	0.38	0.06	0.50	1.19	1.13	0.06
ADIM6	3.36	-2.24	2.24	0.67	2.47	4.66	3.88	0.78
3B-69-TRIM	12.30	3.12	4.01	0.33	5.38	29.21	28.58	0.63
ISOL24	21.92	-0.48	2.91	0.13	3.63	7.90	7.62	0.28
C60ISO	98.25	8.65	8.65	0.09	9.16	18.75	14.40	4.35
L7	18.20	13.09	13.09	0.72	14.37	25.88	20.21	5.67
UPU23	5.72	1.36	1.49	0.26	2.06	4.80	4.78	0.02
ENZYMES23	15.32	0.66	2.99	0.20	4.56	14.92	14.67	0.25

Table S109: Statistical analysis for mPW1B95 for all testset in our databsase. The numbers given (all in kcal/mol) are average reaction energy ($|\overline{\Delta E}|$), mean deviation (MD), mean absolute deviation (MAD), MAD normalized with respect to $|\overline{\Delta E}|$ (NMAD), root-mean-square deviation (RMSD), deviation span (Δ_{err}), maximum (max) and minimum deviation (min).

Test set	$\lvert\overline{\Delta E}\rvert$	MD	MAD	NMAD	RMSD	Δ_{err}	max	min
FH51	31.01	0.16	1.69	0.05	2.27	6.18	6.16	0.02
YBDE18	49.28	31.30	31.44	0.64	44.05	64.14	64.11	0.03
AL2X6	35.88	3.49	3.49	0.10	3.57	7.08	4.63	2.45
DARC	32.47	-5.64	5.64	0.17	6.27	12.95	10.05	2.90
NBPRC	27.71	-2.08	3.53	0.13	4.13	8.24	7.78	0.46
HEAVYSB9	58.02	-3.32	3.32	0.06	3.84	7.13	6.68	0.45
BSR36	16.20	-5.15	5.15	0.32	5.69	13.78	13.01	0.77
RSE43	7.60	-1.96	1.96	0.26	2.25	5.44	5.06	0.38
W4-11	306.91	-1.93	2.74	0.01	4.31	27.21	27.19	0.02
G21EA	33.62	-5.65	5.67	0.17	6.68	12.89	12.59	0.30
G21IP	257.61	-0.46	2.67	0.01	3.26	9.01	8.86	0.15
DIPCS10	654.26	-2.71	2.95	0.00	3.87	7.77	7.44	0.33
PA26	189.05	0.84	1.54	0.01	2.15	6.32	6.27	0.05
SIE4x4	33.72	13.56	13.56	0.40	15.79	33.50	32.43	1.07
ALKBDE10	100.69	42.44	42.44	0.42	48.37	91.80	74.52	17.28
RC21	35.70	2.46	2.89	0.08	3.50	7.64	7.44	0.20
ALK8	62.60	2.09	2.80	0.04	3.61	5.88	5.84	0.04
DC13	54.98	-0.50	9.12	0.17	10.87	22.97	22.47	0.50
G2RC	51.26	-1.62	3.91	0.08	4.72	10.08	9.99	0.09
BH76RC	21.39	-0.18	1.72	0.08	2.20	5.31	5.21	0.10
MOR23	35.57	7.23	7.41	0.21	14.85	65.83	65.72	0.11
WCPT18	34.99	-1.85	1.90	0.05	2.16	4.93	4.51	0.42
BHROT27	6.37	0.51	0.59	0.09	0.79	1.47	1.47	0.00
BHPERI	20.87	0.91	1.65	0.08	1.93	3.89	3.70	0.19
BHDIV10	45.33	-2.32	2.51	0.06	2.76	5.34	4.39	0.95
INV24	32.85	-0.98	1.62	0.05	2.07	4.93	4.92	0.01
CR20	19.31	3.38	3.38	0.18	3.60	6.60	5.60	1.00
CRBH20	46.13	2.90	2.90	0.06	2.98	6.03	4.21	1.82
TMBH17	12.76	2.52	3.49	0.27	4.50	8.55	8.55	0.00
LTMBH26	9.98	-0.31	1.97	0.20	3.53	15.16	15.13	0.03
BH76	18.61	-3.02	3.25	0.17	3.69	9.87	9.67	0.20
ISO34	14.57	-0.68	1.49	0.10	1.88	4.26	4.25	0.01
ICONF	3.27	0.20	0.31	0.09	0.45	1.17	1.17	0.00
ACONF	1.83	0.23	0.23	0.13	0.25	0.52	0.43	0.09
TAUT15	3.05	0.44	0.92	0.30	1.29	2.71	2.67	0.04
Amino20x4	2.44	0.05	0.41	0.17	0.51	1.33	1.33	0.00
PCONF	1.62	0.29	1.60	0.98	1.91	3.65	3.56	0.09
MCONF	4.97	-0.90	0.99	0.20	1.16	2.22	2.16	0.06

Continued on next page

Test set	$\lvert\overline{\Delta E}\rvert$	MD	MAD	NMAD	RMSD	Δ_{err}	max	min
SCONF	4.60	0.10	0.19	0.04	0.23	0.60	0.59	0.01
PArel	4.63	0.67	1.01	0.22	1.58	4.96	4.95	0.01
BUT14DIOL	2.80	-0.39	0.39	0.14	0.41	0.66	0.57	0.09
EIE22	5.44	1.16	1.18	0.22	1.34	2.47	2.37	0.10
Styrene45	62.64	-0.59	2.62	0.04	3.47	9.74	9.74	0.00
ISOMERIZATION20	31.84	2.29	4.17	0.13	9.78	42.32	42.16	0.16
DIE60	4.71	1.32	1.34	0.29	1.55	2.94	2.93	0.01
IDISP	14.22	1.24	6.18	0.43	7.43	15.29	13.97	1.32
C20C24	30.77	-14.94	14.94	0.49	15.32	31.54	19.78	11.76
S66	5.47	-2.06	2.06	0.38	2.27	6.02	5.48	0.54
S10x8	6.59	1.11	1.29	0.20	1.70	7.04	6.99	0.05
X40	3.76	1.35	1.35	0.36	1.60	4.87	4.59	0.28
HEAVY28	1.24	-0.09	0.18	0.14	0.23	0.63	0.63	0.00
CHB6	26.79	0.28	0.94	0.04	1.16	2.02	1.96	0.06
AHB21	22.49	-0.61	1.15	0.05	1.50	4.03	3.97	0.06
IL16	109.04	1.84	1.84	0.02	2.01	3.56	3.33	0.23
PNICO23	4.27	-0.82	0.85	0.20	0.95	2.84	2.57	0.27
CT20	0.98	0.52	0.52	0.53	0.54	1.09	0.84	0.25
CARBHB12	6.04	0.02	0.39	0.06	0.51	1.23	1.23	0.00
ADIM6	3.36	-2.43	2.43	0.72	2.68	5.08	4.23	0.85
3B-69-TRIM	12.30	0.95	7.43	0.60	16.03	82.27	81.30	0.97
ISOL24	21.92	-1.67	3.80	0.17	5.25	15.88	15.73	0.15
C60ISO	98.25	2.92	2.93	0.03	3.38	5.69	5.66	0.03
L7	18.20	12.58	12.58	0.69	14.05	27.92	21.99	5.93
UPU23	5.72	1.36	1.55	0.27	2.19	5.47	5.31	0.16
ENZYMES23	15.32	-0.77	3.05	0.20	4.38	13.86	13.84	0.02

Table S110: Statistical analysis for mPW1PW for all testset in our databsase. The numbers given (all in kcal/mol) are average reaction energy ($\lvert\overline{\Delta E}\rvert$), mean deviation (MD), mean absolute deviation (MAD), MAD normalized with respect to $\lvert\overline{\Delta E}\rvert$ (NMAD), root-mean-square deviation (RMSD), deviation span (Δ_{err}), maximum (max) and minimum deviation (min).

Test set	$\lvert\overline{\Delta E}\rvert$	MD	MAD	NMAD	RMSD	Δ_{err}	max	min
FH51	31.01	0.34	2.29	0.07	3.05	8.70	8.70	0.00
YBDE18	49.28	-5.90	5.90	0.12	6.24	14.24	10.94	3.30
AL2X6	35.88	-4.54	4.54	0.13	5.05	8.04	6.91	1.13
DARC	32.47	3.52	4.28	0.13	5.03	7.39	7.00	0.39
NBPRC	27.71	0.74	2.09	0.08	2.76	6.38	5.96	0.42
HEAVYSB9	58.02	-6.55	6.55	0.11	6.86	13.47	10.16	3.31
BSR36	16.20	-9.09	9.09	0.56	10.24	25.69	23.12	2.57
RSE43	7.60	-1.23	1.23	0.16	1.47	3.97	3.86	0.11
W4-11	306.91	-7.37	7.50	0.02	8.92	32.39	31.41	0.98

Continued on next page

Test set	$\lvert\overline{\Delta E}\rvert$	MD	MAD	NMAD	RMSD	Δ_{err}	max	min
G21EA	33.62	-3.49	4.28	0.13	5.65	11.49	11.48	0.01
G21IP	257.61	1.74	3.97	0.02	4.77	10.57	10.50	0.07
DIPCS10	654.26	2.40	3.70	0.01	4.18	8.47	7.57	0.90
PA26	189.05	2.35	2.40	0.01	3.20	9.05	8.65	0.40
SIE4x4	33.72	15.45	15.45	0.46	17.35	36.18	32.25	3.93
ALKBDE10	100.69	41.82	41.82	0.42	48.24	89.08	73.43	15.65
RC21	35.70	1.99	2.36	0.07	2.88	6.88	6.54	0.34
ALK8	62.60	-0.27	1.63	0.03	2.15	4.29	4.02	0.27
DC13	54.98	0.60	9.55	0.17	12.10	21.99	21.93	0.06
G2RC	51.26	-1.95	4.62	0.09	5.67	12.52	12.36	0.16
BH76RC	21.39	-0.38	1.78	0.08	2.44	6.51	6.34	0.17
MOR23	35.57	5.47	6.32	0.18	7.65	13.35	13.18	0.17
WCPT18	34.99	-2.43	2.43	0.07	2.90	6.08	6.08	0.00
BHROT27	6.37	0.44	0.52	0.08	0.70	1.43	1.41	0.02
BHPERI	20.87	1.07	1.78	0.09	2.03	4.42	4.41	0.01
BHDIV10	45.33	-2.46	3.18	0.07	3.65	5.49	5.46	0.03
INV24	32.85	-1.20	1.71	0.05	2.06	4.85	4.74	0.11
CR20	19.31	-4.18	4.18	0.22	4.45	9.16	7.33	1.83
CRBH20	46.13	-1.21	1.30	0.03	1.69	3.52	3.43	0.09
TMBH17	12.76	2.45	3.56	0.28	4.44	8.36	8.36	0.00
LTMBH26	9.98	-1.00	2.46	0.25	3.88	15.50	15.30	0.20
BH76	18.61	-3.44	3.77	0.20	4.18	10.84	10.48	0.36
ISO34	14.57	-0.42	1.46	0.10	2.20	7.98	7.96	0.02
ICONF	3.27	0.26	0.50	0.15	0.64	1.59	1.47	0.12
ACONF	1.83	0.79	0.79	0.43	0.88	1.73	1.44	0.29
TAUT15	3.05	0.29	1.10	0.36	1.38	2.85	2.74	0.11
Amino20x4	2.44	-0.04	0.55	0.23	0.69	1.95	1.95	0.00
PCONF	1.62	-0.44	3.60	2.22	4.07	6.57	6.39	0.18
MCONF	4.97	-1.98	2.04	0.41	2.31	3.77	3.65	0.12
SCONF	4.60	-0.11	0.40	0.09	0.54	1.30	1.30	0.00
PArel	4.63	0.47	1.05	0.23	1.55	5.11	5.08	0.03
BUT14DIOL	2.80	-0.20	0.35	0.12	0.40	0.79	0.77	0.02
EIE22	5.44	1.37	1.38	0.25	1.56	2.92	2.87	0.05
Styrene45	62.64	1.05	2.10	0.03	2.79	6.48	6.48	0.00
ISOMERIZATION20	31.84	0.07	1.91	0.06	2.46	5.28	5.09	0.19
DIE60	4.71	1.45	1.47	0.31	1.68	3.40	3.31	0.09
IDISP	14.22	1.67	12.10	0.85	13.22	22.95	19.78	3.17
C20C24	30.77	-8.22	21.82	0.71	27.59	56.23	50.31	5.92
S66	5.47	-3.12	3.12	0.57	3.60	9.31	8.71	0.60
S10x8	6.59	1.47	1.47	0.22	2.04	8.18	8.13	0.05
X40	3.76	1.91	1.91	0.51	2.35	7.39	7.34	0.05
HEAVY28	1.24	-0.96	0.96	0.77	1.04	1.89	1.85	0.04
CHB6	26.79	1.38	1.38	0.05	1.73	3.43	3.30	0.13
AHB21	22.49	-0.76	1.43	0.06	1.81	4.46	4.22	0.24

Continued on next page

Test set	$\lvert\overline{\Delta E}\rvert$	MD	MAD	NMAD	RMSD	Δ_{err}	max	min
IL16	109.04	2.34	2.34	0.02	2.62	5.21	4.91	0.30
PNICO23	4.27	-1.10	1.16	0.27	1.26	3.67	3.11	0.56
CT20	0.98	0.81	0.81	0.83	0.85	1.65	1.22	0.43
CARBHB12	6.04	0.27	0.55	0.09	0.80	2.01	1.98	0.03
ADIM6	3.36	-4.62	4.62	1.38	5.07	9.47	7.76	1.71
3B-69-TRIM	12.30	5.77	5.77	0.47	6.42	14.26	13.48	0.78
ISOL24	21.92	-2.02	4.81	0.22	7.81	28.04	27.70	0.34
C60ISO	98.25	2.43	2.88	0.03	3.17	6.39	5.57	0.82
L7	18.20	21.73	21.73	1.19	24.25	48.90	40.30	8.60
UPU23	5.72	2.01	2.31	0.40	3.42	9.08	9.08	0.00
ENZYMES23	15.32	-1.24	3.74	0.24	5.00	15.46	15.08	0.38

Table S111: Statistical analysis for PWB6K for all testset in our databsase. The numbers given (all in kcal/mol) are average reaction energy ($\lvert\overline{\Delta E}\rvert$), mean deviation (MD), mean absolute deviation (MAD), MAD normalized with respect to $\lvert\overline{\Delta E}\rvert$ (NMAD), root-mean-square deviation (RMSD), deviation span (Δ_{err}), maximum (max) and minimum deviation (min).

Test set	$\lvert\overline{\Delta E}\rvert$	MD	MAD	NMAD	RMSD	Δ_{err}	max	min
FH51	31.01	-1.27	2.07	0.07	2.58	5.91	5.91	0.00
YBDE18	49.28	-3.31	3.31	0.07	3.65	7.25	6.22	1.03
AL2X6	35.88	-0.46	0.90	0.03	1.12	2.14	1.98	0.16
DARC	32.47	-0.86	1.54	0.05	2.07	5.07	5.03	0.04
NBPRC	27.71	-1.08	2.17	0.08	2.43	4.27	3.69	0.58
HEAVYSB9	58.02	-3.78	3.78	0.07	3.93	7.35	5.37	1.98
BSR36	16.20	-5.65	5.65	0.35	6.35	16.17	14.43	1.74
RSE43	7.60	-1.13	1.13	0.15	1.36	3.43	3.31	0.12
W4-11	306.91	-10.81	10.84	0.04	13.37	40.53	40.50	0.03
G21EA	33.62	-5.76	5.88	0.18	7.32	15.48	14.26	1.22
G21IP	257.61	0.73	2.91	0.01	3.96	10.79	10.67	0.12
DIPCS10	654.26	0.24	3.47	0.01	4.47	9.33	9.27	0.06
PA26	189.05	1.46	1.99	0.01	2.66	6.96	6.91	0.05
SIE4x4	33.72	9.61	9.61	0.29	11.29	25.61	22.95	2.66
ALKBDE10	100.69	45.34	45.34	0.45	54.51	106.99	99.76	7.23
RC21	35.70	2.59	2.85	0.08	3.70	10.77	10.68	0.09
ALK8	62.60	4.04	4.04	0.06	5.06	7.80	7.62	0.18
DC13	54.98	-2.44	9.29	0.17	11.40	22.09	21.96	0.13
G2RC	51.26	-3.78	4.99	0.10	6.05	14.04	13.33	0.71
BH76RC	21.39	-0.31	2.78	0.13	3.17	7.64	7.00	0.64
MOR23	35.57	3.86	4.52	0.13	5.68	13.24	13.12	0.12
WCPT18	34.99	1.10	1.19	0.03	1.68	5.38	5.36	0.02
BHROT27	6.37	0.63	0.68	0.11	0.93	1.98	1.95	0.03
BHPERI	20.87	2.88	3.05	0.15	3.38	7.78	6.89	0.89

Continued on next page

Test set	$\lvert\overline{\Delta E}\rvert$	MD	MAD	NMAD	RMSD	Δ_{err}	max	min
BHDIV10	45.33	0.30	1.16	0.03	1.37	2.57	2.52	0.05
INV24	32.85	0.12	2.03	0.06	3.25	10.94	10.91	0.03
CR20	19.31	-2.48	2.50	0.13	2.92	5.69	5.55	0.14
CRBH20	46.13	1.83	1.98	0.04	2.23	4.24	3.71	0.53
TMBH17	12.76	4.54	4.88	0.38	6.52	13.57	13.51	0.06
LTMBH26	9.98	1.38	2.02	0.20	3.85	16.79	16.65	0.14
BH76	18.61	-0.47	1.48	0.08	1.96	6.68	6.68	0.00
ISO34	14.57	-0.39	1.47	0.10	1.84	3.97	3.96	0.01
ICONF	3.27	0.23	0.39	0.12	0.57	1.44	1.37	0.07
ACONF	1.83	0.17	0.17	0.09	0.18	0.40	0.33	0.07
TAUT15	3.05	0.36	0.74	0.24	0.98	2.03	1.98	0.05
Amino20x4	2.44	0.07	0.41	0.17	0.54	1.23	1.22	0.01
PCONF	1.62	0.55	1.00	0.62	1.33	2.89	2.82	0.07
MCONF	4.97	-0.65	0.70	0.14	0.84	1.64	1.62	0.02
SCONF	4.60	-0.08	0.22	0.05	0.29	0.91	0.84	0.07
PArel	4.63	0.81	1.00	0.22	1.51	4.39	4.39	0.00
BUT14DIOL	2.80	-0.35	0.35	0.12	0.36	0.55	0.51	0.04
EIE22	5.44	0.82	0.83	0.15	0.97	1.83	1.82	0.01
Styrene45	62.64	0.69	2.66	0.04	3.47	9.49	9.49	0.00
ISOMERIZATION20	31.84	-0.39	1.80	0.06	2.18	4.33	4.32	0.01
DIE60	4.71	1.02	1.08	0.23	1.31	2.93	2.87	0.06
IDISP	14.22	0.56	5.14	0.36	6.27	11.35	10.44	0.91
C20C24	30.77	-9.50	13.56	0.44	18.97	38.42	36.44	1.98
S66	5.47	-1.09	1.09	0.20	1.31	3.68	3.67	0.01
S10x8	6.59	0.50	0.60	0.09	0.94	3.71	3.71	0.00
X40	3.76	0.66	0.71	0.19	0.99	3.11	3.09	0.02
HEAVY28	1.24	-0.36	0.38	0.31	0.42	0.83	0.80	0.03
CHB6	26.79	-1.05	1.17	0.04	1.65	3.74	3.44	0.30
AHB21	22.49	-1.33	1.47	0.07	2.15	6.28	6.26	0.02
IL16	109.04	1.33	1.33	0.01	1.48	2.74	2.69	0.05
PNICO23	4.27	-0.38	0.41	0.10	0.56	1.95	1.92	0.03
CT20	0.98	1.64	1.78	1.81	6.39	25.57	25.55	0.02
CARBHB12	6.04	0.40	0.47	0.08	0.62	1.50	1.41	0.09
ADIM6	3.36	-1.18	1.18	0.35	1.39	2.55	2.38	0.17
3B-69-TRIM	12.30	2.12	3.59	0.29	8.94	50.67	50.62	0.05
ISOL24	21.92	-0.73	2.98	0.14	3.74	9.28	9.24	0.04
C60ISO	98.25	9.70	9.70	0.10	10.31	20.69	16.07	4.62
L7	18.20	18.40	18.40	1.01	23.24	46.73	41.55	5.18
UPU23	5.72	1.17	1.25	0.22	1.69	3.73	3.63	0.10
ENZYMES23	15.32	0.59	3.02	0.20	4.52	14.44	14.27	0.17

Table S112: Statistical analysis for SOGGA11-X for all testset in our databsase. The numbers given (all in kcal/mol) are average reaction energy ($|\overline{\Delta E}|$), mean deviation (MD), mean absolute deviation (MAD), MAD normalized with respect to $|\overline{\Delta E}|$ (NMAD), root-mean-square deviation (RMSD), deviation span (Δ_{err}), maximum (max) and minimum deviation (min).

Test set	$\lvert\overline{\Delta E}\rvert$	MD	MAD	NMAD	RMSD	Δ_{err}	max	min
FH51	31.01	-0.35	2.07	0.07	2.65	7.27	7.21	0.06
YBDE18	49.28	1.09	6.08	0.12	6.44	12.24	9.53	2.71
AL2X6	35.88	-4.34	4.34	0.12	4.79	9.38	7.22	2.16
DARC	32.47	0.18	2.27	0.07	2.56	5.82	5.05	0.77
NBPRC	27.71	1.94	2.70	0.10	3.45	7.76	7.45	0.31
HEAVYSB9	58.02	-4.41	4.41	0.08	4.74	7.93	6.62	1.31
BSR36	16.20	-9.01	9.01	0.56	10.35	26.83	23.89	2.94
RSE43	7.60	-1.23	1.23	0.16	1.42	3.42	3.14	0.28
W4-11	306.91	-6.11	6.18	0.02	8.16	32.81	32.61	0.20
G21EA	33.62	-2.76	3.32	0.10	4.77	10.65	10.63	0.02
G21IP	257.61	2.40	3.28	0.01	4.26	9.40	9.22	0.18
DIPCS10	654.26	1.90	4.81	0.01	5.72	9.85	9.83	0.02
PA26	189.05	2.41	2.53	0.01	3.50	9.15	9.12	0.03
SIE4x4	33.72	9.64	9.64	0.29	11.54	25.58	25.08	0.50
ALKBDE10	100.69	-7.15	7.15	0.07	8.63	20.87	19.47	1.40
RC21	35.70	2.14	2.61	0.07	3.23	7.24	7.21	0.03
ALK8	62.60	-0.18	1.56	0.02	2.07	4.06	4.04	0.02
DC13	54.98	-0.46	8.49	0.15	10.26	20.71	20.21	0.50
G2RC	51.26	-2.55	4.22	0.08	5.49	13.23	13.00	0.23
BH76RC	21.39	0.17	2.33	0.11	2.69	5.80	5.69	0.11
MOR23	35.57	10.12	10.88	0.31	16.99	69.66	68.25	1.41
WCPT18	34.99	0.98	1.23	0.04	1.54	4.17	4.03	0.14
BHROT27	6.37	0.33	0.38	0.06	0.49	1.15	1.13	0.02
BHPERI	20.87	3.51	3.64	0.17	3.89	7.49	6.68	0.81
BHDIV10	45.33	0.09	1.18	0.03	1.56	3.42	3.39	0.03
INV24	32.85	-0.03	1.76	0.05	2.89	9.92	9.79	0.13
CR20	19.31	-1.66	1.67	0.09	2.00	4.35	4.35	0.00
CRBH20	46.13	0.41	1.11	0.02	1.34	2.56	2.49	0.07
TMBH17	12.76	4.34	4.75	0.37	5.88	11.44	11.32	0.12
LTMBH26	9.98	0.19	2.10	0.21	3.83	17.27	17.26	0.01
BH76	18.61	-1.27	1.73	0.09	2.16	6.02	6.02	0.00
ISO34	14.57	-0.53	1.40	0.10	1.95	6.67	6.63	0.04
ICONF	3.27	0.22	0.40	0.12	0.54	1.19	1.19	0.00
ACONF	1.83	0.44	0.44	0.24	0.49	1.05	0.90	0.15
TAUT15	3.05	0.50	0.50	0.17	0.75	1.94	1.92	0.02
Amino20x4	2.44	-0.08	0.39	0.16	0.49	1.48	1.48	0.00
PCONF	1.62	0.29	1.92	1.18	2.37	4.49	4.24	0.25
MCONF	4.97	-1.44	1.48	0.30	1.68	2.70	2.68	0.02

Continued on next page

Test set	$\lvert\overline{\Delta E}\rvert$	MD	MAD	NMAD	RMSD	Δ_{err}	max	min
SCONF	4.60	-0.04	0.22	0.05	0.26	0.48	0.47	0.01
PArel	4.63	0.54	0.85	0.18	1.25	3.53	3.53	0.00
BUT14DIOL	2.80	0.12	0.18	0.06	0.24	0.69	0.69	0.00
EIE22	5.44	0.96	0.96	0.18	1.09	2.08	2.05	0.03
Styrene45	62.64	1.08	2.26	0.04	2.85	6.76	6.76	0.00
ISOMERIZATION20	31.84	0.21	1.66	0.05	1.93	3.62	3.55	0.07
DIE60	4.71	1.05	1.10	0.23	1.34	2.94	2.94	0.00
IDISP	14.22	1.70	9.74	0.68	10.84	19.29	16.83	2.46
C20C24	30.77	-9.05	15.61	0.51	20.73	43.18	38.42	4.76
S66	5.47	-2.65	2.65	0.49	3.01	7.20	6.60	0.60
S10x8	6.59	1.42	1.42	0.22	1.84	7.09	7.04	0.05
X40	3.76	1.81	1.81	0.48	2.11	6.50	6.21	0.29
HEAVY28	1.24	-0.89	0.89	0.72	0.94	1.73	1.70	0.03
CHB6	26.79	1.13	1.26	0.05	1.62	3.06	3.04	0.02
AHB21	22.49	-0.56	1.12	0.05	1.52	4.40	4.32	0.08
IL16	109.04	2.33	2.33	0.02	2.46	5.08	4.10	0.98
PNICO23	4.27	-1.22	1.22	0.29	1.31	3.84	3.00	0.84
CT20	0.98	1.21	1.21	1.23	1.25	2.40	1.82	0.58
CARBHB12	6.04	-0.06	0.47	0.08	0.59	1.31	1.29	0.02
ADIM6	3.36	-4.34	4.34	1.29	4.61	8.61	6.51	2.10
3B-69-TRIM	12.30	5.62	5.62	0.46	6.16	13.36	12.67	0.69
ISOL24	21.92	-1.68	3.50	0.16	5.58	21.42	21.32	0.10
C60ISO	98.25	7.55	7.55	0.08	8.05	17.18	13.18	4.00
L7	18.20	18.08	18.08	0.99	19.80	39.30	31.59	7.71
UPU23	5.72	1.98	2.15	0.38	3.00	6.83	6.69	0.14
ENZYMES23	15.32	0.22	2.71	0.18	4.87	16.52	16.46	0.06

Table S113: Statistical analysis for M05-2X for all testset in our databsase. The numbers given (all in kcal/mol) are average reaction energy ($\lvert\overline{\Delta E}\rvert$), mean deviation (MD), mean absolute deviation (MAD), MAD normalized with respect to $\lvert\overline{\Delta E}\rvert$ (NMAD), root-mean-square deviation (RMSD), deviation span (Δ_{err}), maximum (max) and minimum deviation (min).

Test set	$\lvert\overline{\Delta E}\rvert$	MD	MAD	NMAD	RMSD	Δ_{err}	max	min
FH51	31.01	-1.09	1.64	0.05	2.11	5.37	5.35	0.02
YBDE18	49.28	14.35	14.35	0.29	19.64	29.51	29.23	0.28
AL2X6	35.88	2.22	2.22	0.06	2.49	5.52	4.25	1.27
DARC	32.47	-3.64	3.64	0.11	3.91	8.36	6.09	2.27
NBPRC	27.71	-0.21	1.30	0.05	1.62	3.45	3.22	0.23
HEAVYSB9	58.02	-4.52	4.52	0.08	5.30	10.00	9.32	0.68
BSR36	16.20	-2.06	2.06	0.13	2.42	6.80	6.01	0.79
RSE43	7.60	-0.52	0.67	0.09	0.83	2.43	2.41	0.02
W4-11	306.91	-4.97	6.74	0.02	9.68	36.00	35.60	0.40

Continued on next page

Test set	$\lvert\overline{\Delta E}\rvert$	MD	MAD	NMAD	RMSD	Δ_{err}	max	min
G21EA	33.62	-2.37	3.31	0.10	4.13	8.70	8.68	0.02
G21IP	257.61	1.46	3.46	0.01	4.65	13.45	13.43	0.02
DIPCS10	654.26	0.75	3.91	0.01	4.59	10.31	9.65	0.66
PA26	189.05	-0.82	1.69	0.01	1.99	4.87	4.79	0.08
SIE4x4	33.72	5.85	5.85	0.17	6.16	12.07	8.76	3.31
ALKBDE10	100.69	-2.10	5.09	0.05	7.43	18.30	18.16	0.14
RC21	35.70	1.68	2.05	0.06	2.48	4.62	4.60	0.02
ALK8	62.60	0.80	2.20	0.04	3.19	6.22	6.18	0.04
DC13	54.98	-1.69	9.78	0.18	12.99	28.49	28.21	0.28
G2RC	51.26	-3.63	4.24	0.08	5.15	11.31	10.26	1.05
BH76RC	21.39	-0.90	1.46	0.07	2.00	5.45	5.37	0.08
MOR23	35.57	5.81	7.91	0.22	15.52	68.43	67.33	1.10
WCPT18	34.99	-1.72	2.68	0.08	3.34	5.65	5.56	0.09
BHROT27	6.37	0.35	0.42	0.07	0.57	1.17	1.16	0.01
BHPERI	20.87	-0.74	1.44	0.07	1.64	3.17	3.05	0.12
BHDIV10	45.33	-0.30	1.54	0.03	1.82	3.40	3.36	0.04
INV24	32.85	-0.12	1.71	0.05	2.55	7.22	7.21	0.01
CR20	19.31	2.00	2.00	0.10	2.21	3.82	3.77	0.05
CRBH20	46.13	4.09	4.09	0.09	4.19	7.69	5.40	2.29
TMBH17	12.76	4.02	5.21	0.41	6.76	13.91	13.43	0.48
LTMBH26	9.98	0.43	1.48	0.15	2.93	13.33	13.31	0.02
BH76	18.61	-0.70	2.05	0.11	2.68	7.89	7.54	0.35
ISO34	14.57	0.07	1.07	0.07	1.56	5.44	5.42	0.02
ICONF	3.27	0.12	0.38	0.12	0.51	1.03	1.00	0.03
ACONF	1.83	0.04	0.05	0.03	0.08	0.24	0.23	0.01
TAUT15	3.05	0.71	1.05	0.35	1.35	3.07	2.99	0.08
Amino20x4	2.44	0.10	0.25	0.10	0.33	0.92	0.92	0.00
PCONF	1.62	0.20	0.45	0.28	0.52	1.09	1.01	0.08
MCONF	4.97	-0.09	0.16	0.03	0.19	0.46	0.45	0.01
SCONF	4.60	0.19	0.29	0.06	0.34	0.68	0.63	0.05
PArel	4.63	0.26	0.96	0.21	1.49	3.84	3.82	0.02
BUT14DIOL	2.80	-0.13	0.14	0.05	0.19	0.59	0.58	0.01
EIE22	5.44	0.15	0.32	0.06	0.39	0.94	0.90	0.04
Styrene45	62.64	3.67	3.76	0.06	4.52	10.98	10.98	0.00
ISOMERIZATION20	31.84	-0.15	1.49	0.05	1.82	3.59	3.47	0.12
DIE60	4.71	0.22	0.43	0.09	0.67	1.66	1.66	0.00
IDISP	14.22	0.45	1.19	0.08	1.61	3.40	3.24	0.16
C20C24	30.77	10.69	18.11	0.59	19.02	35.55	24.84	10.71
S66	5.47	-0.63	0.65	0.12	0.79	1.68	1.67	0.01
S10x8	6.59	0.24	0.36	0.05	0.54	1.96	1.96	0.00
X40	3.76	0.38	0.46	0.12	0.63	1.63	1.62	0.01
HEAVY28	1.24	0.12	0.27	0.22	0.36	0.87	0.85	0.02
CHB6	26.79	-0.53	0.72	0.03	1.17	2.76	2.74	0.02
AHB21	22.49	-1.63	1.70	0.08	2.50	7.22	7.19	0.03

Continued on next page

Test set	$\lvert\overline{\Delta E}\rvert$	MD	MAD	NMAD	RMSD	Δ_{err}	max	min
IL16	109.04	1.51	1.51	0.01	1.65	3.49	2.88	0.61
PNICO23	4.27	0.04	0.20	0.05	0.31	1.07	1.06	0.01
CT20	0.98	0.14	0.19	0.19	0.25	0.70	0.68	0.02
CARBHB12	6.04	0.42	0.43	0.07	0.60	1.34	1.33	0.01
ADIM6	3.36	-0.85	0.85	0.25	0.96	1.68	1.48	0.20
3B-69-TRIM	12.30	1.33	1.40	0.11	1.73	4.85	4.84	0.01
ISOL24	21.92	0.19	2.45	0.11	3.81	13.92	13.78	0.14
C60ISO	98.25	10.81	10.81	0.11	11.51	21.10	16.36	4.74
L7	18.20	8.59	8.59	0.47	9.95	19.56	16.60	2.96
UPU23	5.72	0.95	1.01	0.18	1.26	3.07	2.96	0.11
ENZYMES23	15.32	-0.12	1.82	0.12	2.66	9.66	9.64	0.02

Table S114: Statistical analysis for M06 for all testset in our databsase. The numbers given (all in kcal/mol) are average reaction energy ($\lvert\overline{\Delta E}\rvert$), mean deviation (MD), mean absolute deviation (MAD), MAD normalized with respect to $\lvert\overline{\Delta E}\rvert$ (NMAD), root-mean-square deviation (RMSD), deviation span (Δ_{err}), maximum (max) and minimum deviation (min).

Test set	$\lvert\overline{\Delta E}\rvert$	MD	MAD	NMAD	RMSD	Δ_{err}	max	min
FH51	31.01	-0.02	1.94	0.06	2.47	6.03	6.01	0.02
YBDE18	49.28	33.88	34.83	0.71	48.59	70.42	70.39	0.03
AL2X6	35.88	2.95	2.95	0.08	3.46	7.85	6.41	1.44
DARC	32.47	-4.63	4.63	0.14	4.88	10.37	7.17	3.20
NBPRC	27.71	-1.38	2.65	0.10	3.01	5.46	5.11	0.35
HEAVYSB9	58.02	-0.72	1.73	0.03	1.96	4.25	3.96	0.29
BSR36	16.20	-1.82	1.82	0.11	2.29	7.28	6.73	0.55
RSE43	7.60	-1.46	1.46	0.19	1.85	6.68	6.35	0.33
W4-11	306.91	-2.25	4.52	0.01	6.04	21.13	20.89	0.24
G21EA	33.62	-5.17	5.30	0.16	6.02	11.73	10.94	0.79
G21IP	257.61	-0.93	3.04	0.01	3.87	8.96	8.89	0.07
DIPCS10	654.26	-5.18	5.39	0.01	7.01	13.23	12.96	0.27
PA26	189.05	1.72	2.12	0.01	3.04	8.46	8.34	0.12
SIE4x4	33.72	11.81	11.81	0.35	13.23	23.60	21.32	2.28
ALKBDE10	100.69	-0.24	3.84	0.04	4.96	11.52	11.33	0.19
RC21	35.70	0.22	1.51	0.04	2.05	4.76	4.68	0.08
ALK8	62.60	-0.15	3.40	0.05	4.06	6.35	5.81	0.54
DC13	54.98	-1.04	7.68	0.14	10.10	20.51	19.80	0.71
G2RC	51.26	-0.34	3.92	0.08	5.31	13.87	13.73	0.14
BH76RC	21.39	-0.67	1.58	0.07	2.20	6.27	6.12	0.15
MOR23	35.57	4.04	5.15	0.14	6.10	11.91	11.49	0.42
WCPT18	34.99	-1.87	2.08	0.06	2.58	5.43	5.12	0.31
BHROT27	6.37	0.63	0.65	0.10	0.88	2.23	2.22	0.01
BHPERI	20.87	1.77	2.22	0.11	2.61	5.10	4.81	0.29

Continued on next page

Test set	$\lvert\overline{\Delta E}\rvert$	MD	MAD	NMAD	RMSD	Δ_{err}	max	min
BHDIV10	45.33	-1.29	1.89	0.04	2.43	4.45	4.33	0.12
INV24	32.85	-0.93	1.55	0.05	2.30	7.35	7.34	0.01
CR20	19.31	-3.06	3.06	0.16	3.26	6.29	5.28	1.01
CRBH20	46.13	-3.62	3.62	0.08	3.67	7.36	4.83	2.53
TMBH17	12.76	2.70	3.45	0.27	4.89	12.22	11.95	0.27
LTMBH26	9.98	-1.22	3.36	0.34	4.89	17.95	17.94	0.01
BH76	18.61	-2.15	2.69	0.14	3.31	10.28	10.23	0.05
ISO34	14.57	-0.66	1.38	0.09	1.85	4.72	4.70	0.02
ICONF	3.27	-0.06	0.41	0.12	0.54	1.24	1.24	0.00
ACONF	1.83	-0.22	0.22	0.12	0.26	0.45	0.44	0.01
TAUT15	3.05	-0.33	0.83	0.27	1.05	2.29	2.25	0.04
Amino20x4	2.44	-0.04	0.33	0.13	0.42	1.11	1.10	0.01
PCONF	1.62	0.30	0.43	0.26	0.50	1.00	0.95	0.05
MCONF	4.97	-0.12	0.30	0.06	0.37	0.93	0.90	0.03
SCONF	4.60	-0.15	0.23	0.05	0.29	0.69	0.68	0.01
PArel	4.63	0.56	0.98	0.21	1.63	5.72	5.72	0.00
BUT14DIOL	2.80	0.18	0.19	0.07	0.22	0.42	0.42	0.00
EIE22	5.44	1.11	1.13	0.21	1.34	2.64	2.53	0.11
Styrene45	62.64	-2.61	3.80	0.06	5.08	12.90	12.90	0.00
ISOMERIZATION20	31.84	1.97	4.41	0.14	10.39	44.45	44.42	0.03
DIE60	4.71	1.21	1.27	0.27	1.53	3.22	3.16	0.06
IDISP	14.22	1.76	3.79	0.27	5.64	12.37	12.23	0.14
C20C24	30.77	-15.41	15.57	0.51	25.22	49.15	49.10	0.05
S66	5.47	-1.06	1.06	0.19	1.17	2.93	2.71	0.22
S10x8	6.59	0.85	0.85	0.13	1.00	2.57	2.57	0.00
X40	3.76	0.75	0.75	0.20	0.87	2.48	2.41	0.07
HEAVY28	1.24	0.75	0.78	0.63	0.89	1.80	1.62	0.18
CHB6	26.79	2.40	2.40	0.09	2.65	4.69	3.69	1.00
AHB21	22.49	-0.62	0.75	0.03	1.13	3.14	3.13	0.01
IL16	109.04	0.40	0.83	0.01	0.94	1.68	1.55	0.13
PNICO23	4.27	-0.23	0.25	0.06	0.33	0.74	0.73	0.01
CT20	0.98	0.77	0.77	0.78	0.81	1.32	1.16	0.16
CARBHB12	6.04	0.05	0.30	0.05	0.42	0.95	0.92	0.03
ADIM6	3.36	-0.16	0.27	0.08	0.28	0.55	0.39	0.16
3B-69-TRIM	12.30	2.73	2.73	0.22	2.94	5.99	5.59	0.40
ISOL24	21.92	-2.55	3.82	0.17	5.75	19.63	19.49	0.14
C60ISO	98.25	-0.19	1.82	0.02	2.21	4.76	4.46	0.30
L7	18.20	7.63	7.63	0.42	8.58	17.66	14.13	3.53
UPU23	5.72	0.30	0.54	0.09	0.72	1.93	1.93	0.00
ENZYMES23	15.32	-2.35	4.49	0.29	5.79	16.90	16.83	0.07

Table S115: Statistical analysis for M06-2X for all testset in our databsase. The numbers given (all in kcal/mol) are average reaction energy ($|\overline{\Delta E}|$), mean deviation (MD), mean absolute deviation (MAD), MAD normalized with respect to $|\overline{\Delta E}|$ (NMAD), root-mean-square deviation (RMSD), deviation span (Δ_{err}), maximum (max) and minimum deviation (min).

Test set	$\|\overline{\Delta E}\|$	MD	MAD	NMAD	RMSD	Δ_{err}	max	min
FH51	31.01	1.27	1.27	0.04	1.63	4.06	4.01	0.05
YBDE18	49.28	-3.42	3.42	0.07	3.70	6.45	5.79	0.66
AL2X6	35.88	0.48	0.95	0.03	1.40	2.91	2.86	0.05
DARC	32.47	1.83	1.94	0.06	2.23	3.19	3.04	0.15
NBPRC	27.71	0.06	0.85	0.03	1.11	2.45	2.41	0.04
HEAVYSB9	58.02	-10.13	10.13	0.17	11.13	19.67	17.19	2.48
BSR36	16.20	-3.14	3.14	0.19	3.99	12.40	11.39	1.01
RSE43	7.60	-0.24	0.52	0.07	0.62	1.47	1.45	0.02
W4-11	306.91	-4.12	4.42	0.01	6.27	30.39	30.39	0.00
G21EA	33.62	-3.64	3.85	0.11	4.76	9.94	9.58	0.36
G21IP	257.61	0.47	2.70	0.01	3.71	11.01	10.83	0.18
DIPCS10	654.26	0.15	2.80	0.00	3.37	7.49	6.66	0.83
PA26	189.05	-1.24	1.76	0.01	2.00	4.18	4.10	0.08
SIE4x4	33.72	8.12	8.12	0.24	9.87	22.87	22.53	0.34
ALKBDE10	100.69	-4.76	5.54	0.06	7.83	19.06	18.65	0.41
RC21	35.70	0.71	1.49	0.04	1.85	4.91	4.71	0.20
ALK8	62.60	1.13	2.37	0.04	3.35	6.50	6.31	0.19
DC13	54.98	-3.28	8.81	0.16	12.18	29.93	29.31	0.62
G2RC	51.26	-1.33	1.95	0.04	2.58	7.21	7.13	0.08
BH76RC	21.39	-0.45	1.40	0.07	1.95	6.04	5.95	0.09
MOR23	35.57	8.09	10.22	0.29	16.38	67.13	67.09	0.04
WCPT18	34.99	-1.23	2.01	0.06	2.52	4.86	4.70	0.16
BHROT27	6.37	0.34	0.34	0.05	0.47	1.18	1.14	0.04
BHPERI	20.87	0.67	1.36	0.07	1.78	4.59	4.50	0.09
BHDIV10	45.33	0.95	0.95	0.02	1.26	2.82	2.65	0.17
INV24	32.85	1.49	1.49	0.05	2.23	8.17	8.04	0.13
CR20	19.31	-2.21	2.26	0.12	2.59	5.02	4.48	0.54
CRBH20	46.13	0.92	1.31	0.03	1.53	2.97	2.94	0.03
TMBH17	12.76	4.36	5.67	0.44	7.36	15.36	15.19	0.17
LTMBH26	9.98	1.21	1.89	0.19	3.25	13.90	13.88	0.02
BH76	18.61	-0.63	1.49	0.08	1.95	7.87	7.83	0.04
ISO34	14.57	1.20	1.20	0.08	1.65	4.68	4.65	0.03
ICONF	3.27	0.01	0.38	0.12	0.55	1.25	1.20	0.05
ACONF	1.83	-0.20	0.20	0.11	0.23	0.48	0.45	0.03
TAUT15	3.05	0.62	0.62	0.20	0.74	1.45	1.37	0.08
Amino20x4	2.44	0.27	0.27	0.11	0.34	0.84	0.83	0.01
PCONF	1.62	0.59	0.91	0.56	1.08	2.19	2.12	0.07
MCONF	4.97	0.13	0.22	0.05	0.27	0.53	0.52	0.01

Continued on next page

Test set	$\lvert\overline{\Delta E}\rvert$	MD	MAD	NMAD	RMSD	Δ_{err}	max	min
SCONF	4.60	-0.01	0.24	0.05	0.37	1.15	1.13	0.02
PArel	4.63	0.22	0.88	0.19	1.35	3.34	3.31	0.03
BUT14DIOL	2.80	-0.00	0.12	0.04	0.17	0.57	0.57	0.00
EIE22	5.44	0.08	0.26	0.05	0.30	0.61	0.59	0.02
Styrene45	62.64	0.04	2.47	0.04	3.04	8.51	8.51	0.00
ISOMERIZATION20	31.84	0.86	1.24	0.04	1.69	3.86	3.78	0.08
DIE60	4.71	0.40	0.58	0.12	0.88	2.18	2.17	0.01
IDISP	14.22	1.71	1.71	0.12	2.56	5.41	5.39	0.02
C20C24	30.77	3.79	30.96	1.01	32.17	59.66	44.14	15.52
S66	5.47	-0.45	0.45	0.08	0.52	1.18	1.18	0.00
S10x8	6.59	0.32	0.37	0.06	0.46	1.00	0.99	0.01
X40	3.76	0.32	0.32	0.09	0.39	0.83	0.82	0.01
HEAVY28	1.24	-0.17	0.31	0.25	0.36	0.75	0.74	0.01
CHB6	26.79	-0.18	0.46	0.02	0.70	1.59	1.58	0.01
AHB21	22.49	-1.52	1.58	0.07	2.20	6.00	5.83	0.17
IL16	109.04	0.44	0.63	0.01	0.78	1.46	1.44	0.02
PNICO23	4.27	0.03	0.20	0.05	0.29	0.82	0.82	0.00
CT20	0.98	0.27	0.27	0.27	0.32	0.72	0.70	0.02
CARBHB12	6.04	0.40	0.41	0.07	0.55	1.28	1.26	0.02
ADIM6	3.36	-0.62	0.62	0.18	0.67	1.12	0.93	0.19
3B-69-TRIM	12.30	1.38	1.40	0.11	1.61	3.26	3.19	0.07
ISOL24	21.92	-1.16	2.73	0.12	4.39	16.97	16.89	0.08
C60ISO	98.25	8.56	8.56	0.09	9.08	16.35	12.57	3.78
L7	18.20	5.81	5.81	0.32	6.40	11.88	9.44	2.44
UPU23	5.72	0.62	0.71	0.12	0.90	2.46	2.27	0.19
ENZYMES23	15.32	-0.48	1.59	0.10	2.43	8.94	8.80	0.14

Table S116: Statistical analysis for M08-SO for all testset in our databsase. The numbers given (all in kcal/mol) are average reaction energy ($\lvert\overline{\Delta E}\rvert$), mean deviation (MD), mean absolute deviation (MAD), MAD normalized with respect to $\lvert\overline{\Delta E}\rvert$ (NMAD), root-mean-square deviation (RMSD), deviation span (Δ_{err}), maximum (max) and minimum deviation (min).

Test set	$\lvert\overline{\Delta E}\rvert$	MD	MAD	NMAD	RMSD	Δ_{err}	max	min
FH51	31.01	0.06	1.25	0.04	1.81	6.84	6.81	0.03
YBDE18	49.28	-4.31	4.31	0.09	4.63	7.96	7.10	0.86
AL2X6	35.88	0.11	0.68	0.02	0.86	1.63	1.56	0.07
DARC	32.47	0.31	0.57	0.02	0.69	1.47	1.39	0.08
NBPRC	27.71	-0.51	1.89	0.07	2.18	4.03	3.69	0.34
HEAVYSB9	58.02	-3.74	3.74	0.06	4.79	10.62	10.59	0.03
BSR36	16.20	-2.99	2.99	0.18	3.45	9.38	8.02	1.36
RSE43	7.60	-0.29	0.72	0.10	0.84	2.23	2.10	0.13
W4-11	306.91	-4.65	4.95	0.02	7.11	33.50	33.49	0.01

Continued on next page

Test set	$\lvert\overline{\Delta E}\rvert$	MD	MAD	NMAD	RMSD	Δ_{err}	max	min
G21EA	33.62	-0.77	2.78	0.08	3.47	8.81	8.40	0.41
G21IP	257.61	3.38	4.05	0.02	5.37	12.88	12.87	0.01
DIPCS10	654.26	3.06	4.03	0.01	4.98	10.07	9.56	0.51
PA26	189.05	-1.16	1.92	0.01	2.12	4.06	4.05	0.01
SIE4x4	33.72	10.02	10.02	0.30	11.92	26.83	24.98	1.85
ALKBDE10	100.69	40.15	40.15	0.40	45.66	85.80	71.78	14.02
RC21	35.70	0.54	1.48	0.04	1.75	3.49	3.31	0.18
ALK8	62.60	2.93	3.04	0.05	4.49	9.54	9.38	0.16
DC13	54.98	-2.37	8.84	0.16	11.76	23.14	22.75	0.39
G2RC	51.26	-1.67	2.43	0.05	3.41	11.96	11.46	0.50
BH76RC	21.39	-0.43	1.20	0.06	1.66	4.09	4.05	0.04
MOR23	35.57	4.09	5.15	0.14	6.14	14.86	14.45	0.41
WCPT18	34.99	0.24	1.14	0.03	1.47	3.47	3.42	0.05
BHROT27	6.37	0.13	0.30	0.05	0.40	0.89	0.89	0.00
BHPERI	20.87	1.05	1.34	0.06	2.05	5.27	5.27	0.00
BHDIV10	45.33	-0.09	0.80	0.02	1.24	3.28	3.26	0.02
INV24	32.85	-0.62	2.33	0.07	3.32	8.24	8.15	0.09
CR20	19.31	-3.28	3.28	0.17	3.39	5.97	4.59	1.38
CRBH20	46.13	-1.32	1.35	0.03	1.50	2.21	2.20	0.01
TMBH17	12.76	4.18	5.28	0.41	6.75	14.96	14.11	0.85
LTMBH26	9.98	0.76	1.92	0.19	3.72	16.12	15.98	0.14
BH76	18.61	-0.60	1.30	0.07	1.85	8.55	8.52	0.03
ISO34	14.57	-0.66	0.94	0.06	1.30	3.35	3.33	0.02
ICONF	3.27	-0.03	0.58	0.18	0.79	2.04	2.02	0.02
ACONF	1.83	-0.35	0.35	0.19	0.40	0.78	0.72	0.06
TAUT15	3.05	0.09	0.53	0.17	0.71	1.89	1.82	0.07
Amino20x4	2.44	-0.01	0.40	0.16	0.52	1.60	1.59	0.01
PCONF	1.62	0.54	0.75	0.46	0.96	1.86	1.78	0.08
MCONF	4.97	-0.04	0.26	0.05	0.32	0.68	0.67	0.01
SCONF	4.60	0.19	0.63	0.14	0.79	2.33	2.19	0.14
PArel	4.63	0.30	0.83	0.18	1.29	3.46	3.44	0.02
BUT14DIOL	2.80	-0.07	0.14	0.05	0.22	0.81	0.81	0.00
EIE22	5.44	0.01	0.24	0.04	0.26	0.48	0.46	0.02
Styrene45	62.64	-0.76	1.79	0.03	2.47	9.10	9.10	0.00
ISOMERIZATION20	31.84	1.47	1.58	0.05	2.10	4.78	4.71	0.07
DIE60	4.71	0.37	0.62	0.13	0.95	2.39	2.39	0.00
IDISP	14.22	-0.29	1.94	0.14	2.22	4.38	3.76	0.62
C20C24	30.77	3.14	22.93	0.75	23.81	44.07	32.50	11.57
S66	5.47	-0.58	0.58	0.11	0.66	1.51	1.49	0.02
S10x8	6.59	0.52	0.52	0.08	0.66	2.11	2.10	0.01
X40	3.76	0.38	0.39	0.10	0.49	1.62	1.58	0.04
HEAVY28	1.24	-0.00	0.22	0.18	0.29	0.73	0.72	0.01
CHB6	26.79	-0.52	0.67	0.02	1.06	2.49	2.45	0.04
AHB21	22.49	-0.72	0.80	0.04	1.14	3.14	3.13	0.01

Continued on next page

Test set	$\|\overline{\Delta E}\|$	MD	MAD	NMAD	RMSD	Δ_{err}	max	min
IL16	109.04	1.56	1.65	0.02	1.74	3.19	2.48	0.71
PNICO23	4.27	0.23	0.47	0.11	0.60	1.61	1.60	0.01
CT20	0.98	0.41	0.41	0.42	0.46	0.74	0.74	0.00
CARBHB12	6.04	0.14	0.47	0.08	0.58	1.32	1.28	0.04
ADIM6	3.36	-0.42	0.42	0.12	0.44	0.67	0.55	0.12
3B-69-TRIM	12.30	1.85	1.86	0.15	2.06	4.21	4.07	0.14
ISOL24	21.92	-0.60	2.02	0.09	3.07	11.65	11.55	0.10
C60ISO	98.25	7.51	7.51	0.08	8.01	14.41	11.13	3.28
L7	18.20	6.48	6.48	0.36	7.06	13.74	10.81	2.93
UPU23	5.72	0.78	0.83	0.14	1.05	2.48	2.38	0.10
ENZYMES23	15.32	-0.20	1.90	0.12	2.96	9.83	9.75	0.08

Table S117: Statistical analysis for M08-HX for all testset in our databsase. The numbers given (all in kcal/mol) are average reaction energy ($|\overline{\Delta E}|$), mean deviation (MD), mean absolute deviation (MAD), MAD normalized with respect to $|\overline{\Delta E}|$ (NMAD), root-mean-square deviation (RMSD), deviation span (Δ_{err}), maximum (max) and minimum deviation (min).

Test set	$\|\overline{\Delta E}\|$	MD	MAD	NMAD	RMSD	Δ_{err}	max	min
FH51	31.01	-0.27	1.41	0.05	1.92	6.44	6.42	0.02
YBDE18	49.28	15.34	15.58	0.32	21.17	32.02	31.95	0.07
AL2X6	35.88	4.69	4.69	0.13	4.70	9.08	5.10	3.98
DARC	32.47	-3.96	3.96	0.12	4.40	8.59	6.68	1.91
NBPRC	27.71	-1.01	2.97	0.11	3.34	6.54	5.71	0.83
HEAVYSB9	58.02	-3.90	3.90	0.07	5.30	13.85	13.17	0.68
BSR36	16.20	-2.54	2.54	0.16	3.09	8.47	7.98	0.49
RSE43	7.60	-0.04	0.55	0.07	0.68	1.23	1.21	0.02
W4-11	306.91	-4.08	4.40	0.01	6.61	36.70	36.57	0.13
G21EA	33.62	-1.40	2.88	0.09	3.62	8.67	8.18	0.49
G21IP	257.61	3.46	3.69	0.01	4.94	14.97	14.85	0.12
DIPCS10	654.26	3.88	4.05	0.01	4.58	9.59	8.76	0.83
PA26	189.05	-0.77	1.11	0.01	1.52	4.22	4.13	0.09
SIE4x4	33.72	8.09	8.09	0.24	9.65	24.04	21.89	2.15
ALKBDE10	100.69	-4.16	4.25	0.04	6.00	15.67	15.22	0.45
RC21	35.70	0.59	1.32	0.04	1.73	3.82	3.80	0.02
ALK8	62.60	1.90	2.66	0.04	3.88	8.97	8.72	0.25
DC13	54.98	-1.48	8.81	0.16	11.87	26.45	26.44	0.01
G2RC	51.26	-2.31	3.53	0.07	4.66	14.85	14.58	0.27
BH76RC	21.39	-0.23	1.16	0.05	1.58	4.09	4.04	0.05
MOR23	35.57	4.30	6.93	0.19	8.15	18.56	16.88	1.68
WCPT18	34.99	0.97	1.53	0.04	1.83	3.47	3.44	0.03
BHROT27	6.37	0.29	0.41	0.06	0.53	1.17	1.16	0.01
BHPERI	20.87	0.28	1.69	0.08	2.06	5.11	4.98	0.13

Continued on next page

Test set	$\|\overline{\Delta E}\|$	MD	MAD	NMAD	RMSD	Δ_{err}	max	min
BHDIV10	45.33	-0.16	1.06	0.02	1.32	2.71	2.55	0.16
INV24	32.85	0.23	2.08	0.06	3.32	11.65	11.53	0.12
CR20	19.31	1.01	1.11	0.06	1.25	2.38	2.15	0.23
CRBH20	46.13	2.86	2.86	0.06	2.96	5.41	4.14	1.27
TMBH17	12.76	4.78	5.92	0.46	7.67	15.88	15.65	0.23
LTMBH26	9.98	1.05	2.14	0.21	3.58	14.56	14.25	0.31
BH76	18.61	-0.14	1.15	0.06	1.80	10.08	10.05	0.03
ISO34	14.57	0.91	0.91	0.06	1.25	3.20	3.12	0.08
ICONF	3.27	-0.07	0.60	0.18	0.79	1.97	1.94	0.03
ACONF	1.83	-0.38	0.38	0.21	0.43	0.83	0.72	0.11
TAUT15	3.05	0.12	0.59	0.19	0.81	1.86	1.86	0.00
Amino20x4	2.44	0.02	0.37	0.15	0.50	1.32	1.32	0.00
PCONF	1.62	0.75	0.97	0.60	1.14	2.29	2.10	0.19
MCONF	4.97	0.17	0.35	0.07	0.41	0.92	0.91	0.01
SCONF	4.60	0.11	0.27	0.06	0.40	1.17	1.16	0.01
PArel	4.63	0.28	0.84	0.18	1.27	3.17	3.13	0.04
BUT14DIOL	2.80	-0.23	0.23	0.08	0.29	0.85	0.82	0.03
EIE22	5.44	0.18	0.34	0.06	0.40	0.82	0.78	0.04
Styrene45	62.64	0.61	1.99	0.03	2.43	6.41	6.41	0.00
ISOMERIZATION20	31.84	1.47	1.89	0.06	3.00	11.30	11.14	0.16
DIE60	4.71	0.51	0.73	0.15	0.99	2.49	2.49	0.00
IDISP	14.22	-0.18	2.75	0.19	3.10	4.04	3.95	0.09
C20C24	30.77	5.22	18.00	0.58	18.48	30.87	21.50	9.37
S66	5.47	-0.50	0.50	0.09	0.57	1.28	1.25	0.03
S10x8	6.59	0.43	0.44	0.07	0.54	1.65	1.64	0.01
X40	3.76	0.48	0.48	0.13	0.58	1.45	1.39	0.06
HEAVY28	1.24	0.76	0.78	0.63	0.92	1.96	1.94	0.02
CHB6	26.79	-0.81	0.95	0.04	1.40	3.28	3.14	0.14
AHB21	22.49	-0.86	0.90	0.04	1.32	3.83	3.81	0.02
IL16	109.04	1.02	1.03	0.01	1.19	1.98	1.95	0.03
PNICO23	4.27	0.26	0.46	0.11	0.55	1.41	1.34	0.07
CT20	0.98	0.42	0.42	0.42	0.44	0.68	0.66	0.02
CARBHB12	6.04	0.30	0.55	0.09	0.59	1.30	1.05	0.25
ADIM6	3.36	-0.76	0.76	0.23	0.80	1.37	1.05	0.32
3B-69-TRIM	12.30	1.90	1.91	0.16	2.16	4.61	4.48	0.13
ISOL24	21.92	-0.10	2.22	0.10	3.52	12.96	12.90	0.06
C60ISO	98.25	9.03	9.03	0.09	9.62	17.91	13.97	3.94
L7	18.20	6.99	6.99	0.38	7.59	13.94	10.90	3.04
UPU23	5.72	0.79	0.92	0.16	1.21	3.08	2.95	0.13
ENZYMES23	15.32	0.39	1.50	0.10	2.08	5.98	5.96	0.02

Table S118: Statistical analysis for MN12-SX for all testset in our databsase. The numbers given (all in kcal/mol) are average reaction energy ($|\overline{\Delta E}|$), mean deviation (MD), mean absolute deviation (MAD), MAD normalized with respect to $|\overline{\Delta E}|$ (NMAD), root-mean-square deviation (RMSD), deviation span (Δ_{err}), maximum (max) and minimum deviation (min).

Test set	$\|\overline{\Delta E}\|$	MD	MAD	NMAD	RMSD	Δ_{err}	max	min
FH51	31.01	1.35	2.07	0.07	2.98	10.20	10.12	0.08
YBDE18	49.28	-3.49	4.05	0.08	4.87	10.00	9.62	0.38
AL2X6	35.88	-3.25	3.25	0.09	3.40	6.09	4.44	1.65
DARC	32.47	4.56	4.56	0.14	5.00	7.77	6.56	1.21
NBPRC	27.71	0.96	2.75	0.10	3.10	6.31	5.52	0.79
HEAVYSB9	58.02	23.51	23.51	0.41	25.47	52.09	43.04	9.05
BSR36	16.20	-3.54	3.54	0.22	4.04	10.18	9.21	0.97
RSE43	7.60	-1.65	1.65	0.22	1.91	5.97	5.52	0.45
W4-11	306.91	-1.17	3.30	0.01	5.59	35.40	35.39	0.01
G21EA	33.62	-4.70	4.94	0.15	7.02	24.35	24.05	0.30
G21IP	257.61	-0.08	3.86	0.01	4.92	10.74	10.70	0.04
DIPCS10	654.26	-6.61	6.98	0.01	9.46	19.98	19.70	0.28
PA26	189.05	-0.32	1.63	0.01	1.87	4.04	3.90	0.14
SIE4x4	33.72	8.27	8.68	0.26	10.54	20.03	19.42	0.61
ALKBDE10	100.69	45.50	45.50	0.45	51.78	102.63	84.27	18.36
RC21	35.70	0.57	1.31	0.04	1.78	5.17	5.17	0.00
ALK8	62.60	-4.67	6.77	0.11	9.19	19.98	19.93	0.05
DC13	54.98	-0.98	10.03	0.18	12.27	26.82	25.81	1.01
G2RC	51.26	0.04	2.51	0.05	2.93	7.30	6.88	0.42
BH76RC	21.39	-0.16	1.68	0.08	2.36	7.01	7.00	0.01
MOR23	35.57	3.33	4.37	0.12	5.12	9.79	9.33	0.46
WCPT18	34.99	0.53	0.93	0.03	1.07	2.06	1.94	0.12
BHROT27	6.37	0.57	0.60	0.09	0.87	2.41	2.40	0.01
BHPERI	20.87	2.40	2.70	0.13	3.10	5.87	5.58	0.29
BHDIV10	45.33	0.33	1.63	0.04	1.74	2.53	2.42	0.11
INV24	32.85	-0.37	1.38	0.04	2.12	7.69	7.62	0.07
CR20	19.31	-4.21	4.21	0.22	4.80	8.78	8.21	0.57
CRBH20	46.13	-4.00	4.00	0.09	4.22	8.98	7.13	1.85
TMBH17	12.76	3.19	3.71	0.29	5.49	15.27	15.03	0.24
LTMBH26	9.98	1.66	4.70	0.47	14.69	73.97	73.87	0.10
BH76	18.61	-0.49	1.40	0.08	2.01	9.16	9.13	0.03
ISO34	14.57	-0.30	1.22	0.08	1.61	4.50	4.46	0.04
ICONF	3.27	-0.04	0.35	0.11	0.50	1.55	1.51	0.04
ACONF	1.83	0.03	0.05	0.03	0.06	0.15	0.14	0.01
TAUT15	3.05	0.78	1.43	0.47	1.76	3.22	3.22	0.00
Amino20x4	2.44	0.01	0.47	0.19	0.60	1.49	1.48	0.01
PCONF	1.62	0.85	0.90	0.55	1.09	2.58	2.48	0.10
MCONF	4.97	-0.19	0.38	0.08	0.45	1.10	1.08	0.02

Continued on next page

Test set	$\|\overline{\Delta E}\|$	MD	MAD	NMAD	RMSD	Δ_{err}	max	min
SCONF	4.60	-0.26	0.45	0.10	0.53	1.27	1.26	0.01
PArel	4.63	1.05	1.36	0.29	2.24	6.83	6.78	0.05
BUT14DIOL	2.80	-0.56	0.56	0.20	0.57	0.81	0.73	0.08
EIE22	5.44	1.21	1.23	0.23	1.35	2.73	2.48	0.25
Styrene45	62.64	0.56	2.31	0.04	2.90	9.87	9.87	0.00
ISOMERIZATION20	31.84	0.20	3.22	0.10	4.30	11.69	11.65	0.04
DIE60	4.71	1.47	1.49	0.32	1.70	3.26	3.23	0.03
IDISP	14.22	2.53	3.60	0.25	6.68	16.19	16.07	0.12
C20C24	30.77	-8.11	29.18	0.95	35.10	76.41	63.38	13.03
S66	5.47	-1.31	1.31	0.24	1.38	2.94	2.45	0.49
S10x8	6.59	1.09	1.09	0.17	1.22	2.30	2.24	0.06
X40	3.76	1.07	1.07	0.28	1.15	2.78	2.27	0.51
HEAVY28	1.24	-0.43	0.43	0.35	0.47	0.90	0.88	0.02
CHB6	26.79	1.28	1.28	0.05	1.38	2.77	2.17	0.60
AHB21	22.49	0.31	0.78	0.03	0.88	1.98	1.80	0.18
IL16	109.04	1.90	1.90	0.02	1.98	3.91	2.91	1.00
PNICO23	4.27	-0.56	0.57	0.13	0.70	1.54	1.50	0.04
CT20	0.98	0.83	0.83	0.84	0.85	1.50	1.14	0.36
CARBHB12	6.04	-0.60	0.60	0.10	0.74	1.81	1.68	0.13
ADIM6	3.36	-1.32	1.32	0.39	1.38	2.47	1.79	0.68
3B-69-TRIM	12.30	3.25	3.25	0.26	3.42	7.32	6.13	1.19
ISOL24	21.92	-1.61	4.08	0.19	5.50	15.32	15.23	0.09
C60ISO	98.25	0.93	2.47	0.03	2.67	3.85	3.68	0.17
L7	18.20	8.84	8.84	0.49	9.55	19.04	14.93	4.11
UPU23	5.72	0.26	0.72	0.13	0.98	2.63	2.62	0.01
ENZYMES23	15.32	-3.34	3.98	0.26	6.57	22.07	22.06	0.01

S5.7 D3-Dispersion-Corrected (meta-)GGAs

Table S119: Statistical analysis for BLYP-D3(BJ) for all testset in our databsase. The numbers given (all in kcal/mol) are average reaction energy ($|\overline{\Delta E}|$), mean deviation (MD), mean absolute deviation (MAD), MAD normalized with respect to $|\overline{\Delta E}|$ (NMAD), root-mean-square deviation (RMSD), deviation span (Δ_{err}), maximum (max) and minimum deviation (min).

Test set	$\|\overline{\Delta E}\|$	MD	MAD	NMAD	RMSD	Δ_{err}	max	min
FH51	31.01	4.73	4.73	0.15	6.21	18.41	18.33	0.08
YBDE18	49.28	-6.07	7.52	0.15	9.68	19.57	19.52	0.05
AL2X6	35.88	-4.65	4.65	0.13	4.91	10.71	7.49	3.22
DARC	32.47	13.50	13.50	0.42	13.63	24.75	15.14	9.61
NBPRC	27.71	2.39	3.96	0.14	5.53	14.01	13.85	0.16
HEAVYSB9	58.02	-3.40	4.16	0.07	4.91	10.63	9.49	1.14

Continued on next page

Test set	$\lvert\overline{\Delta E}\rvert$	MD	MAD	NMAD	RMSD	Δ_{err}	max	min
BSR36	16.20	-2.46	2.46	0.15	2.93	7.81	6.93	0.88
RSE43	7.60	-2.60	2.60	0.34	2.96	7.33	6.92	0.41
W4-11	306.91	4.88	6.82	0.02	9.75	37.41	37.31	0.10
G21EA	33.62	-4.12	4.66	0.14	5.16	10.66	9.52	1.14
G21IP	257.61	-1.54	4.62	0.02	5.53	13.17	12.81	0.36
DIPCS10	654.26	-4.97	6.94	0.01	8.03	12.18	12.10	0.08
PA26	189.05	1.61	2.61	0.01	3.45	8.82	8.74	0.08
SIE4x4	33.72	24.73	24.73	0.73	28.06	55.61	52.50	3.11
ALKBDE10	100.69	53.85	53.85	0.53	59.87	109.78	88.87	20.91
RC21	35.70	1.09	4.22	0.12	4.92	11.59	10.70	0.89
ALK8	62.60	-0.65	2.35	0.04	3.12	7.88	7.11	0.77
DC13	54.98	4.99	16.91	0.31	19.90	38.50	35.84	2.66
G2RC	51.26	4.35	5.35	0.10	6.69	16.50	16.18	0.32
BH76RC	21.39	-0.05	2.86	0.13	3.75	10.79	10.69	0.10
MOR23	35.57	-0.91	4.84	0.14	7.63	25.32	25.27	0.05
WCPT18	34.99	5.80	5.80	0.17	6.27	13.09	11.22	1.87
BHROT27	6.37	0.45	0.45	0.07	0.58	1.36	1.35	0.01
BHPERI	20.87	3.55	3.55	0.17	4.29	11.43	11.39	0.04
BHDIV10	45.33	6.06	6.06	0.13	7.12	12.31	11.45	0.86
INV24	32.85	2.32	2.32	0.07	2.99	8.43	7.96	0.47
CR20	19.31	-10.23	10.23	0.53	10.33	19.42	12.65	6.77
CRBH20	46.13	-16.82	16.82	0.36	16.87	33.28	19.37	13.91
TMBH17	12.76	-1.34	5.51	0.43	7.07	14.24	14.17	0.07
LTMBH26	9.98	-5.12	6.24	0.63	7.52	16.31	16.15	0.16
BH76	18.61	-9.76	9.76	0.52	10.85	24.75	24.33	0.42
ISO34	14.57	2.79	2.79	0.19	3.96	12.00	11.90	0.10
ICONF	3.27	0.33	0.33	0.10	0.41	0.95	0.93	0.02
ACONF	1.83	0.15	0.15	0.08	0.20	0.50	0.49	0.01
TAUT15	3.05	1.70	1.70	0.56	1.96	3.34	3.33	0.01
Amino20x4	2.44	0.34	0.34	0.14	0.44	1.53	1.53	0.00
PCONF	1.62	0.81	0.81	0.50	0.94	2.01	1.85	0.16
MCONF	4.97	0.38	0.38	0.08	0.46	1.06	1.06	0.00
SCONF	4.60	0.76	0.76	0.16	1.09	3.32	3.29	0.03
PArel	4.63	-0.51	1.76	0.38	2.69	8.04	8.01	0.03
BUT14DIOL	2.80	0.50	0.51	0.18	0.57	1.42	1.30	0.12
EIE22	5.44	1.71	1.73	0.32	1.92	3.83	3.72	0.11
Styrene45	62.64	4.16	7.03	0.11	9.47	25.31	25.31	0.00
ISOMERIZATION20	31.84	0.86	3.33	0.10	4.62	11.82	11.78	0.04
DIE60	4.71	1.43	1.44	0.30	1.54	3.07	3.06	0.01
IDISP	14.22	4.74	4.74	0.33	7.85	19.05	18.52	0.53
C20C24	30.77	1.32	52.63	1.71	55.63	119.15	82.88	36.27
S66	5.47	-0.20	0.20	0.04	0.25	0.66	0.66	0.00
S10x8	6.59	-0.01	0.21	0.03	0.26	0.61	0.60	0.01
X40	3.76	0.25	0.25	0.07	0.33	0.93	0.93	0.00

Continued on next page

Test set	$\lvert\overline{\Delta E}\rvert$	MD	MAD	NMAD	RMSD	Δ_{err}	max	min
HEAVY28	1.24	0.44	0.46	0.37	0.53	1.28	1.25	0.03
CHB6	26.79	0.87	1.12	0.04	1.45	3.16	2.96	0.20
AHB21	22.49	-0.40	0.66	0.03	0.78	1.86	1.81	0.05
IL16	109.04	-0.40	0.56	0.01	0.68	1.56	1.48	0.08
PNICO23	4.27	0.49	0.56	0.13	0.76	1.96	1.95	0.01
CT20	0.98	0.15	0.34	0.35	0.41	0.83	0.83	0.00
CARBHB12	6.04	1.17	1.17	0.19	1.47	3.33	3.04	0.29
ADIM6	3.36	0.24	0.24	0.07	0.29	0.46	0.46	0.00
3B-69-TRIM	12.30	0.43	0.59	0.05	0.77	1.91	1.91	0.00
ISOL24	21.92	-4.19	9.09	0.41	12.55	31.03	30.99	0.04
C60ISO	98.25	-8.64	8.64	0.09	10.68	19.36	18.86	0.50
L7	18.20	-0.40	0.85	0.05	1.05	2.34	2.17	0.17
UPU23	5.72	0.49	0.67	0.12	0.80	1.83	1.82	0.01
ENZYMES23	15.32	-5.32	6.96	0.45	8.37	15.94	15.88	0.06

Table S120: Statistical analysis for PBE-D3(BJ) for all testset in our databsase. The numbers given (all in kcal/mol) are average reaction energy ($\lvert\overline{\Delta E}\rvert$), mean deviation (MD), mean absolute deviation (MAD), MAD normalized with respect to $\lvert\overline{\Delta E}\rvert$ (NMAD), root-mean-square deviation (RMSD), deviation span (Δ_{err}), maximum (max) and minimum deviation (min).

Test set	$\lvert\overline{\Delta E}\rvert$	MD	MAD	NMAD	RMSD	Δ_{err}	max	min
FH51	31.01	0.97	3.14	0.10	4.45	12.79	12.66	0.13
YBDE18	49.28	-0.82	5.00	0.10	5.81	11.54	10.96	0.58
AL2X6	35.88	-1.11	1.96	0.05	2.83	6.12	5.89	0.23
DARC	32.47	2.02	3.27	0.10	3.70	5.13	4.96	0.17
NBPRC	27.71	-1.71	2.56	0.09	3.27	6.56	6.25	0.31
HEAVYSB9	58.02	-0.59	2.85	0.05	3.20	6.68	6.17	0.51
BSR36	16.20	-2.90	2.90	0.18	3.54	9.87	8.95	0.92
RSE43	7.60	-2.74	2.74	0.36	3.09	7.73	7.33	0.40
W4-11	306.91	12.89	14.09	0.05	17.53	52.72	52.69	0.03
G21EA	33.62	-2.09	3.22	0.10	4.02	9.07	8.69	0.38
G21IP	257.61	0.45	3.76	0.01	4.77	10.95	10.87	0.08
DIPCS10	654.26	-0.88	3.54	0.01	4.73	10.77	10.06	0.71
PA26	189.05	0.10	1.62	0.01	2.19	5.92	5.86	0.06
SIE4x4	33.72	27.64	27.64	0.82	29.41	58.64	46.89	11.75
ALKBDE10	100.69	55.14	55.14	0.55	61.29	116.45	92.07	24.38
RC21	35.70	5.84	6.83	0.19	7.75	13.80	13.47	0.33
ALK8	62.60	4.25	4.65	0.07	6.11	12.11	11.94	0.17
DC13	54.98	0.85	8.05	0.15	11.75	31.00	30.84	0.16
G2RC	51.26	0.57	6.72	0.13	8.15	20.39	20.15	0.24
BH76RC	21.39	0.14	3.61	0.17	4.64	14.16	14.04	0.12
MOR23	35.57	-2.36	4.36	0.12	5.76	17.18	16.99	0.19

Continued on next page

Test set	$\lvert\overline{\Delta E}\rvert$	MD	MAD	NMAD	RMSD	Δ_{err}	max	min
WCPT18	34.99	-9.49	9.49	0.27	9.98	22.22	17.13	5.09
BHROT27	6.37	0.34	0.47	0.07	0.65	1.69	1.68	0.01
BHPERI	20.87	-6.56	6.56	0.31	6.78	13.27	9.70	3.57
BHDIV10	45.33	-8.25	8.74	0.19	9.78	17.22	15.81	1.41
INV24	32.85	-0.96	2.63	0.08	4.13	15.68	15.60	0.08
CR20	19.31	-0.70	1.40	0.07	1.77	3.74	3.63	0.11
CRBH20	46.13	-7.99	7.99	0.17	8.12	16.17	10.86	5.31
TMBH17	12.76	-2.29	4.82	0.38	5.87	10.96	10.91	0.05
LTMBH26	9.98	-3.72	4.91	0.49	5.76	12.90	12.55	0.35
BH76	18.61	-10.01	10.03	0.54	11.29	32.37	31.32	1.05
ISO34	14.57	-0.70	1.51	0.10	2.11	6.97	6.85	0.12
ICONF	3.27	0.18	0.32	0.10	0.44	0.95	0.95	0.00
ACONF	1.83	-0.03	0.08	0.04	0.10	0.23	0.21	0.02
TAUT15	3.05	0.24	1.85	0.61	2.28	5.05	4.97	0.08
Amino20x4	2.44	0.04	0.37	0.15	0.46	1.13	1.13	0.00
PCONF	1.62	-0.60	1.28	0.79	1.55	2.95	2.83	0.12
MCONF	4.97	0.01	0.46	0.09	0.51	0.94	0.93	0.01
SCONF	4.60	0.44	1.02	0.22	1.23	3.16	3.16	0.00
PArel	4.63	0.21	1.82	0.39	2.53	6.48	6.43	0.05
BUT14DIOL	2.80	0.65	0.65	0.23	0.69	1.33	1.31	0.02
EIE22	5.44	1.82	1.87	0.34	2.10	3.96	3.74	0.22
Styrene45	62.64	-0.36	2.42	0.04	3.47	12.45	12.45	0.00
ISOMERIZATION20	31.84	0.79	3.29	0.10	4.63	12.37	12.36	0.01
DIE60	4.71	1.80	1.80	0.38	1.94	3.78	3.55	0.23
IDISP	14.22	2.50	2.53	0.18	4.13	9.37	9.34	0.03
C20C24	30.77	-13.18	15.76	0.51	25.43	49.28	49.15	0.13
S66	5.47	0.04	0.29	0.05	0.40	1.32	1.31	0.01
S10x8	6.59	-0.29	0.37	0.06	0.53	1.66	1.66	0.00
X40	3.76	-0.14	0.33	0.09	0.54	2.35	2.35	0.00
HEAVY28	1.24	0.52	0.52	0.42	0.61	1.55	1.47	0.08
CHB6	26.79	0.65	0.66	0.02	0.96	2.11	2.10	0.01
AHB21	22.49	-1.87	1.87	0.08	2.17	4.50	4.36	0.14
IL16	109.04	-1.65	1.65	0.02	1.84	3.94	3.31	0.63
PNICO23	4.27	1.24	1.24	0.29	1.79	6.23	6.09	0.14
CT20	0.98	-0.17	0.27	0.27	0.41	1.24	1.22	0.02
CARBHB12	6.04	2.00	2.00	0.33	2.38	5.21	4.57	0.64
ADIM6	3.36	0.40	0.40	0.12	0.40	0.83	0.49	0.34
3B-69-TRIM	12.30	0.07	0.73	0.06	0.94	2.50	2.47	0.03
ISOL24	21.92	-1.93	4.43	0.20	6.46	17.03	16.91	0.12
C60ISO	98.25	-9.50	9.50	0.10	11.62	21.29	20.48	0.81
L7	18.20	3.28	3.84	0.21	4.63	9.49	8.22	1.27
UPU23	5.72	0.39	0.58	0.10	0.71	1.79	1.77	0.02
ENZYMES23	15.32	-3.89	5.81	0.38	6.81	13.55	12.34	1.21

Table S121: Statistical analysis for REVPBE-D3(BJ) for all testset in our databsase. The numbers given (all in kcal/mol) are average reaction energy ($|\overline{\Delta E}|$), mean deviation (MD), mean absolute deviation (MAD), MAD normalized with respect to $|\overline{\Delta E}|$ (NMAD), root-mean-square deviation (RMSD), deviation span (Δ_{err}), maximum (max) and minimum deviation (min).

Test set	$\lvert\overline{\Delta E}\rvert$	MD	MAD	NMAD	RMSD	Δ_{err}	max	min
FH51	31.01	2.04	3.32	0.11	4.50	12.77	12.64	0.13
YBDE18	49.28	4.78	4.78	0.10	5.45	10.84	9.73	1.11
AL2X6	35.88	7.76	7.76	0.22	8.60	14.93	13.23	1.70
DARC	32.47	3.65	3.67	0.11	4.27	5.92	5.84	0.08
NBPRC	27.71	-4.56	6.61	0.24	8.08	18.97	17.82	1.15
HEAVYSB9	58.02	-2.77	3.20	0.06	4.14	8.84	8.60	0.24
BSR36	16.20	-1.48	1.48	0.09	1.68	4.21	3.84	0.37
RSE43	7.60	-2.10	2.10	0.28	2.41	6.41	6.28	0.13
W4-11	306.91	1.56	6.81	0.02	9.18	32.72	32.70	0.02
G21EA	33.62	-3.66	4.16	0.12	5.27	10.33	10.32	0.01
G21IP	257.61	-0.62	3.98	0.02	4.86	9.41	9.27	0.14
DIPCS10	654.26	-2.46	3.72	0.01	4.97	10.44	10.32	0.12
PA26	189.05	3.55	3.56	0.02	4.43	10.29	10.28	0.01
SIE4x4	33.72	22.79	22.79	0.68	25.83	52.38	48.47	3.91
ALKBDE10	100.69	1.18	5.09	0.05	7.75	22.21	21.76	0.45
RC21	35.70	3.53	4.65	0.13	5.39	9.47	9.40	0.07
ALK8	62.60	3.48	3.66	0.06	5.16	10.85	10.58	0.27
DC13	54.98	2.66	9.34	0.17	11.06	24.87	22.95	1.92
G2RC	51.26	2.76	5.87	0.11	7.40	19.87	19.82	0.05
BH76RC	21.39	0.29	2.29	0.11	3.41	11.05	10.97	0.08
MOR23	35.57	0.99	6.80	0.19	13.53	57.88	57.44	0.44
WCPT18	34.99	-7.31	7.31	0.21	7.78	17.76	14.10	3.66
BHROT27	6.37	0.20	0.41	0.06	0.53	1.29	1.24	0.05
BHPERI	20.87	-6.16	6.16	0.30	6.45	12.79	9.67	3.12
BHDIV10	45.33	-7.23	7.66	0.17	8.60	15.85	13.73	2.12
INV24	32.85	-1.21	2.17	0.07	2.87	7.29	7.21	0.08
CR20	19.31	9.24	9.24	0.48	9.51	18.02	12.41	5.61
CRBH20	46.13	-4.05	4.05	0.09	4.43	6.73	6.34	0.39
TMBH17	12.76	-1.90	4.22	0.33	5.27	11.36	11.24	0.12
LTMBH26	9.98	-4.81	5.79	0.58	6.74	13.99	13.60	0.39
BH76	18.61	-8.56	8.58	0.46	9.55	25.99	25.77	0.22
ISO34	14.57	-0.72	1.51	0.10	2.18	7.31	7.29	0.02
ICONF	3.27	0.07	0.31	0.10	0.40	0.95	0.94	0.01
ACONF	1.83	-0.11	0.11	0.06	0.15	0.33	0.33	0.00
TAUT15	3.05	-0.27	1.57	0.51	1.80	3.07	2.87	0.20
Amino20x4	2.44	-0.12	0.37	0.15	0.46	1.21	1.20	0.01
PCONF	1.62	-0.62	0.89	0.55	1.12	2.32	2.29	0.03
MCONF	4.97	0.09	0.45	0.09	0.54	1.09	1.09	0.00

Continued on next page

Test set	$\|\overline{\Delta E}\|$	MD	MAD	NMAD	RMSD	Δ_{err}	max	min
SCONF	4.60	0.09	0.62	0.13	0.86	2.50	2.39	0.11
PArel	4.63	0.01	1.54	0.33	2.37	7.13	7.06	0.07
BUT14DIOL	2.80	-0.01	0.30	0.11	0.37	1.04	1.02	0.02
EIE22	5.44	1.60	1.66	0.31	1.85	3.55	3.34	0.21
Styrene45	62.64	0.37	2.72	0.04	3.47	12.50	12.50	0.00
ISOMERIZATION20	31.84	-1.88	6.83	0.21	12.99	52.66	52.59	0.07
DIE60	4.71	1.64	1.64	0.35	1.75	3.53	3.40	0.13
IDISP	14.22	2.87	2.87	0.20	3.76	7.27	6.95	0.32
C20C24	30.77	-17.36	17.36	0.56	19.64	28.90	25.69	3.21
S66	5.47	-4.69	4.69	0.86	5.17	13.24	12.16	1.08
S10x8	6.59	0.23	0.37	0.06	0.53	1.24	1.24	0.00
X40	3.76	0.23	0.39	0.10	0.52	1.30	1.29	0.01
HEAVY28	1.24	1.42	1.46	1.18	1.62	2.83	2.80	0.03
CHB6	26.79	1.88	1.88	0.07	2.20	5.11	4.26	0.85
AHB21	22.49	-0.14	0.80	0.04	1.04	2.52	2.50	0.02
IL16	109.04	-0.42	0.69	0.01	0.86	1.89	1.84	0.05
PNICO23	4.27	0.77	0.91	0.21	1.37	4.84	4.75	0.09
CT20	0.98	0.24	0.34	0.35	0.39	0.82	0.69	0.13
CARBHB12	6.04	1.23	1.23	0.20	1.64	4.00	3.59	0.41
ADIM6	3.36	0.46	0.46	0.14	0.48	0.91	0.62	0.29
3B-69-TRIM	12.30	0.99	1.12	0.09	1.39	2.90	2.90	0.00
ISOL24	21.92	-2.07	4.72	0.22	6.52	16.48	16.35	0.13
C60ISO	98.25	-9.17	9.17	0.09	11.35	20.76	20.19	0.57
L7	18.20	0.48	1.09	0.06	1.47	2.94	2.93	0.01
UPU23	5.72	0.32	0.52	0.09	0.67	1.58	1.57	0.01
ENZYMES23	15.32	-4.14	5.69	0.37	6.77	14.22	13.67	0.55

Table S122: Statistical analysis for TPSS-D3(BJ) for all testset in our databsase. The numbers given (all in kcal/mol) are average reaction energy ($|\overline{\Delta E}|$), mean deviation (MD), mean absolute deviation (MAD), MAD normalized with respect to $|\overline{\Delta E}|$ (NMAD), root-mean-square deviation (RMSD), deviation span (Δ_{err}), maximum (max) and minimum deviation (min).

Test set	$\|\overline{\Delta E}\|$	MD	MAD	NMAD	RMSD	Δ_{err}	max	min
FH51	31.01	2.65	4.22	0.14	5.58	15.12	15.06	0.06
YBDE18	49.28	-4.89	5.30	0.11	5.83	12.16	10.54	1.62
AL2X6	35.88	0.53	2.32	0.06	2.60	5.57	4.48	1.09
DARC	32.47	4.80	4.80	0.15	5.28	7.73	7.09	0.64
NBPRC	27.71	-1.69	2.15	0.08	2.58	5.16	4.87	0.29
HEAVYSB9	58.02	-1.21	2.73	0.05	3.06	5.97	5.15	0.82
BSR36	16.20	-4.19	4.19	0.26	4.71	12.11	10.87	1.24
RSE43	7.60	-1.81	1.81	0.24	2.12	6.04	5.69	0.35
W4-11	306.91	2.75	4.81	0.02	6.32	24.40	24.37	0.03

Continued on next page

Test set	$\|\overline{\Delta E}\|$	MD	MAD	NMAD	RMSD	Δ_{err}	max	min
G21EA	33.62	-3.60	4.31	0.13	5.61	11.24	11.19	0.05
G21IP	257.61	-0.17	3.67	0.01	4.52	11.67	11.48	0.19
DIPCS10	654.26	-1.57	2.57	0.00	3.22	6.84	6.38	0.46
PA26	189.05	2.85	2.90	0.02	3.77	10.01	9.96	0.05
SIE4x4	33.72	21.14	21.14	0.63	24.23	49.18	46.16	3.02
ALKBDE10	100.69	50.40	50.40	0.50	56.19	110.68	87.35	23.33
RC21	35.70	3.33	4.39	0.12	4.94	8.85	8.07	0.78
ALK8	62.60	4.50	4.50	0.07	5.84	9.79	9.09	0.70
DC13	54.98	1.13	8.70	0.16	11.60	26.49	25.70	0.79
G2RC	51.26	3.22	6.76	0.13	8.71	21.97	20.80	1.17
BH76RC	21.39	-0.01	3.16	0.15	4.34	10.05	10.01	0.04
MOR23	35.57	-4.09	5.16	0.15	6.39	17.74	17.66	0.08
WCPT18	34.99	-6.50	6.50	0.19	6.90	14.08	11.34	2.74
BHROT27	6.37	0.33	0.54	0.08	0.71	1.66	1.63	0.03
BHPERI	20.87	-5.42	5.42	0.26	5.64	11.19	8.28	2.91
BHDIV10	45.33	-6.57	6.87	0.15	7.56	14.39	12.89	1.50
INV24	32.85	-1.34	1.95	0.06	2.37	5.40	5.09	0.31
CR20	19.31	-2.78	2.91	0.15	3.19	5.15	5.01	0.14
CRBH20	46.13	-10.26	10.26	0.22	10.29	20.90	12.02	8.88
TMBH17	12.76	-1.84	4.57	0.36	5.65	11.15	11.07	0.08
LTMBH26	9.98	-3.38	4.73	0.47	5.73	15.44	14.82	0.62
BH76	18.61	-9.40	9.40	0.51	10.36	23.37	23.30	0.07
ISO34	14.57	-1.59	2.33	0.16	3.25	12.62	12.46	0.16
ICONF	3.27	0.01	0.19	0.06	0.23	0.46	0.44	0.02
ACONF	1.83	-0.11	0.11	0.06	0.14	0.29	0.28	0.01
TAUT15	3.05	0.06	1.66	0.54	1.92	3.54	3.42	0.12
Amino20x4	2.44	0.03	0.35	0.14	0.42	0.91	0.91	0.00
PCONF	1.62	-0.54	1.08	0.66	1.34	2.49	2.40	0.09
MCONF	4.97	0.05	0.41	0.08	0.49	0.95	0.95	0.00
SCONF	4.60	0.51	1.31	0.28	1.58	3.79	3.77	0.02
PArel	4.63	0.05	1.56	0.34	2.23	6.06	6.03	0.03
BUT14DIOL	2.80	0.47	0.47	0.17	0.50	0.89	0.88	0.01
EIE22	5.44	1.92	1.93	0.35	2.13	3.89	3.79	0.10
Styrene45	62.64	0.67	3.04	0.05	3.73	12.42	12.42	0.00
ISOMERIZATION20	31.84	1.52	3.44	0.11	4.67	11.18	11.04	0.14
DIE60	4.71	1.79	1.79	0.38	1.89	3.63	3.31	0.32
IDISP	14.22	2.93	2.93	0.21	4.78	11.24	11.22	0.02
C20C24	30.77	-12.29	24.96	0.81	33.55	69.13	63.17	5.96
S66	5.47	-0.25	0.34	0.06	0.42	1.28	1.28	0.00
S10x8	6.59	-0.08	0.26	0.04	0.34	1.21	1.20	0.01
X40	3.76	0.11	0.30	0.08	0.43	1.69	1.68	0.01
HEAVY28	1.24	0.39	0.42	0.34	0.50	1.27	1.22	0.05
CHB6	26.79	0.92	0.92	0.03	1.24	2.64	2.54	0.10
AHB21	22.49	-1.13	1.22	0.05	1.46	2.98	2.84	0.14

Continued on next page

Test set	$\|\overline{\Delta E}\|$	MD	MAD	NMAD	RMSD	Δ_{err}	max	min
IL16	109.04	-0.54	0.57	0.01	0.75	1.69	1.68	0.01
PNICO23	4.27	1.06	1.11	0.26	1.47	4.51	4.39	0.12
CT20	0.98	0.13	0.25	0.26	0.30	0.58	0.56	0.02
CARBHB12	6.04	1.47	1.47	0.24	1.80	3.99	3.56	0.43
ADIM6	3.36	-0.19	0.23	0.07	0.29	0.61	0.57	0.04
3B-69-TRIM	12.30	0.62	0.85	0.07	1.08	2.96	2.92	0.04
ISOL24	21.92	-3.23	5.95	0.27	8.07	20.47	20.35	0.12
C60ISO	98.25	-7.82	7.82	0.08	9.87	18.13	17.96	0.17
L7	18.20	2.16	2.45	0.13	2.75	5.84	4.82	1.02
UPU23	5.72	0.40	0.55	0.10	0.67	1.46	1.46	0.00
ENZYMES23	15.32	-4.69	5.23	0.34	6.78	13.43	13.40	0.03

Table S123: Statistical analysis for revTPSS-D3(BJ) for all testset in our databsase. The numbers given (all in kcal/mol) are average reaction energy ($|\overline{\Delta E}|$), mean deviation (MD), mean absolute deviation (MAD), MAD normalized with respect to $|\overline{\Delta E}|$ (NMAD), root-mean-square deviation (RMSD), deviation span (Δ_{err}), maximum (max) and minimum deviation (min).

Test set	$\|\overline{\Delta E}\|$	MD	MAD	NMAD	RMSD	Δ_{err}	max	min
FH51	31.01	5.16	5.16	0.17	6.83	20.29	20.18	0.11
YBDE18	49.28	-4.63	4.89	0.10	5.51	9.79	9.49	0.30
AL2X6	35.88	0.60	1.97	0.06	2.17	4.03	3.44	0.59
DARC	32.47	3.41	3.72	0.11	4.09	6.24	5.39	0.85
NBPRC	27.71	-0.86	1.62	0.06	2.03	4.60	4.47	0.13
HEAVYSB9	58.02	-0.59	2.44	0.04	2.81	4.63	4.57	0.06
BSR36	16.20	-2.79	2.79	0.17	3.08	7.23	6.76	0.47
RSE43	7.60	-1.57	1.57	0.21	1.91	5.64	5.36	0.28
W4-11	306.91	1.63	4.95	0.02	6.70	24.91	24.86	0.05
G21EA	33.62	-4.71	5.13	0.15	6.52	13.33	13.11	0.22
G21IP	257.61	-0.95	3.68	0.01	4.56	11.23	11.16	0.07
DIPCS10	654.26	-3.03	3.16	0.00	4.34	9.01	8.96	0.05
PA26	189.05	3.49	3.55	0.02	4.52	11.40	11.16	0.24
SIE4x4	33.72	20.68	20.68	0.61	23.73	47.71	44.89	2.82
ALKBDE10	100.69	-0.29	4.42	0.04	6.56	18.31	18.17	0.14
RC21	35.70	3.54	4.78	0.13	5.49	10.73	10.09	0.64
ALK8	62.60	2.24	2.60	0.04	3.69	7.29	6.98	0.31
DC13	54.98	0.08	8.69	0.16	12.56	34.22	33.13	1.09
G2RC	51.26	5.51	9.96	0.19	12.78	28.28	28.17	0.11
BH76RC	21.39	0.37	4.44	0.21	5.61	13.45	13.26	0.19
MOR23	35.57	-0.09	7.10	0.20	13.44	59.59	59.19	0.40
WCPT18	34.99	5.56	5.56	0.16	5.93	11.93	9.44	2.49
BHROT27	6.37	0.51	0.51	0.08	0.70	1.53	1.53	0.00
BHPERI	20.87	5.72	5.72	0.27	6.00	11.16	8.73	2.43

Continued on next page

Test set	$\lvert\overline{\Delta E}\rvert$	MD	MAD	NMAD	RMSD	Δ_{err}	max	min
BHDIV10	45.33	6.03	6.03	0.13	6.73	12.45	11.93	0.52
INV24	32.85	1.48	1.48	0.04	1.97	5.15	4.83	0.32
CR20	19.31	0.34	1.16	0.06	1.69	5.29	5.17	0.12
CRBH20	46.13	-8.83	8.83	0.19	8.87	17.18	10.17	7.01
TMBH17	12.76	-1.87	4.56	0.36	5.42	11.28	10.66	0.62
LTMBH26	9.98	-3.74	4.85	0.49	5.75	14.80	14.43	0.37
BH76	18.61	-11.18	11.18	0.60	13.29	38.37	37.60	0.77
ISO34	14.57	2.86	2.86	0.20	4.17	15.92	15.85	0.07
ICONF	3.27	0.22	0.22	0.07	0.29	0.70	0.67	0.03
ACONF	1.83	0.07	0.07	0.04	0.09	0.19	0.18	0.01
TAUT15	3.05	1.32	1.32	0.43	1.73	3.54	3.50	0.04
Amino20x4	2.44	0.26	0.26	0.11	0.34	1.11	1.11	0.00
PCONF	1.62	0.85	0.85	0.53	1.04	1.94	1.82	0.12
MCONF	4.97	0.41	0.41	0.08	0.46	0.95	0.93	0.02
SCONF	4.60	1.23	1.23	0.27	1.39	2.80	2.76	0.04
PArel	4.63	0.31	1.53	0.33	2.03	5.55	5.46	0.09
BUT14DIOL	2.80	0.05	0.17	0.06	0.22	0.64	0.64	0.00
EIE22	5.44	1.84	1.84	0.34	2.00	3.59	3.57	0.02
Styrene45	62.64	-0.27	2.83	0.05	3.64	10.63	10.63	0.00
ISOMERIZATION20	31.84	2.07	3.95	0.12	5.33	12.19	12.18	0.01
DIE60	4.71	1.64	1.64	0.35	1.70	3.49	2.89	0.60
IDISP	14.22	3.00	3.00	0.21	4.85	9.92	9.88	0.04
C20C24	30.77	-18.39	18.39	0.60	27.52	55.01	54.44	0.57
S66	5.47	-0.58	0.58	0.11	0.67	1.90	1.90	0.00
S10x8	6.59	0.23	0.28	0.04	0.36	0.90	0.90	0.00
X40	3.76	0.43	0.43	0.11	0.51	1.71	1.62	0.09
HEAVY28	1.24	0.17	0.24	0.20	0.34	0.95	0.94	0.01
CHB6	26.79	1.06	1.06	0.04	1.33	2.61	2.58	0.03
AHB21	22.49	-0.62	0.71	0.03	0.88	1.76	1.67	0.09
IL16	109.04	-0.09	0.37	0.00	0.49	1.24	1.23	0.01
PNICO23	4.27	0.84	0.89	0.21	1.26	3.89	3.87	0.02
CT20	0.98	0.19	0.31	0.31	0.35	0.68	0.62	0.06
CARBHB12	6.04	0.98	0.98	0.16	1.18	2.83	2.52	0.31
ADIM6	3.36	-0.39	0.39	0.12	0.46	0.85	0.75	0.10
3B-69-TRIM	12.30	1.41	1.42	0.12	1.64	3.70	3.62	0.08
ISOL24	21.92	-3.04	5.32	0.24	6.81	18.00	17.82	0.18
C60ISO	98.25	-8.75	8.75	0.09	10.87	20.03	19.54	0.49
L7	18.20	4.55	4.55	0.25	5.25	10.22	8.93	1.29
UPU23	5.72	0.37	0.56	0.10	0.72	1.70	1.70	0.00
ENZYMES23	15.32	-4.27	5.89	0.38	7.20	12.91	12.43	0.48

Table S124: Statistical analysis for SCAN-D3(BJ) for all testset in our databsase. The numbers given (all in kcal/mol) are average reaction energy ($|\overline{\Delta E}|$), mean deviation (MD), mean absolute deviation (MAD), MAD normalized with respect to $|\overline{\Delta E}|$ (NMAD), root-mean-square deviation (RMSD), deviation span (Δ_{err}), maximum (max) and minimum deviation (min).

Test set	$\lvert\overline{\Delta E}\rvert$	MD	MAD	NMAD	RMSD	Δ_{err}	max	min
FH51	31.01	2.78	2.78	0.09	4.11	11.26	11.20	0.06
YBDE18	49.28	-3.41	3.99	0.08	4.22	7.45	6.22	1.23
AL2X6	35.88	2.04	2.04	0.06	2.41	3.24	3.24	0.00
DARC	32.47	-1.37	2.41	0.07	2.92	6.63	5.65	0.98
NBPRC	27.71	-1.04	1.87	0.07	2.27	4.66	4.24	0.42
HEAVYSB9	58.02	-2.37	2.47	0.04	3.01	5.56	5.27	0.29
BSR36	16.20	-1.03	1.04	0.06	1.50	4.78	4.77	0.01
RSE43	7.60	-1.17	1.18	0.16	1.75	5.61	5.60	0.01
W4-11	306.91	-1.65	3.48	0.01	4.81	26.93	26.91	0.02
G21EA	33.62	-3.61	5.47	0.16	6.69	13.68	13.32	0.36
G21IP	257.61	0.20	4.69	0.02	5.67	12.28	12.21	0.07
DIPCS10	654.26	-1.49	4.87	0.01	5.68	13.58	12.14	1.44
PA26	189.05	1.99	2.27	0.01	3.33	9.73	9.71	0.02
SIE4x4	33.72	2.47	25.59	0.76	32.32	88.46	85.91	2.55
ALKBDE10	100.69	1.07	5.55	0.06	7.56	20.54	19.71	0.83
RC21	35.70	5.06	5.68	0.16	6.30	11.26	10.46	0.80
ALK8	62.60	2.96	3.55	0.06	4.71	8.24	8.09	0.15
DC13	54.98	-0.32	7.70	0.14	10.85	29.93	29.63	0.30
G2RC	51.26	-1.25	6.14	0.12	7.63	18.43	18.37	0.06
BH76RC	21.39	-0.50	2.92	0.14	3.88	8.92	8.92	0.00
MOR23	35.57	-0.80	7.15	0.20	13.51	58.85	58.64	0.21
WCPT18	34.99	6.58	6.58	0.19	7.34	15.46	13.12	2.34
BHROT27	6.37	0.80	0.82	0.13	1.15	2.40	2.40	0.00
BHPERI	20.87	5.58	5.58	0.27	5.89	9.12	8.37	0.75
BHDIV10	45.33	-5.51	6.48	0.14	7.05	12.92	11.33	1.59
INV24	32.85	1.23	1.23	0.04	1.75	5.75	5.75	0.00
CR20	19.31	0.73	1.21	0.06	1.40	2.61	2.59	0.02
CRBH20	46.13	-6.57	6.57	0.14	6.60	13.51	7.98	5.53
TMBH17	12.76	-1.52	4.79	0.38	5.51	8.74	8.24	0.50
LTMBH26	9.98	-3.78	4.99	0.50	5.81	15.57	14.06	1.51
BH76	18.61	-8.09	8.17	0.44	8.93	18.85	18.38	0.47
ISO34	14.57	1.34	1.34	0.09	1.95	6.32	6.29	0.03
ICONF	3.27	0.35	0.35	0.11	0.46	0.96	0.95	0.01
ACONF	1.83	0.11	0.11	0.06	0.13	0.27	0.25	0.02
TAUT15	3.05	0.37	1.79	0.59	2.24	5.08	5.01	0.07
Amino20x4	2.44	0.14	0.23	0.10	0.33	0.96	0.96	0.00
PCONF	1.62	0.51	0.51	0.31	0.56	0.91	0.90	0.01
MCONF	4.97	0.59	0.59	0.12	0.73	1.27	1.25	0.02

Continued on next page

Test set	$\|\overline{\Delta E}\|$	MD	MAD	NMAD	RMSD	Δ_{err}	max	min
SCONF	4.60	0.89	0.89	0.19	0.97	1.89	1.83	0.06
PArel	4.63	0.59	1.50	0.32	2.19	7.23	7.19	0.04
BUT14DIOL	2.80	0.51	0.51	0.18	0.53	0.84	0.82	0.02
EIE22	5.44	2.02	2.04	0.37	2.23	4.12	3.90	0.22
Styrene45	62.64	2.08	2.73	0.04	3.52	9.53	9.53	0.00
ISOMERIZATION20	31.84	-0.23	2.60	0.08	3.50	6.74	6.62	0.12
DIE60	4.71	1.69	1.69	0.36	1.79	2.71	2.61	0.10
IDISP	14.22	2.06	2.06	0.14	3.40	8.13	8.03	0.10
C20C24	30.77	-9.81	15.23	0.49	22.24	43.99	42.36	1.63
S66	5.47	0.22	0.30	0.05	0.48	2.35	2.35	0.00
S10x8	6.59	-0.45	0.52	0.08	0.87	3.68	3.68	0.00
X40	3.76	-0.29	0.36	0.10	0.61	2.60	2.60	0.00
HEAVY28	1.24	-0.04	0.23	0.19	0.28	0.72	0.68	0.04
CHB6	26.79	1.21	1.21	0.05	1.34	2.40	2.01	0.39
AHB21	22.49	-2.55	2.55	0.11	2.98	5.77	5.47	0.30
IL16	109.04	-1.68	1.68	0.02	1.80	3.31	2.77	0.54
PNICO23	4.27	1.05	1.06	0.25	1.51	5.21	5.16	0.05
CT20	0.98	-0.17	0.21	0.22	0.33	1.12	1.10	0.02
CARBHB12	6.04	1.68	1.68	0.28	2.12	4.54	4.28	0.26
ADIM6	3.36	0.06	0.06	0.02	0.07	0.14	0.11	0.03
3B-69-TRIM	12.30	-0.48	0.80	0.07	1.10	2.82	2.81	0.01
ISOL24	21.92	-0.45	3.14	0.14	4.65	15.50	15.17	0.33
C60ISO	98.25	-5.41	5.55	0.06	7.42	14.72	14.38	0.34
L7	18.20	2.56	2.56	0.14	3.40	5.99	5.89	0.10
UPU23	5.72	-0.16	0.40	0.07	0.51	1.43	1.39	0.04
ENZYMES23	15.32	-3.60	8.35	0.54	14.68	49.61	49.58	0.03

S5.8 D4-Dispersion-Corrected (meta-)GGAs

Table S125: Statistical analysis for BLYP-D4 for all testset in our databsase. The numbers given (all in kcal/mol) are average reaction energy ($|\overline{\Delta E}|$), mean deviation (MD), mean absolute deviation (MAD), MAD normalized with respect to $|\overline{\Delta E}|$ (NMAD), root-mean-square deviation (RMSD), deviation span (Δ_{err}), maximum (max) and minimum deviation (min).

Test set	$\|\overline{\Delta E}\|$	MD	MAD	NMAD	RMSD	Δ_{err}	max	min
FH51	31.01	5.41	6.53	0.21	8.39	24.76	24.58	0.18
YBDE18	49.28	-10.94	11.84	0.24	14.37	26.53	25.70	0.83
AL2X6	35.88	-11.88	11.88	0.33	12.48	24.23	17.56	6.67
DARC	32.47	22.36	22.36	0.69	22.64	41.38	25.82	15.56
NBPRC	27.71	4.53	4.78	0.17	6.84	15.08	14.93	0.15
HEAVYSB9	58.02	-9.64	9.64	0.17	10.47	20.76	16.24	4.52

Continued on next page

Test set	$\|\overline{\Delta E}\|$	MD	MAD	NMAD	RMSD	Δ_{err}	max	min
BSR36	16.20	-11.27	11.27	0.70	12.89	32.29	29.67	2.62
RSE43	7.60	-2.90	2.90	0.38	3.26	8.26	7.57	0.69
W4-11	306.91	2.60	6.97	0.02	9.87	35.98	35.97	0.01
G21EA	33.62	-4.12	4.67	0.14	5.16	10.66	9.52	1.14
G21IP	257.61	-1.53	4.61	0.02	5.52	13.15	12.79	0.36
DIPCS10	654.26	-5.00	6.97	0.01	8.06	12.27	12.19	0.08
PA26	189.05	0.82	2.42	0.01	3.11	8.30	8.03	0.27
SIE4x4	33.72	24.20	24.20	0.72	27.70	55.30	52.44	2.86
ALKBDE10	100.69	52.74	52.74	0.52	59.00	106.52	88.12	18.40
RC21	35.70	-1.43	3.95	0.11	5.72	17.38	17.04	0.34
ALK8	62.60	-9.07	9.07	0.14	11.90	22.82	21.86	0.96
DC13	54.98	7.88	22.66	0.41	27.46	48.32	46.54	1.78
G2RC	51.26	5.00	6.00	0.12	7.54	15.83	15.71	0.12
BH76RC	21.39	-0.05	2.86	0.13	3.73	10.87	10.80	0.07
MOR23	35.57	11.24	12.96	0.36	15.26	28.33	27.17	1.16
WCPT18	34.99	-4.34	4.34	0.12	4.70	8.97	7.77	1.20
BHROT27	6.37	0.05	0.44	0.07	0.56	1.33	1.32	0.01
BHPERI	20.87	2.04	4.59	0.22	5.32	11.99	11.76	0.23
BHDIV10	45.33	-3.59	5.27	0.12	6.25	11.11	10.97	0.14
INV24	32.85	-2.33	3.52	0.11	4.13	10.70	9.71	0.99
CR20	19.31	-15.47	15.47	0.80	15.61	31.25	19.81	11.44
CRBH20	46.13	-16.99	16.99	0.37	17.06	33.84	19.93	13.91
TMBH17	12.76	-0.30	10.11	0.79	14.67	39.60	38.75	0.85
LTMBH26	9.98	-2.79	4.51	0.45	5.84	16.19	15.91	0.28
BH76	18.61	-8.79	8.81	0.47	10.02	23.98	23.93	0.05
ISO34	14.57	-0.89	3.36	0.23	4.73	13.27	13.23	0.04
ICONF	3.27	-0.03	0.68	0.21	0.92	2.14	2.11	0.03
ACONF	1.83	1.05	1.05	0.57	1.17	2.55	2.15	0.40
TAUT15	3.05	-0.22	1.64	0.54	1.88	2.95	2.94	0.01
Amino20x4	2.44	-0.17	0.77	0.31	0.97	2.48	2.46	0.02
PCONF	1.62	-0.76	4.62	2.85	5.15	8.75	8.13	0.62
MCONF	4.97	-2.84	2.90	0.58	3.27	5.02	4.96	0.06
SCONF	4.60	-0.19	0.51	0.11	0.67	1.44	1.44	0.00
PArel	4.63	-0.48	1.73	0.37	2.74	8.31	8.30	0.01
BUT14DIOL	2.80	-0.09	0.40	0.14	0.49	1.15	1.11	0.04
EIE22	5.44	2.00	2.00	0.37	2.20	3.81	3.79	0.02
Styrene45	62.64	2.62	7.52	0.12	10.22	25.17	25.17	0.00
ISOMERIZATION20	31.84	0.86	3.31	0.10	4.63	12.03	11.96	0.07
DIE60	4.71	1.69	1.70	0.36	1.86	3.73	3.62	0.11
IDISP	14.22	5.96	19.19	1.35	23.05	49.49	45.20	4.29
C20C24	30.77	6.57	62.76	2.04	65.00	137.98	91.87	46.11
S66	5.47	-0.19	0.34	0.06	0.40	0.85	0.85	0.00
S10x8	6.59	-0.03	0.31	0.05	0.38	0.96	0.95	0.01
X40	3.76	2.67	2.67	0.71	3.19	10.19	9.62	0.57

Continued on next page

Test set	$\overline{\|\Delta E\|}$	MD	MAD	NMAD	RMSD	Δ_{err}	max	min
HEAVY28	1.24	-1.49	1.49	1.20	1.59	3.20	2.68	0.52
CHB6	26.79	0.75	0.81	0.03	1.20	2.27	2.26	0.01
AHB21	22.49	-0.06	0.67	0.03	0.80	1.72	1.59	0.13
IL16	109.04	3.62	3.62	0.03	3.80	7.53	5.97	1.56
PNICO23	4.27	-2.22	2.22	0.52	2.35	5.70	4.36	1.34
CT20	0.98	0.08	0.28	0.28	0.36	1.02	1.02	0.00
CARBHB12	6.04	-0.44	0.79	0.13	0.96	1.63	1.61	0.02
ADIM6	3.36	-5.84	5.84	1.74	6.38	12.05	9.73	2.32
3B-69-TRIM	12.30	0.22	0.55	0.04	0.69	1.97	1.93	0.04
ISOL24	21.92	-3.95	8.29	0.38	11.45	27.71	27.70	0.01
C60ISO	98.25	-10.65	10.65	0.11	12.79	23.01	21.75	1.26
L7	18.20	-0.66	1.09	0.06	1.35	2.71	2.63	0.08
UPU23	5.72	0.47	0.64	0.11	0.75	1.89	1.82	0.07
ENZYMES23	15.32	-5.45	6.98	0.46	8.50	17.33	17.07	0.26

Table S126: Statistical analysis for PBE-D4 for all testset in our databsase. The numbers given (all in kcal/mol) are average reaction energy ($\overline{|\Delta E|}$), mean deviation (MD), mean absolute deviation (MAD), MAD normalized with respect to $\overline{|\Delta E|}$ (NMAD), root-mean-square deviation (RMSD), deviation span (Δ_{err}), maximum (max) and minimum deviation (min).

Test set	$\overline{\|\Delta E\|}$	MD	MAD	NMAD	RMSD	Δ_{err}	max	min
FH51	31.01	1.82	3.41	0.11	4.67	12.62	12.58	0.04
YBDE18	49.28	-3.13	6.06	0.12	7.31	14.52	13.65	0.87
AL2X6	35.88	-4.45	4.45	0.12	5.24	7.72	7.59	0.13
DARC	32.47	6.40	6.49	0.20	7.53	10.65	10.28	0.37
NBPRC	27.71	0.12	2.76	0.10	3.46	6.06	6.03	0.03
HEAVYSB9	58.02	-3.46	4.19	0.07	5.08	9.92	9.39	0.53
BSR36	16.20	-7.41	7.41	0.46	8.59	21.78	19.87	1.91
RSE43	7.60	-2.90	2.90	0.38	3.25	8.20	7.65	0.55
W4-11	306.91	11.97	13.44	0.04	16.84	52.00	51.91	0.09
G21EA	33.62	-2.30	3.27	0.10	4.08	9.29	8.85	0.44
G21IP	257.61	0.37	3.79	0.01	4.80	10.89	10.85	0.04
DIPCS10	654.26	-1.26	3.70	0.01	5.00	11.16	10.86	0.30
PA26	189.05	0.33	1.67	0.01	2.29	6.45	6.37	0.08
SIE4x4	33.72	22.83	22.83	0.68	25.82	49.64	46.85	2.79
ALKBDE10	100.69	54.69	54.69	0.54	60.92	114.93	91.54	23.39
RC21	35.70	4.45	5.46	0.15	6.36	13.00	12.32	0.68
ALK8	62.60	0.70	2.70	0.04	3.41	7.66	6.98	0.68
DC13	54.98	1.98	10.86	0.20	13.69	29.40	29.28	0.12
G2RC	51.26	0.90	6.09	0.12	7.52	20.15	19.94	0.21
BH76RC	21.39	0.15	3.58	0.17	4.62	14.17	14.09	0.08
MOR23	35.57	3.99	6.71	0.19	7.82	15.41	13.63	1.78

Continued on next page

Test set	$\lvert\overline{\Delta E}\rvert$	MD	MAD	NMAD	RMSD	Δ_{err}	max	min
WCPT18	34.99	-8.76	8.76	0.25	9.13	20.45	15.39	5.06
BHROT27	6.37	0.31	0.47	0.07	0.64	1.69	1.67	0.02
BHPERI	20.87	-3.82	3.82	0.18	4.28	9.35	8.32	1.03
BHDIV10	45.33	-7.55	8.08	0.18	9.08	15.20	13.90	1.30
INV24	32.85	-0.12	4.86	0.15	11.40	54.25	53.70	0.55
CR20	19.31	-3.18	3.18	0.16	3.63	6.15	6.15	0.00
CRBH20	46.13	-8.05	8.05	0.17	8.18	16.43	11.13	5.30
TMBH17	12.76	-2.98	9.31	0.73	13.80	34.41	34.27	0.14
LTMBH26	9.98	-3.15	4.40	0.44	5.36	13.35	13.13	0.22
BH76	18.61	-9.53	9.56	0.51	10.85	31.49	30.63	0.86
ISO34	14.57	-0.89	1.82	0.13	2.53	7.45	7.41	0.04
ICONF	3.27	0.22	0.46	0.14	0.63	1.88	1.83	0.05
ACONF	1.83	0.58	0.58	0.32	0.66	1.43	1.24	0.19
TAUT15	3.05	0.33	1.83	0.60	2.29	4.84	4.81	0.03
Amino20x4	2.44	-0.01	0.52	0.21	0.66	1.81	1.81	0.00
PCONF	1.62	-0.80	3.51	2.17	3.81	5.97	5.58	0.39
MCONF	4.97	-1.59	1.70	0.34	1.94	3.40	3.32	0.08
SCONF	4.60	0.26	0.43	0.09	0.60	1.30	1.30	0.00
PArel	4.63	0.23	1.83	0.40	2.55	6.66	6.63	0.03
BUT14DIOL	2.80	0.22	0.28	0.10	0.39	1.22	1.21	0.01
EIE22	5.44	1.97	2.00	0.37	2.24	3.92	3.80	0.12
Styrene45	62.64	-1.06	2.99	0.05	4.15	14.72	14.72	0.00
ISOMERIZATION20	31.84	0.79	3.30	0.10	4.64	12.38	12.35	0.03
DIE60	4.71	1.94	1.94	0.41	2.11	3.83	3.78	0.05
IDISP	14.22	2.99	10.79	0.76	12.62	26.13	23.66	2.47
C20C24	30.77	-13.70	23.09	0.75	32.85	67.36	63.07	4.29
S66	5.47	0.06	0.28	0.05	0.37	1.03	1.02	0.01
S10x8	6.59	-0.34	0.42	0.06	0.58	1.81	1.80	0.01
X40	3.76	1.20	1.28	0.34	1.77	6.36	6.36	0.00
HEAVY28	1.24	-0.37	0.48	0.39	0.56	1.27	1.18	0.09
CHB6	26.79	0.44	0.44	0.02	0.64	1.40	1.39	0.01
AHB21	22.49	-1.73	1.75	0.08	2.08	4.37	4.23	0.14
IL16	109.04	0.42	1.13	0.01	1.29	2.43	2.42	0.01
PNICO23	4.27	-0.07	0.79	0.19	1.19	4.39	4.32	0.07
CT20	0.98	-0.21	0.26	0.27	0.42	1.35	1.34	0.01
CARBHB12	6.04	1.23	1.23	0.20	1.69	3.99	3.87	0.12
ADIM6	3.36	-3.19	3.19	0.95	3.56	6.62	5.63	0.99
3B-69-TRIM	12.30	-0.18	0.64	0.05	0.87	2.56	2.55	0.01
ISOL24	21.92	-1.89	4.24	0.19	6.09	15.48	15.37	0.11
C60ISO	98.25	-10.57	10.57	0.11	12.76	23.26	22.06	1.20
L7	18.20	2.42	2.74	0.15	3.49	6.83	6.36	0.47
UPU23	5.72	0.37	0.57	0.10	0.69	1.81	1.77	0.04
ENZYMES23	15.32	-4.09	5.17	0.34	6.32	14.15	13.47	0.68

Table S127: Statistical analysis for REVPBE-D4 for all testset in our databsase. The numbers given (all in kcal/mol) are average reaction energy ($|\overline{\Delta E}|$), mean deviation (MD), mean absolute deviation (MAD), MAD normalized with respect to $|\overline{\Delta E}|$ (NMAD), root-mean-square deviation (RMSD), deviation span (Δ_{err}), maximum (max) and minimum deviation (min).

Test set	$\lvert\overline{\Delta E}\rvert$	MD	MAD	NMAD	RMSD	Δ_{err}	max	min
FH51	31.01	1.57	3.16	0.10	4.41	12.72	12.70	0.02
YBDE18	49.28	6.35	6.35	0.13	6.97	13.97	11.53	2.44
AL2X6	35.88	7.98	7.98	0.22	8.82	15.81	13.78	2.03
DARC	32.47	0.69	2.26	0.07	2.56	3.54	3.33	0.21
NBPRC	27.71	-5.61	6.87	0.25	8.05	14.29	12.76	1.53
HEAVYSB9	58.02	-3.10	3.31	0.06	4.54	9.68	9.53	0.15
BSR36	16.20	1.15	1.15	0.07	1.67	5.25	5.24	0.01
RSE43	7.60	-2.17	2.17	0.29	2.47	6.76	6.36	0.40
W4-11	306.91	2.27	6.91	0.02	9.30	34.09	34.07	0.02
G21EA	33.62	5.77	6.52	0.19	10.28	34.97	34.51	0.46
G21IP	257.61	-0.29	3.75	0.01	4.60	9.41	9.27	0.14
DIPCS10	654.26	-0.95	2.78	0.00	3.58	7.22	7.13	0.09
PA26	189.05	1.75	2.15	0.01	2.94	7.68	7.67	0.01
SIE4x4	33.72	22.66	22.66	0.67	25.73	51.96	48.38	3.58
ALKBDE10	100.69	1.49	5.26	0.05	8.07	23.57	22.79	0.78
RC21	35.70	4.10	5.15	0.14	5.84	11.11	10.16	0.95
ALK8	62.60	0.99	1.61	0.03	2.49	6.29	6.00	0.29
DC13	54.98	2.82	8.36	0.15	10.64	22.39	21.94	0.45
G2RC	51.26	2.53	6.44	0.13	8.04	20.57	20.50	0.07
BH76RC	21.39	0.31	2.30	0.11	3.43	11.27	11.21	0.06
MOR23	35.57	0.01	6.88	0.19	13.11	57.14	56.09	1.05
WCPT18	34.99	-7.75	7.75	0.22	8.24	18.74	14.96	3.78
BHROT27	6.37	0.25	0.45	0.07	0.60	1.29	1.29	0.00
BHPERI	20.87	-7.86	7.86	0.38	8.28	16.23	12.20	4.03
BHDIV10	45.33	-7.90	8.24	0.18	9.24	17.32	15.60	1.72
INV24	32.85	-1.40	1.98	0.06	2.72	6.93	6.93	0.00
CR20	19.31	12.19	12.19	0.63	12.38	24.19	15.24	8.95
CRBH20	46.13	-2.15	2.66	0.06	2.89	4.97	4.65	0.32
TMBH17	12.76	-2.68	5.56	0.44	6.24	12.85	11.25	1.60
LTMBH26	9.98	-4.56	5.68	0.57	6.50	13.47	13.38	0.09
BH76	18.61	-8.89	8.90	0.48	9.89	26.37	26.33	0.04
ISO34	14.57	-0.27	1.76	0.12	3.34	15.36	15.36	0.00
ICONF	3.27	-0.02	0.32	0.10	0.38	0.80	0.75	0.05
ACONF	1.83	-0.27	0.27	0.15	0.29	0.62	0.51	0.11
TAUT15	3.05	0.08	1.58	0.52	1.84	3.63	3.51	0.12
Amino20x4	2.44	-0.09	0.34	0.14	0.44	1.05	1.05	0.00
PCONF	1.62	-0.58	0.72	0.45	0.85	1.65	1.53	0.12
MCONF	4.97	0.08	0.45	0.09	0.55	1.09	1.05	0.04

Continued on next page

Test set	$\lvert\overline{\Delta E}\rvert$	MD	MAD	NMAD	RMSD	Δ_{err}	max	min
SCONF	4.60	0.39	0.98	0.21	1.18	2.97	2.89	0.08
PArel	4.63	0.20	1.53	0.33	2.30	6.31	6.17	0.14
BUT14DIOL	2.80	0.41	0.41	0.15	0.45	0.94	0.86	0.08
EIE22	5.44	1.64	1.70	0.31	1.91	4.08	3.73	0.35
Styrene45	62.64	0.57	2.32	0.04	3.20	10.00	10.00	0.00
ISOMERIZATION20	31.84	-1.82	6.91	0.22	13.03	52.69	52.60	0.09
DIE60	4.71	1.67	1.67	0.36	1.78	3.57	3.36	0.21
IDISP	14.22	1.43	2.20	0.16	3.54	8.66	8.24	0.42
C20C24	30.77	-25.45	32.20	1.05	35.81	54.39	45.62	8.77
S66	5.47	-0.39	0.44	0.08	0.53	1.32	1.27	0.05
S10x8	6.59	0.14	0.27	0.04	0.34	1.03	1.02	0.01
X40	3.76	0.23	0.46	0.12	0.61	2.04	1.99	0.05
HEAVY28	1.24	1.32	1.39	1.12	1.55	2.54	2.46	0.08
CHB6	26.79	1.12	1.29	0.05	1.41	2.86	2.36	0.50
AHB21	22.49	0.45	1.07	0.05	1.36	3.13	3.09	0.04
IL16	109.04	-0.12	0.55	0.01	0.68	1.39	1.36	0.03
PNICO23	4.27	0.94	1.00	0.23	1.48	5.09	5.07	0.02
CT20	0.98	0.11	0.25	0.26	0.31	0.85	0.83	0.02
CARBHB12	6.04	1.14	1.14	0.19	1.60	3.82	3.56	0.26
ADIM6	3.36	-0.18	0.24	0.07	0.28	0.58	0.51	0.07
3B-69-TRIM	12.30	0.71	0.88	0.07	1.03	2.14	2.13	0.01
ISOL24	21.92	-1.08	3.38	0.15	4.92	13.29	13.25	0.04
C60ISO	98.25	-11.27	11.27	0.11	13.58	24.65	23.29	1.36
L7	18.20	1.65	1.77	0.10	2.16	3.60	3.39	0.21
UPU23	5.72	0.31	0.51	0.09	0.62	1.44	1.43	0.01
ENZYMES23	15.32	-4.11	5.63	0.37	7.00	14.92	14.84	0.08

Table S128: Statistical analysis for TPSS-D4 for all testset in our databsase. The numbers given (all in kcal/mol) are average reaction energy ($\lvert\overline{\Delta E}\rvert$), mean deviation (MD), mean absolute deviation (MAD), MAD normalized with respect to $\lvert\overline{\Delta E}\rvert$ (NMAD), root-mean-square deviation (RMSD), deviation span (Δ_{err}), maximum (max) and minimum deviation (min).

Test set	$\lvert\overline{\Delta E}\rvert$	MD	MAD	NMAD	RMSD	Δ_{err}	max	min
FH51	31.01	2.57	4.17	0.13	5.56	15.09	15.08	0.01
YBDE18	49.28	-4.89	5.30	0.11	5.83	12.16	10.54	1.62
AL2X6	35.88	0.53	2.32	0.06	2.60	5.57	4.48	1.09
DARC	32.47	4.82	4.82	0.15	5.37	7.82	7.09	0.73
NBPRC	27.71	-1.62	2.08	0.07	2.55	5.41	4.82	0.59
HEAVYSB9	58.02	-1.56	2.62	0.05	3.00	5.68	5.28	0.40
BSR36	16.20	-2.96	2.96	0.18	3.24	8.21	7.46	0.75
RSE43	7.60	-1.85	1.85	0.24	2.15	6.23	5.76	0.47
W4-11	306.91	2.75	4.81	0.02	6.32	24.40	24.37	0.03

Continued on next page

Test set	$\|\overline{\Delta E}\|$	MD	MAD	NMAD	RMSD	Δ_{err}	max	min
G21EA	33.62	-3.60	4.31	0.13	5.61	11.24	11.19	0.05
G21IP	257.61	-0.17	3.67	0.01	4.52	11.67	11.48	0.19
DIPCS10	654.26	-1.57	2.57	0.00	3.22	6.84	6.38	0.46
PA26	189.05	2.85	2.90	0.02	3.77	10.01	9.96	0.05
SIE4x4	33.72	21.14	21.14	0.63	24.23	49.18	46.16	3.02
ALKBDE10	100.69	3.47	5.31	0.05	9.40	22.56	22.45	0.11
RC21	35.70	3.09	4.20	0.12	4.73	8.93	8.21	0.72
ALK8	62.60	2.71	2.71	0.04	3.58	6.50	6.01	0.49
DC13	54.98	1.13	8.70	0.16	11.60	26.49	25.70	0.79
G2RC	51.26	3.25	6.90	0.13	8.86	21.83	20.98	0.85
BH76RC	21.39	-0.00	3.17	0.15	4.35	10.14	10.07	0.07
MOR23	35.57	-3.88	4.99	0.14	6.25	17.74	17.66	0.08
WCPT18	34.99	-6.50	6.50	0.19	6.90	14.09	11.35	2.74
BHROT27	6.37	0.33	0.54	0.08	0.71	1.66	1.63	0.03
BHPERI	20.87	-5.79	5.79	0.28	6.04	11.87	8.87	3.00
BHDIV10	45.33	-6.57	6.87	0.15	7.56	14.39	12.89	1.50
INV24	32.85	-1.34	1.95	0.06	2.37	5.39	5.09	0.30
CR20	19.31	-2.01	2.24	0.12	2.47	4.35	4.08	0.27
CRBH20	46.13	-9.65	9.65	0.21	9.68	19.48	11.12	8.36
TMBH17	12.76	-2.11	5.69	0.45	6.39	12.12	11.06	1.06
LTMBH26	9.98	-3.38	4.73	0.47	5.73	15.44	14.82	0.62
BH76	18.61	-9.42	9.43	0.51	10.40	23.43	23.29	0.14
ISO34	14.57	-1.61	2.33	0.16	3.28	12.86	12.69	0.17
ICONF	3.27	-0.03	0.19	0.06	0.22	0.37	0.35	0.02
ACONF	1.83	-0.18	0.18	0.10	0.21	0.46	0.40	0.06
TAUT15	3.05	0.18	1.62	0.53	1.90	3.63	3.57	0.06
Amino20x4	2.44	0.05	0.37	0.15	0.45	1.11	1.11	0.00
PCONF	1.62	-0.46	0.83	0.51	1.04	2.17	2.07	0.10
MCONF	4.97	0.14	0.44	0.09	0.55	1.07	1.05	0.02
SCONF	4.60	0.51	1.31	0.28	1.58	3.80	3.78	0.02
PArel	4.63	0.12	1.54	0.33	2.19	5.74	5.71	0.03
BUT14DIOL	2.80	0.47	0.47	0.17	0.50	0.90	0.88	0.02
EIE22	5.44	1.92	1.94	0.36	2.14	4.11	3.98	0.13
Styrene45	62.64	0.57	2.86	0.05	3.59	11.88	11.88	0.00
ISOMERIZATION20	31.84	1.53	3.45	0.11	4.69	11.17	11.07	0.10
DIE60	4.71	1.82	1.82	0.39	1.92	3.71	3.36	0.35
IDISP	14.22	2.37	2.48	0.17	3.82	8.78	8.46	0.32
C20C24	30.77	-16.82	18.29	0.59	29.81	58.56	58.52	0.04
S66	5.47	-0.21	0.36	0.07	0.42	1.04	1.00	0.04
S10x8	6.59	-0.14	0.30	0.05	0.42	1.50	1.50	0.00
X40	3.76	0.00	0.41	0.11	0.64	2.24	2.22	0.02
HEAVY28	1.24	0.39	0.43	0.35	0.52	1.26	1.22	0.04
CHB6	26.79	0.73	0.73	0.03	0.93	2.07	1.85	0.22
AHB21	22.49	-0.97	1.17	0.05	1.40	2.82	2.69	0.13

Continued on next page

Test set	$\|\overline{\Delta E}\|$	MD	MAD	NMAD	RMSD	Δ_{err}	max	min
IL16	109.04	-0.42	0.51	0.00	0.64	1.56	1.46	0.10
PNICO23	4.27	1.06	1.12	0.26	1.48	4.50	4.39	0.11
CT20	0.98	0.06	0.21	0.21	0.27	0.75	0.74	0.01
CARBHB12	6.04	1.47	1.47	0.24	1.80	3.99	3.56	0.43
ADIM6	3.36	-0.19	0.23	0.07	0.29	0.61	0.57	0.04
3B-69-TRIM	12.30	0.31	0.68	0.06	0.88	2.31	2.30	0.01
ISOL24	21.92	-3.04	5.41	0.25	7.30	18.30	18.12	0.18
C60ISO	98.25	-9.16	9.16	0.09	11.29	20.61	19.94	0.67
L7	18.20	1.23	1.23	0.07	1.58	2.86	2.73	0.13
UPU23	5.72	0.37	0.52	0.09	0.63	1.45	1.44	0.01
ENZYMES23	15.32	-4.86	12.17	0.79	24.27	78.90	78.71	0.19

Table S129: Statistical analysis for revTPSS-D4 for all testset in our databsase. The numbers given (all in kcal/mol) are average reaction energy ($|\overline{\Delta E}|$), mean deviation (MD), mean absolute deviation (MAD), MAD normalized with respect to $|\overline{\Delta E}|$ (NMAD), root-mean-square deviation (RMSD), deviation span (Δ_{err}), maximum (max) and minimum deviation (min).

Test set	$\|\overline{\Delta E}\|$	MD	MAD	NMAD	RMSD	Δ_{err}	max	min
FH51	31.01	3.12	5.12	0.17	6.84	20.26	20.10	0.16
YBDE18	49.28	-4.43	4.72	0.10	5.31	9.64	9.41	0.23
AL2X6	35.88	0.90	2.22	0.06	2.33	4.47	3.29	1.18
DARC	32.47	2.66	3.15	0.10	3.44	4.96	4.59	0.37
NBPRC	27.71	-0.97	1.64	0.06	2.13	4.51	4.41	0.10
HEAVYSB9	58.02	-0.67	2.27	0.04	2.57	4.83	4.13	0.70
BSR36	16.20	-1.46	1.46	0.09	1.58	3.06	3.05	0.01
RSE43	7.60	-1.60	1.60	0.21	1.94	5.74	5.42	0.32
W4-11	306.91	1.62	4.92	0.02	6.68	25.03	25.03	0.00
G21EA	33.62	-2.18	7.30	0.22	10.94	35.40	35.09	0.31
G21IP	257.61	-0.87	3.62	0.01	4.50	11.23	11.16	0.07
DIPCS10	654.26	-2.63	2.96	0.00	3.94	8.32	7.93	0.39
PA26	189.05	2.86	3.02	0.02	3.97	10.39	10.26	0.13
SIE4x4	33.72	20.60	20.60	0.61	23.68	47.54	44.85	2.69
ALKBDE10	100.69	-0.23	4.46	0.04	6.65	18.59	18.50	0.09
RC21	35.70	3.51	4.75	0.13	5.44	10.73	10.18	0.55
ALK8	62.60	1.22	1.81	0.03	2.48	5.18	5.17	0.01
DC13	54.98	0.30	8.65	0.16	12.30	31.31	31.15	0.16
G2RC	51.26	5.56	10.09	0.20	12.92	28.85	28.65	0.20
BH76RC	21.39	0.37	4.45	0.21	5.62	13.44	13.31	0.13
MOR23	35.57	-1.20	7.76	0.22	13.68	58.73	58.58	0.15
WCPT18	34.99	-5.56	5.56	0.16	5.92	11.90	9.41	2.49
BHROT27	6.37	0.30	0.51	0.08	0.69	1.52	1.51	0.01
BHPERI	20.87	-6.06	6.06	0.29	6.38	11.76	9.33	2.43

Continued on next page

Test set	$\|\overline{\Delta E}\|$	MD	MAD	NMAD	RMSD	Δ_{err}	max	min
BHDIV10	45.33	-5.98	6.07	0.13	6.78	12.52	12.08	0.44
INV24	32.85	-1.13	1.55	0.05	2.01	5.13	4.87	0.26
CR20	19.31	0.94	1.19	0.06	1.80	5.59	5.53	0.06
CRBH20	46.13	-8.40	8.40	0.18	8.44	16.54	9.81	6.73
TMBH17	12.76	-2.17	5.52	0.43	6.16	11.68	10.74	0.94
LTMBH26	9.98	-3.20	4.51	0.45	5.44	14.85	14.39	0.46
BH76	18.61	-10.91	10.91	0.59	13.13	38.20	37.61	0.59
ISO34	14.57	-2.27	2.87	0.20	4.23	16.19	16.08	0.11
ICONF	3.27	-0.00	0.24	0.07	0.30	0.67	0.66	0.01
ACONF	1.83	-0.17	0.17	0.09	0.19	0.34	0.31	0.03
TAUT15	3.05	0.16	1.29	0.42	1.69	3.50	3.50	0.00
Amino20x4	2.44	0.08	0.29	0.12	0.36	1.07	1.07	0.00
PCONF	1.62	-0.30	0.63	0.39	0.72	1.34	1.32	0.02
MCONF	4.97	0.18	0.65	0.13	1.17	5.52	5.52	0.00
SCONF	4.60	0.64	1.36	0.30	1.55	3.20	3.17	0.03
PArel	4.63	0.35	1.50	0.32	2.00	5.24	5.24	0.00
BUT14DIOL	2.80	0.23	0.24	0.09	0.28	0.65	0.61	0.04
EIE22	5.44	1.83	1.84	0.34	2.01	3.82	3.74	0.08
Styrene45	62.64	-0.38	2.85	0.05	3.70	10.26	10.26	0.00
ISOMERIZATION20	31.84	2.08	3.97	0.12	5.35	12.27	12.27	0.00
DIE60	4.71	1.66	1.66	0.35	1.72	3.58	2.94	0.64
IDISP	14.22	2.34	3.03	0.21	4.44	8.97	8.84	0.13
C20C24	30.77	-22.00	22.00	0.71	26.71	59.68	50.33	9.35
S66	5.47	-0.28	0.34	0.06	0.38	0.84	0.84	0.00
S10x8	6.59	0.03	0.19	0.03	0.23	0.50	0.50	0.00
X40	3.76	0.19	0.33	0.09	0.45	1.67	1.66	0.01
HEAVY28	1.24	0.26	0.34	0.28	0.42	1.00	0.95	0.05
CHB6	26.79	0.84	0.84	0.03	1.01	1.91	1.86	0.05
AHB21	22.49	-0.51	0.70	0.03	0.85	1.64	1.61	0.03
IL16	109.04	-0.14	0.35	0.00	0.49	1.44	1.43	0.01
PNICO23	4.27	0.98	1.02	0.24	1.34	4.06	3.94	0.12
CT20	0.98	0.10	0.24	0.25	0.30	0.81	0.77	0.04
CARBHB12	6.04	1.00	1.00	0.17	1.19	2.85	2.51	0.34
ADIM6	3.36	-0.18	0.18	0.05	0.23	0.41	0.41	0.00
3B-69-TRIM	12.30	0.65	0.75	0.06	0.91	2.04	1.98	0.06
ISOL24	21.92	-2.89	4.87	0.22	6.03	15.53	15.30	0.23
C60ISO	98.25	-9.88	9.88	0.10	12.09	22.12	21.24	0.88
L7	18.20	1.40	1.40	0.08	1.69	2.91	2.73	0.18
UPU23	5.72	0.19	0.39	0.07	0.49	1.14	1.09	0.05
ENZYMES23	15.32	-4.49	5.94	0.39	7.37	13.87	13.59	0.28

Table S130: Statistical analysis for SCAN-D4 for all testset in our databsase. The numbers given (all in kcal/mol) are average reaction energy ($|\overline{\Delta E}|$), mean deviation (MD), mean absolute deviation (MAD), MAD normalized with respect to $|\overline{\Delta E}|$ (NMAD), root-mean-square deviation (RMSD), deviation span (Δ_{err}), maximum (max) and minimum deviation (min).

Test set	$\|\overline{\Delta E}\|$	MD	MAD	NMAD	RMSD	Δ_{err}	max	min
FH51	31.01	-0.70	2.72	0.09	4.05	11.16	11.09	0.07
YBDE18	49.28	-3.62	4.17	0.08	4.42	8.00	6.67	1.33
AL2X6	35.88	1.81	1.89	0.05	2.19	3.34	3.13	0.21
DARC	32.47	-1.01	2.45	0.08	2.82	6.75	5.40	1.35
NBPRC	27.71	-2.50	2.50	0.09	3.12	5.77	5.29	0.48
HEAVYSB9	58.02	-2.92	2.92	0.05	3.36	6.01	5.64	0.37
BSR36	16.20	-1.35	1.35	0.08	1.77	5.16	5.11	0.05
RSE43	7.60	-1.19	1.21	0.16	1.77	5.68	5.65	0.03
W4-11	306.91	-1.73	3.52	0.01	4.83	26.96	26.95	0.01
G21EA	33.62	-1.52	7.56	0.22	11.34	39.29	39.00	0.29
G21IP	257.61	0.21	4.69	0.02	5.67	12.29	12.21	0.08
DIPCS10	654.26	-1.44	4.86	0.01	5.66	13.62	12.14	1.48
PA26	189.05	-0.41	4.36	0.02	11.76	57.85	57.70	0.15
SIE4x4	33.72	2.42	25.55	0.76	32.31	88.50	85.94	2.56
ALKBDE10	100.69	1.05	5.56	0.06	7.56	20.53	19.71	0.82
RC21	35.70	4.90	5.50	0.15	6.12	11.05	10.30	0.75
ALK8	62.60	2.64	3.23	0.05	4.34	7.68	7.66	0.02
DC13	54.98	-0.15	7.78	0.14	10.85	30.61	29.42	1.19
G2RC	51.26	-1.21	6.08	0.12	7.55	18.12	18.00	0.12
BH76RC	21.39	-0.50	2.92	0.14	3.87	8.90	8.89	0.01
MOR23	35.57	-0.51	6.88	0.19	13.42	59.02	58.77	0.25
WCPT18	34.99	-6.50	6.50	0.19	7.24	15.26	12.92	2.34
BHROT27	6.37	0.79	0.82	0.13	1.15	2.40	2.39	0.01
BHPERI	20.87	-5.31	5.31	0.25	5.61	8.88	8.16	0.72
BHDIV10	45.33	-5.45	6.42	0.14	6.99	12.78	11.18	1.60
INV24	32.85	-0.56	1.23	0.04	1.75	5.85	5.84	0.01
CR20	19.31	0.57	1.17	0.06	1.35	2.61	2.49	0.12
CRBH20	46.13	-6.52	6.52	0.14	6.55	13.42	7.96	5.46
TMBH17	12.76	-1.52	5.38	0.42	5.96	9.07	8.23	0.84
LTMBH26	9.98	-3.63	5.07	0.51	5.92	15.62	14.11	1.51
BH76	18.61	-8.04	8.12	0.44	8.89	18.78	18.30	0.48
ISO34	14.57	-0.27	1.37	0.09	1.98	6.37	6.36	0.01
ICONF	3.27	0.31	0.34	0.11	0.46	1.04	1.03	0.01
ACONF	1.83	0.19	0.19	0.10	0.21	0.46	0.40	0.06
TAUT15	3.05	0.40	1.79	0.59	2.25	5.10	5.01	0.09
Amino20x4	2.44	0.14	0.24	0.10	0.34	1.00	1.00	0.00
PCONF	1.62	-0.08	0.44	0.27	0.49	0.83	0.79	0.04
MCONF	4.97	0.45	0.50	0.10	0.65	1.12	1.12	0.00

Continued on next page

Test set	$\vert\overline{\Delta E}\vert$	MD	MAD	NMAD	RMSD	Δ_{err}	max	min
SCONF	4.60	0.53	0.84	0.18	0.91	1.70	1.64	0.06
PArel	4.63	0.60	1.49	0.32	2.19	7.24	7.24	0.00
BUT14DIOL	2.80	0.48	0.48	0.17	0.50	0.81	0.78	0.03
EIE22	5.44	2.04	2.06	0.38	2.26	4.14	3.93	0.21
Styrene45	62.64	2.01	2.70	0.04	3.50	9.44	9.44	0.00
ISOMERIZATION20	31.84	-0.22	2.60	0.08	3.50	6.73	6.61	0.12
DIE60	4.71	1.71	1.71	0.36	1.82	2.77	2.64	0.13
IDISP	14.22	1.51	2.37	0.17	3.72	8.68	8.53	0.15
C20C24	30.77	-10.21	15.08	0.49	22.42	44.03	42.89	1.14
S66	5.47	0.01	0.34	0.06	0.48	2.23	2.22	0.01
S10x8	6.59	-0.35	0.50	0.08	0.83	3.54	3.54	0.00
X40	3.76	-0.19	0.42	0.11	0.64	2.46	2.46	0.00
HEAVY28	1.24	-0.10	0.26	0.21	0.29	0.71	0.62	0.09
CHB6	26.79	1.20	1.20	0.04	1.32	2.43	2.00	0.43
AHB21	22.49	-2.47	2.47	0.11	2.93	5.61	5.40	0.21
IL16	109.04	-1.48	1.48	0.01	1.63	2.97	2.60	0.37
PNICO23	4.27	0.95	0.96	0.23	1.43	5.05	5.03	0.02
CT20	0.98	-0.14	0.20	0.21	0.31	1.06	1.06	0.00
CARBHB12	6.04	1.60	1.60	0.26	2.04	4.37	4.16	0.21
ADIM6	3.36	-0.30	0.30	0.09	0.33	0.54	0.47	0.07
3B-69-TRIM	12.30	-0.24	0.80	0.06	1.03	2.57	2.56	0.01
ISOL24	21.92	-0.59	3.26	0.15	4.83	15.86	15.58	0.28
C60ISO	98.25	-5.61	5.73	0.06	7.60	15.12	14.64	0.48
L7	18.20	3.03	3.03	0.17	3.78	6.76	6.37	0.39
UPU23	5.72	-0.14	0.38	0.07	0.47	1.11	1.08	0.03
ENZYMES23	15.32	-3.81	8.32	0.54	14.58	49.48	49.20	0.28

S5.9 Dispersion-Uncorrected (meta-)GGAs

Table S131: Statistical analysis for BLYP for all testset in our databsase. The numbers given (all in kcal/mol) are average reaction energy ($\vert\overline{\Delta E}\vert$), mean deviation (MD), mean absolute deviation (MAD), MAD normalized with respect to $\vert\overline{\Delta E}\vert$ (NMAD), root-mean-square deviation (RMSD), deviation span (Δ_{err}), maximum (max) and minimum deviation (min).

Test set	$\vert\overline{\Delta E}\vert$	MD	MAD	NMAD	RMSD	Δ_{err}	max	min
FH51	31.01	6.53	6.53	0.21	8.39	24.76	24.58	0.18
YBDE18	49.28	-10.94	11.84	0.24	14.37	26.52	25.69	0.83
AL2X6	35.88	-11.88	11.88	0.33	12.48	24.23	17.56	6.67
DARC	32.47	22.36	22.36	0.69	22.64	41.38	25.82	15.56
NBPRC	27.71	6.15	8.00	0.29	10.84	26.25	26.11	0.14
HEAVYSB9	58.02	-9.64	9.64	0.17	10.47	20.76	16.24	4.52

Continued on next page

Test set	$\lvert\overline{\Delta E}\rvert$	MD	MAD	NMAD	RMSD	Δ_{err}	max	min
BSR36	16.20	-11.27	11.27	0.70	12.89	32.29	29.67	2.62
RSE43	7.60	-2.90	2.90	0.38	3.26	8.26	7.57	0.69
W4-11	306.91	2.79	6.77	0.02	9.57	35.92	35.92	0.00
G21EA	33.62	-4.12	4.67	0.14	5.16	10.66	9.52	1.14
G21IP	257.61	-1.53	4.61	0.02	5.52	13.17	12.81	0.36
DIPCS10	654.26	-5.00	6.97	0.01	8.06	12.27	12.19	0.08
PA26	189.05	0.82	2.42	0.01	3.11	8.32	8.05	0.27
SIE4x4	33.72	24.20	24.20	0.72	27.70	55.30	52.44	2.86
ALKBDE10	100.69	52.74	52.74	0.52	59.00	106.52	88.12	18.40
RC21	35.70	-1.72	3.93	0.11	5.78	17.38	17.04	0.34
ALK8	62.60	-8.40	8.40	0.13	11.21	22.82	21.86	0.96
DC13	54.98	7.88	22.65	0.41	27.46	48.26	46.53	1.73
G2RC	51.26	4.99	5.99	0.12	7.54	15.83	15.71	0.12
BH76RC	21.39	-0.06	3.04	0.14	3.93	10.63	10.62	0.01
MOR23	35.57	11.25	12.97	0.36	15.27	28.32	27.17	1.15
WCPT18	34.99	4.34	4.34	0.12	4.70	8.97	7.77	1.20
BHROT27	6.37	0.44	0.44	0.07	0.56	1.33	1.32	0.01
BHPERI	20.87	4.59	4.59	0.22	5.32	12.00	11.77	0.23
BHDIV10	45.33	5.27	5.27	0.12	6.25	11.11	10.97	0.14
INV24	32.85	3.47	3.47	0.11	4.03	9.57	8.58	0.99
CR20	19.31	-15.47	15.47	0.80	15.61	31.25	19.81	11.44
CRBH20	46.13	-16.99	16.99	0.37	17.06	33.84	19.93	13.91
TMBH17	12.76	0.52	5.51	0.43	6.78	18.30	17.45	0.85
LTMBH26	9.98	-3.99	5.44	0.55	6.92	16.19	15.91	0.28
BH76	18.61	-8.79	8.81	0.47	10.02	24.00	23.95	0.05
ISO34	14.57	3.36	3.36	0.23	4.73	13.27	13.23	0.04
ICONF	3.27	0.68	0.68	0.21	0.92	2.14	2.11	0.03
ACONF	1.83	1.05	1.05	0.57	1.17	2.55	2.15	0.40
TAUT15	3.05	1.64	1.64	0.54	1.88	2.95	2.94	0.01
Amino20x4	2.44	0.77	0.77	0.31	0.97	2.48	2.46	0.02
PCONF	1.62	4.62	4.62	2.85	5.15	8.75	8.13	0.62
MCONF	4.97	2.99	2.99	0.60	3.33	5.02	4.96	0.06
SCONF	4.60	0.51	0.51	0.11	0.67	1.44	1.44	0.00
PArel	4.63	-0.48	1.73	0.37	2.74	8.31	8.30	0.01
BUT14DIOL	2.80	-0.09	0.40	0.14	0.49	1.15	1.11	0.04
EIE22	5.44	2.00	2.00	0.37	2.20	3.81	3.79	0.02
Styrene45	62.64	2.62	7.53	0.12	10.22	25.17	25.17	0.00
ISOMERIZATION20	31.84	0.86	3.31	0.10	4.63	12.04	11.97	0.07
DIE60	4.71	1.69	1.70	0.36	1.86	3.73	3.62	0.11
IDISP	14.22	5.97	19.19	1.35	23.05	49.49	45.20	4.29
C20C24	30.77	5.54	63.79	2.07	66.25	137.98	91.87	46.11
S66	5.47	-4.26	4.26	0.78	4.80	12.32	11.30	1.02
S10x8	6.59	2.39	2.39	0.36	3.12	11.85	11.72	0.13
X40	3.76	2.67	2.67	0.71	3.19	10.19	9.62	0.57

Continued on next page

Test set	$\lvert\overline{\Delta E}\rvert$	MD	MAD	NMAD	RMSD	Δ_{err}	max	min
HEAVY28	1.24	-1.49	1.49	1.20	1.59	3.20	2.68	0.52
CHB6	26.79	2.43	2.43	0.09	2.94	5.75	5.29	0.46
AHB21	22.49	0.84	0.92	0.04	1.25	3.26	3.21	0.05
IL16	109.04	3.62	3.62	0.03	3.80	7.53	5.97	1.56
PNICO23	4.27	-2.22	2.22	0.52	2.35	5.71	4.37	1.34
CT20	0.98	0.92	0.92	0.93	0.97	1.60	1.38	0.22
CARBHB12	6.04	-0.44	0.79	0.13	0.96	1.63	1.61	0.02
ADIM6	3.36	-5.84	5.84	1.74	6.38	12.05	9.73	2.32
3B-69-TRIM	12.30	7.92	7.92	0.64	8.57	18.85	16.79	2.06
ISOL24	21.92	-7.40	13.52	0.62	20.63	68.51	68.33	0.18
C60ISO	98.25	-9.34	9.34	0.10	11.14	20.29	19.15	1.14
L7	18.20	26.74	26.74	1.47	29.83	59.99	49.62	10.37
UPU23	5.72	2.36	2.90	0.51	4.27	11.62	11.55	0.07
ENZYMES23	15.32	-5.27	7.09	0.46	8.36	15.44	15.02	0.42

Table S132: Statistical analysis for PBE for all testset in our databsase. The numbers given (all in kcal/mol) are average reaction energy ($\lvert\overline{\Delta E}\rvert$), mean deviation (MD), mean absolute deviation (MAD), MAD normalized with respect to $\lvert\overline{\Delta E}\rvert$ (NMAD), root-mean-square deviation (RMSD), deviation span (Δ_{err}), maximum (max) and minimum deviation (min).

Test set	$\lvert\overline{\Delta E}\rvert$	MD	MAD	NMAD	RMSD	Δ_{err}	max	min
FH51	31.01	1.82	3.41	0.11	4.67	12.62	12.58	0.04
YBDE18	49.28	-3.13	6.06	0.12	7.31	14.52	13.65	0.87
AL2X6	35.88	-4.45	4.45	0.12	5.24	7.72	7.59	0.13
DARC	32.47	6.40	6.49	0.20	7.53	10.65	10.28	0.37
NBPRC	27.71	0.12	2.76	0.10	3.46	6.06	6.03	0.03
HEAVYSB9	58.02	-3.46	4.19	0.07	5.08	9.92	9.39	0.53
BSR36	16.20	-7.41	7.41	0.46	8.59	21.78	19.87	1.91
RSE43	7.60	-2.90	2.90	0.38	3.25	8.20	7.65	0.55
W4-11	306.91	11.98	13.44	0.04	16.84	51.99	51.90	0.09
G21EA	33.62	-2.30	3.27	0.10	4.08	9.29	8.85	0.44
G21IP	257.61	0.37	3.80	0.01	4.81	10.91	10.87	0.04
DIPCS10	654.26	-1.26	3.70	0.01	5.00	11.16	10.86	0.30
PA26	189.05	0.32	1.67	0.01	2.28	6.43	6.36	0.07
SIE4x4	33.72	27.31	27.31	0.81	29.17	57.63	46.85	10.78
ALKBDE10	100.69	54.69	54.69	0.54	60.92	114.93	91.54	23.39
RC21	35.70	4.45	5.46	0.15	6.36	13.00	12.32	0.68
ALK8	62.60	0.70	2.70	0.04	3.41	7.66	6.98	0.68
DC13	54.98	1.98	10.86	0.20	13.69	29.40	29.28	0.12
G2RC	51.26	0.90	6.09	0.12	7.52	20.15	19.94	0.21
BH76RC	21.39	0.15	3.53	0.17	4.58	14.07	14.01	0.06
MOR23	35.57	3.99	6.71	0.19	7.82	15.41	13.63	1.78

Continued on next page

Test set	$\overline{\|\Delta E\|}$	MD	MAD	NMAD	RMSD	Δ_{err}	max	min
WCPT18	34.99	-8.76	8.76	0.25	9.13	20.45	15.39	5.06
BHROT27	6.37	0.31	0.47	0.07	0.64	1.69	1.67	0.02
BHPERI	20.87	-3.82	3.82	0.18	4.28	9.35	8.32	1.03
BHDIV10	45.33	-7.55	8.08	0.18	9.08	15.20	13.90	1.30
INV24	32.85	-1.72	3.27	0.10	4.43	15.93	15.38	0.55
CR20	19.31	-3.18	3.18	0.16	3.63	6.15	6.15	0.00
CRBH20	46.13	-8.05	8.05	0.17	8.18	16.43	11.13	5.30
TMBH17	12.76	-1.47	4.44	0.35	5.31	10.25	10.11	0.14
LTMBH26	9.98	-3.15	4.40	0.44	5.36	13.35	13.13	0.22
BH76	18.61	-9.53	9.56	0.51	10.85	31.49	30.63	0.86
ISO34	14.57	-0.89	1.82	0.13	2.53	7.45	7.41	0.04
ICONF	3.27	0.22	0.46	0.14	0.63	1.88	1.83	0.05
ACONF	1.83	0.58	0.58	0.32	0.66	1.43	1.24	0.19
TAUT15	3.05	0.33	1.83	0.60	2.29	4.84	4.81	0.03
Amino20x4	2.44	-0.01	0.52	0.21	0.66	1.81	1.81	0.00
PCONF	1.62	-0.80	3.51	2.17	3.81	5.97	5.58	0.39
MCONF	4.97	-1.59	1.70	0.34	1.94	3.40	3.32	0.08
SCONF	4.60	0.26	0.43	0.09	0.60	1.30	1.30	0.00
PArel	4.63	0.23	1.83	0.40	2.55	6.66	6.63	0.03
BUT14DIOL	2.80	0.22	0.28	0.10	0.39	1.22	1.21	0.01
EIE22	5.44	1.97	2.00	0.37	2.24	3.92	3.80	0.12
Styrene45	62.64	-1.06	2.99	0.05	4.15	14.72	14.72	0.00
ISOMERIZATION20	31.84	0.79	3.30	0.10	4.64	12.38	12.35	0.03
DIE60	4.71	1.94	1.94	0.41	2.11	3.83	3.78	0.05
IDISP	14.22	2.99	10.79	0.76	12.62	26.13	23.66	2.47
C20C24	30.77	-13.70	23.09	0.75	32.85	67.36	63.07	4.29
S66	5.47	-2.30	2.30	0.42	2.79	7.54	7.49	0.05
S10x8	6.59	1.05	1.05	0.16	1.63	7.15	7.14	0.01
X40	3.76	1.20	1.28	0.34	1.77	6.36	6.36	0.00
HEAVY28	1.24	-0.37	0.48	0.39	0.56	1.27	1.18	0.09
CHB6	26.79	1.23	1.23	0.05	1.50	3.29	2.87	0.42
AHB21	22.49	-1.26	1.48	0.07	1.77	3.74	3.64	0.10
IL16	109.04	0.42	1.13	0.01	1.29	2.43	2.42	0.01
PNICO23	4.27	-0.07	0.79	0.19	1.19	4.39	4.32	0.07
CT20	0.98	0.24	0.36	0.36	0.41	0.69	0.68	0.01
CARBHB12	6.04	1.23	1.23	0.20	1.69	3.99	3.87	0.12
ADIM6	3.36	-3.19	3.19	0.95	3.56	6.62	5.63	0.99
3B-69-TRIM	12.30	4.35	4.36	0.35	4.99	11.02	10.99	0.03
ISOL24	21.92	-3.50	6.89	0.31	10.52	34.03	33.62	0.41
C60ISO	98.25	-9.84	9.84	0.10	11.85	21.76	20.63	1.13
L7	18.20	19.13	19.13	1.05	21.83	44.21	37.16	7.05
UPU23	5.72	1.51	1.93	0.34	2.85	7.63	7.63	0.00
ENZYMES23	15.32	-3.81	5.48	0.36	6.34	13.99	11.91	2.08

Table S133: Statistical analysis for REVPBE for all testset in our databsase. The numbers given (all in kcal/mol) are average reaction energy ($|\overline{\Delta E}|$), mean deviation (MD), mean absolute deviation (MAD), MAD normalized with respect to $|\overline{\Delta E}|$ (NMAD), root-mean-square deviation (RMSD), deviation span (Δ_{err}), maximum (max) and minimum deviation (min).

Test set	$\lvert\overline{\Delta E}\rvert$	MD	MAD	NMAD	RMSD	Δ_{err}	max	min
FH51	31.01	4.04	5.00	0.16	6.49	17.11	17.01	0.10
YBDE18	49.28	-2.27	3.31	0.07	3.52	6.04	4.94	1.10
AL2X6	35.88	0.71	2.09	0.06	2.32	4.29	3.59	0.70
DARC	32.47	13.76	13.76	0.42	14.31	24.49	17.61	6.88
NBPRC	27.71	-0.30	2.63	0.10	2.89	5.16	4.25	0.91
HEAVYSB9	58.02	-8.52	8.52	0.15	9.33	17.45	14.24	3.21
BSR36	16.20	-10.63	10.63	0.66	12.01	28.83	27.22	1.61
RSE43	7.60	-2.48	2.48	0.33	2.78	7.57	7.07	0.50
W4-11	306.91	-0.99	7.24	0.02	9.74	31.90	31.84	0.06
G21EA	33.62	-3.65	4.17	0.12	5.28	10.33	10.32	0.01
G21IP	257.61	-0.61	3.97	0.02	4.84	9.41	9.27	0.14
DIPCS10	654.26	-2.52	3.75	0.01	5.02	10.59	10.47	0.12
PA26	189.05	2.48	2.71	0.01	3.62	9.32	9.31	0.01
SIE4x4	33.72	22.14	22.14	0.66	25.39	51.88	48.37	3.51
ALKBDE10	100.69	0.37	5.26	0.05	7.55	21.11	20.53	0.58
RC21	35.70	0.17	2.89	0.08	3.63	7.78	7.55	0.23
ALK8	62.60	-3.46	3.66	0.06	5.45	13.38	13.29	0.09
DC13	54.98	5.81	15.64	0.28	17.83	33.01	30.74	2.27
G2RC	51.26	3.71	4.96	0.10	6.87	19.49	19.26	0.23
BH76RC	21.39	0.30	2.37	0.11	3.40	10.91	10.88	0.03
MOR23	35.57	12.80	13.49	0.38	18.46	62.94	62.88	0.06
WCPT18	34.99	-5.44	5.44	0.16	5.94	11.09	9.74	1.35
BHROT27	6.37	0.10	0.37	0.06	0.49	1.23	1.18	0.05
BHPERI	20.87	0.25	2.98	0.14	3.45	7.05	6.99	0.06
BHDIV10	45.33	-5.47	5.94	0.13	7.02	11.91	11.09	0.82
INV24	32.85	-2.93	3.28	0.10	3.81	8.89	8.36	0.53
CR20	19.31	3.19	3.19	0.17	3.57	5.69	5.58	0.11
CRBH20	46.13	-4.27	4.27	0.09	4.49	8.58	6.67	1.91
TMBH17	12.76	0.16	3.67	0.29	4.47	9.98	9.50	0.48
LTMBH26	9.98	-3.48	4.69	0.47	5.90	14.49	14.19	0.30
BH76	18.61	-7.34	7.41	0.40	8.47	24.79	23.90	0.89
ISO34	14.57	-1.18	2.26	0.16	3.31	10.87	10.81	0.06
ICONF	3.27	0.12	0.70	0.21	0.94	2.54	2.50	0.04
ACONF	1.83	1.18	1.18	0.64	1.32	2.70	2.28	0.42
TAUT15	3.05	-0.10	1.46	0.48	1.65	2.71	2.61	0.10
Amino20x4	2.44	-0.25	0.81	0.33	0.99	2.39	2.37	0.02
PCONF	1.62	-0.96	5.02	3.10	5.58	9.75	8.94	0.81
MCONF	4.97	-2.91	2.98	0.60	3.36	5.19	5.11	0.08

Continued on next page

Test set	$\lvert\overline{\Delta E}\rvert$	MD	MAD	NMAD	RMSD	Δ_{err}	max	min
SCONF	4.60	-0.39	0.91	0.20	1.16	2.40	2.37	0.03
PArel	4.63	0.03	1.49	0.32	2.40	7.42	7.34	0.08
BUT14DIOL	2.80	-0.68	0.83	0.30	0.92	1.64	1.64	0.00
EIE22	5.44	1.91	1.94	0.36	2.15	3.88	3.85	0.03
Styrene45	62.64	-1.51	4.10	0.07	5.46	18.30	18.30	0.00
ISOMERIZATION20	31.84	-1.88	6.85	0.22	13.02	52.74	52.70	0.04
DIE60	4.71	1.96	1.96	0.42	2.16	4.23	4.18	0.05
IDISP	14.22	4.23	17.69	1.24	20.09	39.24	34.75	4.49
C20C24	30.77	-18.38	18.38	0.60	21.38	46.50	38.92	7.58
S66	5.47	-4.69	4.69	0.86	5.17	13.24	12.16	1.08
S10x8	6.59	2.83	2.83	0.43	3.61	11.95	11.86	0.09
X40	3.76	2.93	2.93	0.78	3.39	10.49	9.89	0.60
HEAVY28	1.24	-0.31	0.47	0.38	0.59	1.31	1.31	0.00
CHB6	26.79	3.00	3.00	0.11	3.22	7.27	5.32	1.95
AHB21	22.49	1.31	1.43	0.06	1.80	4.12	4.03	0.09
IL16	109.04	4.04	4.04	0.04	4.31	8.48	7.00	1.48
PNICO23	4.27	-1.98	2.04	0.48	2.21	4.77	4.04	0.73
CT20	0.98	1.08	1.08	1.09	1.11	2.17	1.51	0.66
CARBHB12	6.04	-0.54	0.94	0.16	1.08	2.19	1.87	0.32
ADIM6	3.36	-6.04	6.04	1.80	6.65	12.49	10.29	2.20
3B-69-TRIM	12.30	8.90	8.90	0.72	9.49	20.23	17.81	2.42
ISOL24	21.92	-5.83	9.88	0.45	15.59	55.76	55.46	0.30
C60ISO	98.25	-10.02	10.02	0.10	11.97	21.95	20.66	1.29
L7	18.20	27.83	27.83	1.53	30.88	61.81	51.42	10.39
UPU23	5.72	2.23	2.99	0.52	4.37	12.36	12.34	0.02
ENZYMES23	15.32	-4.15	6.50	0.42	7.67	14.27	14.24	0.03

Table S134: Statistical analysis for TPSS for all testset in our databsase. The numbers given (all in kcal/mol) are average reaction energy ($\lvert\overline{\Delta E}\rvert$), mean deviation (MD), mean absolute deviation (MAD), MAD normalized with respect to $\lvert\overline{\Delta E}\rvert$ (NMAD), root-mean-square deviation (RMSD), deviation span (Δ_{err}), maximum (max) and minimum deviation (min).

Test set	$\lvert\overline{\Delta E}\rvert$	MD	MAD	NMAD	RMSD	Δ_{err}	max	min
FH51	31.01	3.71	5.04	0.16	6.39	14.90	14.84	0.06
YBDE18	49.28	-8.09	8.11	0.16	9.13	14.99	14.86	0.13
AL2X6	35.88	-4.04	4.11	0.11	4.93	7.01	6.83	0.18
DARC	32.47	11.55	11.55	0.36	12.04	19.92	14.46	5.46
NBPRC	27.71	0.69	2.93	0.11	4.01	9.13	8.58	0.55
HEAVYSB9	58.02	-5.15	5.15	0.09	6.22	10.97	10.89	0.08
BSR36	16.20	-10.08	10.08	0.62	11.38	28.34	26.12	2.22
RSE43	7.60	-2.00	2.00	0.26	2.31	6.61	6.09	0.52
W4-11	306.91	1.52	4.26	0.01	5.84	23.33	23.29	0.04

Continued on next page

Test set	$\|\overline{\Delta E}\|$	MD	MAD	NMAD	RMSD	Δ_{err}	max	min
G21EA	33.62	-3.89	4.45	0.13	5.75	11.48	11.43	0.05
G21IP	257.61	-0.28	3.73	0.01	4.59	11.75	11.48	0.27
DIPCS10	654.26	-2.09	2.83	0.00	3.74	7.80	7.34	0.46
PA26	189.05	3.15	3.18	0.02	4.10	10.80	10.63	0.17
SIE4x4	33.72	20.89	20.89	0.62	24.06	49.16	46.16	3.00
ALKBDE10	100.69	49.68	49.68	0.49	55.55	108.20	86.17	22.03
RC21	35.70	1.59	3.24	0.09	3.92	8.90	8.40	0.50
ALK8	62.60	-0.40	1.36	0.02	1.71	3.77	3.55	0.22
DC13	54.98	2.62	13.51	0.25	16.56	37.15	36.10	1.05
G2RC	51.26	3.61	6.19	0.12	8.41	20.71	20.54	0.17
BH76RC	21.39	-0.00	3.24	0.15	4.37	9.99	9.97	0.02
MOR23	35.57	4.40	6.58	0.18	7.81	18.20	17.35	0.85
WCPT18	34.99	-5.58	5.58	0.16	5.86	11.88	9.17	2.71
BHROT27	6.37	0.29	0.54	0.08	0.70	1.65	1.61	0.04
BHPERI	20.87	-1.92	2.17	0.10	2.82	6.66	6.45	0.21
BHDIV10	45.33	-5.62	5.96	0.13	6.64	11.98	10.24	1.74
INV24	32.85	-2.17	2.75	0.08	3.14	7.07	6.68	0.39
CR20	19.31	-5.94	5.94	0.31	6.31	10.35	9.65	0.70
CRBH20	46.13	-10.31	10.31	0.22	10.36	21.18	12.36	8.82
TMBH17	12.76	-0.77	3.94	0.31	5.02	10.01	9.99	0.02
LTMBH26	9.98	-2.60	4.05	0.41	5.26	15.93	15.61	0.32
BH76	18.61	-8.80	8.81	0.47	9.86	22.61	22.44	0.17
ISO34	14.57	-1.83	2.65	0.18	3.68	13.20	13.14	0.06
ICONF	3.27	0.07	0.40	0.12	0.51	1.47	1.39	0.08
ACONF	1.83	0.68	0.68	0.37	0.77	1.59	1.37	0.22
TAUT15	3.05	0.18	1.63	0.54	1.90	3.28	3.23	0.05
Amino20x4	2.44	-0.03	0.54	0.22	0.69	1.92	1.92	0.00
PCONF	1.62	-0.85	3.96	2.44	4.31	6.73	6.13	0.60
MCONF	4.97	-2.03	2.11	0.43	2.40	3.96	3.92	0.04
SCONF	4.60	0.25	0.49	0.11	0.62	1.26	1.24	0.02
PArel	4.63	0.07	1.55	0.34	2.24	6.18	6.13	0.05
BUT14DIOL	2.80	-0.12	0.30	0.11	0.35	0.74	0.73	0.01
EIE22	5.44	2.12	2.12	0.39	2.31	3.97	3.94	0.03
Styrene45	62.64	-0.22	3.63	0.06	4.58	15.29	15.29	0.00
ISOMERIZATION20	31.84	1.52	3.44	0.11	4.70	11.11	11.04	0.07
DIE60	4.71	1.96	1.96	0.42	2.10	3.95	3.60	0.35
IDISP	14.22	3.60	12.87	0.90	15.40	32.89	29.96	2.93
C20C24	30.77	-12.81	34.67	1.13	43.78	94.23	81.27	12.96
S66	5.47	-3.25	3.25	0.59	3.75	9.90	9.37	0.53
S10x8	6.59	1.64	1.64	0.25	2.22	8.56	8.48	0.08
X40	3.76	1.90	1.90	0.51	2.39	7.81	7.76	0.05
HEAVY28	1.24	-0.81	0.82	0.66	0.92	1.78	1.66	0.12
CHB6	26.79	1.78	1.78	0.07	2.09	4.53	3.83	0.70
AHB21	22.49	-0.35	1.02	0.05	1.20	2.54	2.33	0.21

Continued on next page

Test set	$\lvert\overline{\Delta E}\rvert$	MD	MAD	NMAD	RMSD	Δ_{err}	max	min
IL16	109.04	2.13	2.13	0.02	2.37	4.51	4.26	0.25
PNICO23	4.27	-0.72	0.95	0.22	1.14	3.07	3.03	0.04
CT20	0.98	0.66	0.66	0.67	0.71	1.19	1.07	0.12
CARBHB12	6.04	0.44	0.67	0.11	1.02	2.77	2.65	0.12
ADIM6	3.36	-4.51	4.51	1.34	4.99	9.31	7.76	1.55
3B-69-TRIM	12.30	6.11	6.11	0.50	6.76	14.92	13.99	0.93
ISOL24	21.92	-5.20	9.00	0.41	13.57	45.28	45.22	0.06
C60ISO	98.25	-8.22	8.22	0.08	10.11	18.67	18.09	0.58
L7	18.20	22.72	22.72	1.25	25.45	51.28	42.62	8.66
UPU23	5.72	1.83	2.31	0.40	3.47	9.86	9.79	0.07
ENZYMES23	15.32	-4.57	6.24	0.41	7.24	13.35	12.81	0.54

Table S135: Statistical analysis for revTPSS for all testset in our databsase. The numbers given (all in kcal/mol) are average reaction energy ($\lvert\overline{\Delta E}\rvert$), mean deviation (MD), mean absolute deviation (MAD), MAD normalized with respect to $\lvert\overline{\Delta E}\rvert$ (NMAD), root-mean-square deviation (RMSD), deviation span (Δ_{err}), maximum (max) and minimum deviation (min).

Test set	$\lvert\overline{\Delta E}\rvert$	MD	MAD	NMAD	RMSD	Δ_{err}	max	min
FH51	31.01	5.69	5.69	0.18	7.29	20.01	19.93	0.08
YBDE18	49.28	-6.94	6.97	0.14	7.81	13.05	12.81	0.24
AL2X6	35.88	-2.74	2.88	0.08	3.44	5.50	5.13	0.37
DARC	32.47	7.78	7.78	0.24	8.32	13.00	10.43	2.57
NBPRC	27.71	0.98	2.68	0.10	3.70	7.84	7.83	0.01
HEAVYSB9	58.02	-3.46	3.98	0.07	4.78	9.55	8.77	0.78
BSR36	16.20	-7.29	7.29	0.45	8.17	19.57	18.40	1.17
RSE43	7.60	-1.73	1.73	0.23	2.06	6.04	5.68	0.36
W4-11	306.91	0.68	4.70	0.02	6.44	24.19	24.19	0.00
G21EA	33.62	-4.71	5.14	0.15	6.52	13.34	13.11	0.23
G21IP	257.61	-0.95	3.67	0.01	4.56	11.23	11.16	0.07
DIPCS10	654.26	-3.04	3.17	0.00	4.36	9.03	9.00	0.03
PA26	189.05	3.09	3.22	0.02	4.21	11.09	10.79	0.30
SIE4x4	33.72	20.40	20.40	0.60	23.53	47.53	44.85	2.68
ALKBDE10	100.69	-0.63	4.57	0.05	6.54	18.11	17.64	0.47
RC21	35.70	2.14	3.76	0.11	4.56	8.73	8.72	0.01
ALK8	62.60	-1.01	1.57	0.03	1.83	2.95	2.88	0.07
DC13	54.98	1.49	10.58	0.19	14.97	39.79	39.44	0.35
G2RC	51.26	5.85	9.62	0.19	12.61	28.10	28.09	0.01
BH76RC	21.39	0.37	4.48	0.21	5.62	13.28	13.23	0.05
MOR23	35.57	5.62	7.33	0.21	14.29	61.72	61.65	0.07
WCPT18	34.99	4.83	4.83	0.14	5.18	9.55	8.05	1.50
BHROT27	6.37	0.51	0.51	0.08	0.68	1.55	1.55	0.00
BHPERI	20.87	2.98	2.98	0.14	3.38	7.33	7.24	0.09

Continued on next page

Test set	$\lvert\overline{\Delta E}\rvert$	MD	MAD	NMAD	RMSD	Δ_{err}	max	min
BHDIV10	45.33	5.39	5.39	0.12	6.06	10.72	10.11	0.61
INV24	32.85	2.23	2.23	0.07	2.66	6.21	5.84	0.37
CR20	19.31	-2.14	2.68	0.14	2.96	5.35	4.88	0.47
CRBH20	46.13	-8.89	8.89	0.19	8.94	17.12	10.24	6.88
TMBH17	12.76	-1.04	3.92	0.31	4.84	10.23	9.87	0.36
LTMBH26	9.98	-3.22	4.40	0.44	5.43	15.29	14.99	0.30
BH76	18.61	-10.69	10.69	0.57	12.91	37.49	37.40	0.09
ISO34	14.57	3.09	3.09	0.21	4.37	16.50	16.41	0.09
ICONF	3.27	0.30	0.30	0.09	0.37	0.96	0.89	0.07
ACONF	1.83	0.54	0.54	0.30	0.61	1.21	1.05	0.16
TAUT15	3.05	1.30	1.30	0.43	1.68	3.47	3.46	0.01
Amino20x4	2.44	0.43	0.43	0.18	0.54	1.54	1.54	0.00
PCONF	1.62	3.09	3.09	1.91	3.37	5.31	4.84	0.47
MCONF	4.97	1.83	1.83	0.37	2.03	3.39	3.32	0.07
SCONF	4.60	0.69	0.69	0.15	0.77	1.28	1.23	0.05
PArel	4.63	0.32	1.51	0.33	2.04	5.60	5.54	0.06
BUT14DIOL	2.80	-0.25	0.32	0.11	0.37	0.74	0.72	0.02
EIE22	5.44	1.99	1.99	0.37	2.14	3.75	3.67	0.08
Styrene45	62.64	-0.98	3.37	0.05	4.24	12.90	12.90	0.00
ISOMERIZATION20	31.84	2.08	3.97	0.12	5.35	12.31	12.30	0.01
DIE60	4.71	1.78	1.78	0.38	1.86	3.72	3.13	0.59
IDISP	14.22	8.84	8.84	0.62	11.58	26.64	24.19	2.45
C20C24	30.77	-18.92	21.05	0.68	34.25	68.68	68.36	0.32
S66	5.47	-2.91	2.91	0.53	3.31	8.72	8.08	0.64
S10x8	6.59	1.57	1.57	0.24	2.03	7.08	6.99	0.09
X40	3.76	1.71	1.71	0.46	2.11	7.26	6.91	0.35
HEAVY28	1.24	-0.68	0.68	0.55	0.77	1.47	1.39	0.08
CHB6	26.79	1.63	1.63	0.06	1.89	3.80	3.35	0.45
AHB21	22.49	-0.00	0.69	0.03	0.81	2.00	1.93	0.07
IL16	109.04	1.98	1.98	0.02	2.15	4.08	3.46	0.62
PNICO23	4.27	-0.47	0.78	0.18	0.95	2.81	2.63	0.18
CT20	0.98	0.61	0.61	0.62	0.66	1.06	1.03	0.03
CARBHB12	6.04	0.15	0.52	0.09	0.70	1.95	1.86	0.09
ADIM6	3.36	-3.98	3.98	1.18	4.39	8.18	6.75	1.43
3B-69-TRIM	12.30	5.70	5.70	0.46	6.23	13.20	12.15	1.05
ISOL24	21.92	-4.60	7.79	0.36	11.00	35.53	35.39	0.14
C60ISO	98.25	-9.09	9.09	0.09	11.10	20.51	19.70	0.81
L7	18.20	20.40	20.40	1.12	22.75	45.70	37.87	7.83
UPU23	5.72	1.49	2.02	0.35	3.00	8.51	8.47	0.04
ENZYMES23	15.32	-4.18	6.14	0.40	7.01	13.54	12.23	1.31

Table S136: Statistical analysis for SCAN for all testset in our databsase. The numbers given (all in kcal/mol) are average reaction energy ($|\overline{\Delta E}|$), mean deviation (MD), mean absolute deviation (MAD), MAD normalized with respect to $|\overline{\Delta E}|$ (NMAD), root-mean-square deviation (RMSD), deviation span (Δ_{err}), maximum (max) and minimum deviation (min).

Test set	$\lvert\overline{\Delta E}\rvert$	MD	MAD	NMAD	RMSD	Δ_{err}	max	min
FH51	31.01	2.64	2.64	0.09	3.94	10.90	10.82	0.08
YBDE18	49.28	-4.00	4.49	0.09	4.79	9.04	7.54	1.50
AL2X6	35.88	1.15	1.57	0.04	1.75	3.63	2.93	0.70
DARC	32.47	-0.15	2.69	0.08	2.88	5.69	4.92	0.77
NBPRC	27.71	-0.53	1.54	0.06	1.98	3.51	3.47	0.04
HEAVYSB9	58.02	-3.18	3.18	0.05	3.76	6.67	6.53	0.14
BSR36	16.20	-2.56	2.56	0.16	3.12	8.54	7.97	0.57
RSE43	7.60	-1.21	1.23	0.16	1.79	5.72	5.68	0.04
W4-11	306.91	-1.85	3.57	0.01	4.86	27.00	26.99	0.01
G21EA	33.62	-3.61	5.47	0.16	6.69	13.68	13.32	0.36
G21IP	257.61	0.20	4.69	0.02	5.67	12.28	12.21	0.07
DIPCS10	654.26	-1.49	4.87	0.01	5.68	13.58	12.14	1.44
PA26	189.05	1.88	2.22	0.01	3.27	9.76	9.60	0.16
SIE4x4	33.72	2.38	25.51	0.76	32.29	88.50	85.94	2.56
ALKBDE10	100.69	1.01	5.57	0.06	7.54	20.42	19.62	0.80
RC21	35.70	4.67	5.28	0.15	5.87	10.84	10.07	0.77
ALK8	62.60	2.27	2.98	0.05	3.97	7.27	7.17	0.10
DC13	54.98	0.07	8.29	0.15	11.11	31.45	29.25	2.20
G2RC	51.26	-1.17	6.00	0.12	7.43	17.59	17.31	0.28
BH76RC	21.39	-0.49	2.92	0.14	3.87	8.91	8.87	0.04
MOR23	35.57	0.87	6.00	0.17	13.23	59.40	59.30	0.10
WCPT18	34.99	6.40	6.40	0.18	7.11	15.01	12.67	2.34
BHROT27	6.37	0.79	0.82	0.13	1.15	2.40	2.39	0.01
BHPERI	20.87	4.85	4.85	0.23	5.13	8.57	7.88	0.69
BHDIV10	45.33	-5.39	6.36	0.14	6.93	12.64	11.05	1.59
INV24	32.85	1.25	1.25	0.04	1.79	6.24	6.21	0.03
CR20	19.31	0.12	1.09	0.06	1.33	3.03	2.95	0.08
CRBH20	46.13	-6.55	6.55	0.14	6.59	13.49	8.02	5.47
TMBH17	12.76	-1.35	4.67	0.37	5.40	8.64	8.13	0.51
LTMBH26	9.98	-3.67	4.88	0.49	5.72	15.57	14.16	1.41
BH76	18.61	-7.97	8.06	0.43	8.82	18.70	18.22	0.48
ISO34	14.57	1.39	1.39	0.10	2.00	6.39	6.38	0.01
ICONF	3.27	0.36	0.36	0.11	0.47	1.09	1.08	0.01
ACONF	1.83	0.30	0.30	0.16	0.33	0.70	0.60	0.10
TAUT15	3.05	0.40	1.79	0.59	2.26	5.08	4.99	0.09
Amino20x4	2.44	0.13	0.27	0.11	0.36	1.05	1.05	0.00
PCONF	1.62	0.78	0.78	0.48	0.86	1.26	1.23	0.03
MCONF	4.97	0.44	0.44	0.09	0.51	0.96	0.96	0.00

Continued on next page

Test set	$\|\overline{\Delta E}\|$	MD	MAD	NMAD	RMSD	Δ_{err}	max	min
SCONF	4.60	0.78	0.78	0.17	0.84	1.43	1.40	0.03
PArel	4.63	0.60	1.50	0.32	2.20	7.26	7.25	0.01
BUT14DIOL	2.80	0.43	0.43	0.15	0.45	0.79	0.76	0.03
EIE22	5.44	2.06	2.08	0.38	2.28	4.15	3.96	0.19
Styrene45	62.64	1.91	2.65	0.04	3.48	9.24	9.24	0.00
ISOMERIZATION20	31.84	-0.22	2.60	0.08	3.50	6.73	6.61	0.12
DIE60	4.71	1.73	1.73	0.37	1.84	2.81	2.66	0.15
IDISP	14.22	4.76	4.76	0.33	6.30	14.74	13.59	1.15
C20C24	30.77	-10.26	16.74	0.54	24.07	48.55	45.95	2.60
S66	5.47	-0.73	0.90	0.16	1.09	2.11	2.08	0.03
S10x8	6.59	0.09	0.56	0.08	0.84	3.08	3.08	0.00
X40	3.76	0.16	0.51	0.13	0.77	2.34	2.34	0.00
HEAVY28	1.24	-0.26	0.33	0.27	0.38	0.75	0.74	0.01
CHB6	26.79	1.29	1.29	0.05	1.41	2.53	2.05	0.48
AHB21	22.49	-2.38	2.38	0.11	2.87	5.43	5.32	0.11
IL16	109.04	-1.00	1.08	0.01	1.30	2.45	2.37	0.08
PNICO23	4.27	0.66	0.79	0.18	1.27	4.81	4.75	0.06
CT20	0.98	-0.01	0.22	0.22	0.28	0.94	0.90	0.04
CARBHB12	6.04	1.40	1.40	0.23	1.87	4.01	3.92	0.09
ADIM6	3.36	-1.52	1.52	0.45	1.69	3.03	2.58	0.45
3B-69-TRIM	12.30	1.36	1.72	0.14	2.03	4.77	4.75	0.02
ISOL24	21.92	-0.87	3.80	0.17	5.61	17.74	17.44	0.30
C60ISO	98.25	-5.53	5.66	0.06	7.50	14.99	14.48	0.51
L7	18.20	9.92	9.92	0.54	11.33	22.32	18.76	3.56
UPU23	5.72	0.38	0.73	0.13	1.00	2.54	2.53	0.01
ENZYMES23	15.32	-3.52	8.02	0.52	14.23	48.54	48.38	0.16
